# Gene Co-Expression in Breast Cancer: A Matter of Distance

**DOI:** 10.3389/fonc.2021.726493

**Published:** 2021-11-17

**Authors:** Alfredo González-Espinoza, Jose Zamora-Fuentes, Enrique Hernández-Lemus, Jesús Espinal-Enríquez

**Affiliations:** ^1^ Department of Biology, University of Pennsylvania, Philadelphia, PA, United States; ^2^ Computational Genomics Division, National Institute of Genomic Medicine, Mexico City, Mexico; ^3^ Centro de Ciencias de la Complejidad, Universidad Nacional Autόnoma de México, Mexico City, Mexico

**Keywords:** eigenvalue decomposition, gene co-expression clustering, loss of long-distance co-expression, co-expression matrices, breast cancer molecular subtypes

## Abstract

Gene regulatory and signaling phenomena are known to be relevant players underlying the establishment of cellular phenotypes. It is also known that such regulatory programs are disrupted in cancer, leading to the onset and development of malignant phenotypes. Gene co-expression matrices have allowed us to compare and analyze complex phenotypes such as breast cancer (BrCa) and their control counterparts. Global co-expression patterns have revealed, for instance, that the highest gene-gene co-expression interactions often occur between genes from the same chromosome (*cis-*), meanwhile inter-chromosome (*trans-*) interactions are scarce and have lower correlation values. Furthermore, strength of *cis-* correlations have been shown to decay with the chromosome distance of gene couples. Despite this *loss of long-distance co-expression* has been clearly identified, it has been observed only in a small fraction of the whole co-expression landscape, namely the most significant interactions. For that reason, an approach that takes into account the whole interaction set results appealing. In this work, we developed a hybrid method to analyze whole-chromosome Pearson correlation matrices for the four BrCa subtypes (Luminal A, Luminal B, HER2+ and Basal), as well as adjacent normal breast tissue derived matrices. We implemented a systematic method for clustering gene couples, by using eigenvalue spectral decomposition and the *k*–medoids algorithm, allowing us to determine a number of clusters without removing any interaction. With this method we compared, for each chromosome in the five phenotypes: *a)* Whether or not the gene-gene co-expression decays with the distance in the breast cancer subtypes *b)* the chromosome location of *cis-* clusters of gene couples, and *c)* whether or not the *loss of long-distance co-expression* is observed in the whole range of interactions. We found that in the correlation matrix for the control phenotype, positive and negative Pearson correlations deviate from a random null model independently of the distance between couples. Conversely, for all BrCa subtypes, in all chromosomes, positive correlations decay with distance, and negative correlations do not differ from the null model. We also found that BrCa clusters are distance-dependent, meanwhile for the control phenotype, chromosome location does not determine the clustering. To our knowledge, this is the first time that a dependence on distance is reported for gene clusters in breast cancer. Since this method uses the whole *cis-* interaction geneset, combination with other -omics approaches may provide further evidence to understand in a more integrative fashion, the mechanisms that disrupt gene regulation in cancer.

## 1 Introduction

### 1.1 Breast Cancer: A Complex Disease

Breast cancer is the first cancer-related cause of death in women worldwide. It is also, according to the most recent data (1), the most diagnosed neoplasm in the world. Breast cancer is also the malignant neoplasm with the highest incidence (1). Its diagnosis, response to treatment, relapse, and outcome are strongly determined by the molecular profile underlying the disease (2–4). The PAM50 classifier is among the most relevant methods of classification for breast cancer molecular subtypes (5). This molecular classification is based on the expression signature of 50 genes relevant to the oncogenic phenotype (5–7).

Publicly available massive cohorts of genomic and clinical data in the study of cancer, have allowed the analysis of an immeasurable amount of information. The latter has contributed to a better understanding of the oncogenic process (8). Based on gene expression of hundreds-to-thousands of samples, now it is possible to study such vast experimental information to infer and analyze the whole-genome co-expression landscape, aiming to highlight similarities and differences between cancer and non-cancer samples. Among these efforts, The Cancer Genome Atlas (TCGA) has contributed in an outstanding way (9).

### 1.2 Gene Co-Expression Networks

The study of Cancer within the framework of complex networks has become increasingly relevant in the last years (10–20). Given its size and complexity, genome-wide regulation may include a large number of features (all the genes), potentially inducing a fully connected network, with contributions of very different relevance and certainty. For this reason, several approaches to reduce its dimensionality have been implemented, including the use of threshold methods, to look for the most significant co-expression relationships (18, 21). In particular, in the case of breast cancer molecular subtype networks, the most significant co-expressed pairs have been used as connected nodes in biologically relevant modules (22–25).

Further approaches to determine the optimal network size may analyze a wide range of network scales (13, 26, 27) or backbone-related threshold networks (28), and even use gene co-expression subsets of clinical/biological relevance (29).

In the attempt of reducing the dimensionality of a fully-connected network, identification of groups of genes that behave in a similar way –indicating that their expression profiles are correlated– is a relevant problem and is still an open challenge in network biology (29, 30). The latter point is closely related to the so-called graph sparsification problem in graph theory. The choose of a significance threshold then becomes relevant.

For instance, in a recent study by Kimura et al. (31), an approach was developed to select parameters in genetic networks by computational methods (mainly Machine Learning and Artificial Intelligence). Other approaches have used the complete set of interactions in order to construct a network backbone (28). There, the authors used the complete matrix of interactions to obtain the most important relationships, preserving those edges with statistically significant deviations with respect to a null model for the local edge’s weight assignment.

### 1.3 Gene Co-Expression Is Distance Dependent

In cancer, gene co-expression networks have been used to uncover genes and relationships that may represent crucial elements to determine differences between phenotypes (32). In particular, in breast cancer and breast cancer molecular subtypes (4), gene co-expression networks have been useful to identify the phenomenon of *loss of long-range co-expression* (10, 12, 14, 33): this is, a property observed in cancer networks in which the most significant gene co-expression relationships occur between genes that belong to the same chromosome, i.e., *cis-* interactions. Conversely, inter-chromosome (*trans-*) interactions are often weak in cancer.

Furthermore, the loss of long-range co-expression is not only observed at the level of genes located on different chromosomes. Regarding *cis-* (intra-chromosome) gene interactions, there is an exponential decay of strength of correlations (14) as genes become more distant. This situation could be related to a diminishing of the *accessibility* that a certain region of the genome may have of its environment during the carcinogenic process. Importantly, this lack of accessibility can be attributed to several factors, among which we can mention aberrant expression of transcription factors, copy number alterations, incorrect binding to CTCF, or changes in Topologically Associated Domains (TADs). All of these factors have the potential to alter, both, the structure of DNA and gene expression.

Despite this phenomenon has been discovered not only in breast cancer, but also in clear cell renal carcinoma (13), lung adenocarcinoma and squamous cell lung carcinoma (12), loss of long-range co-expression has been determined for the top highest interactions: a small subset of the most co-expressed gene-gene interactions (tens-to-hundreds of thousands) of the whole co-expression landscape is observed to be biased to *cis-* interactions.

Since the strength of intra-chromosome interactions have been observed to be the highest ones, it becomes important to evaluate the behavior of the whole intra-chromosome landscape of cancer networks. In these terms, network clustering may provide us with information related to, for example, sets of genes constrained by physical restrictions in certain regions of the genome, genes that act in tandem, events related with the transcriptional process, etc.

To address the questions above, we performed a data-driven clustering analysis using a hybrid algorithm that involves eigenvalue decomposition and *k*–medoids from correlation matrices of each chromosome. These matrices were inferred from RNA-Seq-based gene expression. We evaluated whether or not the loss of long-range co-expression is preserved, by studying all chromosomes for the four breast cancer subtypes as compared with normal tumor-adjacent tissue as control.

With this approach, we constructed co-expression matrices for all chromosomes in adjacent normal breast tissue network, as well as in all four breast cancer subtypes. We analyzed the statistics for their clustering nearest neighbor distributions within each chromosome, comparing each breast cancer molecular subtype as well as the adjacent normal tissue. Additionally, for all phenotypes, we constructed a null model to provide statistical robustness to our analyses. With this, we present a systematic method for intra-chromosome gene clustering, which allows to compare the whole co-expression landscape between a cancerous phenotype with its control counterpart.

## 2 Materials and Methods

### 2.1 Data Acquisition

Gene expression data of breast invasive carcinoma was collected from The Cancer Genome Atlas (TCGA) (34). 735 tumor and 113 non-cancerous (adjacent normal), samples were considered, see **Table 1**. Illumina HiSeq RNASeq samples were filtered (biotype, expression mean >10), pre-processed, and *log*_2_ normalized gene expression values as described in (10). We performed data corrections for transcript length, GC content and RNA composition. Tumor expression values were classified using PAM50 algorithm into the respective intrinsic breast cancer sub-types (Luminal A, Luminal B, Basal, and HER2-Enriched) using the Permutation-Based Confidence for Molecular Classification (35) as implemented in the pbcmc R package (36).

**Table 1:** Samples for each subtype.

Control	Basal	Her2	LumA	LumB
113	221	105	217	192

Tumor samples with a non-reliable breast cancer sub-type call were removed from the analysis. To avoid overlapping patterns among subtype expression values, multidimensional noise reduction was performed using ARSyN R implementation (37), and a multidimensional Principal Component Analysis (PCA) was implemented to confirm noise reduction (14).

Since a crucial part of this work lies in having a highly-confident set of matrices, it is necessary to obtain as many well-characterized samples as possible, for each molecular subtype. Due to this fact, we decided to include all the available samples with a molecular subtype classification i.e., those samples with a molecular subtype label from the original source. Further investigations must be conducted with even more stringent inclusion and exclusion criteria, such as histologically confirmed diagnosis, histopathologically-assessed axillary lymph nodes, metastatic disease at presentation, adjuvant treatment, etc.

In order to provide all the information to reproduce our results, the clinical information about histological data by subtype-samples is now included in the **Supplementary Material S1**. There, for each breast cancer subtype sample we describe: 1) availability of historical adjuvant treatment, 2) lymph node assessment existence, 3) histological type of tumor and 4) axillary lymph-node-stage method type.

To show that those samples with the same molecular subtype are indeed properly classified in their molecular profiles to be included in our correlation matrices, we performed a Principal Component Analysis (PCA) for each subtype (**Supplementary Figure S1**). The PCA groups samples based on the main eigenvalues of the expression profiles. In this case, we present the two main principal components (X and Y axes of the **Supplementary Figure S1**) -though the calculations were made with the full eigenvalue spectra of the matrices. Hence, the PCA could indicate those samples that are not similar to the rest of their class (if any) or if there is any “confounded” or misclassified sample.

As it can be noticed in the **Supplementary Figure S1**, all subtype samples are clearly separated based on the molecular classification. All samples are grouped by its subtype (color). Hence, constructing correlation matrices by using these subtype-separated samples, certainly improves the statistical significance without adding a clear source of noise.

### 2.2 Correlation Matrices

We built intra-chromosomal cross-correlation matrices by estimating the Pearson correlation coefficient between the expression of two genes *i* and *j*, defined as follows:


(1) $$C_{ij}=\frac{Cov(g_{i},g_{j})}{\sigma_{gi}\sigma_{gj}}=\frac{1}{N_{s}}\sum_{s=1}^{N_{s}}\frac{\left(g_{is}-\mu_{gi})(g_{js}-\mu_{gj}\right)}{\sigma_{gi}\sigma_{gj}},$$

where *g_i_* is the set of *N_s_* expression samples for gene *i*. By definition, a correlation matrix is symmetric (*C_ij_* = *C_ji_*), the elements in the diagonal are 1 (*C_ii_* = 1, ∀*i*), and its values are bounded to –1 ≤ *C_ij_* ≤ 1, where *C_ij_* = 1 corresponds to perfect correlations, *C_ij_* = –1 corresponds to perfect anticorrelations, and *C_ij_* = 0 corresponds to uncorrelated gene pairs.

We calculated Pearson correlation between all genes for each chromosome for the five phenotypes. The code for calculation of Pearson correlations can be found in (38).

### 2.3 Spectral Decomposition

Pearson correlation matrices for each chromosome were calculated in order to analyze their spectral properties. Previous works on correlation matrices have shown that their spectral properties carry information about the structure and dynamics of the system (39–48).

For example, in stock market data, the first eigenvectors correspond to clusters of related industries (49, 50). In Electroencephalography measurements, these eigenvectors correspond to different functional regions in the brain (51). However, not all of the eigenvalues carry relevant information about the system. It has been shown that the smallest ones are the most sensitive to noise and some of them correspond to weak interrelations between small components from different clusters (47, 48). To distinguish how many eigenvalues contain useful information to identify clusters, we compared the spectral properties of the empirical correlation matrix to a null model represented by an ensemble of random matrices.

This ensemble of random matrices, is obtained by doing non-biased shuffling over the gene expression values for each sample (in this way, the original distribution of the data is preserved while its correlations will be destroyed) and computing the correlation matrix of each randomized data as in equation 1, we generated an ensemble of *n_m_* = 100 random matrices for each chromosome and phenotype.

The *k* deviating eigenvalues of the empirical matrix from the randomized data *max*(λ*_R_*) < {λ_1_, … ,λ*_k_*} are the ones containing correlations that cannot be attributed to either the noise in the system or data randomization. It is worth noticing that instead of using the eigenvectors from the spectral decomposition, which can be difficult to separate into independent clusters (52) (see **Supplementary Figure S2**), we used the number of *k* deviating eigenvalues as the number of independent clusters for a different clustering method.

### 2.4 Clustering Analysis

We implemented a clustering analysis based on the *k-medoids* algorithm. In a similar fashion to *k-means*, *k-medoids* clustering attempts to minimize the *distance* between the elements inside a cluster but one element is designated as the center of the cluster. The *k-medoids* algorithm works not exclusively with Euclidean distances, but with general pairwise interactions, this means we can use the correlation values we have estimated for each intra-chromosome matrix. Since correlation values are signed and their magnitude goes from –1 to 1, we define the pairwise interactions between genes *i* and *j* as:


(2) $$D_{i,j}\equiv 1-|C_{i,j}|,$$

with 0 ≤ *D* ≤ 1, high correlation or anti-correlation values mean close distance between points, while small correlation values will give higher distances. Finally, for the parameter *k* in the clustering algorithm, we considered the number of deviating eigenvalues as obtained from the spectral decomposition.

Given the stochastic nature of the *k-medoids* algorithm, we did *n_r_* = 100 realizations for each clustering computation to ensure statistical significance (*p* < 0.01), choosing the output configuration as the one with the minimum mean distance between the centroids and the elements in each cluster.

In order to compare the clustering results between the control phenotype and any other cancer subtype in a given chromosome, we constructed the intra-cluster Nearest Neighbor Distance (NND) distribution for each subtype. The NND of a given gene *i* in a cluster *k* is defined as:


(3) $$D_{nn}^{i}\equiv \min(|j-i|)\forall j\in C_{k},$$

where *C_k_* refers to the cluster *k*. To quantify the difference between the clustering in adjacent normal and cancer subtypes we compute Shannon’s entropy $H(x)=-\Sigma_{x\in \chi }p(x)\log(p(x))$ for the NND distributions, which in this case can be interpreted as how localized or how spread are the genes within each cluster in the chromosome. We also computed the Kolmogorov-Smirnov distance between the adjacent normal case and each of the Cancer subtypes. Given two cumulative distribution functions (CDF) the Kolmogorov-Smirnov distance is defined as:


(4) $$D_{KS}(F_{n},F_{m})=\underset{x}{\sup}|F_{n}(x)-F_{m}(x)|,$$

where the functions *F_n_* and *F_m_* are the CDFs for two samples *n* and *m*.

## 3 Results

**Figure 1:**
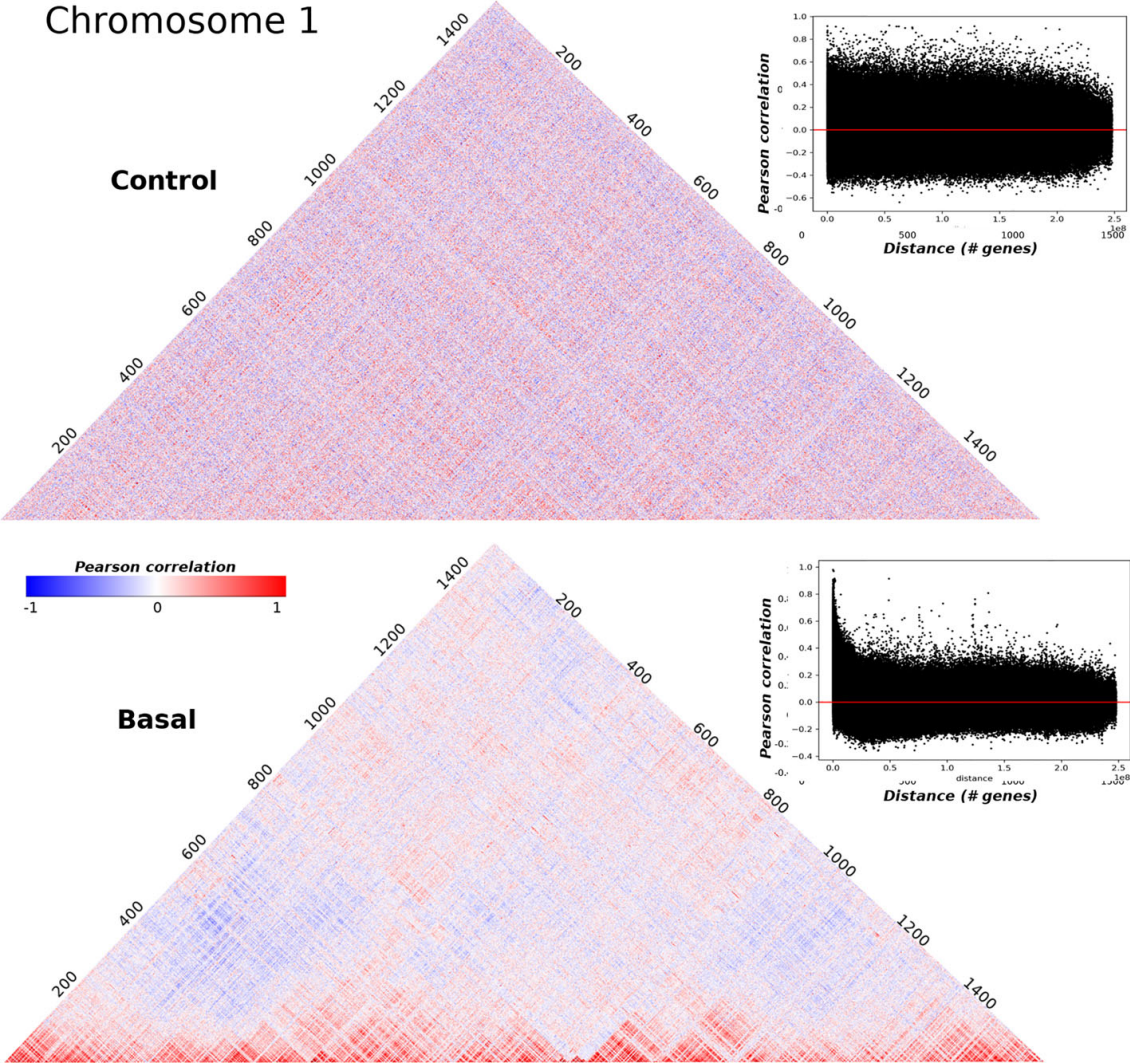
Pearson correlation square matrices for chromosome 1 in control samples (up) and basal breast cancer subtype (down). Genes are placed according to their physical location on the chromosome. Colors represent the correlation value: red corresponds to positive values, meanwhile negative correlations are depicted in blue. The inserts in the right part of both matrices correspond to the scatterplot of Pearson correlations versus distance. The horizontal red line corresponds to Pearson correlation = 0.

A correlation matrix of the sort just described, can be visualized as a heatmap as shown in **Figure 1** where correlation matrices for adjacent normal and basal subtype samples in the chromosome 1 are displayed. The axis represent the genes ordered by their physical location in the chromosome. The clearest difference between both matrices seems to be the lowest value of absolute correlation for genes that are physically distant in the basal subtype case. The heatmaps for each chromosome in the five phenotypes can be observed in **Supplementary Materials S2**–**S6**.

The effect of loss in long range co-expression is consistent with previous works of regulatory networks in breast cancer (10, 12–14, 33, 53). The block-type structure of the basal subtype matrix suggests the utility of clustering analysis to compare the structural properties of the correlation matrices. In what follows, we will present results for these clustering analyses. Through the manuscript, the presented figures will show different chromosomes for the five phenotypes. This has been done, in order to illustrate the universal nature of the gene clustering in breast cancer molecular subtypes, compared with the adjacent normal tissue.

### 3.1 Co-Expression Decays in All Chromosomes in All Subtypes

**Figure 2:**
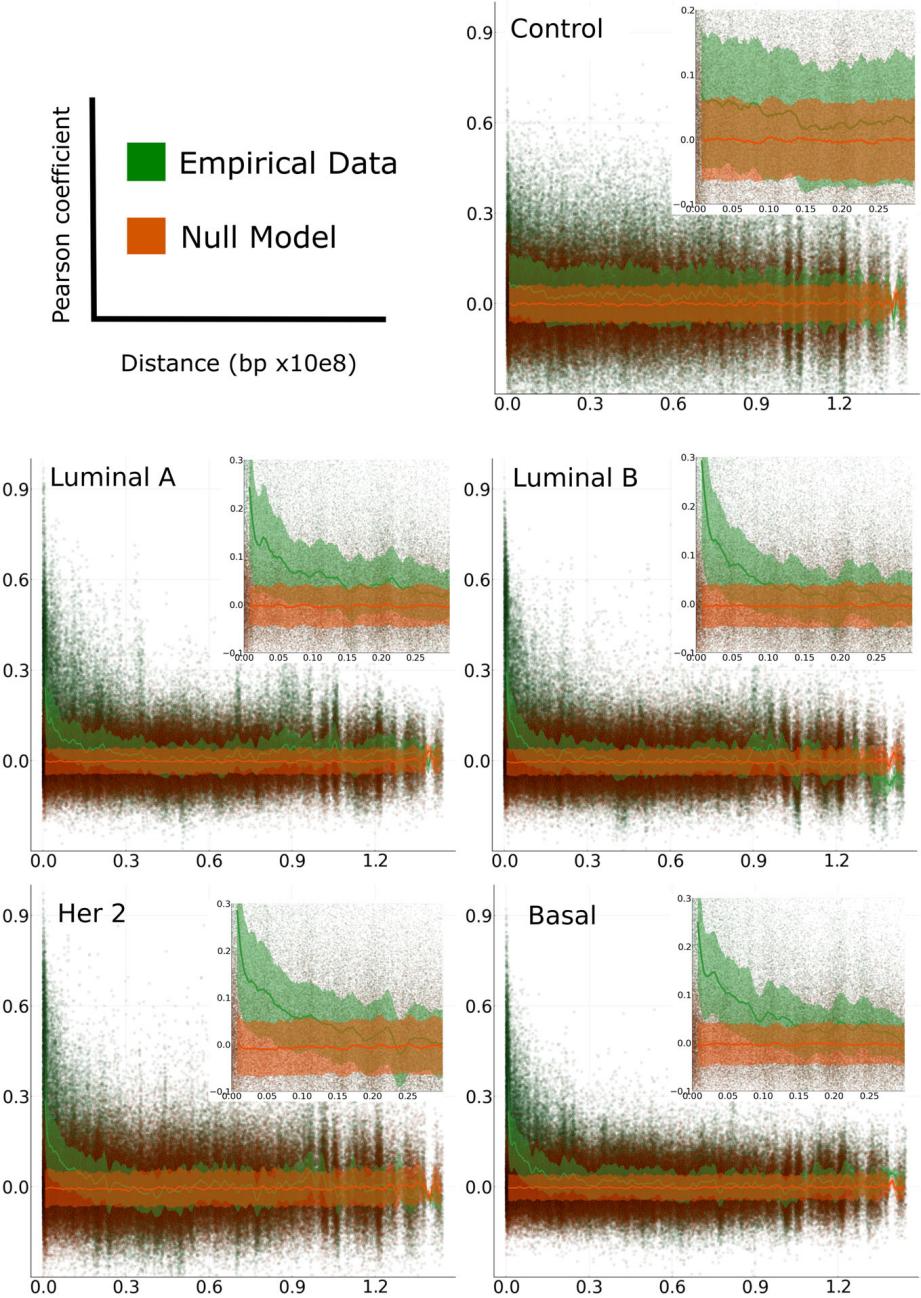
Pearson correlation of gene-gene expression *versus* distance. Plots for adjacent normal and cancer subtypes of chromosome 8 (green) and their respective null model (orange). The solid lines represent the median of a moving average in the distribution of correlation values over each window and the shaded area is the range from its first and third quartiles.

We observed a common pattern of distance dependency in all chromosomes in all breast cancer phenotypes. The decay in gene co-expression corresponds exclusively to positive correlations. In the case of negative correlations, such effect is not observed. Conversely, in adjacent normal chromosomes, there is no dependency of distance neither in negative nor positive interactions. Interestingly, this effect is observed in all chromosomes in the four breast cancer molecular subtypes and not observed in adjacent normal breast tissue-derived correlations (**Figure 2** and **Supplementary Materials S7–S11**).

**Figure 3:**
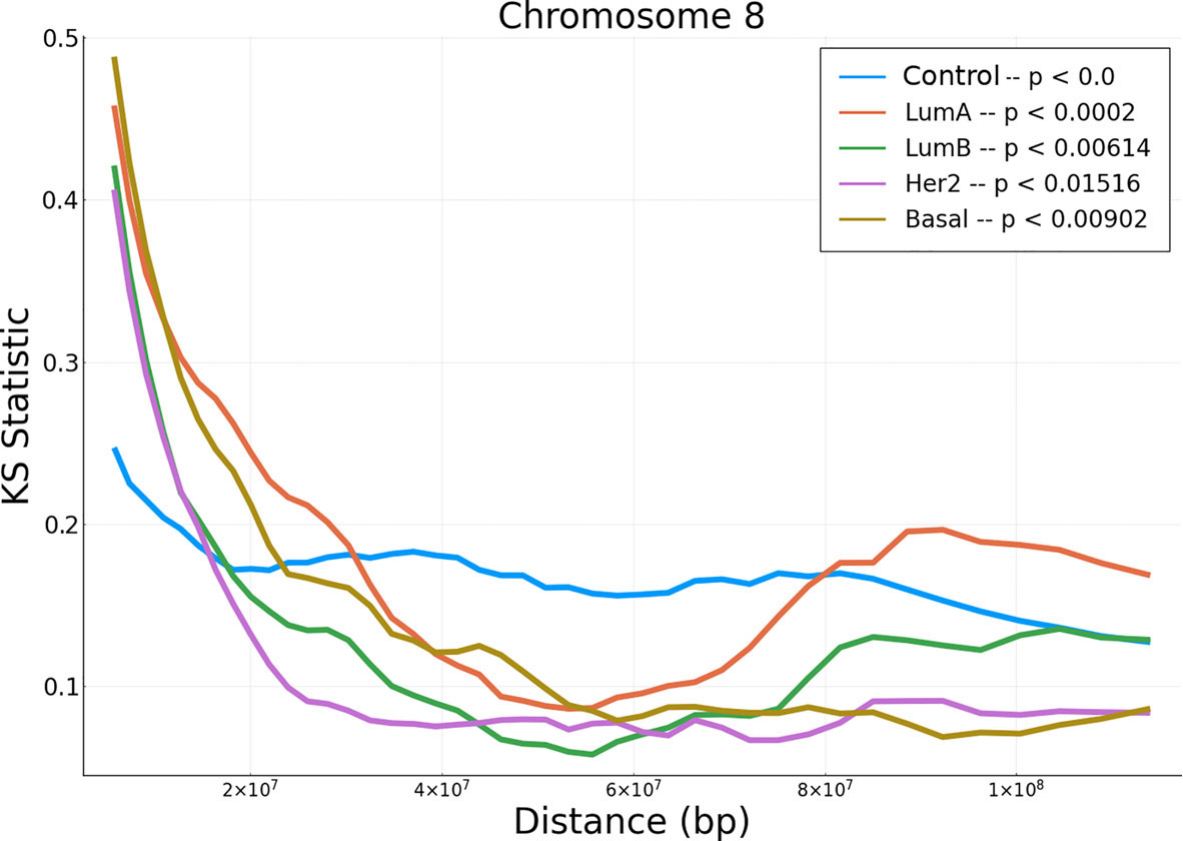
KS hypothesis test between empirical data from chromosome 8 and null model for the five phenotypes. This plot represents the KS statistic *versus* distance for all phenotypes in chromosome 8. The *p*– value for the control phenotype is smaller than 10^–5^.

In order to evaluate the differences between the empirical data and the null model, we performed a non-parametric hypothesis test (Kolmogorov-Smirnov) for the correlation values distributions (in all tumor subtypes and adjacent normal tissue) versus phenotype-specific null models. Additionally we implemented their corresponding significance tests (obtained *via* bootstrap/permutation analysis). The results of the KS test can be observed in **Figure 3**. The results for the rest of chromosomes, as well as their significance p-values, are presented in **Supplementary Materials S12**, **S13**).

Notice that at short distances, the cancer phenotypes have larger values than the adjacent normal correlations. However, at larger distances, KS for adjacent normal network are larger than those for cancerous phenotypes. The p-values shown in the upper right part of the figure, represent the average of all set of distances.

Based on a null model that lacks the linear correlations from the original data (see Methods), we observed that in adjacent normal chromosomes, positive and negative correlation values seem to be independent of the distance between genes, having significantly higher absolute values when compared with the null model at any distance. In the case of cancer subtypes, negative correlations are non-significant, but a few small regions in specific chromosomes (See **Supplementary Figure S3**).

### 3.2 Eigenvalue Decomposition Defines the Number of Clusters in All Phenotypes

**Figure 4:**
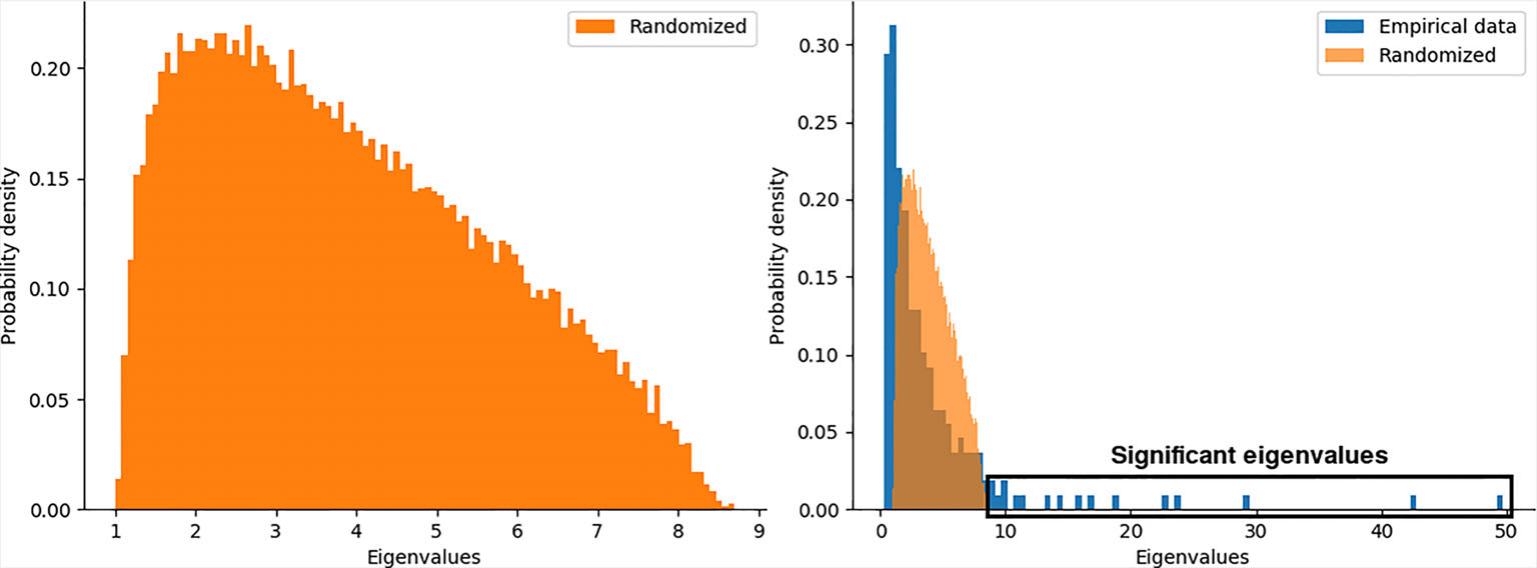
Probability distributions of eigenvalues for a) the ensemble of random matrices, b) random and empirical data for the chromosome 17 in the Basal sub-type.

We generated an ensemble of (*N* = 100) random matrices and compare the eigenvalue distributions from both, original and random matrices (see *Materials and Methods*). The left panel of **Figure 4** shows the eigenvalue distribution for the ensemble of random matrices, where its shape is the well-known Marchenko-Pastur distribution from random matrix theory (54). Overlapped eigenvalue distributions for the original matrix of chromosome 17 and the ensemble of surrogates are shown in the right panel of **Figure 4**. A set of significant eigenvalues was determined by random matrix permutations (*p* < 0.01) (see box in **Figure 4**).

### 3.3 Gene Clustering Is Distance Dependent in Breast Cancer

**Figure 5:**
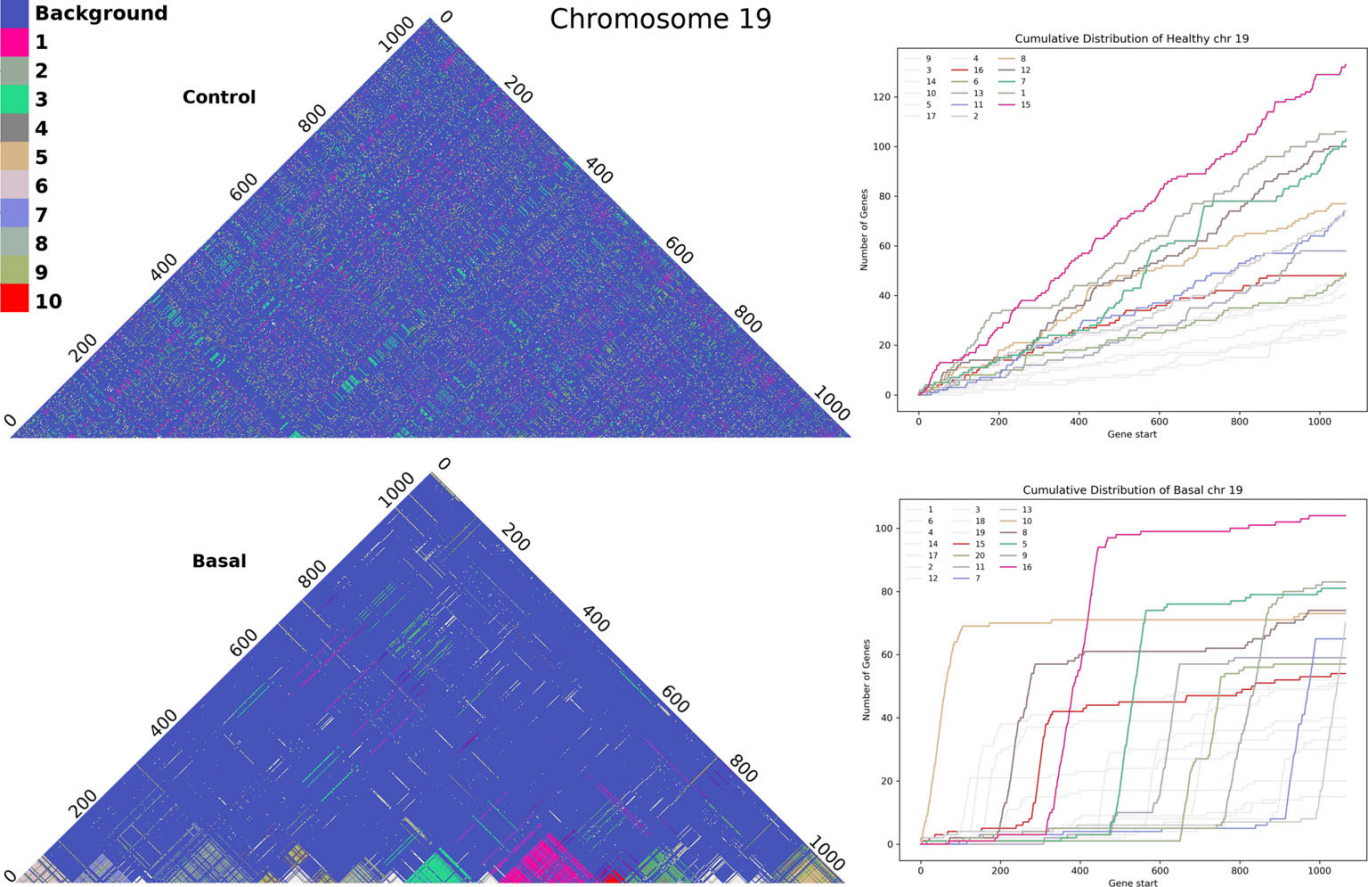
Cluster assembly of Chromosome 19 for adjacent normal-tissue and Basal breast cancer matrices. Upper part: Heatmap for all clusters in the adjacent normal matrix. Lower part: analog heatmap for Basal breast cancer network. The right panels correspond to the cumulative distribution for each cluster in chromosome 19. Colors represent the top-10 largest clusters.

With the method referred in Section 3.2 we obtained the full set of clusters for each chromosome in all phenotypes. **Figure 5** shows Chromosome 19 clusters with genes sorted by gene start base pair position. In the adjacent normal chr19 figure (upper part) we cannot discern a pattern in cluster colors. The distribution of clusters does not seem to depend on the distance between genes. Meanwhile, in basal breast cancer, we can observe cluster panels of colors, clearly detached. In the same figure, in the right panels, we plot the cumulative distribution of genes for each cluster. The larger the slope, the more often contiguous genes belong to the same cluster. All clusters for the five phenotypes in all chromosomes can be found in **Supplementary Material S14**. Cumulative distribution for all clusters can be found in **Supplementary Material S15**.

**Figure 6:**
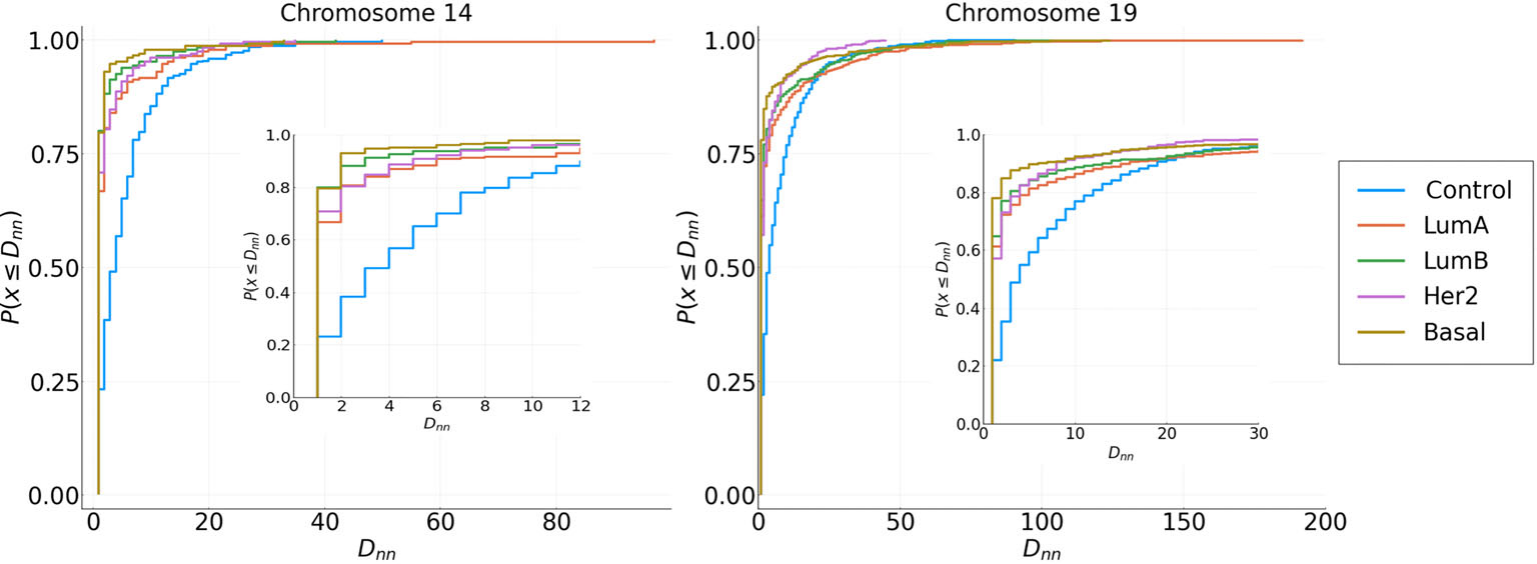
Cumulative NND distributions for each subtype in Chromosomes 14 and 19. Distributions of Cancer subtypes have different behavior in short and long distances compared with the adjacent normal tissue.

Cumulative distributions for the Nearest Neighbor Distance (NND) of two different chromosomes are shown in **Figure 6**, which can be interpreted as the probability distribution of the minimum distance between two genes in the same cluster. The behavior seen in the previous **Figure 5** holds: genes from the same cluster are more likely to be close to each other.

**Figure 7:**
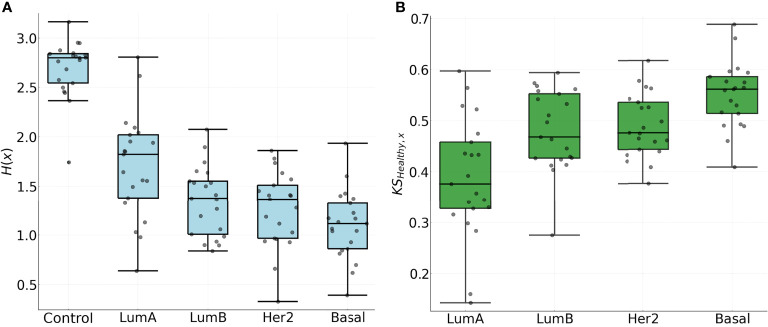
**(A)** Entropy for the NND distribution in all chromosomes. **(B)** Kolmogorov-Smirnov distance between the CDF of the NND in the adjacent normal phenotype and each of the Cancer subtypes for each chromosome.

Results for the entropies for the NND distributions are shown on the left panel of **Figure 7**, where a clear trend with the value *H*(*x*) can be identified: Luminal A, Luminal B, HER2+, Basal. It is worth noticing that the aforementioned order coincides with survival rates and metastatic behavior (14, 55, 56). The subtypes with the lowest survival rates and more metastatic behavior also present lower entropy values.

The latter is in agreement with a previously observed trend for the top 0.1% gene co-expression interactions for the four phenotypes: The most aggressive phenotype (basal) has the lowest number of inter-chromosome interactions, meanwhile the Luminal A subtype, which is considered the one with the best prognosis, contains a much larger fraction of interactions between genes from different chromosomes (14).

The decay in the entropy for the NND distribution presents further evidence that in the cancer subtypes, genes co-express in tighter patterns, in contrast with genes in the control phenotype that co-express at broadly scattered distances over the chromosome. A similar trend holds for the *KS* distance between the control phenotype and each subtype in the right panel of **Figure 7**, where higher values indicate a larger difference in the spatial organization of the clusters. The difference in spatial organization within the clusters in the chromosome is evident with both measures and it is correlated with the survival rate and metastasis of the subtype (14).

## 4 Discussion

Cancer research increasingly requires comprehensive computational analysis tools. In the search for relevant biological information, it is essential to be able to find selective patterns of individual or collective gene expression. In this sense, clustering methods are becoming a pivotal computational tool.

In this work, we studied the co-expression of genes in breast cancer molecular subtypes. We implemented a method to find the optimal clustering between genes that are co-expressed. We observed a grouping pattern in the case of cancer phenotypes with respect to the adjacent normal group. These patterns in the genome indicate that in cancer, physically close genes are co-expressed (*cis-* interactions), while for distant genes (*trans-* interactions) the clustered co-expression is, to a large extent, lost.

The piece-wise Kolmogorov-Smirnov tests for all tumor subtypes and adjacent normal tissue versus phenotype-specific null models and their corresponding significance tests (obtained *via* bootstrap/Permutation analysis) (included in the **Supplementary Materials S12**, **S13**), show that correlation values at short distances are much more significant for all chromosomes in any cancer phenotype than the adjacent normal network.

It is worth noticing that the significance of the KS tests also decays with the distance for all chromosomes in any cancer phenotype. Conversely, for the adjacent normal network, distance does not exert a considerable influence in the significance of the KS test. Finally, the KS test also show that the significance of differences between the correlation of our empirical data with the null model is unique for each chromosome and for each phenotype.

The fact that genes are highly co-expressed in groups with close positions, may be due to a favored number of nearby transcription sites or to the strong presence of transcription factors. It has been observed for instance, that in Luminal A breast cancer gene co-expression networks, co-factors, CTCF binding sites (57, 58) or copy number alterations (59, 60), may remodel chromatin making it more or less accessible, thus allowing gene transcription of local neighborhoods, resulting in the concomitant high co-expression between those neighboring genes (53). On the other hand, TFs influence more often inter-chromosome edges, meanwhile intra-chromosome interactions are less affected by them (53).

Physical interactions such as CTCF binding sites have captured attention in recent years (61, 62), and more importantly, in breast cancer (63).

For instance, in (53), we constructed an intra-chromosome gene-co-expression network for Luminal A breast cancer samples. There, a community detection method was performed to determine whether CTCF binding sites appeared in the borders of those communities. We observed that there is no link between CTCF binding sites and the border of intra-chromosome communities. In that sense, we argued that, at least for Luminal A breast cancer gene co-expression networks, CTCF binding sites are not determinant for network structure.

Transcription factor (TF) regulation is, of course, one of the central mechanisms for gene regulation. With respect to the role that TFs may exert on gene clustering, we have previously shown that TFs influence genes in a trans- fashion, i.e. TFs from a given chromosome regulate genes from different chromosomes. We have shown that in terms of Master Regulators in breast cancer (64, 65), but also in Luminal A breast cancer networks (53). Conversely, for intra-chromosome genes, TFs influence is much less evident.

Finally, Copy Number Variations (CNVs) have been considered as a crucial factor in the rise and development of breast cancer (59). In fact, a correlation between CNVs, protein levels and mRNA gene expression has also been reported previously (66). Hence, high correlations between clusters of physically closed genes appear to be related to copy number alterations.

We have used TCGA-derived CNV data and compared the amplification/deletion peaks with LumA network communities. Interestingly, the community with more overexpressed genes, composed of genes such as FOXM1, HJURP, or CENPA, presented large regions of deletions. The apparently contradictory result suggested that the copy number alterations do not influence the structure of that community. On the other hand, a gene community formed by HLA family genes, presented a common pattern of amplification, but those genes were not differentially expressed (53).

With the aforementioned in mind, we argue that CNVs are not as relevant as one could expect in terms of the gene clustering shown here. Moreover, CNVs influence may be at the expression level, but said effect is more limited regarding co-expression. However, further investigation is necessary to clarify these ideas.

The structure of clustered genes in physically close neighborhoods resembles the images obtained by Hi-C methods (67–69).

In recent times, there has been an increased interest as to how chromosome conformation capture experiments such as Hi-C may lead to relevant clues towards our understanding of further effects in connection with transcriptional regulation. Indeed, we are currently conducting research along these lines in our group. Work is ongoing, however, we can advance that there seems to be important correlations between loss/gain of statistically significant chromosomal contacts and co-expression relationships between genes in the associated genomic regions. It remains to be determined however whether said correlations are significant *via* proper assessment of null models and, more importantly, to determine what may be the biological consequences of these associations.

Preliminary findings from our Hi-C analysis in breast cancer indicate that more relevant contacts are mostly (but not exclusively) on close genomic regions. This is not unlike what we have observed with MI-based gene co-expression networks in which there is a preponderance of co-expression interactions in shorter distances for tumors. Future work undoubtedly will focus on the comparison between the network clusters constructed by this method and those from Hi-C. In particular, the zones/genes between gene groups. The assessment and comparison of both structures will provide us more information regarding the structural alterations during the carcinogenic process.

In brief, after revising the evidence about other mechanisms of gene regulation, we may hypothesize that the ultimate cause of the distance-dependent gene clustering is not a single mechanism, but instead, it could be a non-linear combination of different phenomena. In particular, regarding gene clustering, we have evaluated for the first time the whole set of gene interactions, and the loss of long-distance co-expression remains, which is more evident in the most aggressive subtypes.

Homogeneity/redundancy promotes higher entropy. Systems with redundancy are less likely to fail to catastrophic events. In other words, it seems there are mechanisms that give robustness to gene regulation in a control phenotype. It is still uncertain whether the loss of long range (or gain in short range) gene co-expression is a consequence of cancer, forcing the system to work in a less entropic configuration, but it seems that this preference for a less entropic configuration is common in all cancer subtypes and is consistently progressive with subtype aggressiveness.

As a summary of findings, we may establish the following:

- We used tools previously implemented in time series analysis in the stock market and neuroscience settings (49–51) to develop a systematic, data-driven method for intra-chromosomal gene expression clustering. Using spectral decomposition and a null model, we were able to determine the number of co-expressed group of genes to perform *k*–medoids algorithm calculations and determine the most accurate clustering configuration. This method allowed us to have significant results, avoiding to set an *a priori* threshold for co-expression values.
- In the adjacent normal phenotype matrices, negative and positive correlations are significant throughout the entire chromosome. On the other hand, in breast cancer, negative correlations are observed in the same rank than those from a null model (see **Figure 2**); furthermore, the positive ones are only out of the null model cloud over short distances.
- In cancer, clustering mostly occurs between nearby genes, unlike what happens in the adjacent normal phenotype matrices. This is a representation of high co-expression over short distances. This fact coincides and corroborate previous results on mutual information-based co-expression networks in these and other types of cancer (10, 12–14).
- The intra-cluster Nearest Neighbor Distance (NND) clearly decays from the adjacent normal network to those cancerous ones. Additionally, the NND for breast cancer networks also decays according to the aggressiveness of the subtype: Luminal A, Luminal B, HER2+ and Basal.
- Analogously to the last point, Kolmogorov-Smirnov (KS) distance between the Cumulative Distribution Function of the NND in the adjacent normal and each breast cancer subtype network, increases with the aggressiveness of the subtype, thus indicating that the larger value of the KS distance, the larger difference between adjacent normal and breast cancer phenotypes’ networks.

Clustered genes may be subject to further analyses to reveal, for instance, statistical enrichment of functional categories revealing certain biological functions, additional patterns of coordinated activity, etc. This in turn may lead to the generation of hypotheses to be tested *via* more narrowly targeted assays and interventions.

A closer look at matrices’ patterns generated by other type of sorting methods may shed some light on possible mechanisms behind the regulatory changes in co-expression and perhaps even in the establishment of the tumor phenotypes. This is, indeed, still ongoing work.

Further steps towards the understanding of co-expression patterns and the differences in clustering among adjacent normal and cancerous phenotypes may be also based on the usage of multi-layer approaches (11, 70).

There are remaining questions prompted by this study. For example, while it is evident that there is a decay in the strength of correlations depending on the distance in all chromosomes, it is not fully clear what is the origin of the differences in the slope of the aforementioned decays. Also, the negative correlations in adjacent normal network are significant, independently of the position in the chromosome. Is the anti-correlation between genes a possible mechanism of negative feedback? Is that mechanism disrupted in breast cancer? Another important question regarding the clustering in cancer network is the size of the clusters. Is there an “optimal” cluster size for cancer networks? If so, what is the rationale behind such number?

Finally, the fact that other types of cancer, which have been analyzed in terms of gene co-expression interactions, such as clear cell renal carcinoma (13), lung adenocarcinoma, or lung squamous cell carcinoma (12) have been reported to have the same bias in short-distance interactions, a remaining question is whether the clustering behavior observed in breast cancer subtype networks is a conserved phenomenon along other cancer types.

The above mentioned questions, together with the acquired knowledge on cancer networks, will be eventually answered and that will bring us with complementary information to have a broader point of view on gene regulation in cancer.

## Data Availability Statement

The datasets presented in this study can be found in online repositories. The names of the repository/repositories and accession number(s) can be found below: https://github.com/josemaz/gene-matrices.

## Author Contributions

AG-E and JZ-F performed computational analyses, developed methods and implemented programming code, performed pre-processing and low-level data analysis, participated in the writing of the manuscript. EH-L contributed to the design of the study, co-supervised the project, contributed and supervised the writing of the manuscript. JE-E conceived and designed the project, performed calculations, supervised the project, drafted the manuscript. All authors contributed to the article and approved the submitted version. AG-E and JZ-F contributed equally as first author.

## Funding

This work was supported by the Consejo Nacional de Ciencia y Tecnología [SEP-CONACYT-2016-285544 and FRONTERAS-2017-2115], and the National Institute of Genomic Medicine, México. Additional support has been granted by the Laboratorio Nacional de Ciencias de la Complejidad, from the Universidad Nacional Autónoma de México.

## Conflict of Interest

The authors declare that the research was conducted in the absence of any commercial or financial relationships that could be construed as a potential conflict of interest.

## Publisher’s Note

All claims expressed in this article are solely those of the authors and do not necessarily represent those of their affiliated organizations, or those of the publisher, the editors and the reviewers. Any product that may be evaluated in this article, or claim that may be made by its manufacturer, is not guaranteed or endorsed by the publisher.

## Supplementary Material

Supplementary Figure 1 Principal component analyses of RNA-seq data clustered by breast cancer subtypes.

Supplementary Figure 2 Comparison of the squared components of the four largest eigenvectors from the correlation matrix b in **Figure 1**. It can be seen that there is an overlap between the eigenvectors that does not allow to separate the components into clusters.

Supplementary Figure 3 Outliers of positive correlations (red dots) in some chromosomes of the breast cancer subtype networks. Plots for chromosomes 1, 9, 19 and 17 for the four subtypes. Arrows indicate sets of positive correlations considered outliers. Notice that the outliers in each plot form almost vertical lines, indicating that those interactions present approximately the same distance. Additionally we can see a null model overlapping (blue).

Supplementary Material S1 Excel file containing cross tables between subtype-samples and histological variables.

Supplementary Material S2 Heatmaps of Pearson correlation for each chromosome in the adjacent normal phenotype. The color code is the same than in **Figure 1**.

Supplementary Material S3 Heatmaps of Pearson correlation for each chromosome in the Luminal A phenotype.

Supplementary Material S4 Heatmaps of Pearson correlation for each chromosome in the Luminal B phenotype.

Supplementary Material S5 Heatmaps of Pearson correlation for each chromosome in the HER2+ phenotype.

Supplementary Material S6 Heatmaps of Pearson correlation for each chromosome in the Basal phenotype.

Supplementary Material S7 to S11 Pearson distribution scatter plots for normal adjacent tissue, Basal HER2+, Luminal A and Luminal B, respectively. These plots show correlations sorted by gene start position for the four cancer phenotypes and the adjacent normal network per chromosome.

Supplementary Material S12 Kolmogorov-Smirnov significance tests for all chromosomes in the five phenotypes. As in **Figure 3**, the figures represent the KS statistics of 50 equal-area (same number of data-points) intervals for each chromosome. In the figures, the phenotypes are represented by different colors. The associated p-value for the complete set of correlations are depicted in the upper right part of the figures.

Supplementary Material S13 Piece-wise permutation p-values of the KS statistics, calculated for all bins obtained in **Supplementary Material S8**, in every chromosomal region for each phenotype.

Supplementary Material S14 Clusters by chromosome for the five phenotypes, obtained by eigenvalue decomposition and k-medoids method. The figures are depicted as in the manuscript. Additionally, this material contains files for clusters including the name of the gene, the cluster that the gene belong to, the assignment cost function value, the chromosome location of the gene, and the gene start position of said gene.

Supplementary Material S15 Cumulative distribution for the four cancer phenotypes and the adjacent normal network per chromosome. Color code coincides with clusters in **Supplementary Material S7**. For clarity, only the top-ten clusters were colored.
