# Supplementary figures and images for: Gene Co-Expression in Breast Cancer: A Matter of Distance (part 1 of 5)
Source: Front Oncol. 2021 Nov 17;11:726493. doi: 10.3389/fonc.2021.726493 (PMC8636045; doi:10.3389/fonc.2021.726493)

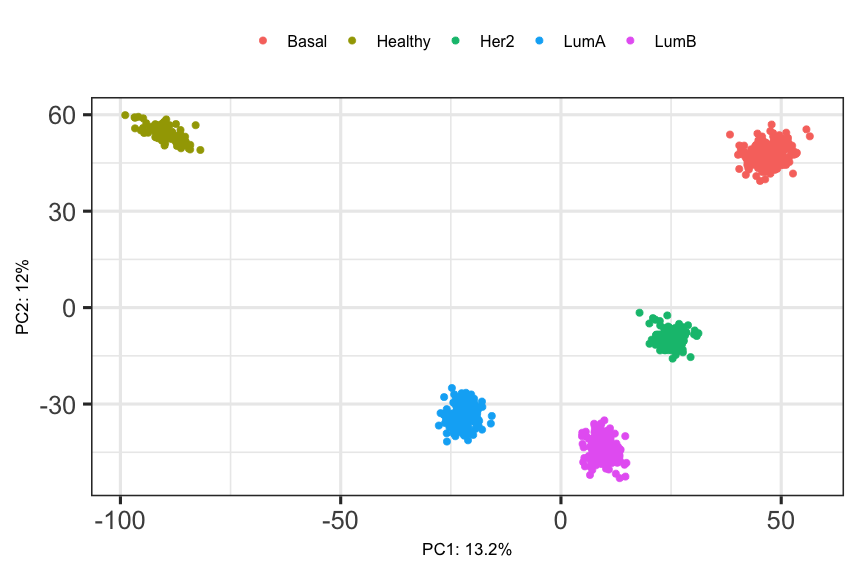

Supplement: Supplementary Figure 1 — Principal component analyses of RNA-seq data clustered by breast cancer subtypes. [file Image_1.png]

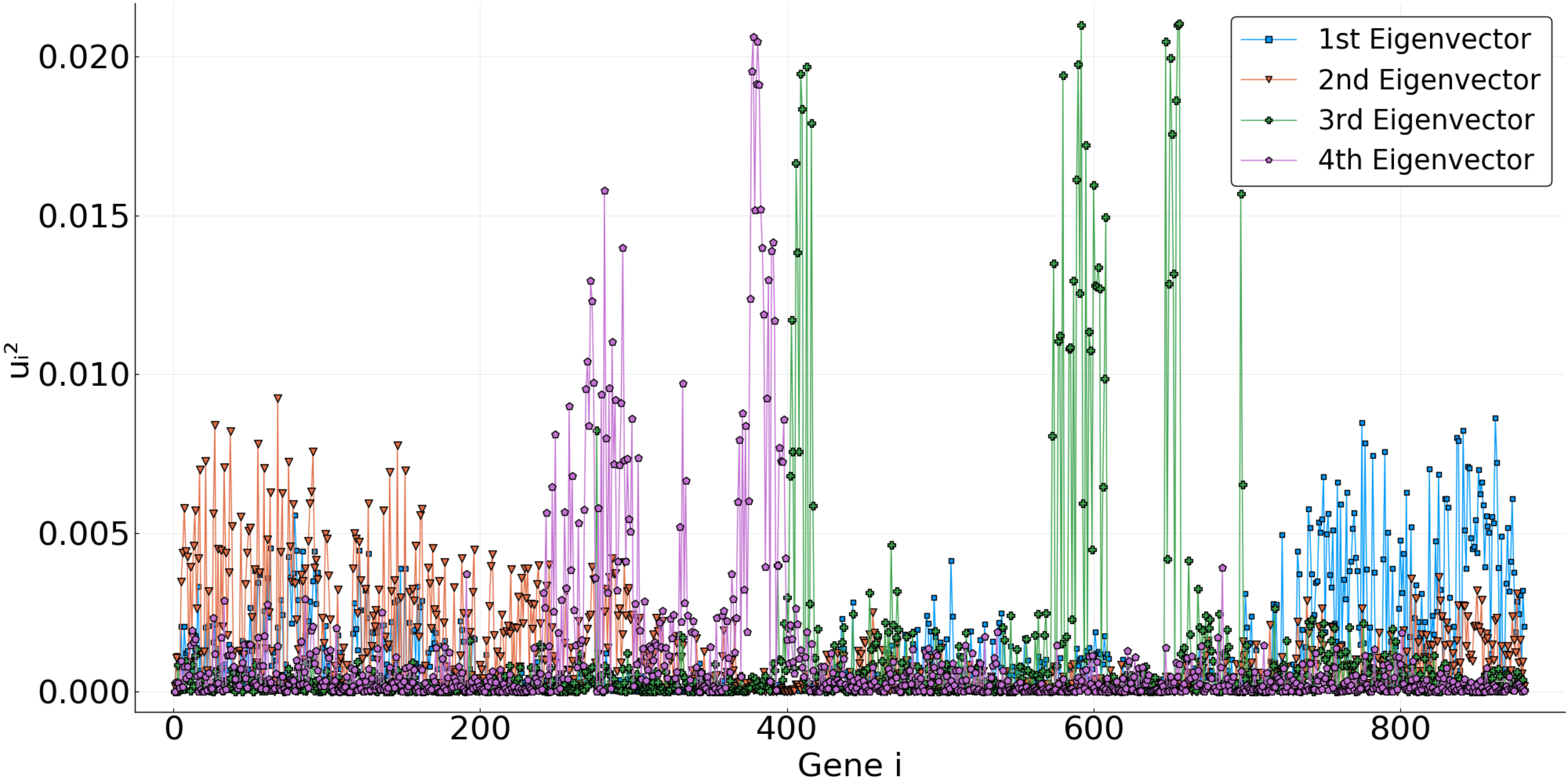

Supplement: Supplementary Figure 2 — Comparison of the squared components of the four largest eigenvectors from the correlation matrix b in Figure 1 . It can be seen that there is an overlap between the eigenvectors that does not allow to separate the components into clusters. [file Image_2.png]

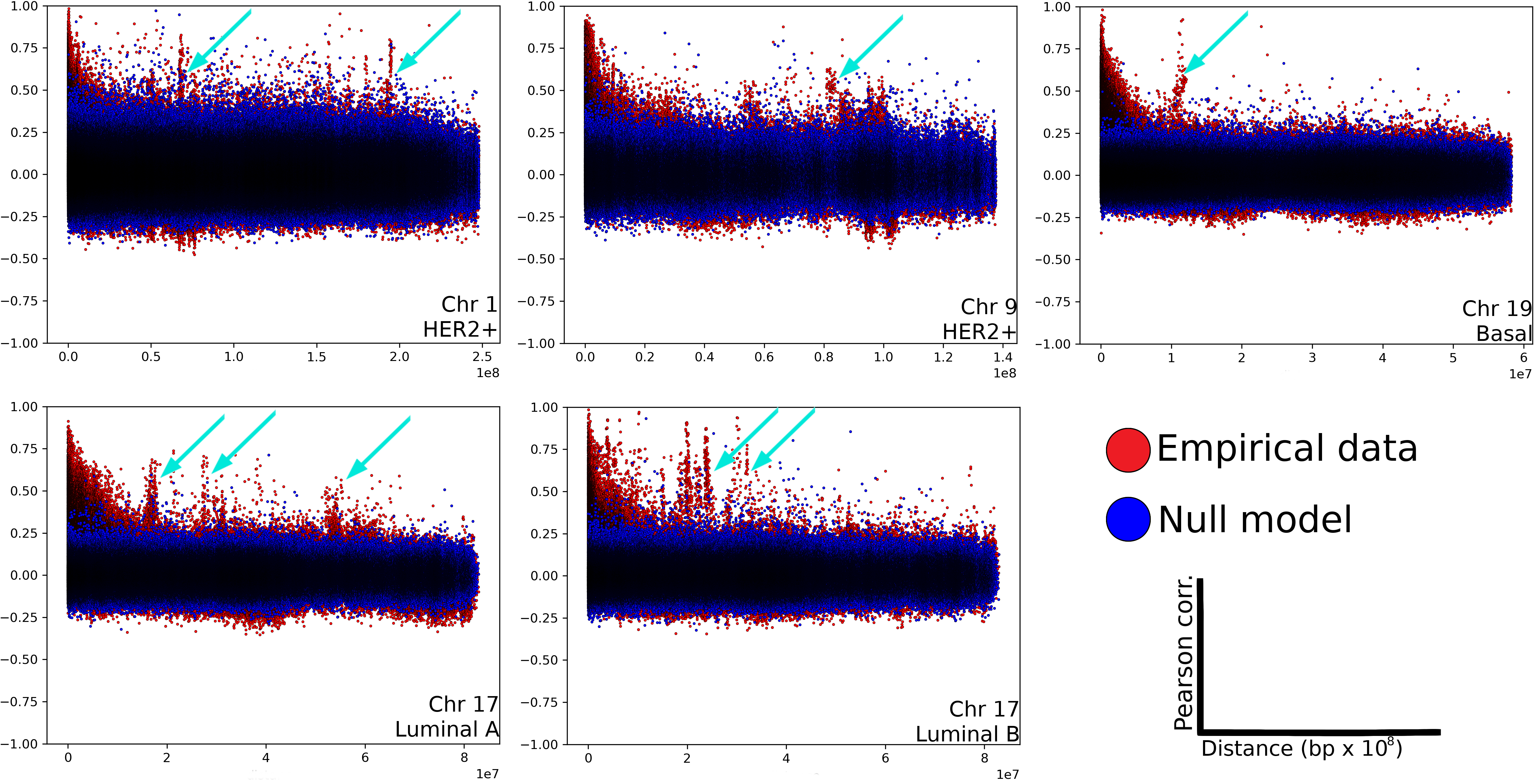

Supplement: Supplementary Figure 3 — Outliers of positive correlations (red dots) in some chromosomes of the breast cancer subtype networks. Plots for chromosomes 1, 9, 19 and 17 for the four subtypes. Arrows indicate sets of positive correlations considered outliers. Notice that the outliers in each plot form almost vertical lines, indicating that those interactions present approximately the same distance. Additionally we can see a null model overlapping (blue). [file Image_3.png]

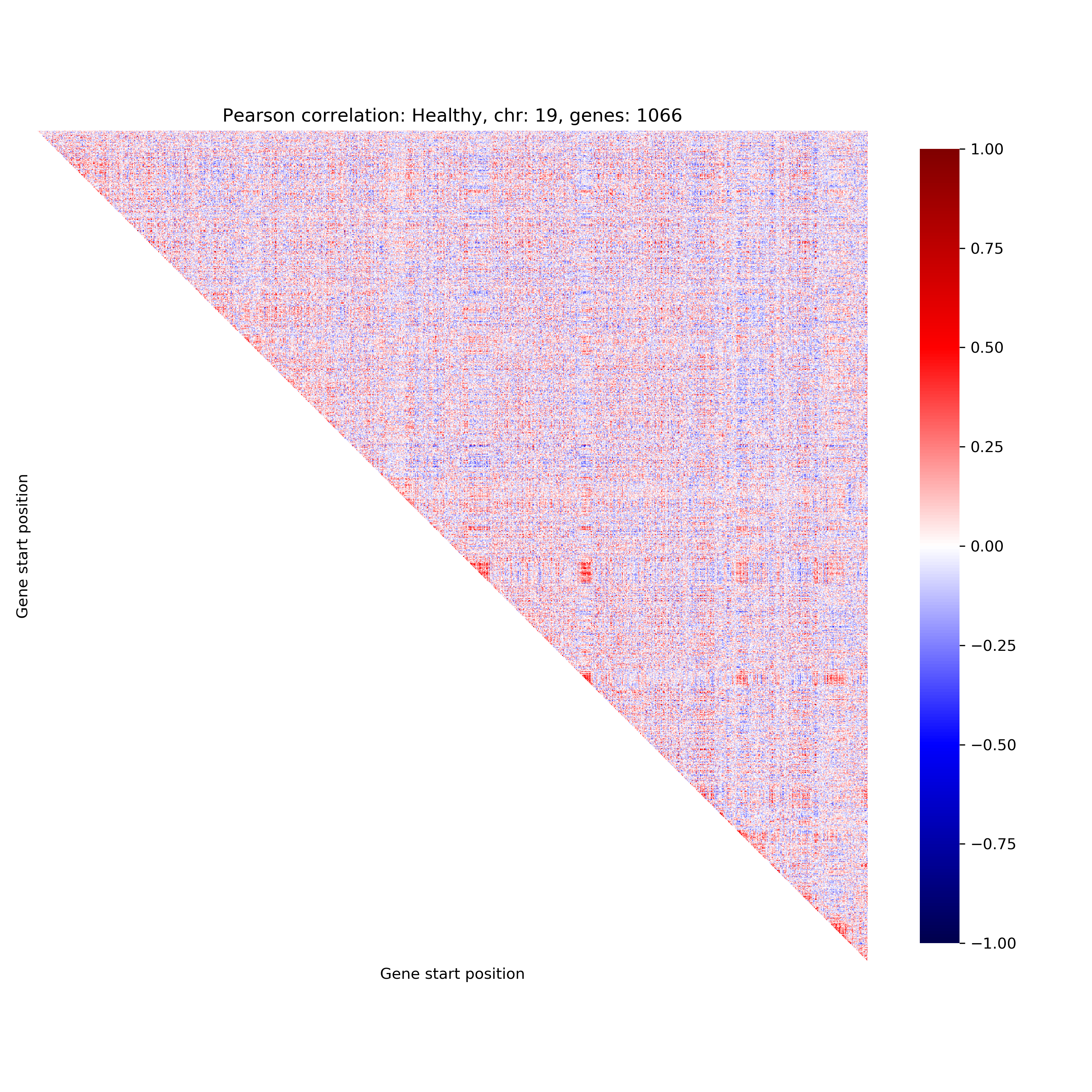

Supplement: Supplementary Material S1 — Excel file containing cross tables between subtype-samples and histological variables. [file DataSheet_1.zip › SuppMat2/Healthy-chr19.png]

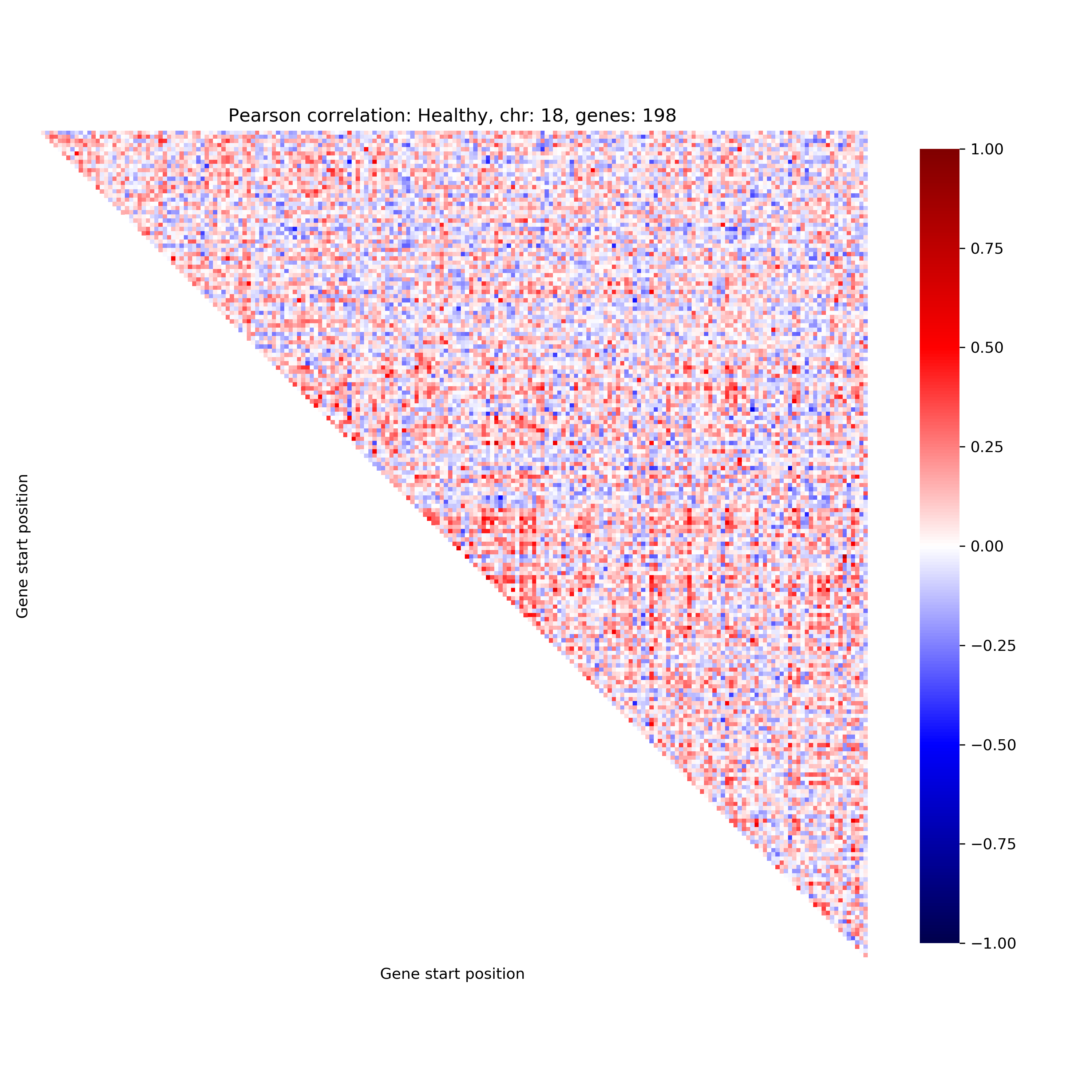

Supplement: Supplementary Material S1 — Excel file containing cross tables between subtype-samples and histological variables. [file DataSheet_1.zip › SuppMat2/Healthy-chr18.png]

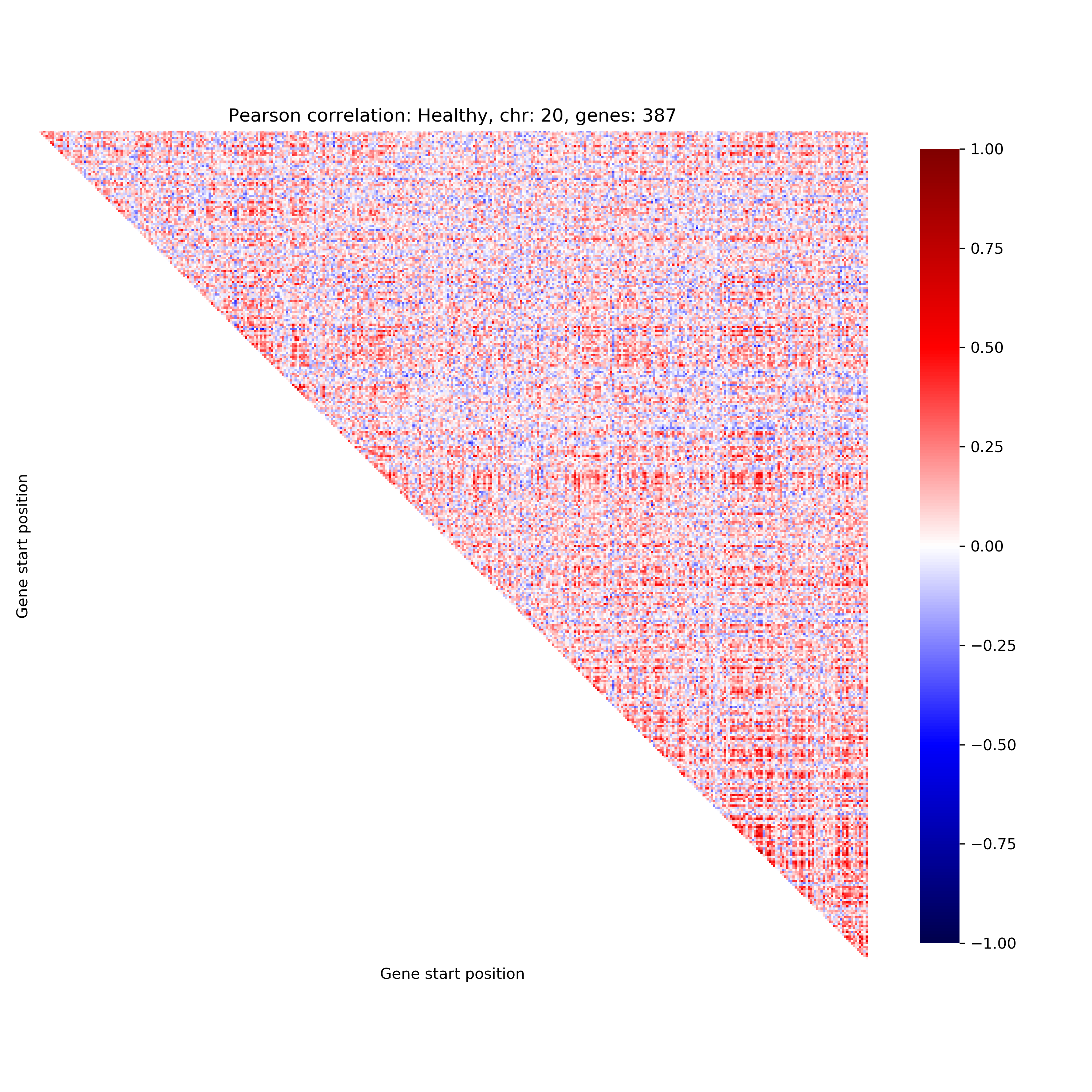

Supplement: Supplementary Material S1 — Excel file containing cross tables between subtype-samples and histological variables. [file DataSheet_1.zip › SuppMat2/Healthy-chr20.png]

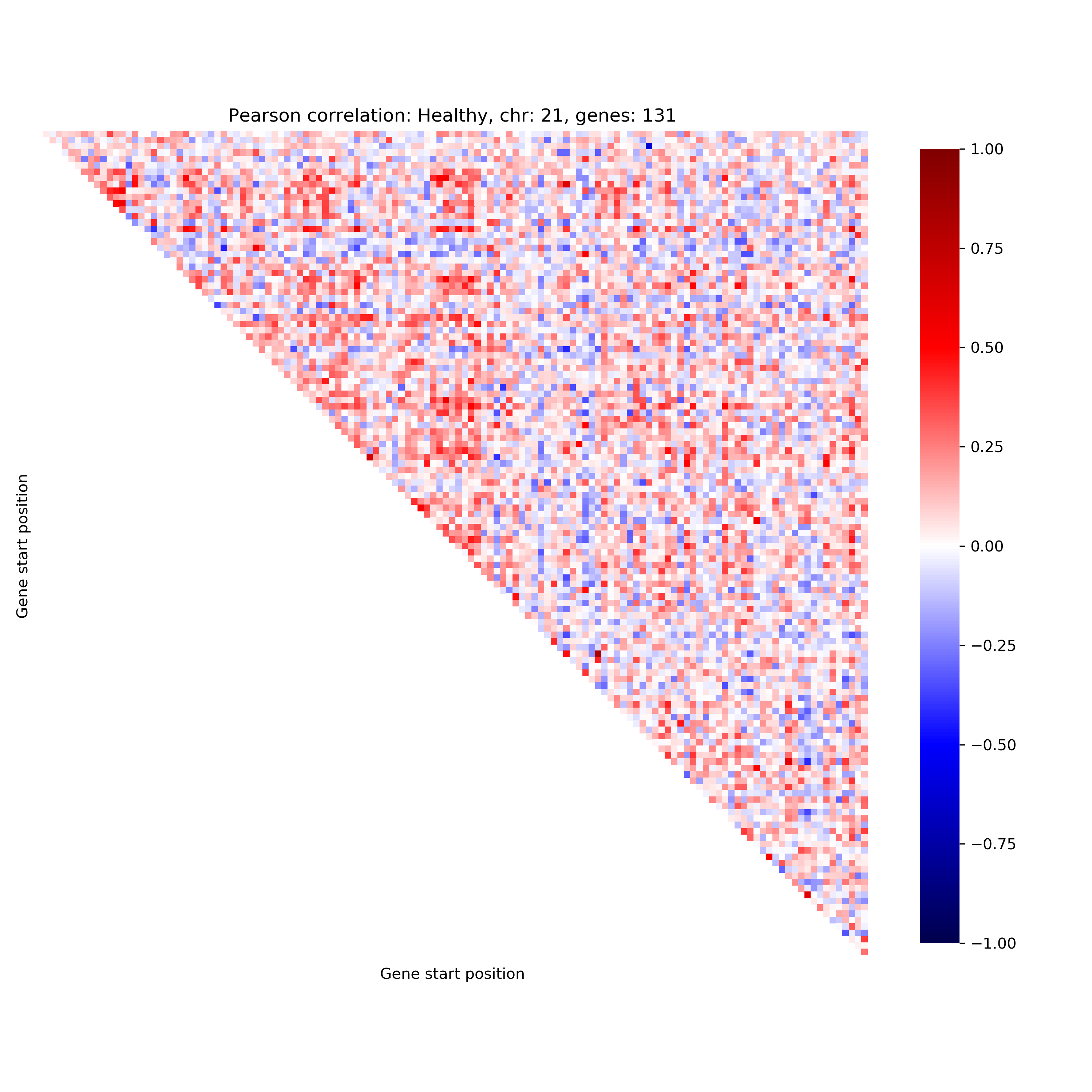

Supplement: Supplementary Material S1 — Excel file containing cross tables between subtype-samples and histological variables. [file DataSheet_1.zip › SuppMat2/Healthy-chr21.png]

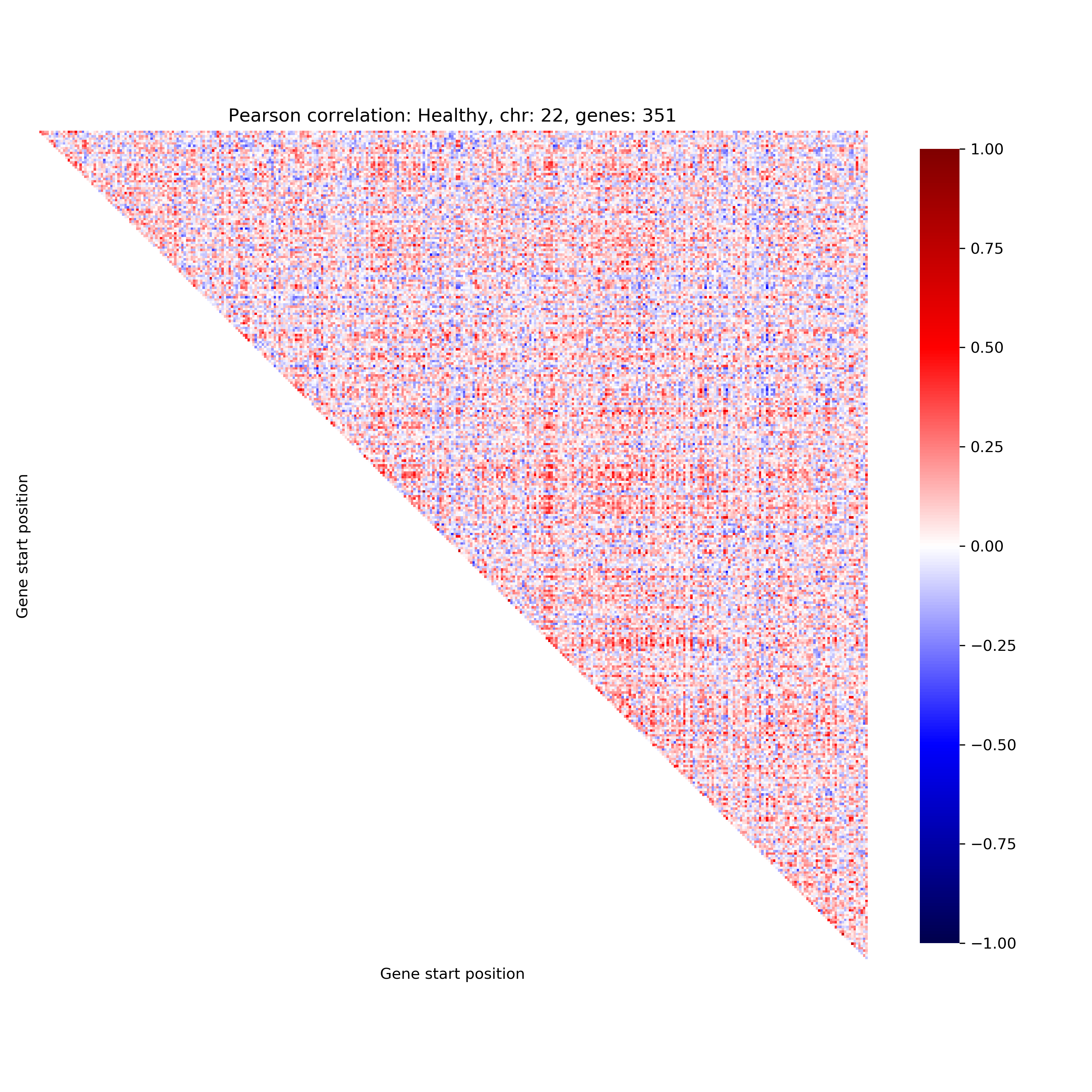

Supplement: Supplementary Material S1 — Excel file containing cross tables between subtype-samples and histological variables. [file DataSheet_1.zip › SuppMat2/Healthy-chr22.png]

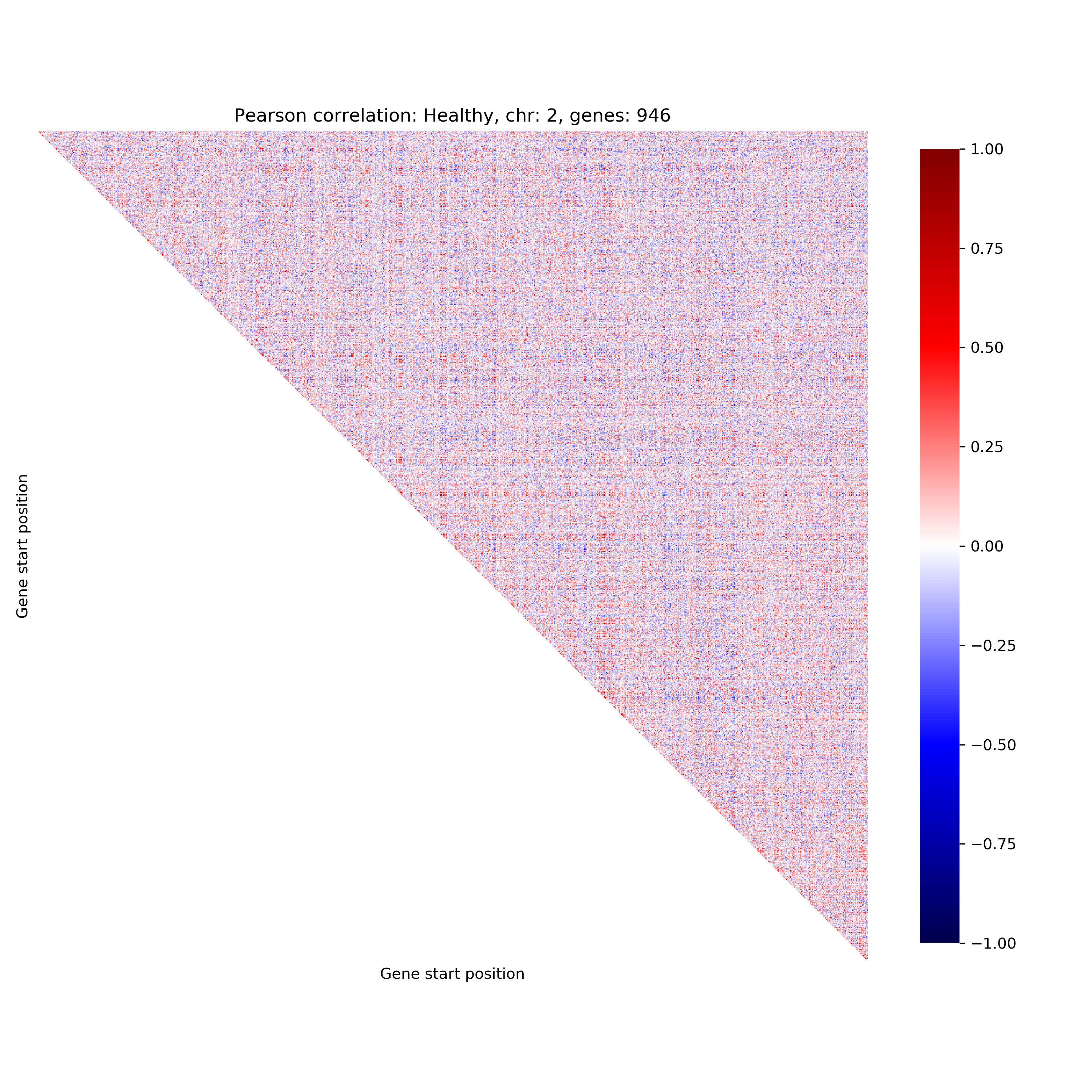

Supplement: Supplementary Material S1 — Excel file containing cross tables between subtype-samples and histological variables. [file DataSheet_1.zip › SuppMat2/Healthy-chr2.png]

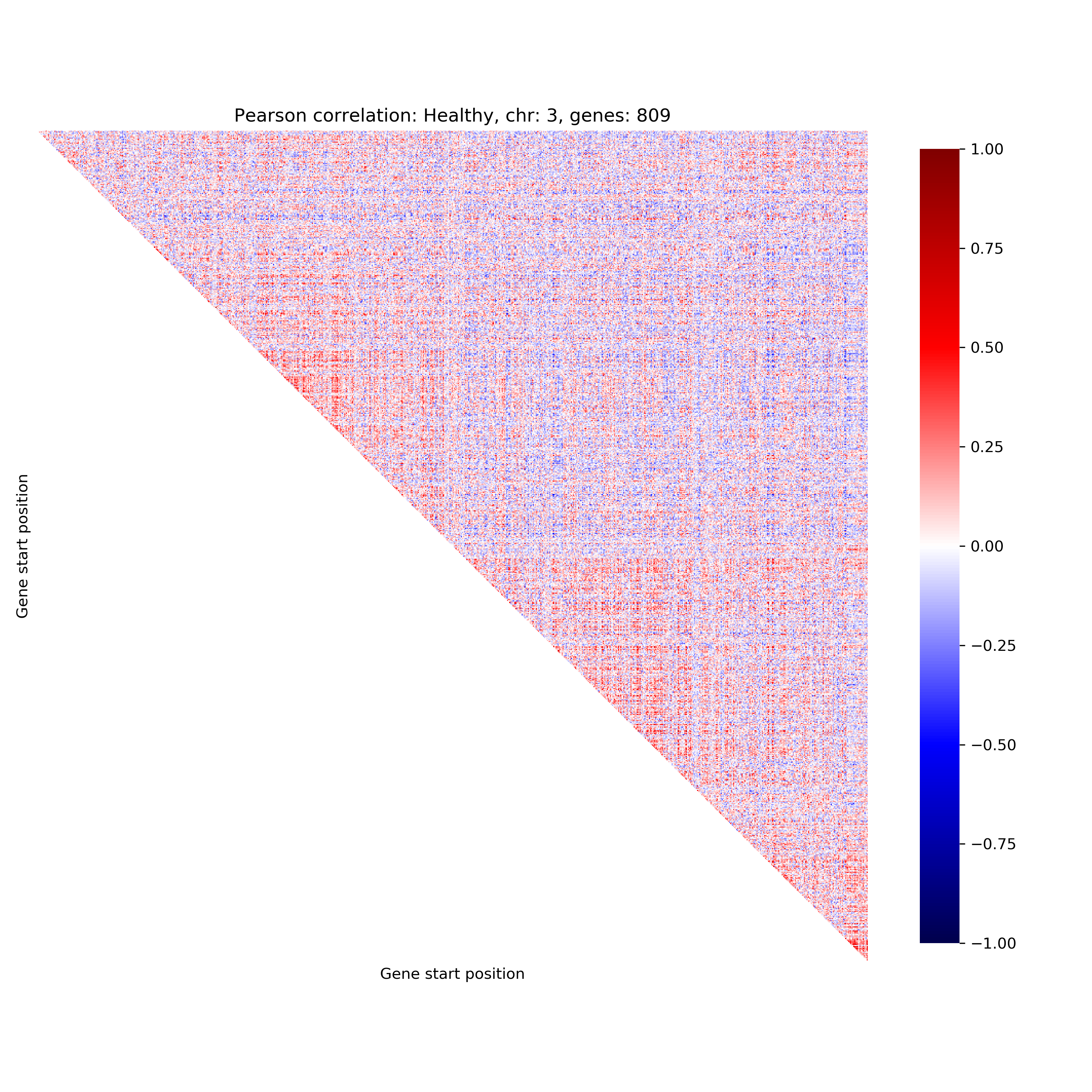

Supplement: Supplementary Material S1 — Excel file containing cross tables between subtype-samples and histological variables. [file DataSheet_1.zip › SuppMat2/Healthy-chr3.png]

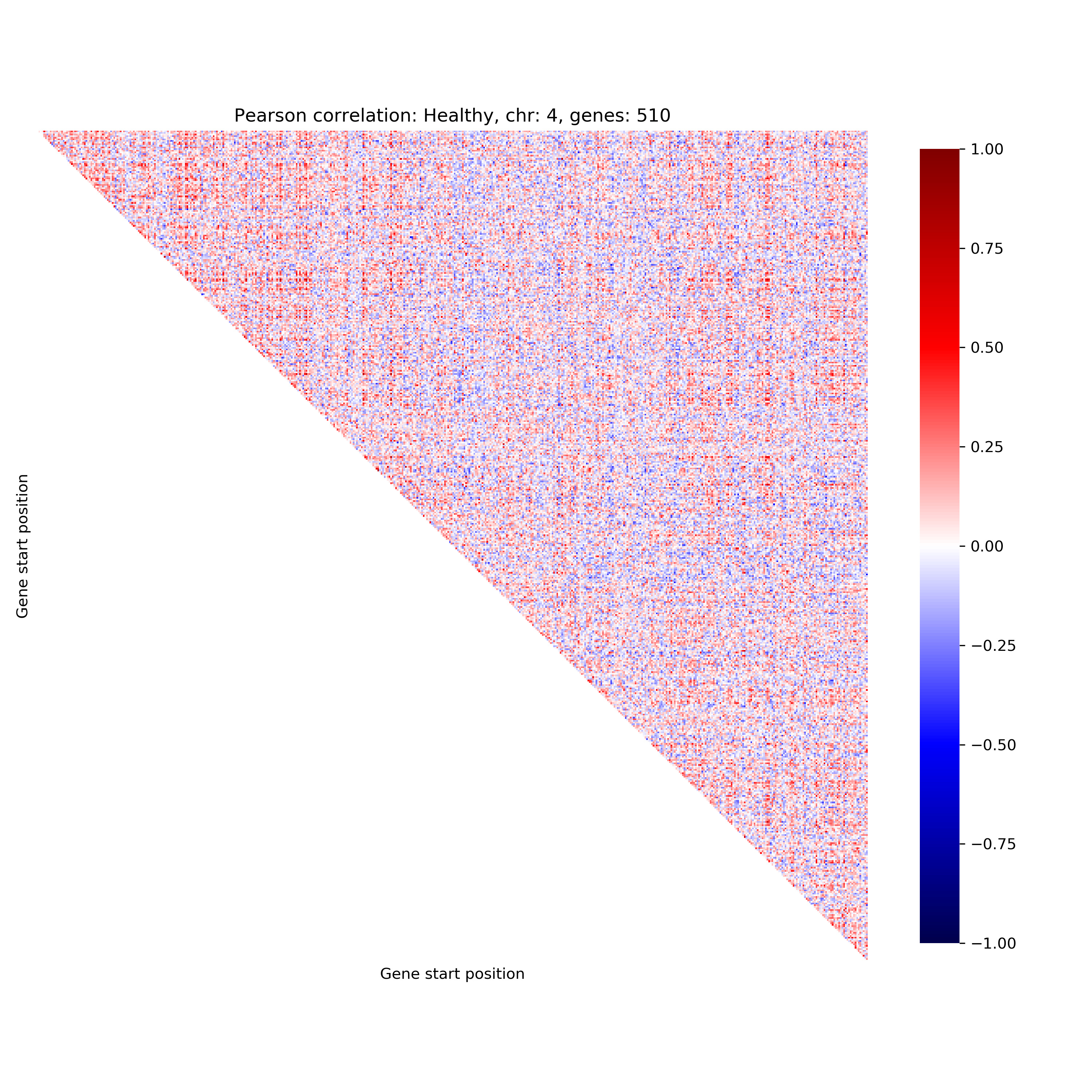

Supplement: Supplementary Material S1 — Excel file containing cross tables between subtype-samples and histological variables. [file DataSheet_1.zip › SuppMat2/Healthy-chr4.png]

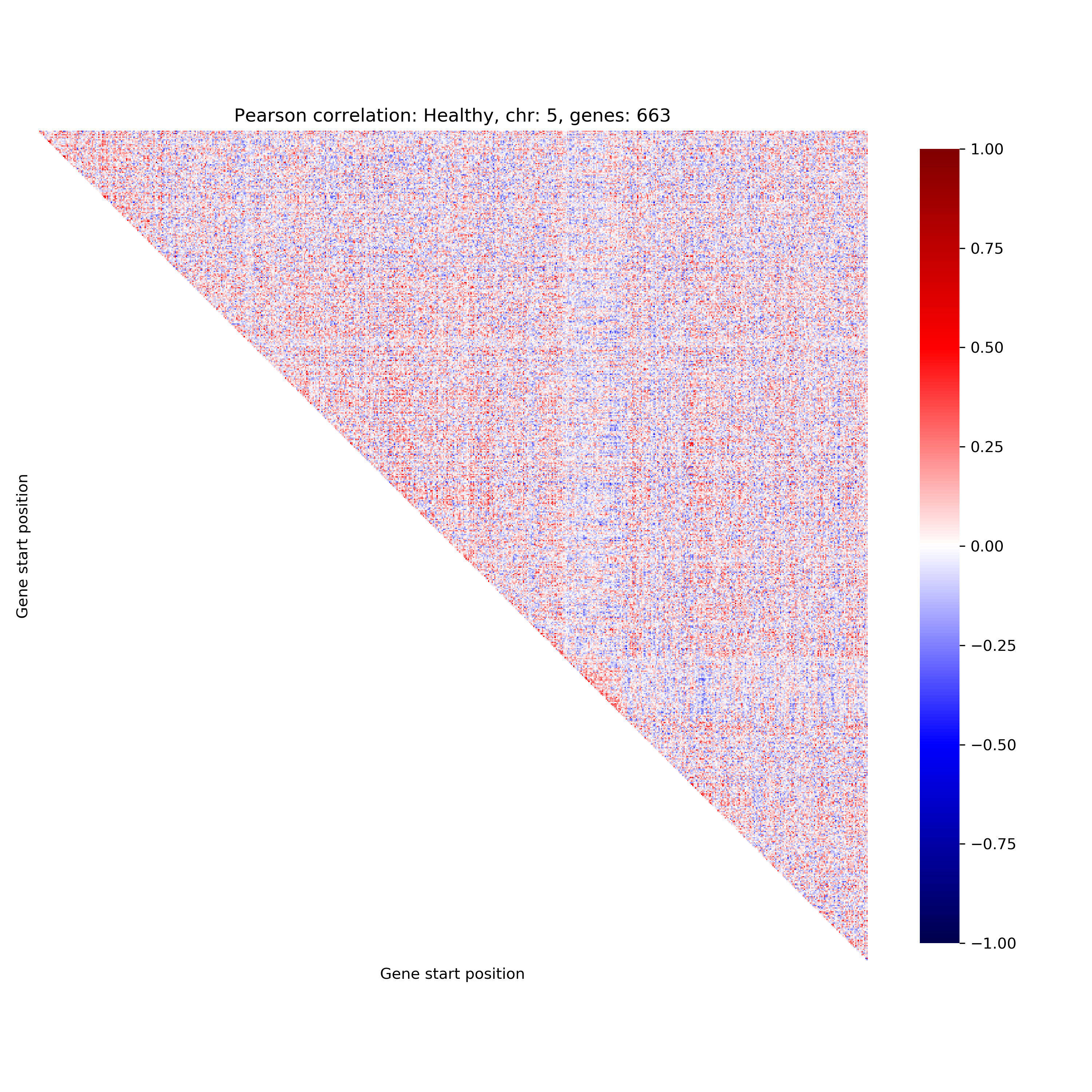

Supplement: Supplementary Material S1 — Excel file containing cross tables between subtype-samples and histological variables. [file DataSheet_1.zip › SuppMat2/Healthy-chr5.png]

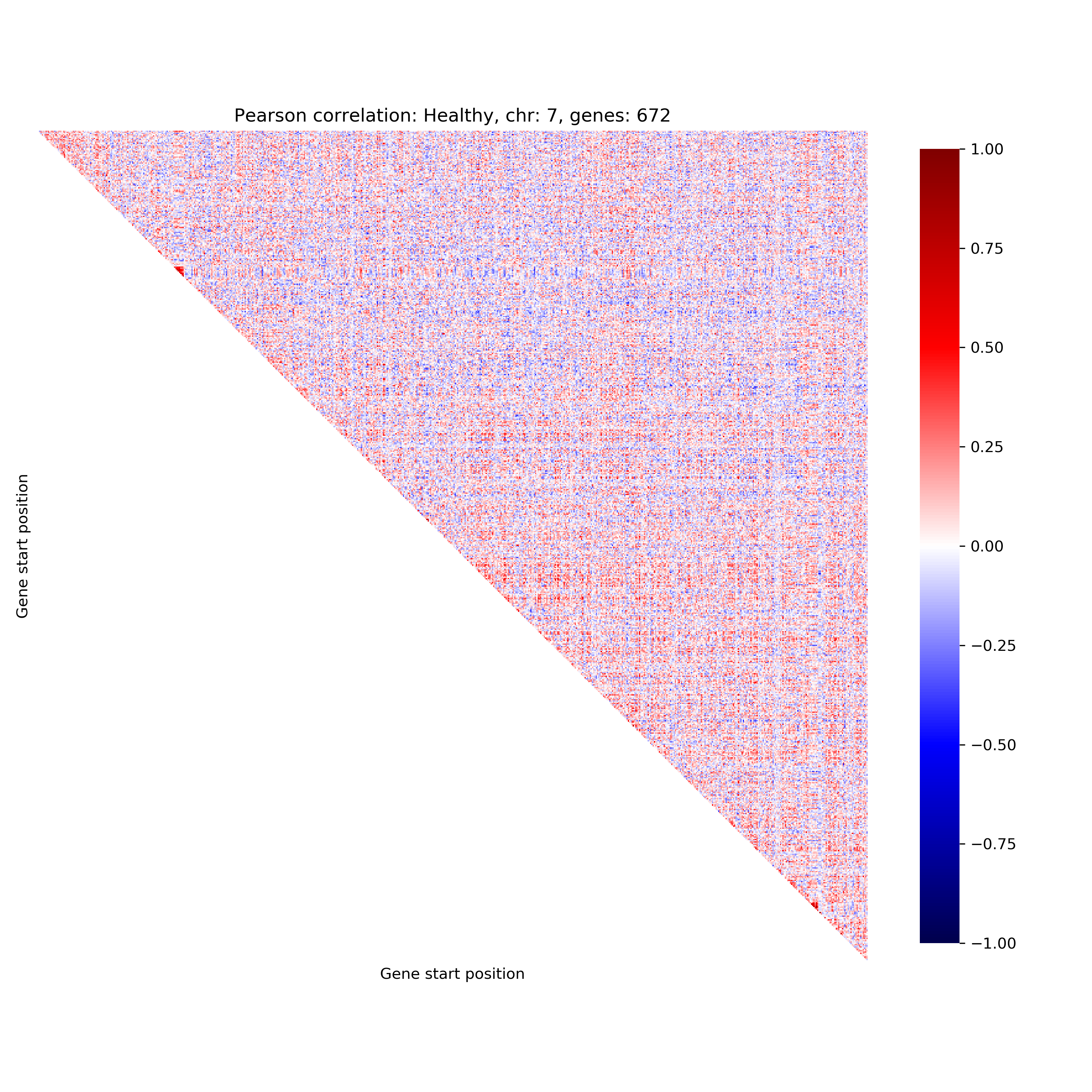

Supplement: Supplementary Material S1 — Excel file containing cross tables between subtype-samples and histological variables. [file DataSheet_1.zip › SuppMat2/Healthy-chr7.png]

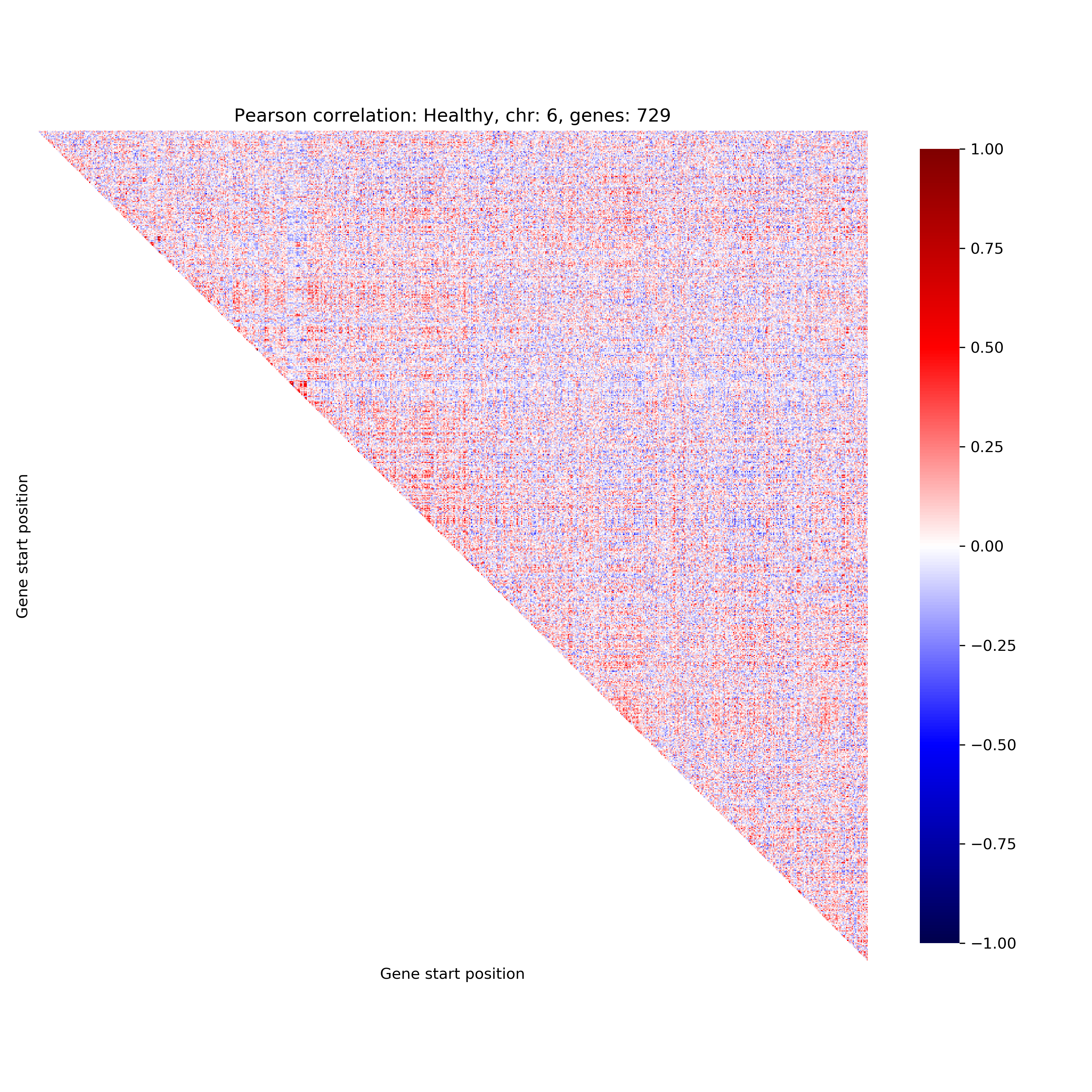

Supplement: Supplementary Material S1 — Excel file containing cross tables between subtype-samples and histological variables. [file DataSheet_1.zip › SuppMat2/Healthy-chr6.png]

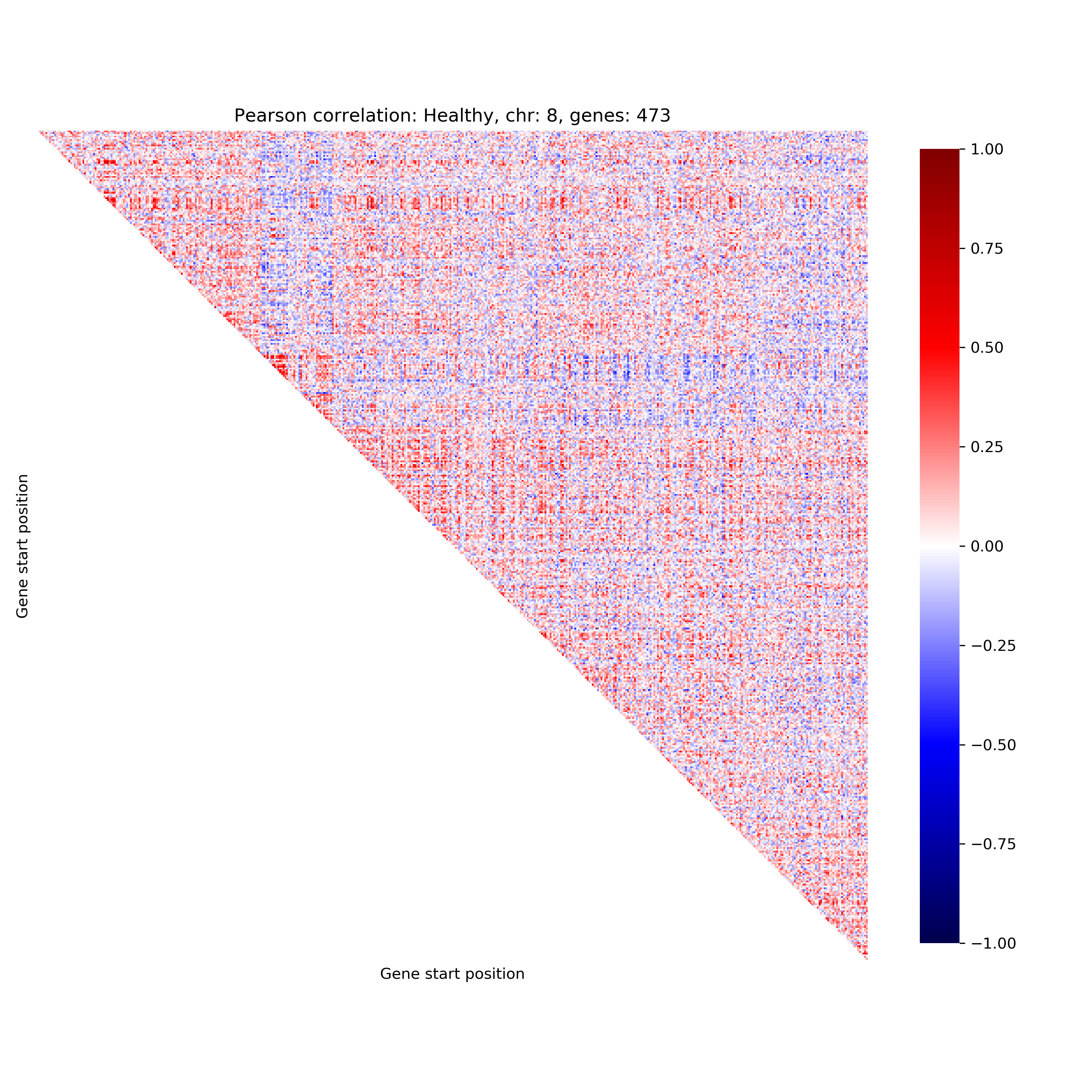

Supplement: Supplementary Material S1 — Excel file containing cross tables between subtype-samples and histological variables. [file DataSheet_1.zip › SuppMat2/Healthy-chr8.png]

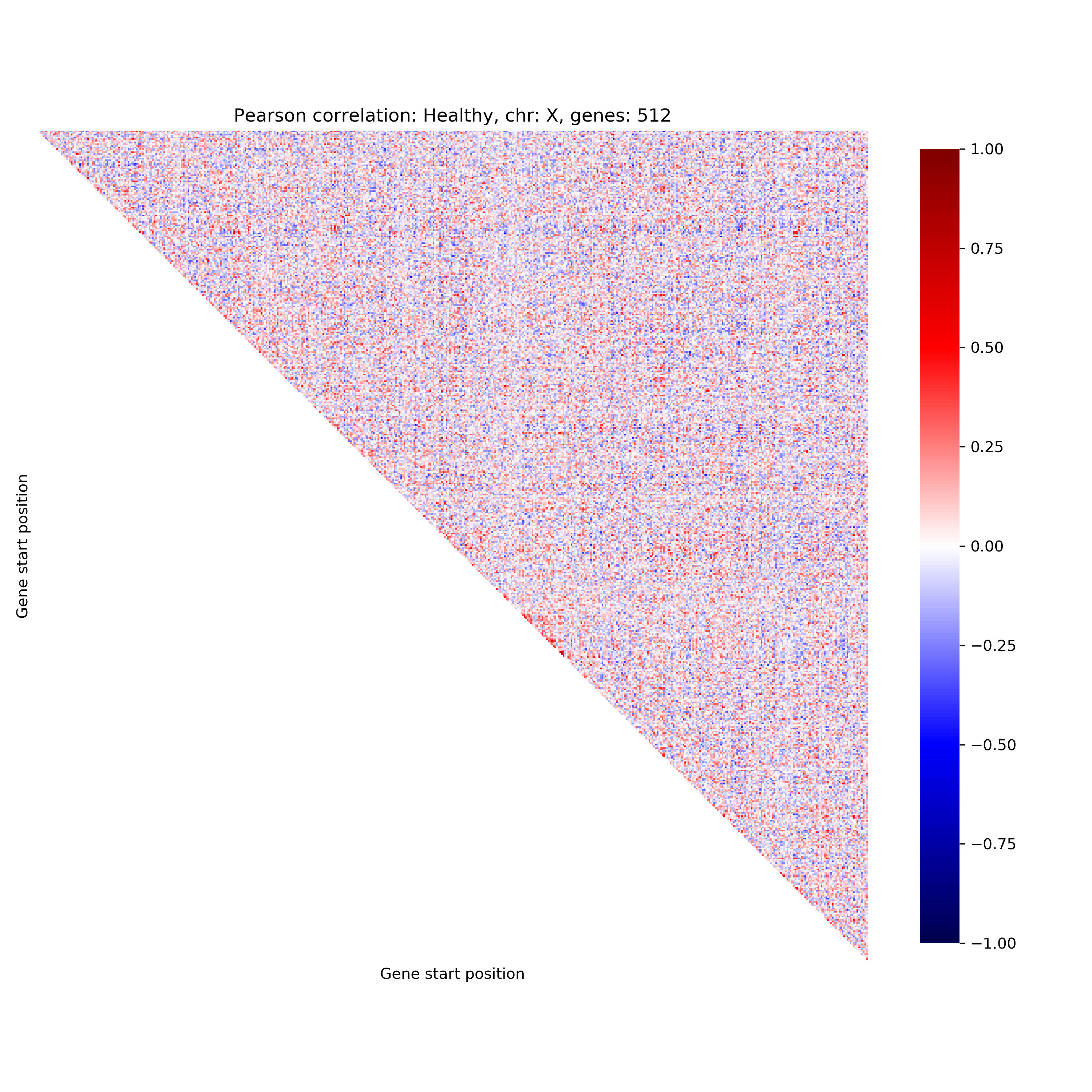

Supplement: Supplementary Material S1 — Excel file containing cross tables between subtype-samples and histological variables. [file DataSheet_1.zip › SuppMat2/Healthy-chrX.png]

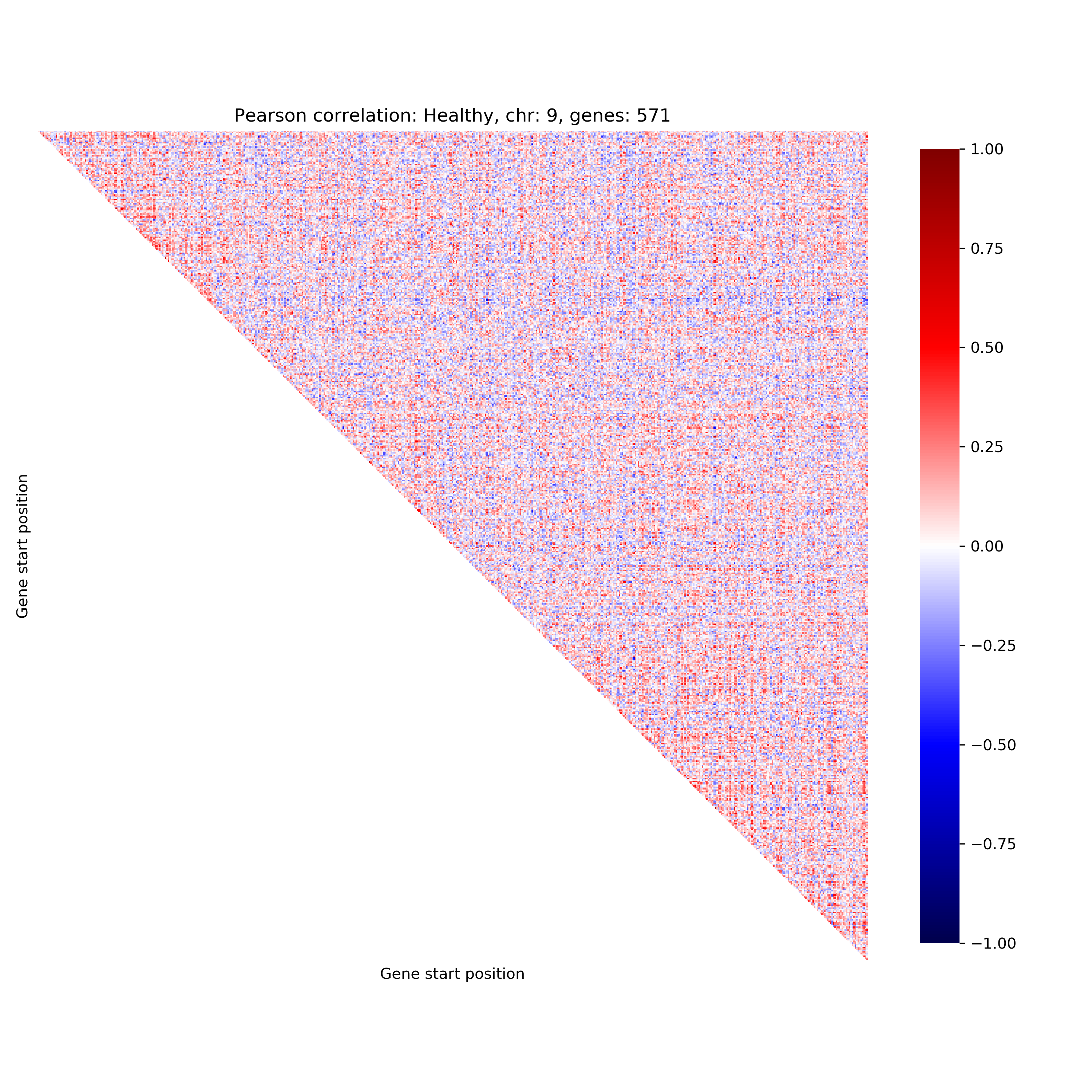

Supplement: Supplementary Material S1 — Excel file containing cross tables between subtype-samples and histological variables. [file DataSheet_1.zip › SuppMat2/Healthy-chr9.png]

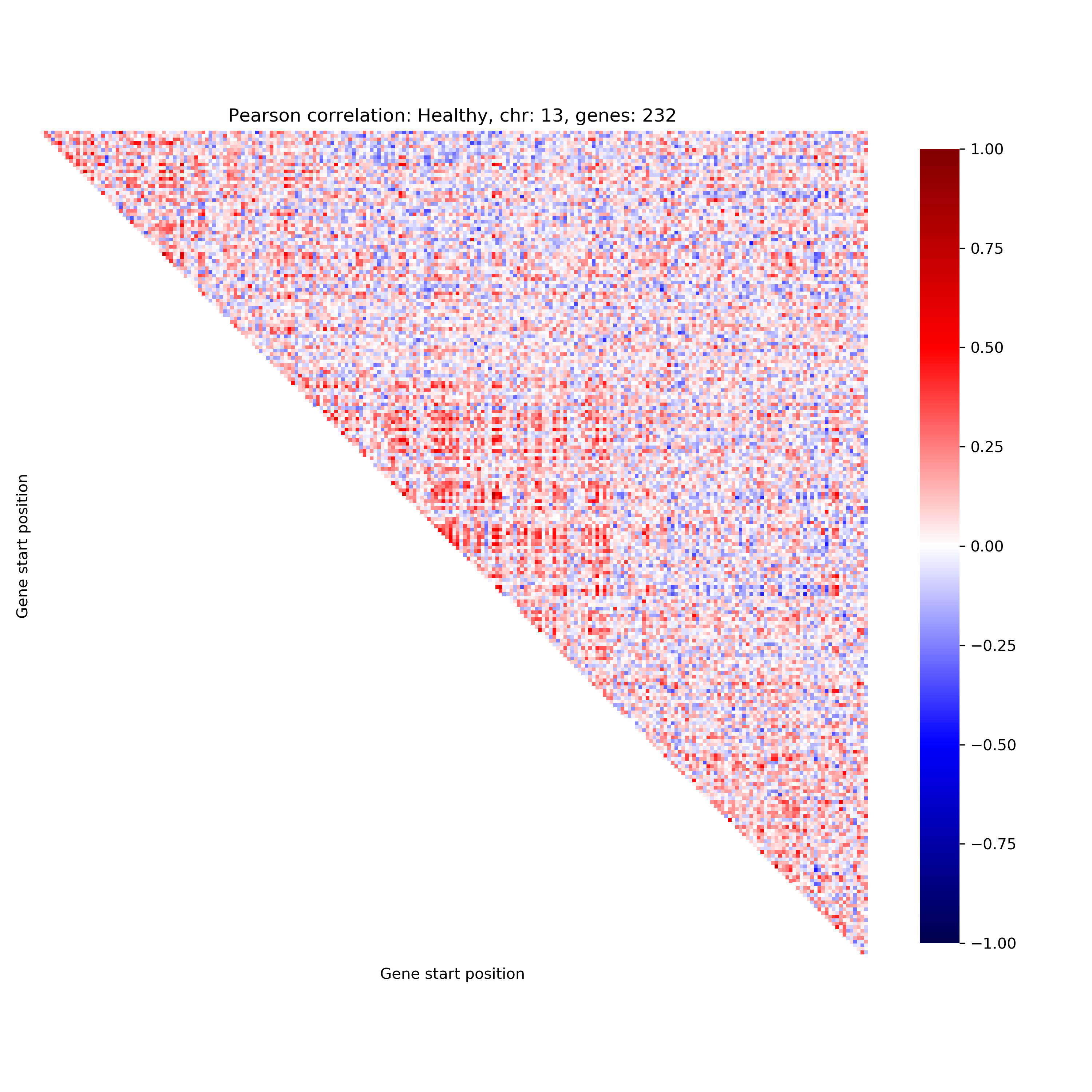

Supplement: Supplementary Material S1 — Excel file containing cross tables between subtype-samples and histological variables. [file DataSheet_1.zip › SuppMat2/Healthy-chr13.png]

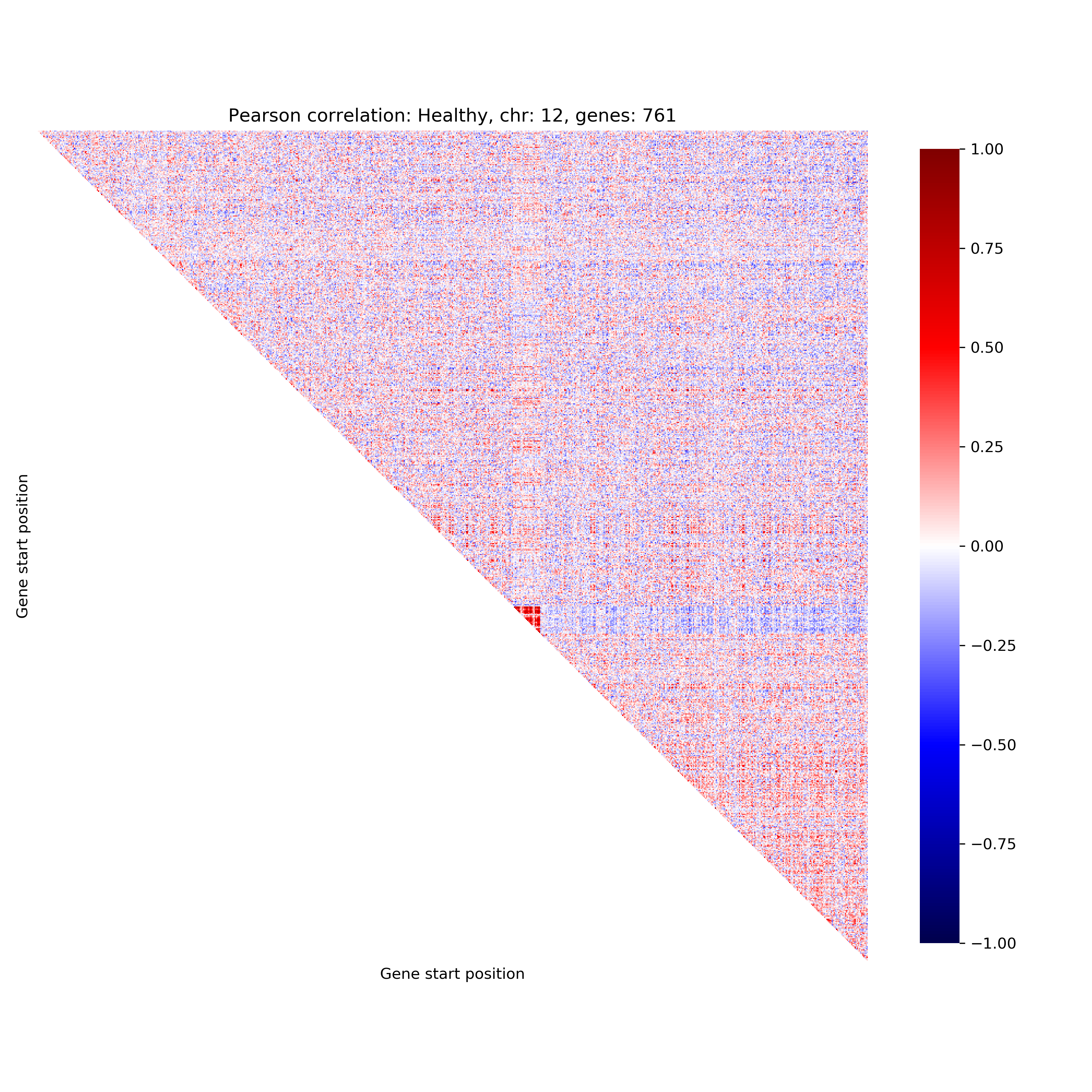

Supplement: Supplementary Material S1 — Excel file containing cross tables between subtype-samples and histological variables. [file DataSheet_1.zip › SuppMat2/Healthy-chr12.png]

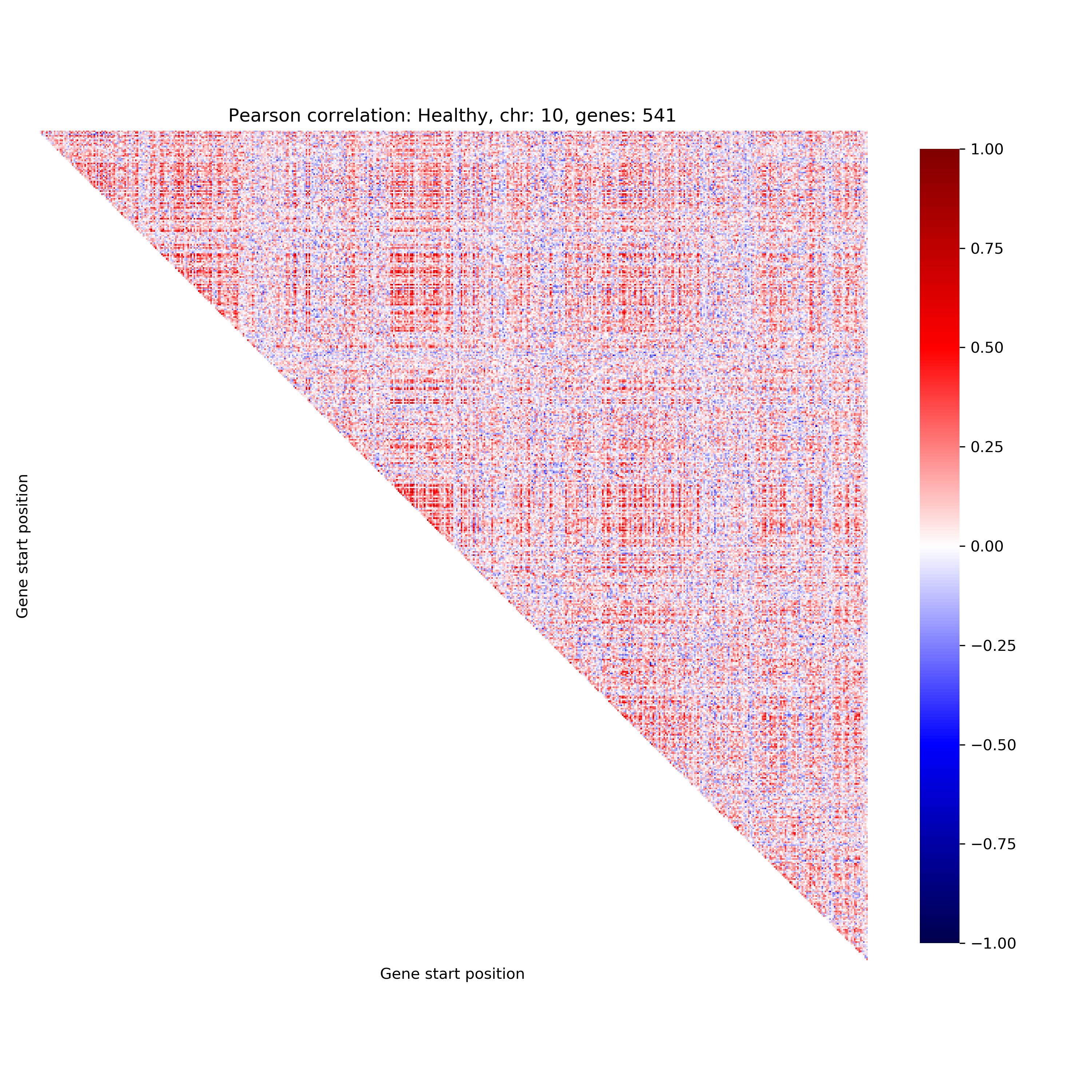

Supplement: Supplementary Material S1 — Excel file containing cross tables between subtype-samples and histological variables. [file DataSheet_1.zip › SuppMat2/Healthy-chr10.png]

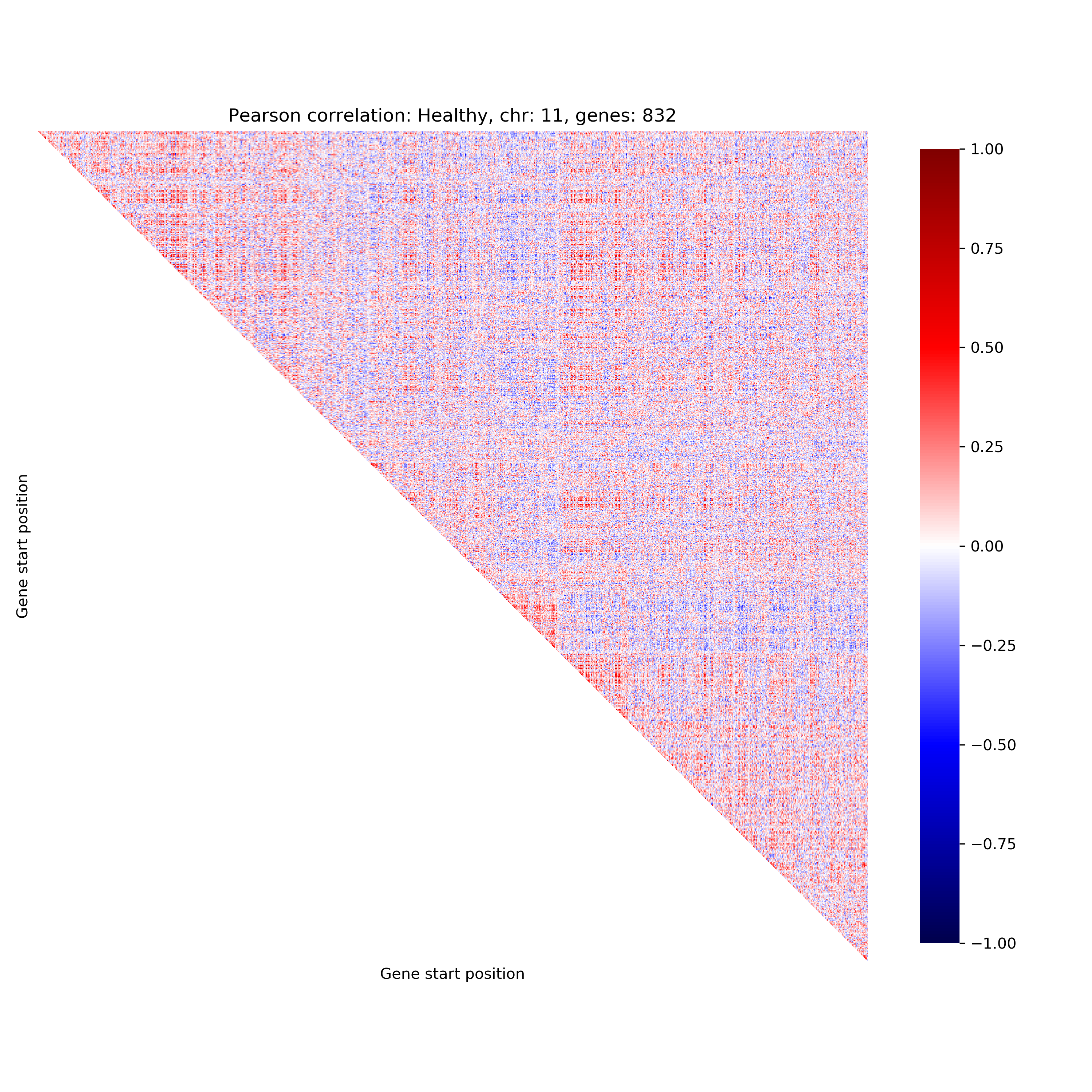

Supplement: Supplementary Material S1 — Excel file containing cross tables between subtype-samples and histological variables. [file DataSheet_1.zip › SuppMat2/Healthy-chr11.png]

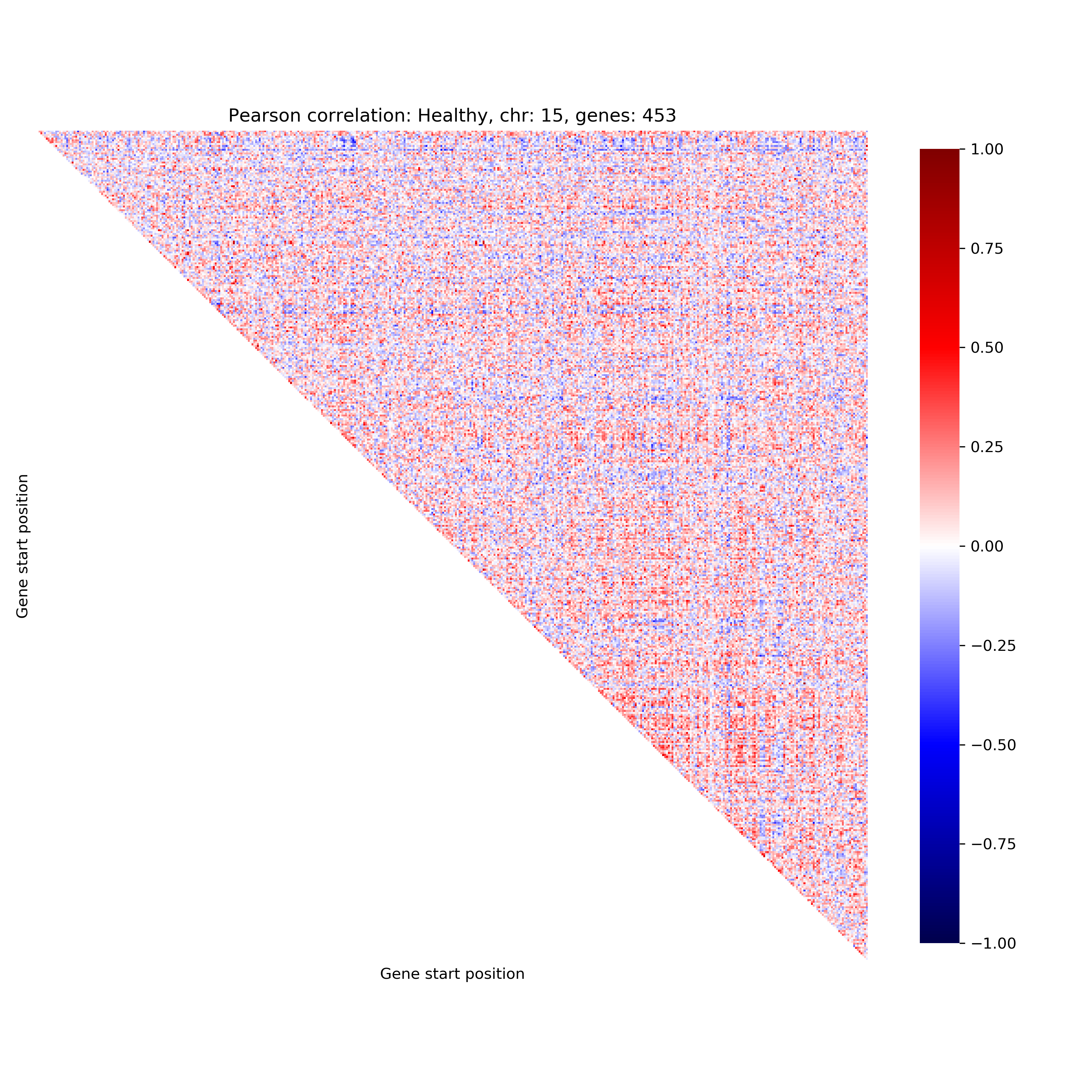

Supplement: Supplementary Material S1 — Excel file containing cross tables between subtype-samples and histological variables. [file DataSheet_1.zip › SuppMat2/Healthy-chr15.png]

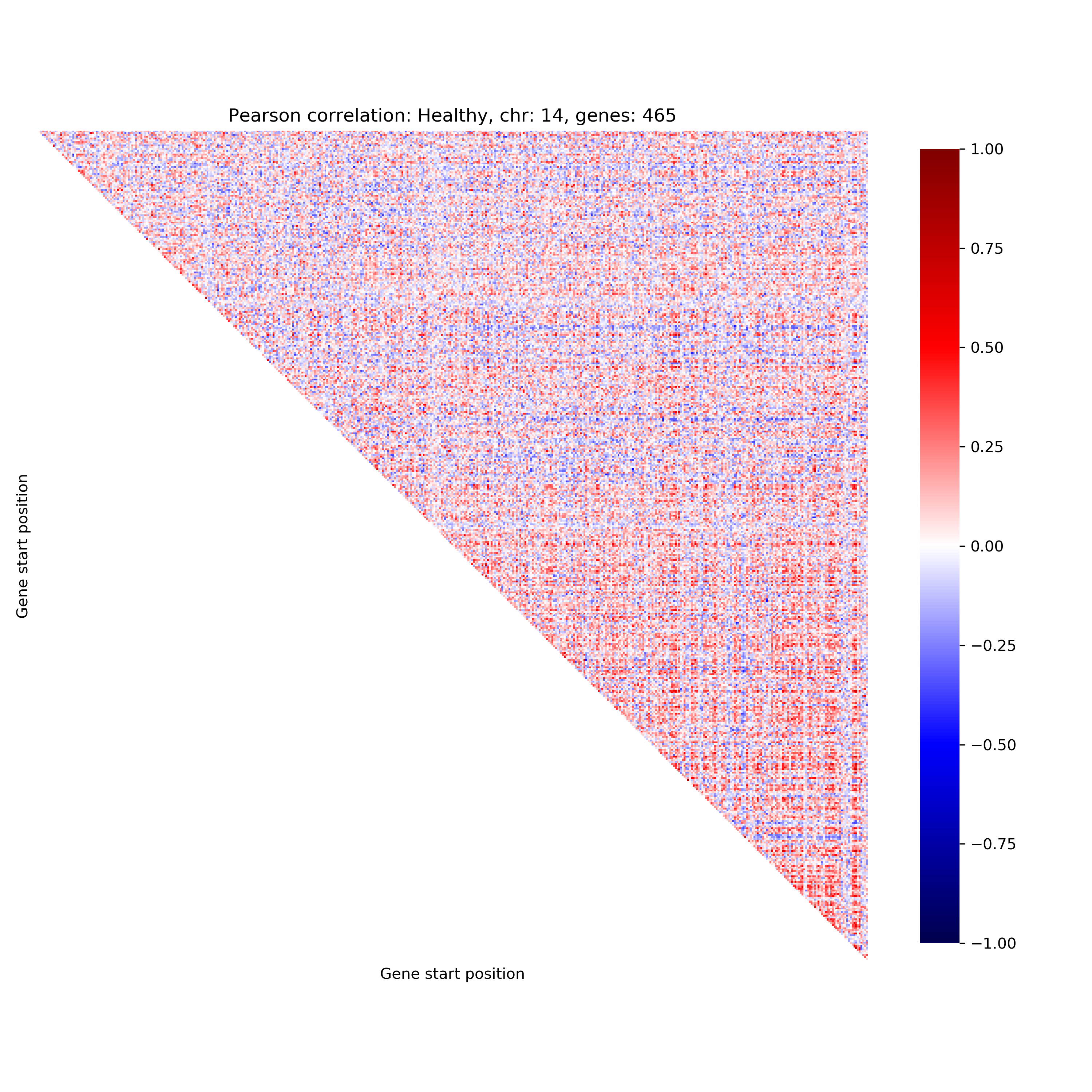

Supplement: Supplementary Material S1 — Excel file containing cross tables between subtype-samples and histological variables. [file DataSheet_1.zip › SuppMat2/Healthy-chr14.png]

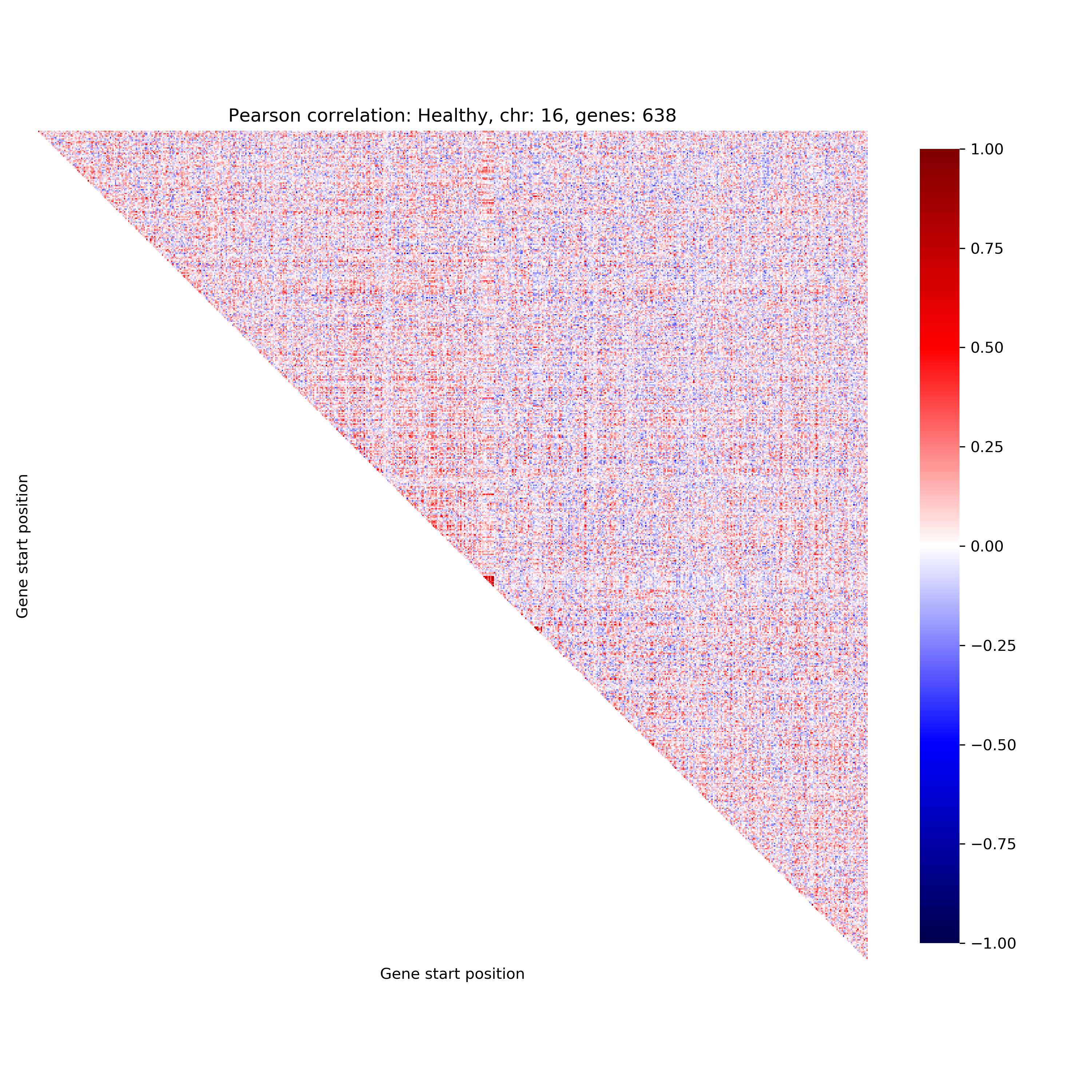

Supplement: Supplementary Material S1 — Excel file containing cross tables between subtype-samples and histological variables. [file DataSheet_1.zip › SuppMat2/Healthy-chr16.png]

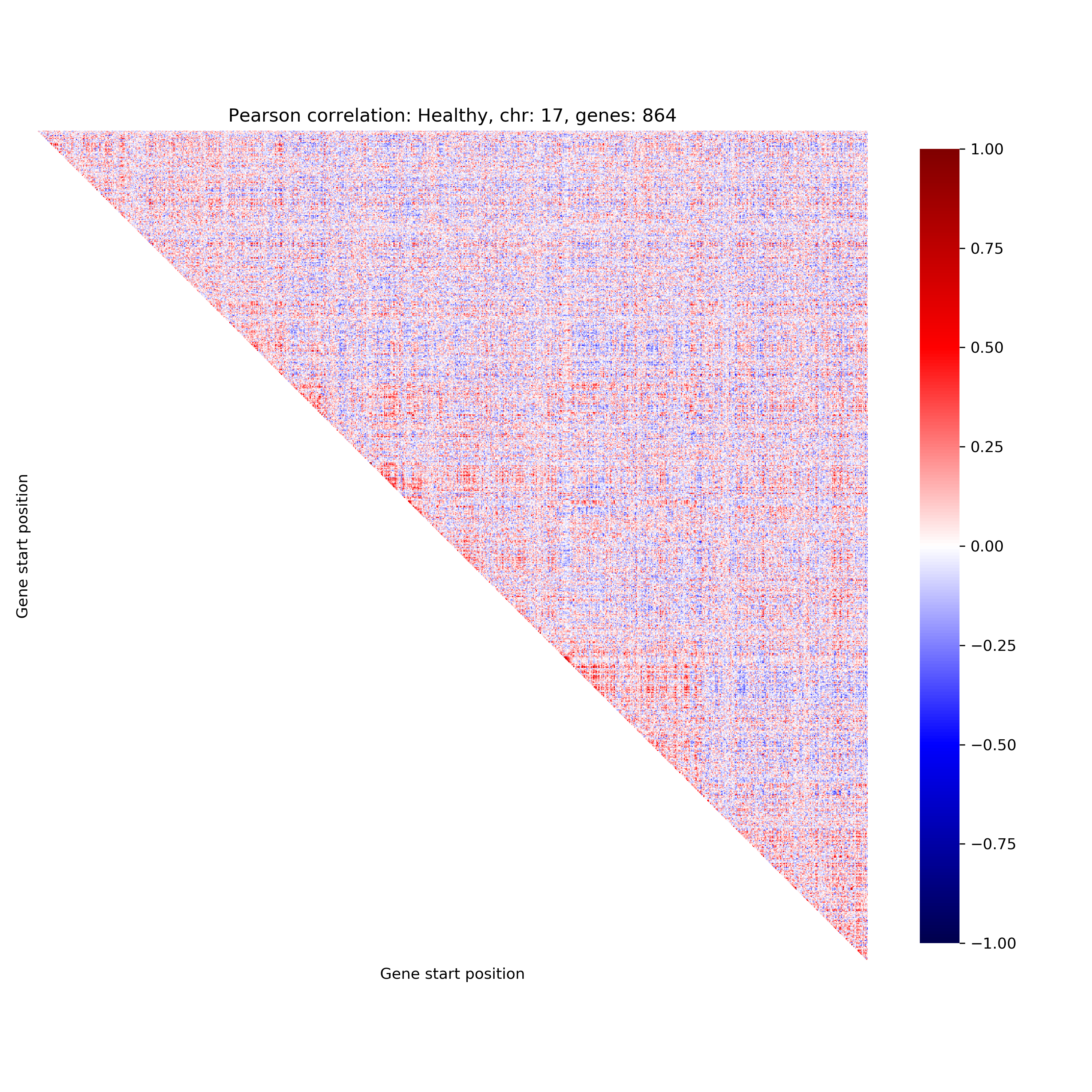

Supplement: Supplementary Material S1 — Excel file containing cross tables between subtype-samples and histological variables. [file DataSheet_1.zip › SuppMat2/Healthy-chr17.png]

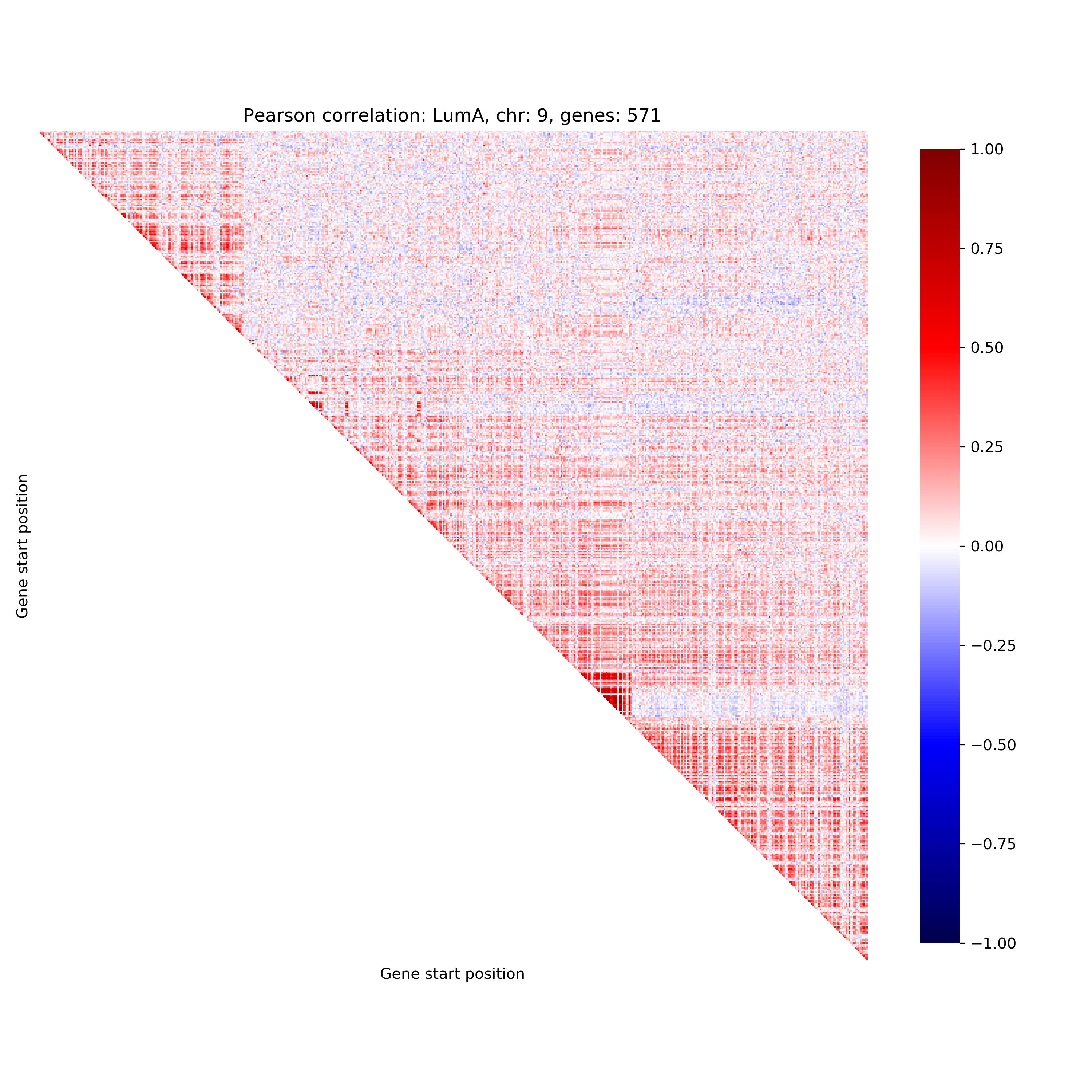

Supplement: Supplementary Material S2 — Heatmaps of Pearson correlation for each chromosome in the adjacent normal phenotype. The color code is the same than in Figure 1 . [file DataSheet_2.zip › SuppMat3/LumA-chr9.png]

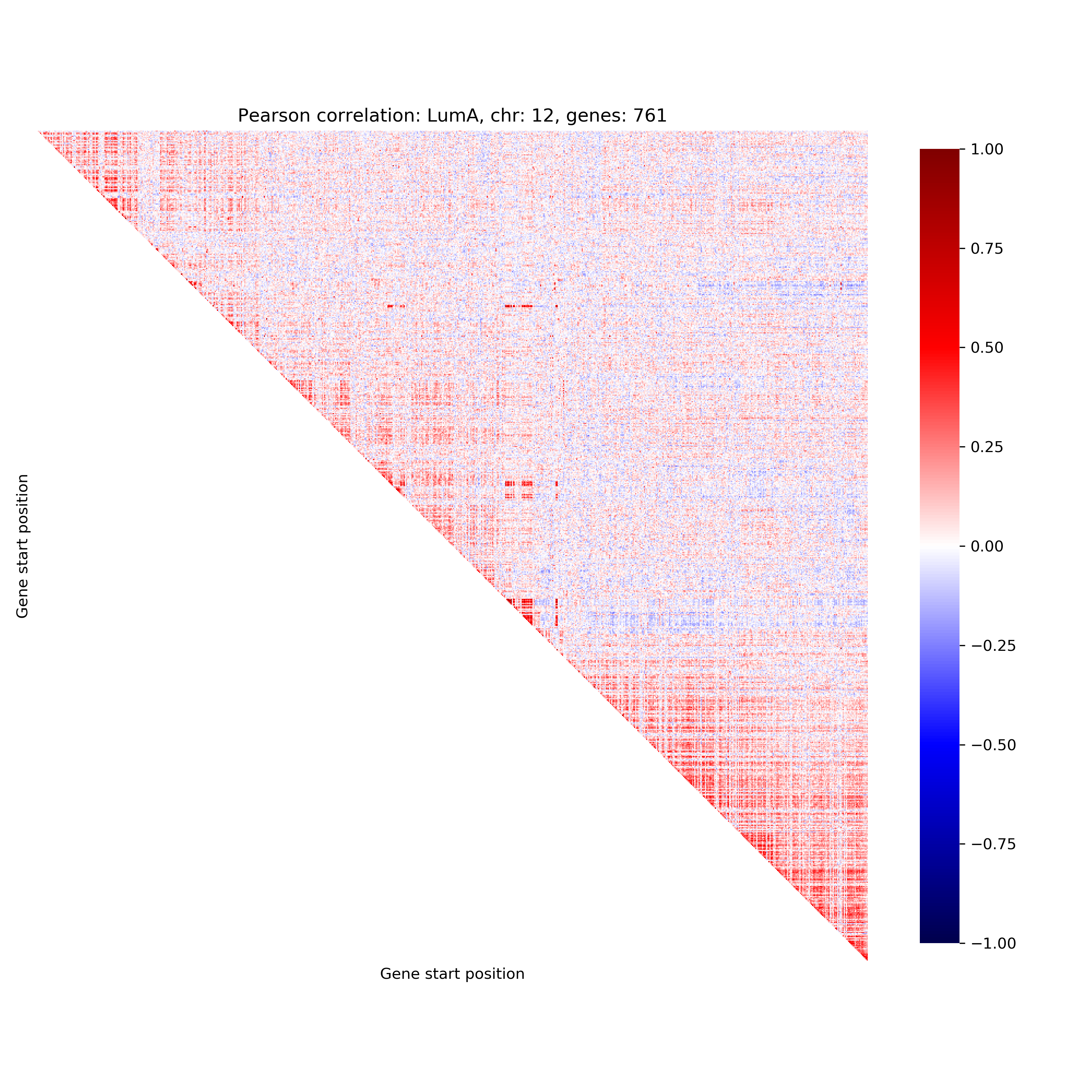

Supplement: Supplementary Material S2 — Heatmaps of Pearson correlation for each chromosome in the adjacent normal phenotype. The color code is the same than in Figure 1 . [file DataSheet_2.zip › SuppMat3/LumA-chr12.png]

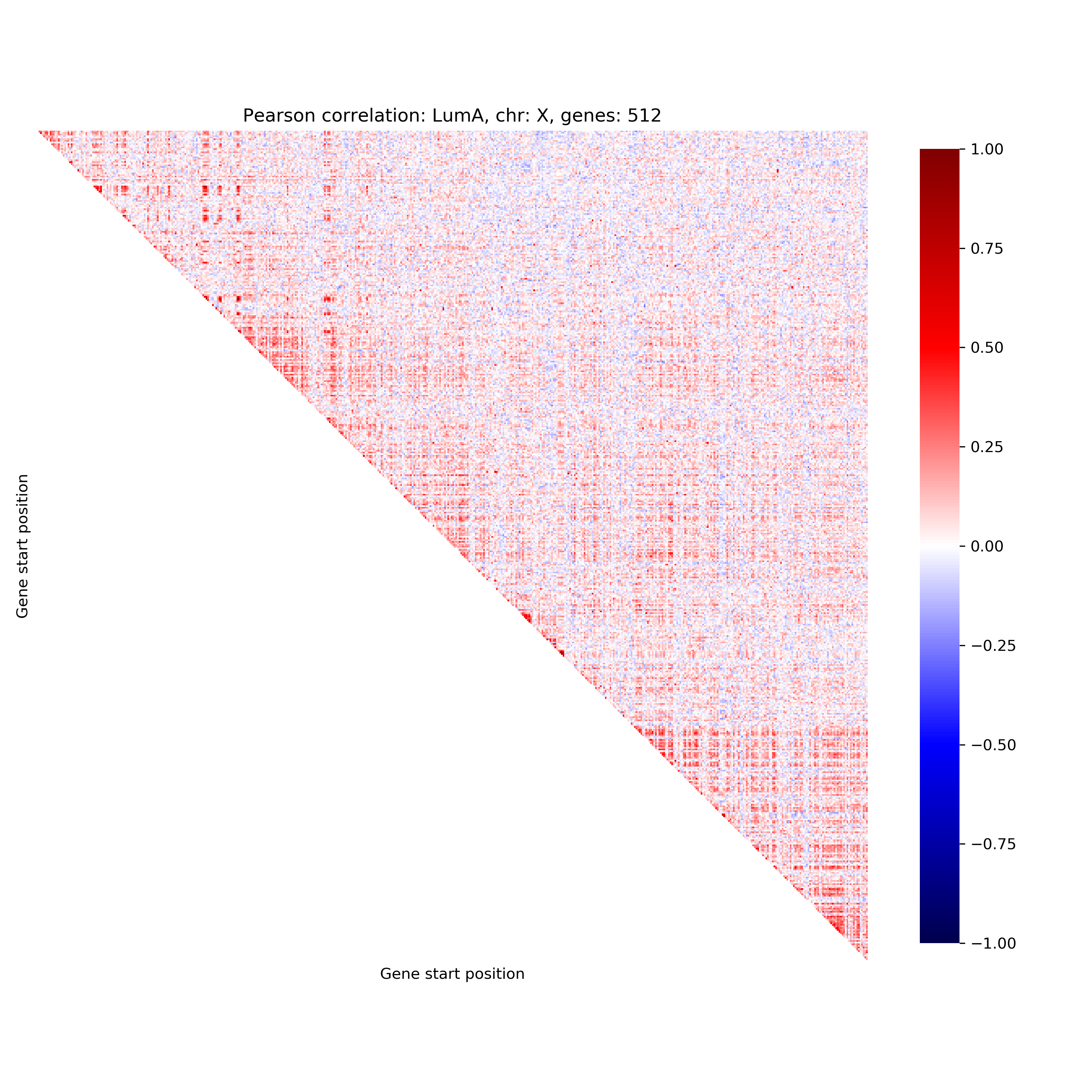

Supplement: Supplementary Material S2 — Heatmaps of Pearson correlation for each chromosome in the adjacent normal phenotype. The color code is the same than in Figure 1 . [file DataSheet_2.zip › SuppMat3/LumA-chrX.png]

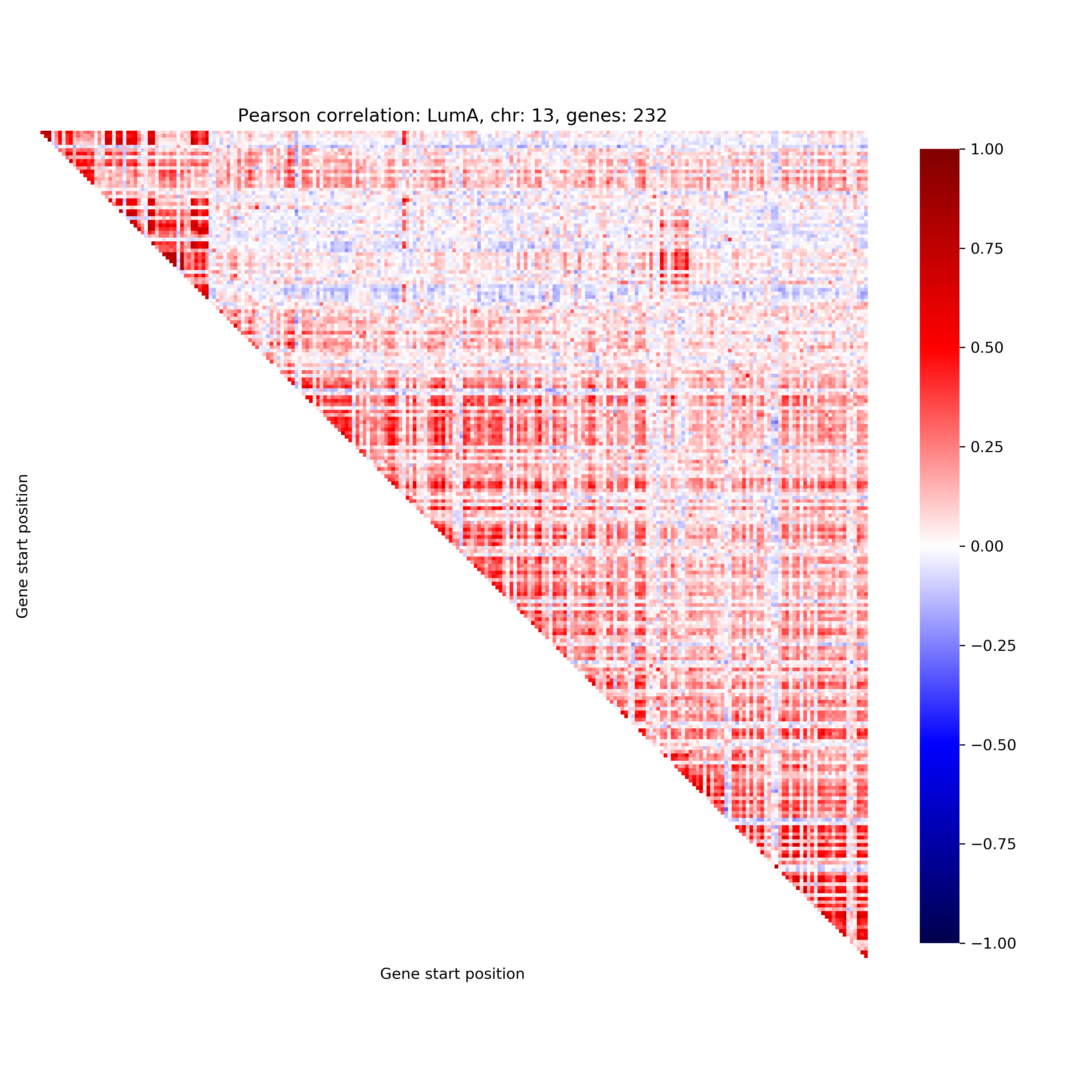

Supplement: Supplementary Material S2 — Heatmaps of Pearson correlation for each chromosome in the adjacent normal phenotype. The color code is the same than in Figure 1 . [file DataSheet_2.zip › SuppMat3/LumA-chr13.png]

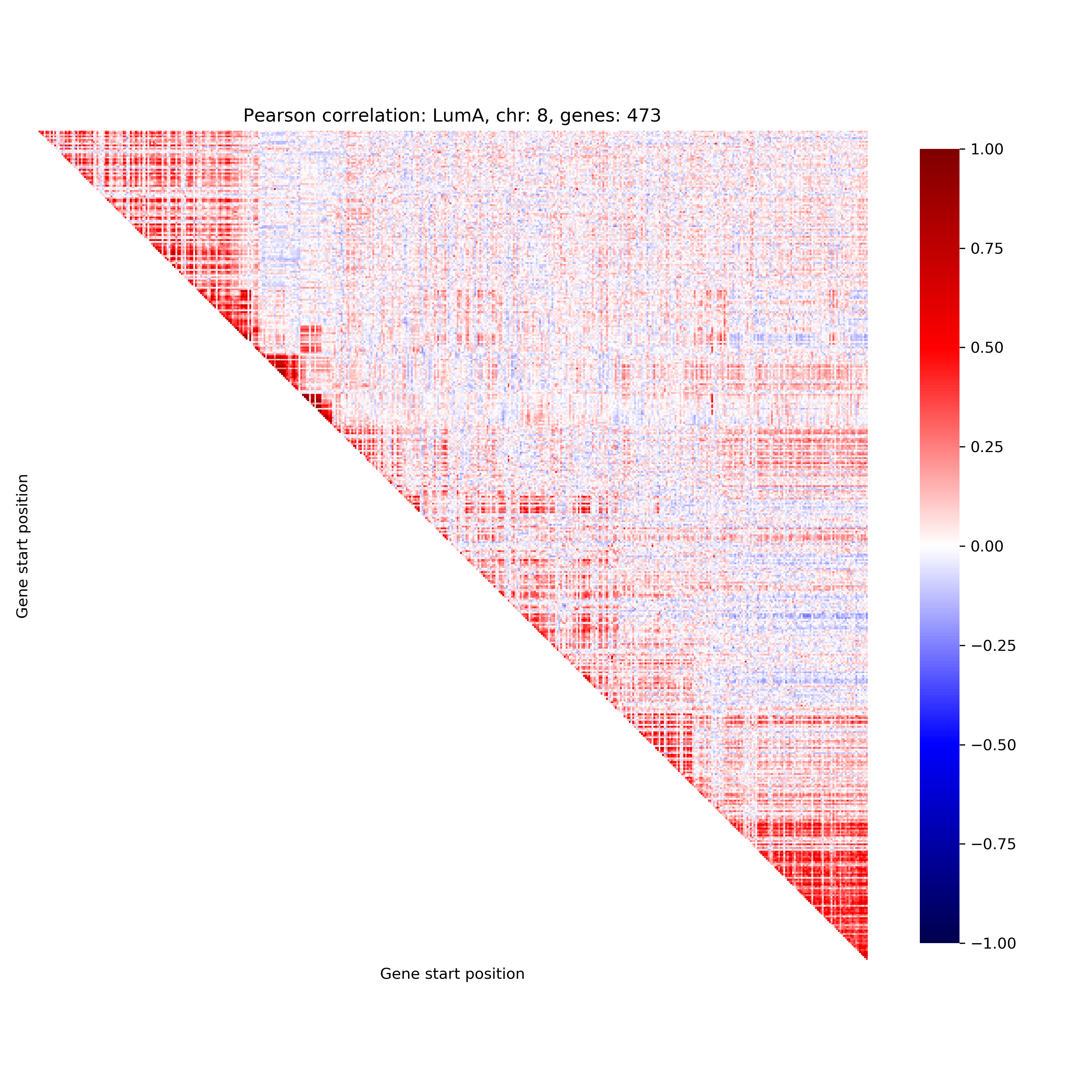

Supplement: Supplementary Material S2 — Heatmaps of Pearson correlation for each chromosome in the adjacent normal phenotype. The color code is the same than in Figure 1 . [file DataSheet_2.zip › SuppMat3/LumA-chr8.png]

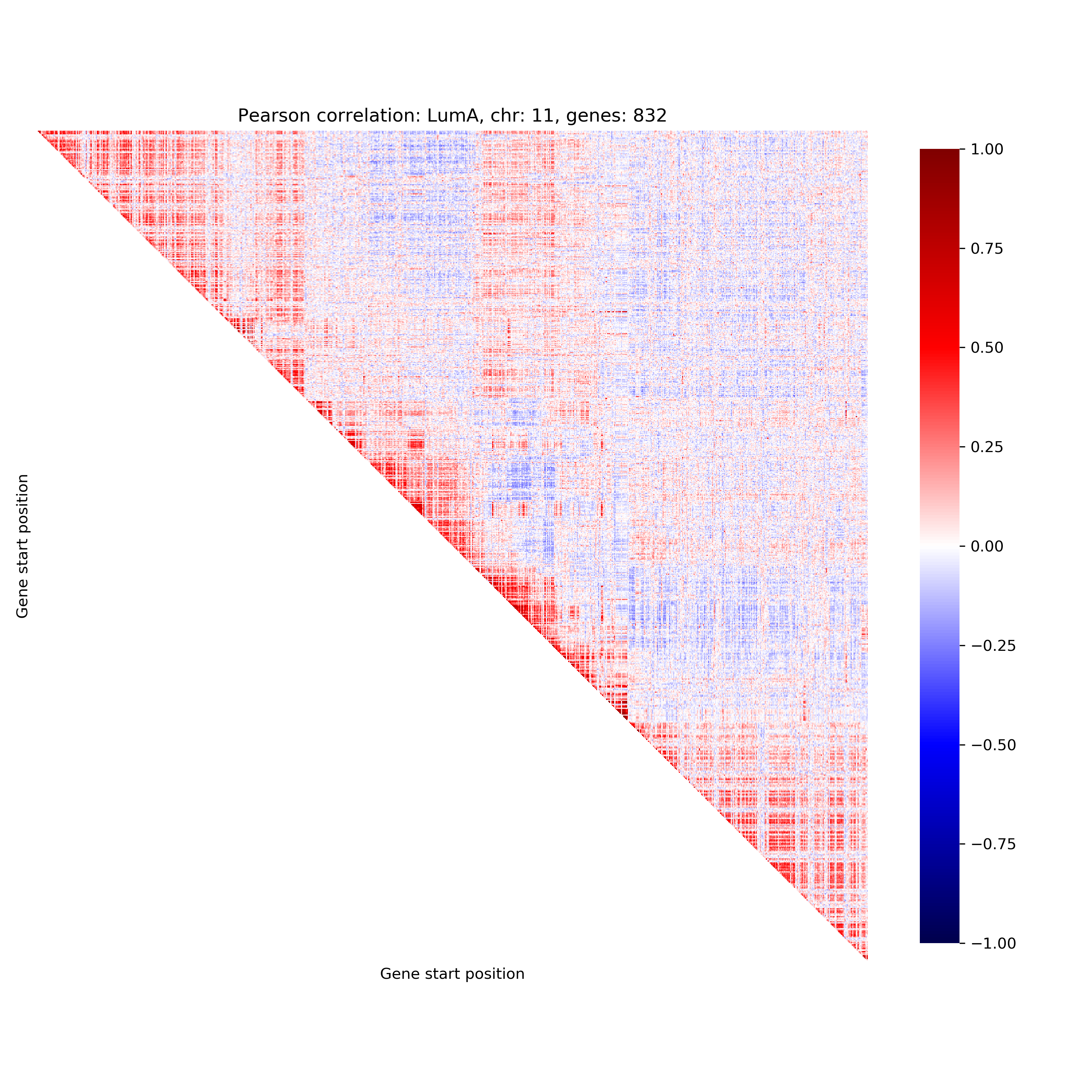

Supplement: Supplementary Material S2 — Heatmaps of Pearson correlation for each chromosome in the adjacent normal phenotype. The color code is the same than in Figure 1 . [file DataSheet_2.zip › SuppMat3/LumA-chr11.png]

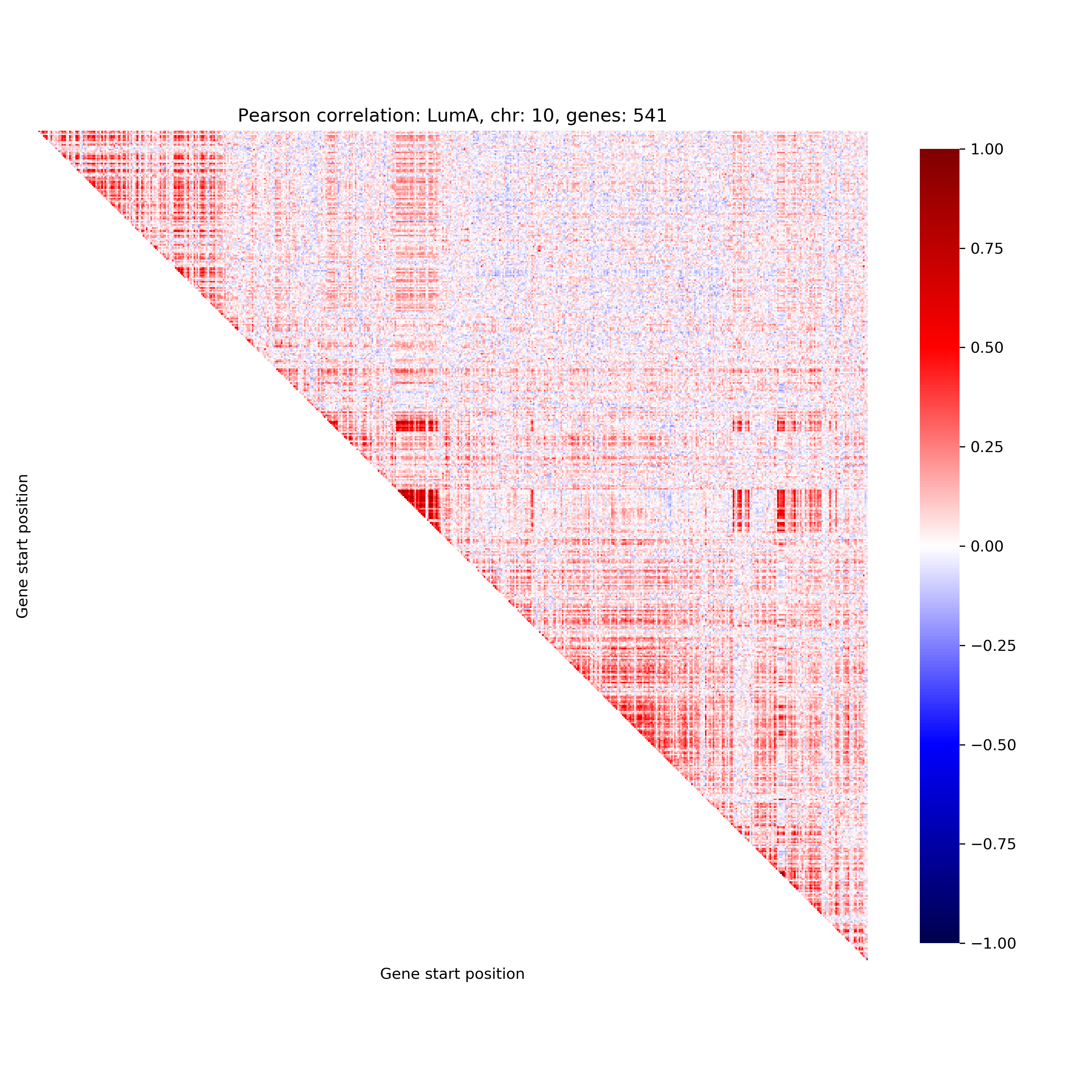

Supplement: Supplementary Material S2 — Heatmaps of Pearson correlation for each chromosome in the adjacent normal phenotype. The color code is the same than in Figure 1 . [file DataSheet_2.zip › SuppMat3/LumA-chr10.png]

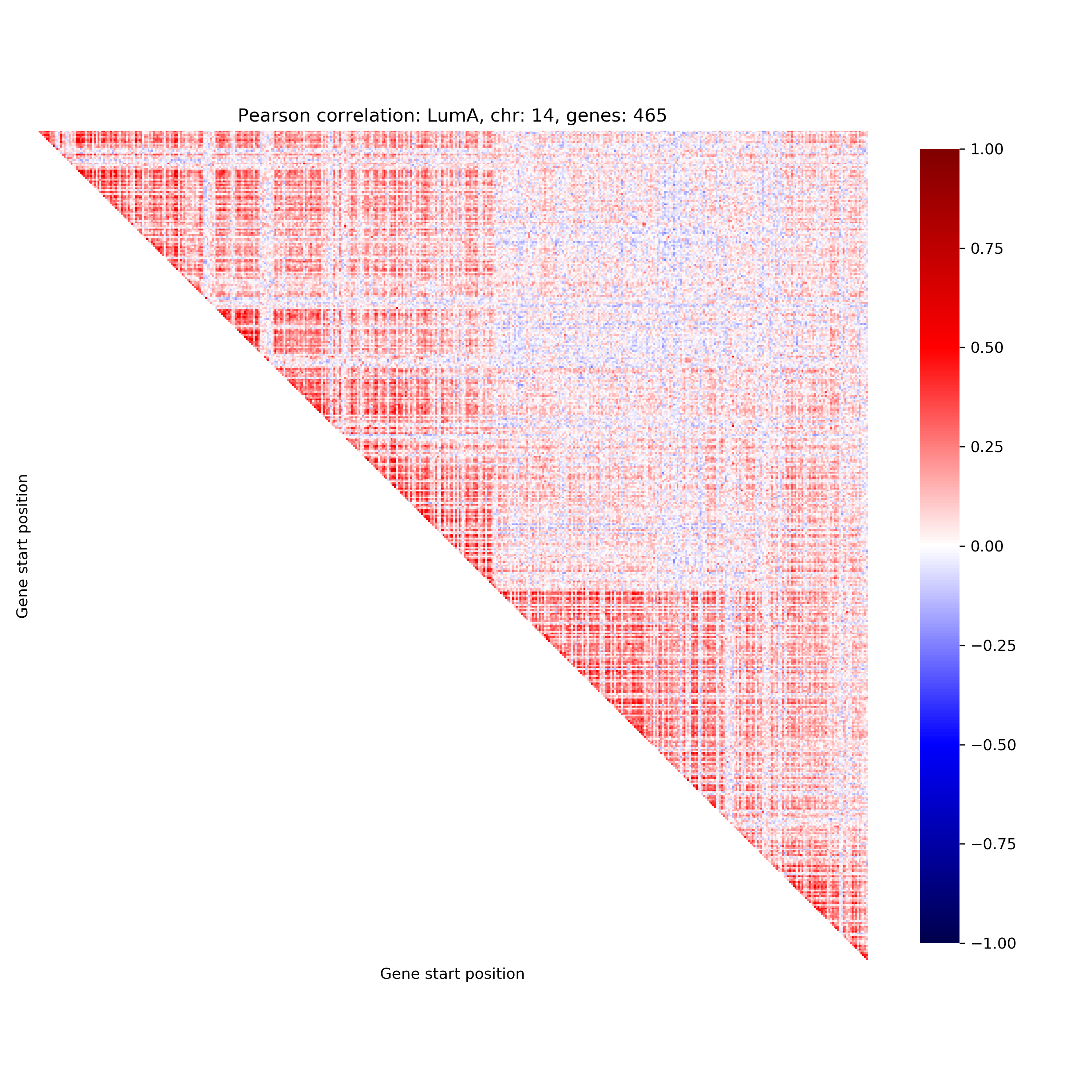

Supplement: Supplementary Material S2 — Heatmaps of Pearson correlation for each chromosome in the adjacent normal phenotype. The color code is the same than in Figure 1 . [file DataSheet_2.zip › SuppMat3/LumA-chr14.png]

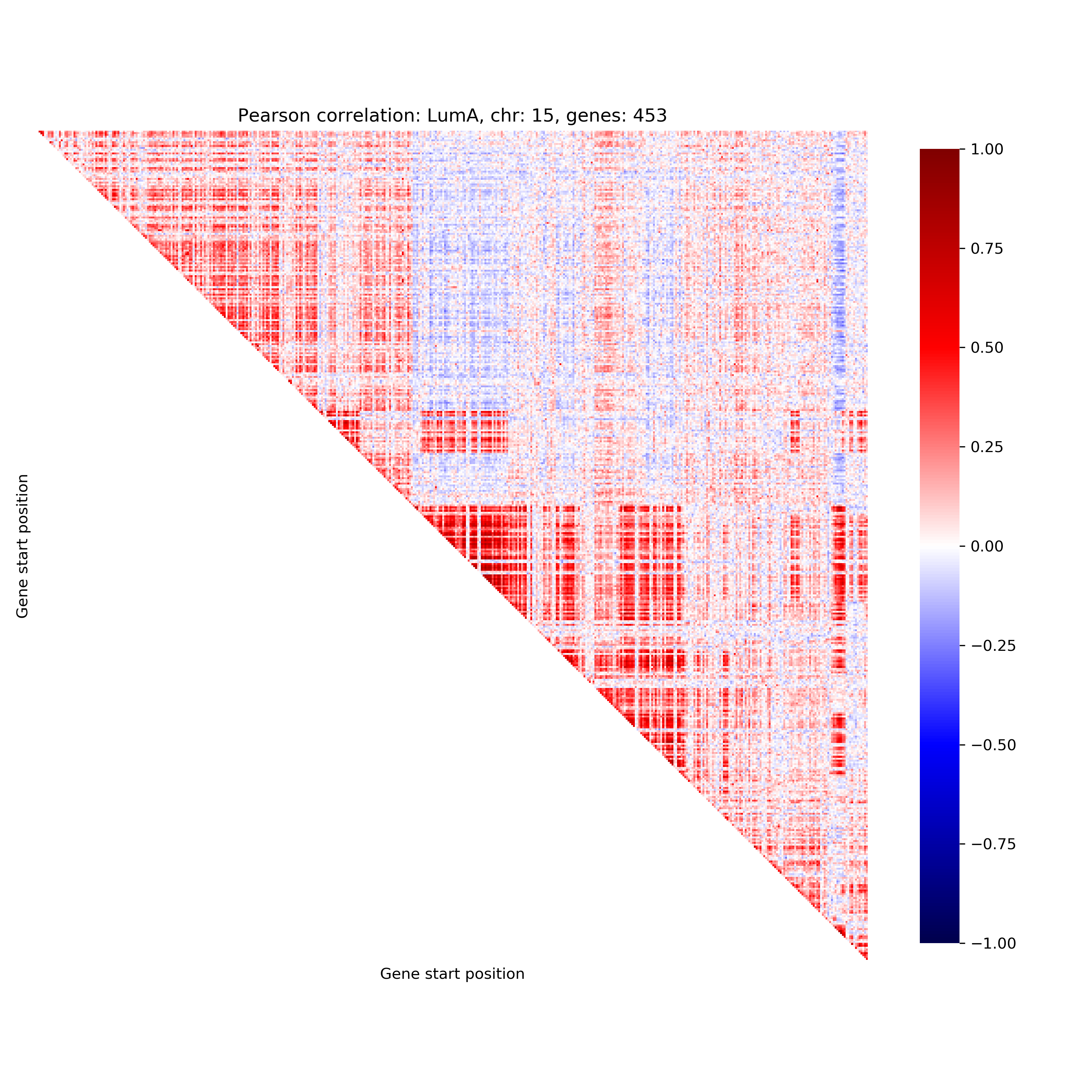

Supplement: Supplementary Material S2 — Heatmaps of Pearson correlation for each chromosome in the adjacent normal phenotype. The color code is the same than in Figure 1 . [file DataSheet_2.zip › SuppMat3/LumA-chr15.png]

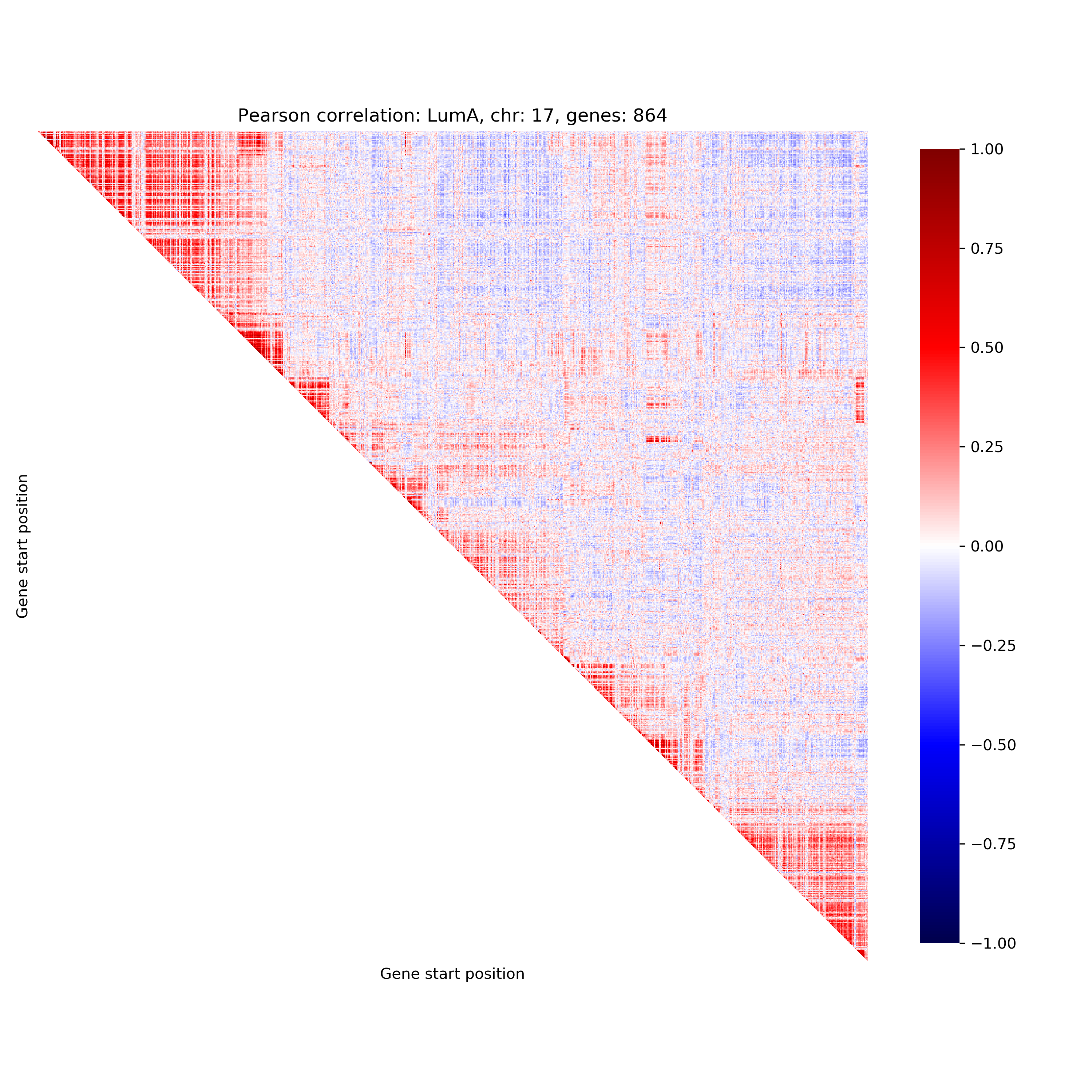

Supplement: Supplementary Material S2 — Heatmaps of Pearson correlation for each chromosome in the adjacent normal phenotype. The color code is the same than in Figure 1 . [file DataSheet_2.zip › SuppMat3/LumA-chr17.png]

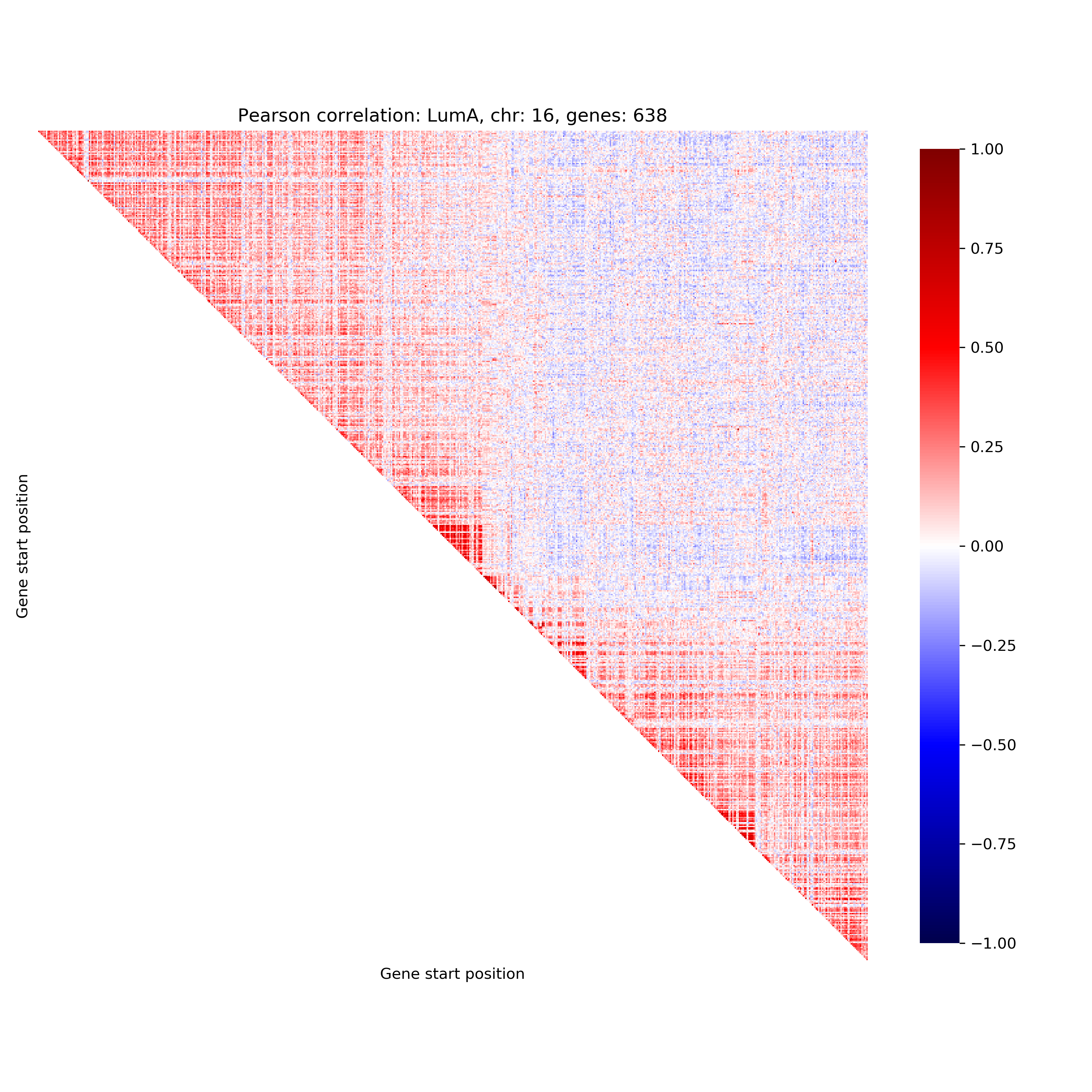

Supplement: Supplementary Material S2 — Heatmaps of Pearson correlation for each chromosome in the adjacent normal phenotype. The color code is the same than in Figure 1 . [file DataSheet_2.zip › SuppMat3/LumA-chr16.png]

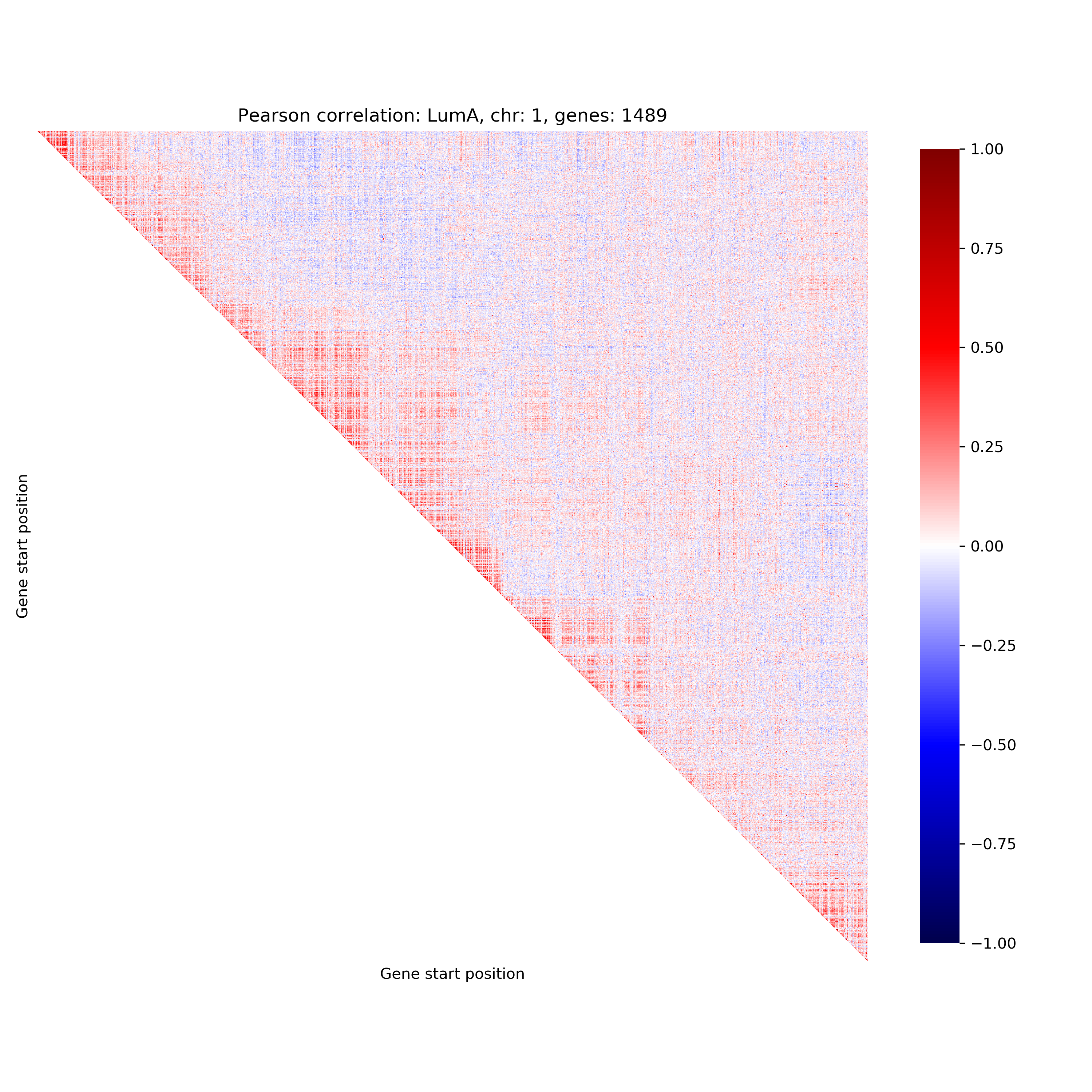

Supplement: Supplementary Material S2 — Heatmaps of Pearson correlation for each chromosome in the adjacent normal phenotype. The color code is the same than in Figure 1 . [file DataSheet_2.zip › SuppMat3/LumA-chr1.png]

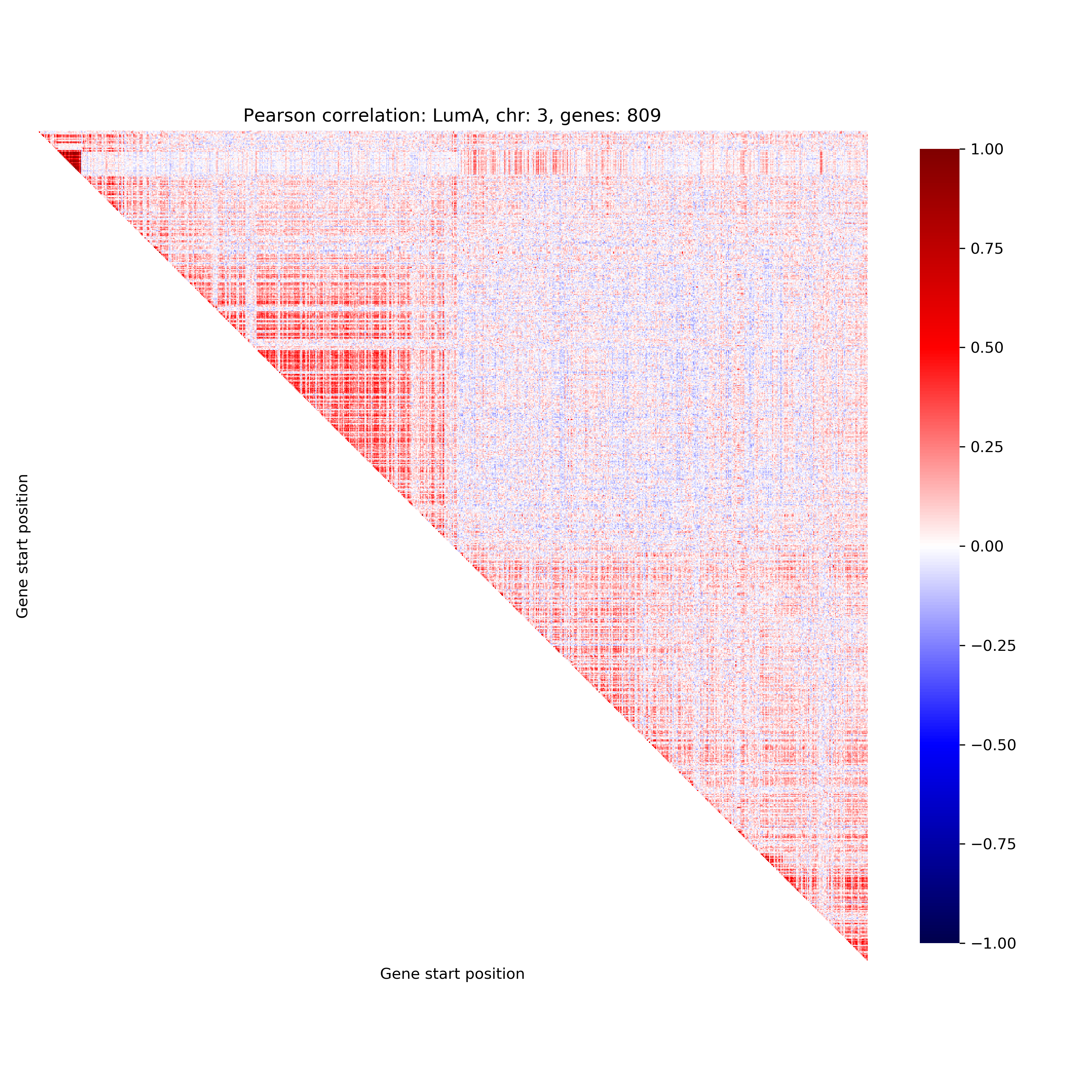

Supplement: Supplementary Material S2 — Heatmaps of Pearson correlation for each chromosome in the adjacent normal phenotype. The color code is the same than in Figure 1 . [file DataSheet_2.zip › SuppMat3/LumA-chr3.png]

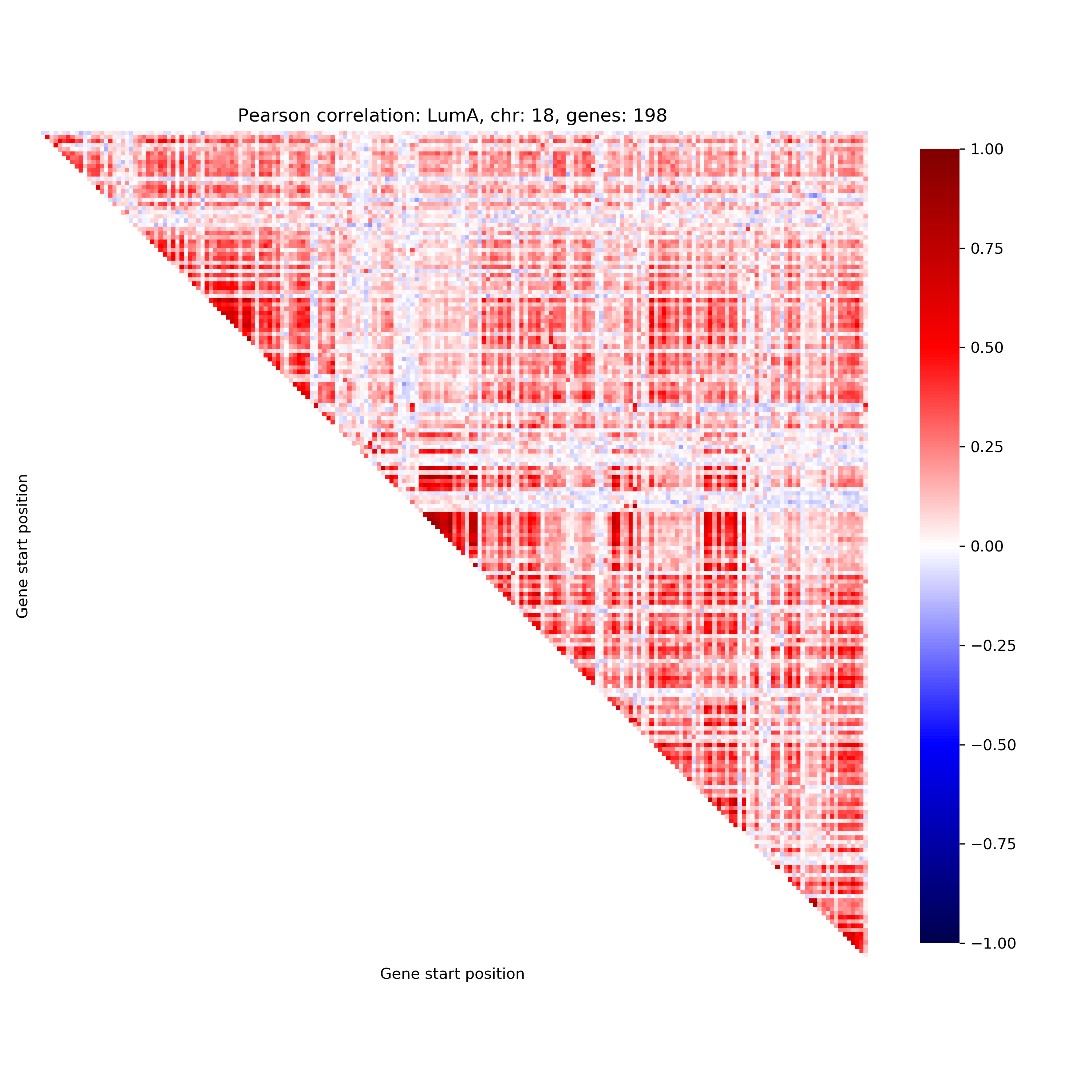

Supplement: Supplementary Material S2 — Heatmaps of Pearson correlation for each chromosome in the adjacent normal phenotype. The color code is the same than in Figure 1 . [file DataSheet_2.zip › SuppMat3/LumA-chr18.png]

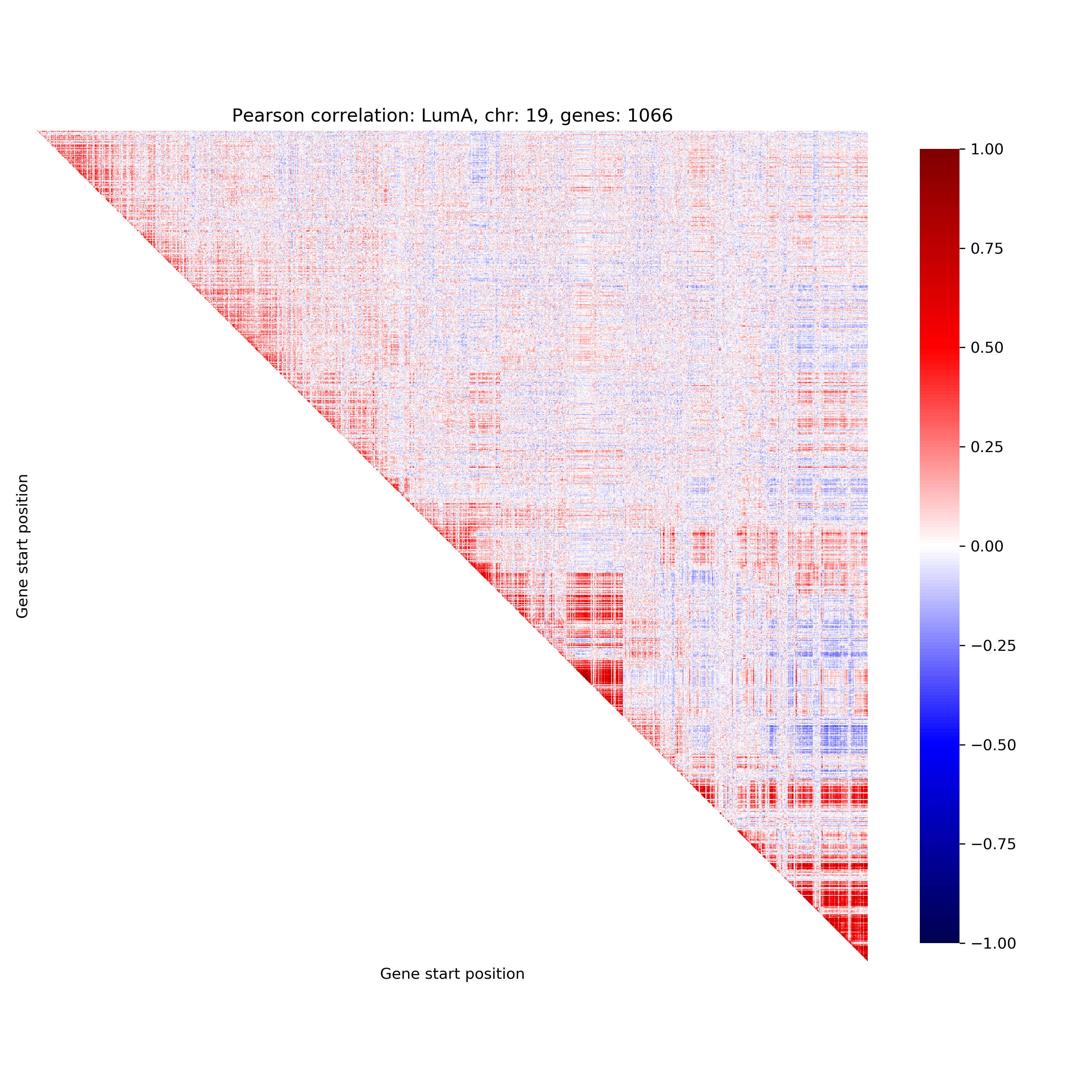

Supplement: Supplementary Material S2 — Heatmaps of Pearson correlation for each chromosome in the adjacent normal phenotype. The color code is the same than in Figure 1 . [file DataSheet_2.zip › SuppMat3/LumA-chr19.png]

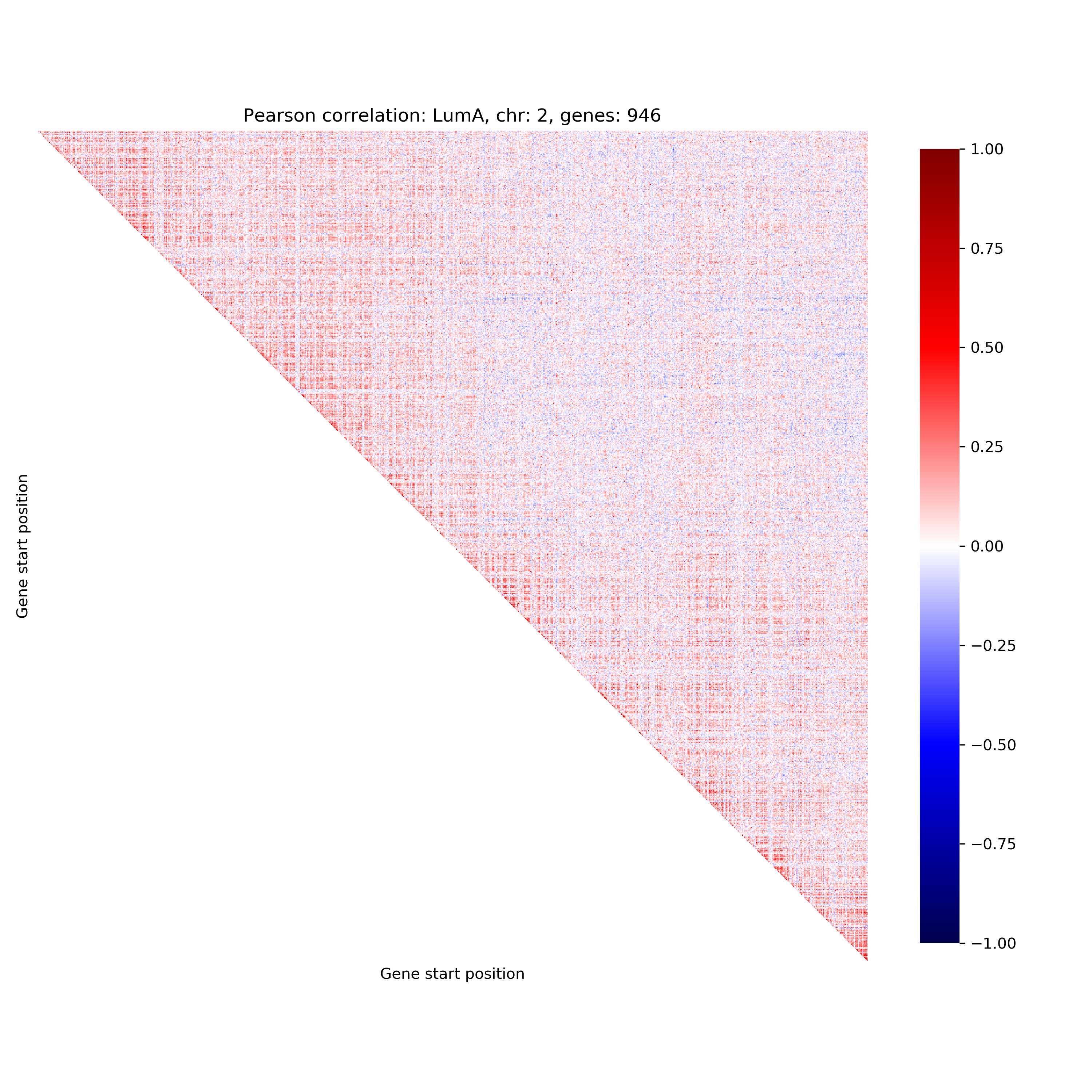

Supplement: Supplementary Material S2 — Heatmaps of Pearson correlation for each chromosome in the adjacent normal phenotype. The color code is the same than in Figure 1 . [file DataSheet_2.zip › SuppMat3/LumA-chr2.png]

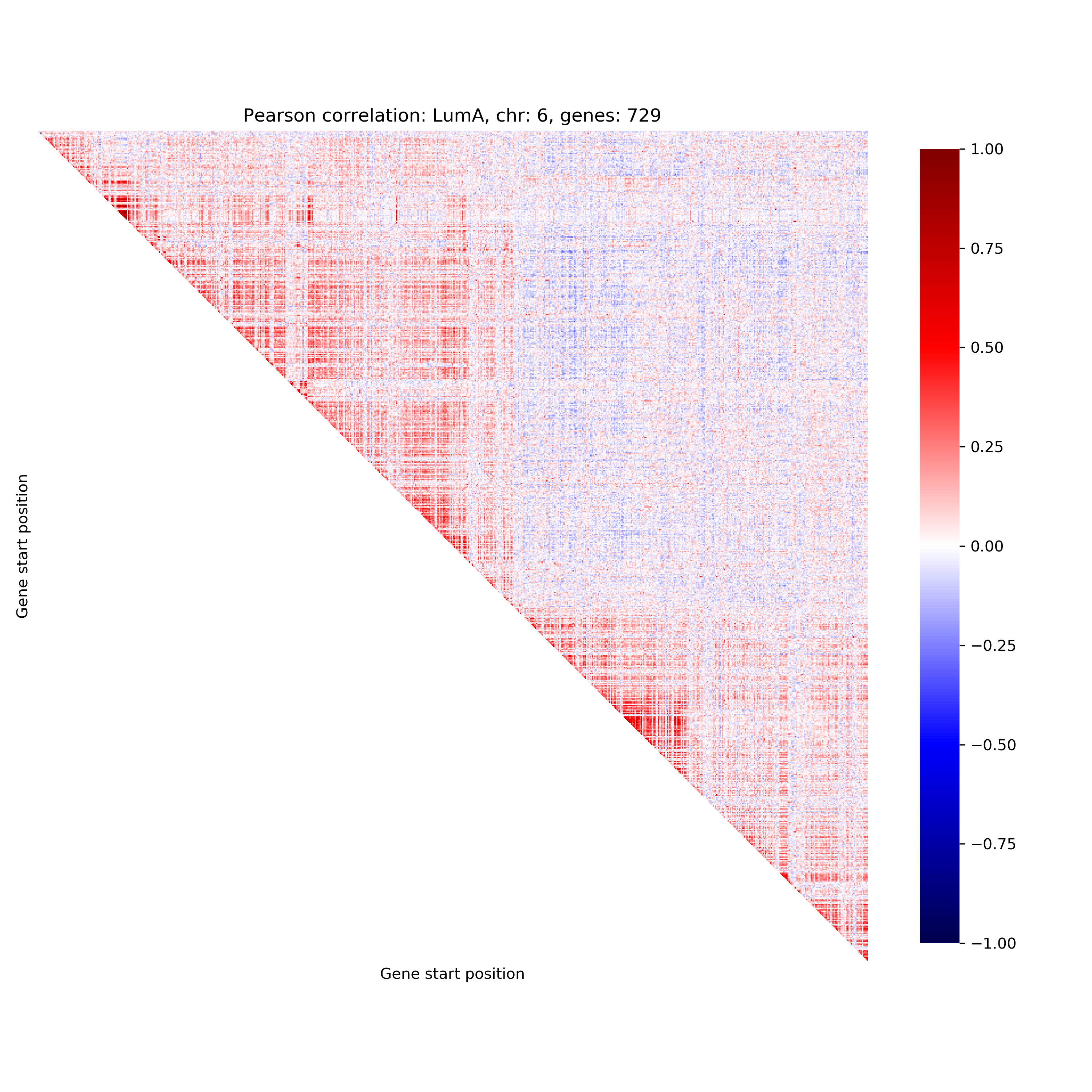

Supplement: Supplementary Material S2 — Heatmaps of Pearson correlation for each chromosome in the adjacent normal phenotype. The color code is the same than in Figure 1 . [file DataSheet_2.zip › SuppMat3/LumA-chr6.png]

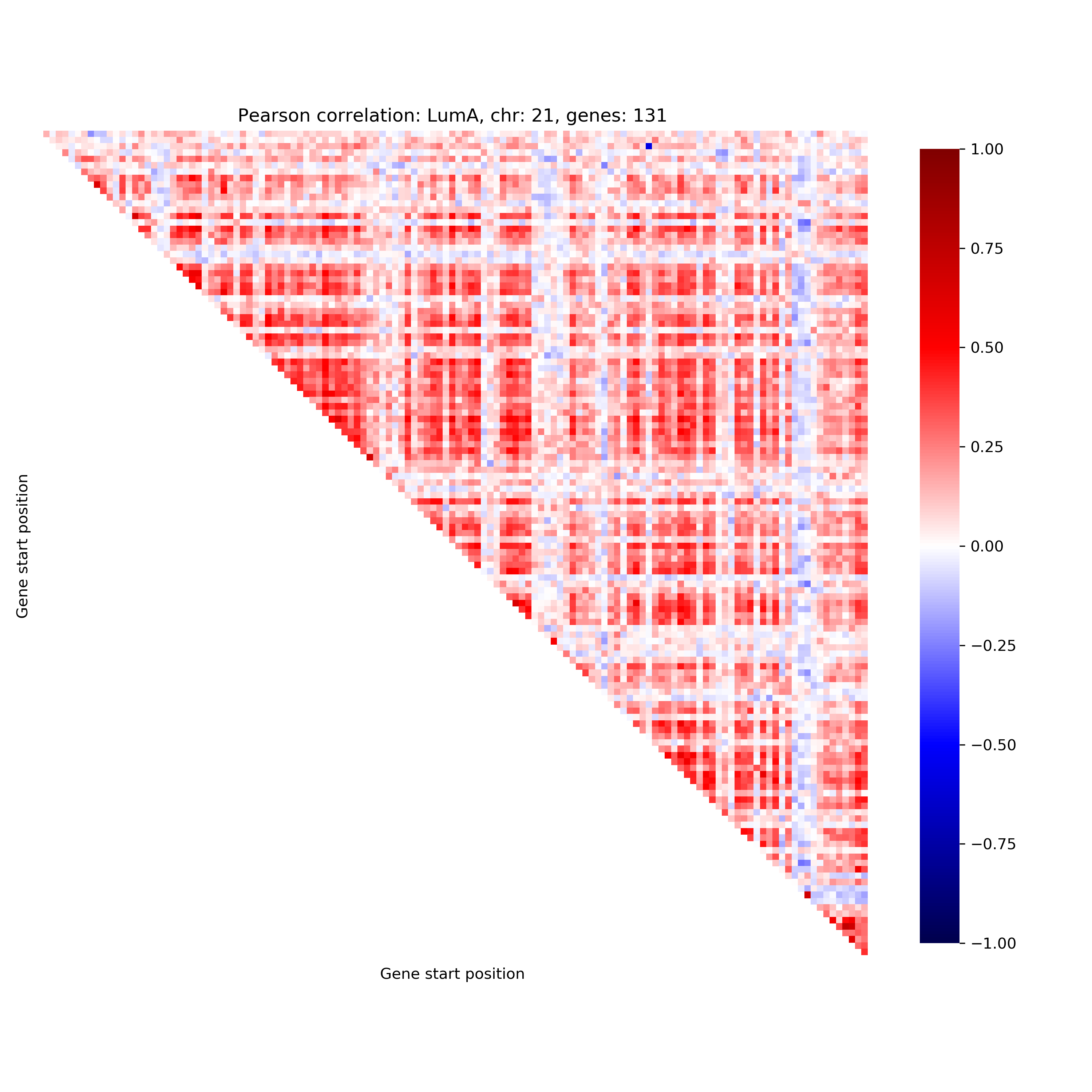

Supplement: Supplementary Material S2 — Heatmaps of Pearson correlation for each chromosome in the adjacent normal phenotype. The color code is the same than in Figure 1 . [file DataSheet_2.zip › SuppMat3/LumA-chr21.png]

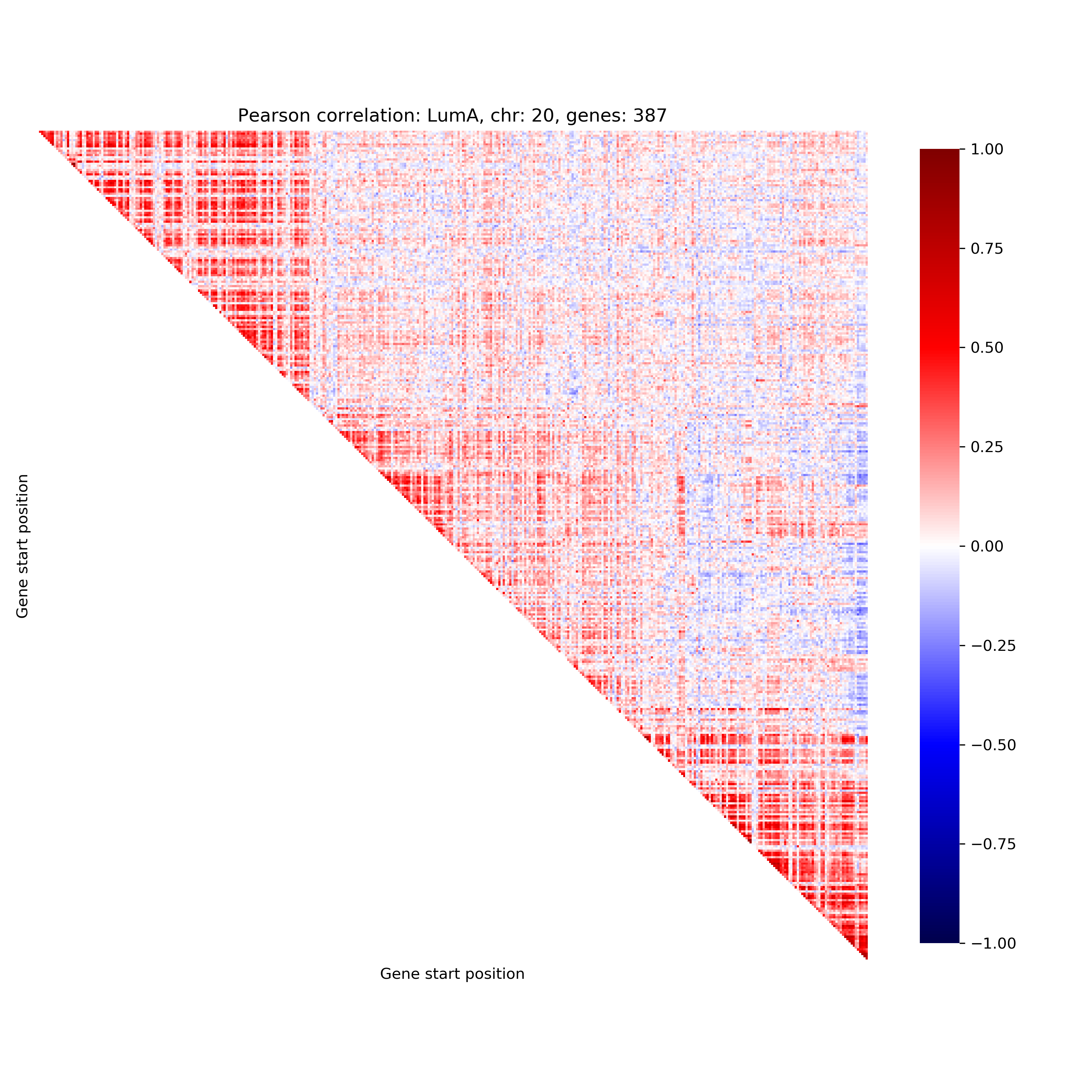

Supplement: Supplementary Material S2 — Heatmaps of Pearson correlation for each chromosome in the adjacent normal phenotype. The color code is the same than in Figure 1 . [file DataSheet_2.zip › SuppMat3/LumA-chr20.png]

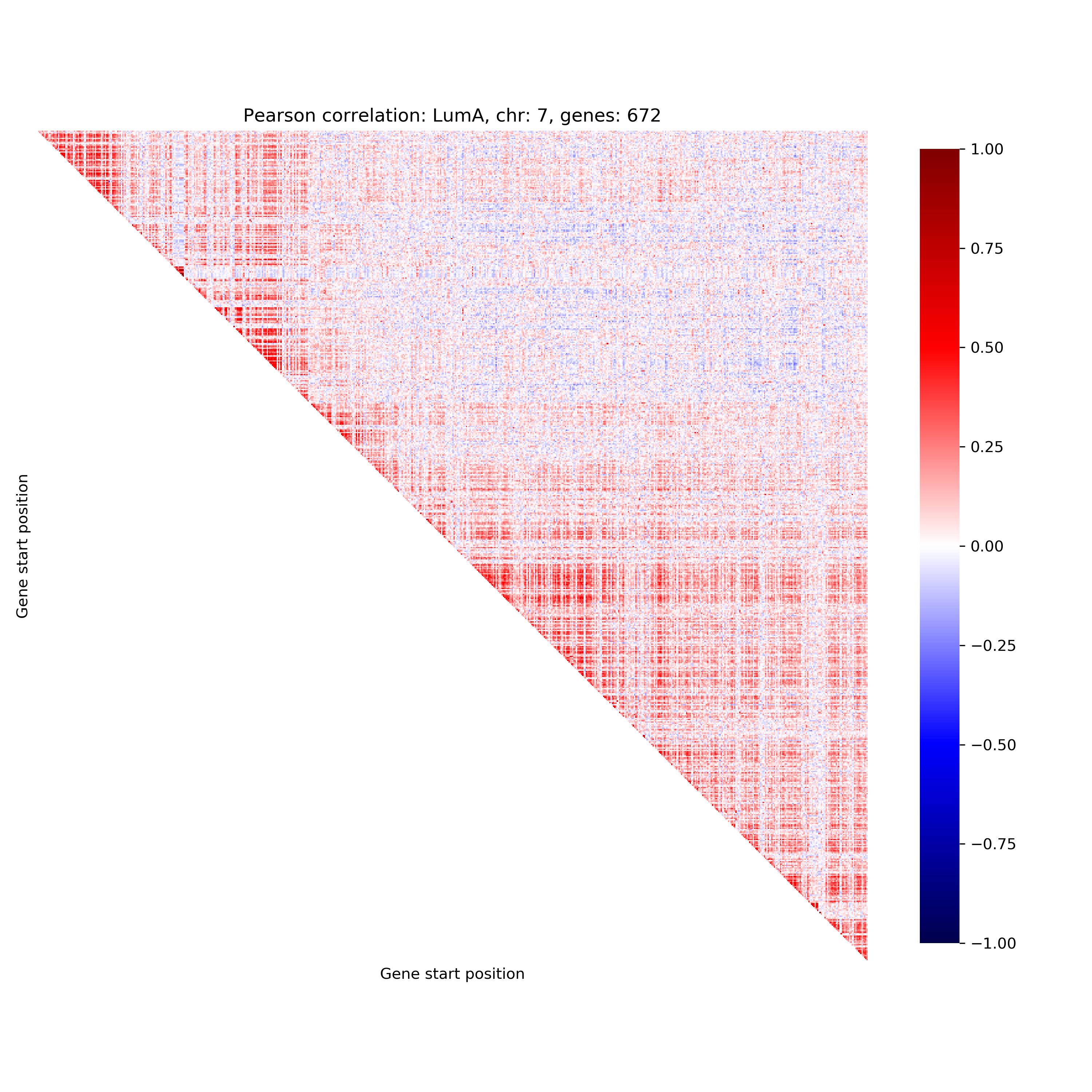

Supplement: Supplementary Material S2 — Heatmaps of Pearson correlation for each chromosome in the adjacent normal phenotype. The color code is the same than in Figure 1 . [file DataSheet_2.zip › SuppMat3/LumA-chr7.png]

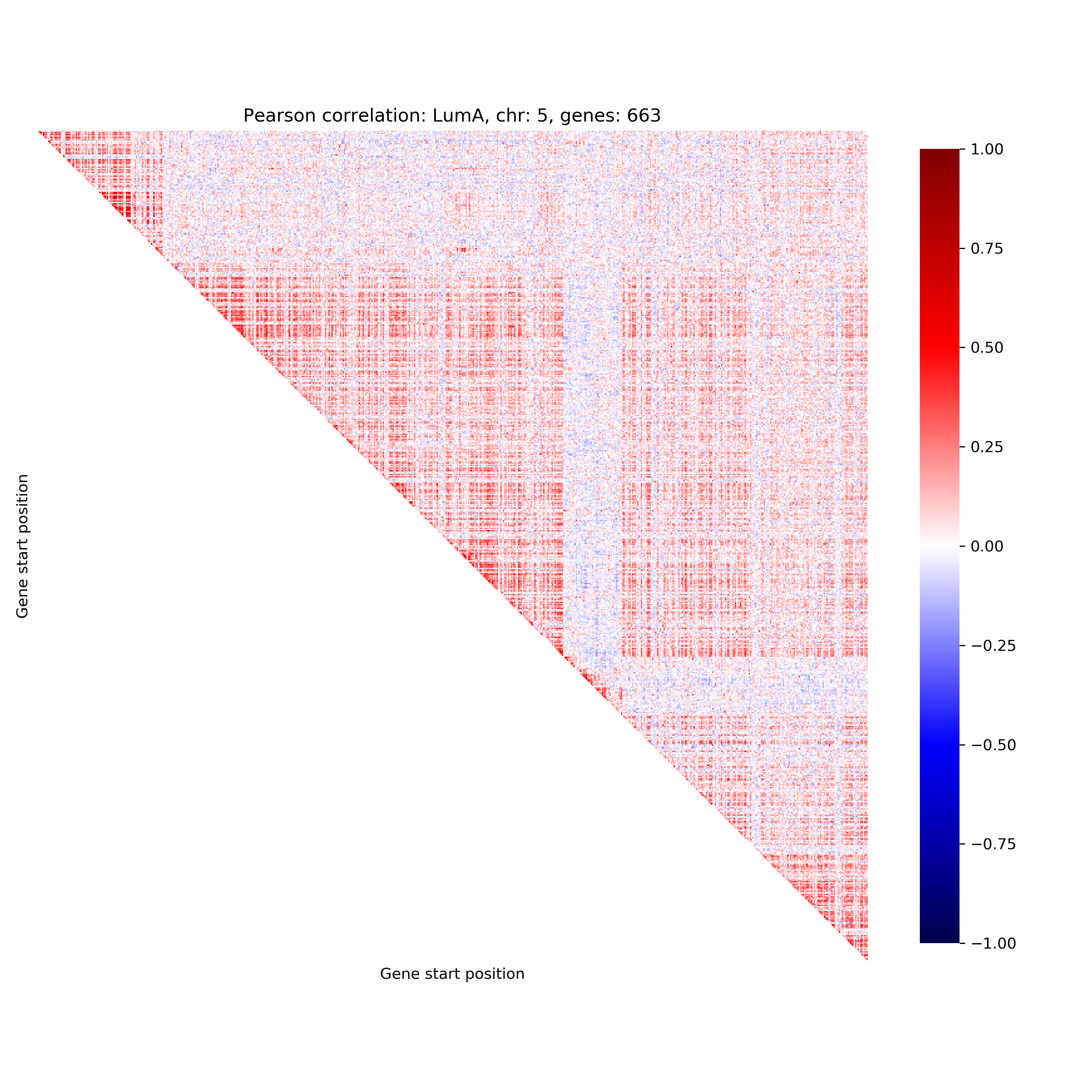

Supplement: Supplementary Material S2 — Heatmaps of Pearson correlation for each chromosome in the adjacent normal phenotype. The color code is the same than in Figure 1 . [file DataSheet_2.zip › SuppMat3/LumA-chr5.png]

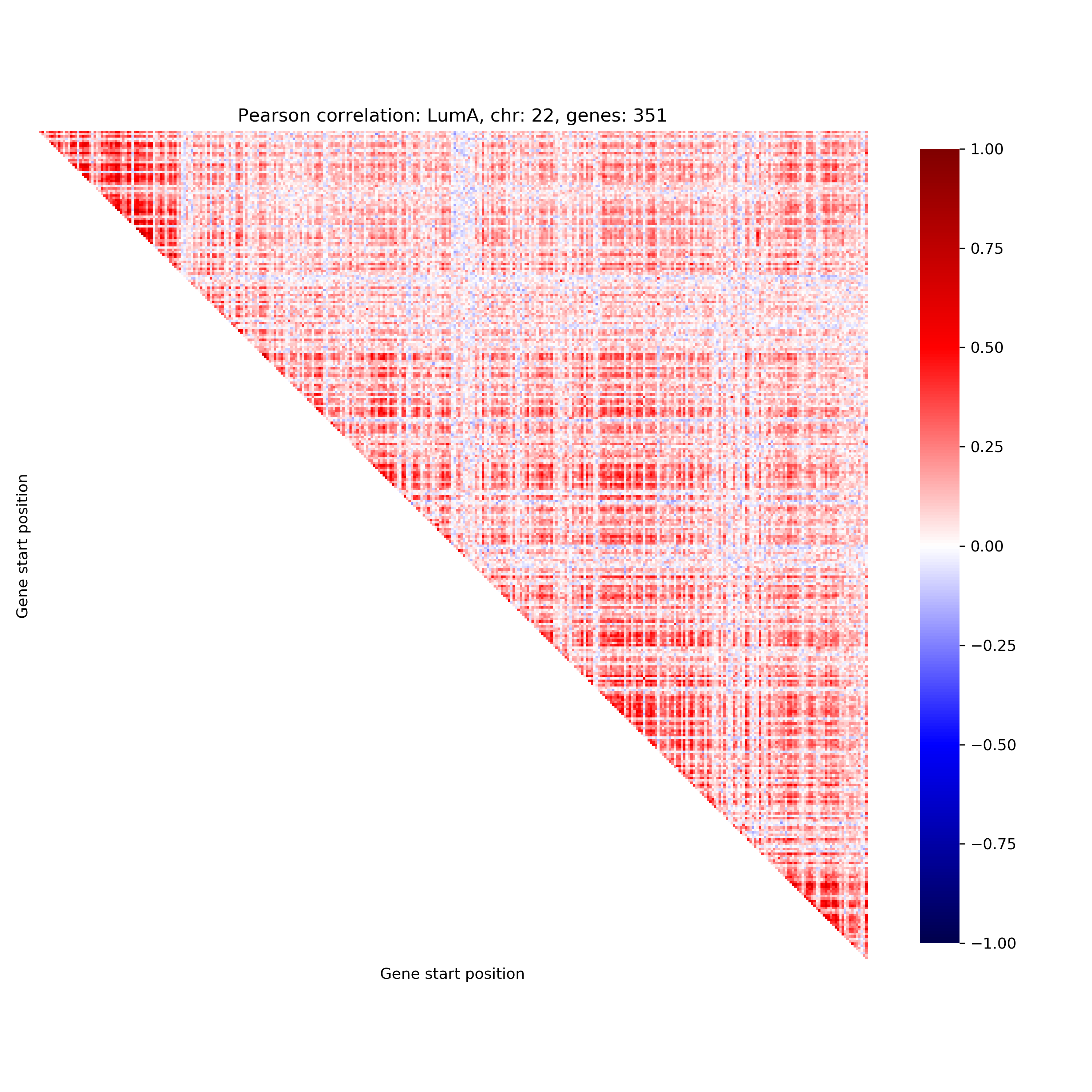

Supplement: Supplementary Material S2 — Heatmaps of Pearson correlation for each chromosome in the adjacent normal phenotype. The color code is the same than in Figure 1 . [file DataSheet_2.zip › SuppMat3/LumA-chr22.png]

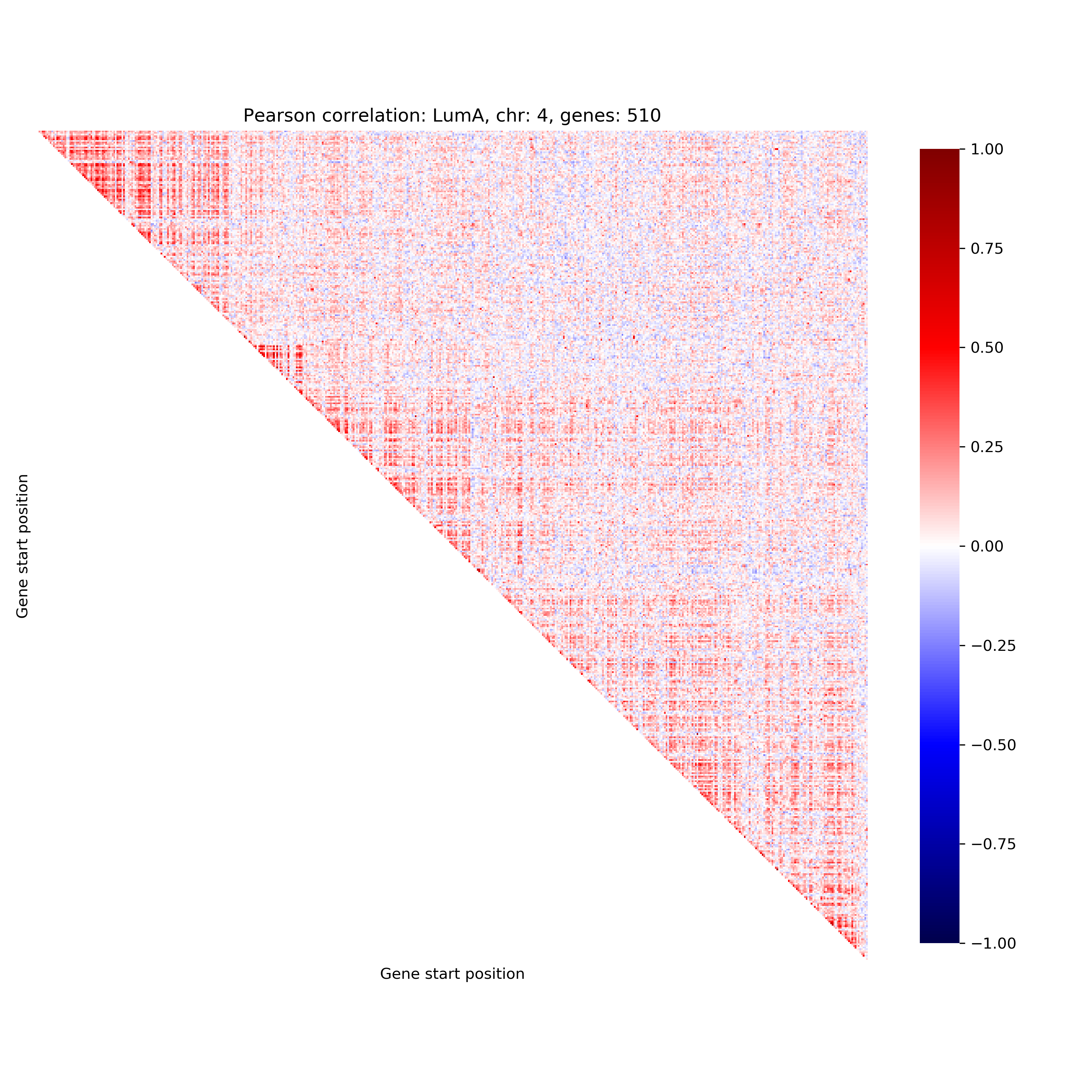

Supplement: Supplementary Material S2 — Heatmaps of Pearson correlation for each chromosome in the adjacent normal phenotype. The color code is the same than in Figure 1 . [file DataSheet_2.zip › SuppMat3/LumA-chr4.png]

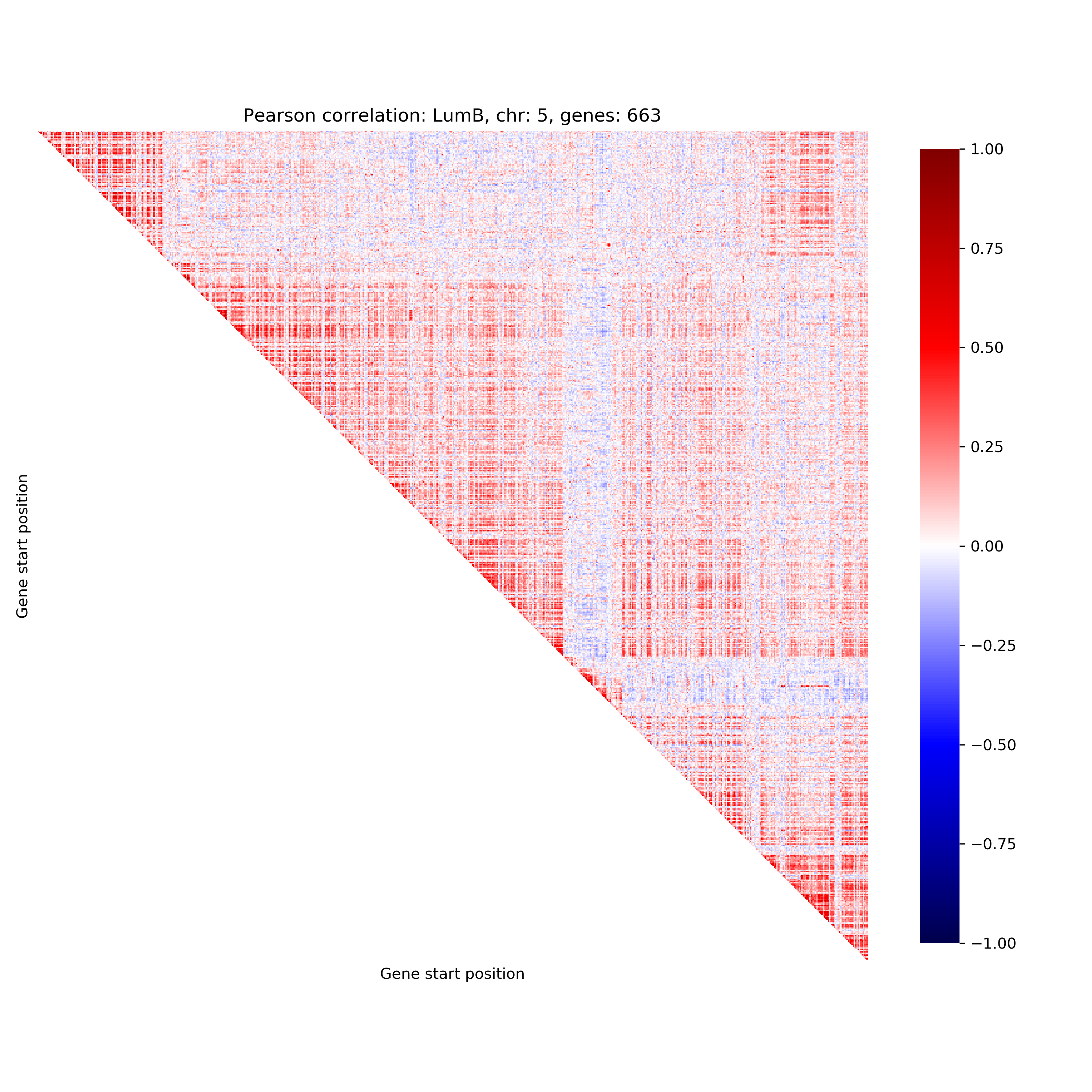

Supplement: Supplementary Material S3 — Heatmaps of Pearson correlation for each chromosome in the Luminal A phenotype. [file DataSheet_3.zip › SuppMat4/LumB-chr5.png]

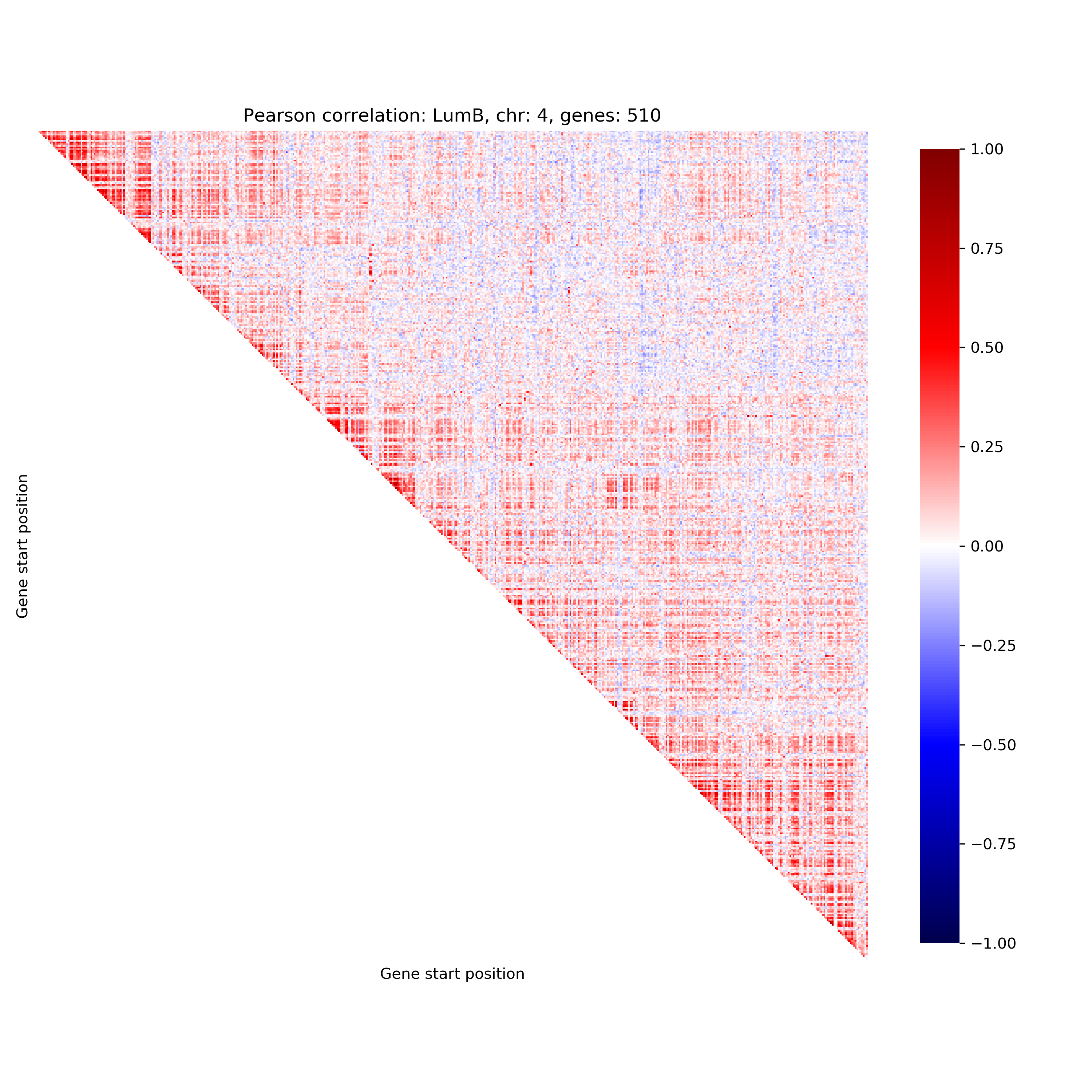

Supplement: Supplementary Material S3 — Heatmaps of Pearson correlation for each chromosome in the Luminal A phenotype. [file DataSheet_3.zip › SuppMat4/LumB-chr4.png]

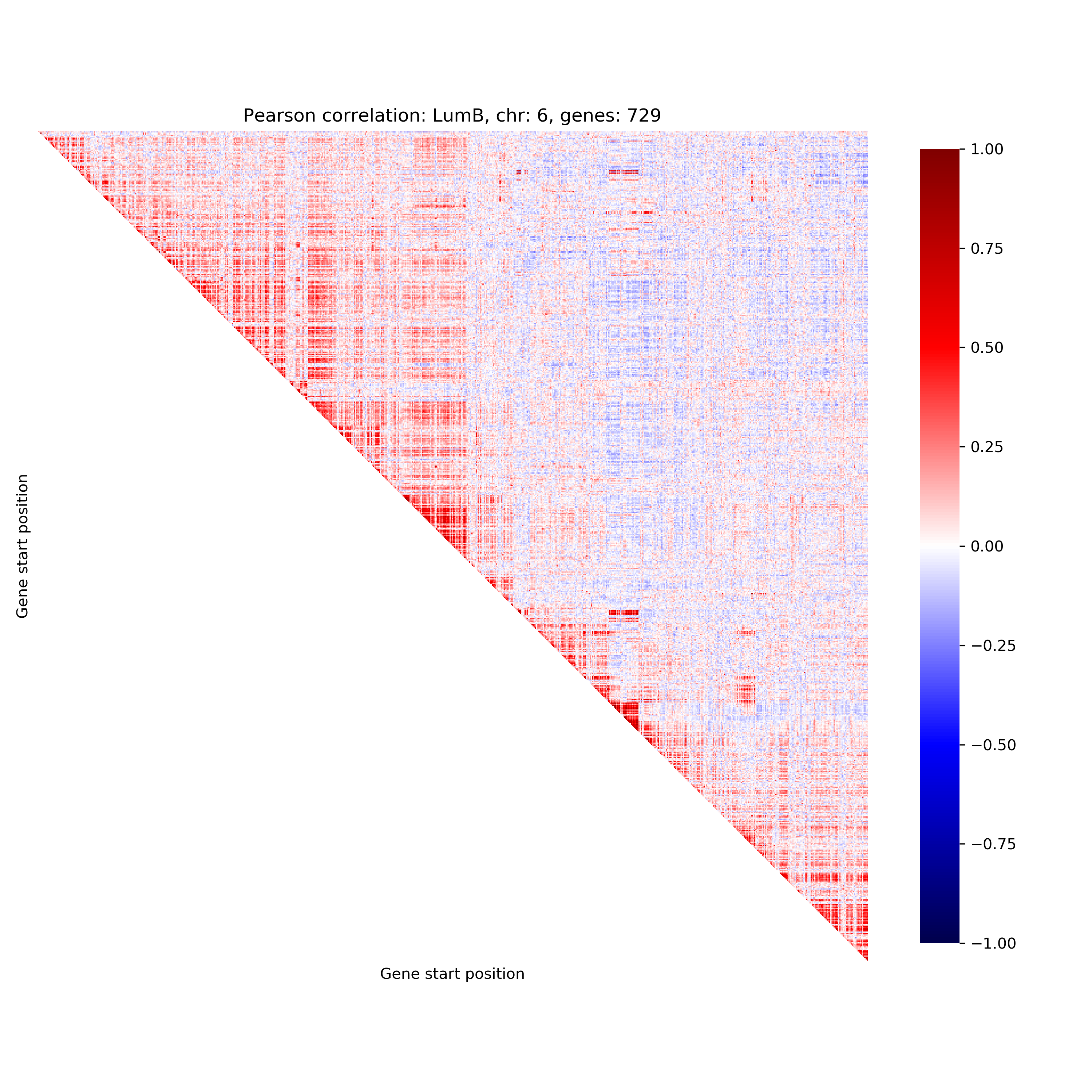

Supplement: Supplementary Material S3 — Heatmaps of Pearson correlation for each chromosome in the Luminal A phenotype. [file DataSheet_3.zip › SuppMat4/LumB-chr6.png]

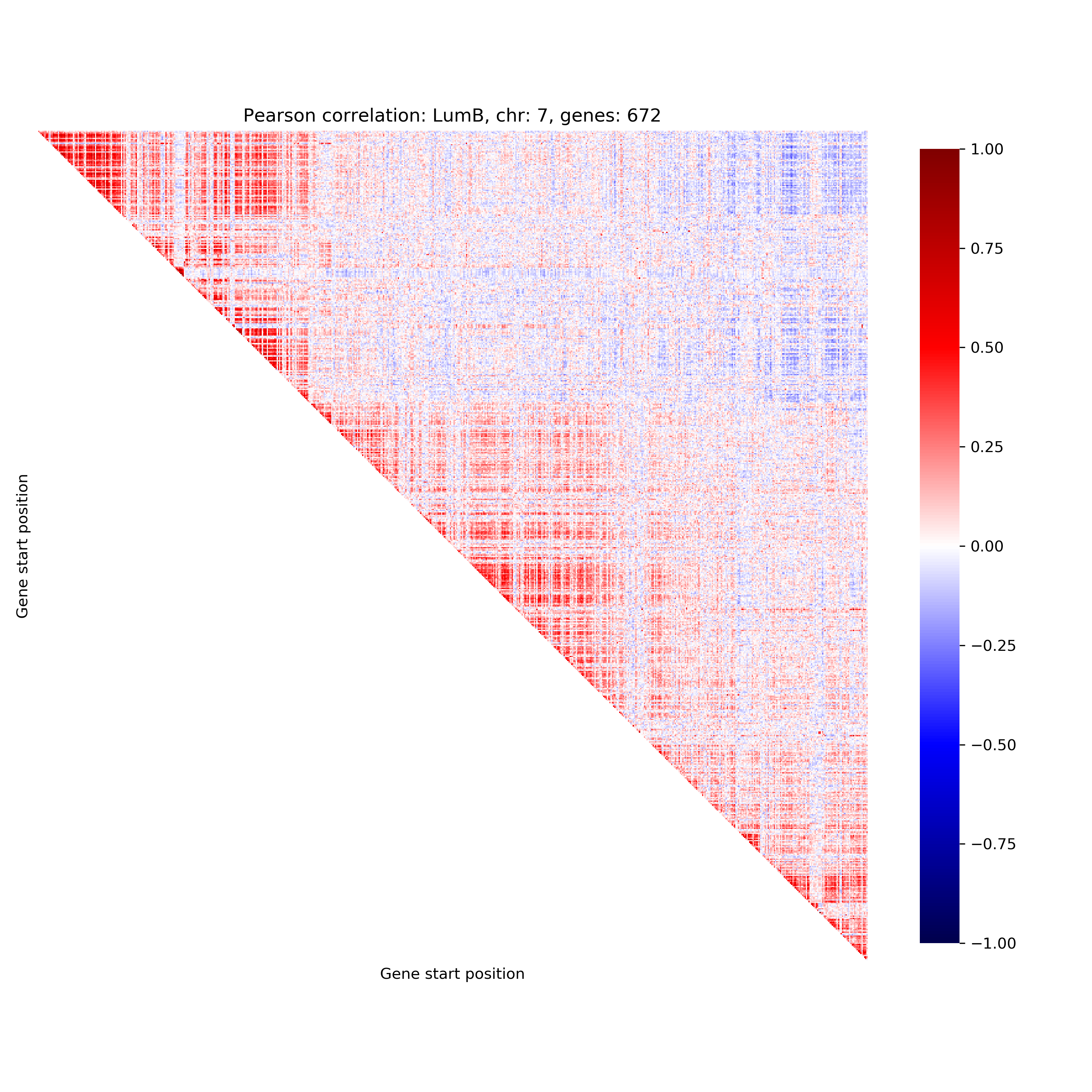

Supplement: Supplementary Material S3 — Heatmaps of Pearson correlation for each chromosome in the Luminal A phenotype. [file DataSheet_3.zip › SuppMat4/LumB-chr7.png]

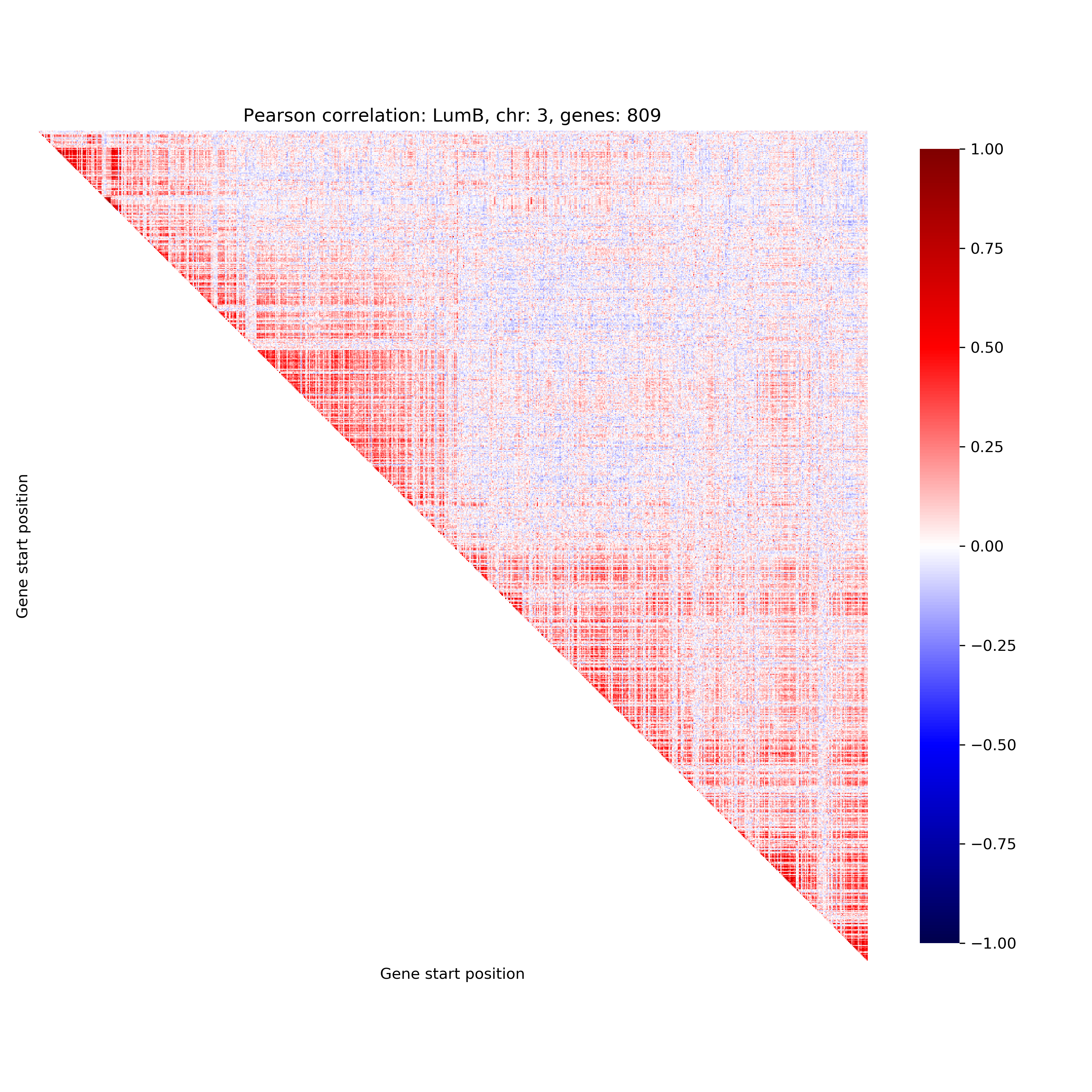

Supplement: Supplementary Material S3 — Heatmaps of Pearson correlation for each chromosome in the Luminal A phenotype. [file DataSheet_3.zip › SuppMat4/LumB-chr3.png]

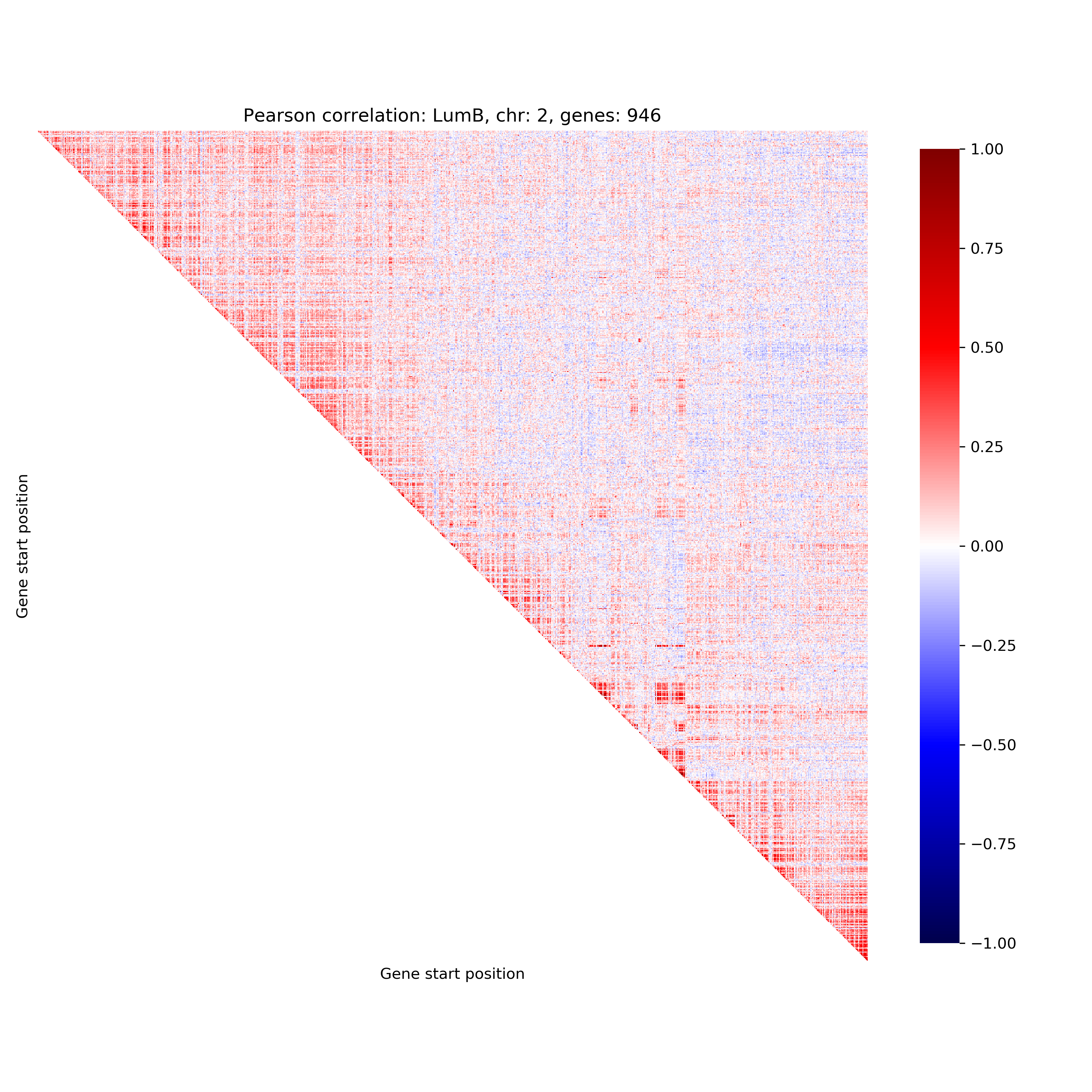

Supplement: Supplementary Material S3 — Heatmaps of Pearson correlation for each chromosome in the Luminal A phenotype. [file DataSheet_3.zip › SuppMat4/LumB-chr2.png]

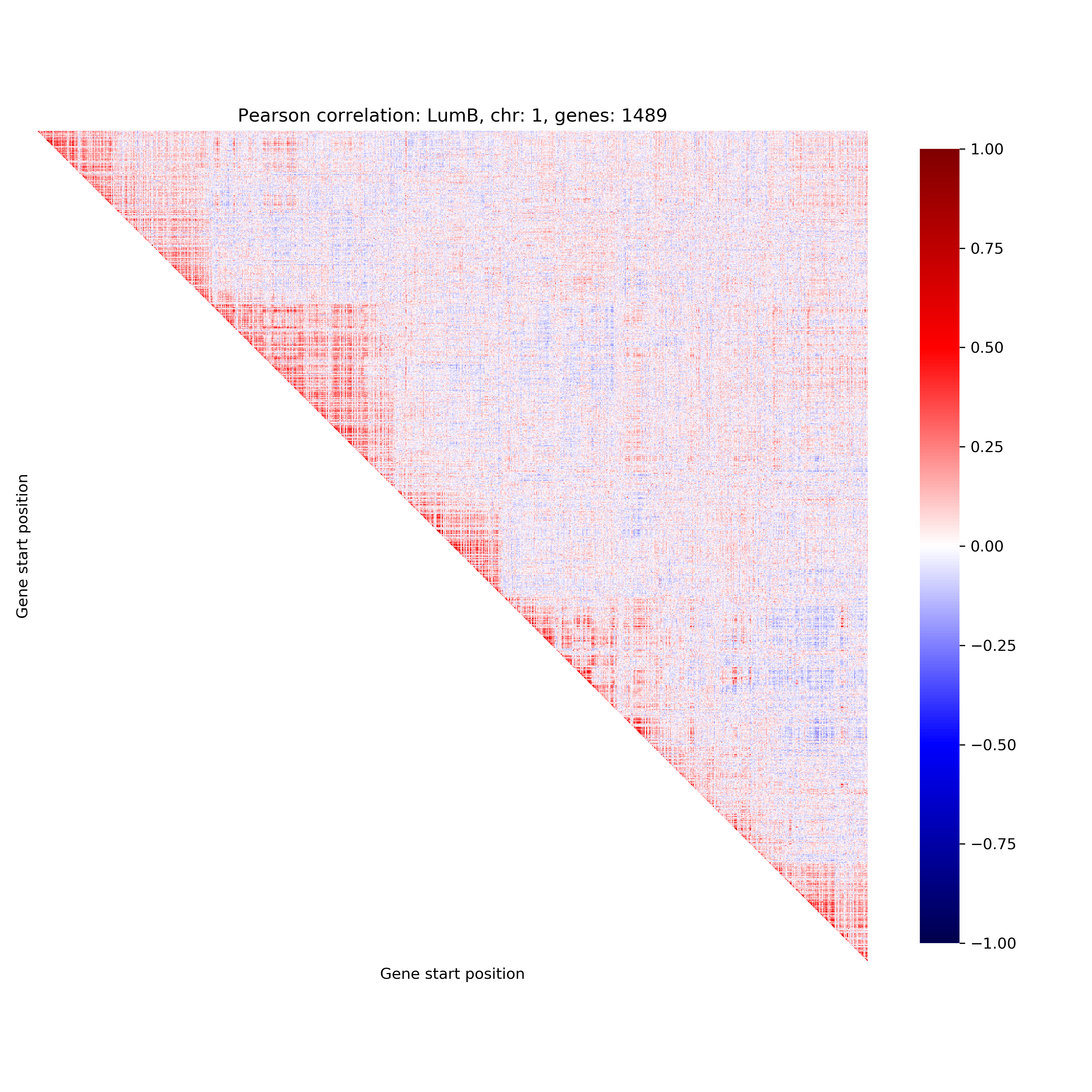

Supplement: Supplementary Material S3 — Heatmaps of Pearson correlation for each chromosome in the Luminal A phenotype. [file DataSheet_3.zip › SuppMat4/LumB-chr1.png]

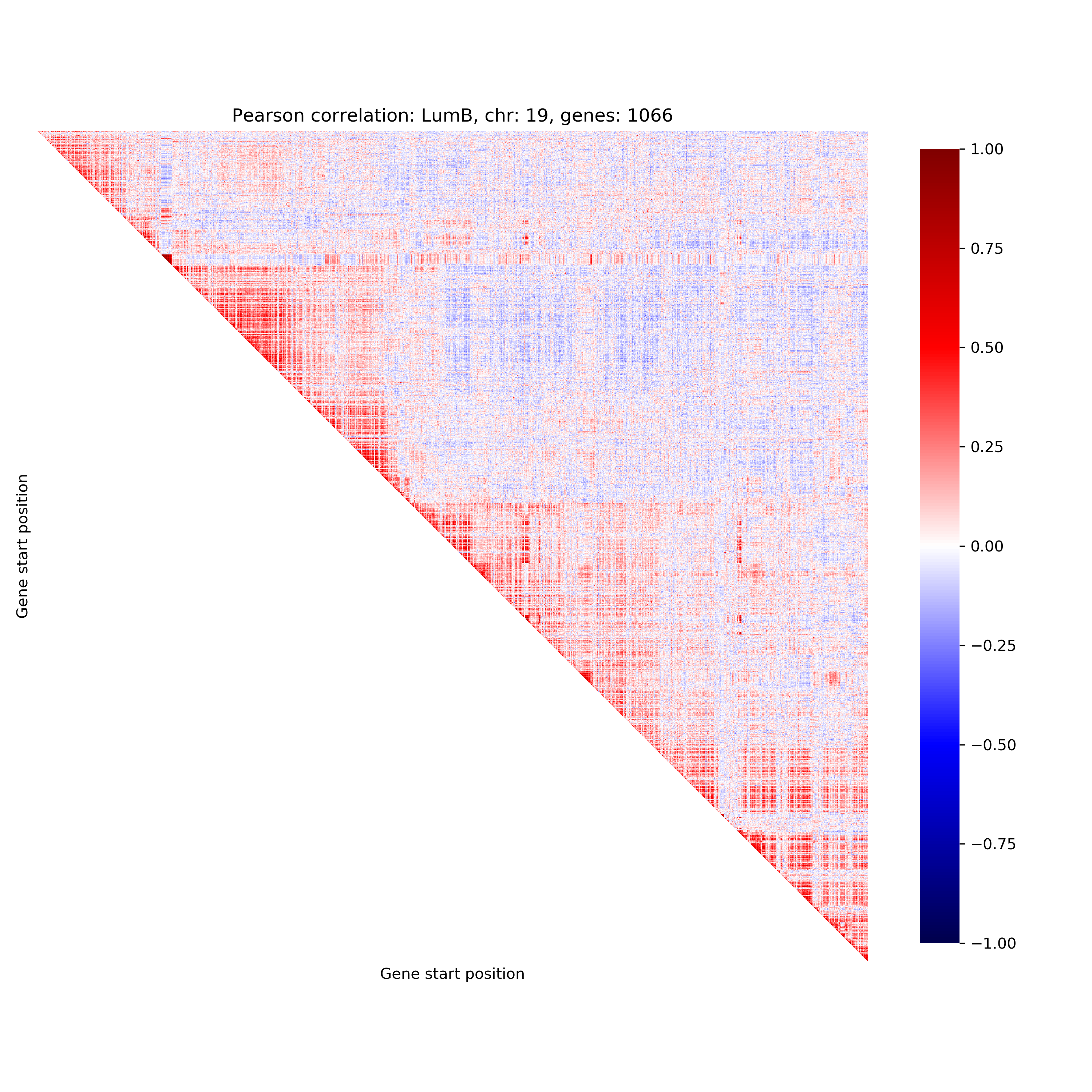

Supplement: Supplementary Material S3 — Heatmaps of Pearson correlation for each chromosome in the Luminal A phenotype. [file DataSheet_3.zip › SuppMat4/LumB-chr19.png]

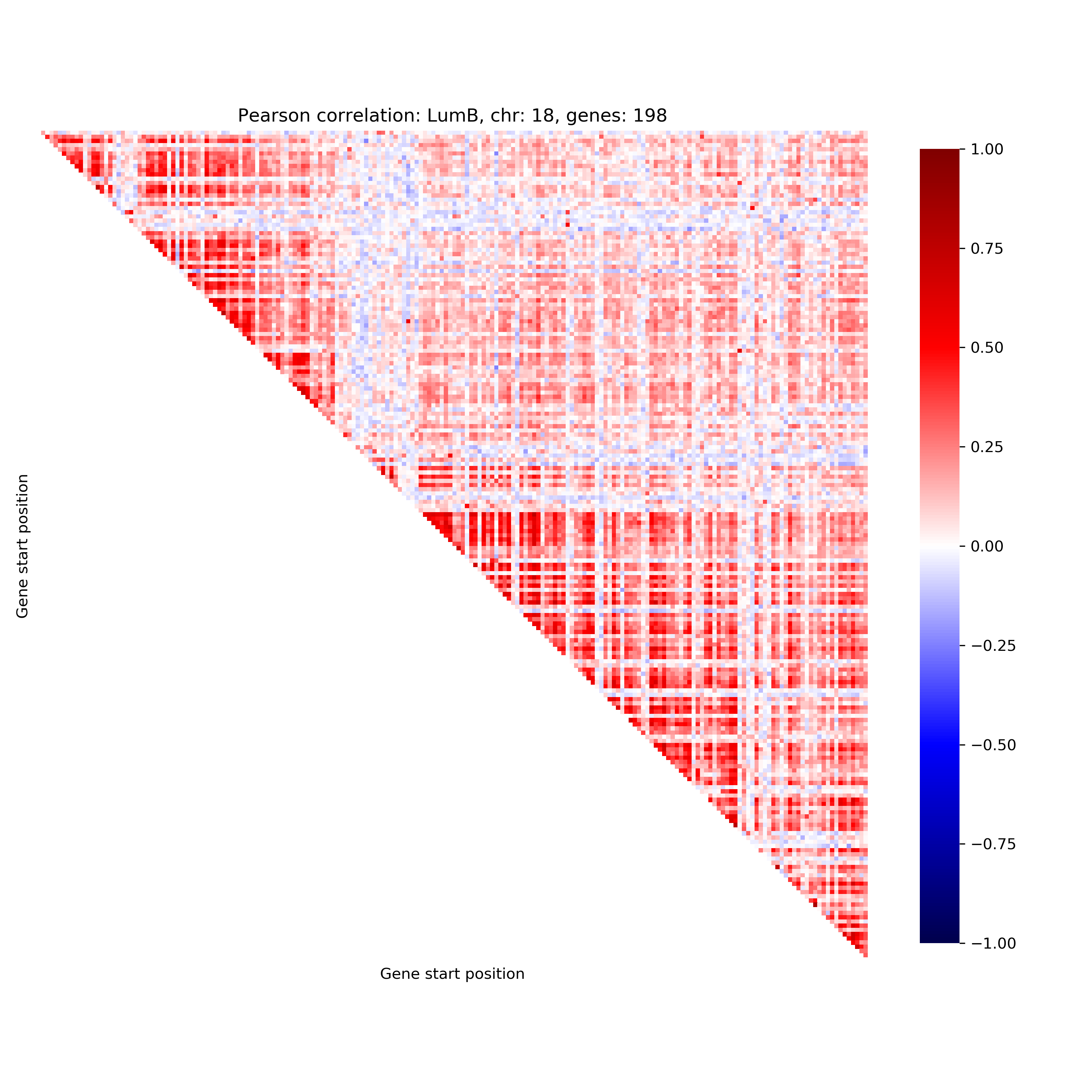

Supplement: Supplementary Material S3 — Heatmaps of Pearson correlation for each chromosome in the Luminal A phenotype. [file DataSheet_3.zip › SuppMat4/LumB-chr18.png]

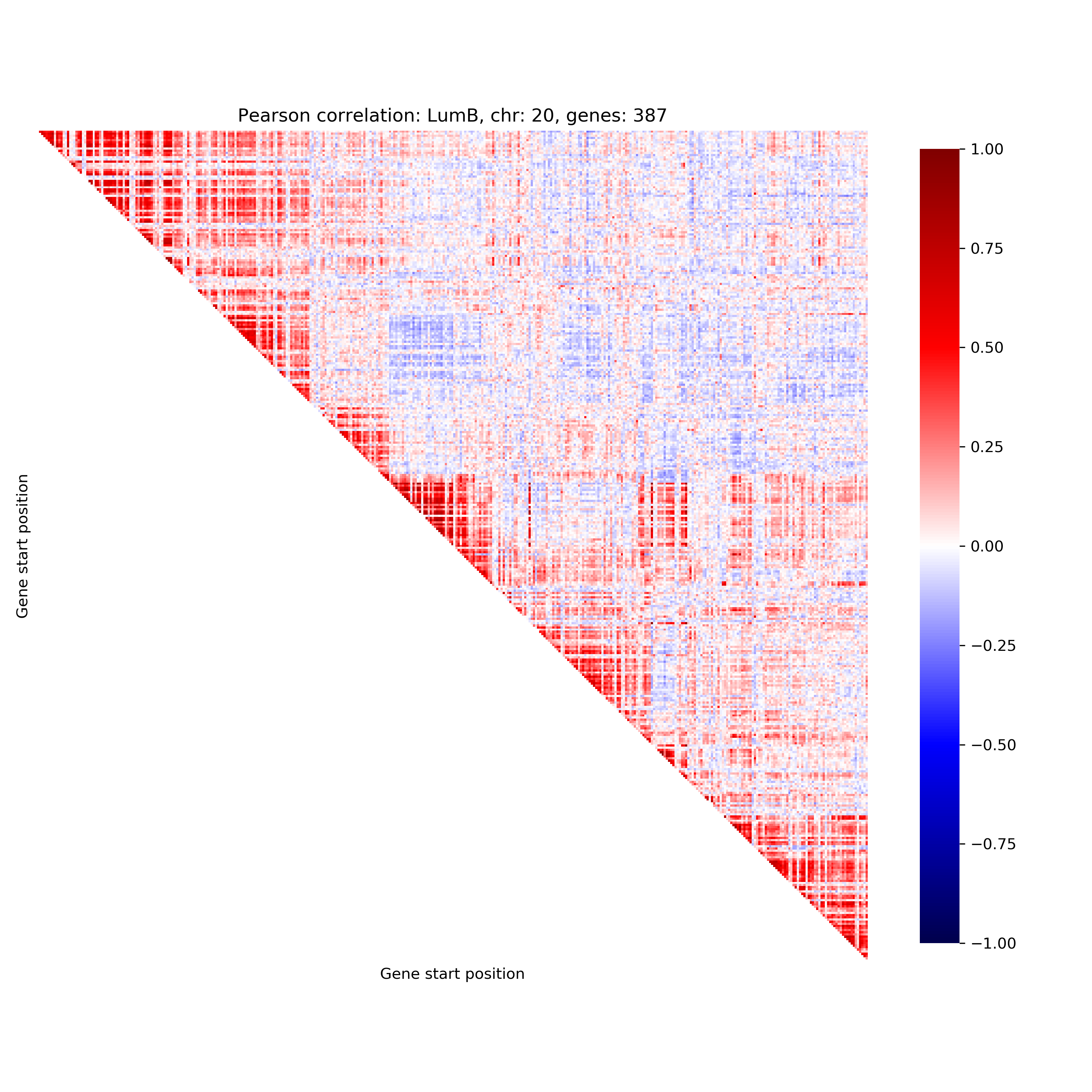

Supplement: Supplementary Material S3 — Heatmaps of Pearson correlation for each chromosome in the Luminal A phenotype. [file DataSheet_3.zip › SuppMat4/LumB-chr20.png]

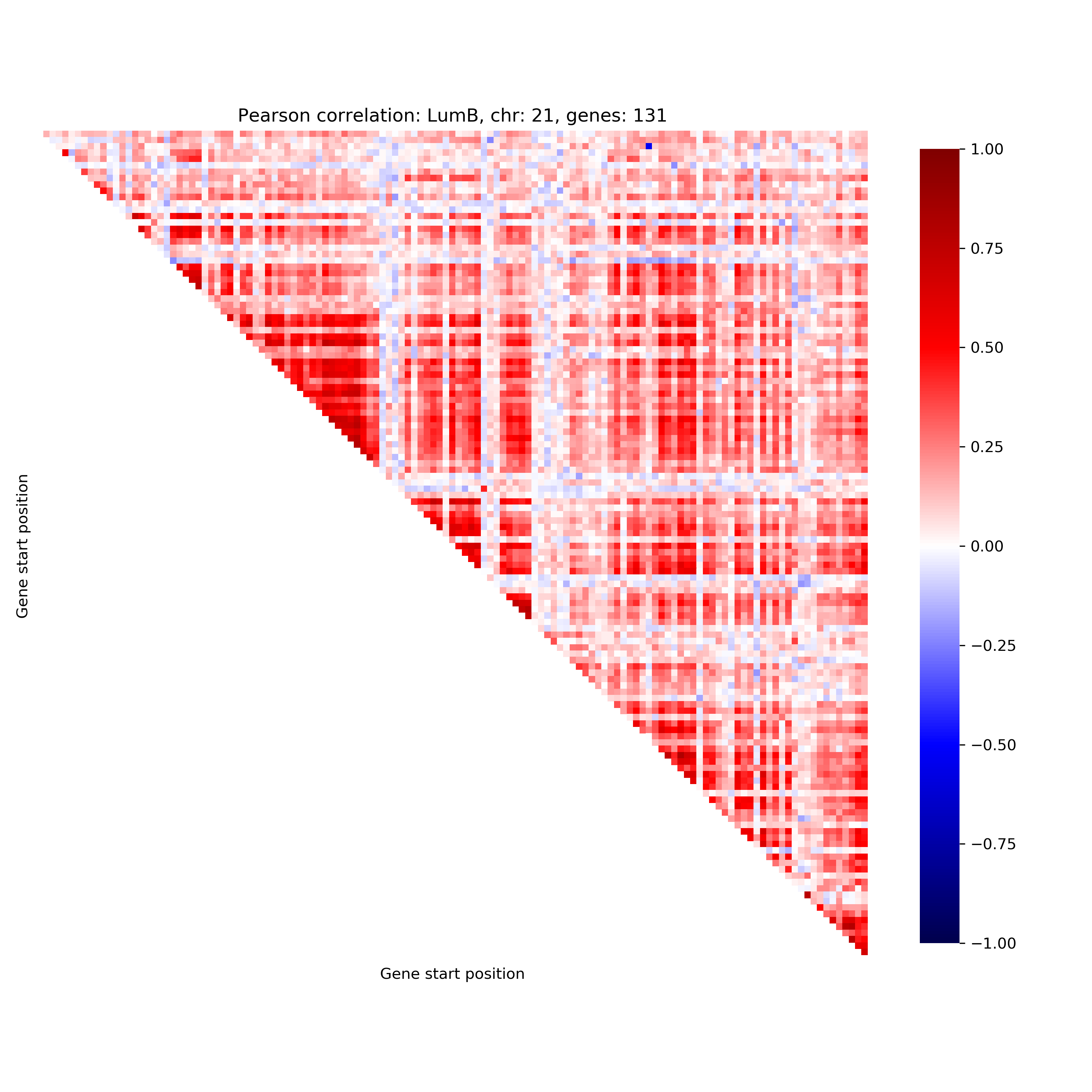

Supplement: Supplementary Material S3 — Heatmaps of Pearson correlation for each chromosome in the Luminal A phenotype. [file DataSheet_3.zip › SuppMat4/LumB-chr21.png]

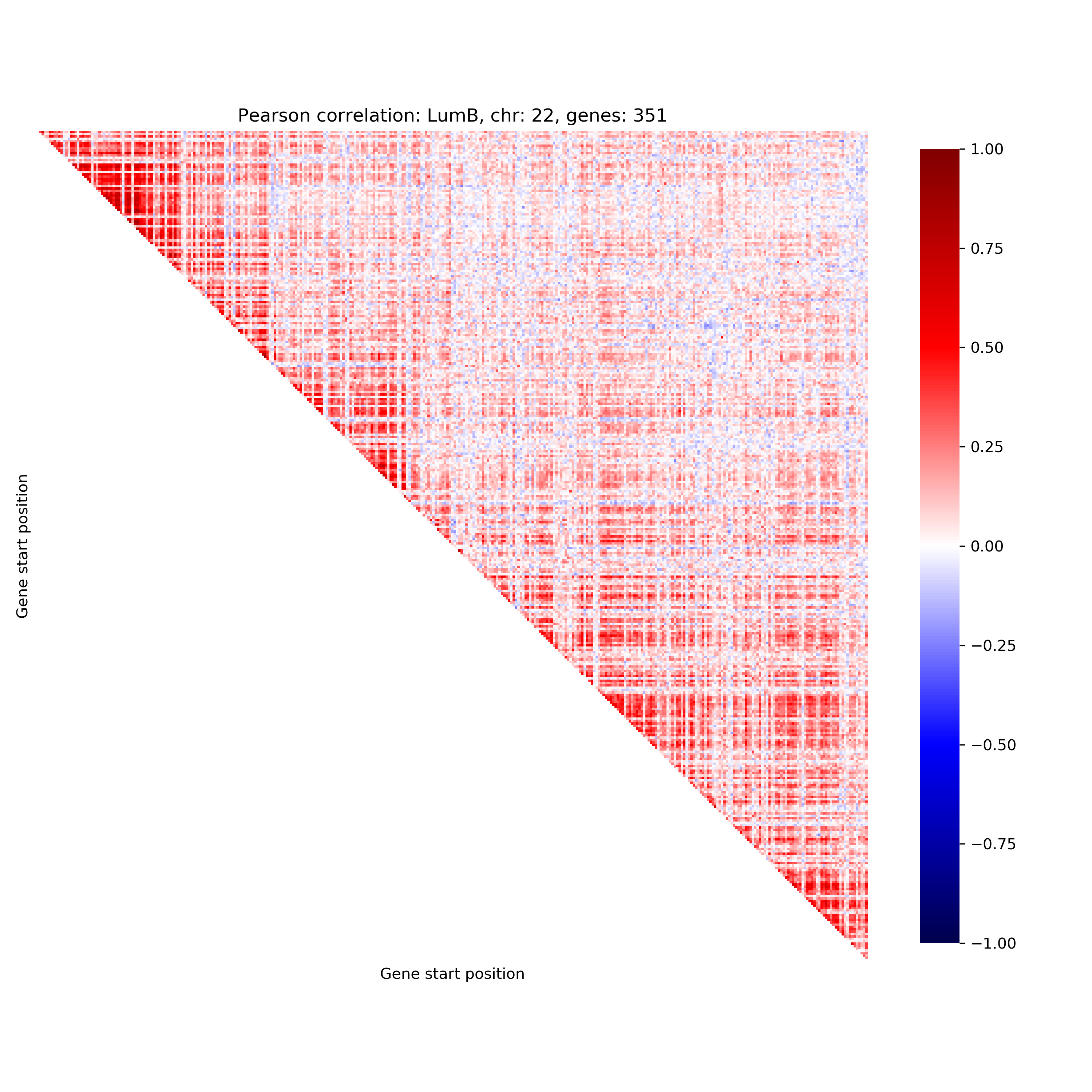

Supplement: Supplementary Material S3 — Heatmaps of Pearson correlation for each chromosome in the Luminal A phenotype. [file DataSheet_3.zip › SuppMat4/LumB-chr22.png]

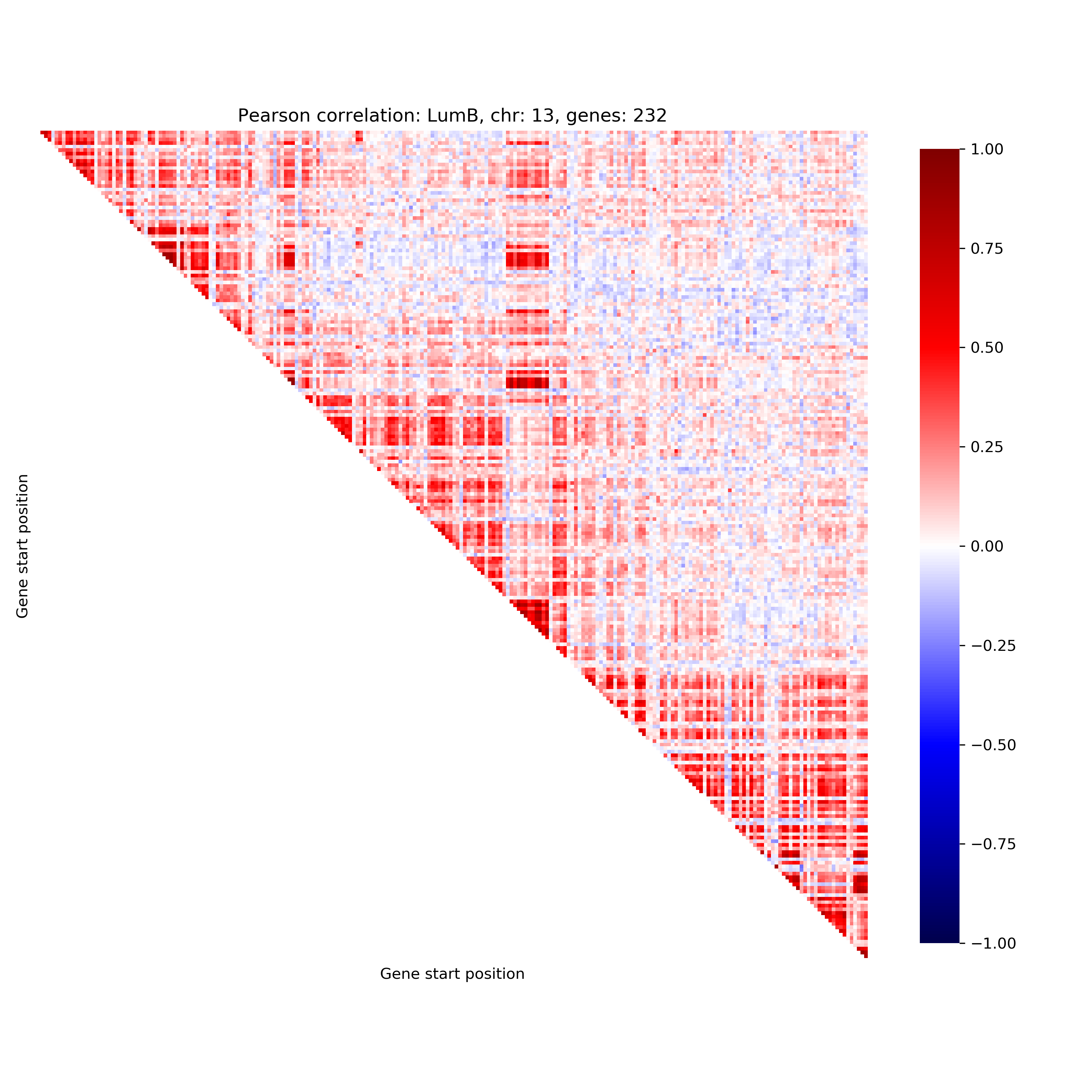

Supplement: Supplementary Material S3 — Heatmaps of Pearson correlation for each chromosome in the Luminal A phenotype. [file DataSheet_3.zip › SuppMat4/LumB-chr13.png]

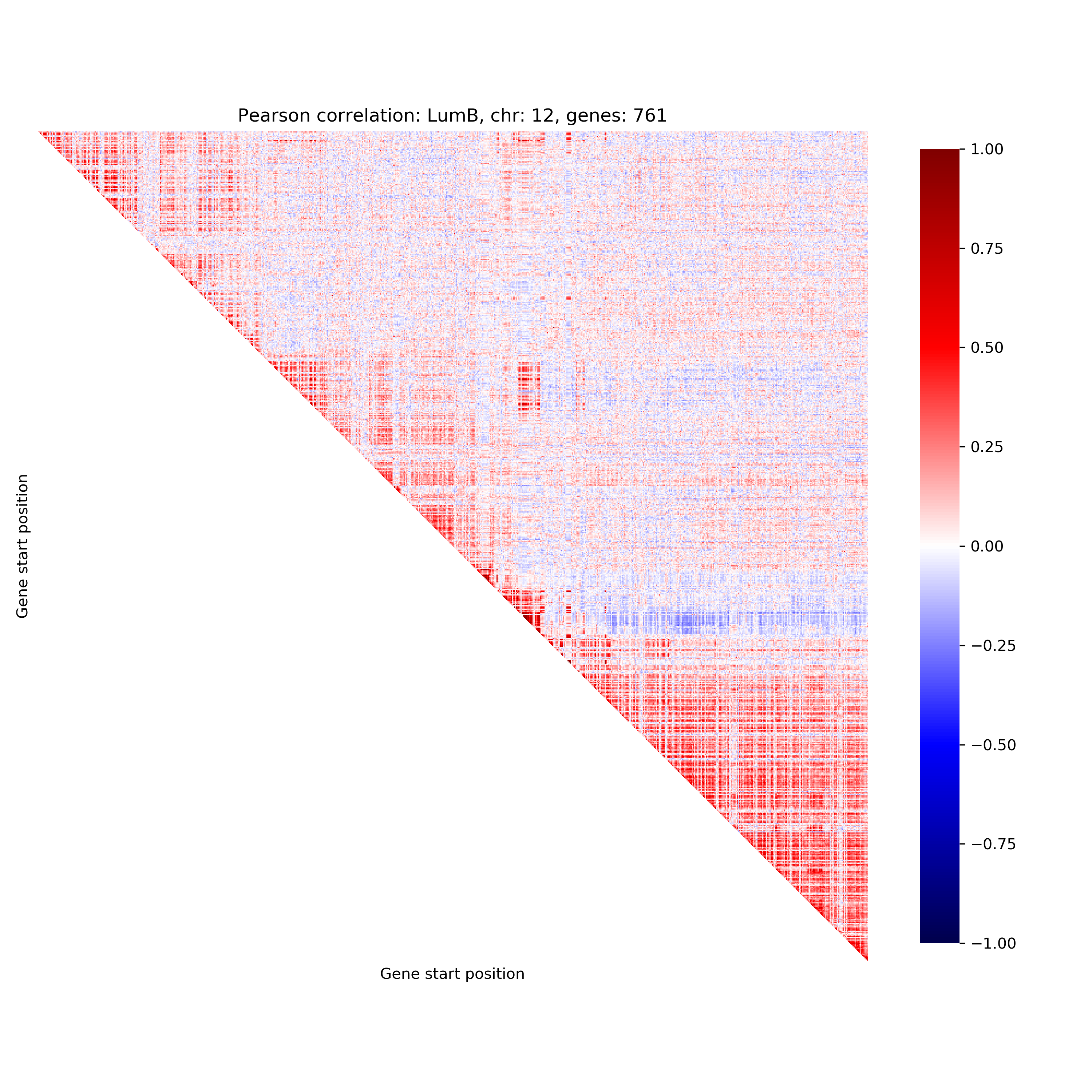

Supplement: Supplementary Material S3 — Heatmaps of Pearson correlation for each chromosome in the Luminal A phenotype. [file DataSheet_3.zip › SuppMat4/LumB-chr12.png]

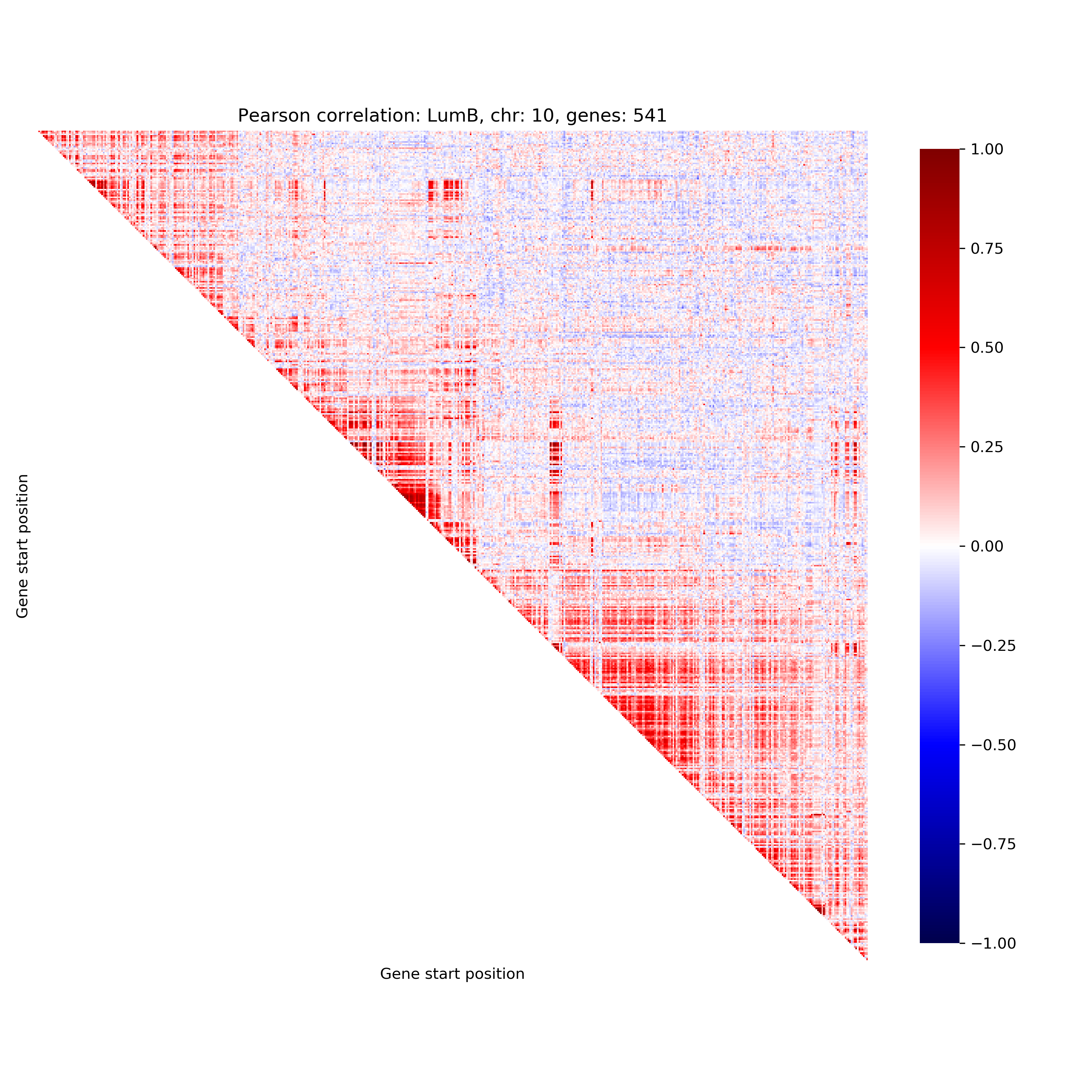

Supplement: Supplementary Material S3 — Heatmaps of Pearson correlation for each chromosome in the Luminal A phenotype. [file DataSheet_3.zip › SuppMat4/LumB-chr10.png]

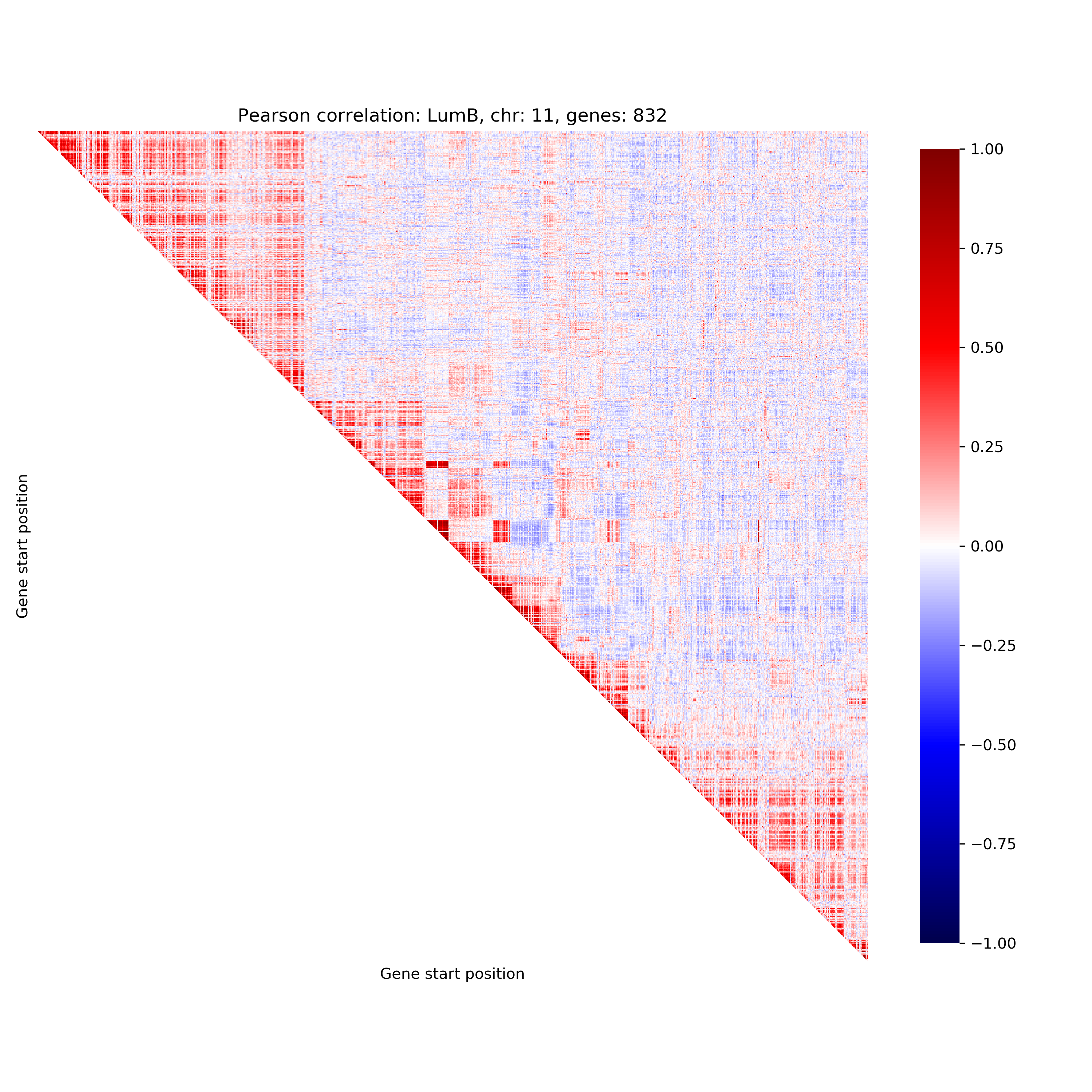

Supplement: Supplementary Material S3 — Heatmaps of Pearson correlation for each chromosome in the Luminal A phenotype. [file DataSheet_3.zip › SuppMat4/LumB-chr11.png]

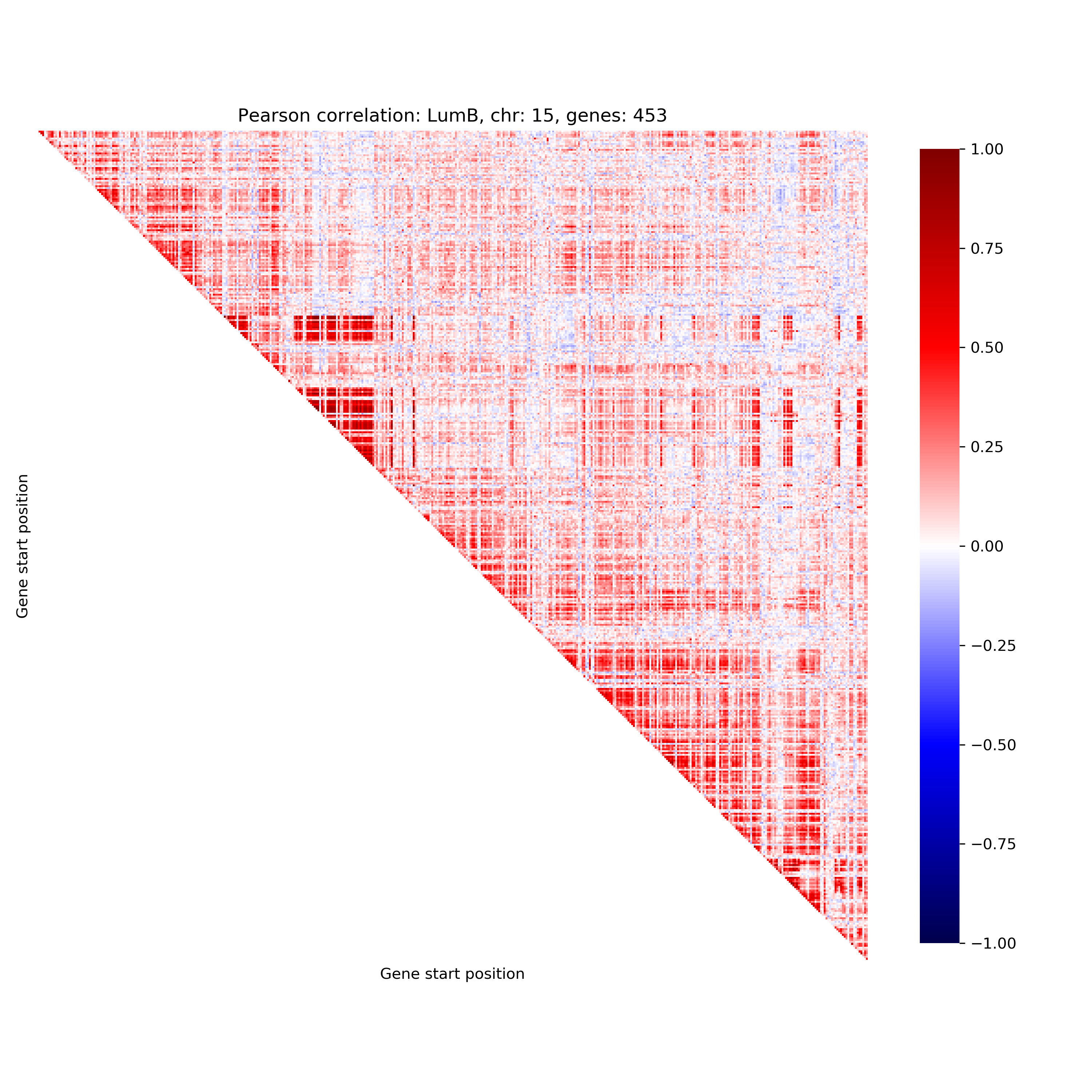

Supplement: Supplementary Material S3 — Heatmaps of Pearson correlation for each chromosome in the Luminal A phenotype. [file DataSheet_3.zip › SuppMat4/LumB-chr15.png]

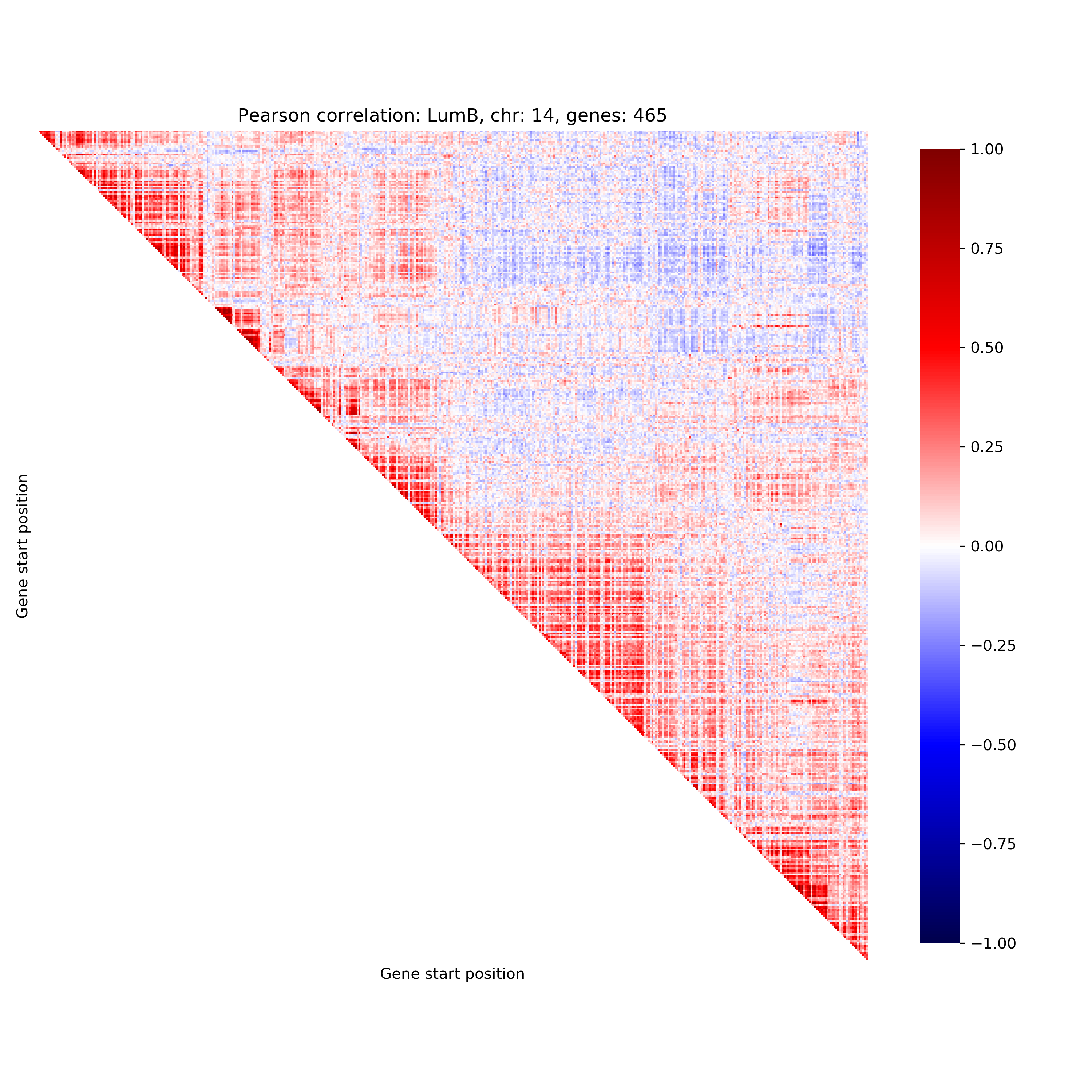

Supplement: Supplementary Material S3 — Heatmaps of Pearson correlation for each chromosome in the Luminal A phenotype. [file DataSheet_3.zip › SuppMat4/LumB-chr14.png]

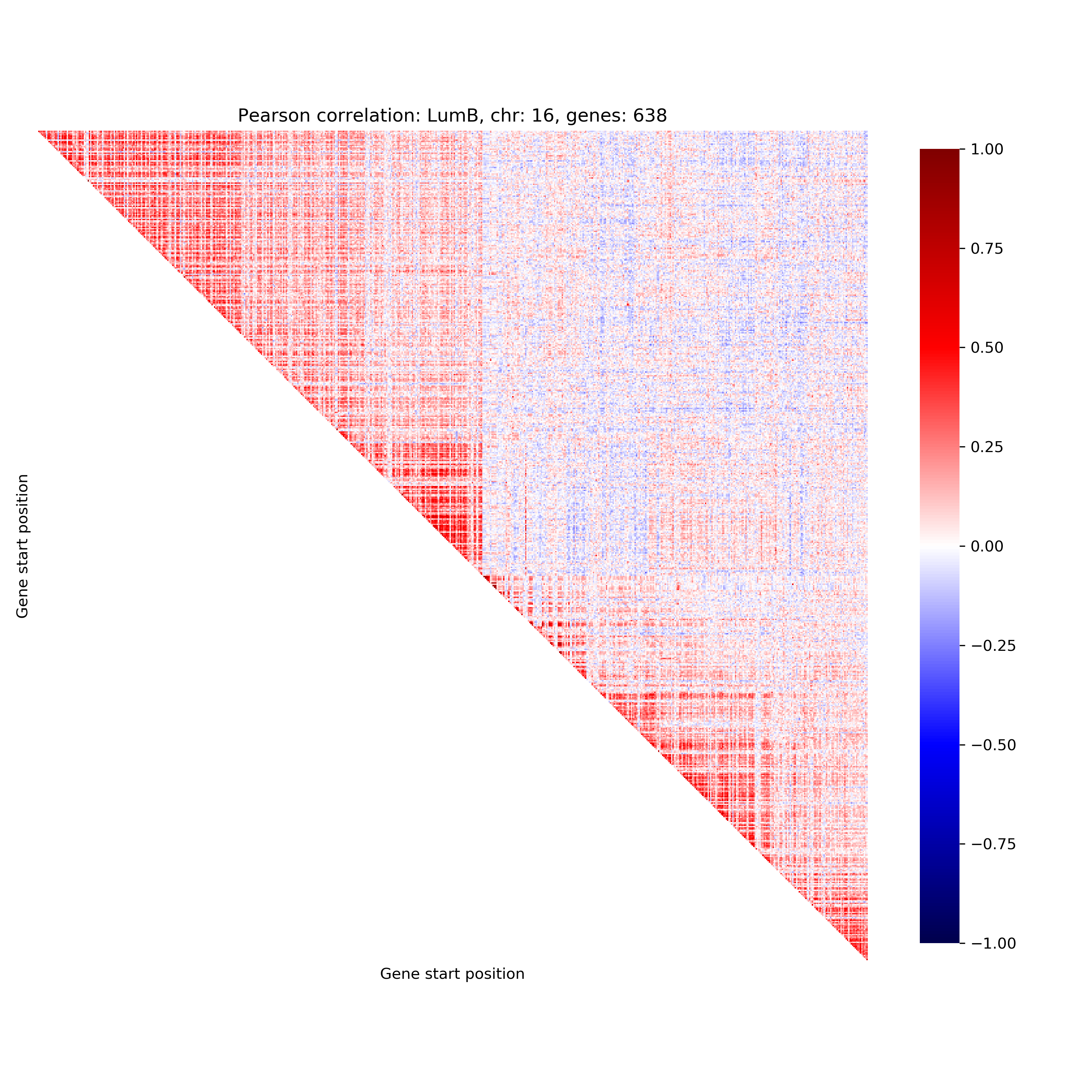

Supplement: Supplementary Material S3 — Heatmaps of Pearson correlation for each chromosome in the Luminal A phenotype. [file DataSheet_3.zip › SuppMat4/LumB-chr16.png]

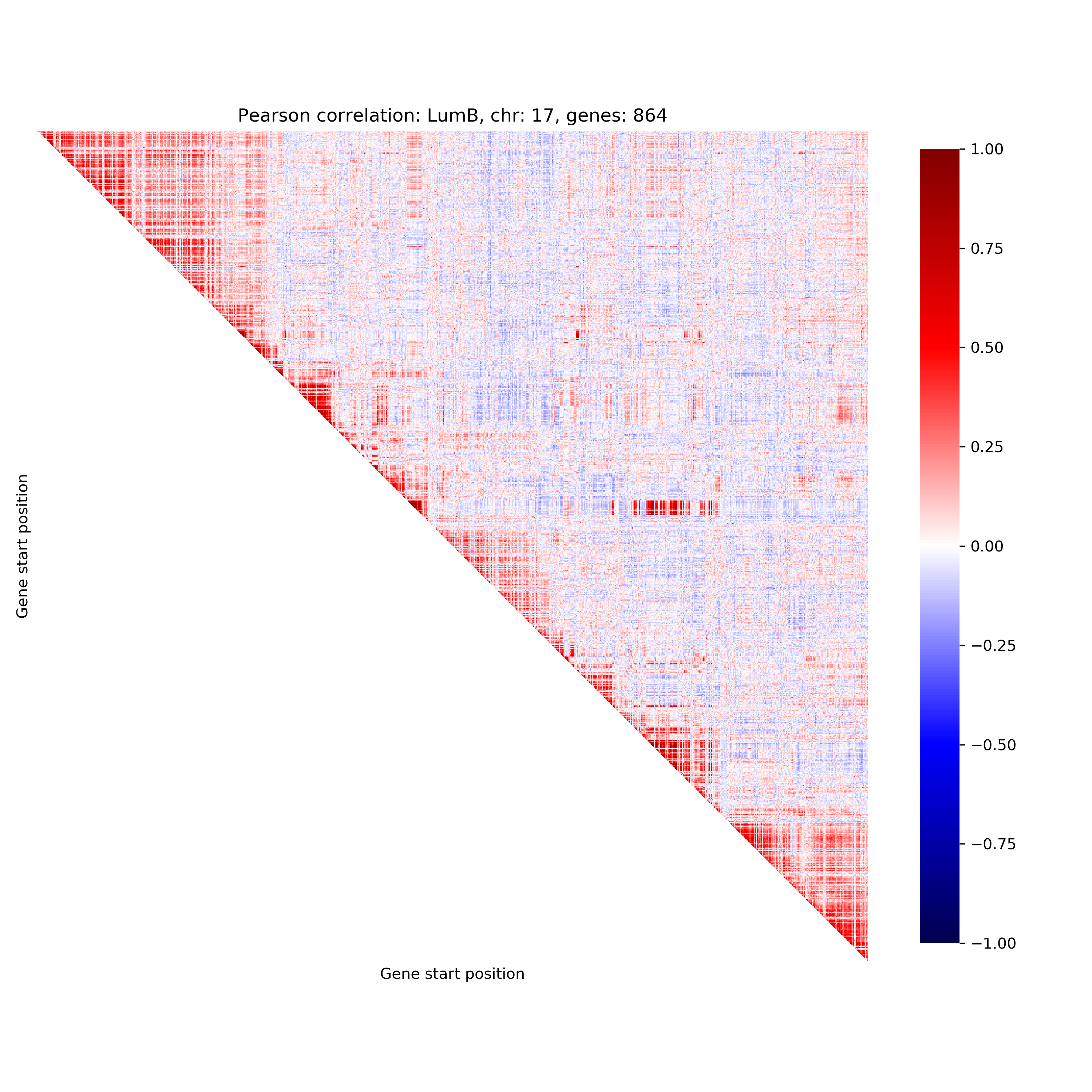

Supplement: Supplementary Material S3 — Heatmaps of Pearson correlation for each chromosome in the Luminal A phenotype. [file DataSheet_3.zip › SuppMat4/LumB-chr17.png]

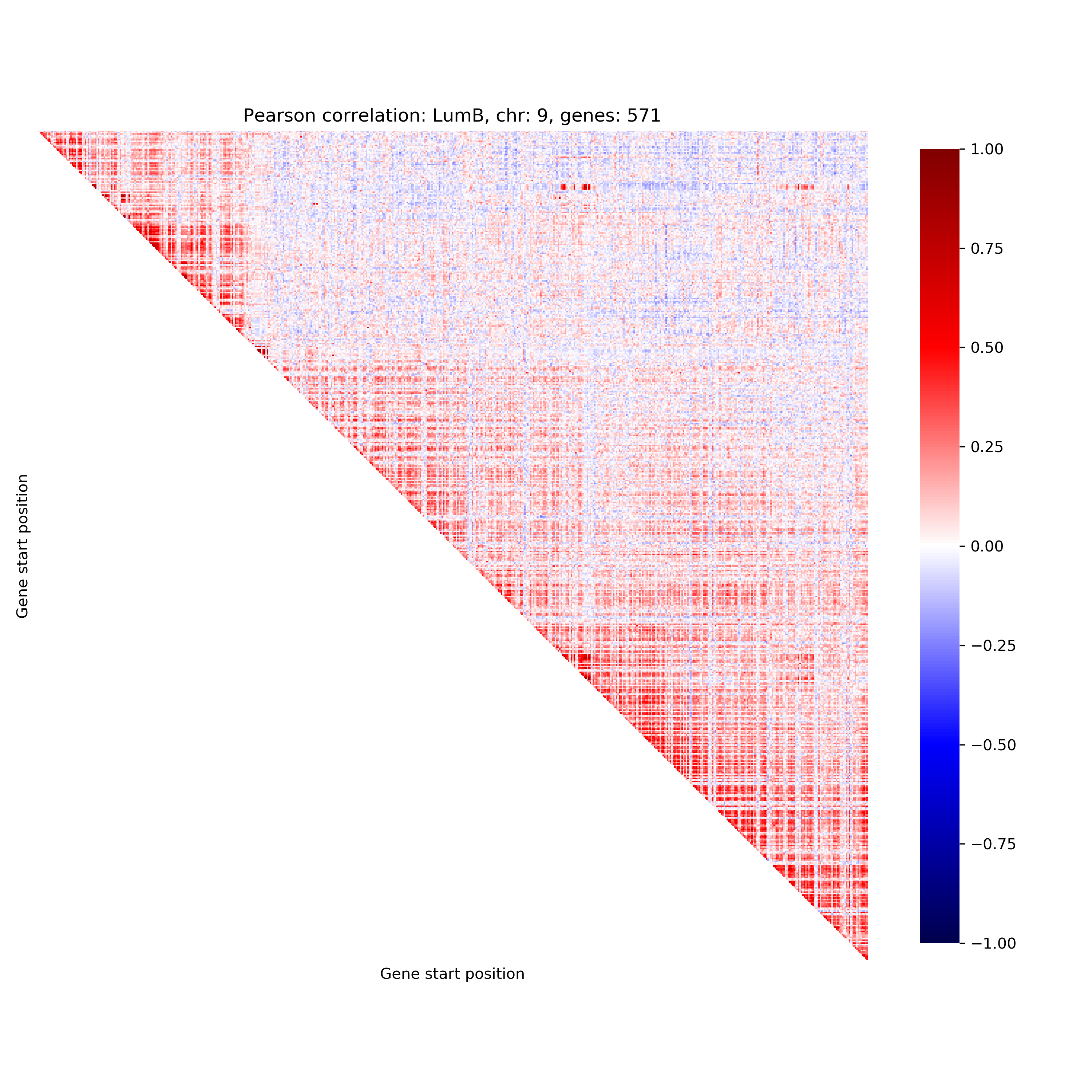

Supplement: Supplementary Material S3 — Heatmaps of Pearson correlation for each chromosome in the Luminal A phenotype. [file DataSheet_3.zip › SuppMat4/LumB-chr9.png]

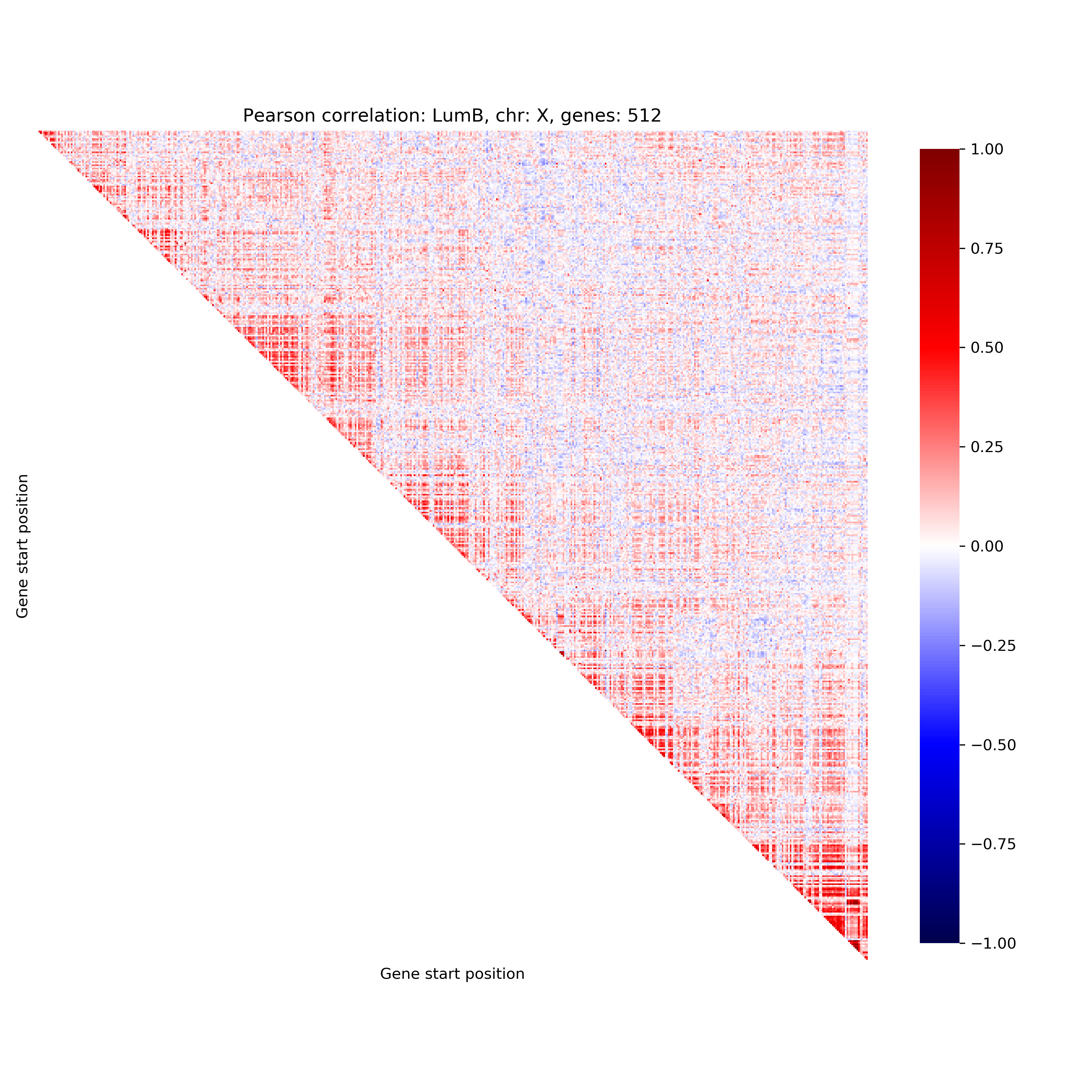

Supplement: Supplementary Material S3 — Heatmaps of Pearson correlation for each chromosome in the Luminal A phenotype. [file DataSheet_3.zip › SuppMat4/LumB-chrX.png]

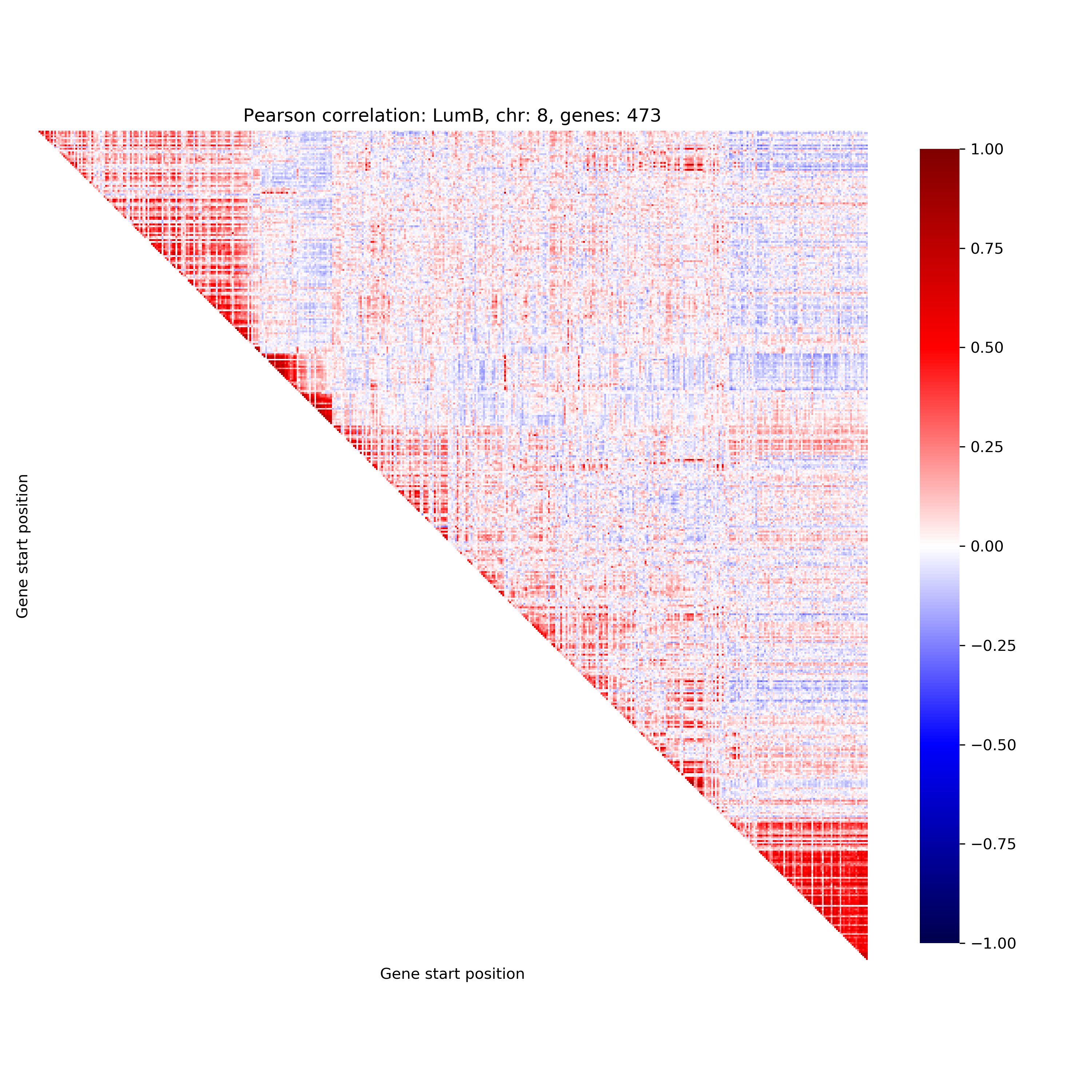

Supplement: Supplementary Material S3 — Heatmaps of Pearson correlation for each chromosome in the Luminal A phenotype. [file DataSheet_3.zip › SuppMat4/LumB-chr8.png]

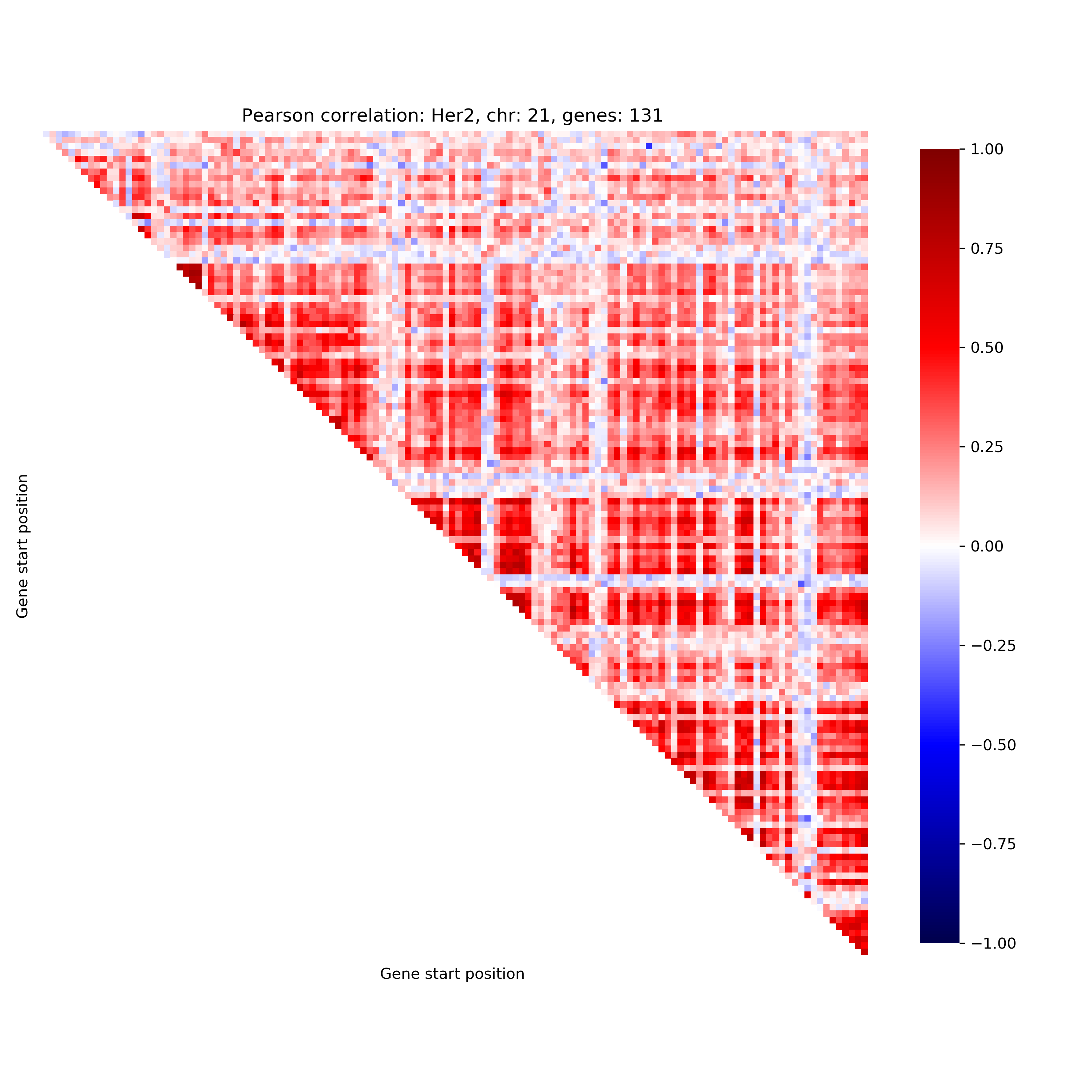

Supplement: Supplementary Material S4 — Heatmaps of Pearson correlation for each chromosome in the Luminal B phenotype. [file DataSheet_4.zip › SuppMat5/Her2-chr21.png]

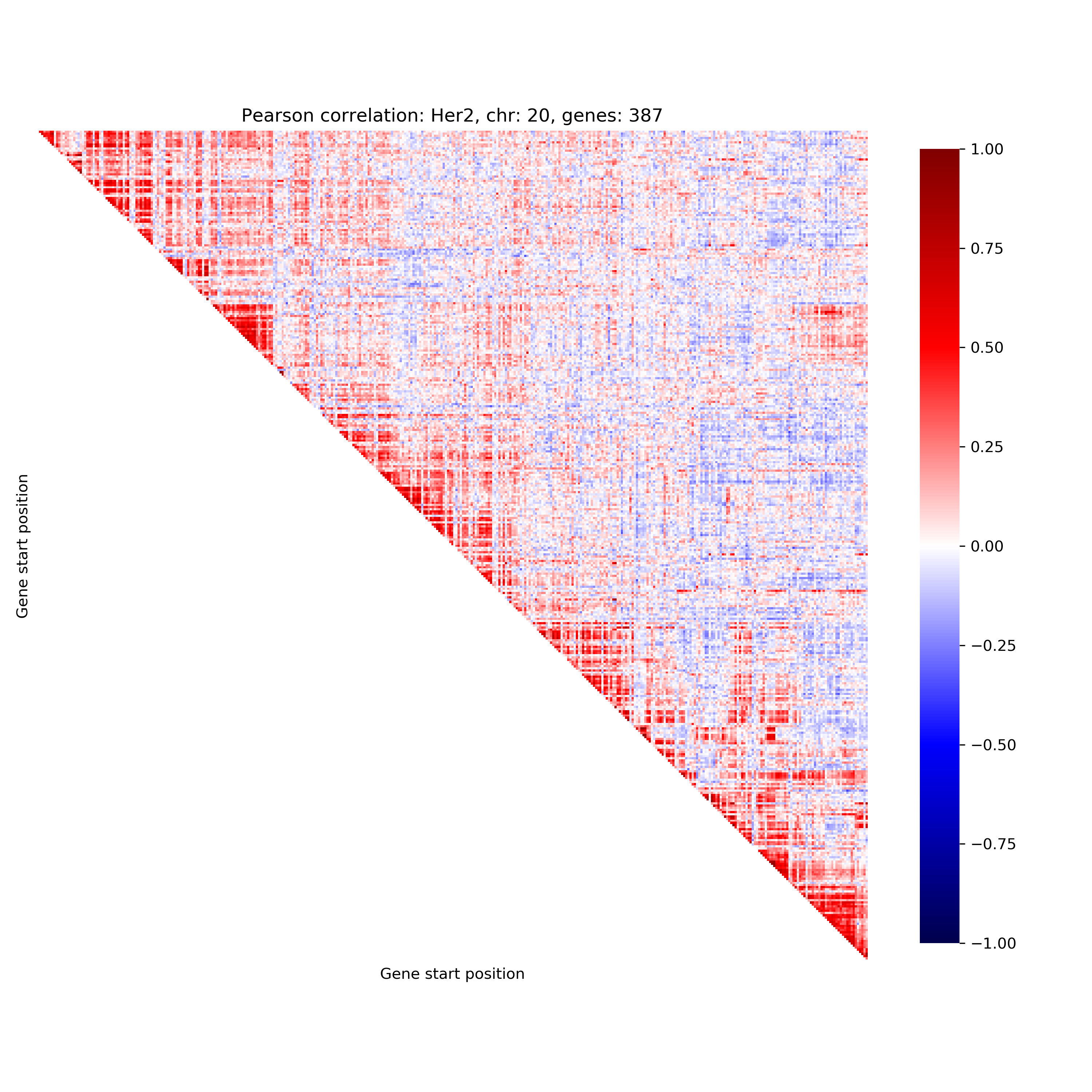

Supplement: Supplementary Material S4 — Heatmaps of Pearson correlation for each chromosome in the Luminal B phenotype. [file DataSheet_4.zip › SuppMat5/Her2-chr20.png]

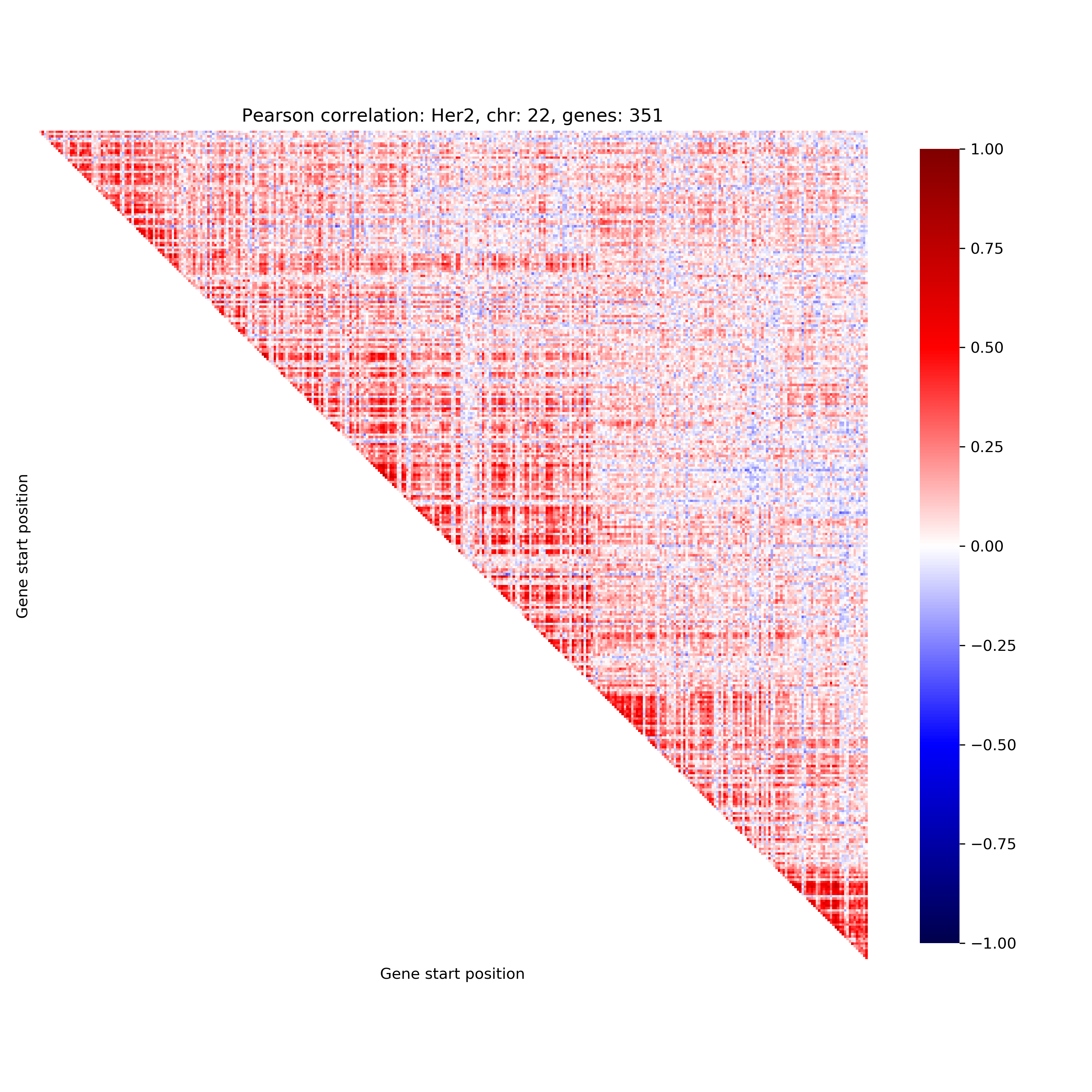

Supplement: Supplementary Material S4 — Heatmaps of Pearson correlation for each chromosome in the Luminal B phenotype. [file DataSheet_4.zip › SuppMat5/Her2-chr22.png]

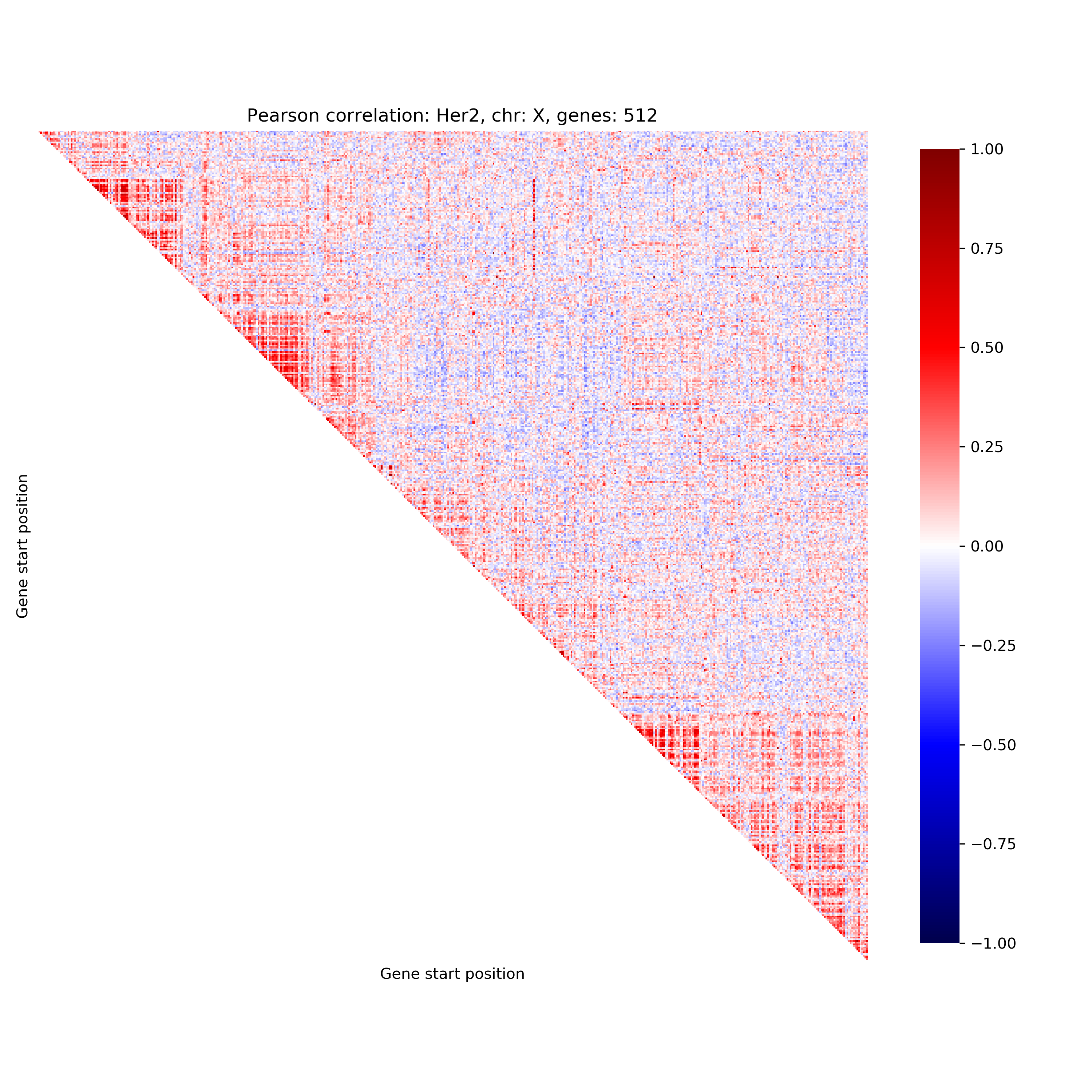

Supplement: Supplementary Material S4 — Heatmaps of Pearson correlation for each chromosome in the Luminal B phenotype. [file DataSheet_4.zip › SuppMat5/Her2-chrX.png]

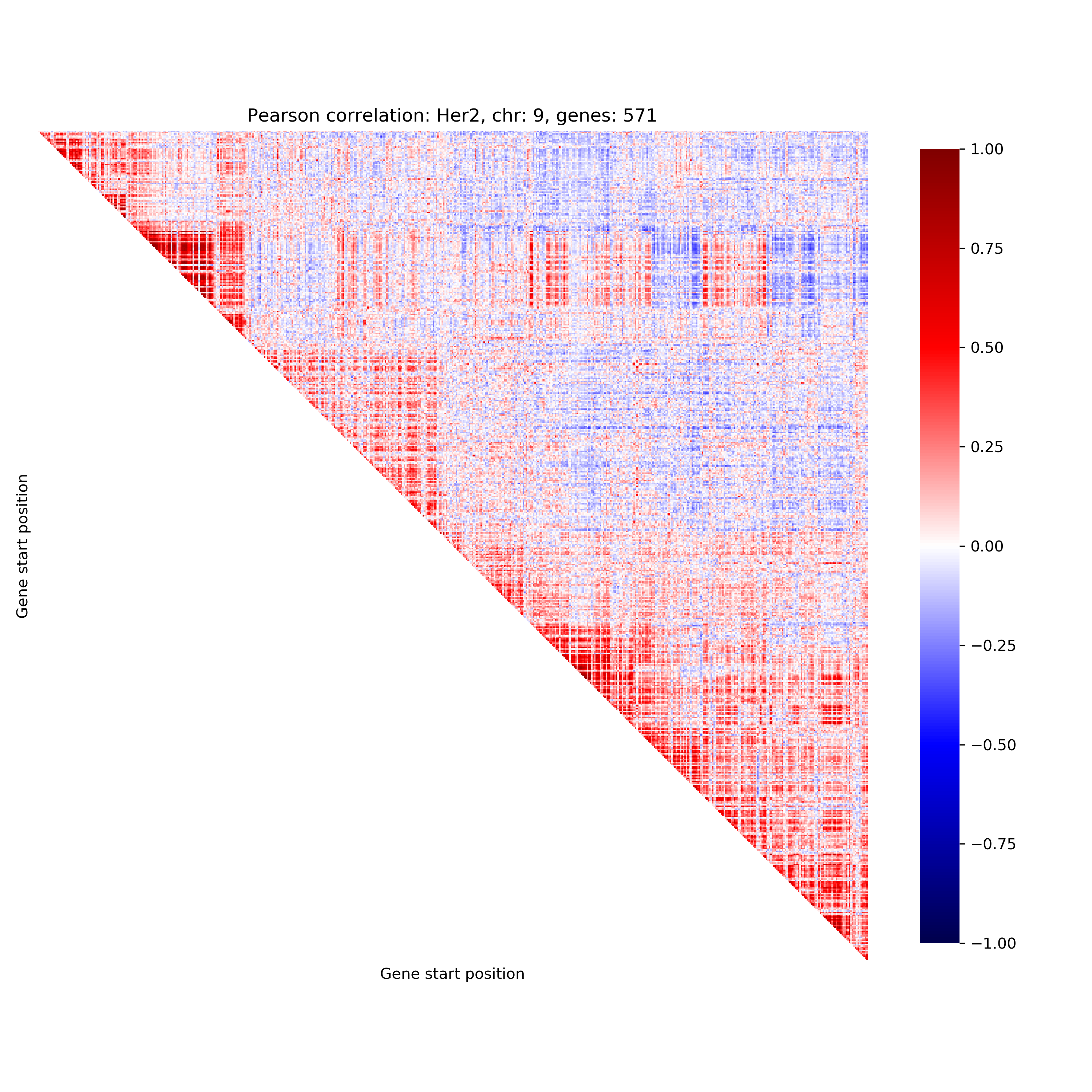

Supplement: Supplementary Material S4 — Heatmaps of Pearson correlation for each chromosome in the Luminal B phenotype. [file DataSheet_4.zip › SuppMat5/Her2-chr9.png]

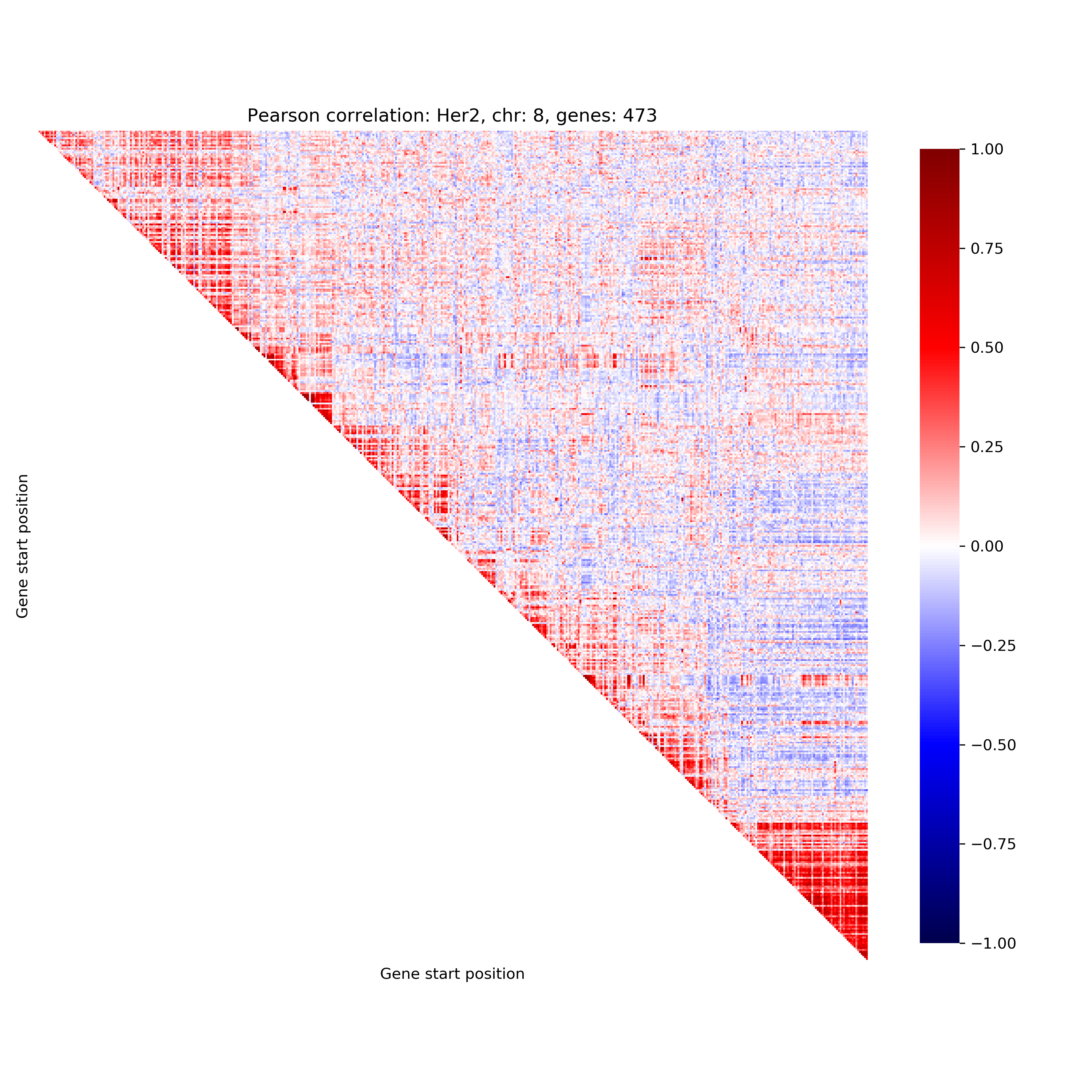

Supplement: Supplementary Material S4 — Heatmaps of Pearson correlation for each chromosome in the Luminal B phenotype. [file DataSheet_4.zip › SuppMat5/Her2-chr8.png]

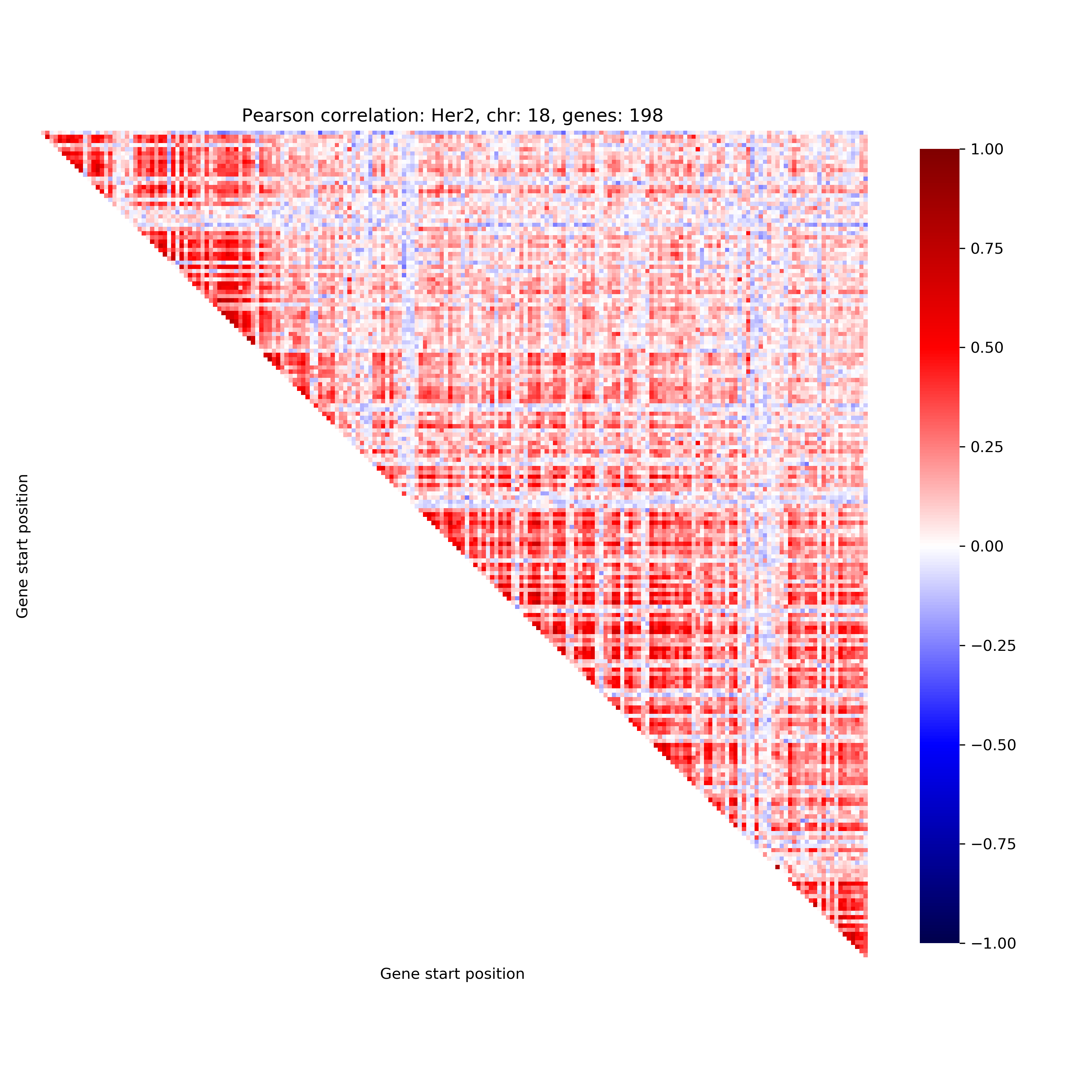

Supplement: Supplementary Material S4 — Heatmaps of Pearson correlation for each chromosome in the Luminal B phenotype. [file DataSheet_4.zip › SuppMat5/Her2-chr18.png]

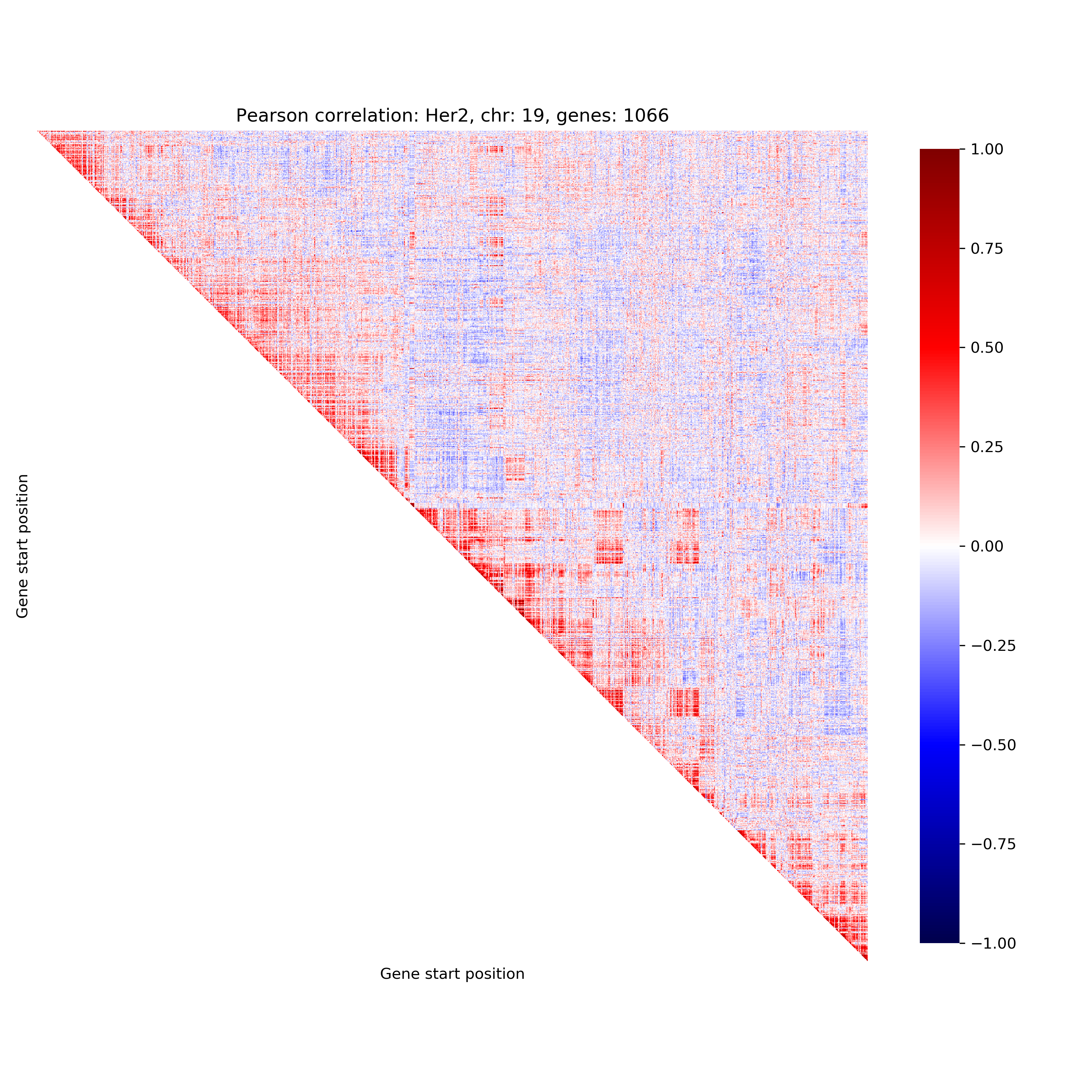

Supplement: Supplementary Material S4 — Heatmaps of Pearson correlation for each chromosome in the Luminal B phenotype. [file DataSheet_4.zip › SuppMat5/Her2-chr19.png]

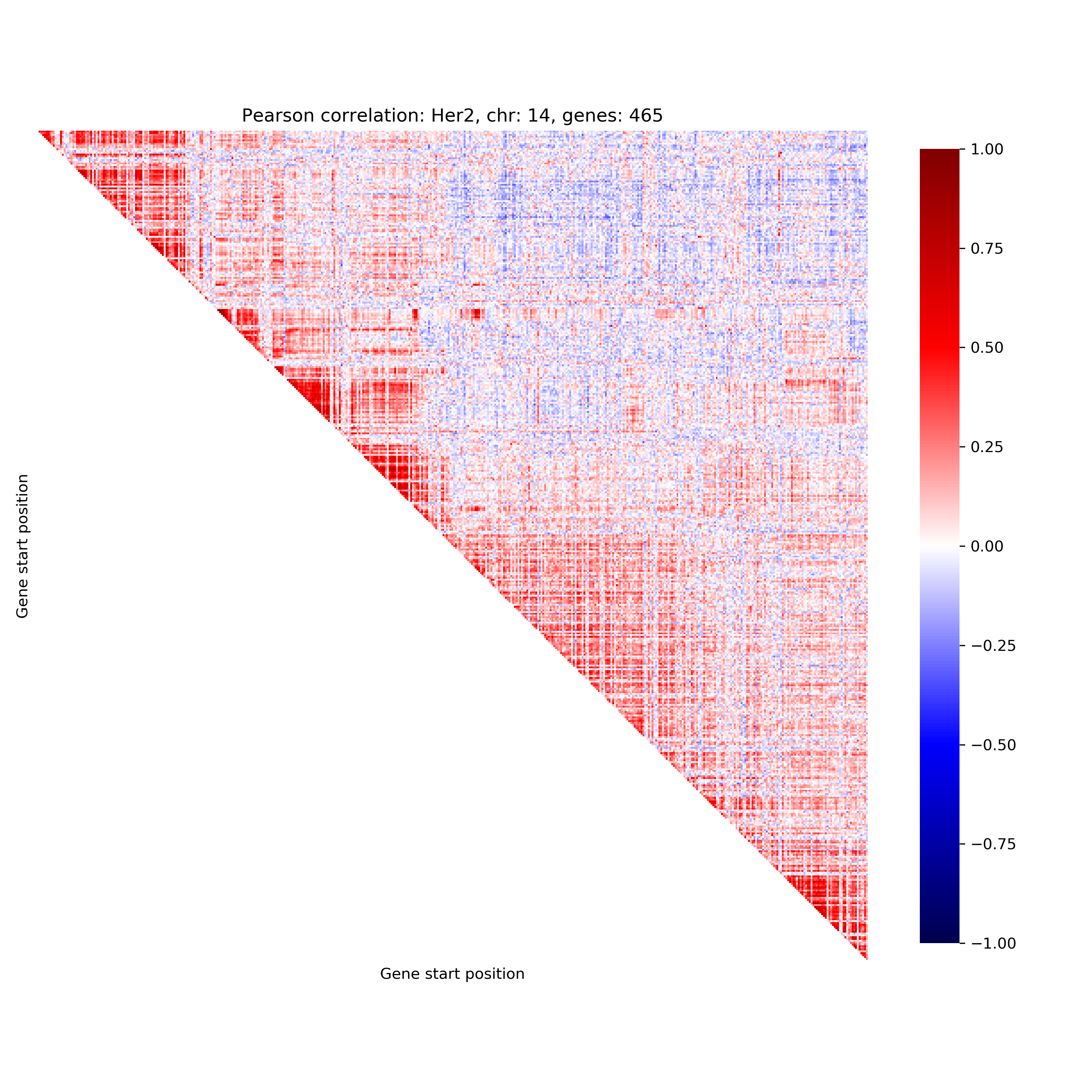

Supplement: Supplementary Material S4 — Heatmaps of Pearson correlation for each chromosome in the Luminal B phenotype. [file DataSheet_4.zip › SuppMat5/Her2-chr14.png]

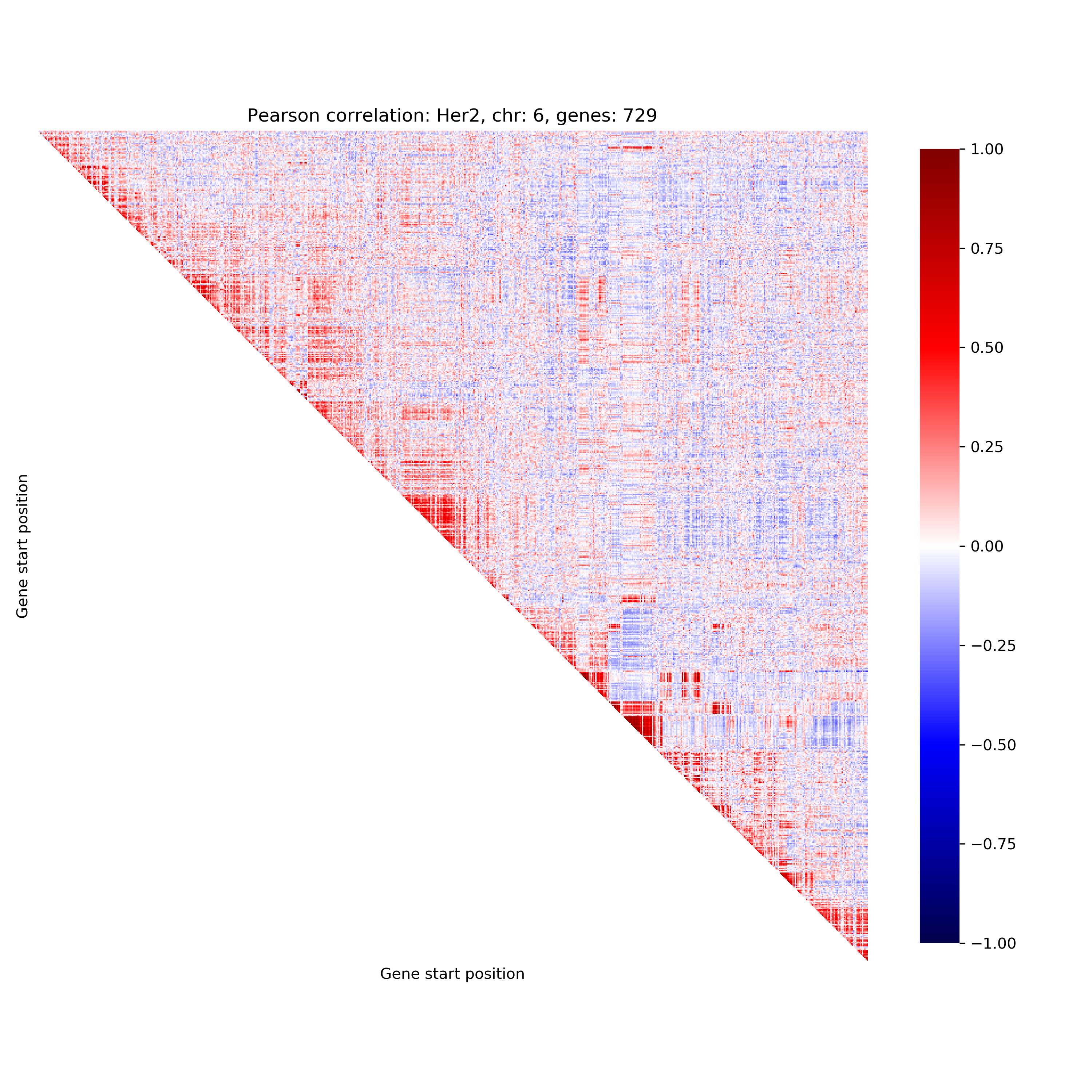

Supplement: Supplementary Material S4 — Heatmaps of Pearson correlation for each chromosome in the Luminal B phenotype. [file DataSheet_4.zip › SuppMat5/Her2-chr6.png]

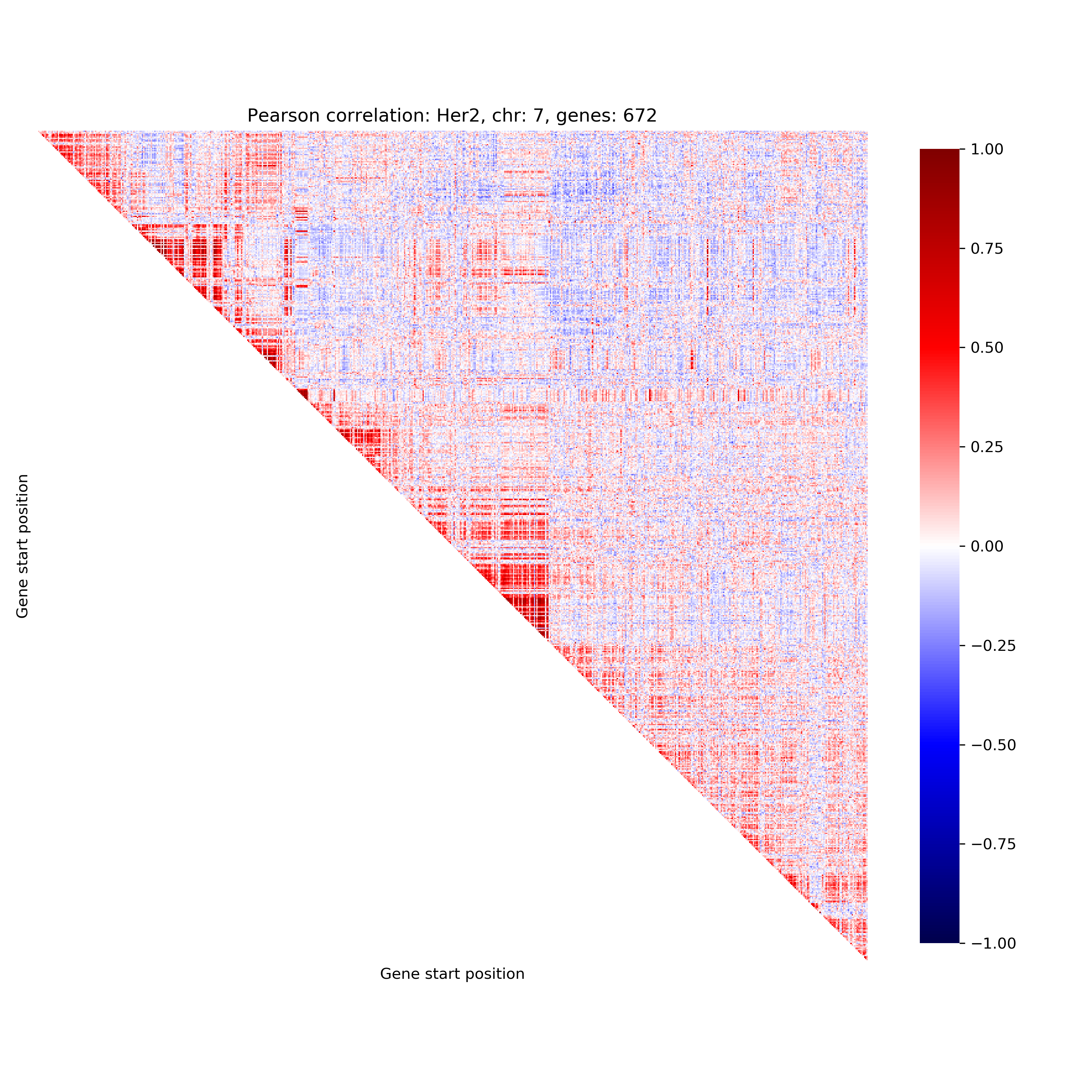

Supplement: Supplementary Material S4 — Heatmaps of Pearson correlation for each chromosome in the Luminal B phenotype. [file DataSheet_4.zip › SuppMat5/Her2-chr7.png]

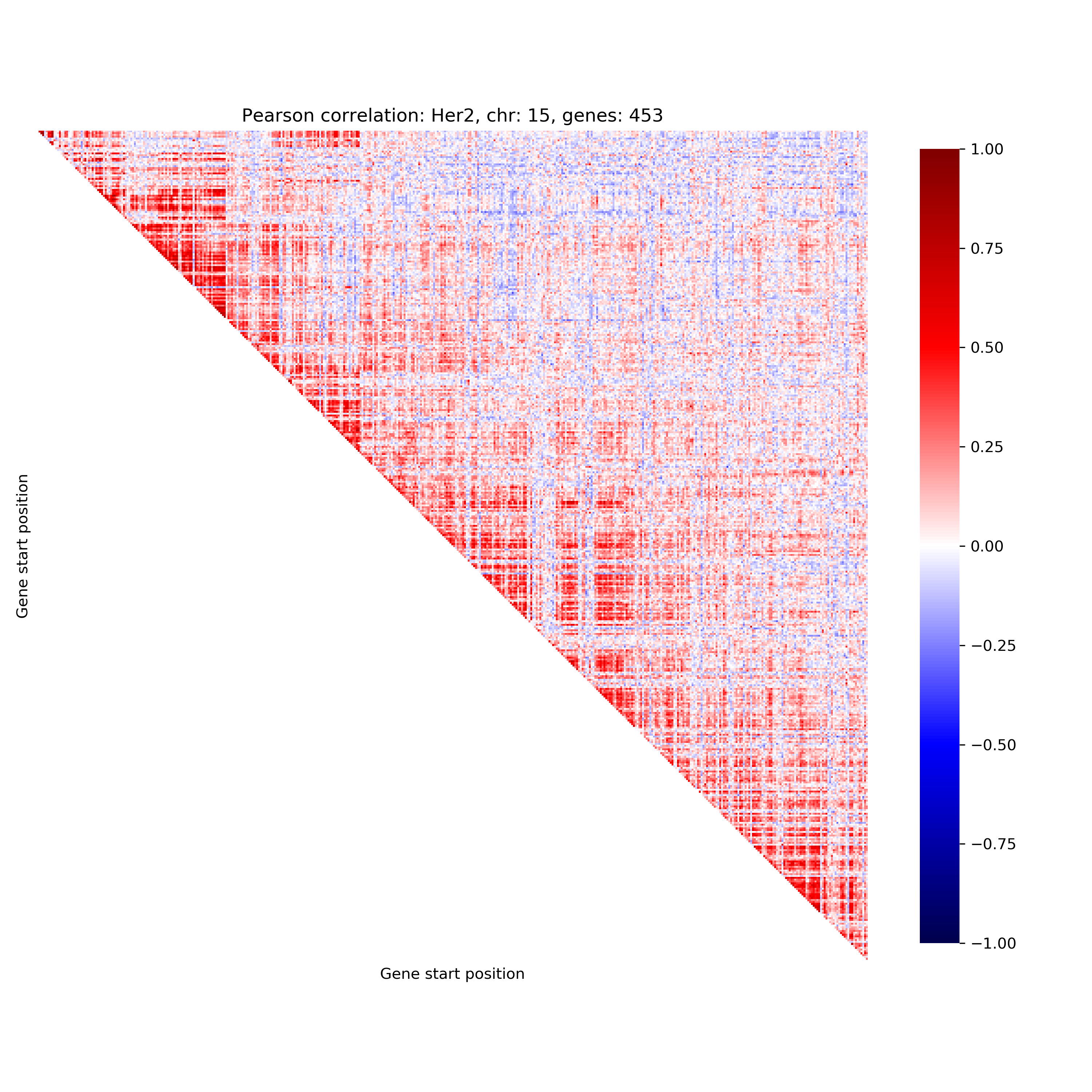

Supplement: Supplementary Material S4 — Heatmaps of Pearson correlation for each chromosome in the Luminal B phenotype. [file DataSheet_4.zip › SuppMat5/Her2-chr15.png]

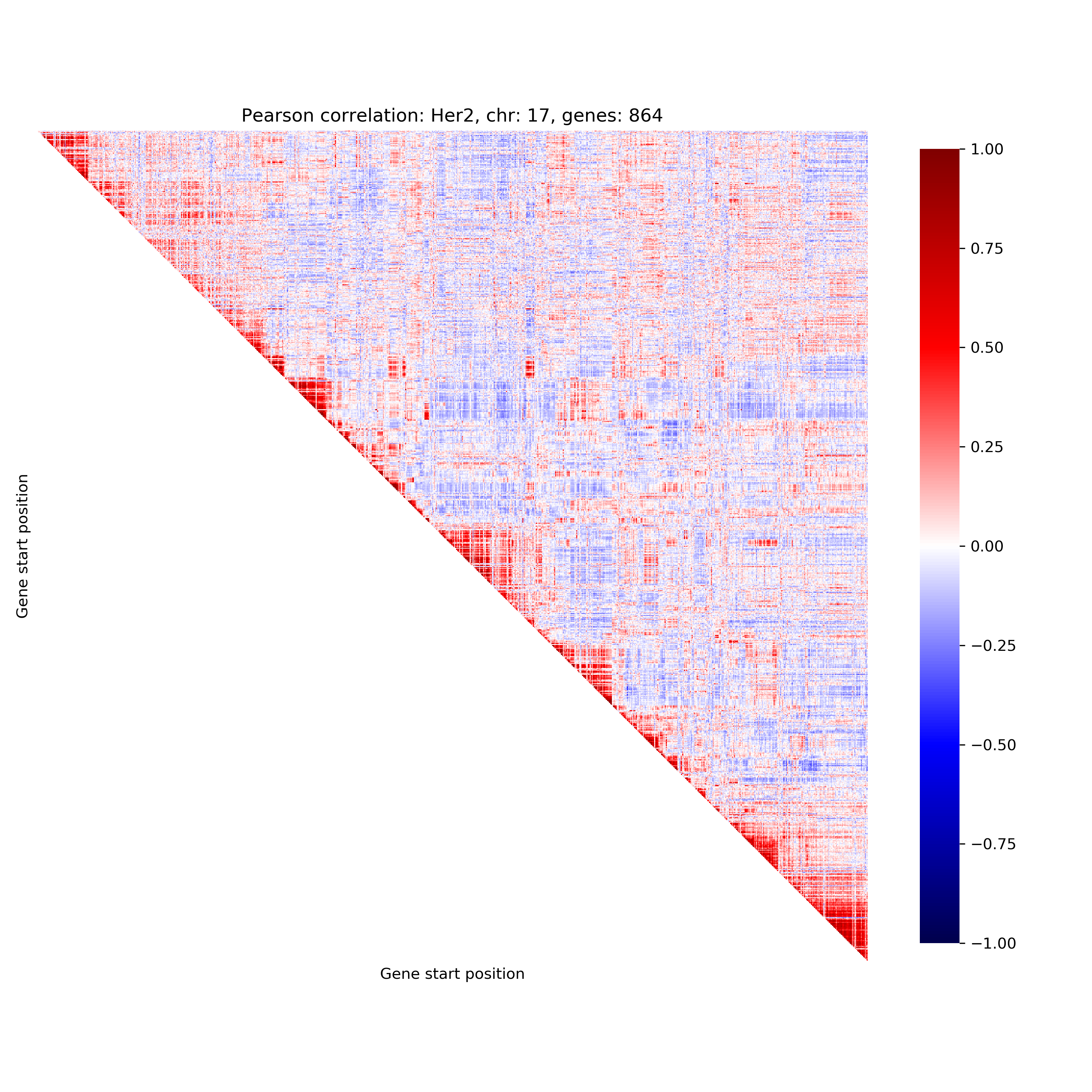

Supplement: Supplementary Material S4 — Heatmaps of Pearson correlation for each chromosome in the Luminal B phenotype. [file DataSheet_4.zip › SuppMat5/Her2-chr17.png]

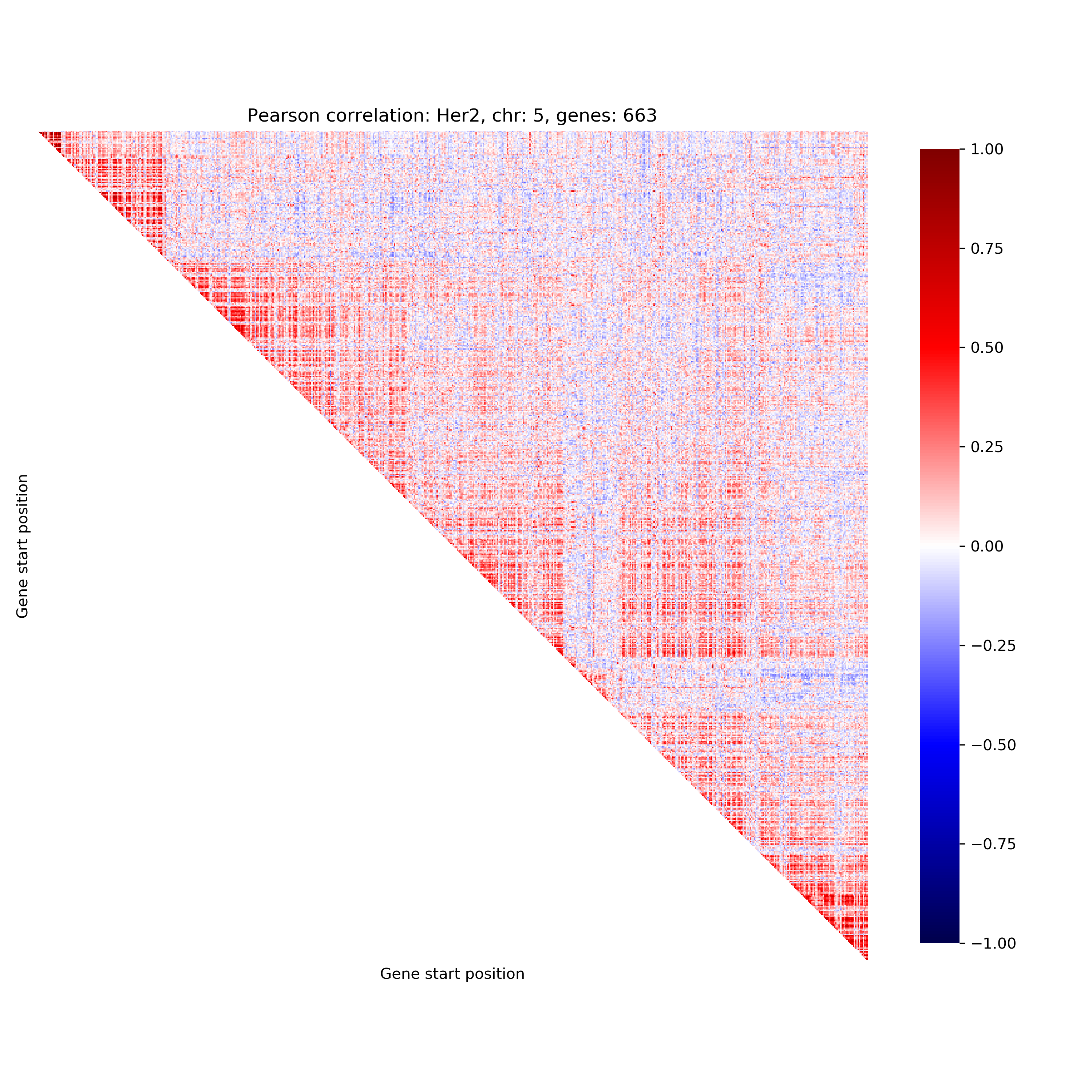

Supplement: Supplementary Material S4 — Heatmaps of Pearson correlation for each chromosome in the Luminal B phenotype. [file DataSheet_4.zip › SuppMat5/Her2-chr5.png]

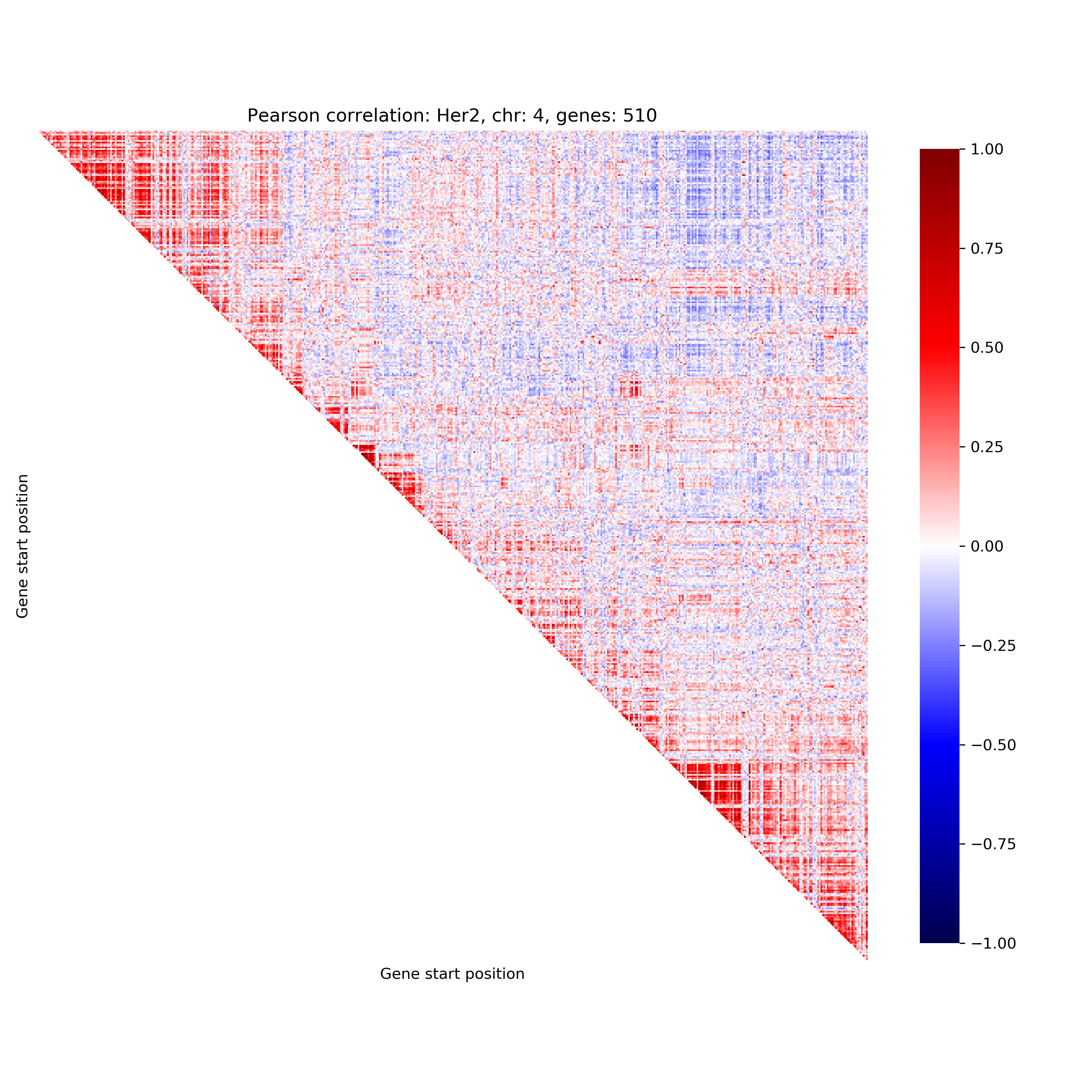

Supplement: Supplementary Material S4 — Heatmaps of Pearson correlation for each chromosome in the Luminal B phenotype. [file DataSheet_4.zip › SuppMat5/Her2-chr4.png]

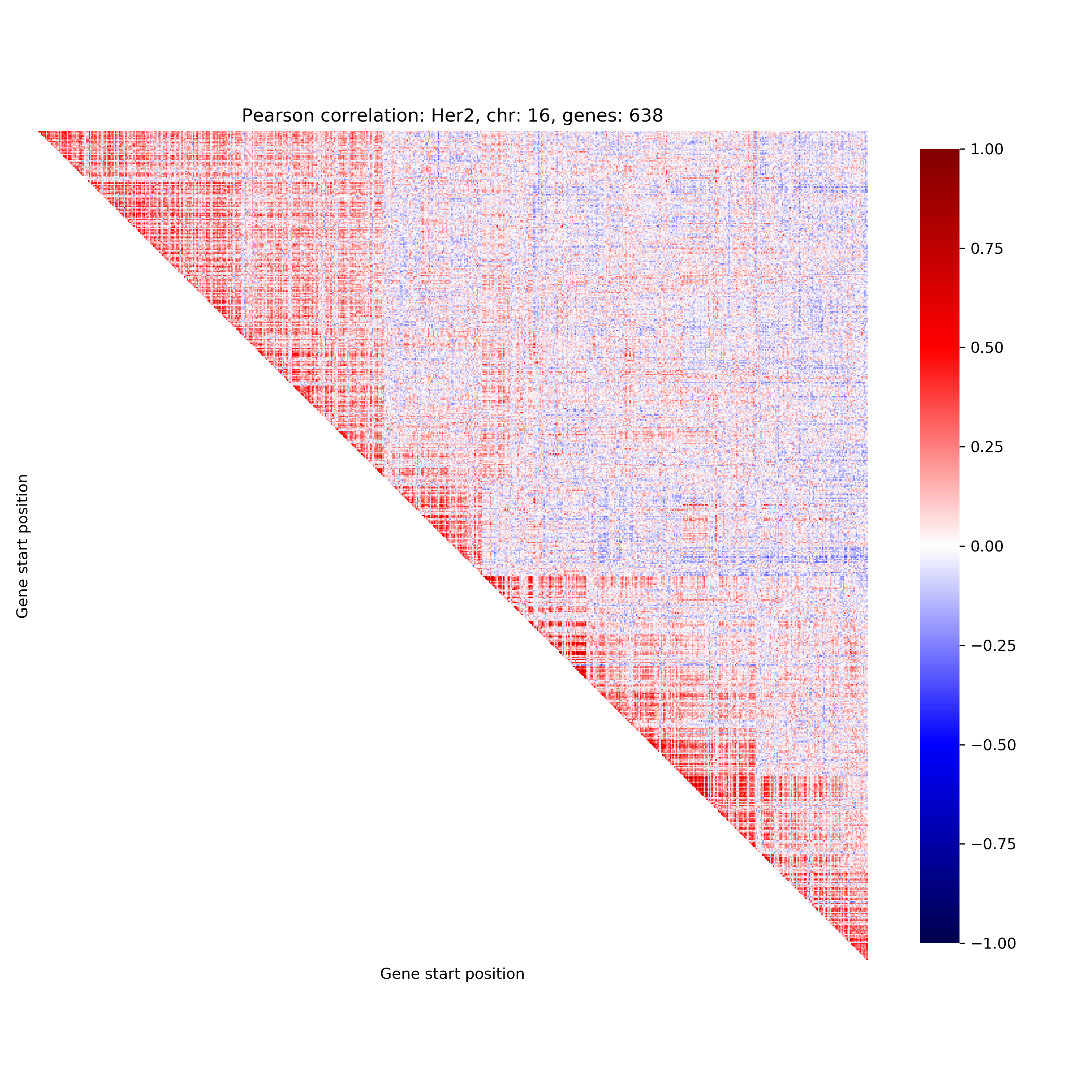

Supplement: Supplementary Material S4 — Heatmaps of Pearson correlation for each chromosome in the Luminal B phenotype. [file DataSheet_4.zip › SuppMat5/Her2-chr16.png]

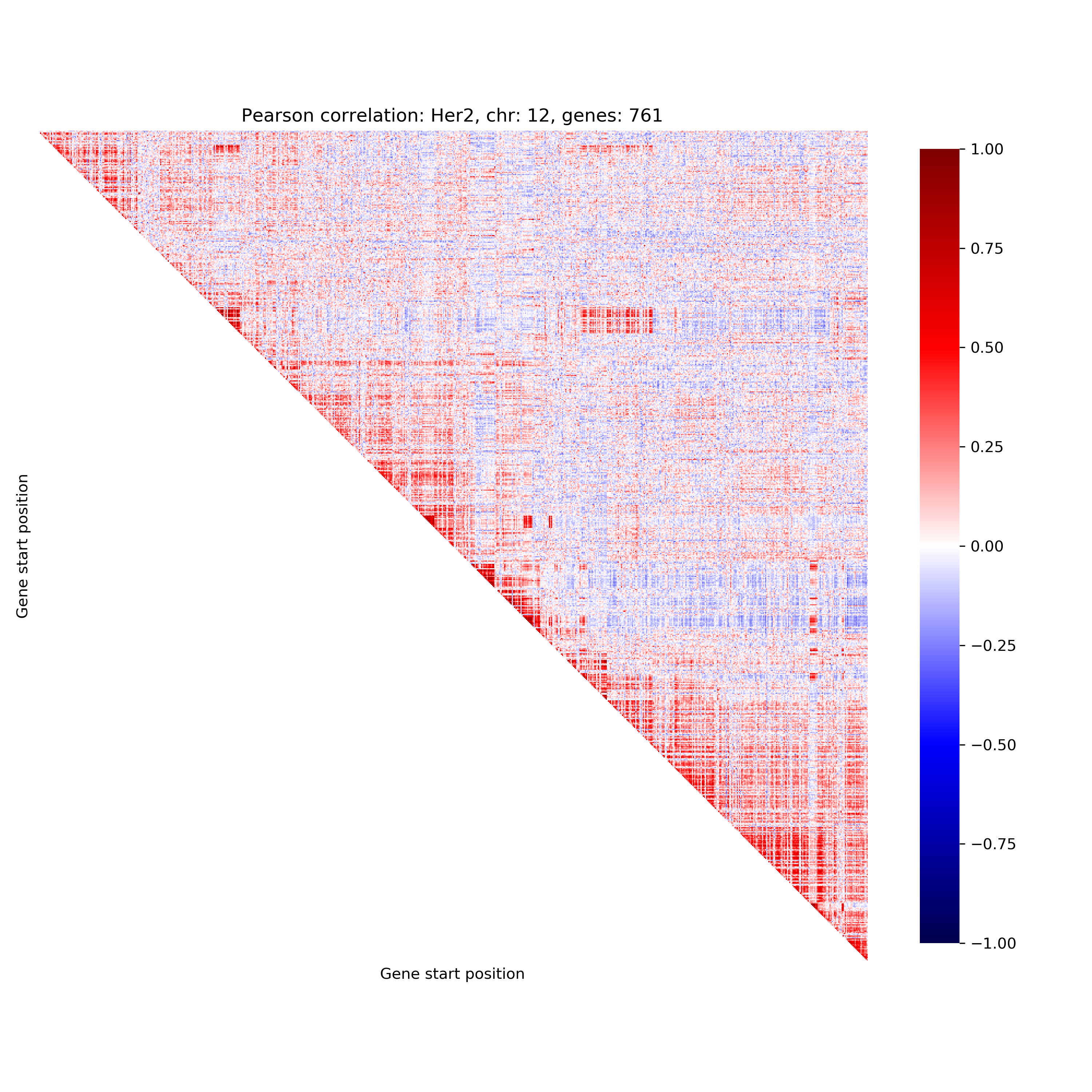

Supplement: Supplementary Material S4 — Heatmaps of Pearson correlation for each chromosome in the Luminal B phenotype. [file DataSheet_4.zip › SuppMat5/Her2-chr12.png]

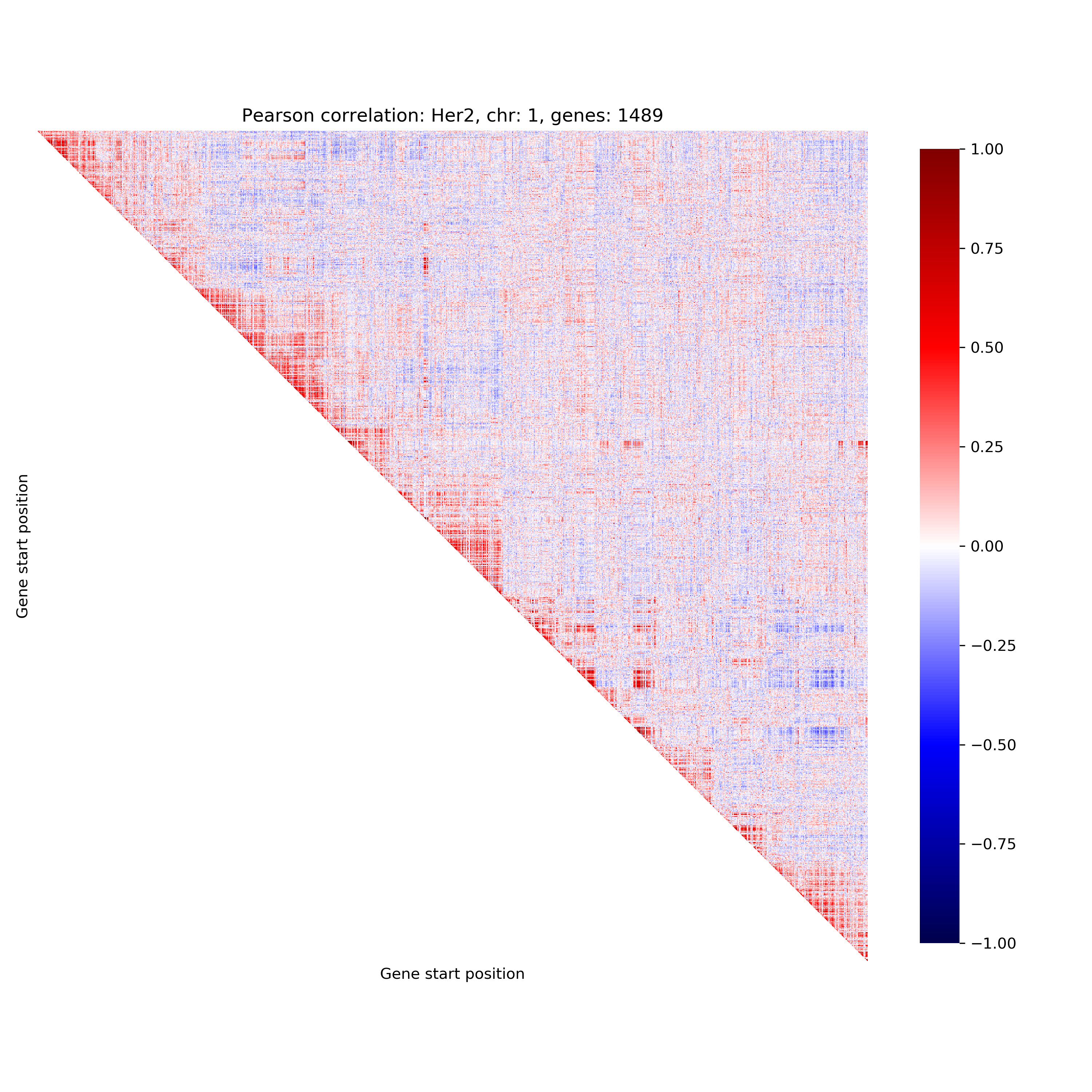

Supplement: Supplementary Material S4 — Heatmaps of Pearson correlation for each chromosome in the Luminal B phenotype. [file DataSheet_4.zip › SuppMat5/Her2-chr1.png]

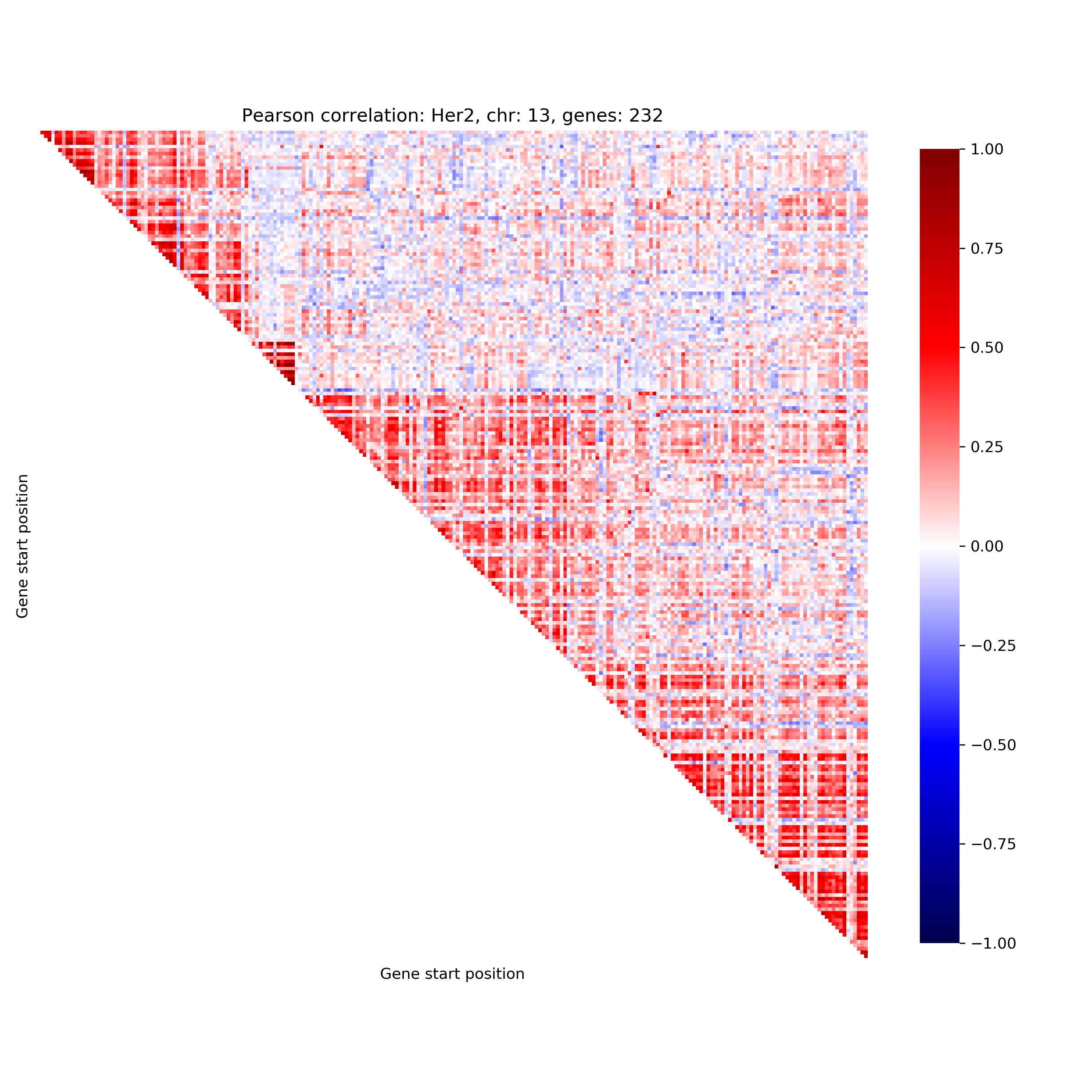

Supplement: Supplementary Material S4 — Heatmaps of Pearson correlation for each chromosome in the Luminal B phenotype. [file DataSheet_4.zip › SuppMat5/Her2-chr13.png]

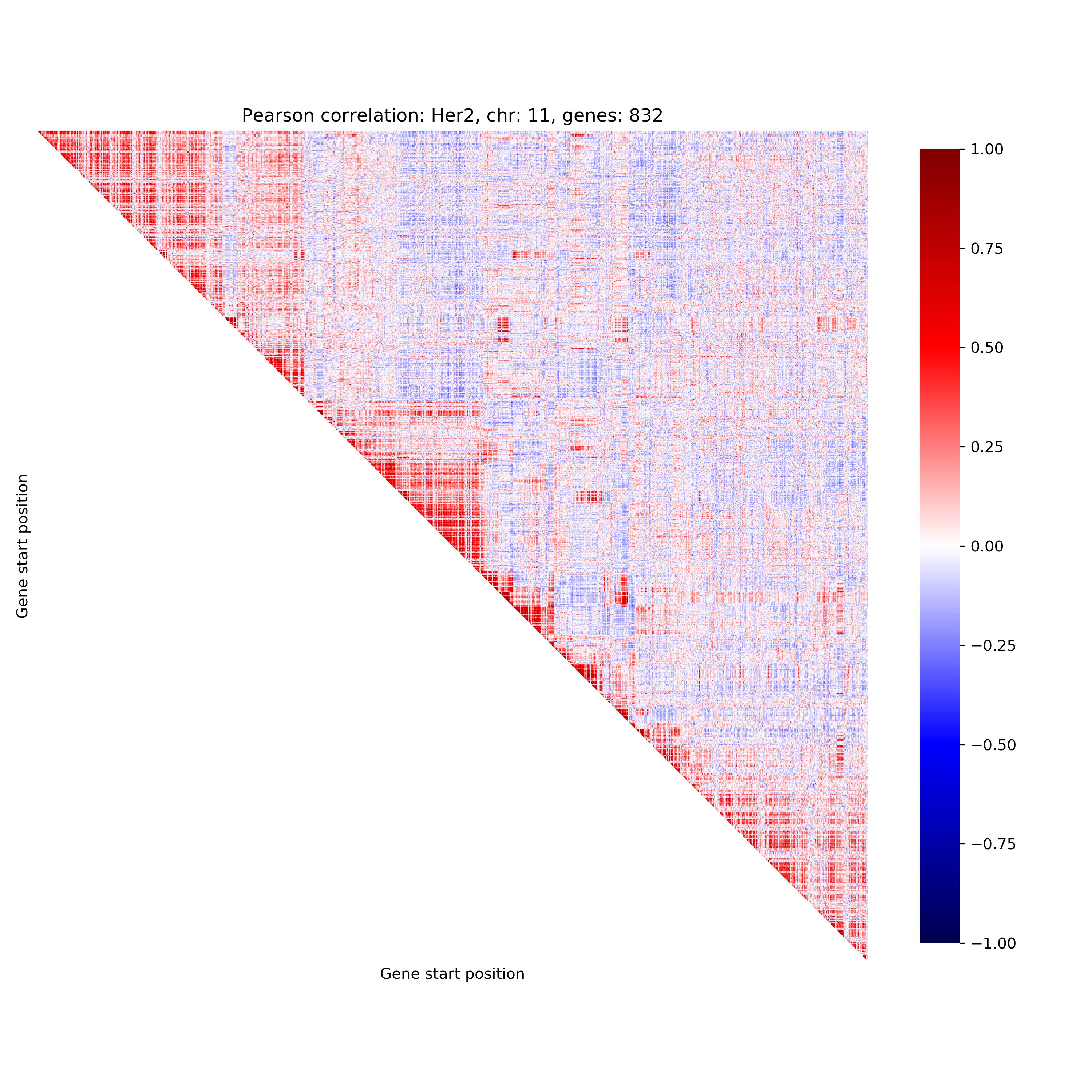

Supplement: Supplementary Material S4 — Heatmaps of Pearson correlation for each chromosome in the Luminal B phenotype. [file DataSheet_4.zip › SuppMat5/Her2-chr11.png]

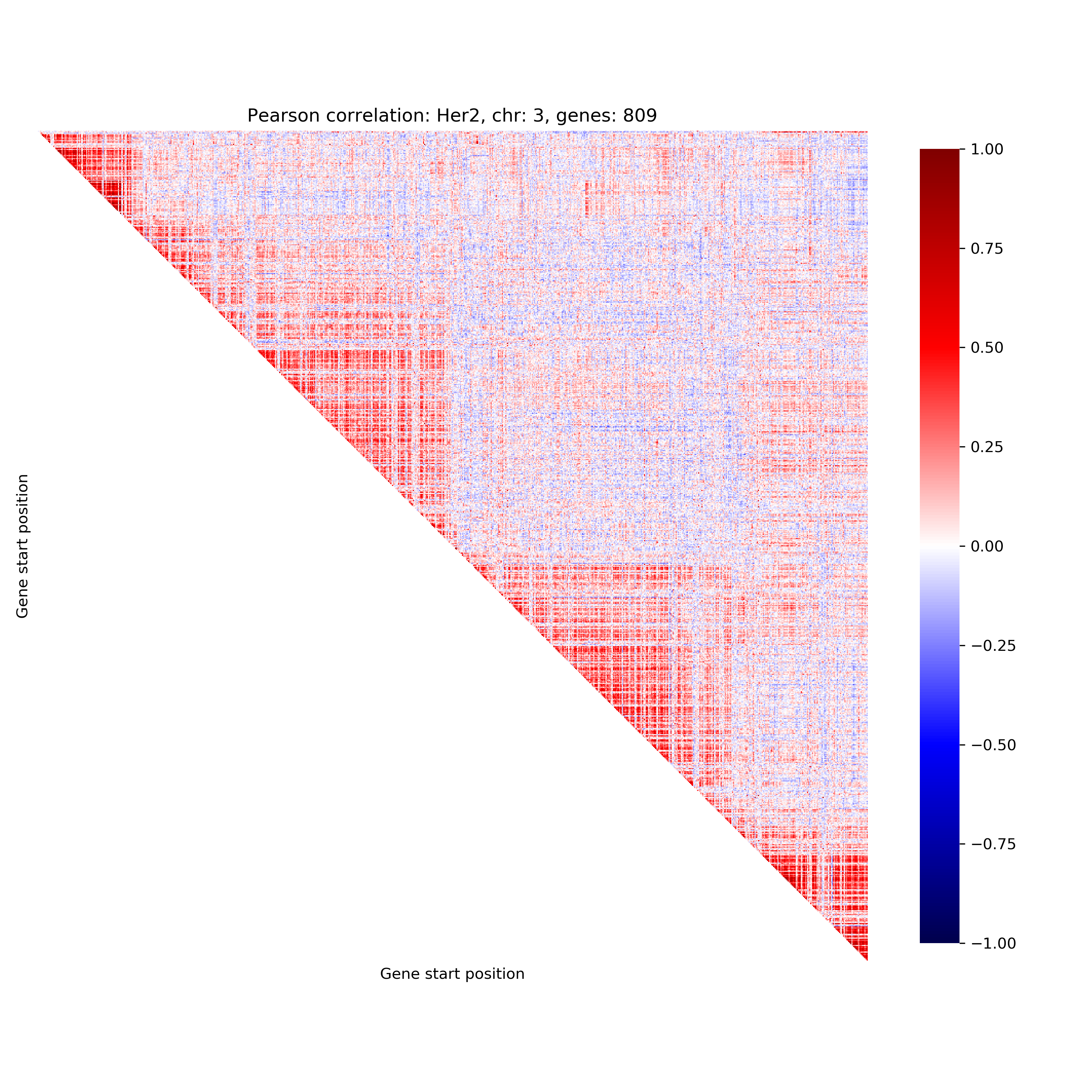

Supplement: Supplementary Material S4 — Heatmaps of Pearson correlation for each chromosome in the Luminal B phenotype. [file DataSheet_4.zip › SuppMat5/Her2-chr3.png]

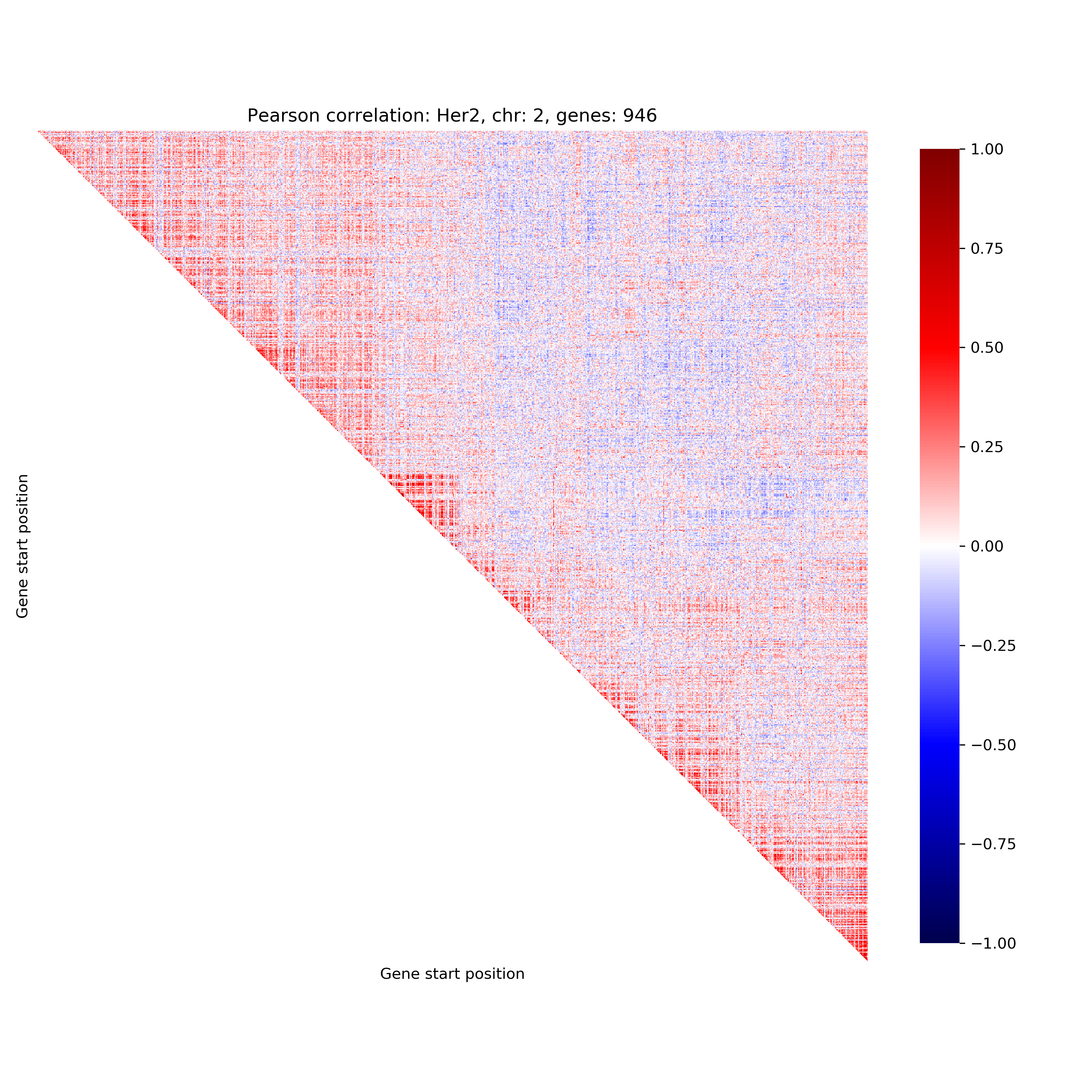

Supplement: Supplementary Material S4 — Heatmaps of Pearson correlation for each chromosome in the Luminal B phenotype. [file DataSheet_4.zip › SuppMat5/Her2-chr2.png]

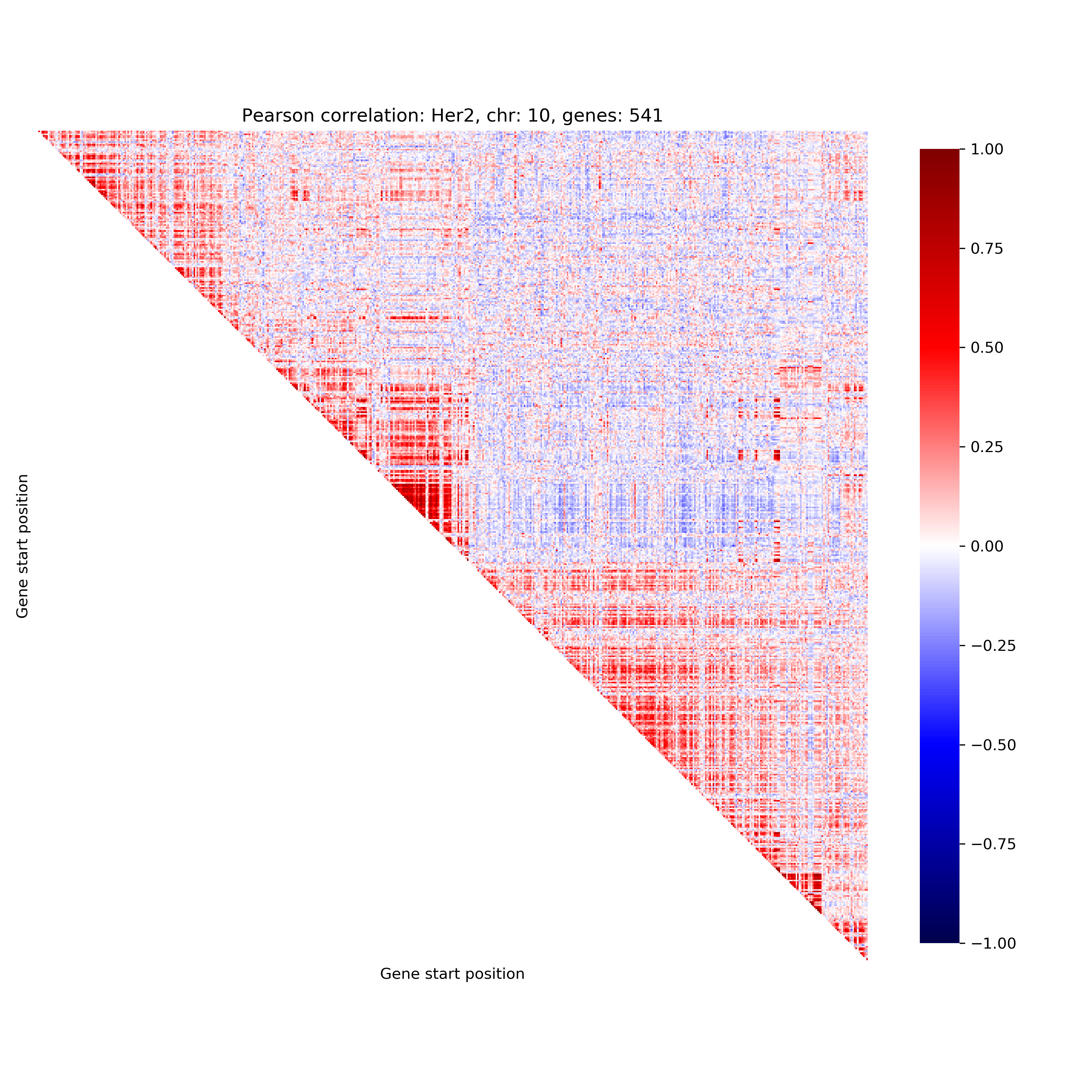

Supplement: Supplementary Material S4 — Heatmaps of Pearson correlation for each chromosome in the Luminal B phenotype. [file DataSheet_4.zip › SuppMat5/Her2-chr10.png]

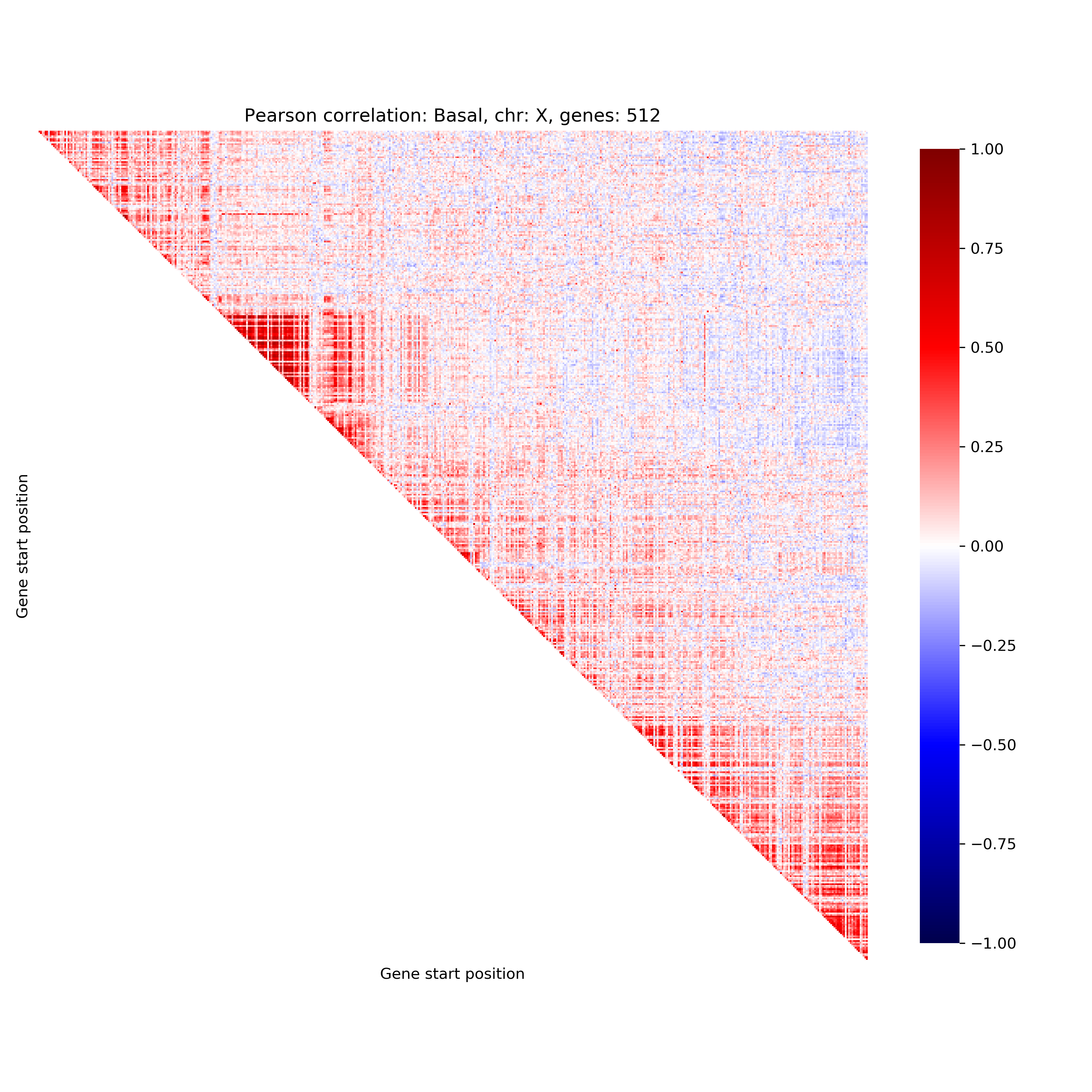

Supplement: Supplementary Material S5 — Heatmaps of Pearson correlation for each chromosome in the HER2+ phenotype. [file DataSheet_5.zip › SuppMat6/Basal-chrX.png]

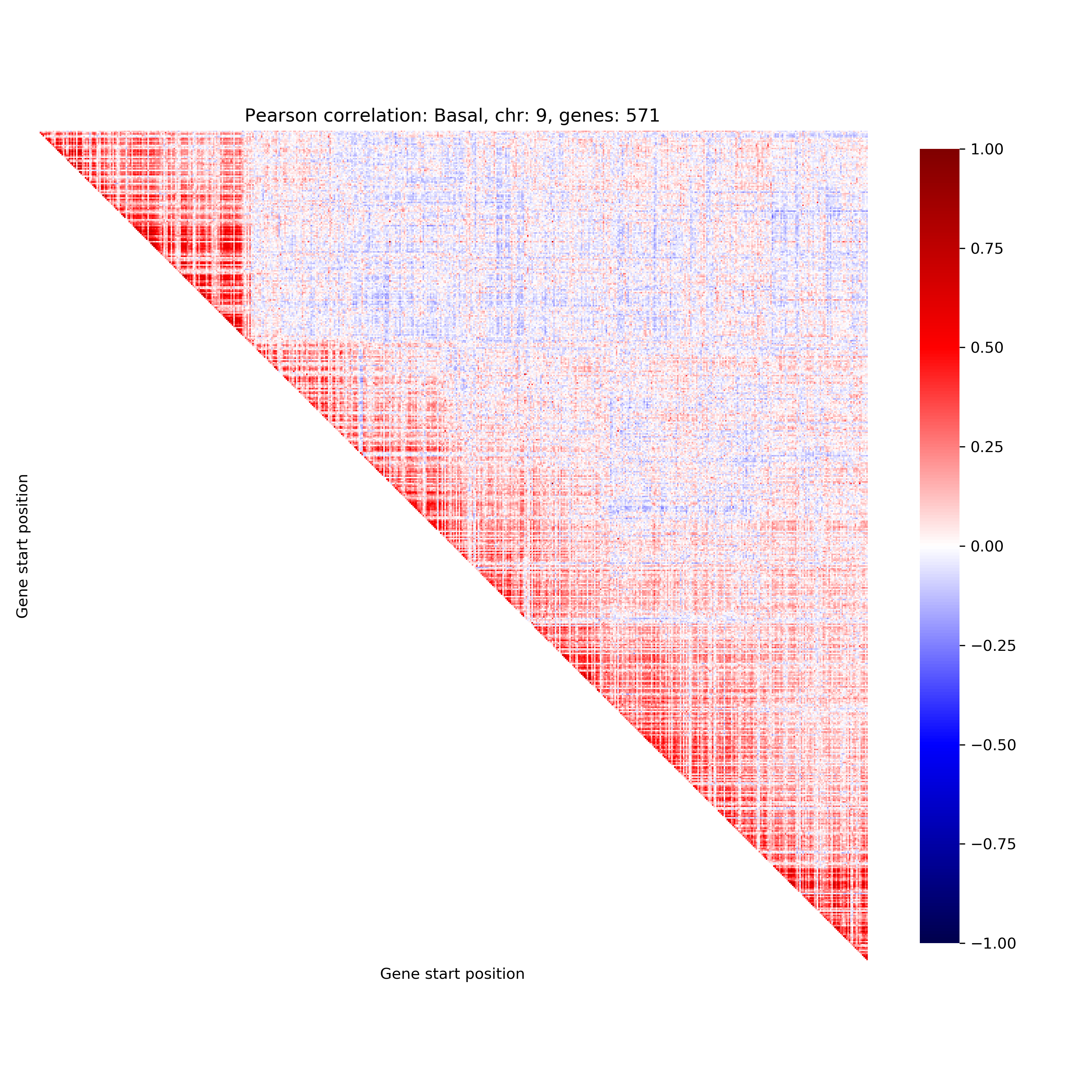

Supplement: Supplementary Material S5 — Heatmaps of Pearson correlation for each chromosome in the HER2+ phenotype. [file DataSheet_5.zip › SuppMat6/Basal-chr9.png]

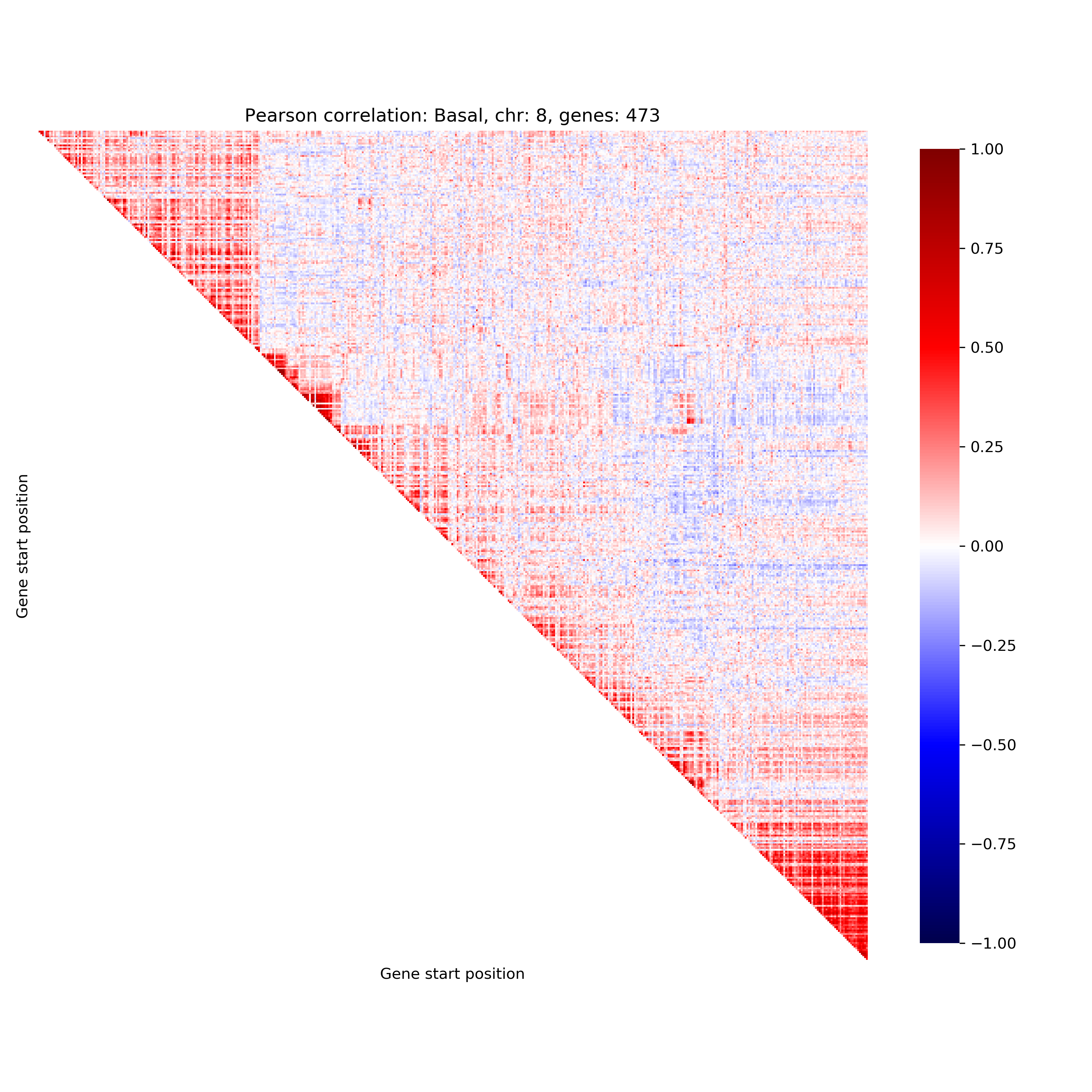

Supplement: Supplementary Material S5 — Heatmaps of Pearson correlation for each chromosome in the HER2+ phenotype. [file DataSheet_5.zip › SuppMat6/Basal-chr8.png]

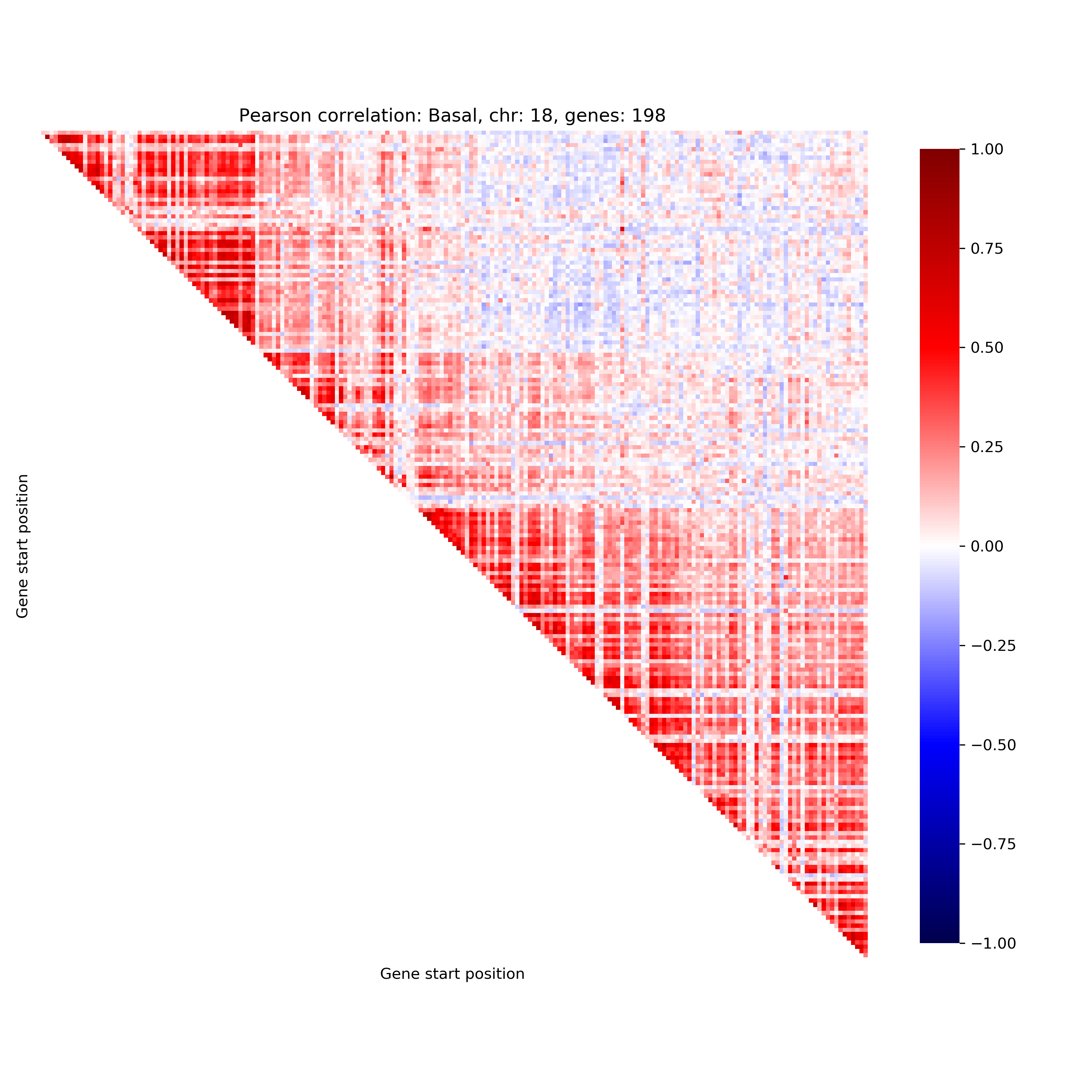

Supplement: Supplementary Material S5 — Heatmaps of Pearson correlation for each chromosome in the HER2+ phenotype. [file DataSheet_5.zip › SuppMat6/Basal-chr18.png]

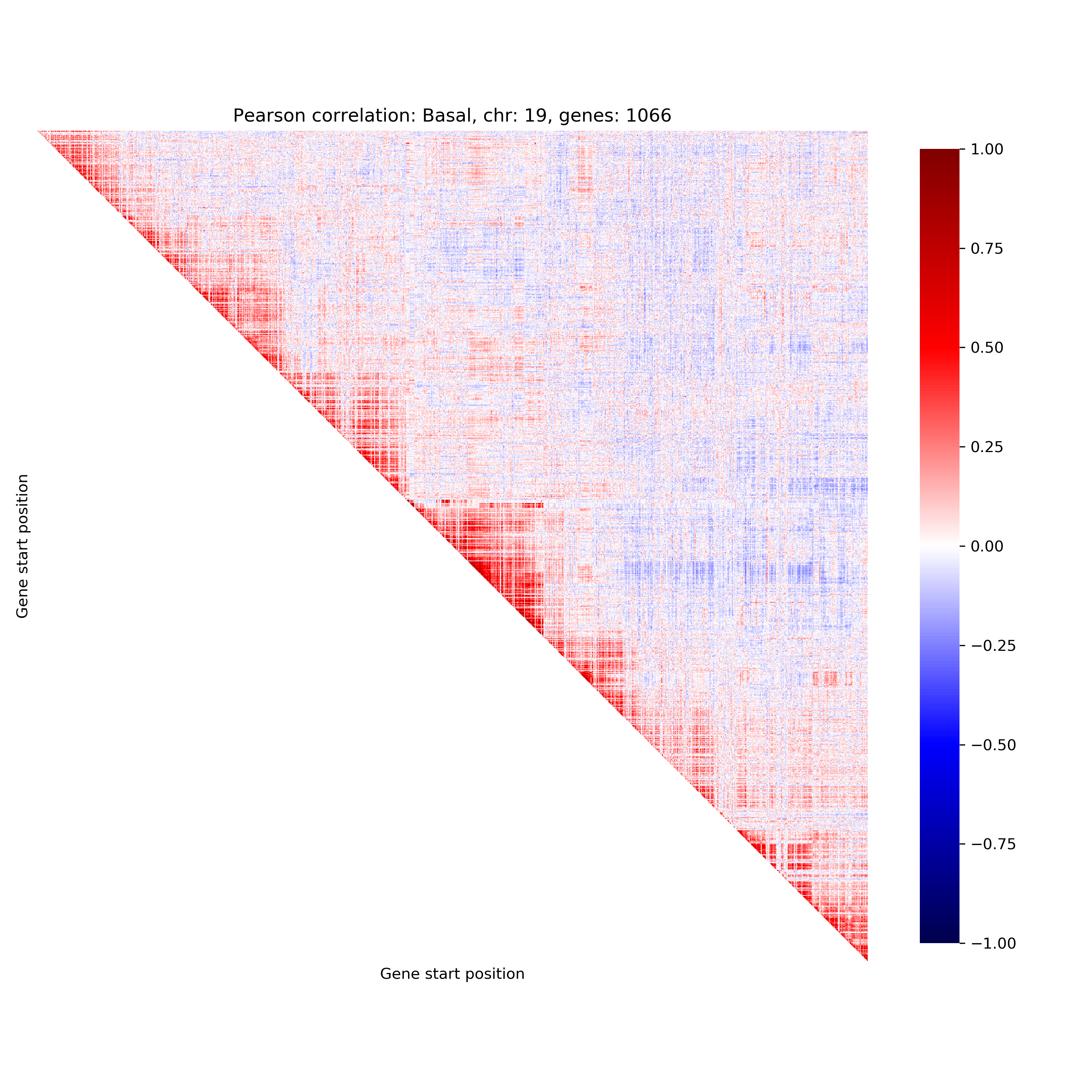

Supplement: Supplementary Material S5 — Heatmaps of Pearson correlation for each chromosome in the HER2+ phenotype. [file DataSheet_5.zip › SuppMat6/Basal-chr19.png]

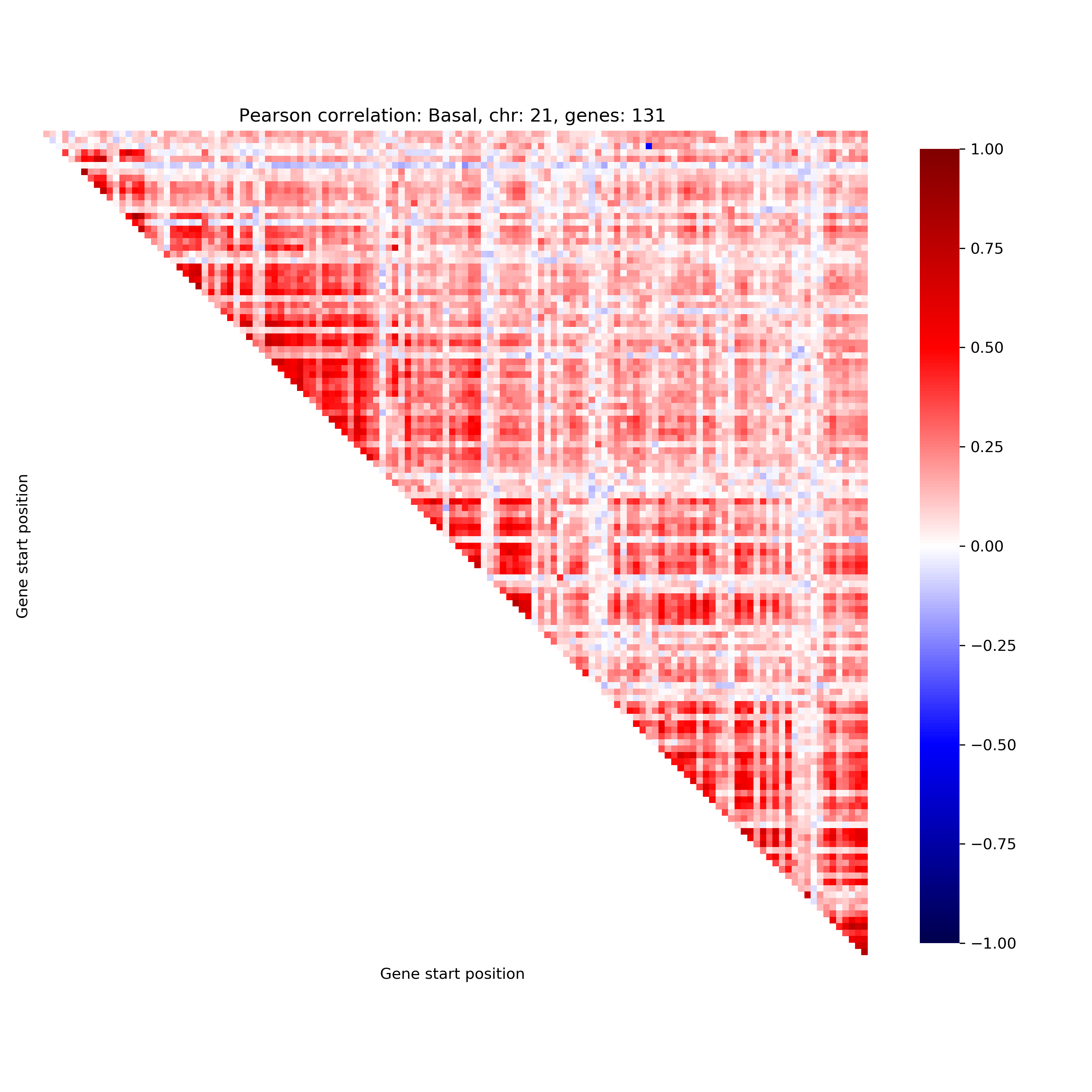

Supplement: Supplementary Material S5 — Heatmaps of Pearson correlation for each chromosome in the HER2+ phenotype. [file DataSheet_5.zip › SuppMat6/Basal-chr21.png]
