# Supplementary figures and images for: Gene Co-Expression in Breast Cancer: A Matter of Distance (part 2 of 5)
Source: Front Oncol. 2021 Nov 17;11:726493. doi: 10.3389/fonc.2021.726493 (PMC8636045; doi:10.3389/fonc.2021.726493)

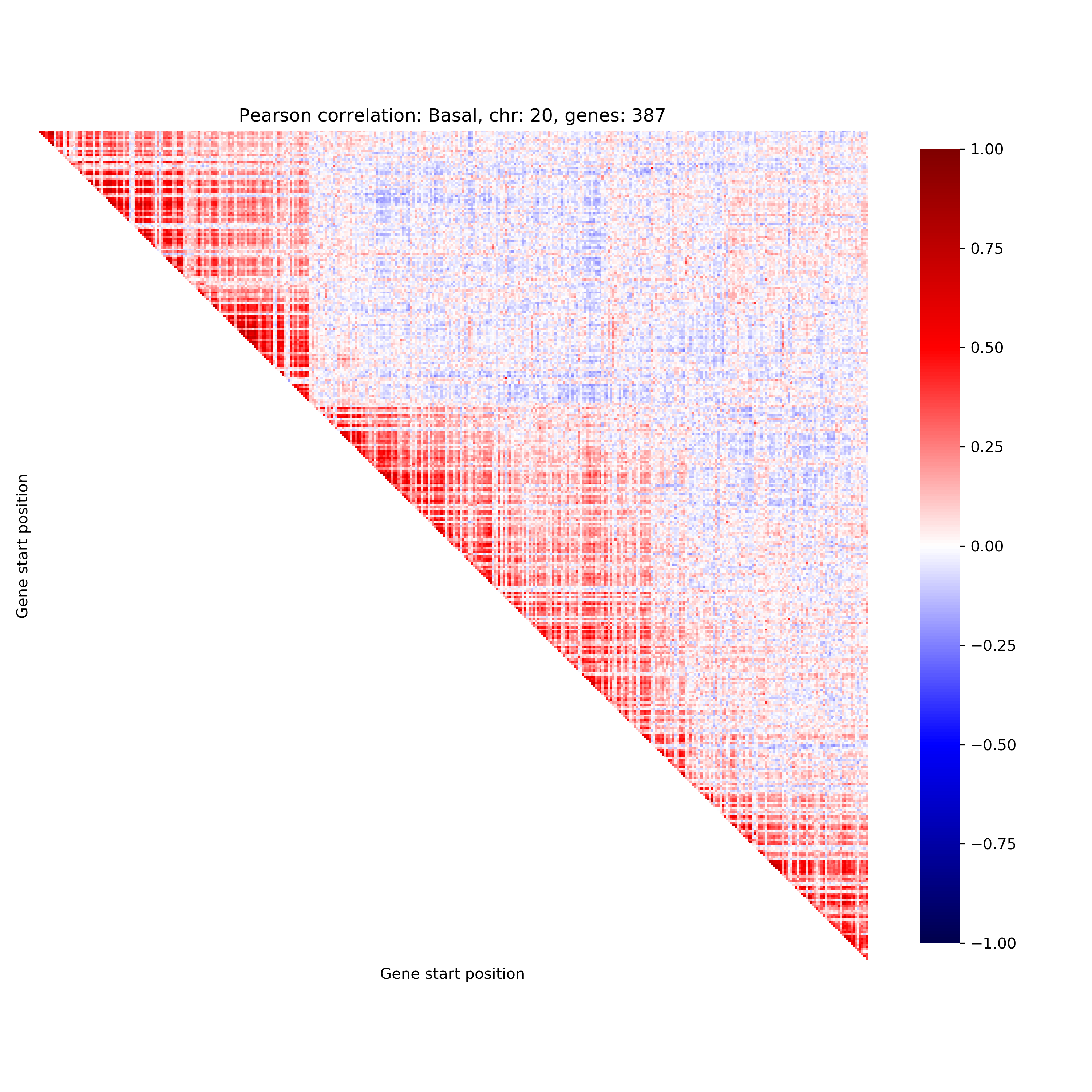

Supplement: Supplementary Material S5 — Heatmaps of Pearson correlation for each chromosome in the HER2+ phenotype. [file DataSheet_5.zip › SuppMat6/Basal-chr20.png]

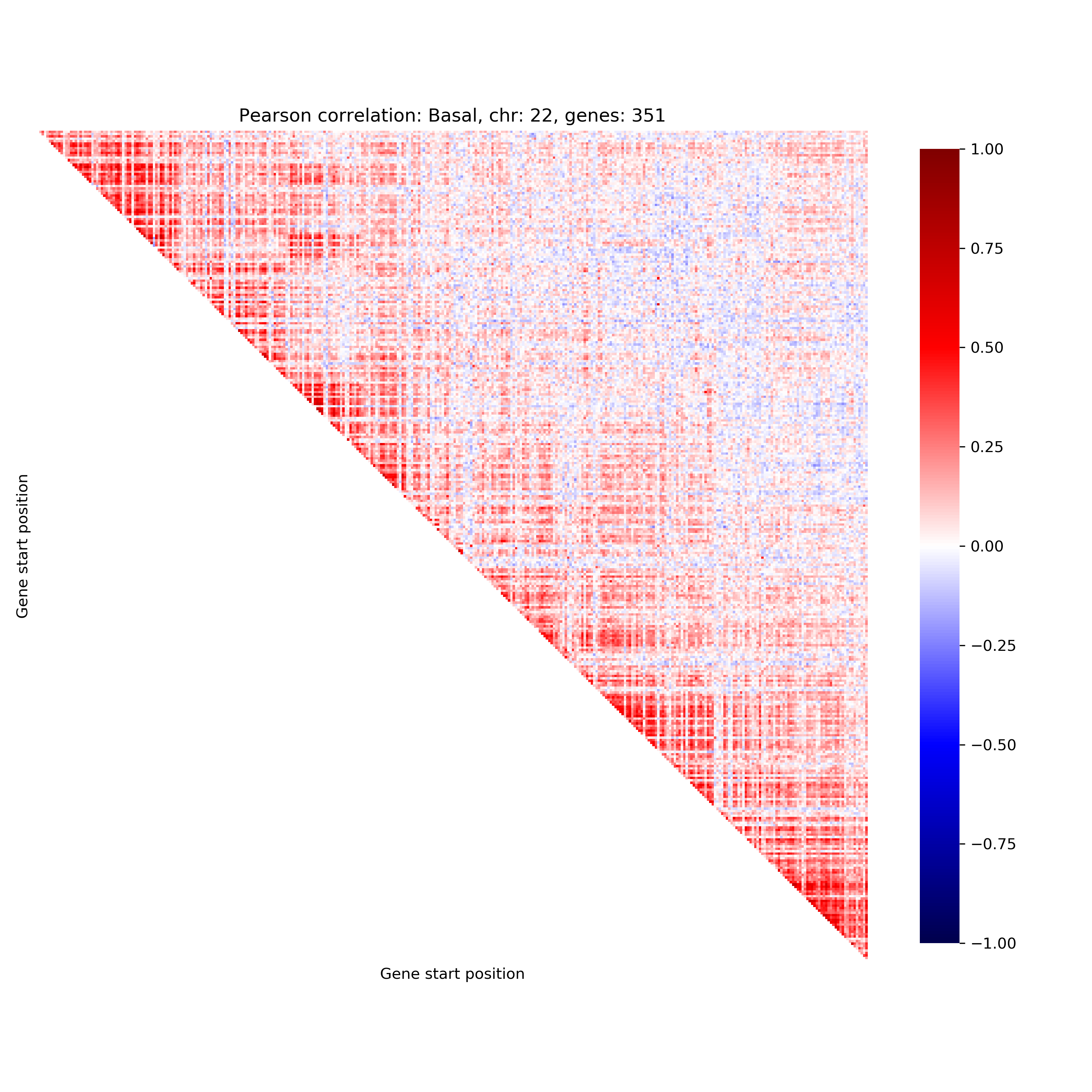

Supplement: Supplementary Material S5 — Heatmaps of Pearson correlation for each chromosome in the HER2+ phenotype. [file DataSheet_5.zip › SuppMat6/Basal-chr22.png]

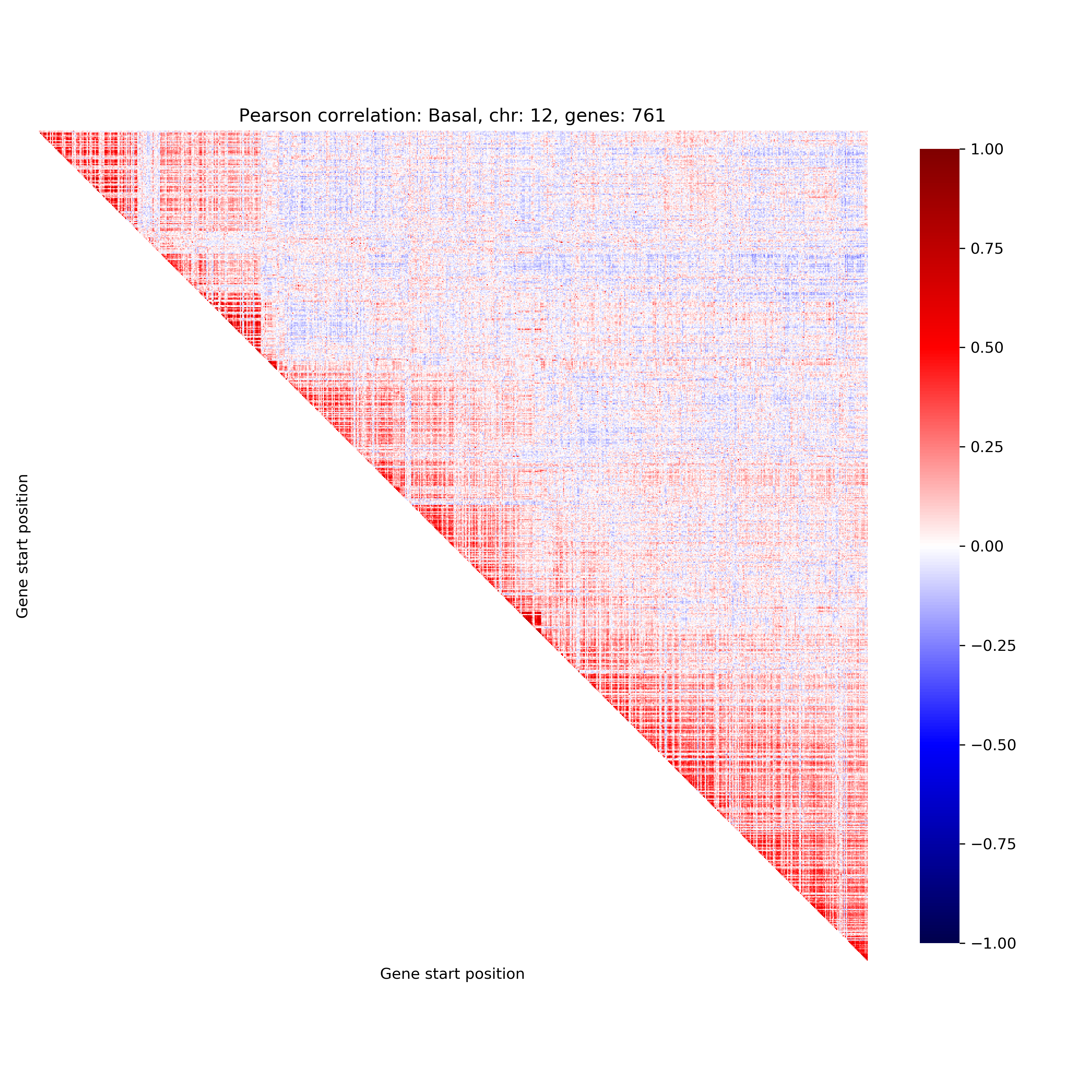

Supplement: Supplementary Material S5 — Heatmaps of Pearson correlation for each chromosome in the HER2+ phenotype. [file DataSheet_5.zip › SuppMat6/Basal-chr12.png]

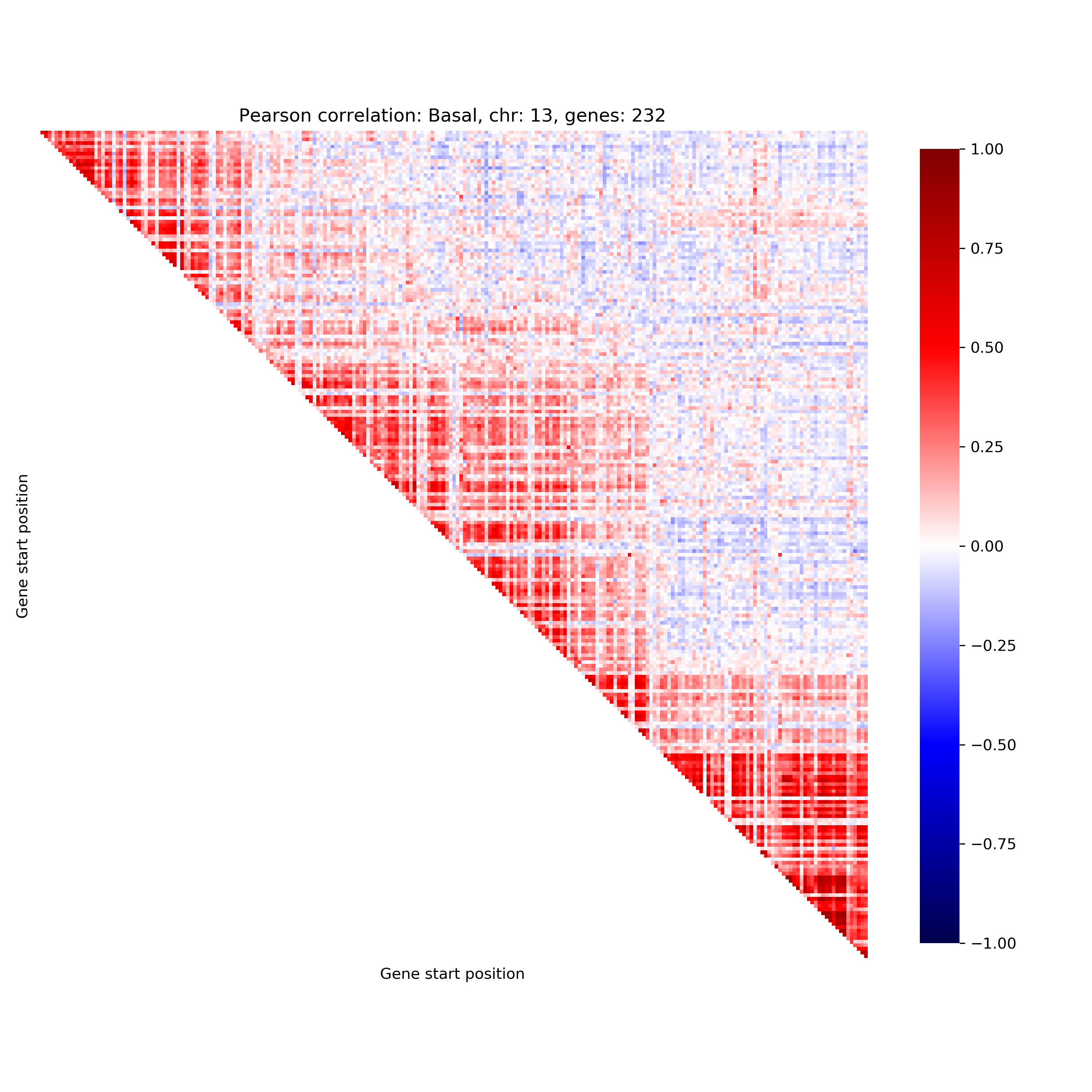

Supplement: Supplementary Material S5 — Heatmaps of Pearson correlation for each chromosome in the HER2+ phenotype. [file DataSheet_5.zip › SuppMat6/Basal-chr13.png]

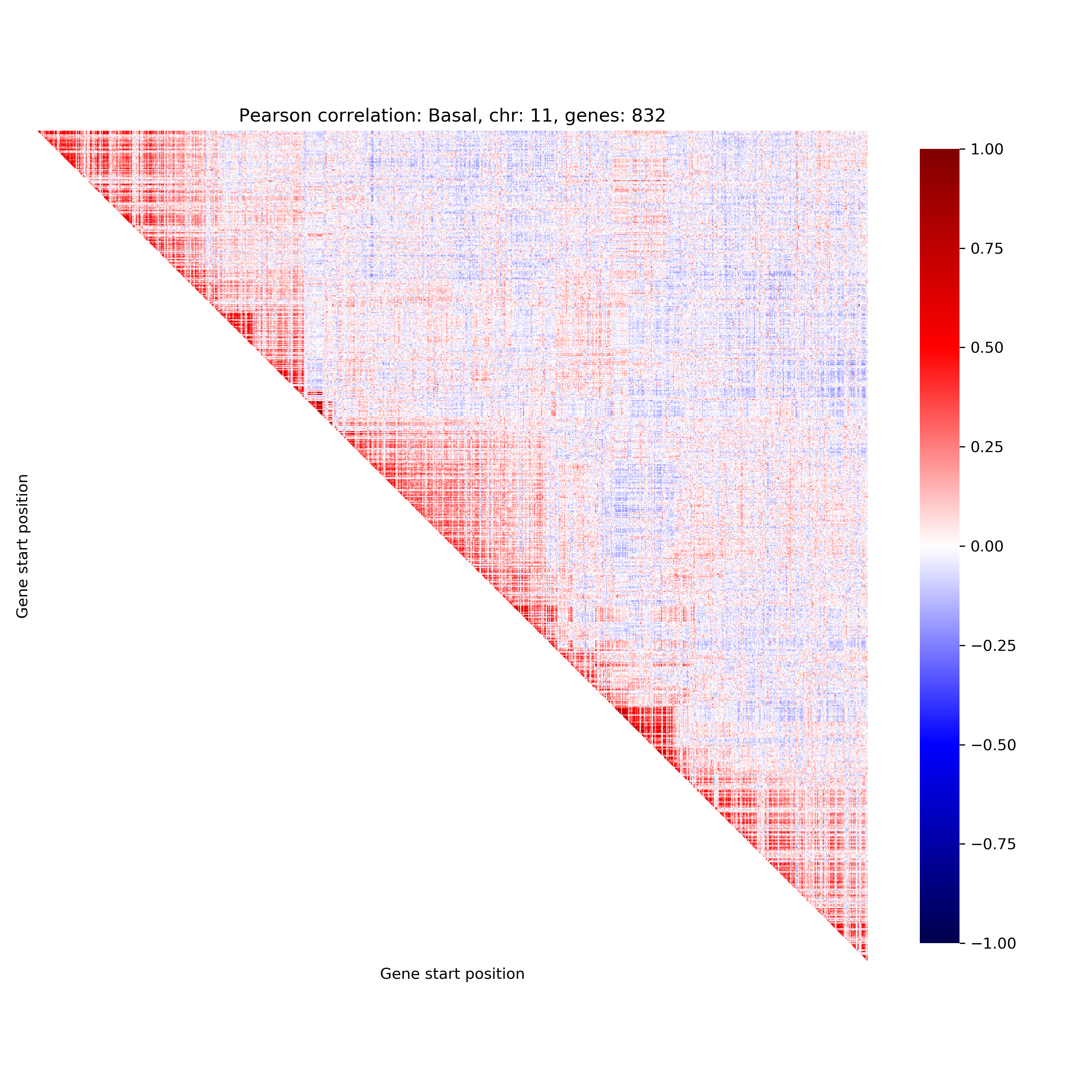

Supplement: Supplementary Material S5 — Heatmaps of Pearson correlation for each chromosome in the HER2+ phenotype. [file DataSheet_5.zip › SuppMat6/Basal-chr11.png]

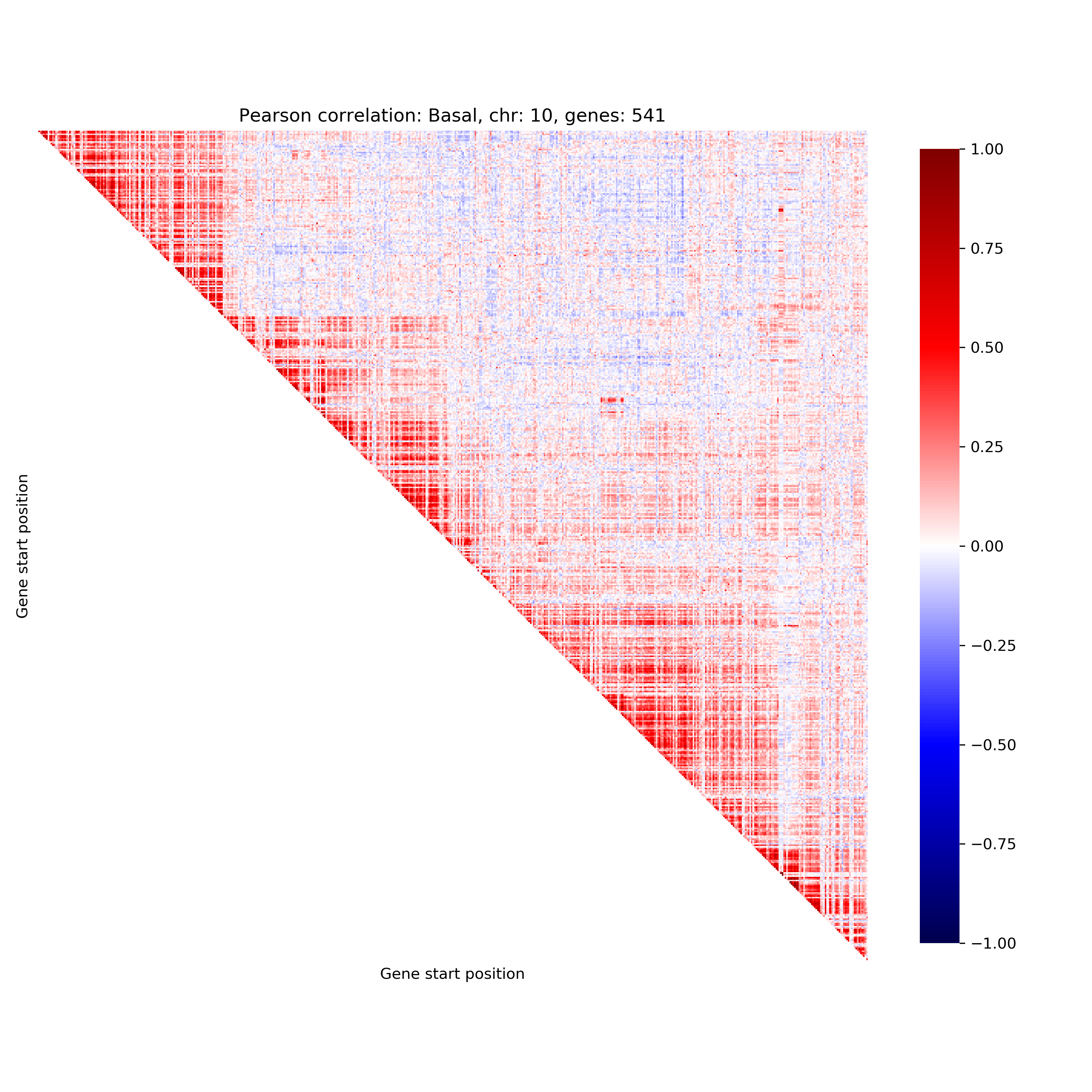

Supplement: Supplementary Material S5 — Heatmaps of Pearson correlation for each chromosome in the HER2+ phenotype. [file DataSheet_5.zip › SuppMat6/Basal-chr10.png]

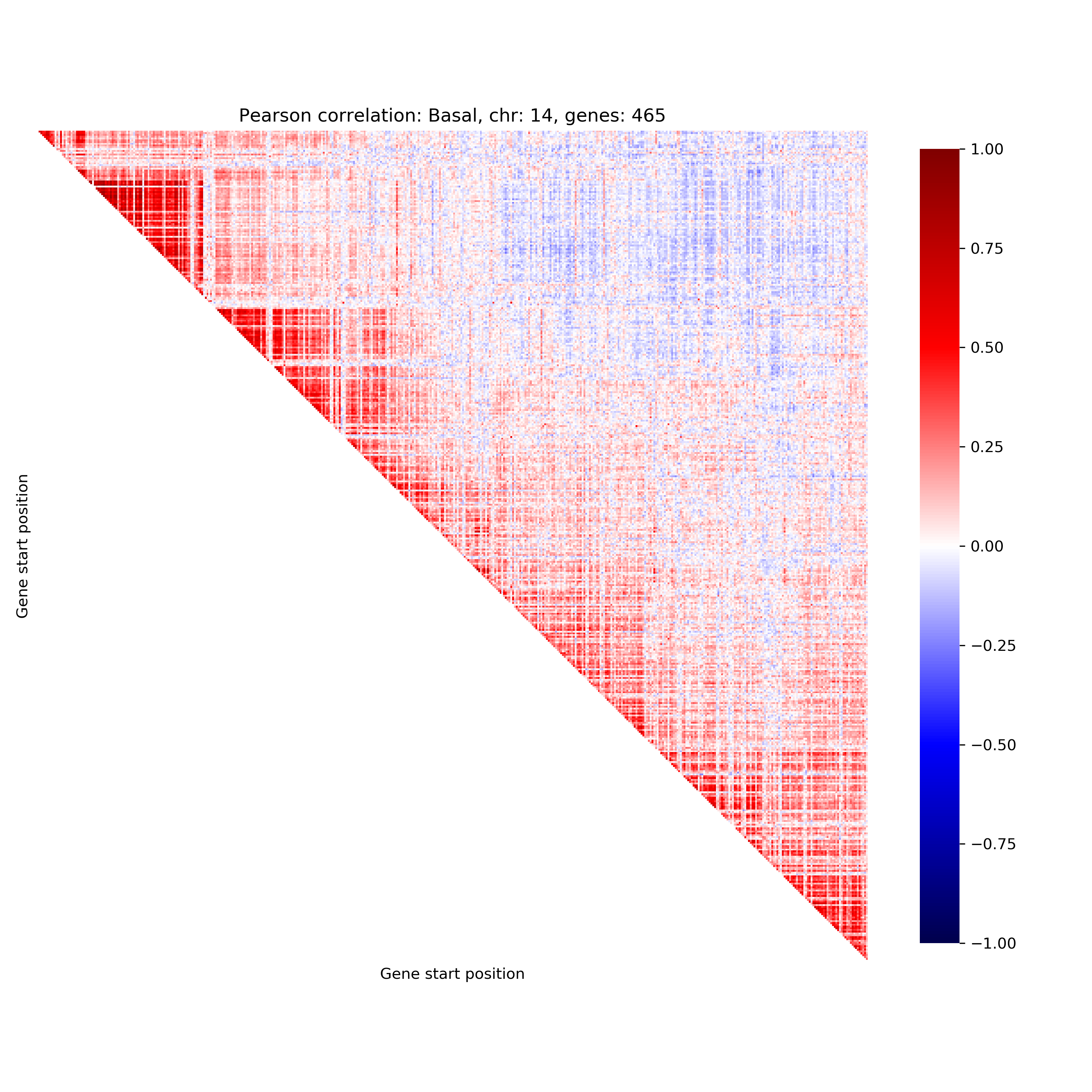

Supplement: Supplementary Material S5 — Heatmaps of Pearson correlation for each chromosome in the HER2+ phenotype. [file DataSheet_5.zip › SuppMat6/Basal-chr14.png]

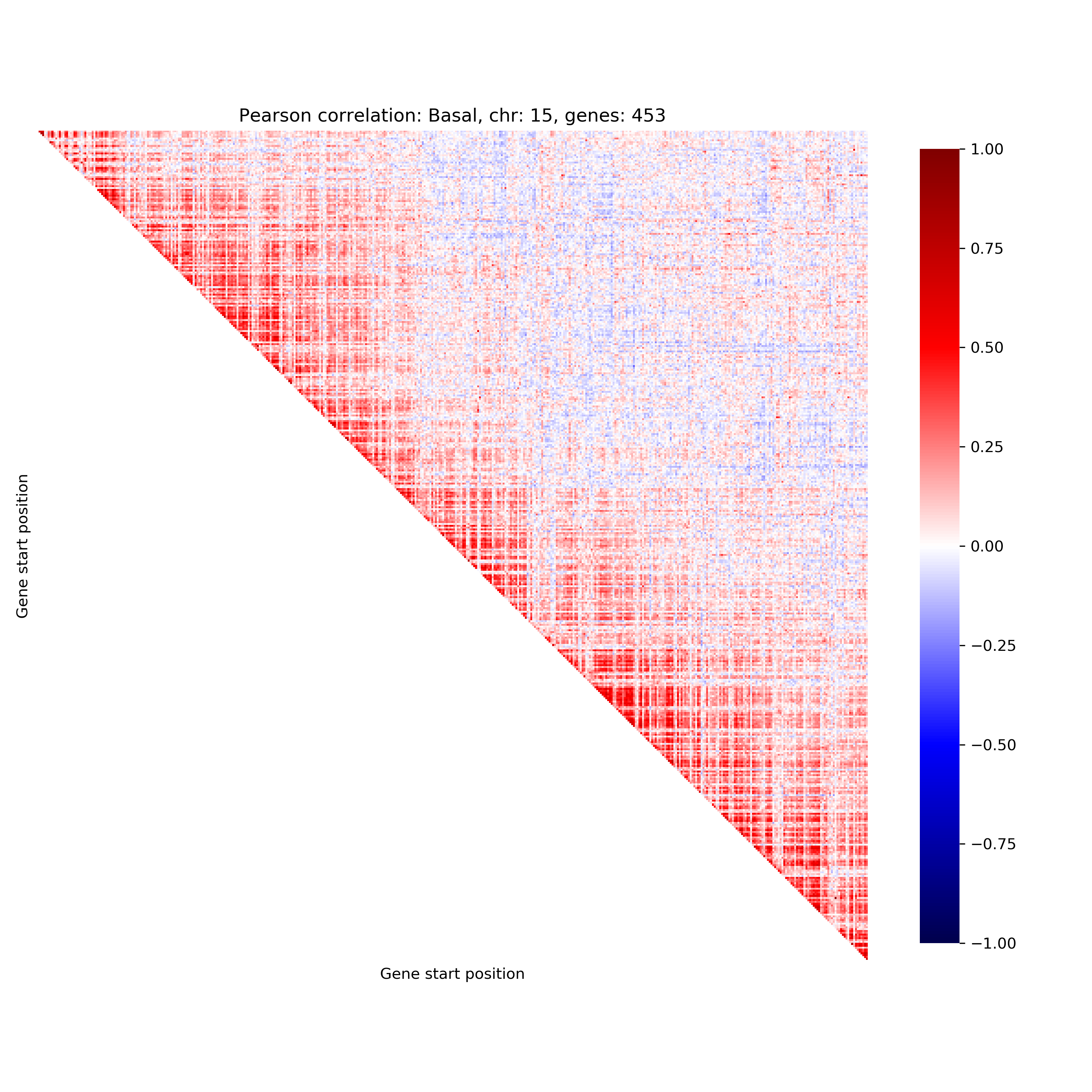

Supplement: Supplementary Material S5 — Heatmaps of Pearson correlation for each chromosome in the HER2+ phenotype. [file DataSheet_5.zip › SuppMat6/Basal-chr15.png]

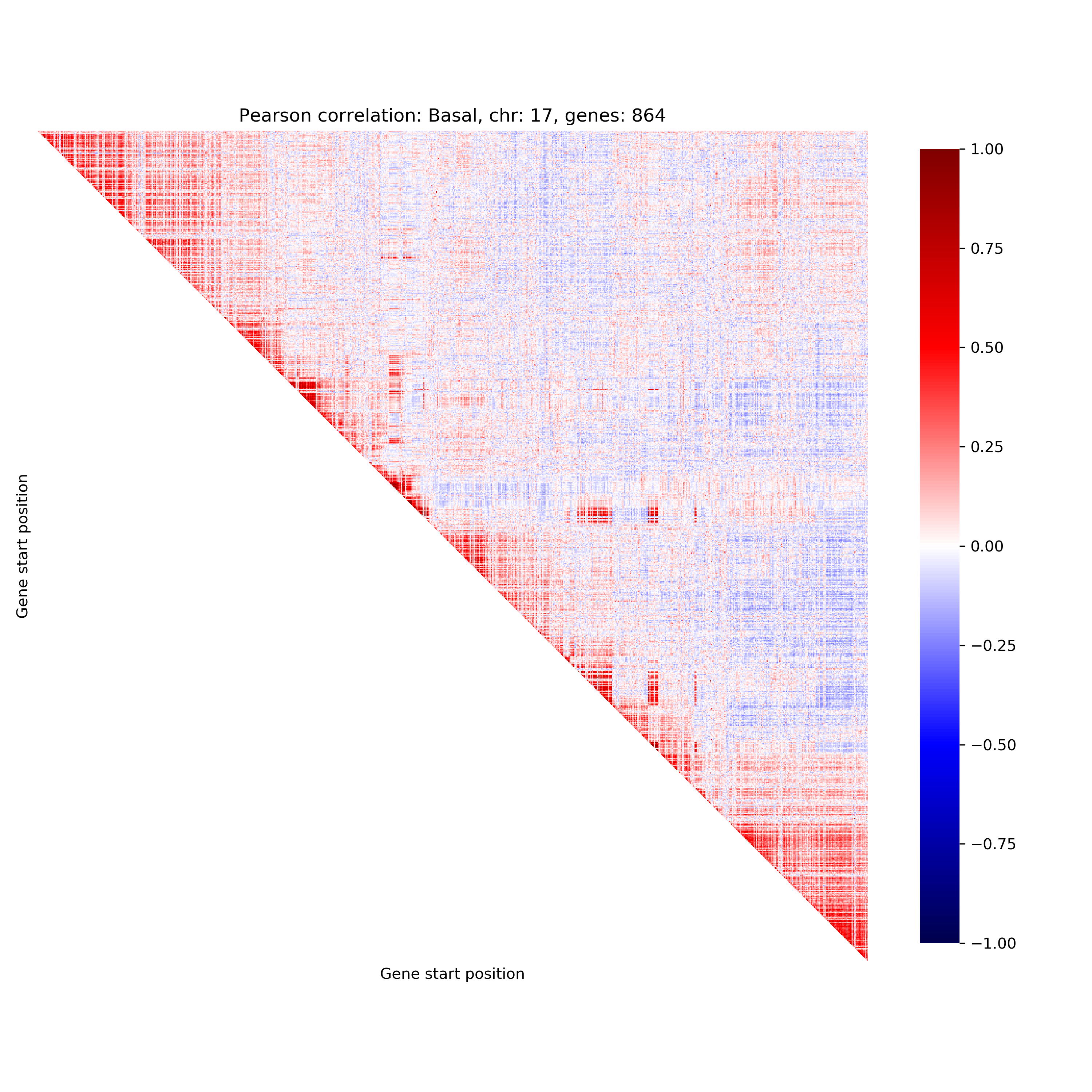

Supplement: Supplementary Material S5 — Heatmaps of Pearson correlation for each chromosome in the HER2+ phenotype. [file DataSheet_5.zip › SuppMat6/Basal-chr17.png]

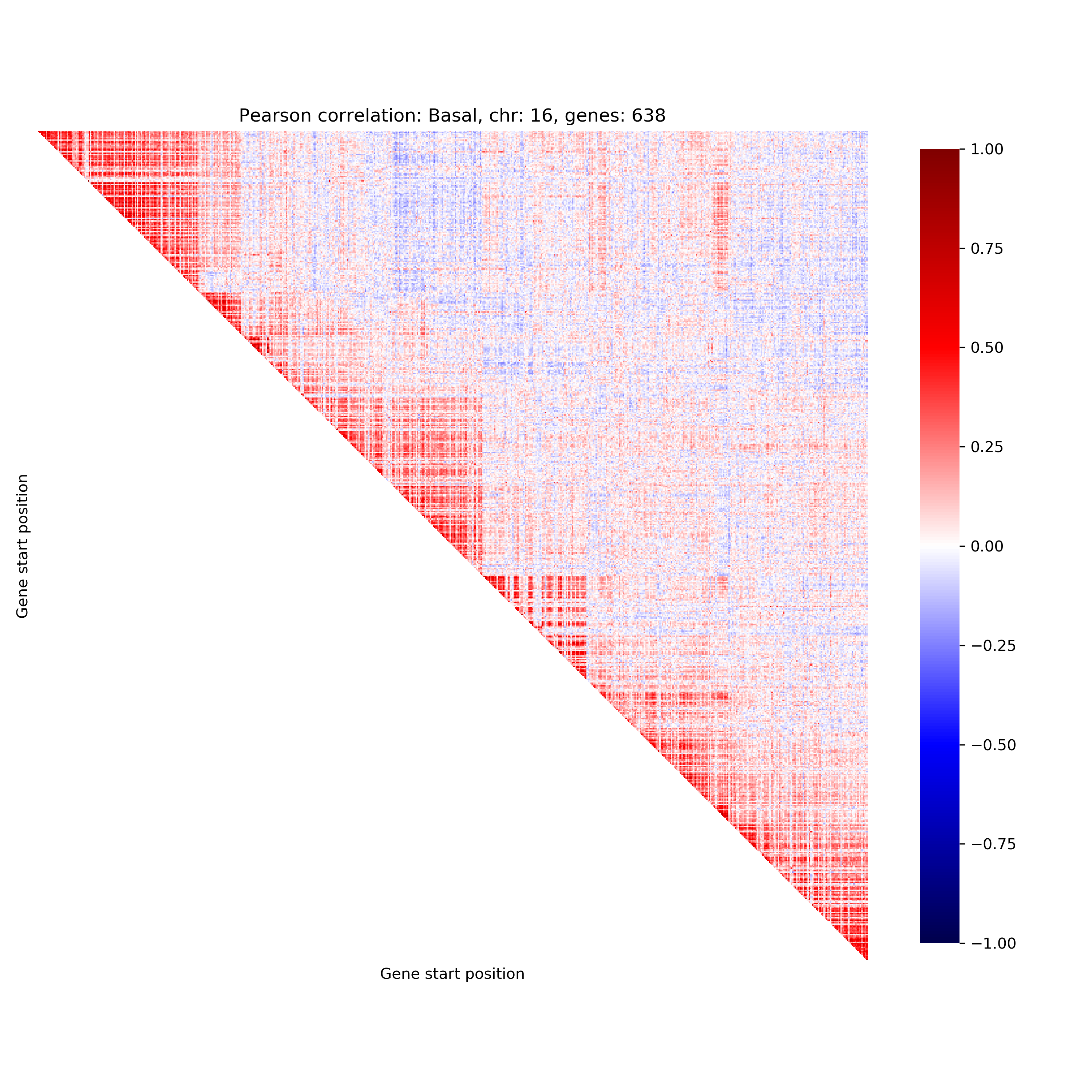

Supplement: Supplementary Material S5 — Heatmaps of Pearson correlation for each chromosome in the HER2+ phenotype. [file DataSheet_5.zip › SuppMat6/Basal-chr16.png]

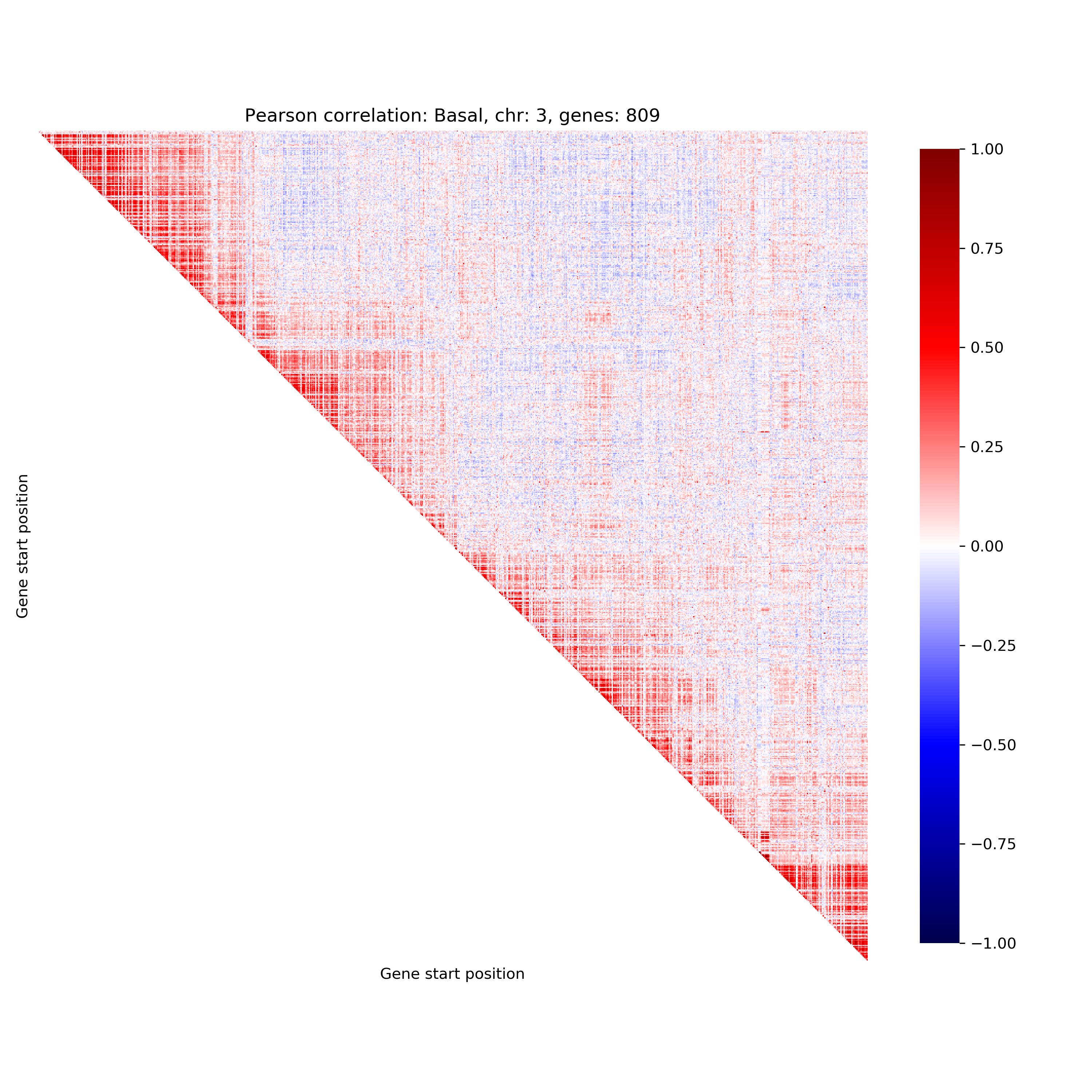

Supplement: Supplementary Material S5 — Heatmaps of Pearson correlation for each chromosome in the HER2+ phenotype. [file DataSheet_5.zip › SuppMat6/Basal-chr3.png]

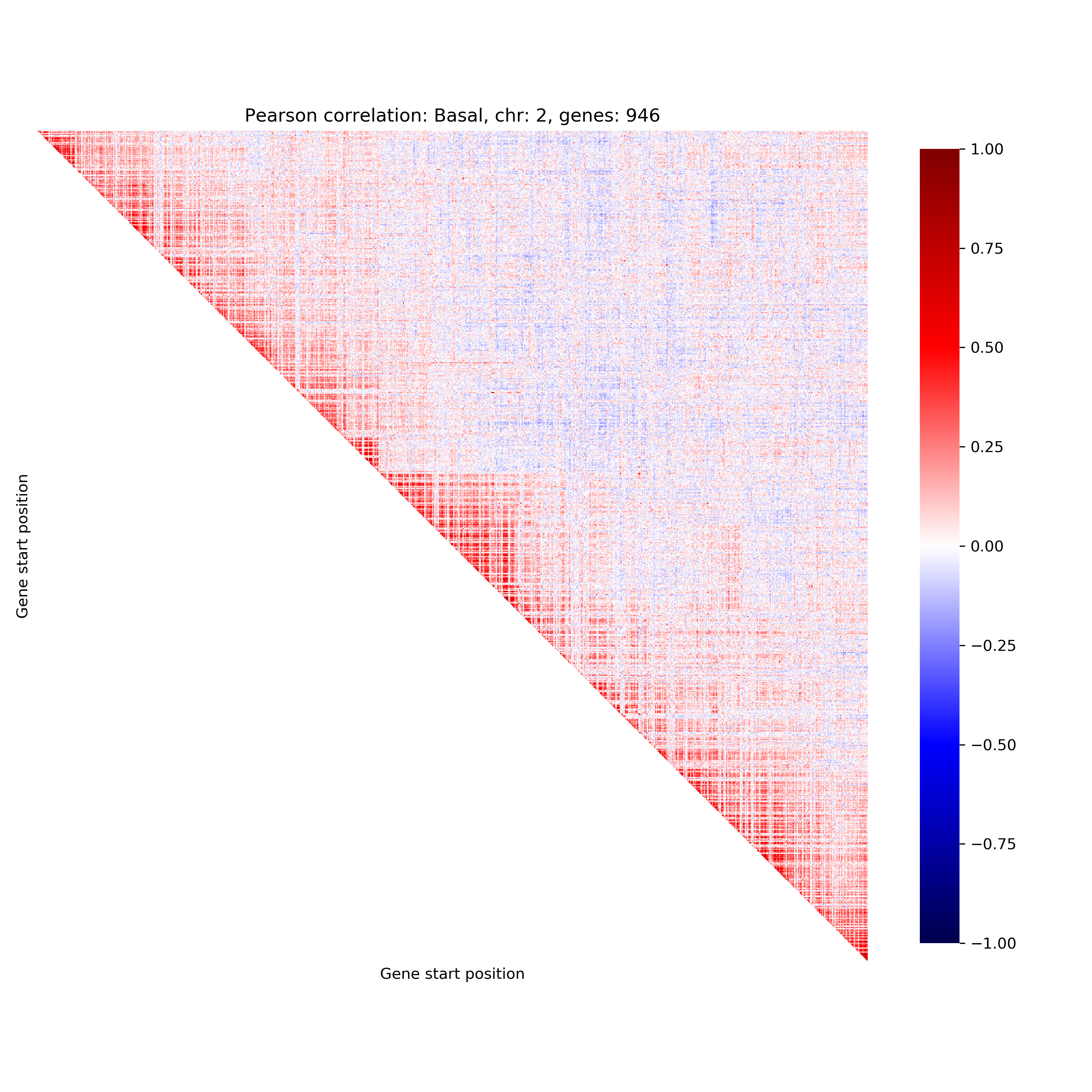

Supplement: Supplementary Material S5 — Heatmaps of Pearson correlation for each chromosome in the HER2+ phenotype. [file DataSheet_5.zip › SuppMat6/Basal-chr2.png]

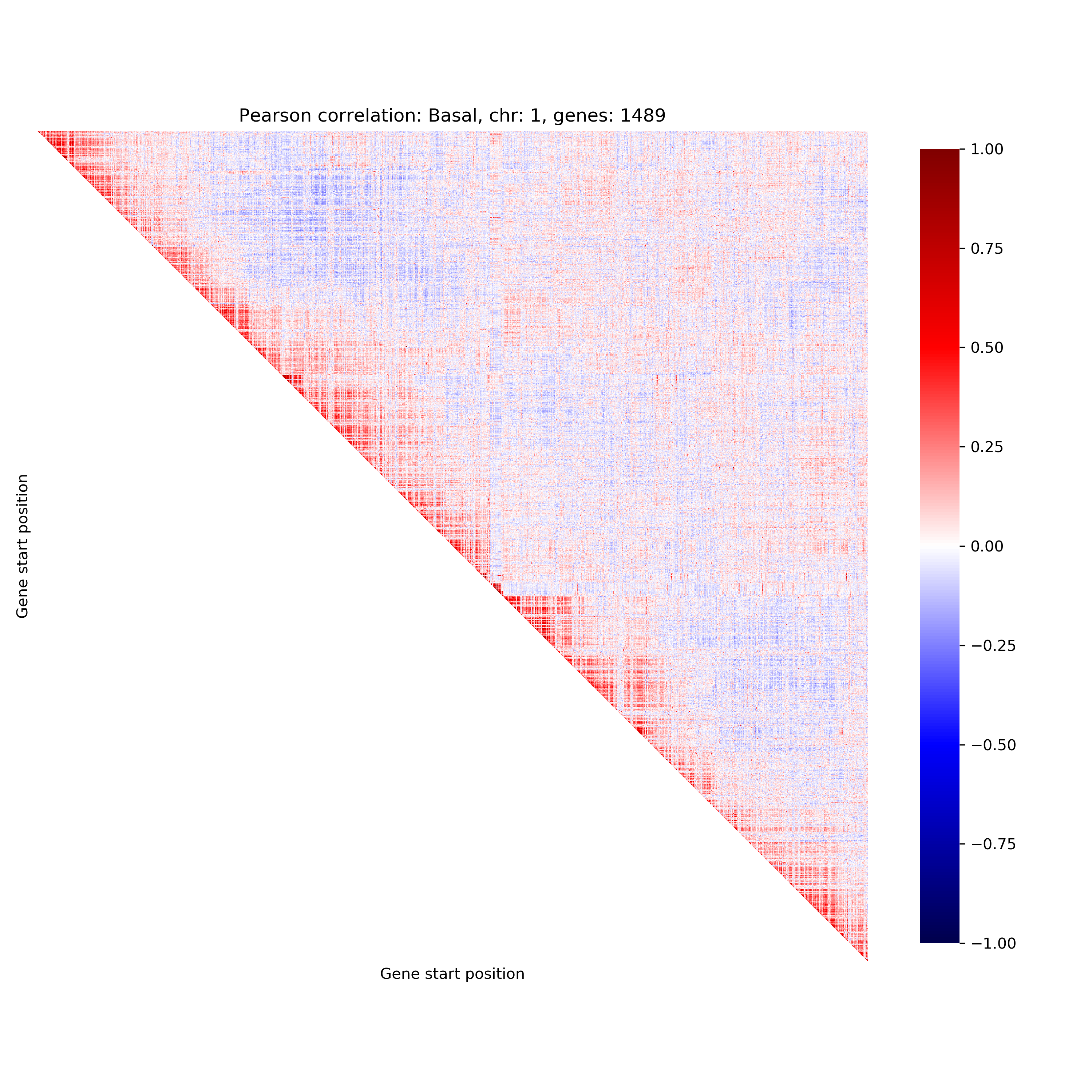

Supplement: Supplementary Material S5 — Heatmaps of Pearson correlation for each chromosome in the HER2+ phenotype. [file DataSheet_5.zip › SuppMat6/Basal-chr1.png]

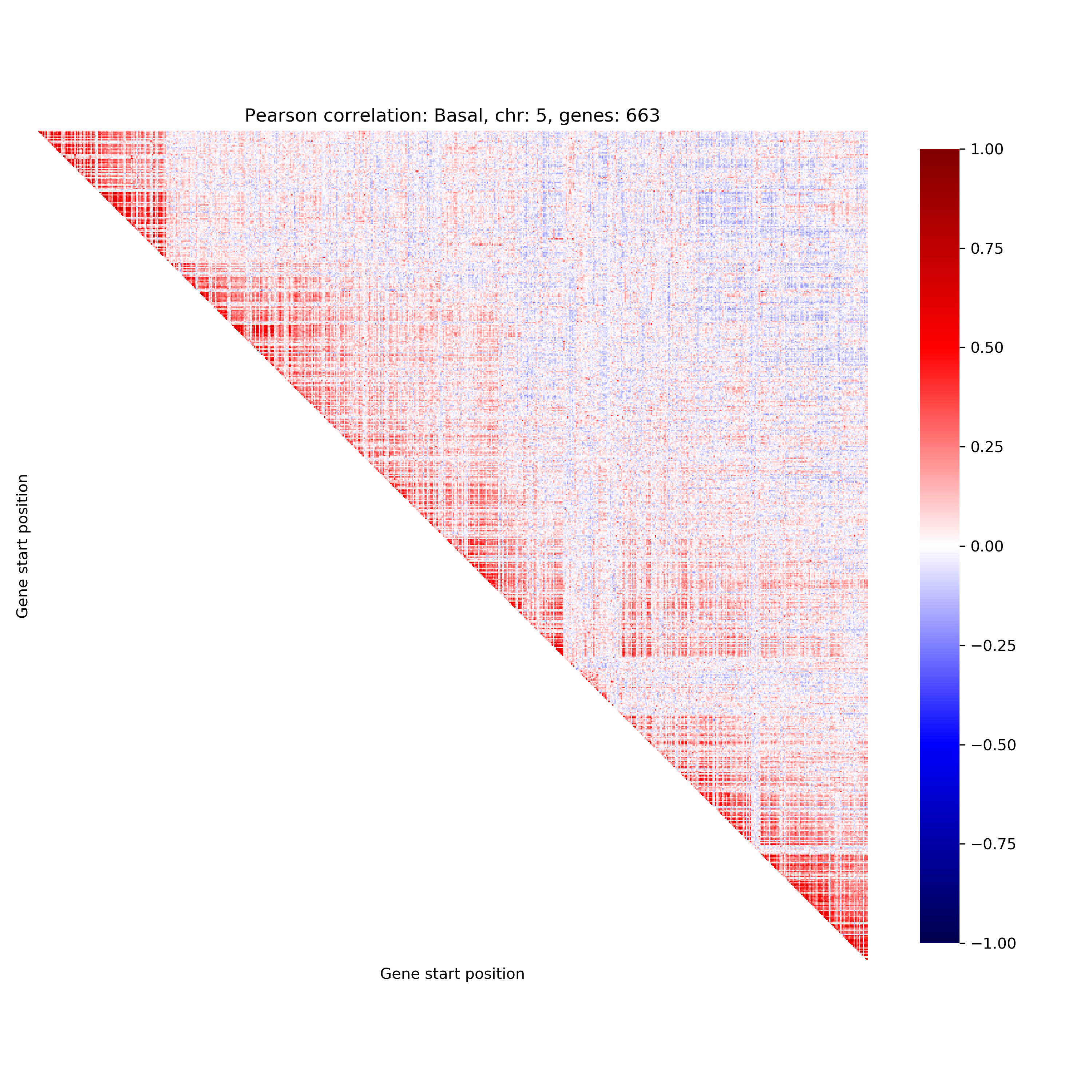

Supplement: Supplementary Material S5 — Heatmaps of Pearson correlation for each chromosome in the HER2+ phenotype. [file DataSheet_5.zip › SuppMat6/Basal-chr5.png]

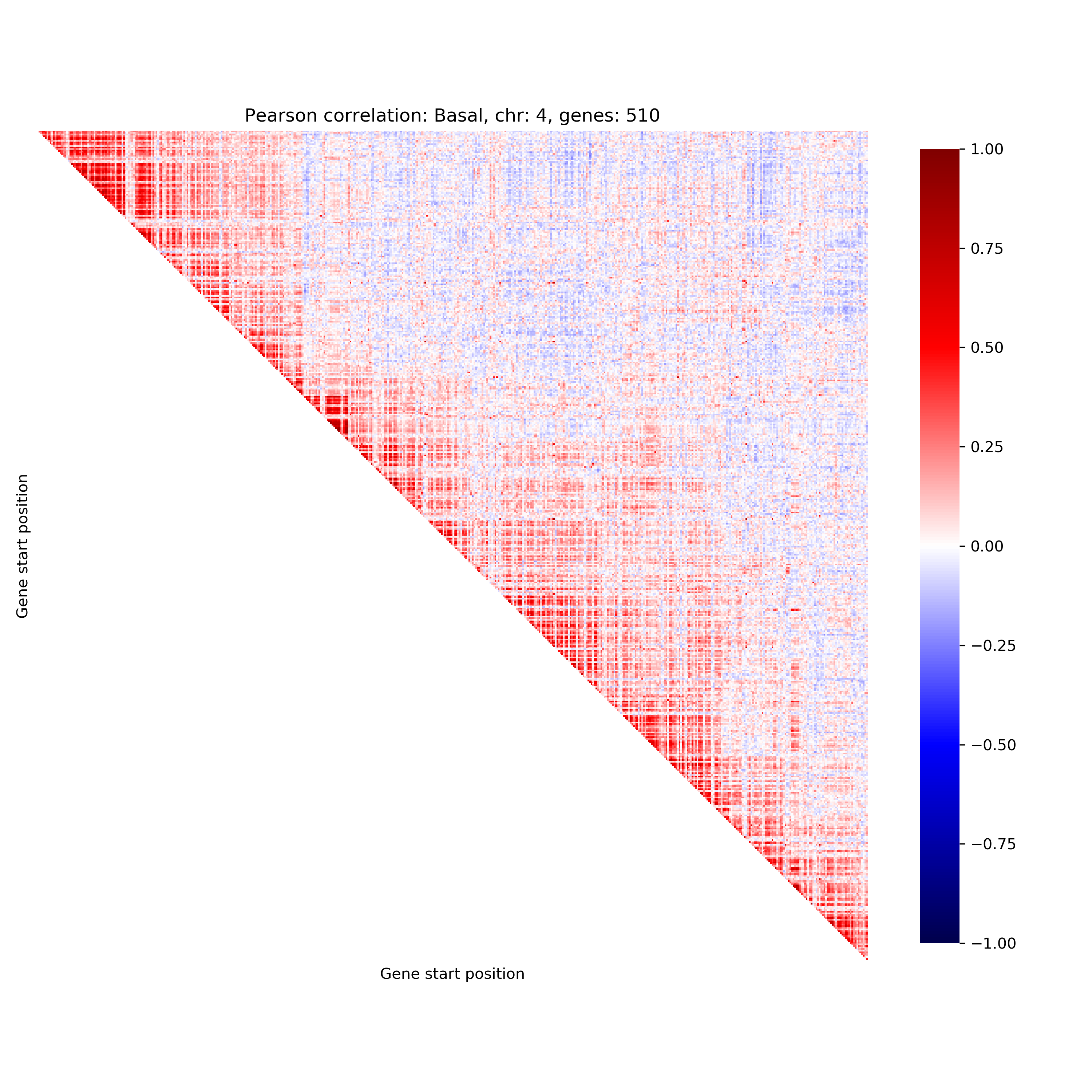

Supplement: Supplementary Material S5 — Heatmaps of Pearson correlation for each chromosome in the HER2+ phenotype. [file DataSheet_5.zip › SuppMat6/Basal-chr4.png]

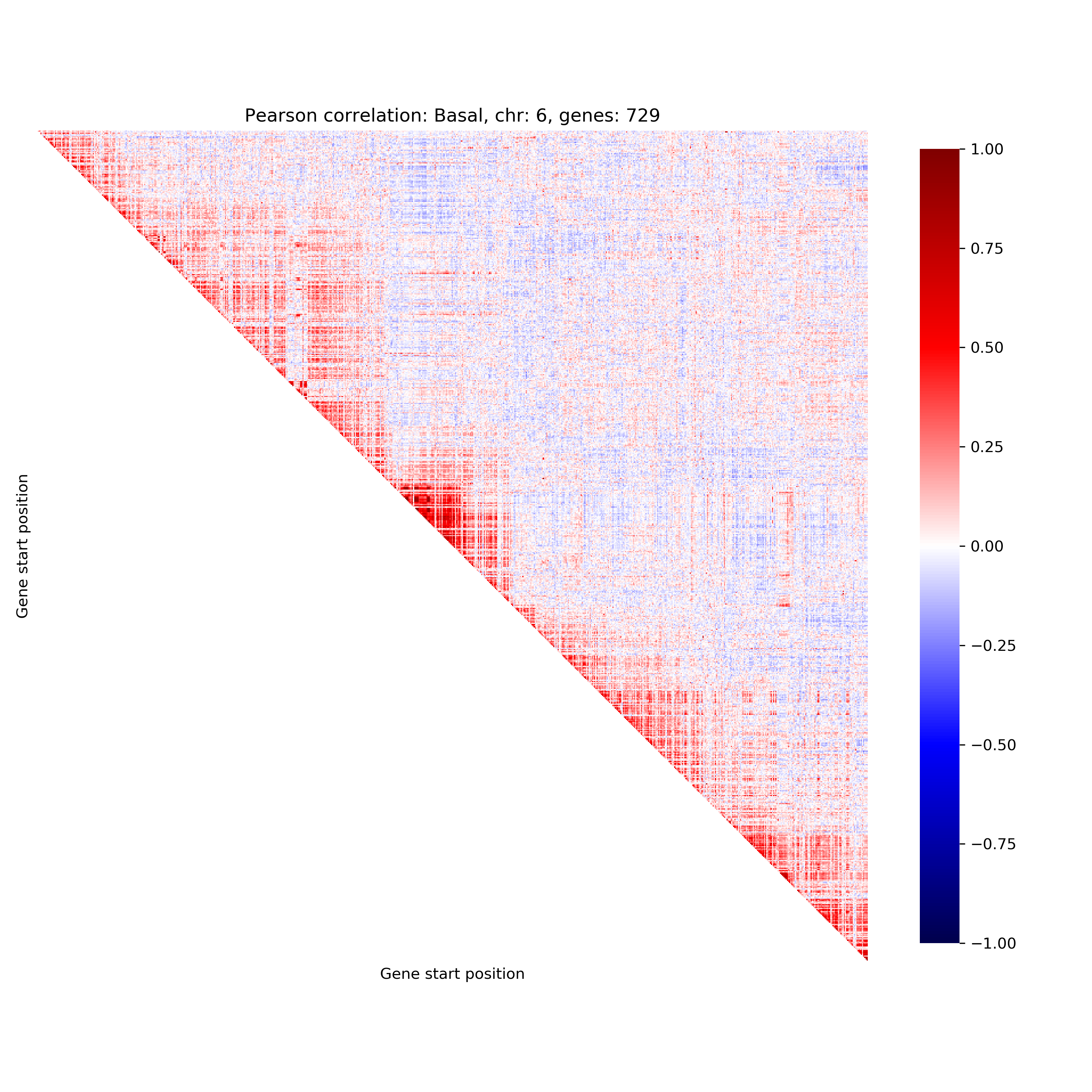

Supplement: Supplementary Material S5 — Heatmaps of Pearson correlation for each chromosome in the HER2+ phenotype. [file DataSheet_5.zip › SuppMat6/Basal-chr6.png]

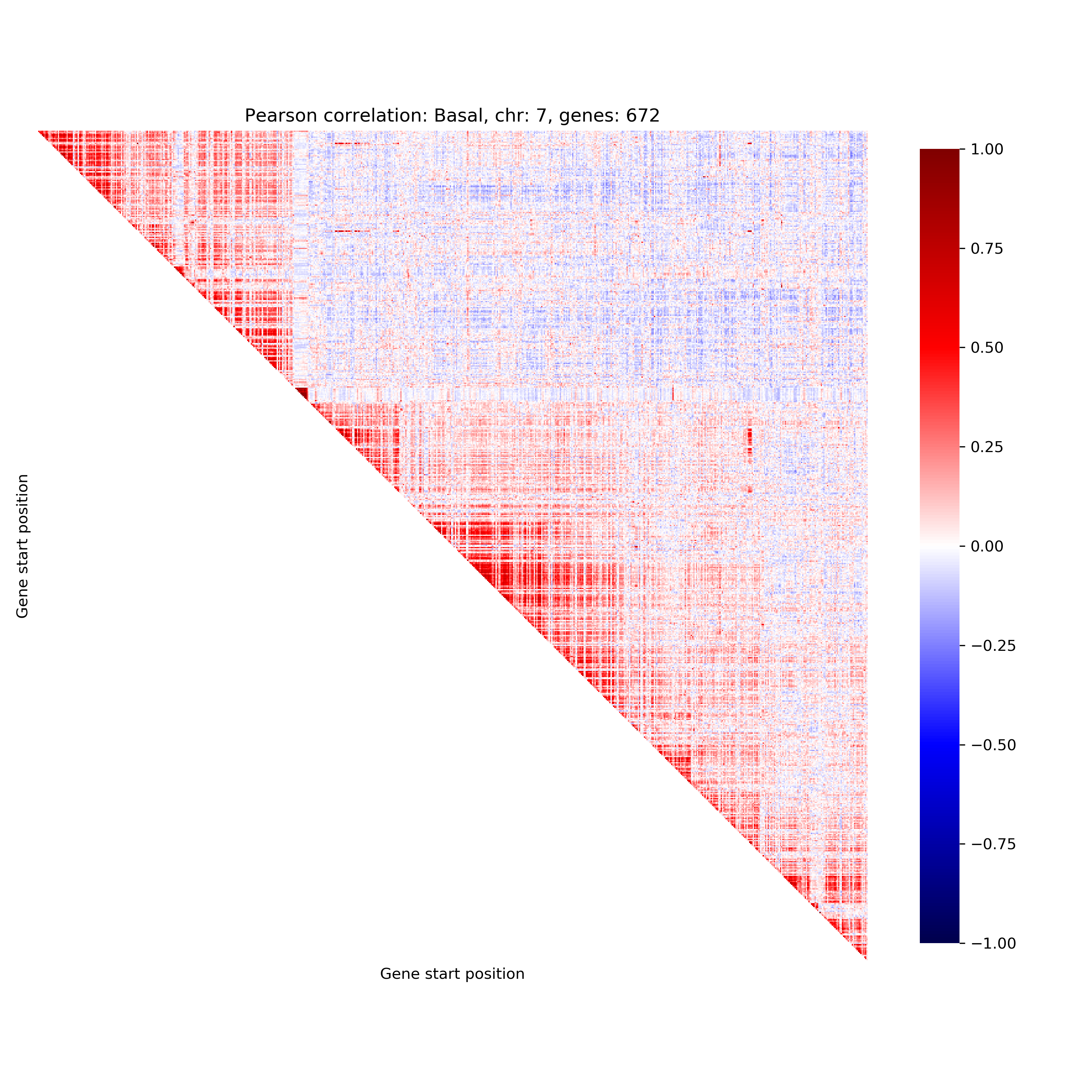

Supplement: Supplementary Material S5 — Heatmaps of Pearson correlation for each chromosome in the HER2+ phenotype. [file DataSheet_5.zip › SuppMat6/Basal-chr7.png]

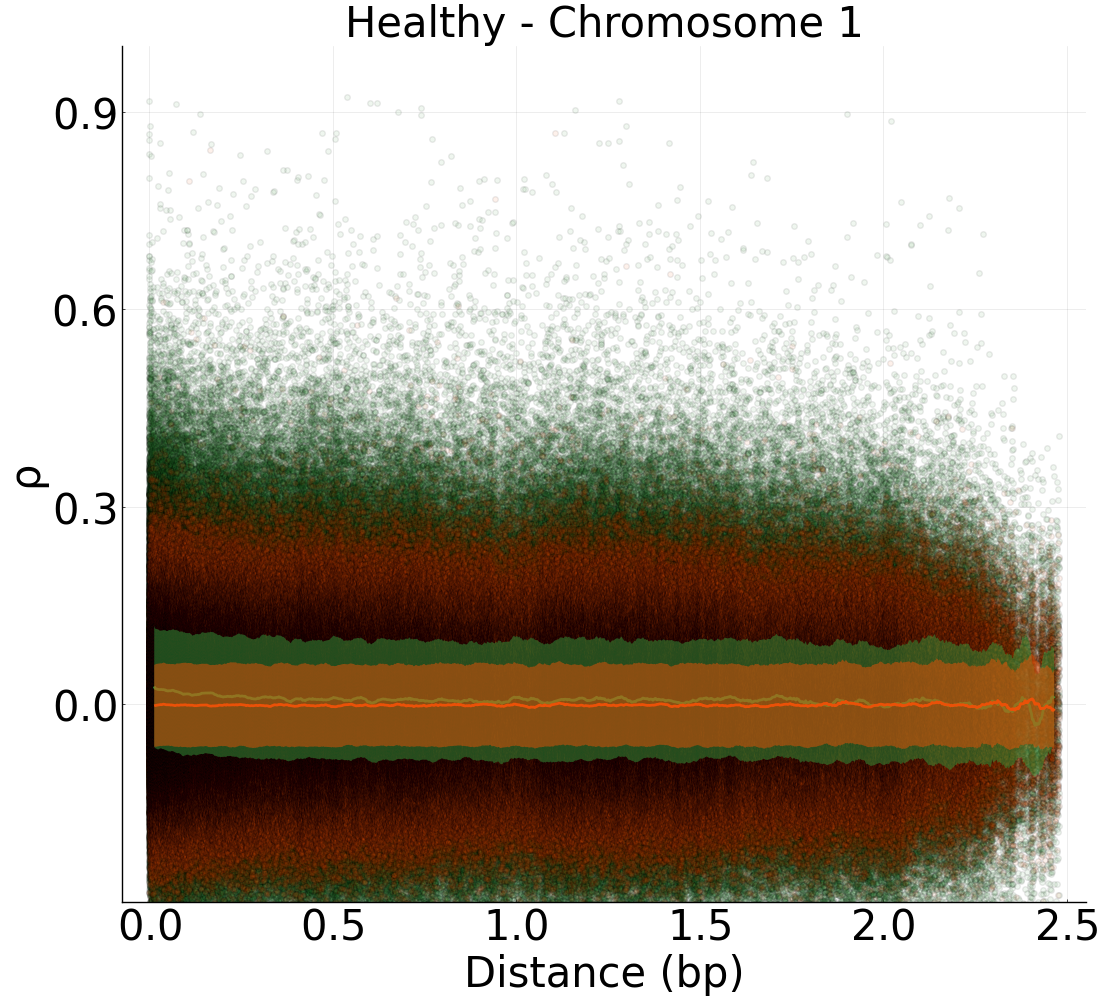

Supplement: Supplementary Material S6 — Heatmaps of Pearson correlation for each chromosome in the Basal phenotype. [file DataSheet_6.zip › SuppMat7Ctrl/Chromosome-1-Healthy.png]

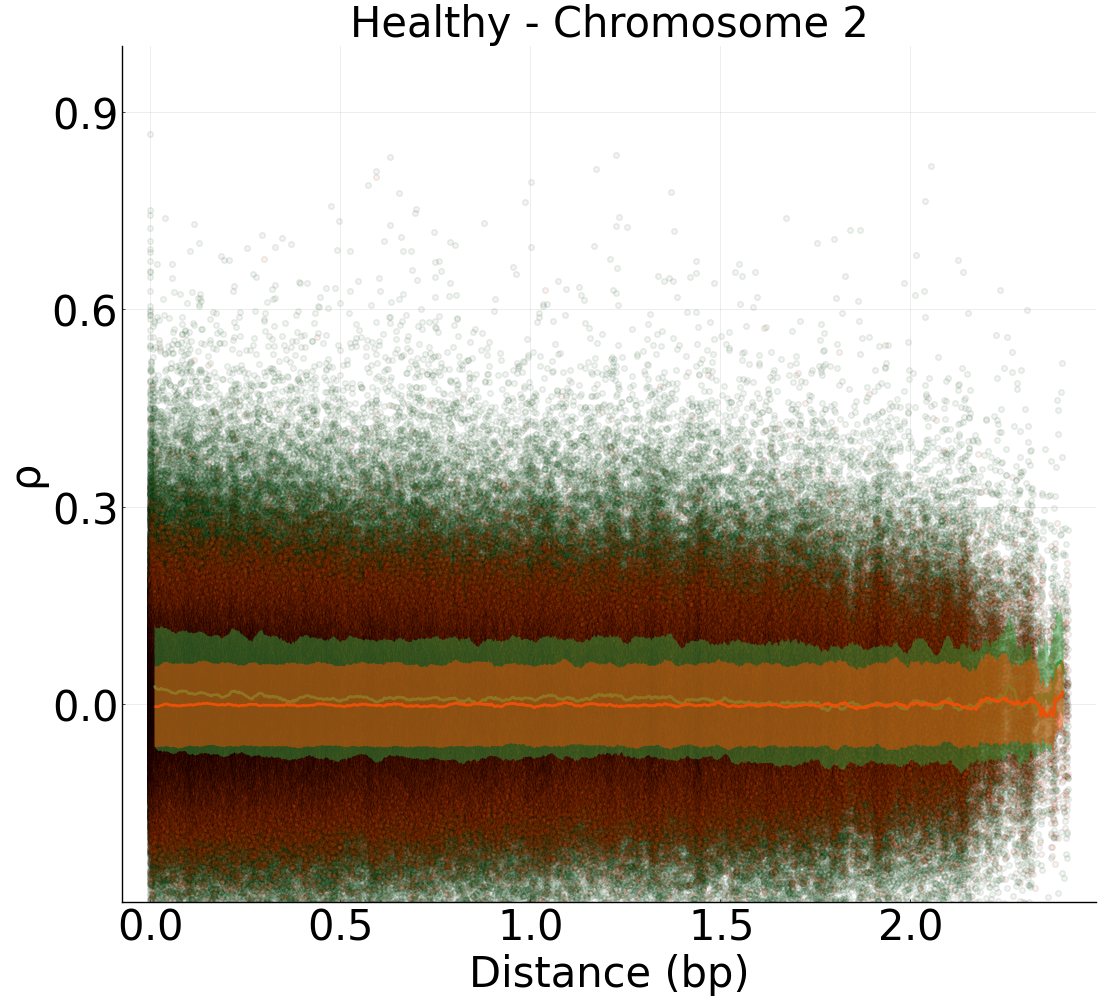

Supplement: Supplementary Material S6 — Heatmaps of Pearson correlation for each chromosome in the Basal phenotype. [file DataSheet_6.zip › SuppMat7Ctrl/Chromosome-2-Healthy.png]

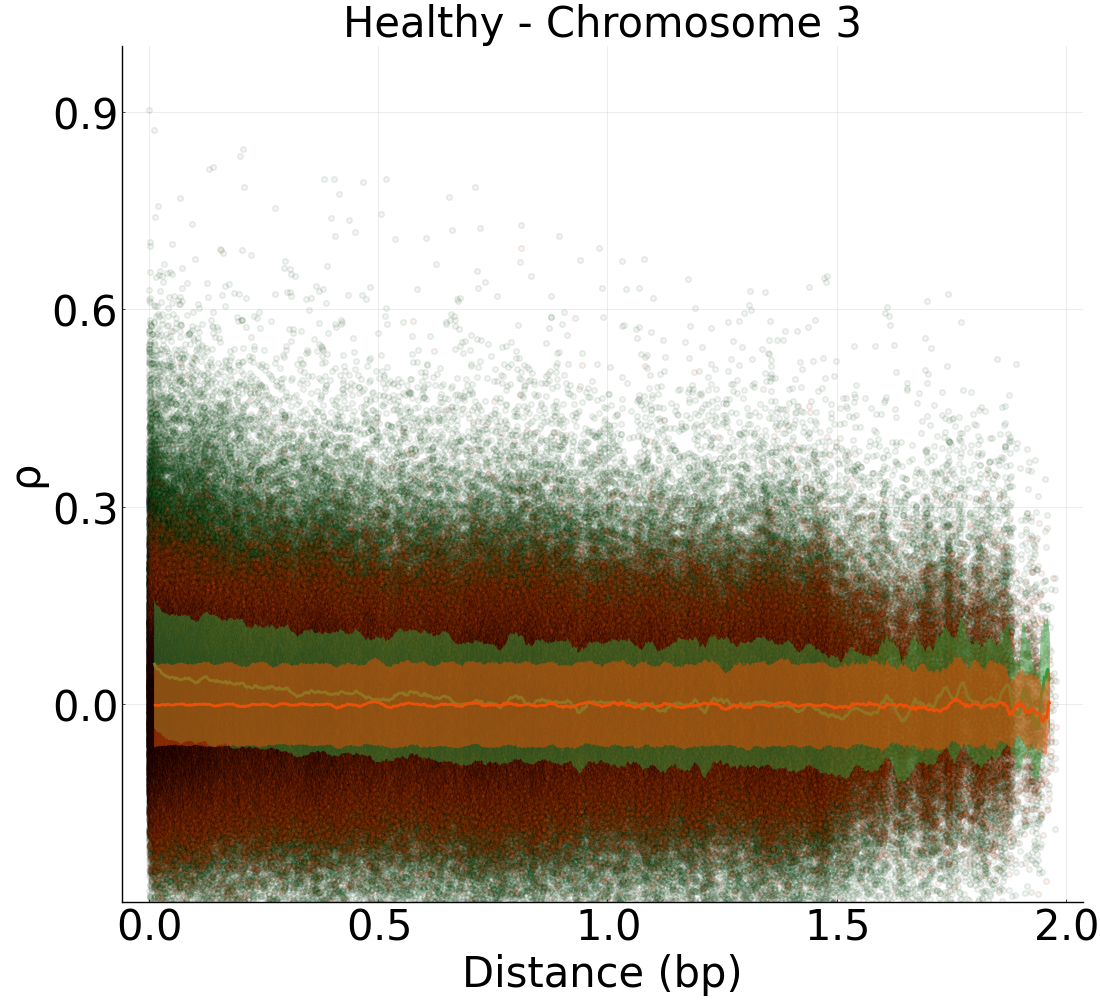

Supplement: Supplementary Material S6 — Heatmaps of Pearson correlation for each chromosome in the Basal phenotype. [file DataSheet_6.zip › SuppMat7Ctrl/Chromosome-3-Healthy.png]

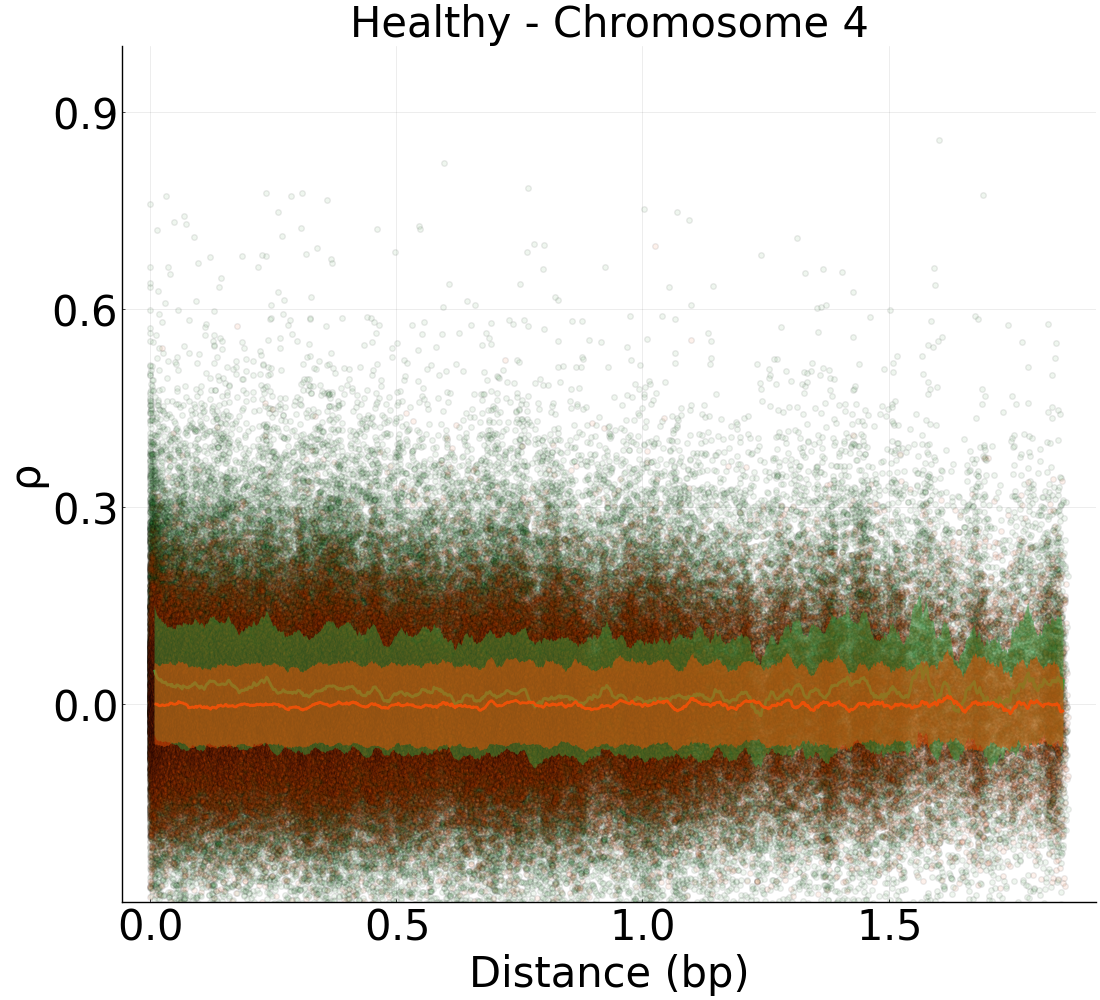

Supplement: Supplementary Material S6 — Heatmaps of Pearson correlation for each chromosome in the Basal phenotype. [file DataSheet_6.zip › SuppMat7Ctrl/Chromosome-4-Healthy.png]

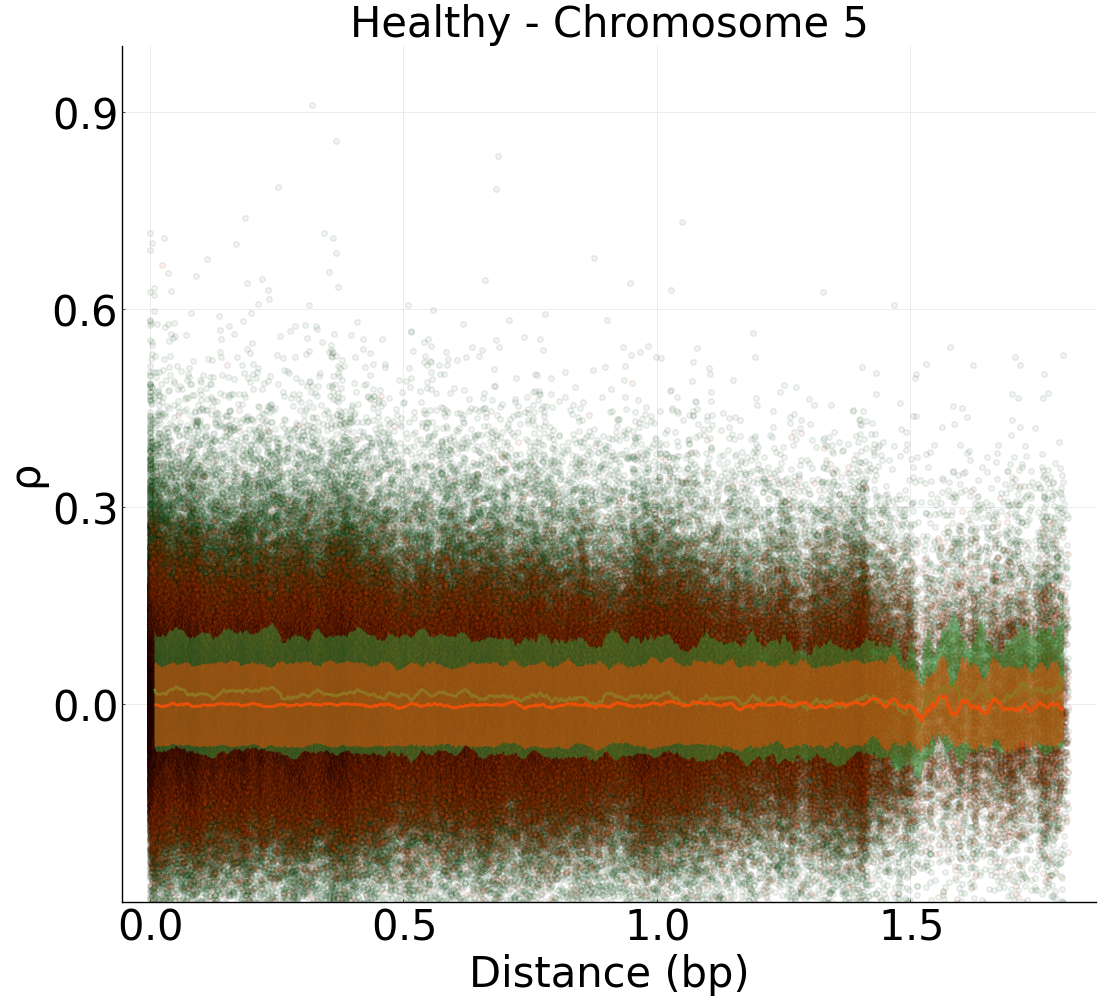

Supplement: Supplementary Material S6 — Heatmaps of Pearson correlation for each chromosome in the Basal phenotype. [file DataSheet_6.zip › SuppMat7Ctrl/Chromosome-5-Healthy.png]

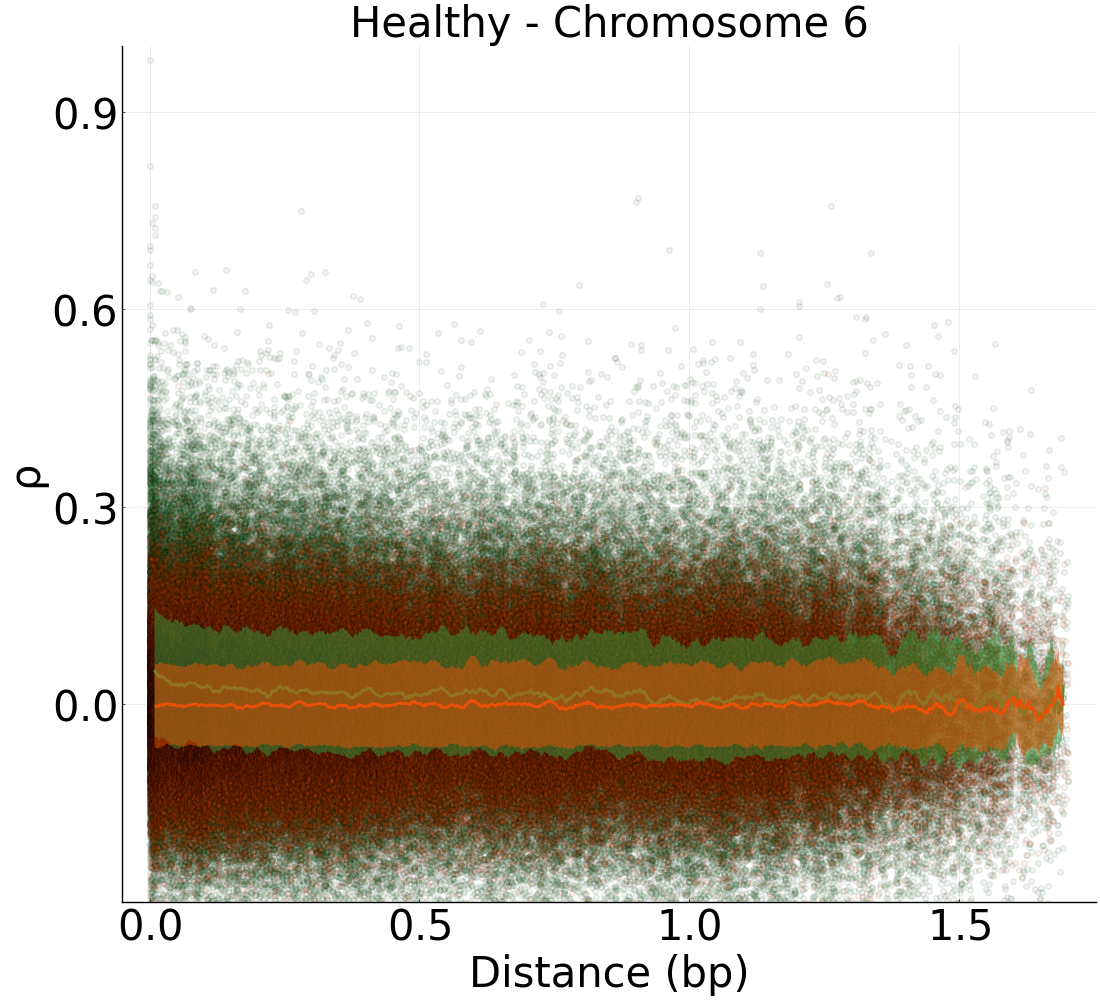

Supplement: Supplementary Material S6 — Heatmaps of Pearson correlation for each chromosome in the Basal phenotype. [file DataSheet_6.zip › SuppMat7Ctrl/Chromosome-6-Healthy.png]

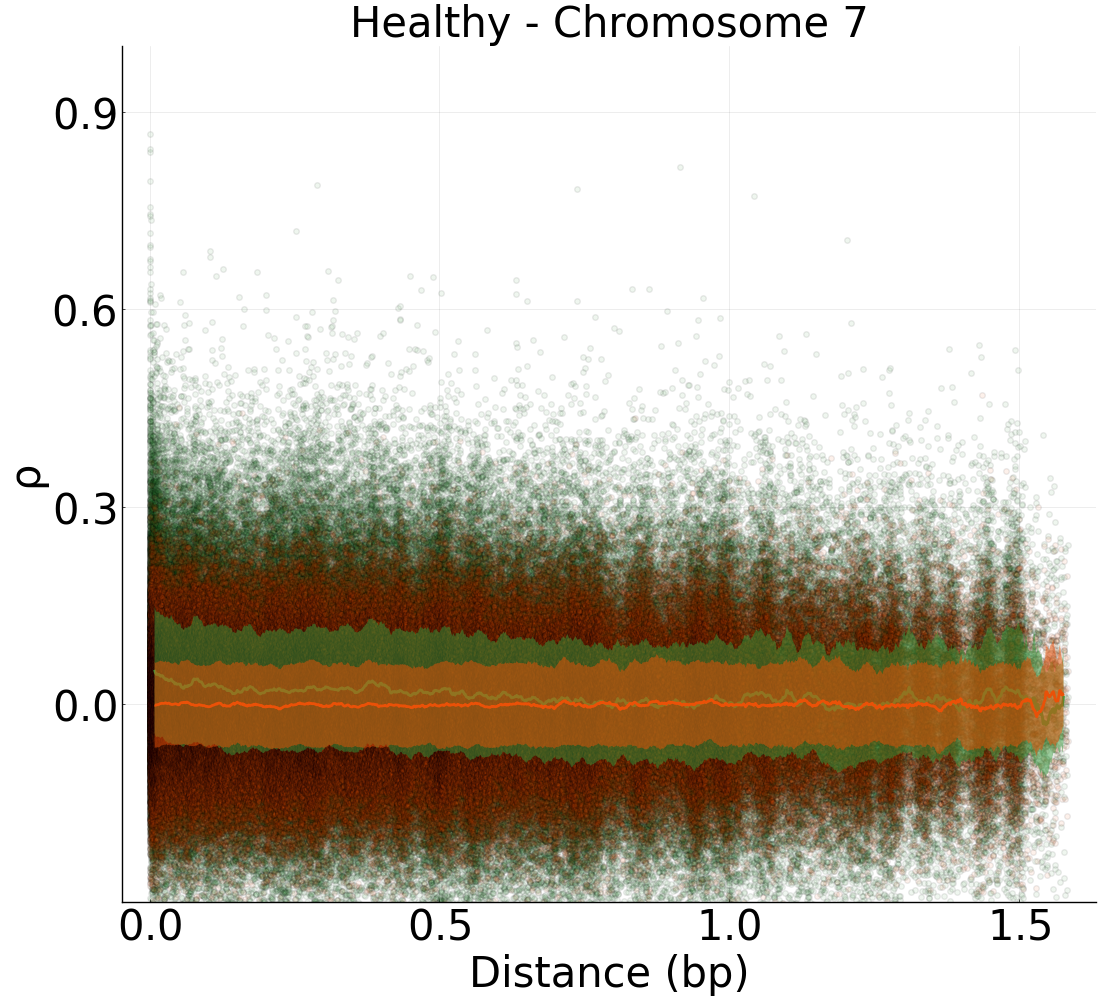

Supplement: Supplementary Material S6 — Heatmaps of Pearson correlation for each chromosome in the Basal phenotype. [file DataSheet_6.zip › SuppMat7Ctrl/Chromosome-7-Healthy.png]

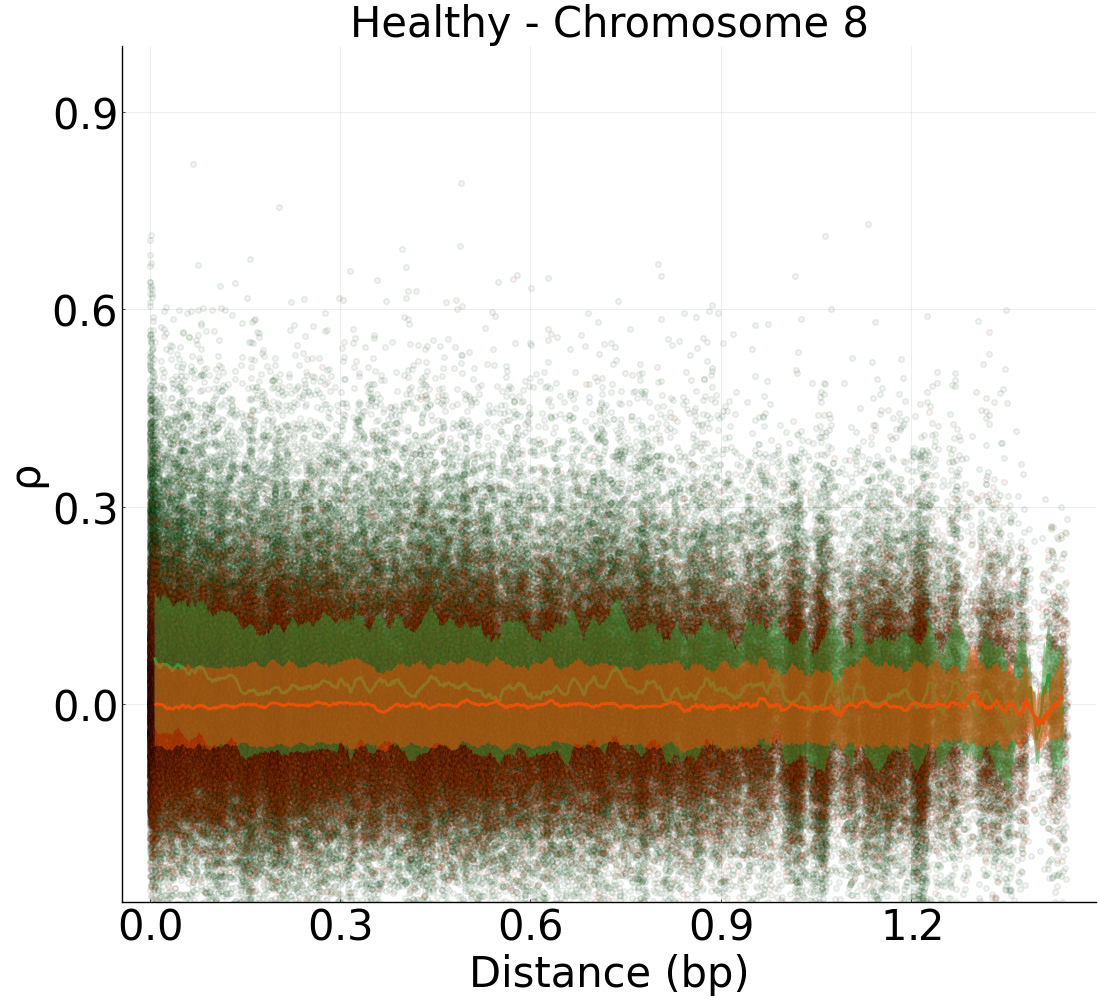

Supplement: Supplementary Material S6 — Heatmaps of Pearson correlation for each chromosome in the Basal phenotype. [file DataSheet_6.zip › SuppMat7Ctrl/Chromosome-8-Healthy.png]

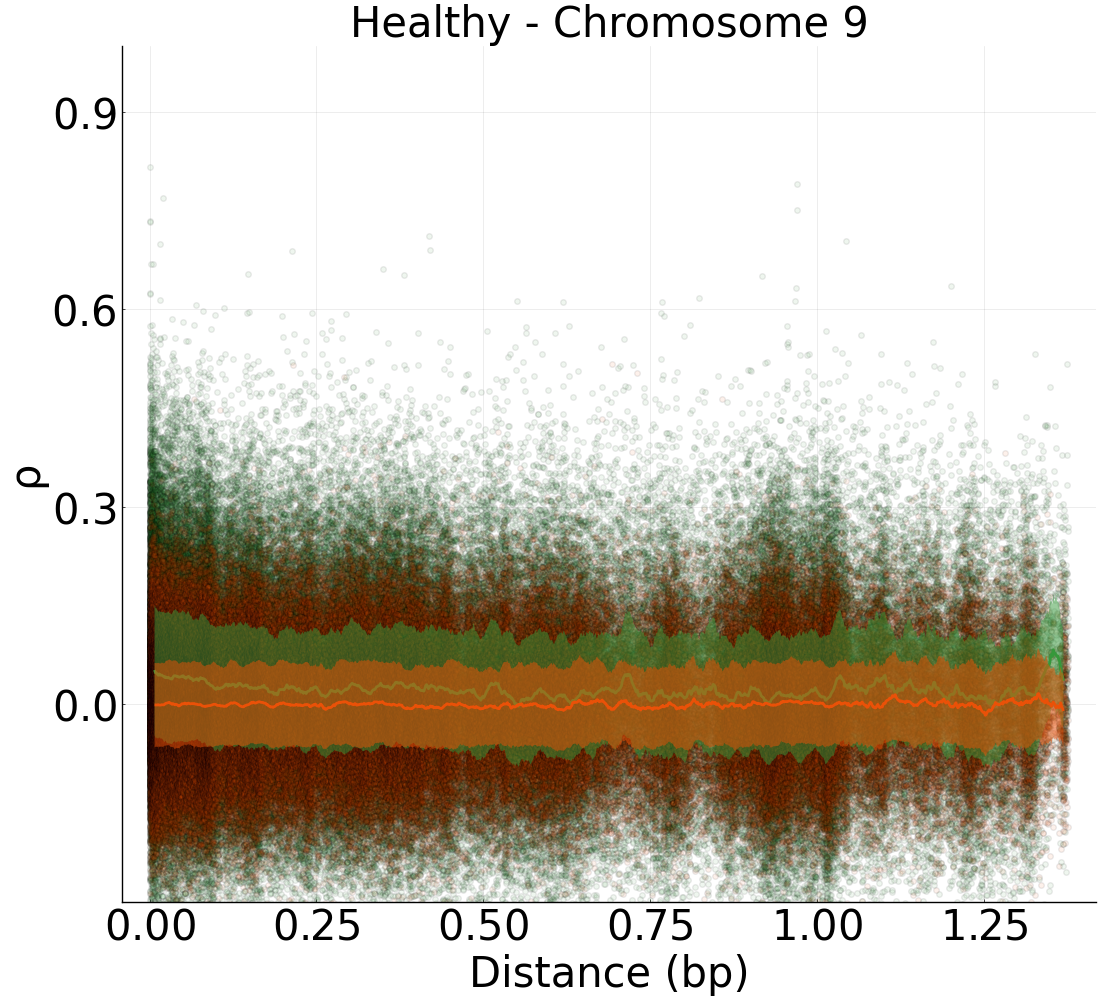

Supplement: Supplementary Material S6 — Heatmaps of Pearson correlation for each chromosome in the Basal phenotype. [file DataSheet_6.zip › SuppMat7Ctrl/Chromosome-9-Healthy.png]

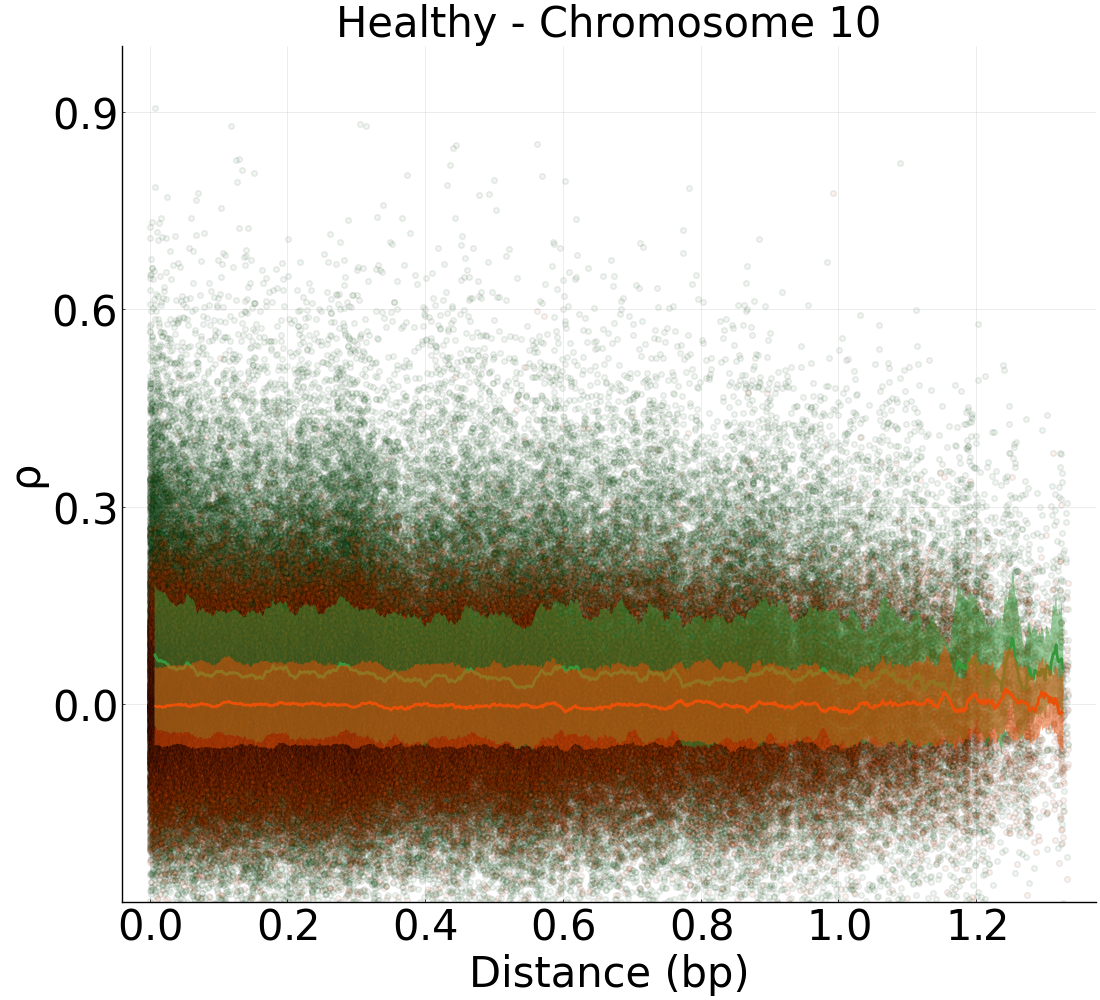

Supplement: Supplementary Material S6 — Heatmaps of Pearson correlation for each chromosome in the Basal phenotype. [file DataSheet_6.zip › SuppMat7Ctrl/Chromosome-10-Healthy.png]

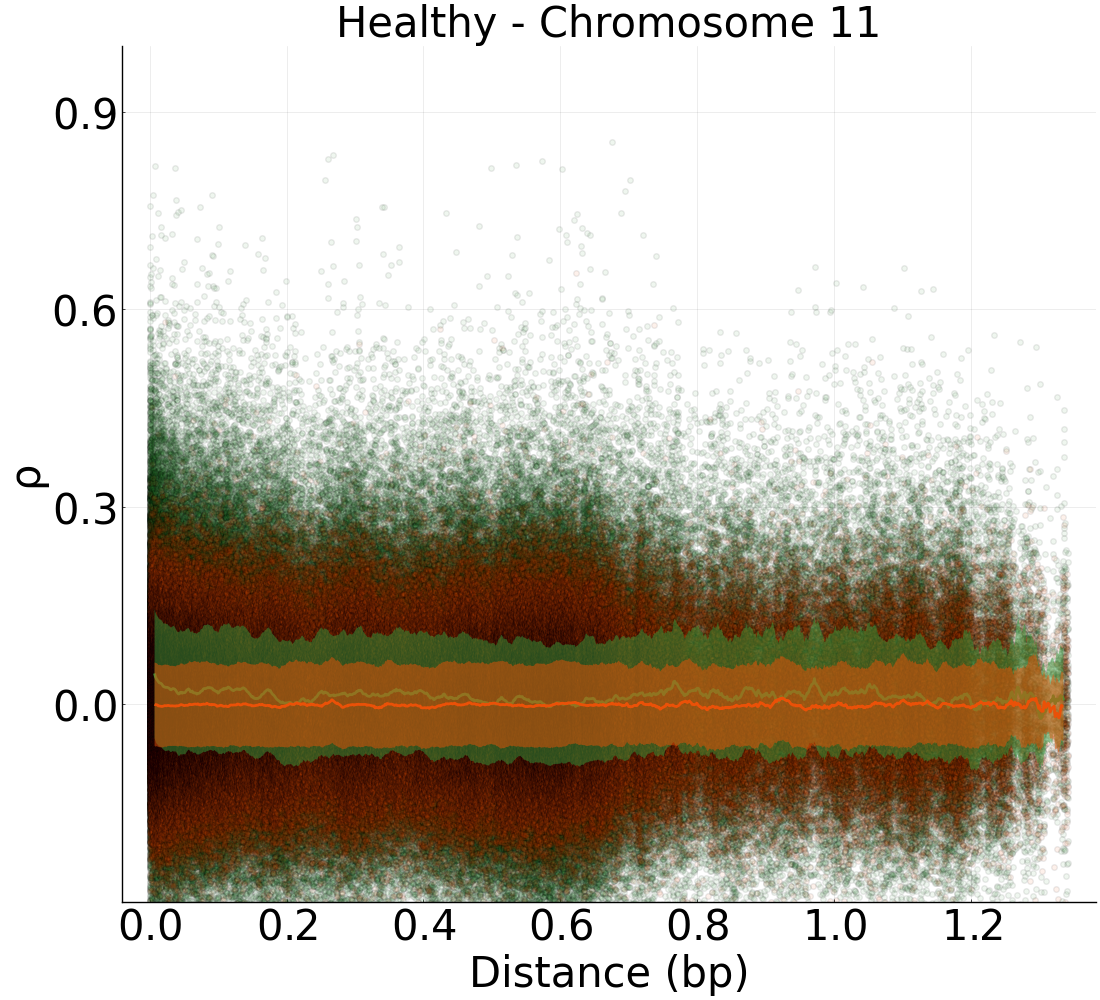

Supplement: Supplementary Material S6 — Heatmaps of Pearson correlation for each chromosome in the Basal phenotype. [file DataSheet_6.zip › SuppMat7Ctrl/Chromosome-11-Healthy.png]

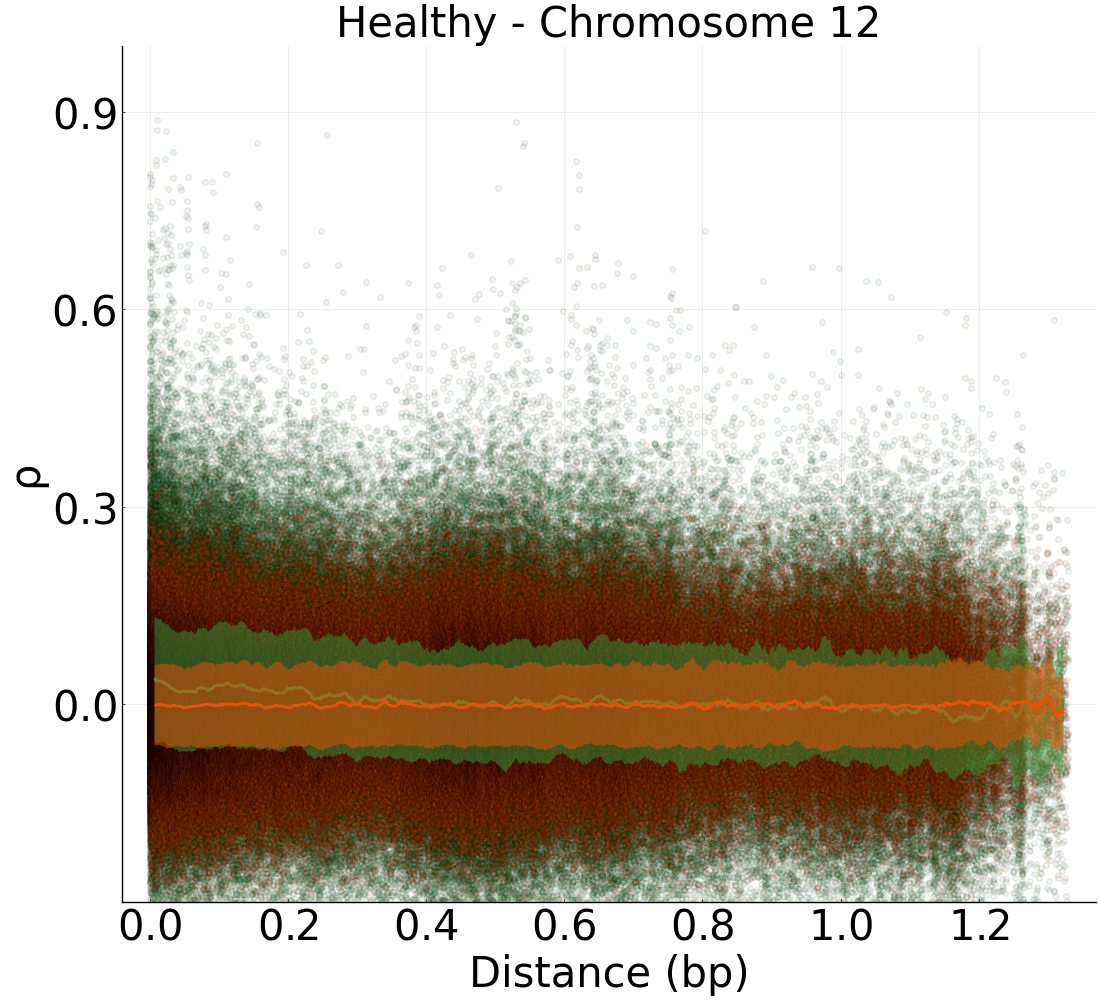

Supplement: Supplementary Material S6 — Heatmaps of Pearson correlation for each chromosome in the Basal phenotype. [file DataSheet_6.zip › SuppMat7Ctrl/Chromosome-12-Healthy.png]

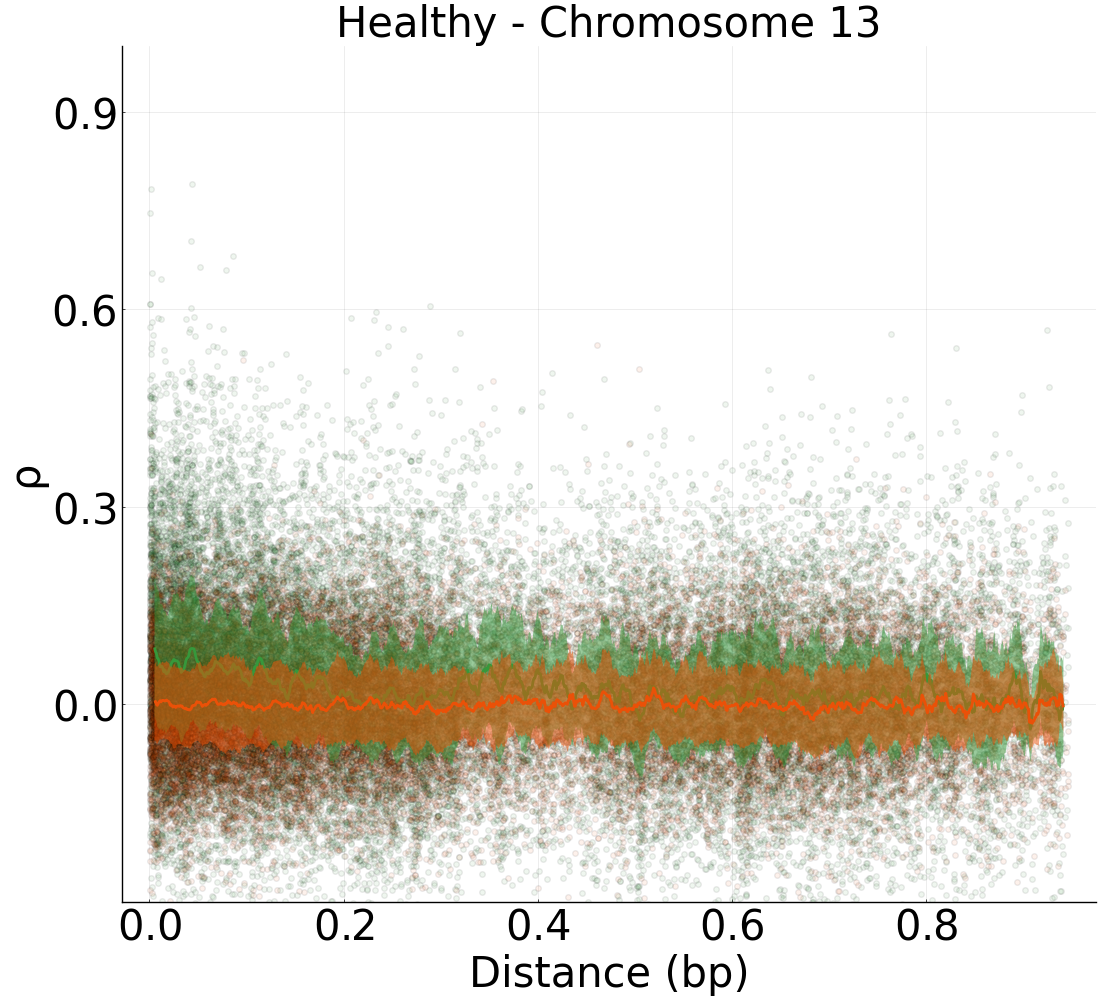

Supplement: Supplementary Material S6 — Heatmaps of Pearson correlation for each chromosome in the Basal phenotype. [file DataSheet_6.zip › SuppMat7Ctrl/Chromosome-13-Healthy.png]

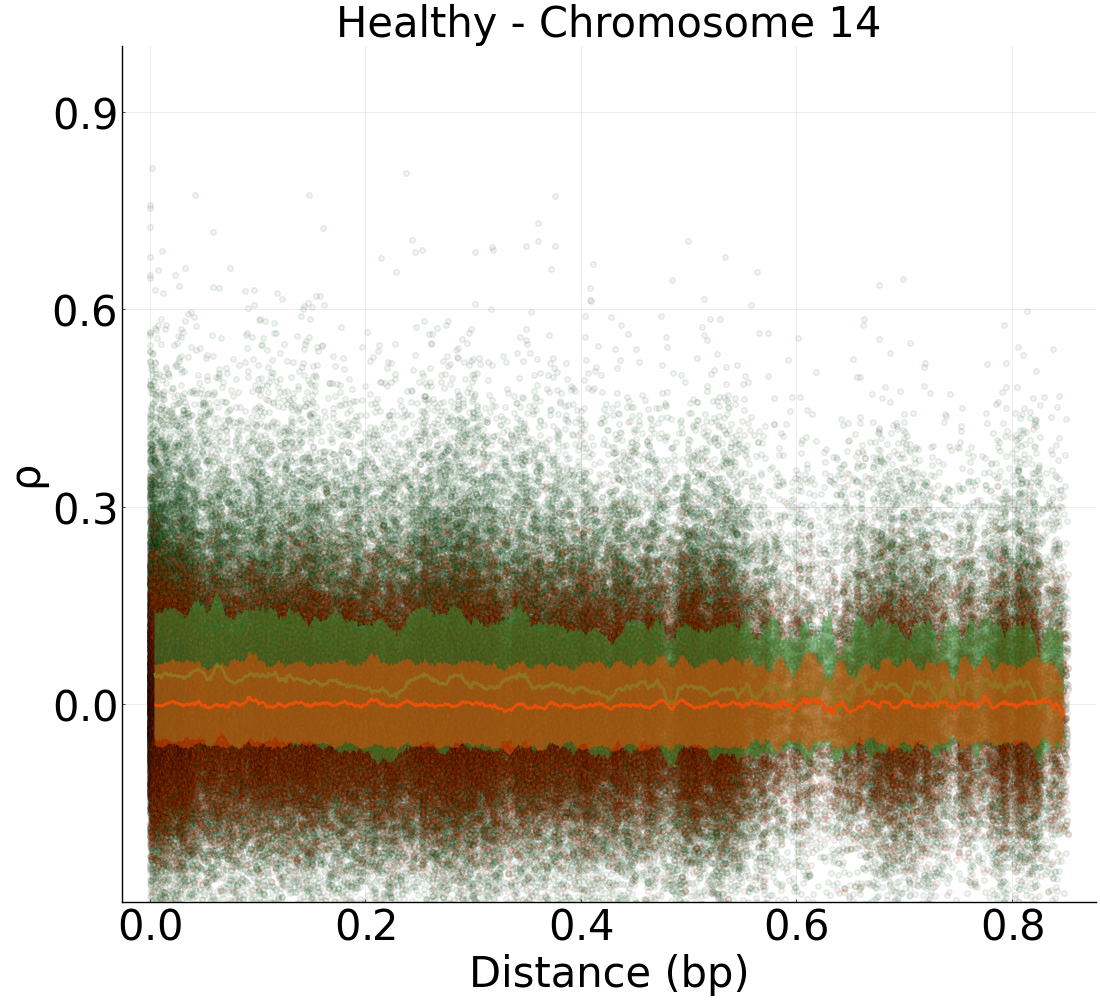

Supplement: Supplementary Material S6 — Heatmaps of Pearson correlation for each chromosome in the Basal phenotype. [file DataSheet_6.zip › SuppMat7Ctrl/Chromosome-14-Healthy.png]

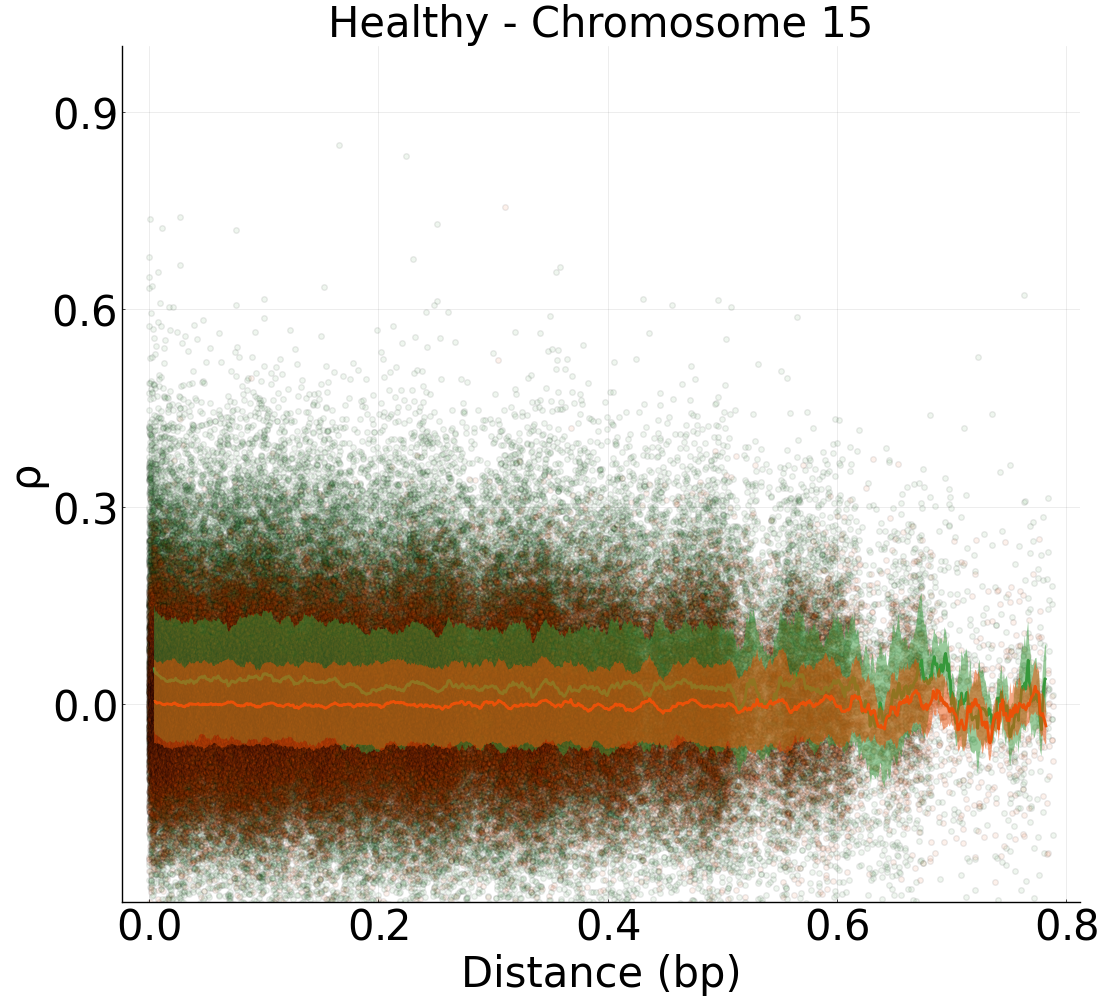

Supplement: Supplementary Material S6 — Heatmaps of Pearson correlation for each chromosome in the Basal phenotype. [file DataSheet_6.zip › SuppMat7Ctrl/Chromosome-15-Healthy.png]

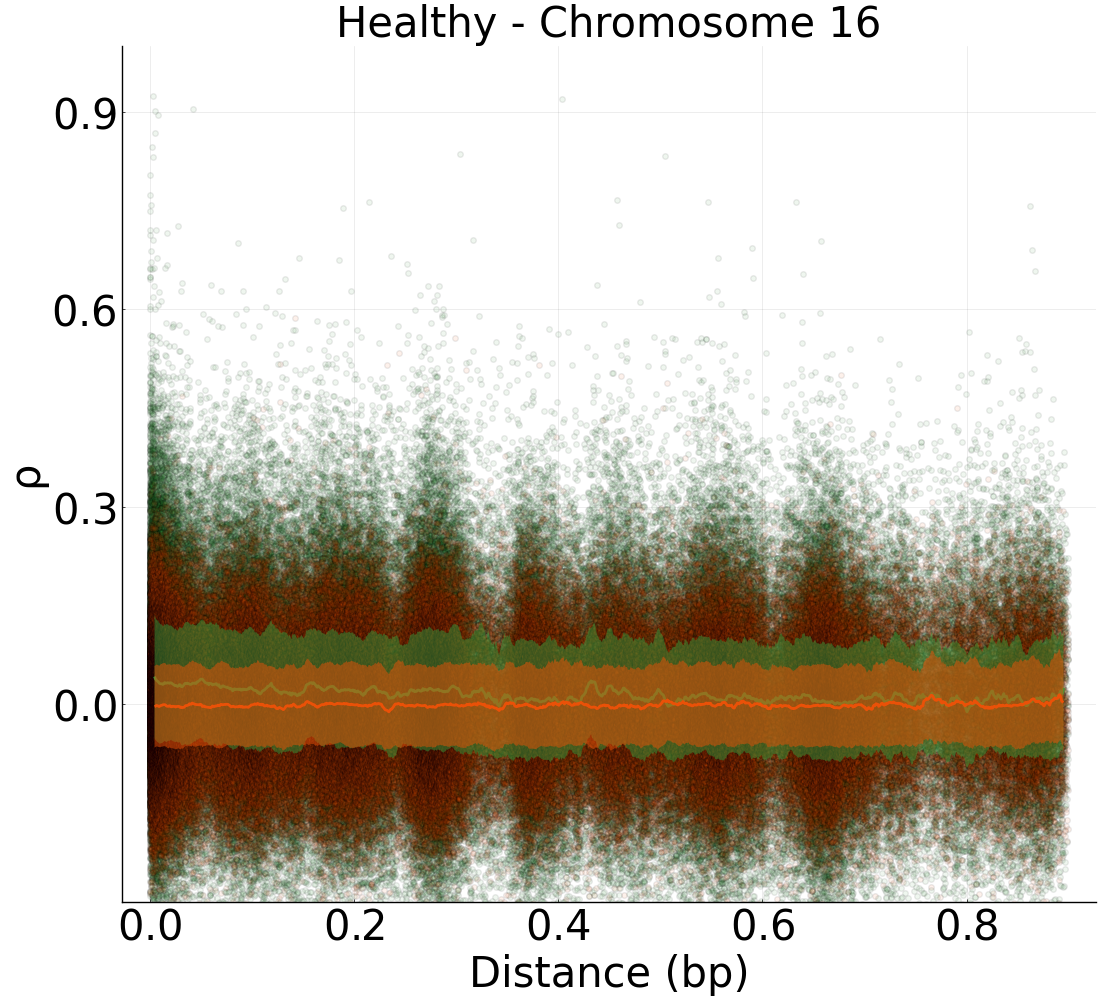

Supplement: Supplementary Material S6 — Heatmaps of Pearson correlation for each chromosome in the Basal phenotype. [file DataSheet_6.zip › SuppMat7Ctrl/Chromosome-16-Healthy.png]

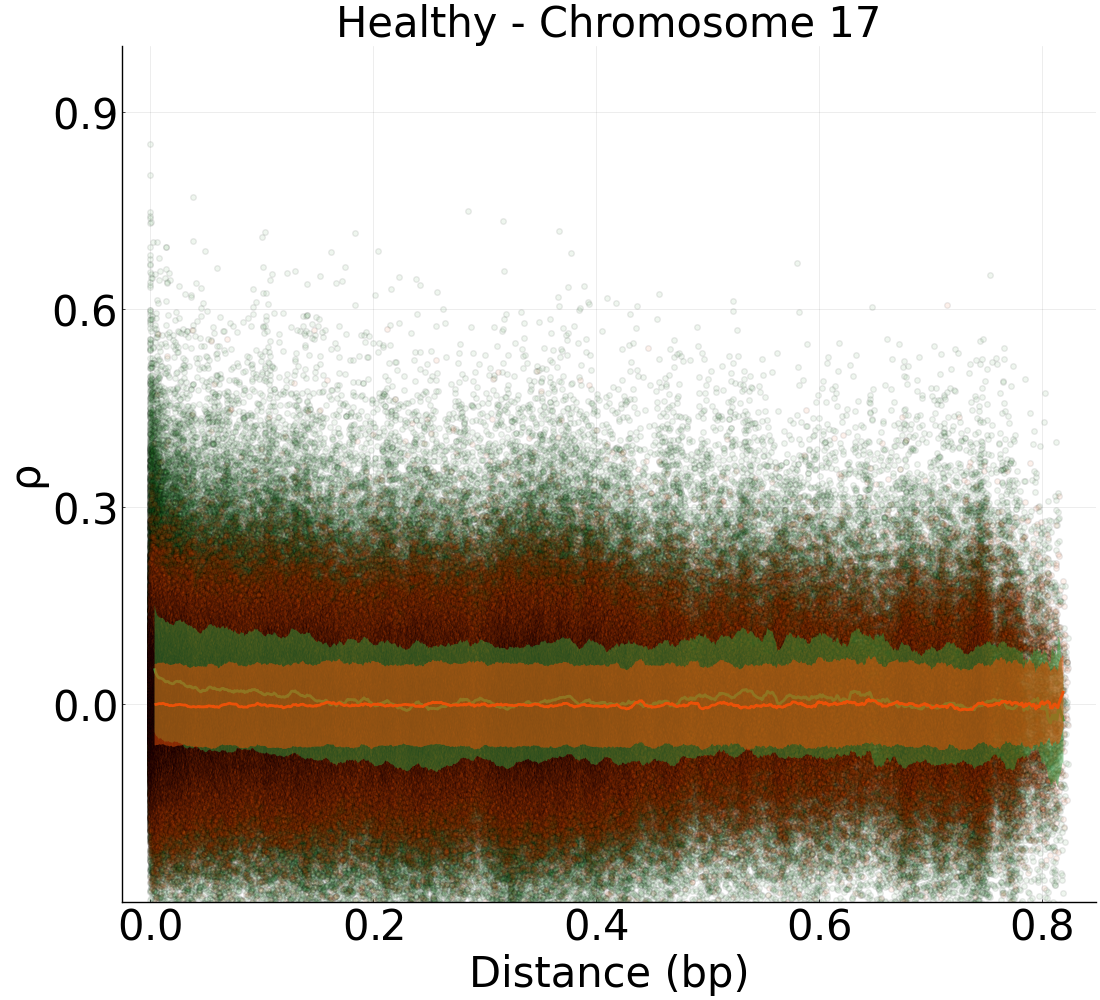

Supplement: Supplementary Material S6 — Heatmaps of Pearson correlation for each chromosome in the Basal phenotype. [file DataSheet_6.zip › SuppMat7Ctrl/Chromosome-17-Healthy.png]

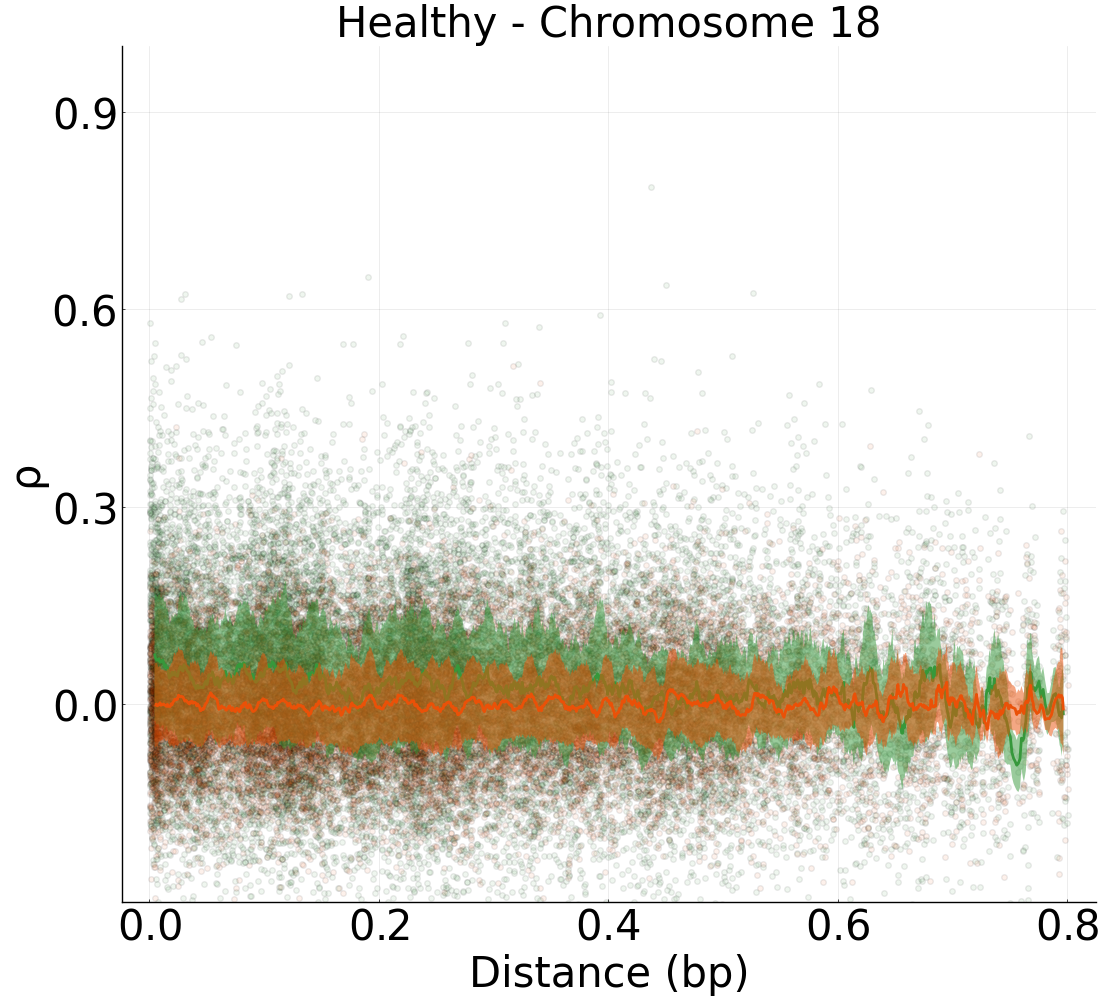

Supplement: Supplementary Material S6 — Heatmaps of Pearson correlation for each chromosome in the Basal phenotype. [file DataSheet_6.zip › SuppMat7Ctrl/Chromosome-18-Healthy.png]

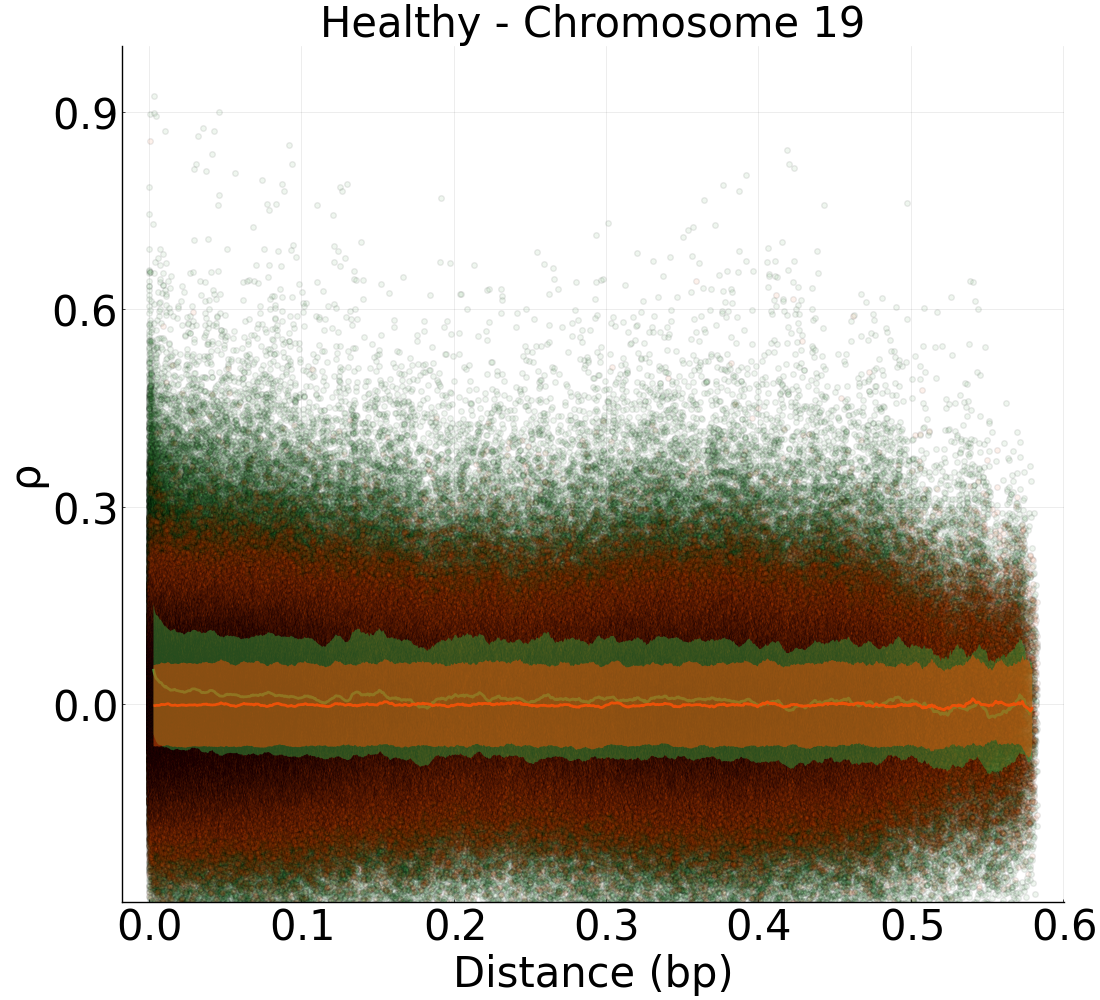

Supplement: Supplementary Material S6 — Heatmaps of Pearson correlation for each chromosome in the Basal phenotype. [file DataSheet_6.zip › SuppMat7Ctrl/Chromosome-19-Healthy.png]

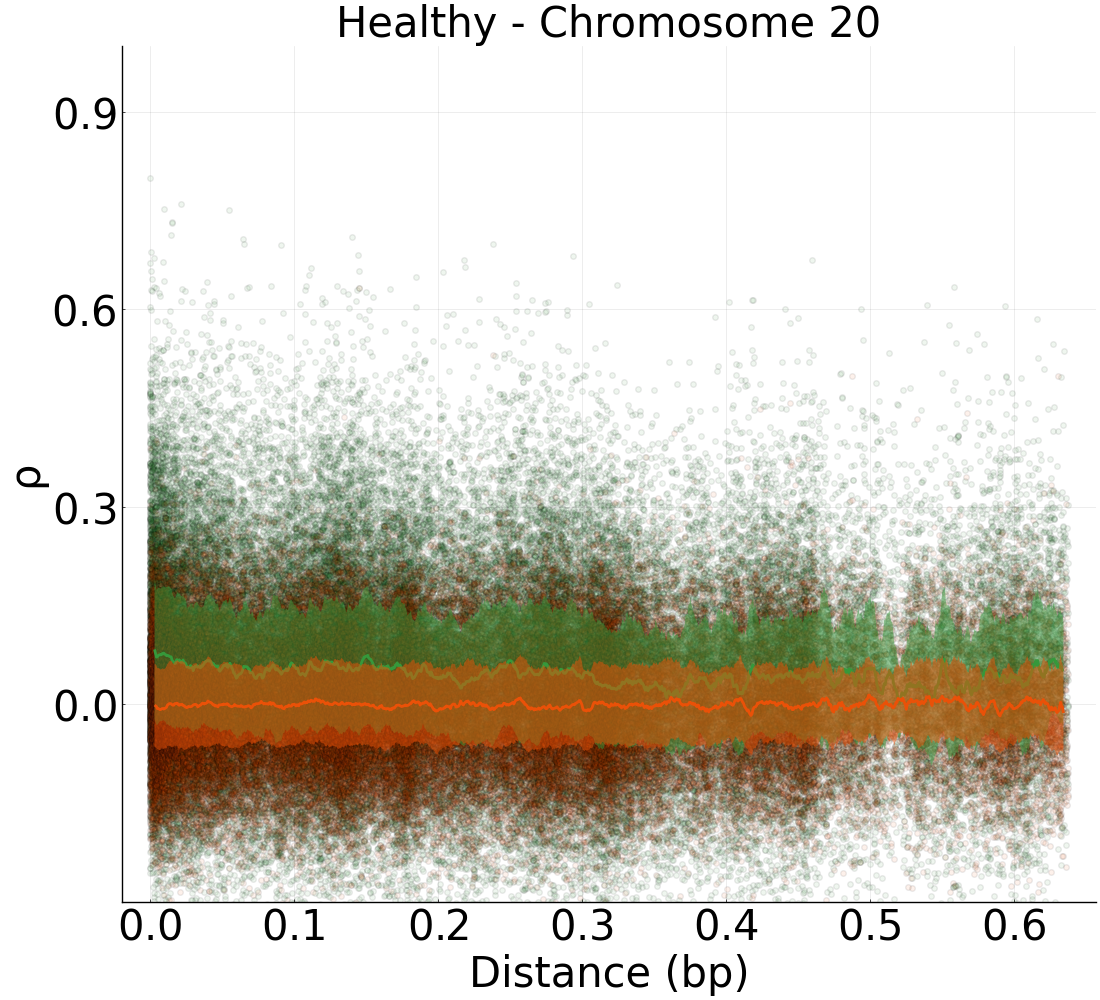

Supplement: Supplementary Material S6 — Heatmaps of Pearson correlation for each chromosome in the Basal phenotype. [file DataSheet_6.zip › SuppMat7Ctrl/Chromosome-20-Healthy.png]

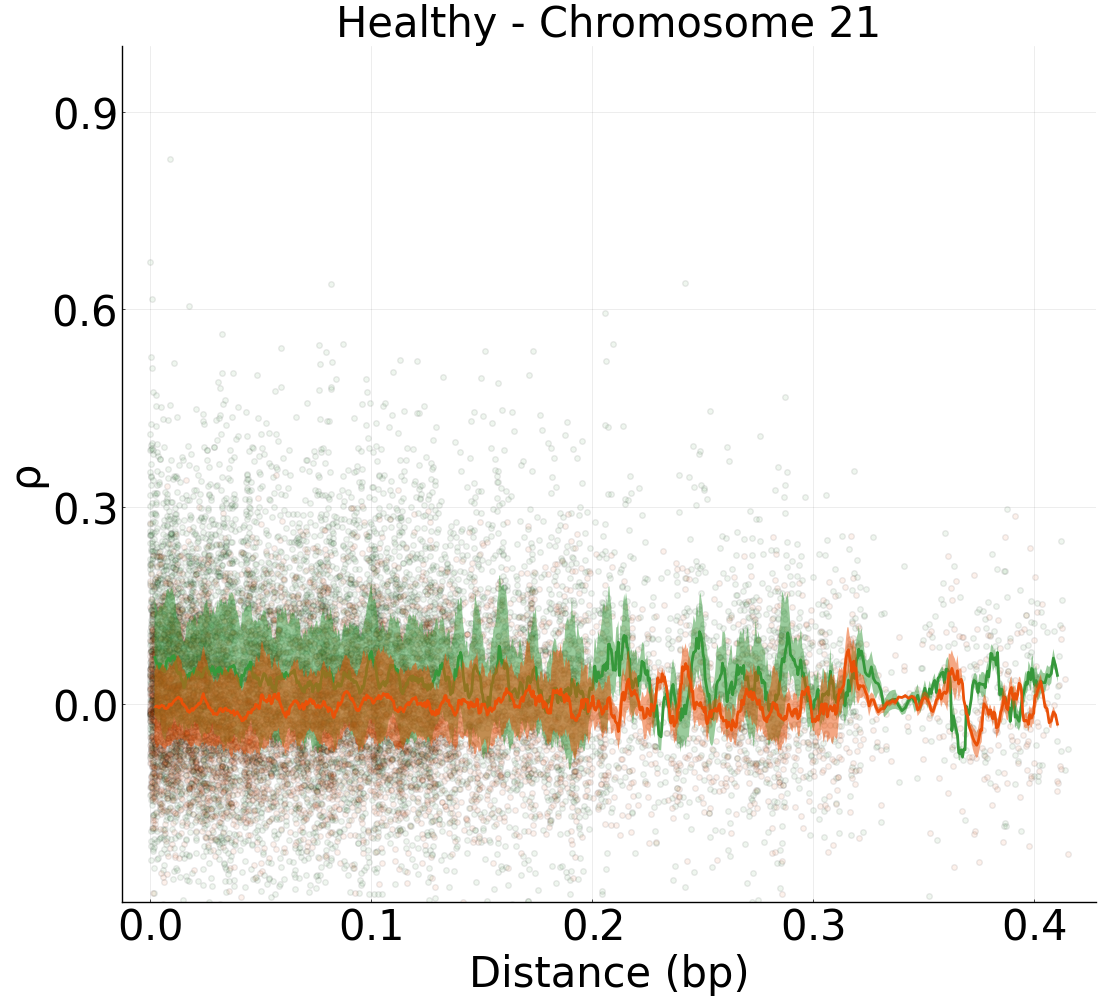

Supplement: Supplementary Material S6 — Heatmaps of Pearson correlation for each chromosome in the Basal phenotype. [file DataSheet_6.zip › SuppMat7Ctrl/Chromosome-21-Healthy.png]

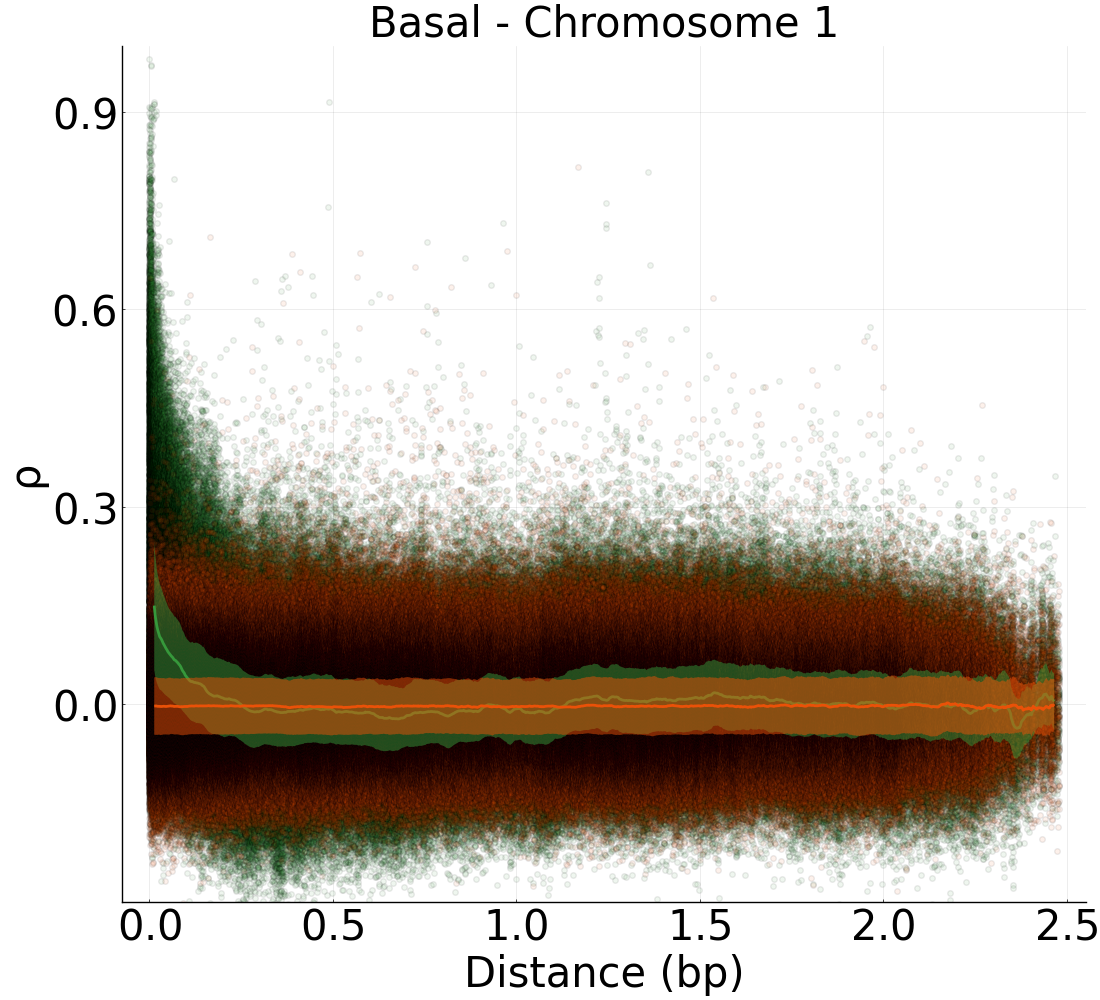

Supplement: Supplementary Material S7 to S11 — Pearson distribution scatter plots for normal adjacent tissue, Basal HER2+, Luminal A and Luminal B, respectively. These plots show correlations sorted by gene start position for the four cancer phenotypes and the adjacent normal network per chromosome. [file DataSheet_7.zip › SuppMat8Basal/Chromosome-1-Basal.png]

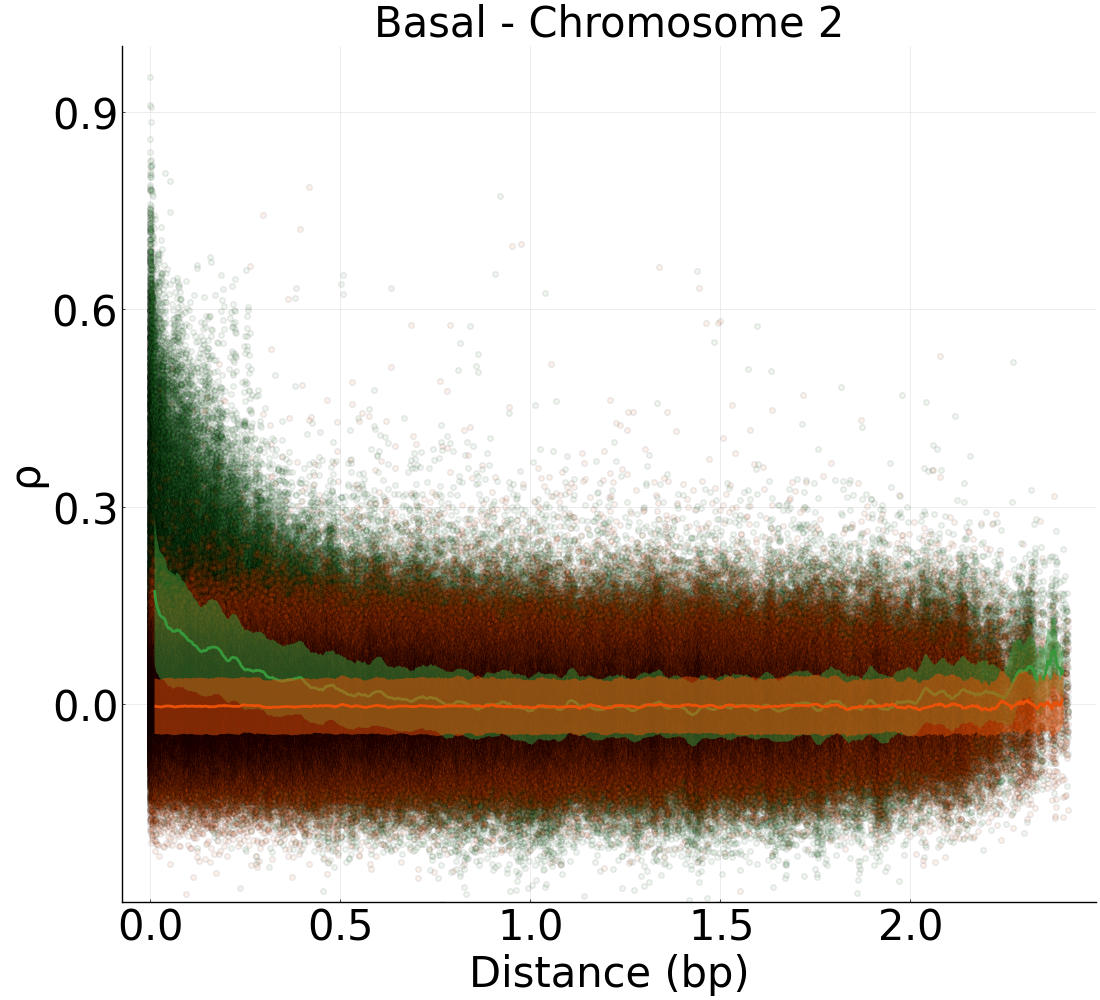

Supplement: Supplementary Material S7 to S11 — Pearson distribution scatter plots for normal adjacent tissue, Basal HER2+, Luminal A and Luminal B, respectively. These plots show correlations sorted by gene start position for the four cancer phenotypes and the adjacent normal network per chromosome. [file DataSheet_7.zip › SuppMat8Basal/Chromosome-2-Basal.png]

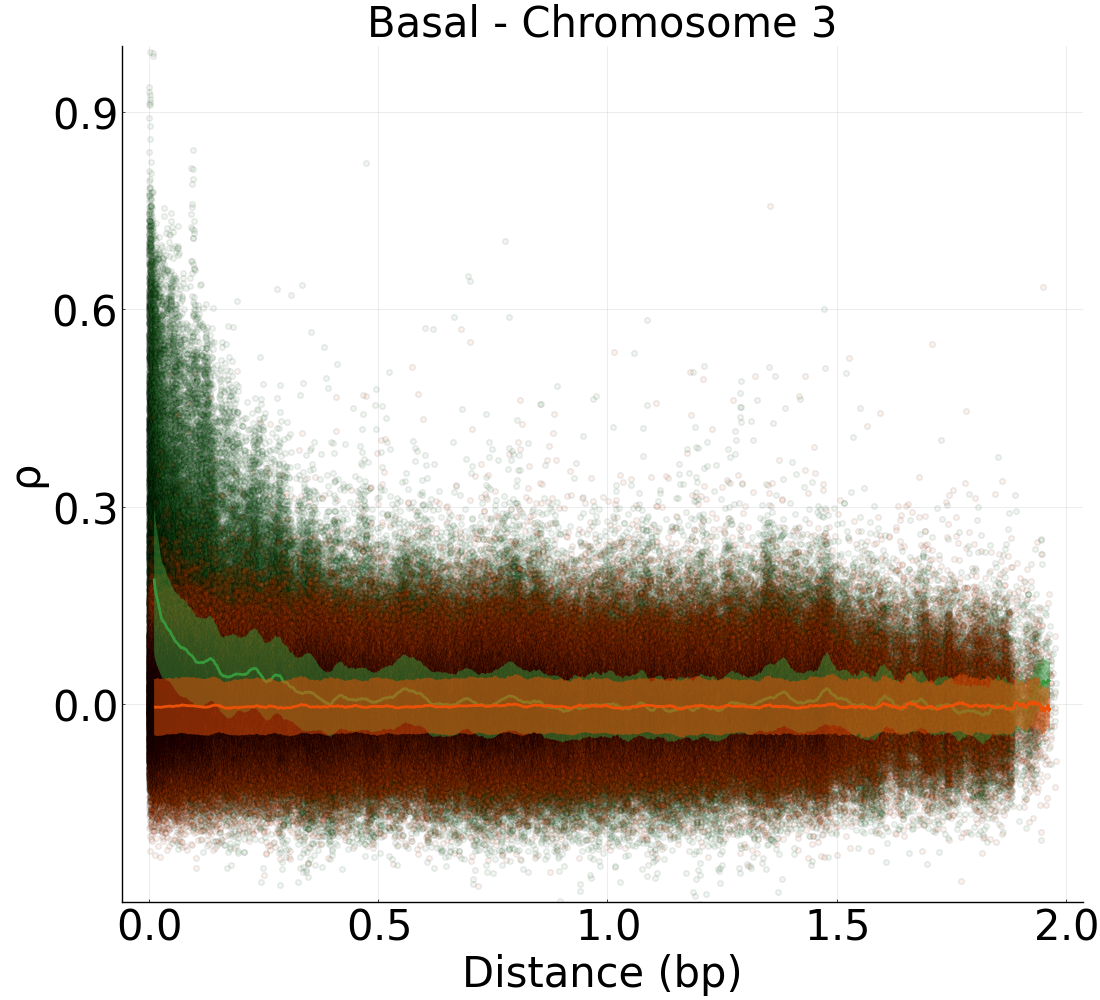

Supplement: Supplementary Material S7 to S11 — Pearson distribution scatter plots for normal adjacent tissue, Basal HER2+, Luminal A and Luminal B, respectively. These plots show correlations sorted by gene start position for the four cancer phenotypes and the adjacent normal network per chromosome. [file DataSheet_7.zip › SuppMat8Basal/Chromosome-3-Basal.png]

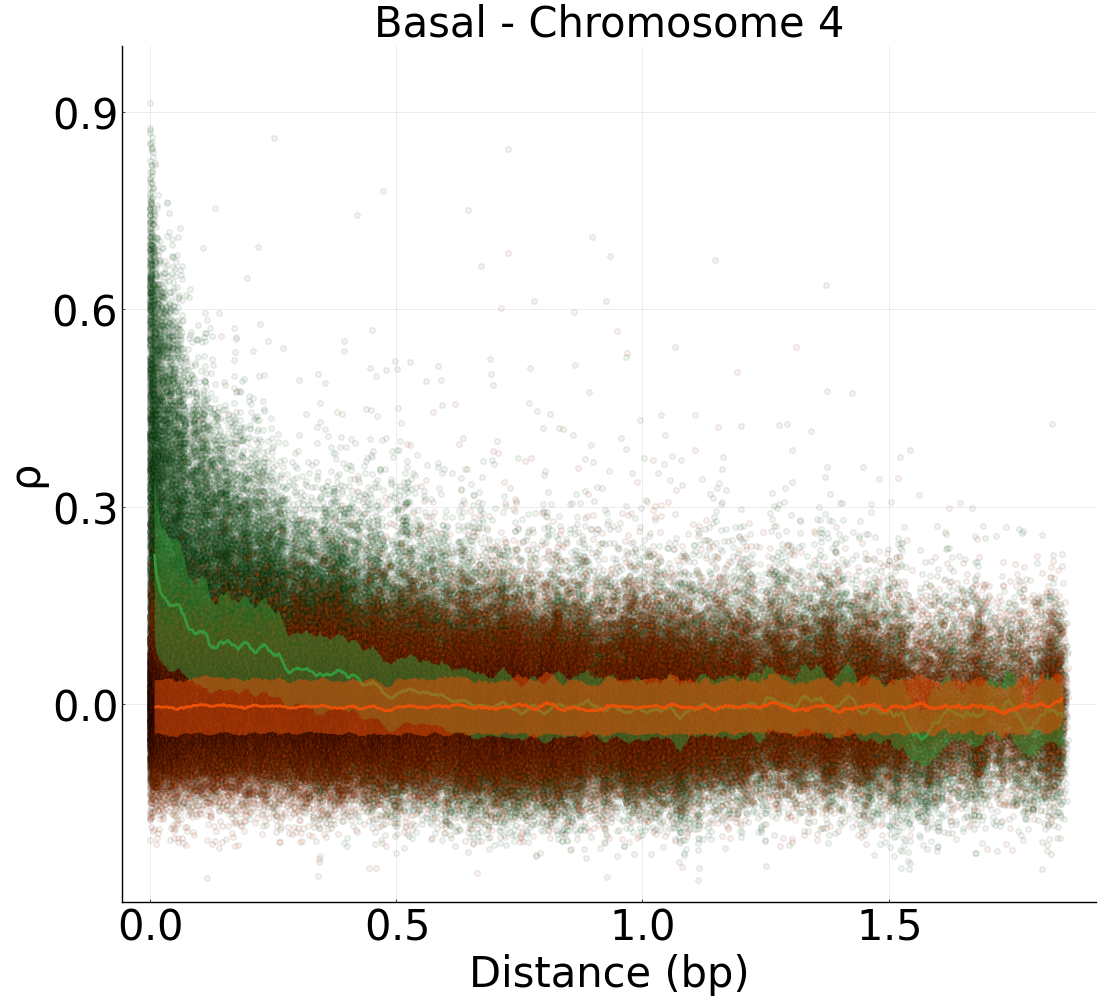

Supplement: Supplementary Material S7 to S11 — Pearson distribution scatter plots for normal adjacent tissue, Basal HER2+, Luminal A and Luminal B, respectively. These plots show correlations sorted by gene start position for the four cancer phenotypes and the adjacent normal network per chromosome. [file DataSheet_7.zip › SuppMat8Basal/Chromosome-4-Basal.png]

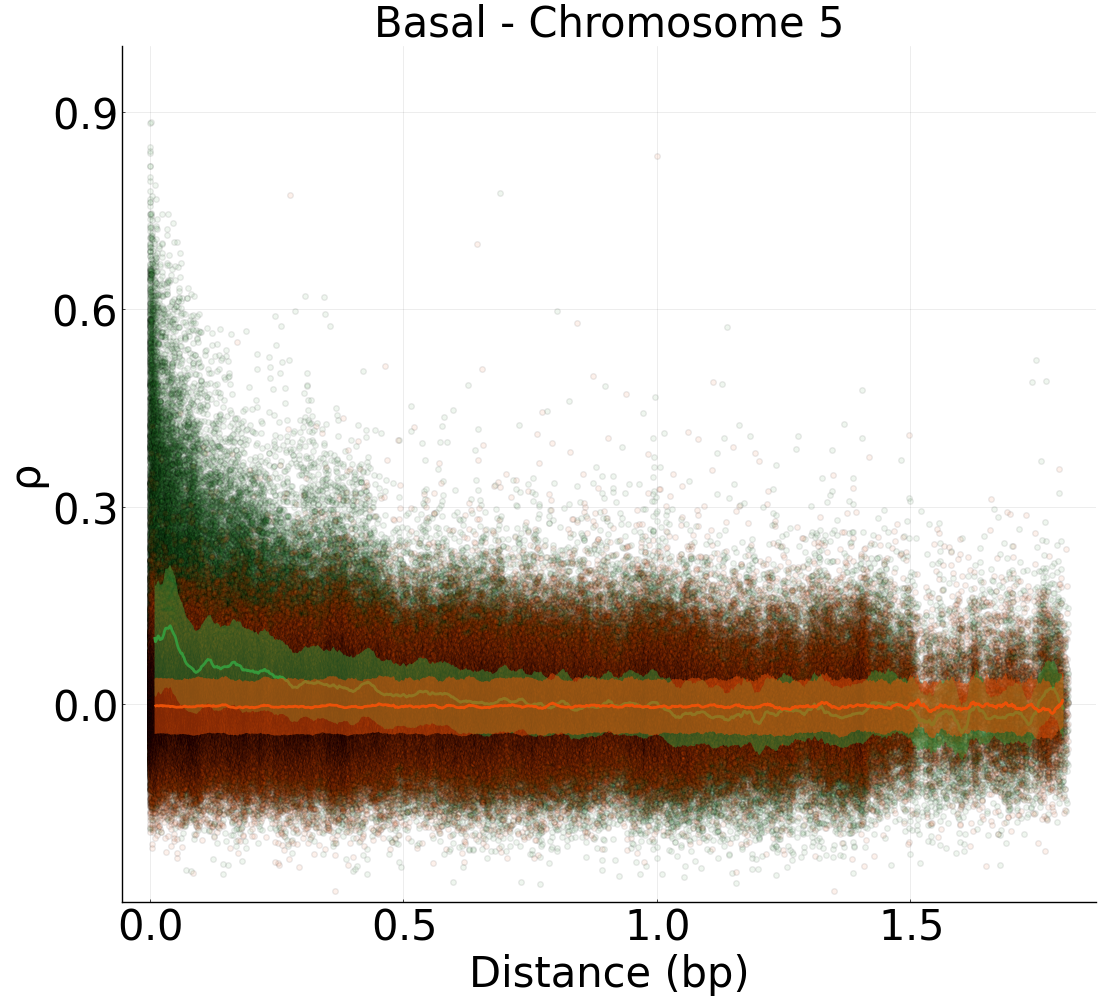

Supplement: Supplementary Material S7 to S11 — Pearson distribution scatter plots for normal adjacent tissue, Basal HER2+, Luminal A and Luminal B, respectively. These plots show correlations sorted by gene start position for the four cancer phenotypes and the adjacent normal network per chromosome. [file DataSheet_7.zip › SuppMat8Basal/Chromosome-5-Basal.png]

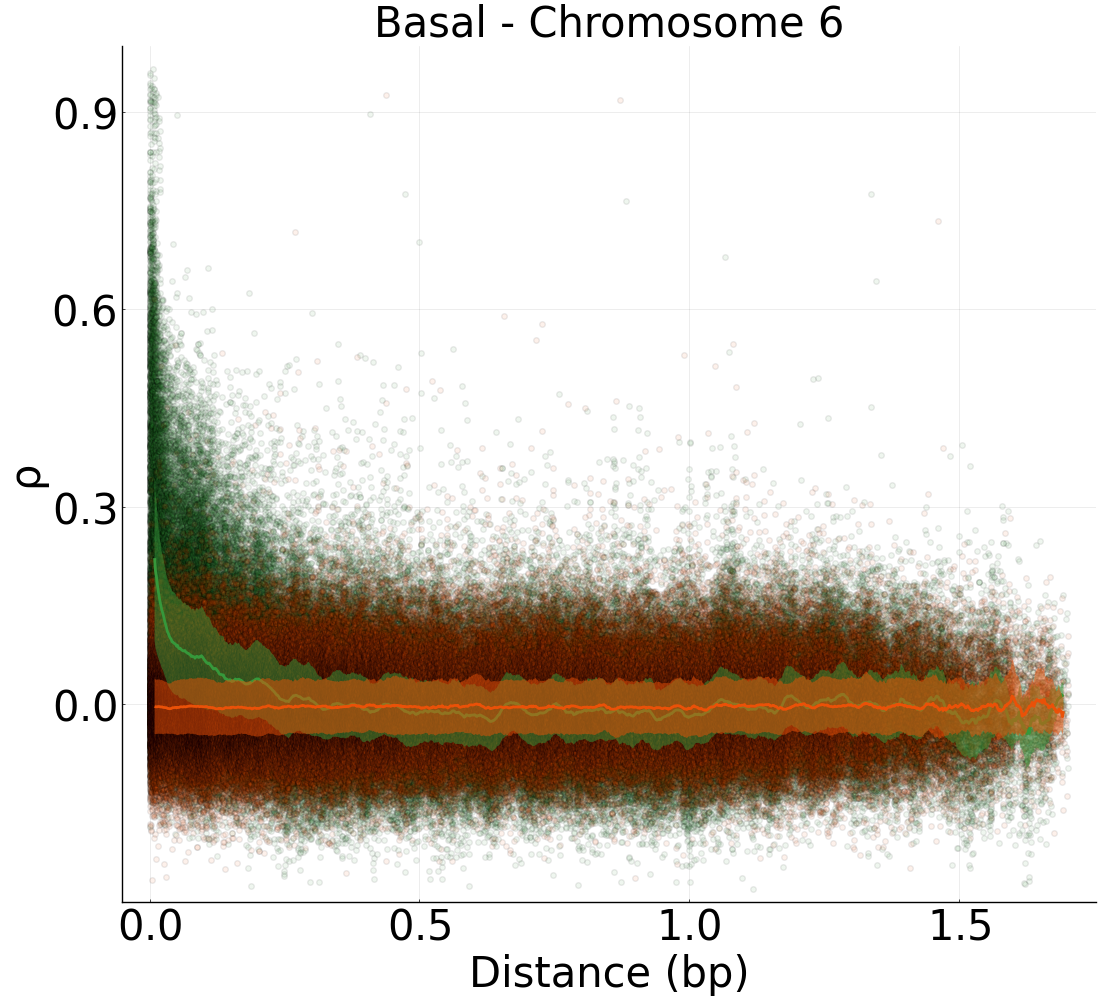

Supplement: Supplementary Material S7 to S11 — Pearson distribution scatter plots for normal adjacent tissue, Basal HER2+, Luminal A and Luminal B, respectively. These plots show correlations sorted by gene start position for the four cancer phenotypes and the adjacent normal network per chromosome. [file DataSheet_7.zip › SuppMat8Basal/Chromosome-6-Basal.png]

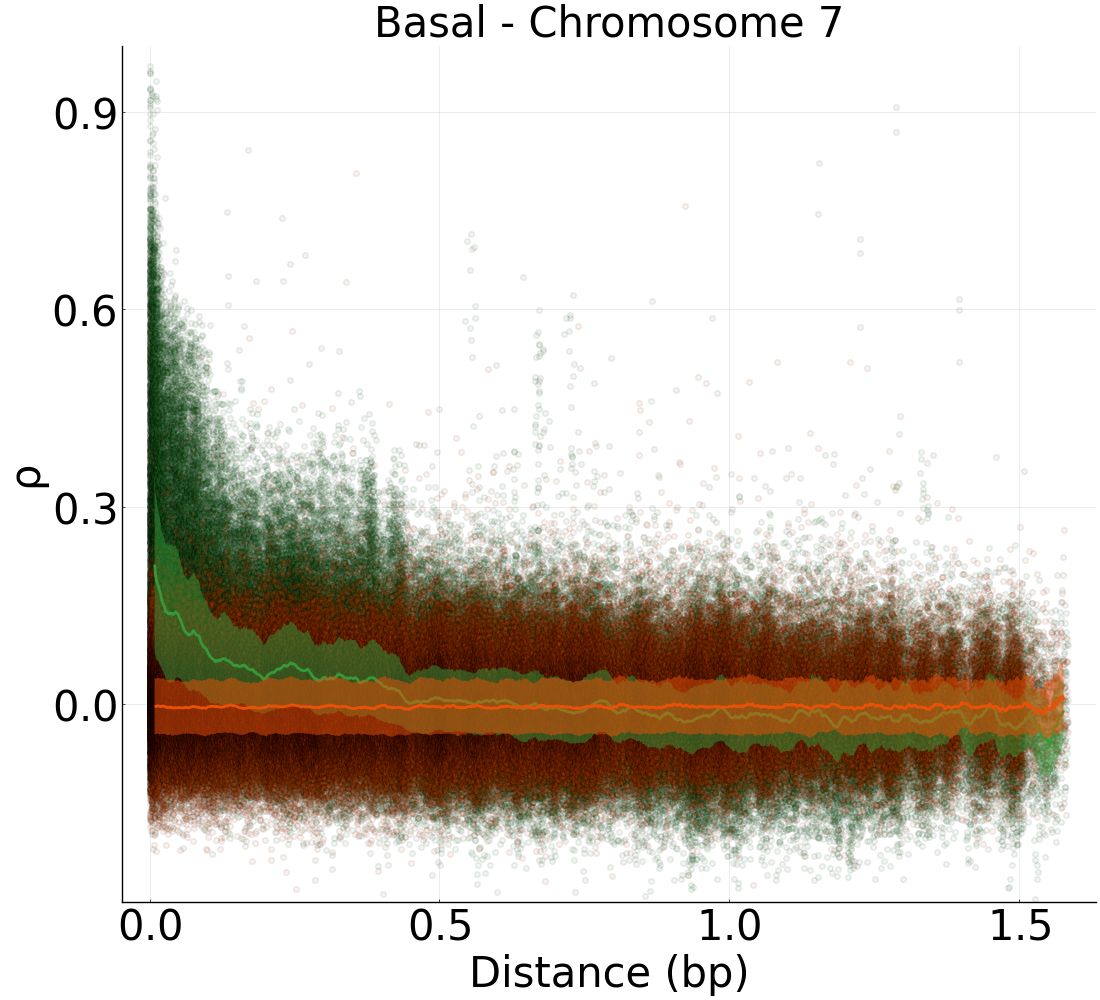

Supplement: Supplementary Material S7 to S11 — Pearson distribution scatter plots for normal adjacent tissue, Basal HER2+, Luminal A and Luminal B, respectively. These plots show correlations sorted by gene start position for the four cancer phenotypes and the adjacent normal network per chromosome. [file DataSheet_7.zip › SuppMat8Basal/Chromosome-7-Basal.png]

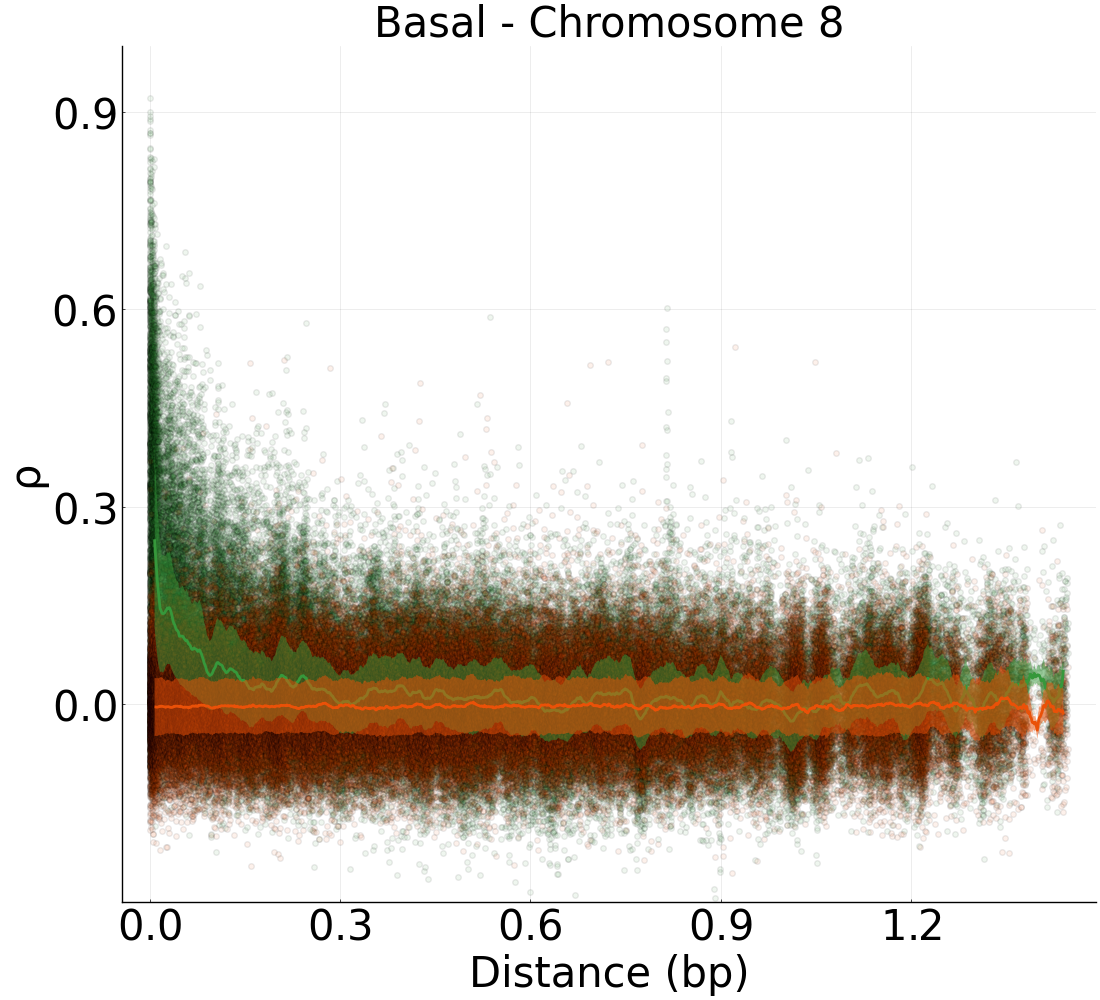

Supplement: Supplementary Material S7 to S11 — Pearson distribution scatter plots for normal adjacent tissue, Basal HER2+, Luminal A and Luminal B, respectively. These plots show correlations sorted by gene start position for the four cancer phenotypes and the adjacent normal network per chromosome. [file DataSheet_7.zip › SuppMat8Basal/Chromosome-8-Basal.png]

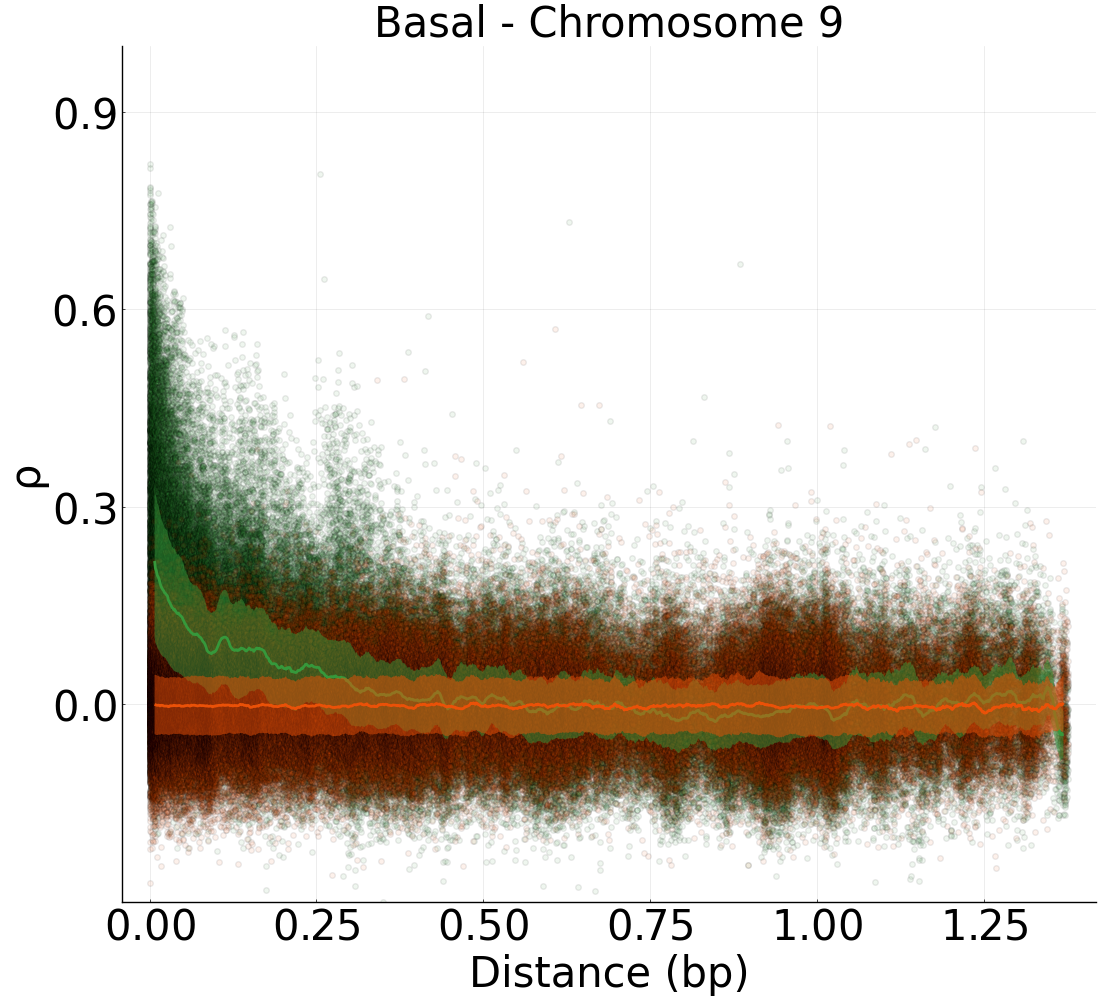

Supplement: Supplementary Material S7 to S11 — Pearson distribution scatter plots for normal adjacent tissue, Basal HER2+, Luminal A and Luminal B, respectively. These plots show correlations sorted by gene start position for the four cancer phenotypes and the adjacent normal network per chromosome. [file DataSheet_7.zip › SuppMat8Basal/Chromosome-9-Basal.png]

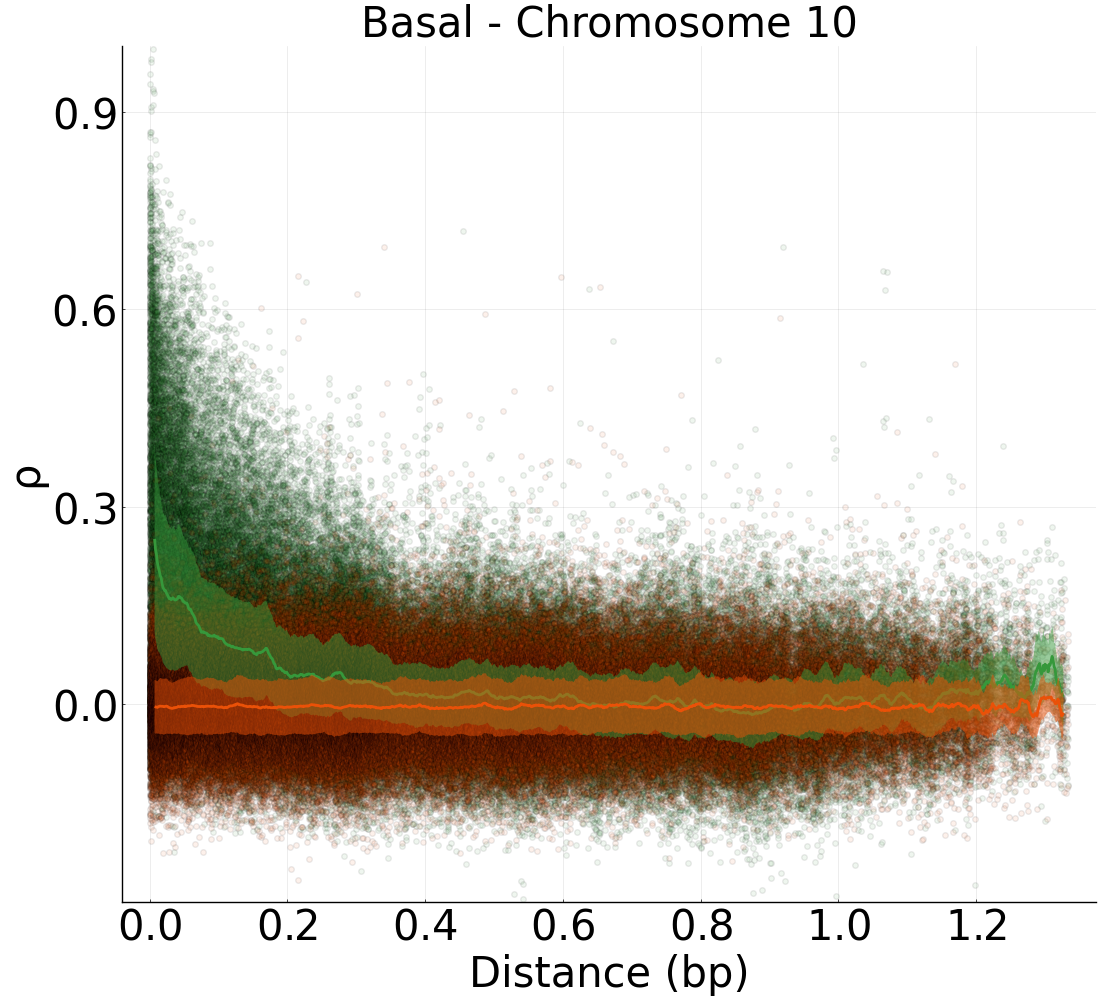

Supplement: Supplementary Material S7 to S11 — Pearson distribution scatter plots for normal adjacent tissue, Basal HER2+, Luminal A and Luminal B, respectively. These plots show correlations sorted by gene start position for the four cancer phenotypes and the adjacent normal network per chromosome. [file DataSheet_7.zip › SuppMat8Basal/Chromosome-10-Basal.png]

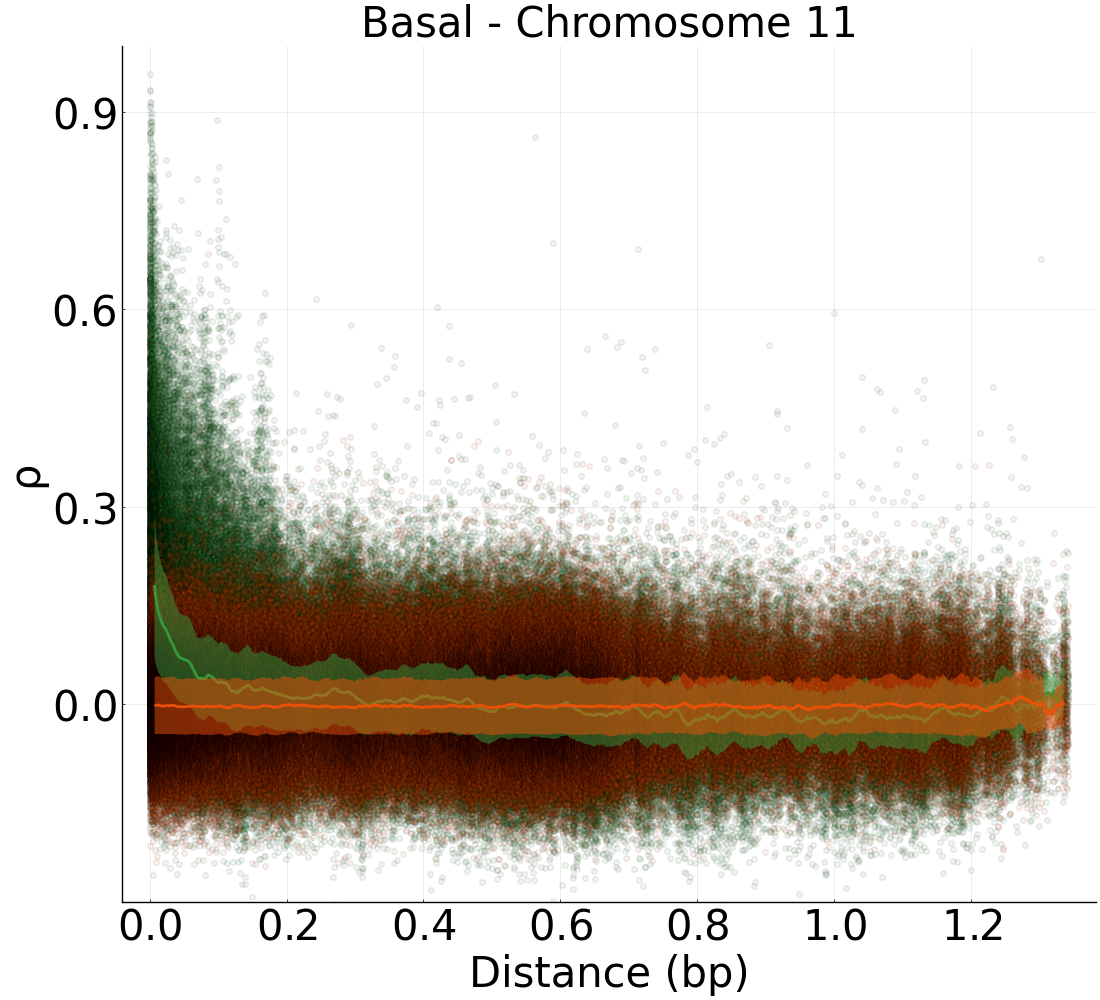

Supplement: Supplementary Material S7 to S11 — Pearson distribution scatter plots for normal adjacent tissue, Basal HER2+, Luminal A and Luminal B, respectively. These plots show correlations sorted by gene start position for the four cancer phenotypes and the adjacent normal network per chromosome. [file DataSheet_7.zip › SuppMat8Basal/Chromosome-11-Basal.png]

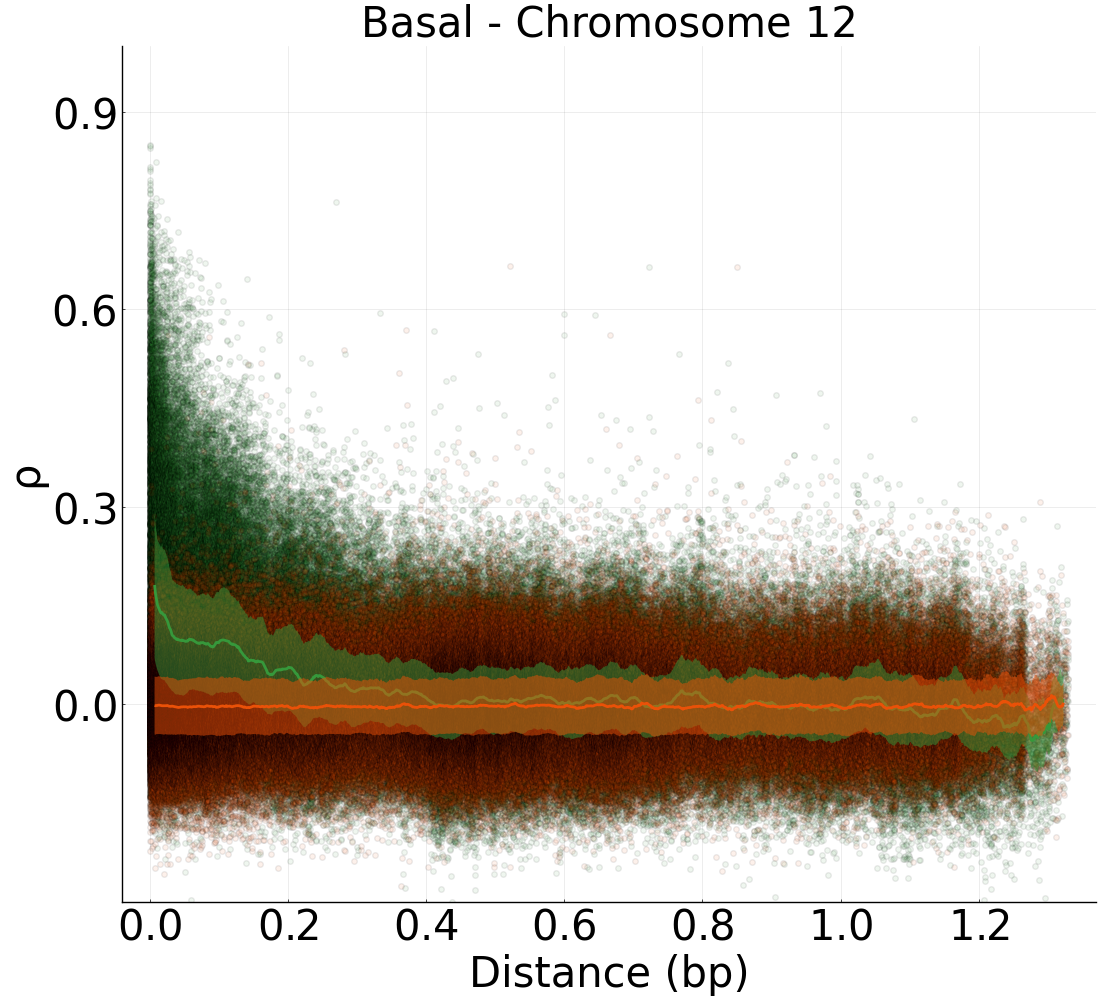

Supplement: Supplementary Material S7 to S11 — Pearson distribution scatter plots for normal adjacent tissue, Basal HER2+, Luminal A and Luminal B, respectively. These plots show correlations sorted by gene start position for the four cancer phenotypes and the adjacent normal network per chromosome. [file DataSheet_7.zip › SuppMat8Basal/Chromosome-12-Basal.png]

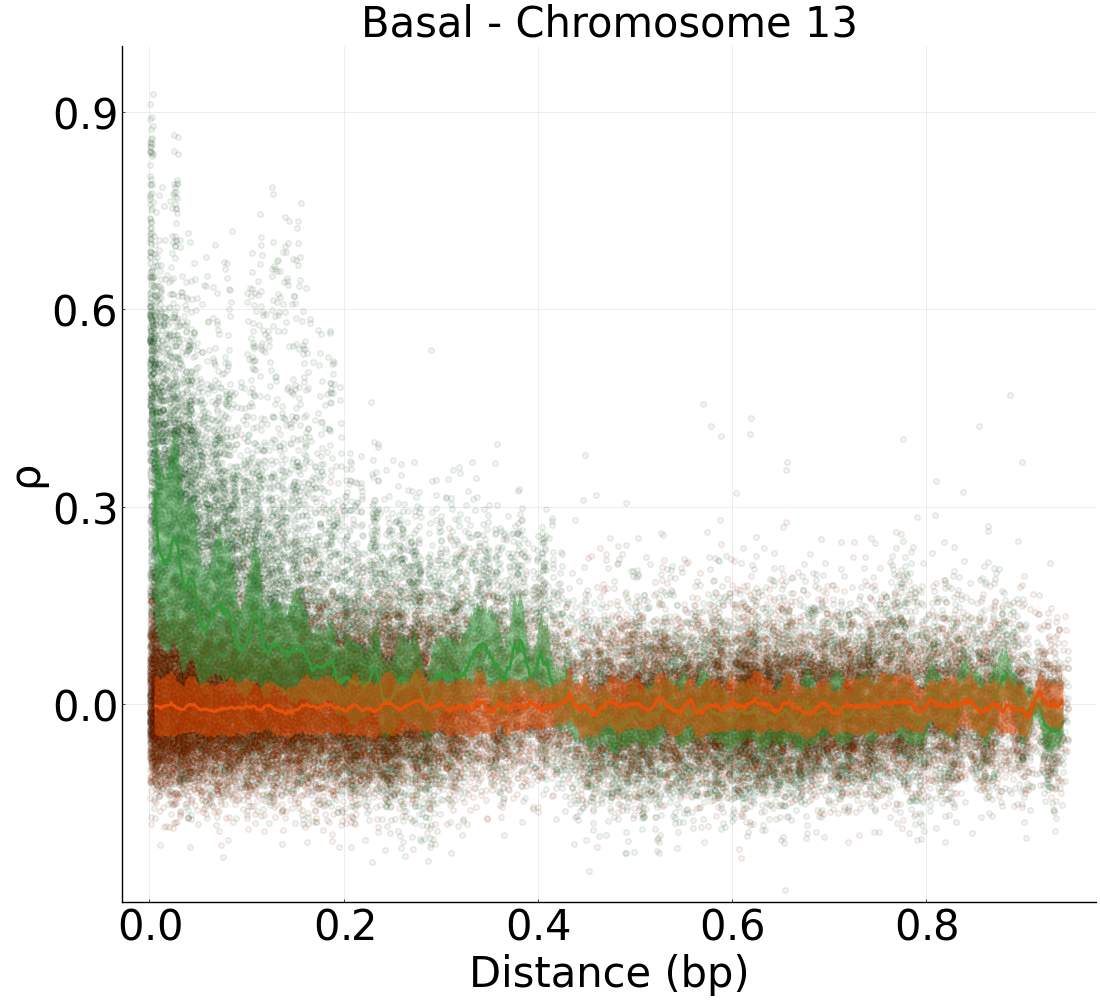

Supplement: Supplementary Material S7 to S11 — Pearson distribution scatter plots for normal adjacent tissue, Basal HER2+, Luminal A and Luminal B, respectively. These plots show correlations sorted by gene start position for the four cancer phenotypes and the adjacent normal network per chromosome. [file DataSheet_7.zip › SuppMat8Basal/Chromosome-13-Basal.png]

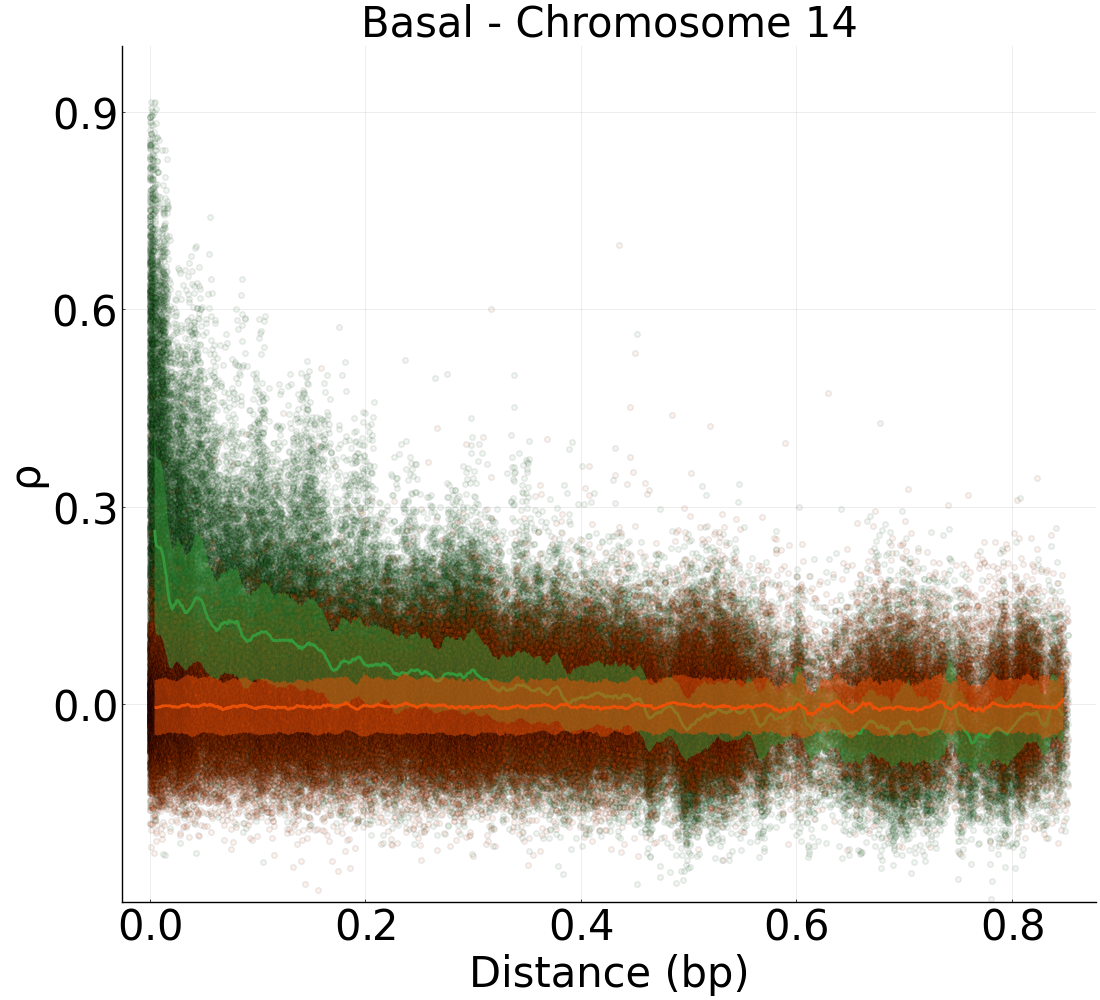

Supplement: Supplementary Material S7 to S11 — Pearson distribution scatter plots for normal adjacent tissue, Basal HER2+, Luminal A and Luminal B, respectively. These plots show correlations sorted by gene start position for the four cancer phenotypes and the adjacent normal network per chromosome. [file DataSheet_7.zip › SuppMat8Basal/Chromosome-14-Basal.png]

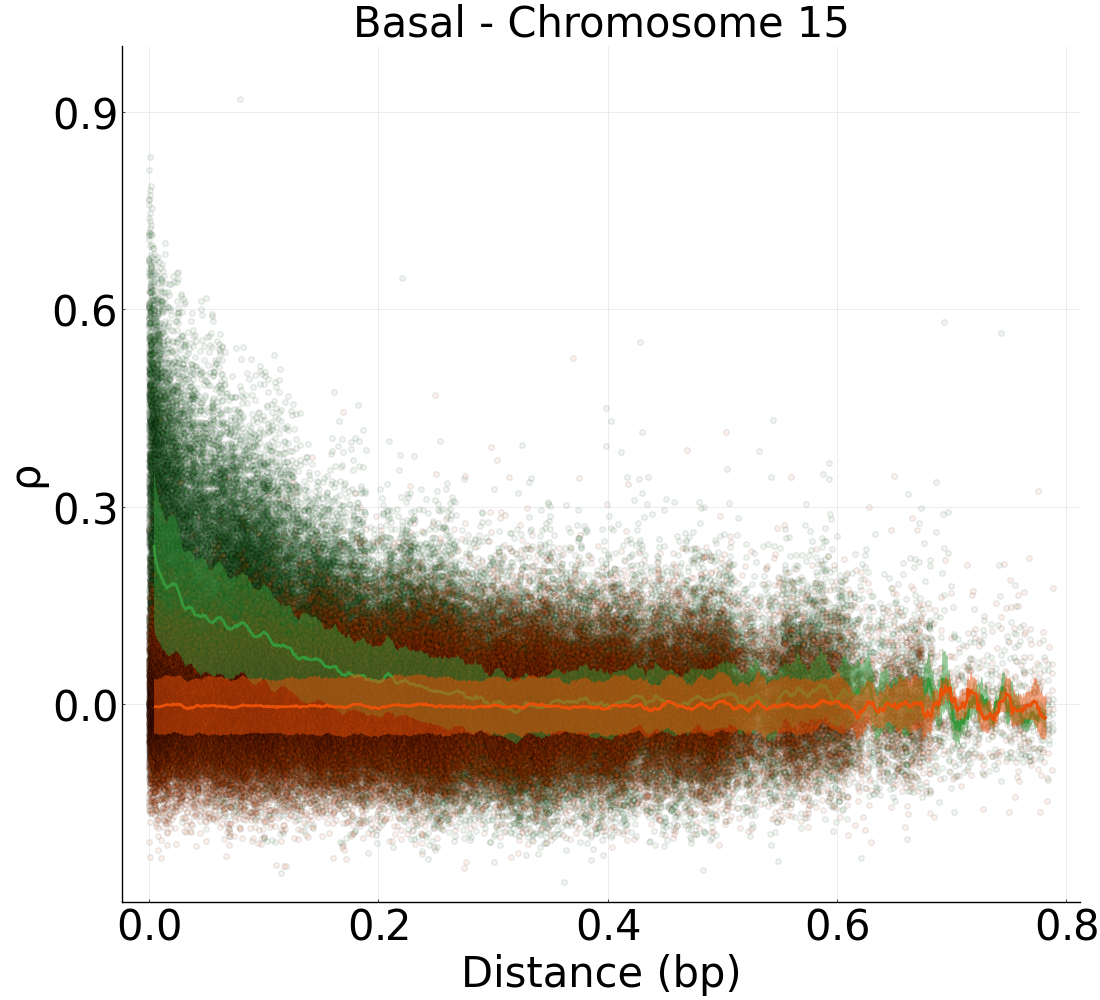

Supplement: Supplementary Material S7 to S11 — Pearson distribution scatter plots for normal adjacent tissue, Basal HER2+, Luminal A and Luminal B, respectively. These plots show correlations sorted by gene start position for the four cancer phenotypes and the adjacent normal network per chromosome. [file DataSheet_7.zip › SuppMat8Basal/Chromosome-15-Basal.png]

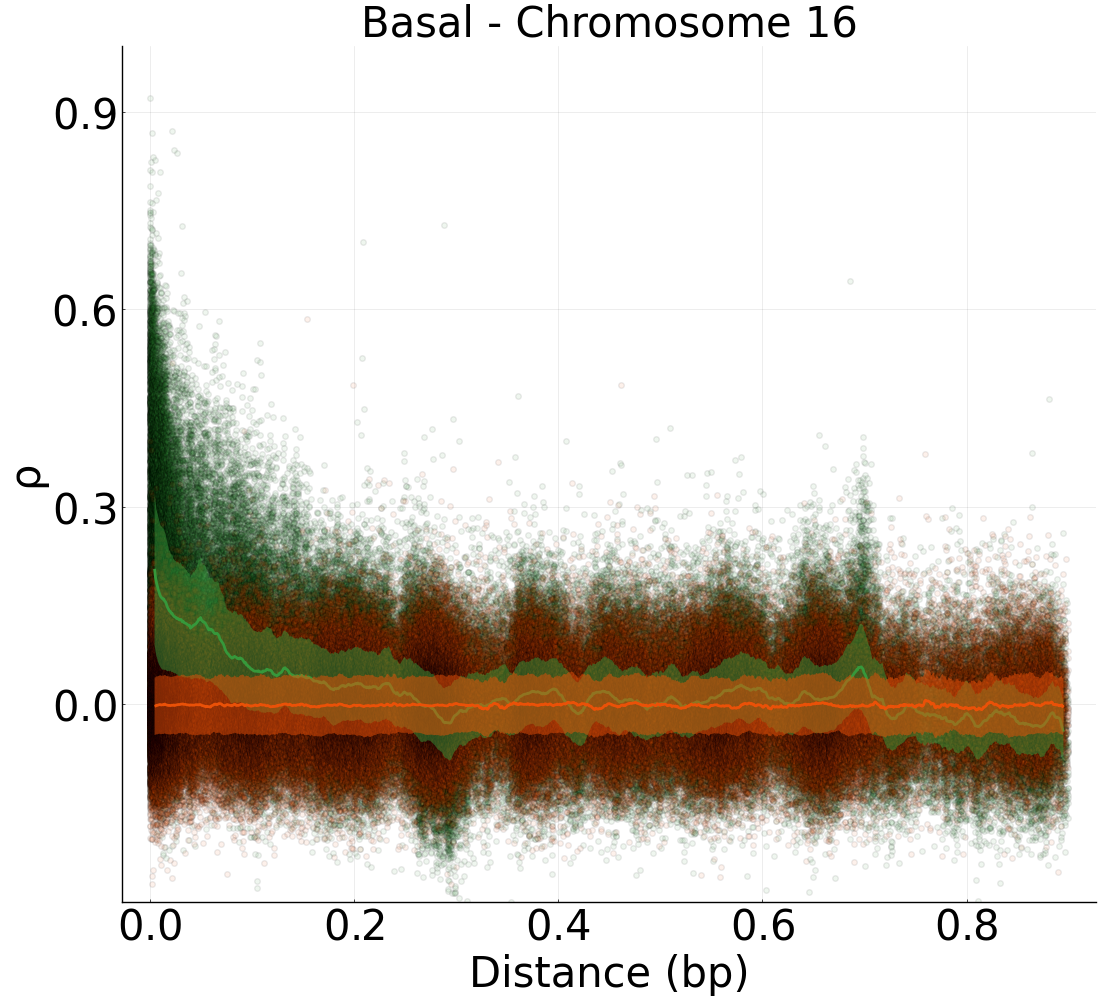

Supplement: Supplementary Material S7 to S11 — Pearson distribution scatter plots for normal adjacent tissue, Basal HER2+, Luminal A and Luminal B, respectively. These plots show correlations sorted by gene start position for the four cancer phenotypes and the adjacent normal network per chromosome. [file DataSheet_7.zip › SuppMat8Basal/Chromosome-16-Basal.png]

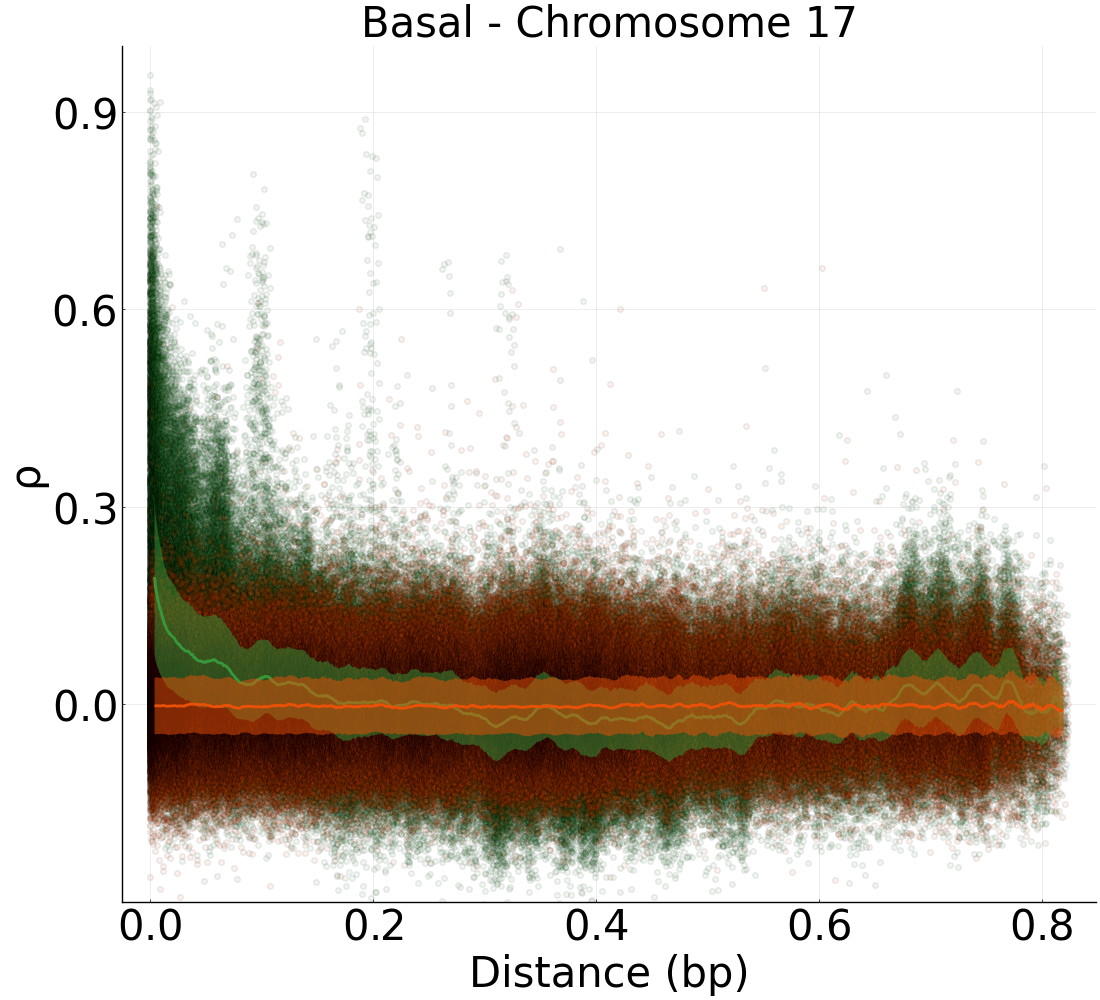

Supplement: Supplementary Material S7 to S11 — Pearson distribution scatter plots for normal adjacent tissue, Basal HER2+, Luminal A and Luminal B, respectively. These plots show correlations sorted by gene start position for the four cancer phenotypes and the adjacent normal network per chromosome. [file DataSheet_7.zip › SuppMat8Basal/Chromosome-17-Basal.png]

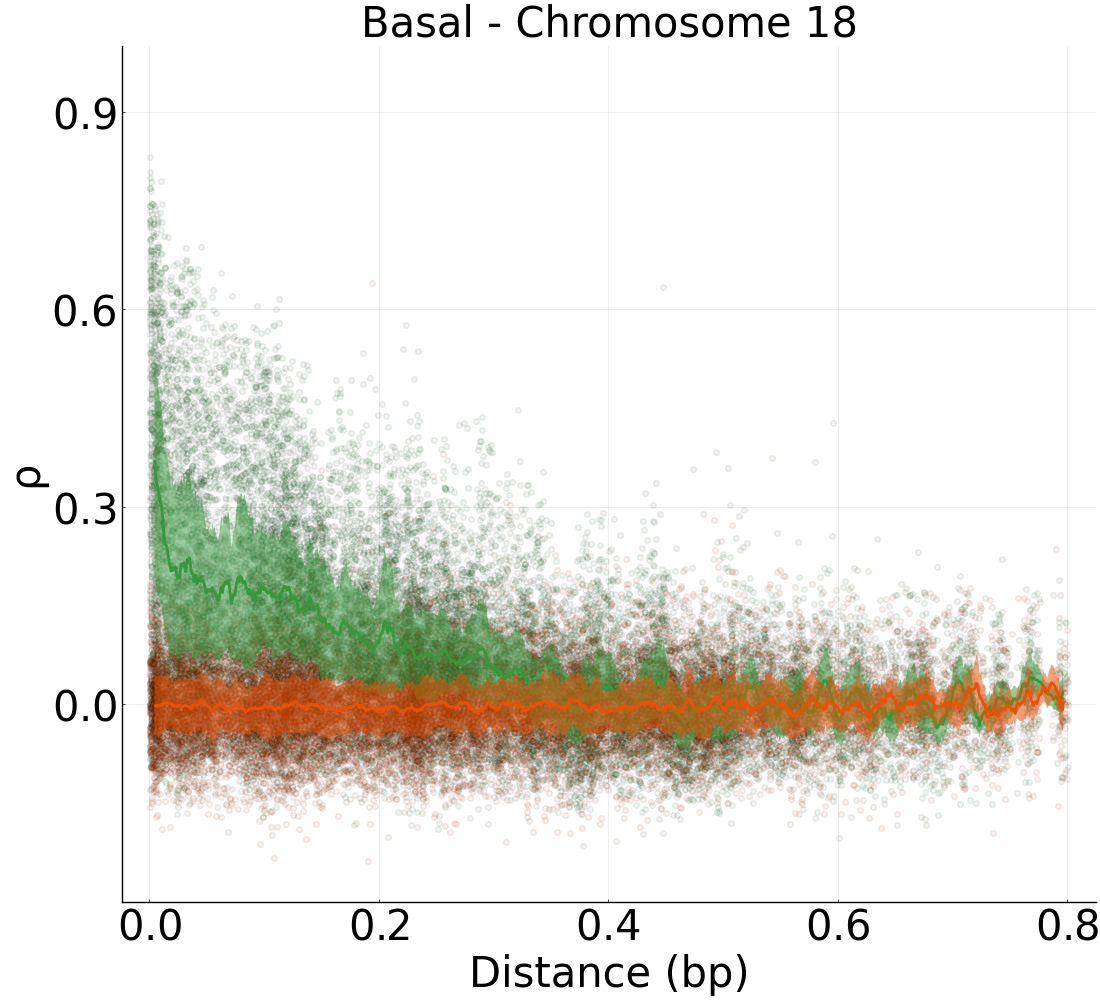

Supplement: Supplementary Material S7 to S11 — Pearson distribution scatter plots for normal adjacent tissue, Basal HER2+, Luminal A and Luminal B, respectively. These plots show correlations sorted by gene start position for the four cancer phenotypes and the adjacent normal network per chromosome. [file DataSheet_7.zip › SuppMat8Basal/Chromosome-18-Basal.png]

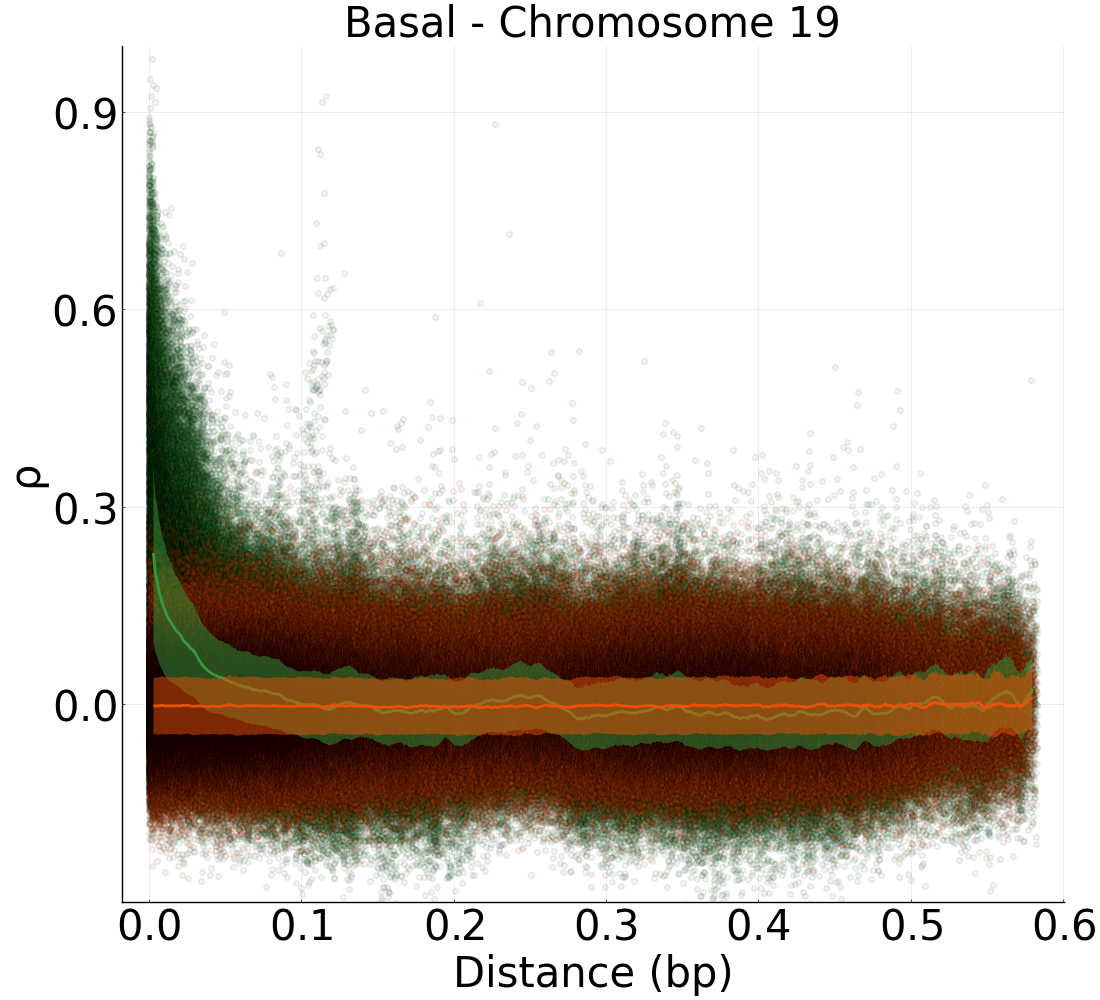

Supplement: Supplementary Material S7 to S11 — Pearson distribution scatter plots for normal adjacent tissue, Basal HER2+, Luminal A and Luminal B, respectively. These plots show correlations sorted by gene start position for the four cancer phenotypes and the adjacent normal network per chromosome. [file DataSheet_7.zip › SuppMat8Basal/Chromosome-19-Basal.png]

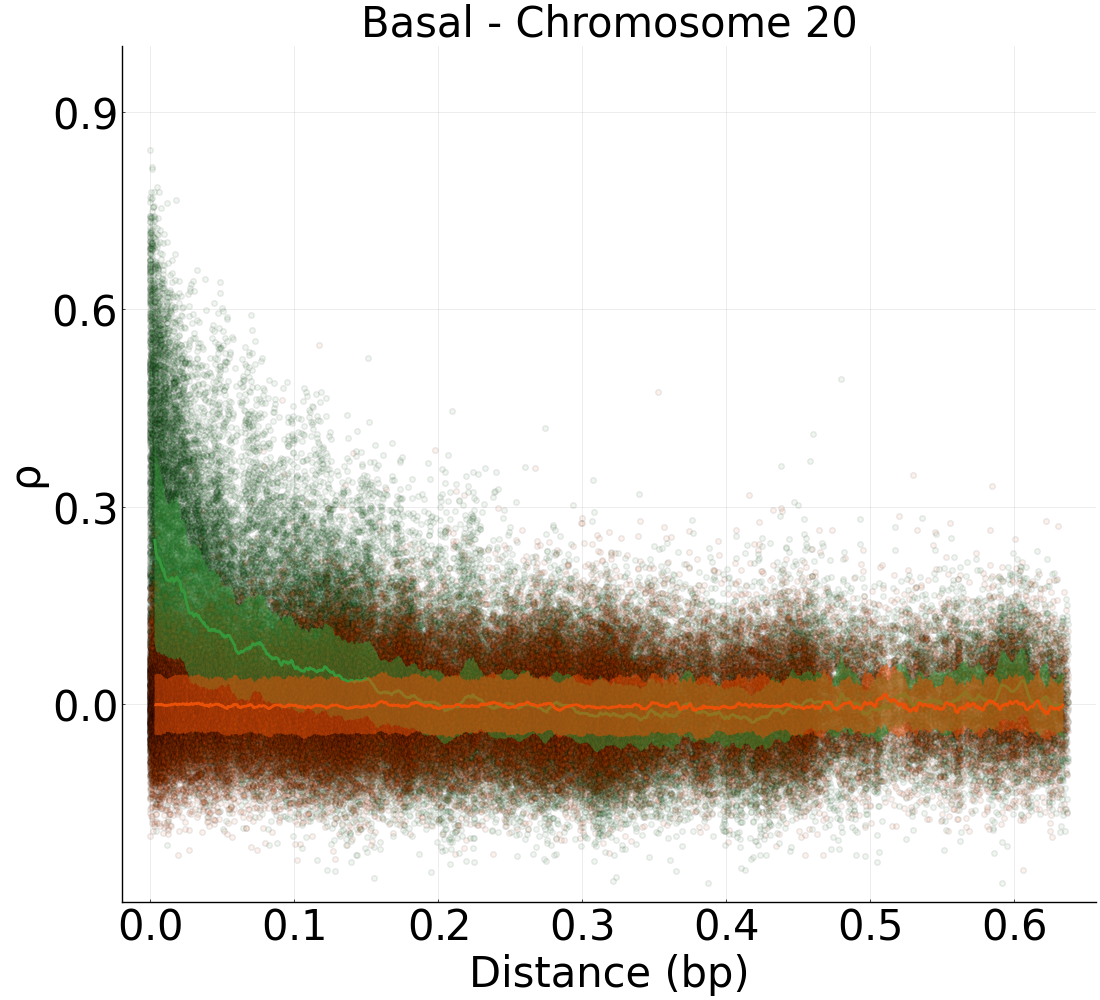

Supplement: Supplementary Material S7 to S11 — Pearson distribution scatter plots for normal adjacent tissue, Basal HER2+, Luminal A and Luminal B, respectively. These plots show correlations sorted by gene start position for the four cancer phenotypes and the adjacent normal network per chromosome. [file DataSheet_7.zip › SuppMat8Basal/Chromosome-20-Basal.png]

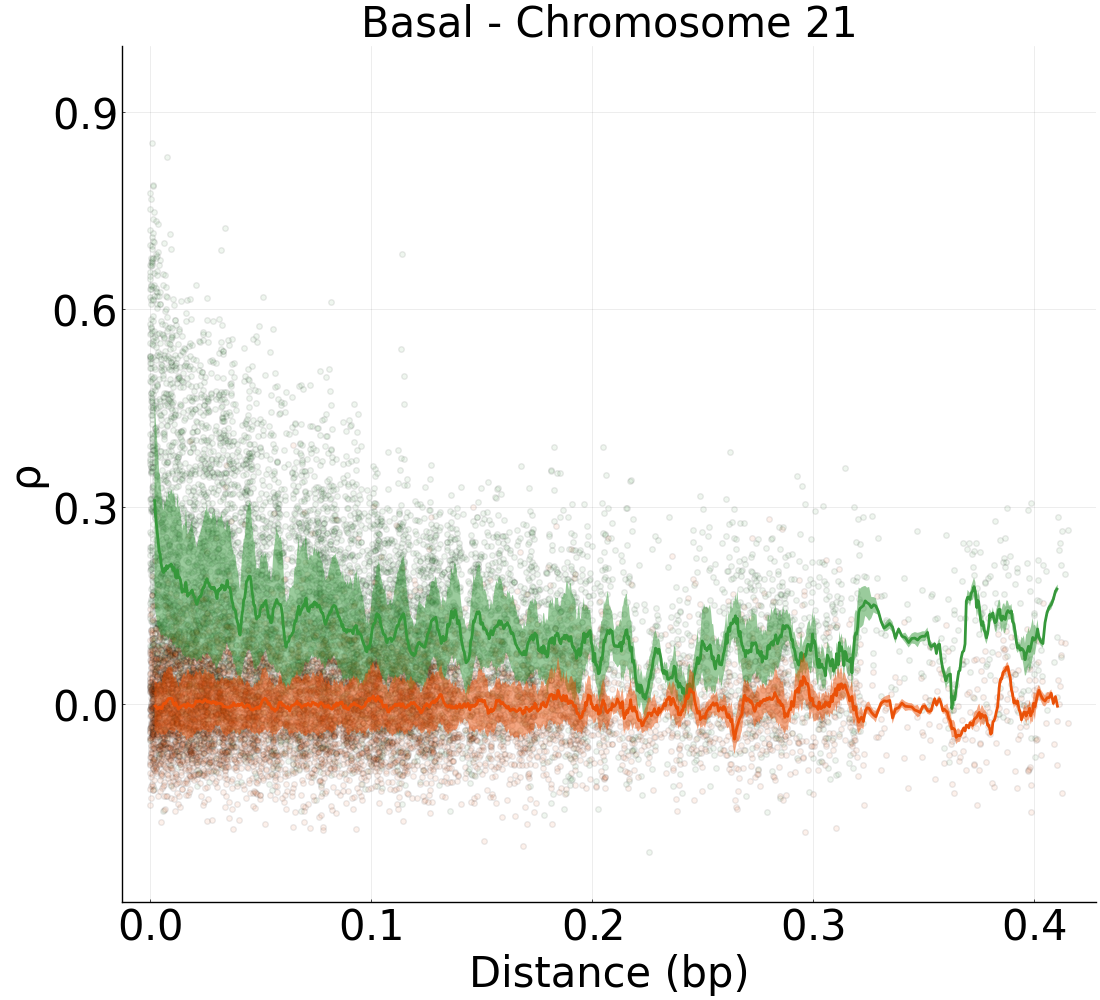

Supplement: Supplementary Material S7 to S11 — Pearson distribution scatter plots for normal adjacent tissue, Basal HER2+, Luminal A and Luminal B, respectively. These plots show correlations sorted by gene start position for the four cancer phenotypes and the adjacent normal network per chromosome. [file DataSheet_7.zip › SuppMat8Basal/Chromosome-21-Basal.png]

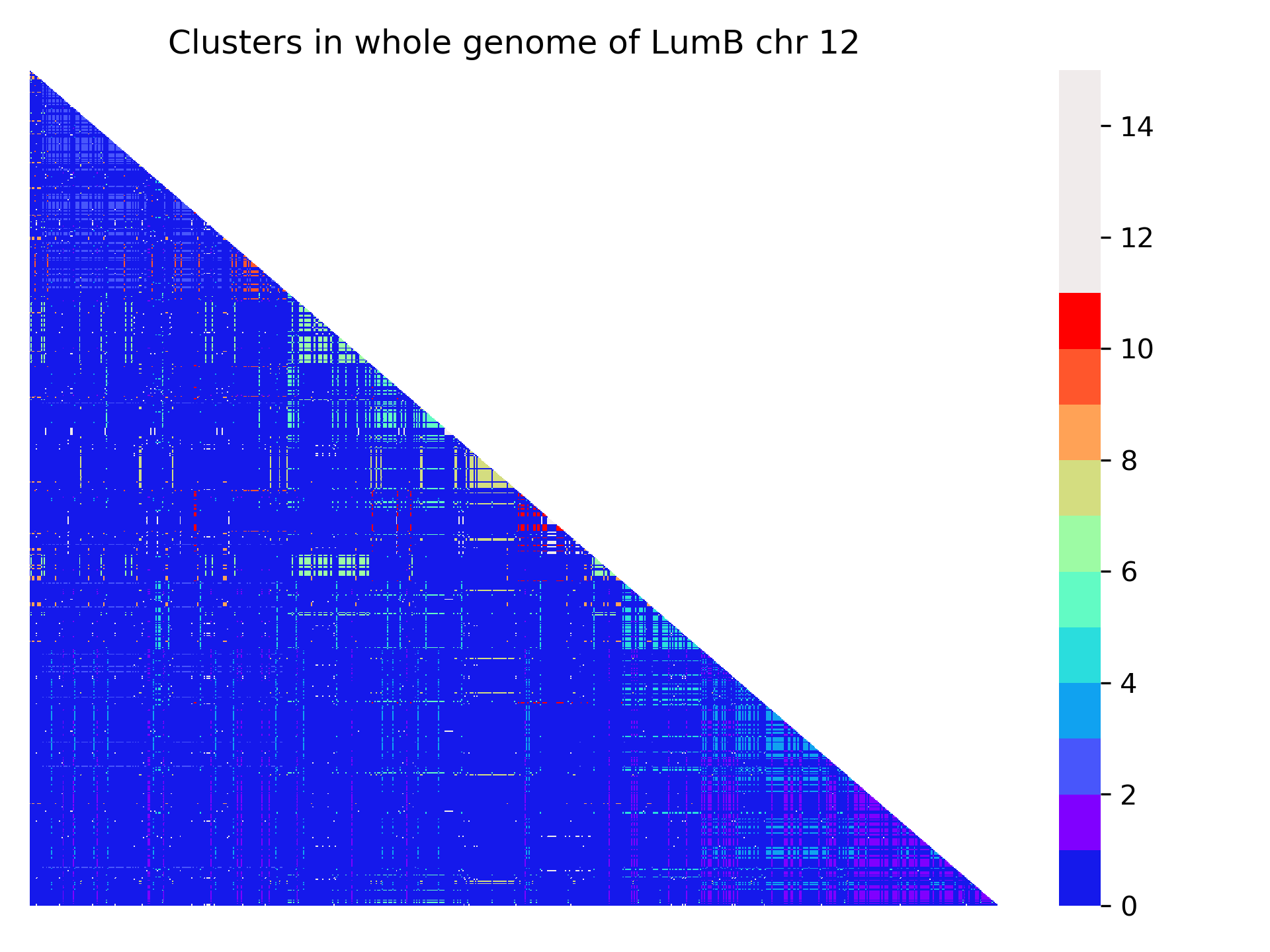

Supplement: Supplementary Material S13 — Piece-wise permutation p-values of the KS statistics, calculated for all bins obtained in Supplementary Material S8 , in every chromosomal region for each phenotype. [file DataSheet_13.zip › SuppMat10/SuppMat10/chr12/LumB-chr12-gstart-heat.png]

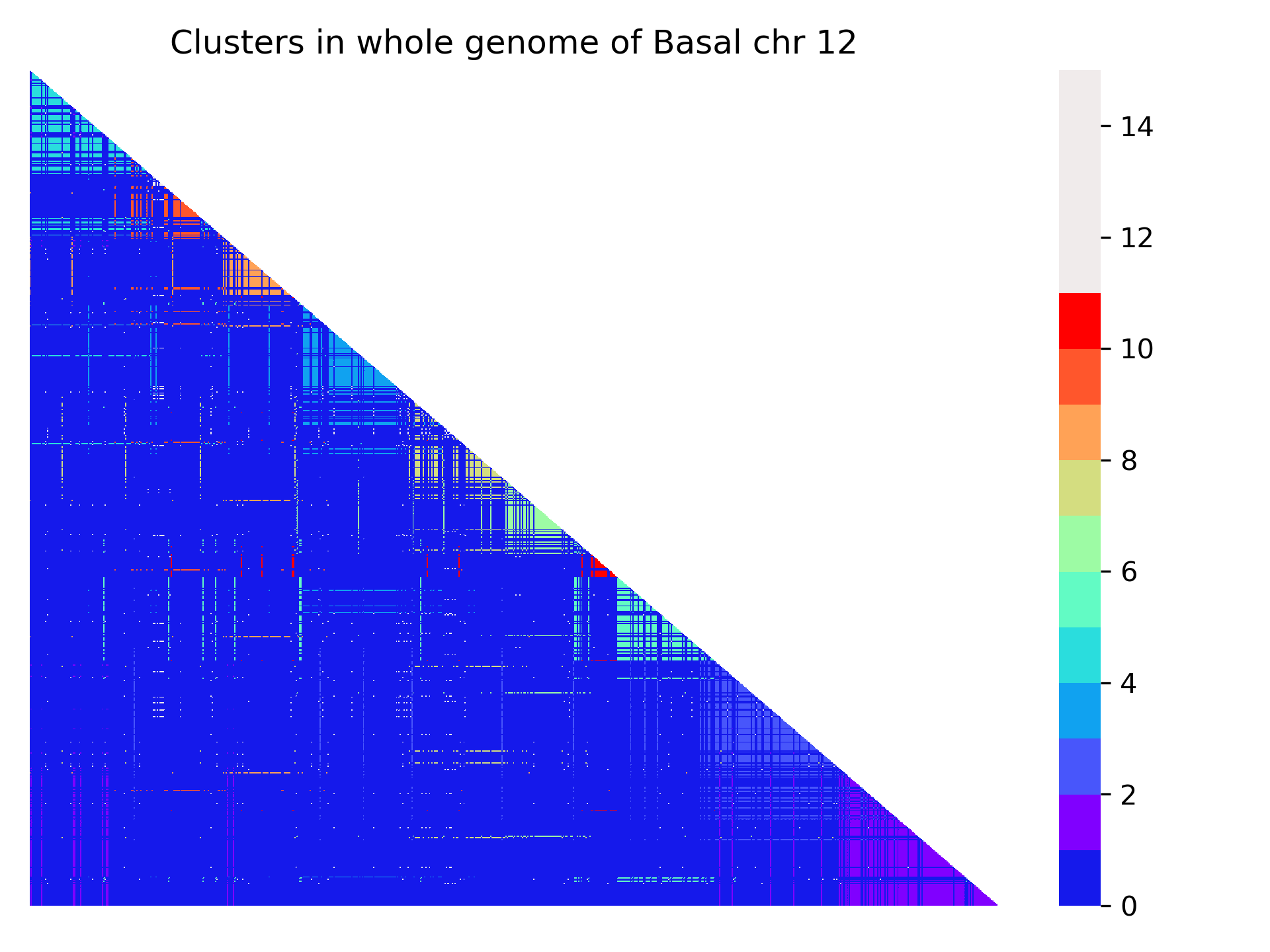

Supplement: Supplementary Material S13 — Piece-wise permutation p-values of the KS statistics, calculated for all bins obtained in Supplementary Material S8 , in every chromosomal region for each phenotype. [file DataSheet_13.zip › SuppMat10/SuppMat10/chr12/Basal-chr12-gstart-heat.png]

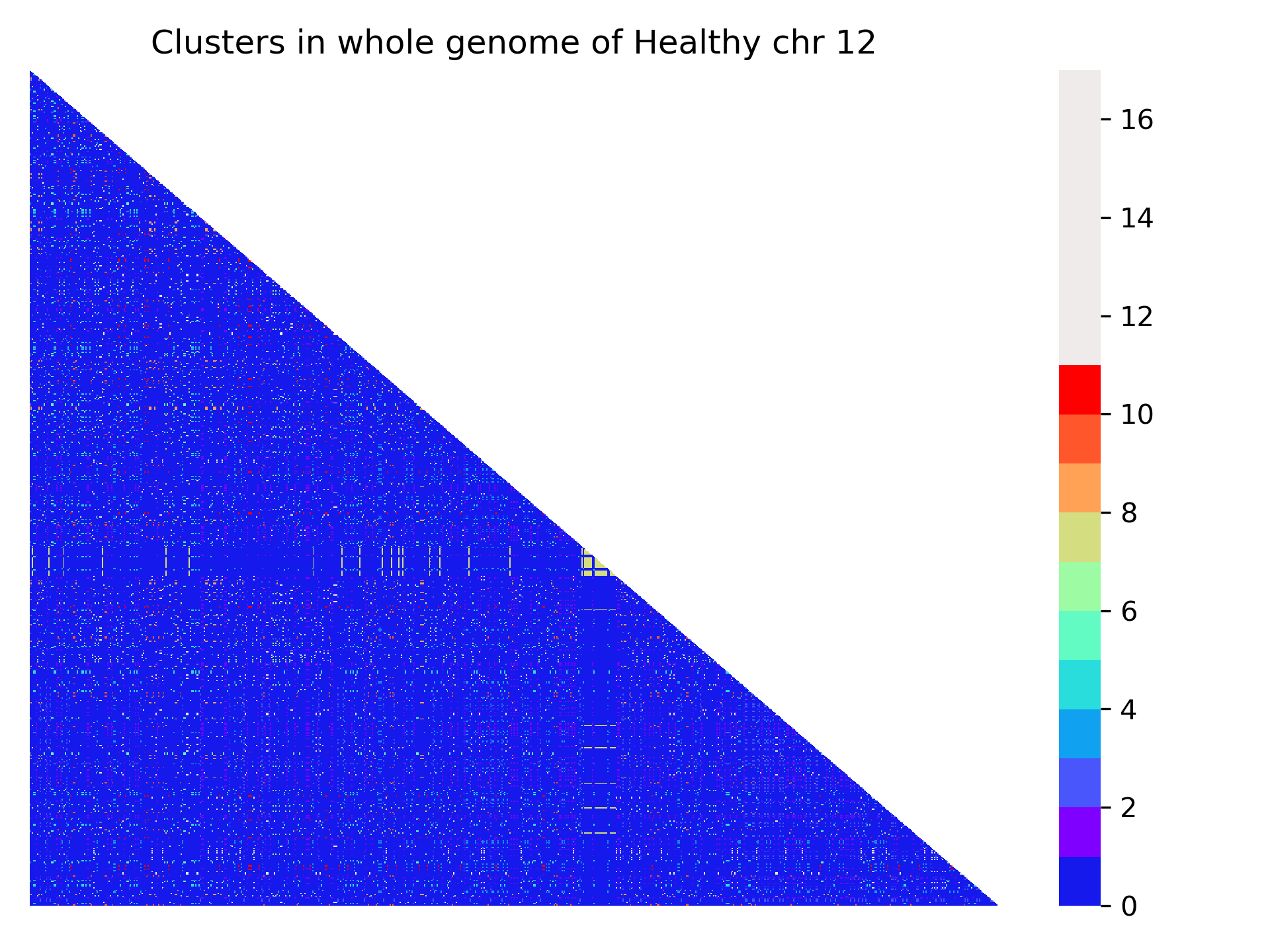

Supplement: Supplementary Material S13 — Piece-wise permutation p-values of the KS statistics, calculated for all bins obtained in Supplementary Material S8 , in every chromosomal region for each phenotype. [file DataSheet_13.zip › SuppMat10/SuppMat10/chr12/Healthy-chr12-gstart-heat.png]

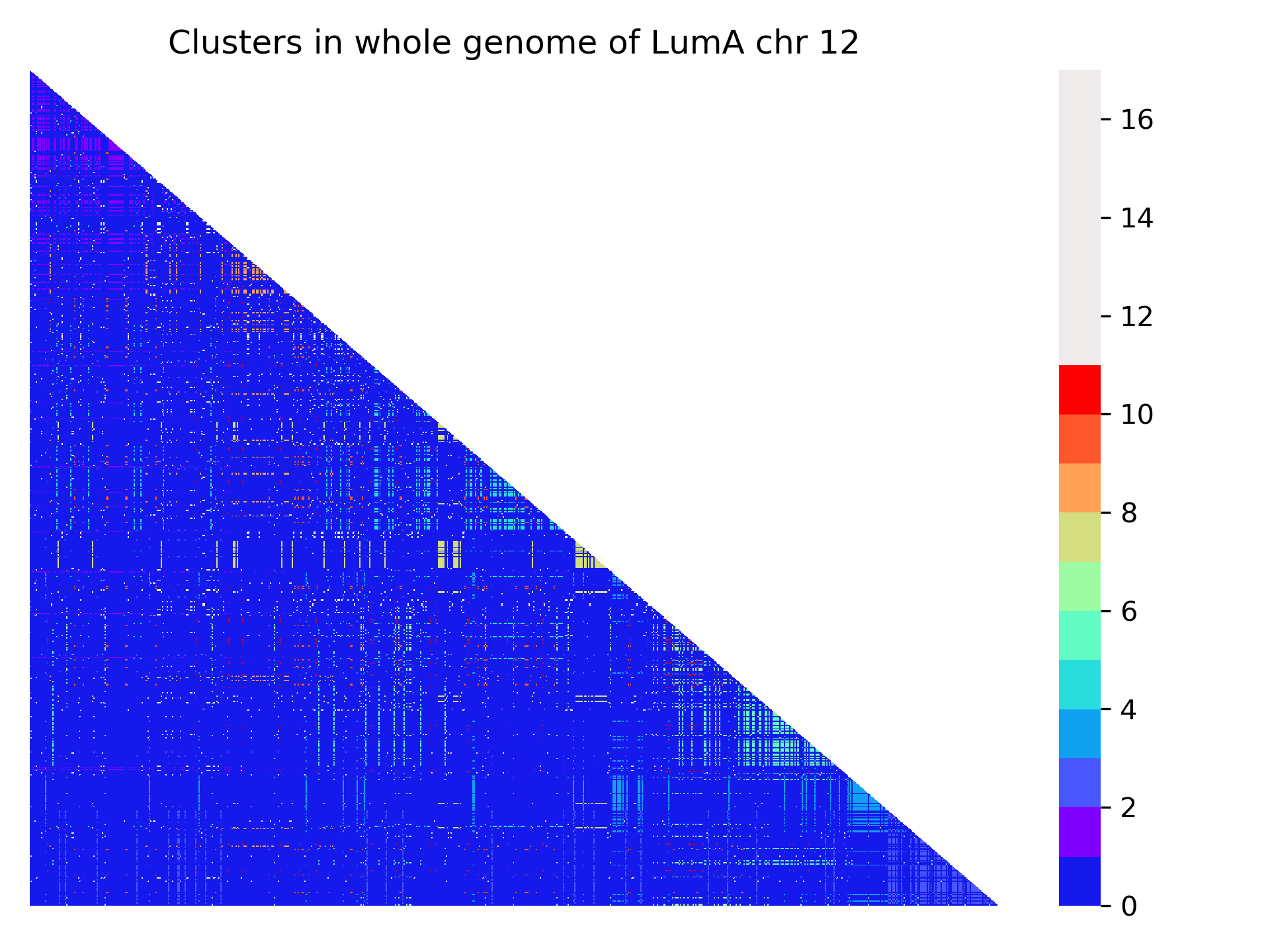

Supplement: Supplementary Material S13 — Piece-wise permutation p-values of the KS statistics, calculated for all bins obtained in Supplementary Material S8 , in every chromosomal region for each phenotype. [file DataSheet_13.zip › SuppMat10/SuppMat10/chr12/LumA-chr12-gstart-heat.png]

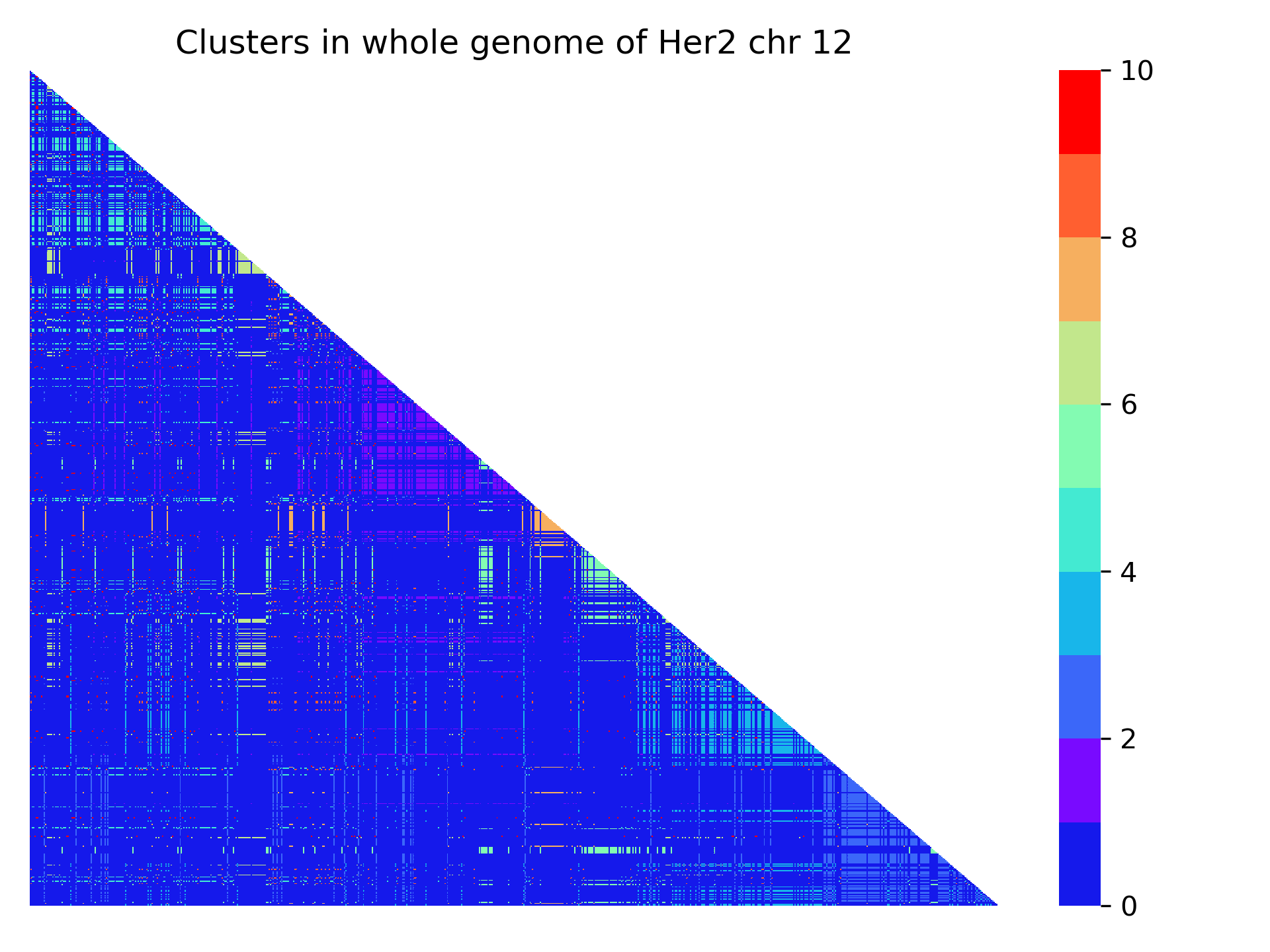

Supplement: Supplementary Material S13 — Piece-wise permutation p-values of the KS statistics, calculated for all bins obtained in Supplementary Material S8 , in every chromosomal region for each phenotype. [file DataSheet_13.zip › SuppMat10/SuppMat10/chr12/Her2-chr12-gstart-heat.png]

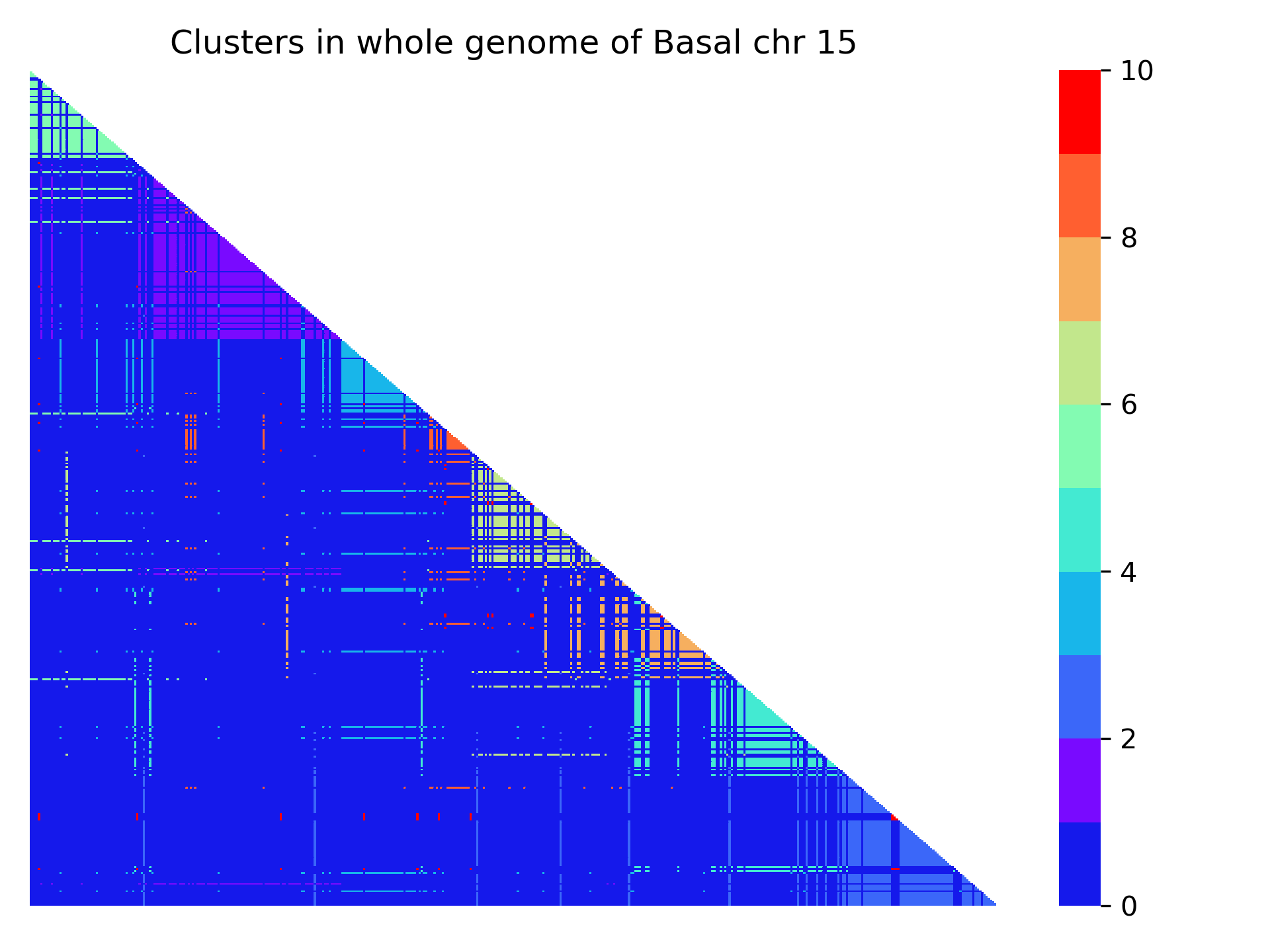

Supplement: Supplementary Material S13 — Piece-wise permutation p-values of the KS statistics, calculated for all bins obtained in Supplementary Material S8 , in every chromosomal region for each phenotype. [file DataSheet_13.zip › SuppMat10/SuppMat10/chr15/Basal-chr15-gstart-heat.png]

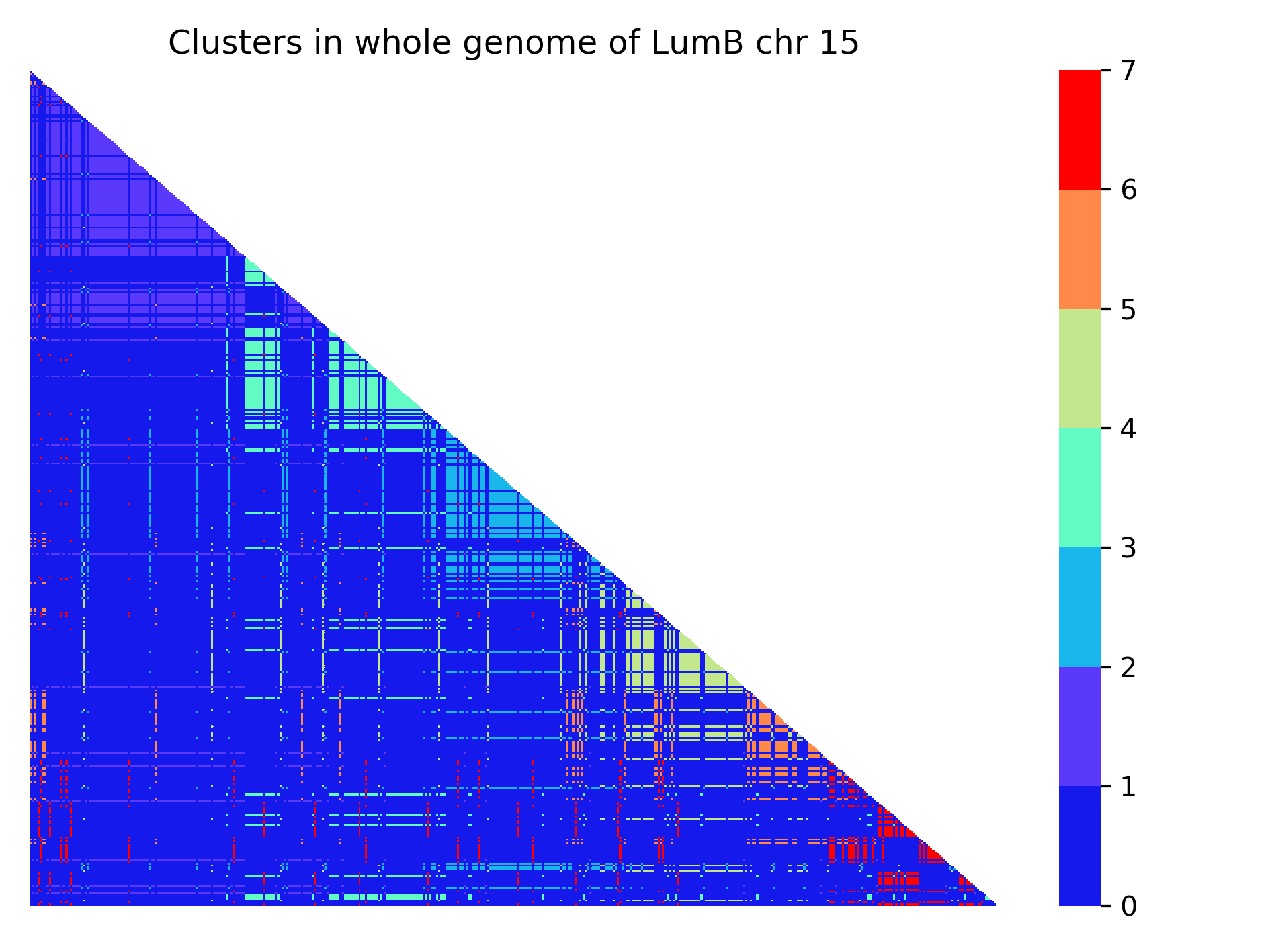

Supplement: Supplementary Material S13 — Piece-wise permutation p-values of the KS statistics, calculated for all bins obtained in Supplementary Material S8 , in every chromosomal region for each phenotype. [file DataSheet_13.zip › SuppMat10/SuppMat10/chr15/LumB-chr15-gstart-heat.png]

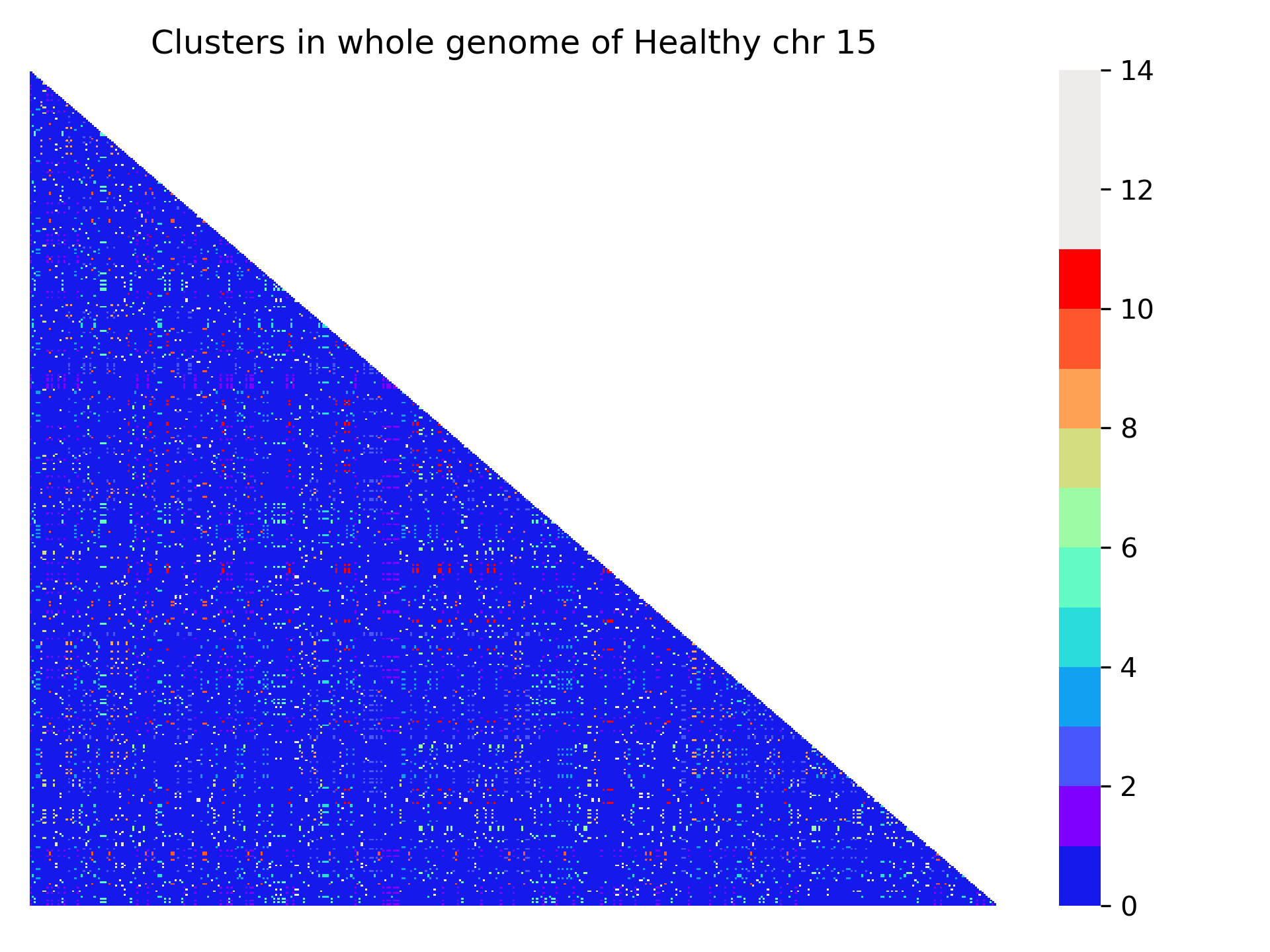

Supplement: Supplementary Material S13 — Piece-wise permutation p-values of the KS statistics, calculated for all bins obtained in Supplementary Material S8 , in every chromosomal region for each phenotype. [file DataSheet_13.zip › SuppMat10/SuppMat10/chr15/Healthy-chr15-gstart-heat.png]

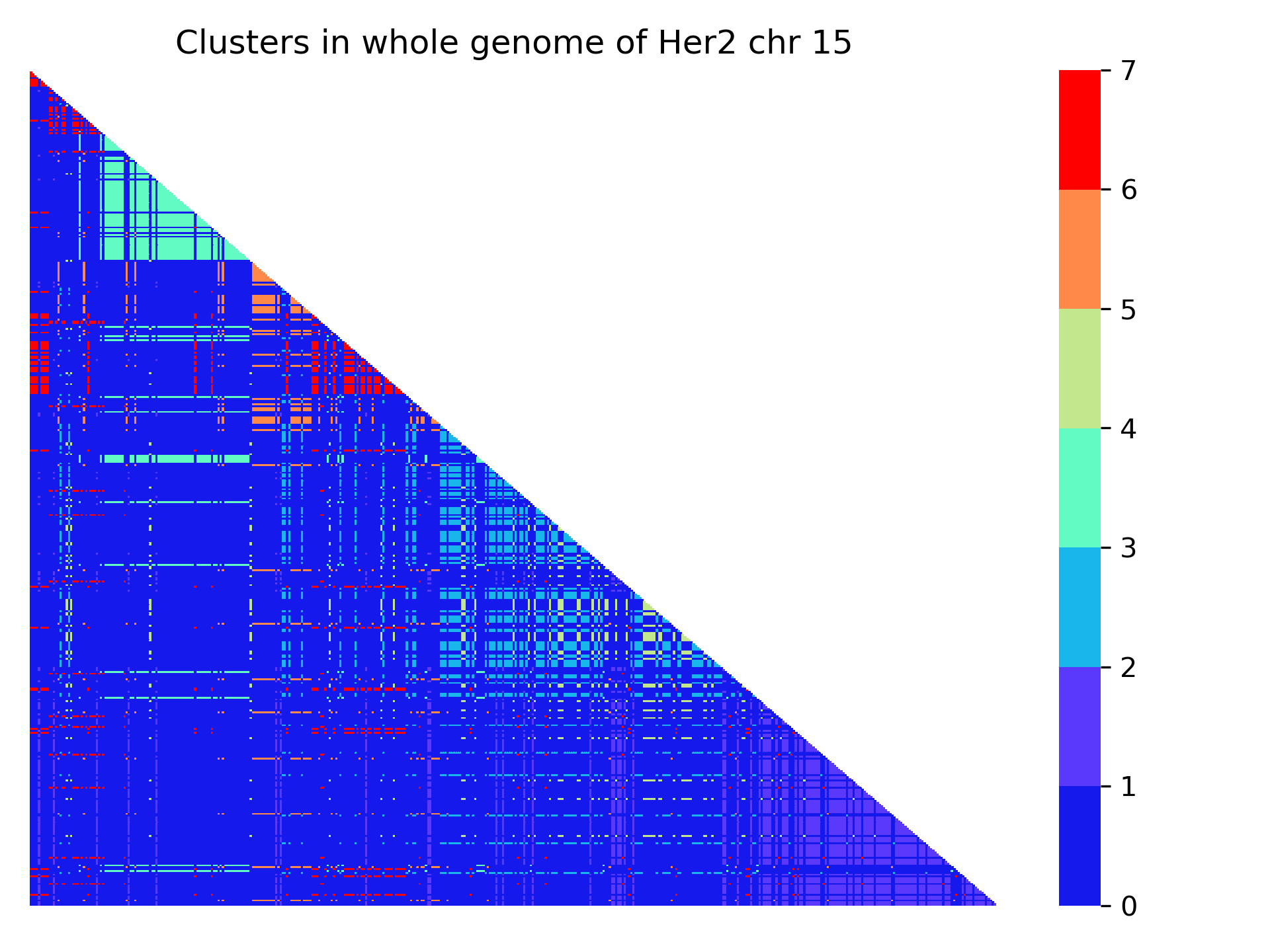

Supplement: Supplementary Material S13 — Piece-wise permutation p-values of the KS statistics, calculated for all bins obtained in Supplementary Material S8 , in every chromosomal region for each phenotype. [file DataSheet_13.zip › SuppMat10/SuppMat10/chr15/Her2-chr15-gstart-heat.png]

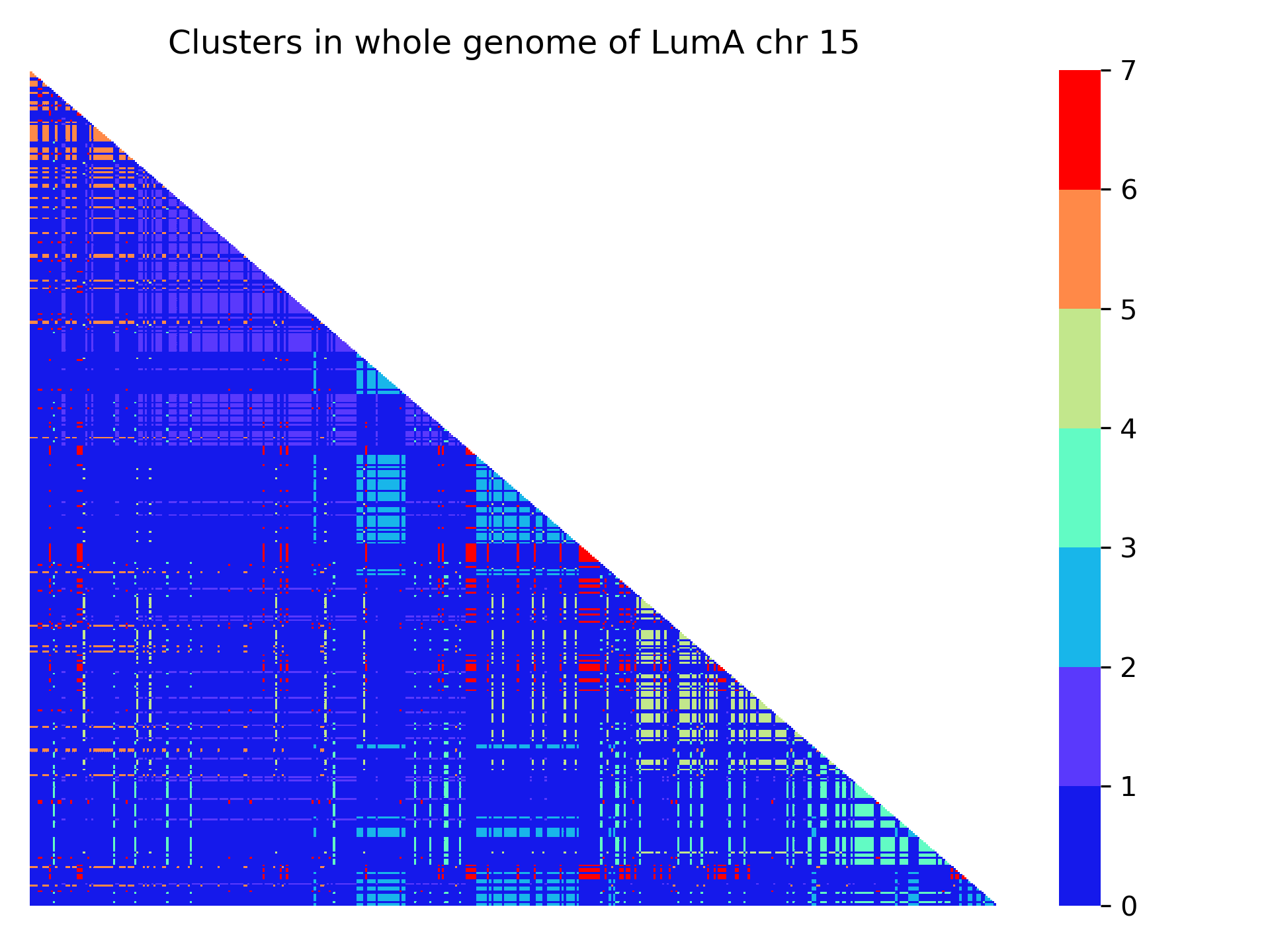

Supplement: Supplementary Material S13 — Piece-wise permutation p-values of the KS statistics, calculated for all bins obtained in Supplementary Material S8 , in every chromosomal region for each phenotype. [file DataSheet_13.zip › SuppMat10/SuppMat10/chr15/LumA-chr15-gstart-heat.png]

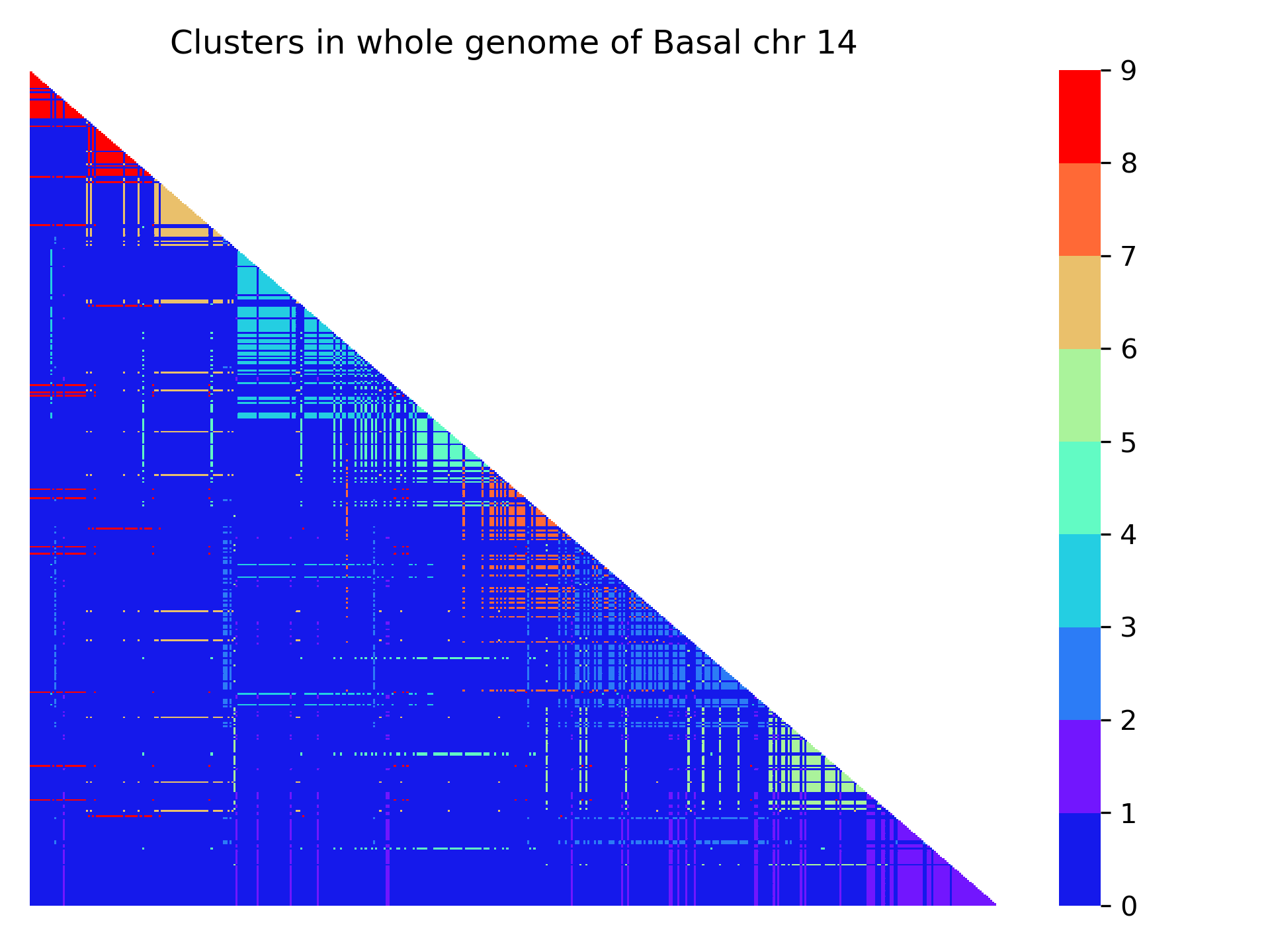

Supplement: Supplementary Material S13 — Piece-wise permutation p-values of the KS statistics, calculated for all bins obtained in Supplementary Material S8 , in every chromosomal region for each phenotype. [file DataSheet_13.zip › SuppMat10/SuppMat10/chr14/Basal-chr14-gstart-heat.png]

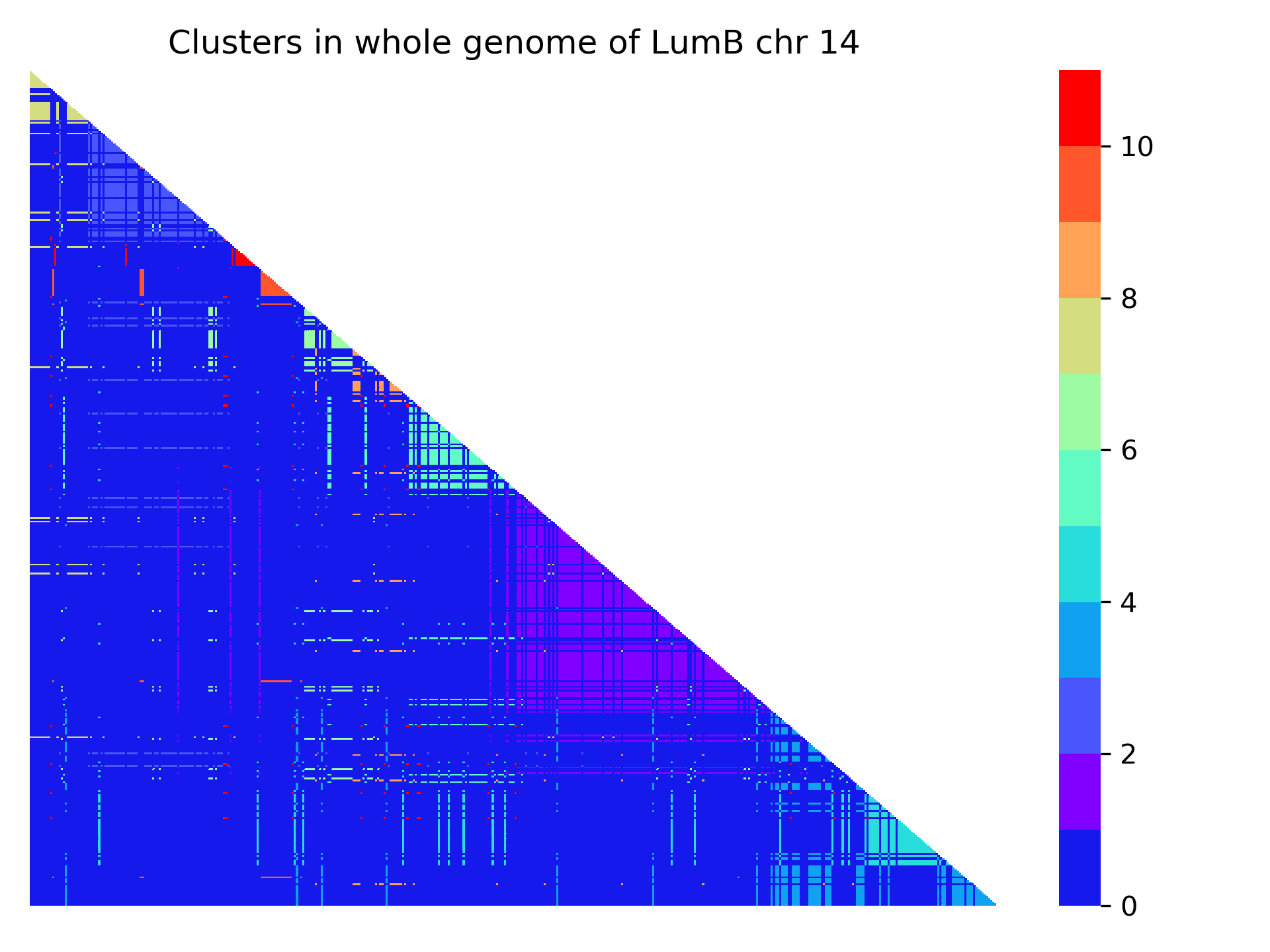

Supplement: Supplementary Material S13 — Piece-wise permutation p-values of the KS statistics, calculated for all bins obtained in Supplementary Material S8 , in every chromosomal region for each phenotype. [file DataSheet_13.zip › SuppMat10/SuppMat10/chr14/LumB-chr14-gstart-heat.png]

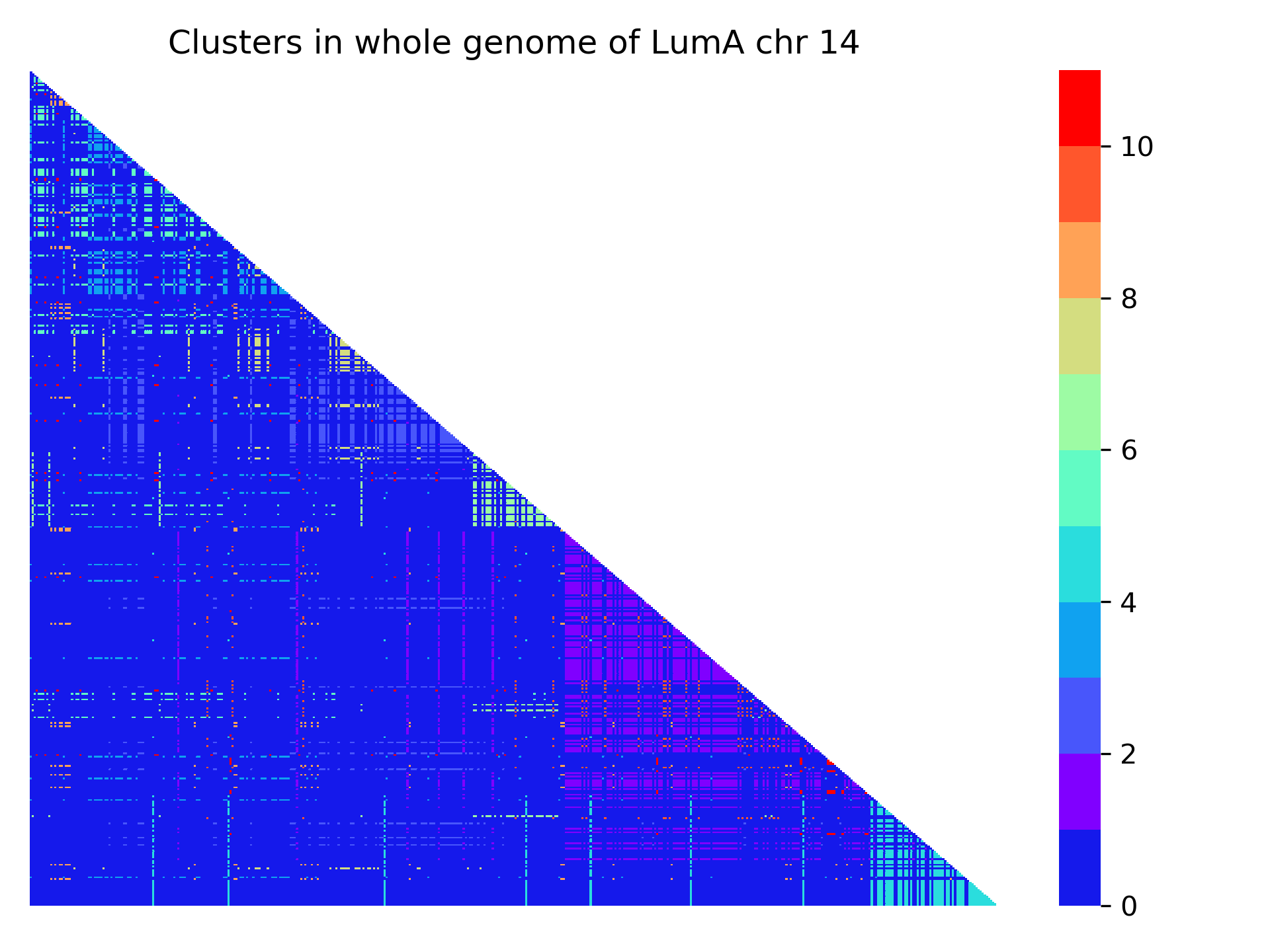

Supplement: Supplementary Material S13 — Piece-wise permutation p-values of the KS statistics, calculated for all bins obtained in Supplementary Material S8 , in every chromosomal region for each phenotype. [file DataSheet_13.zip › SuppMat10/SuppMat10/chr14/LumA-chr14-gstart-heat.png]

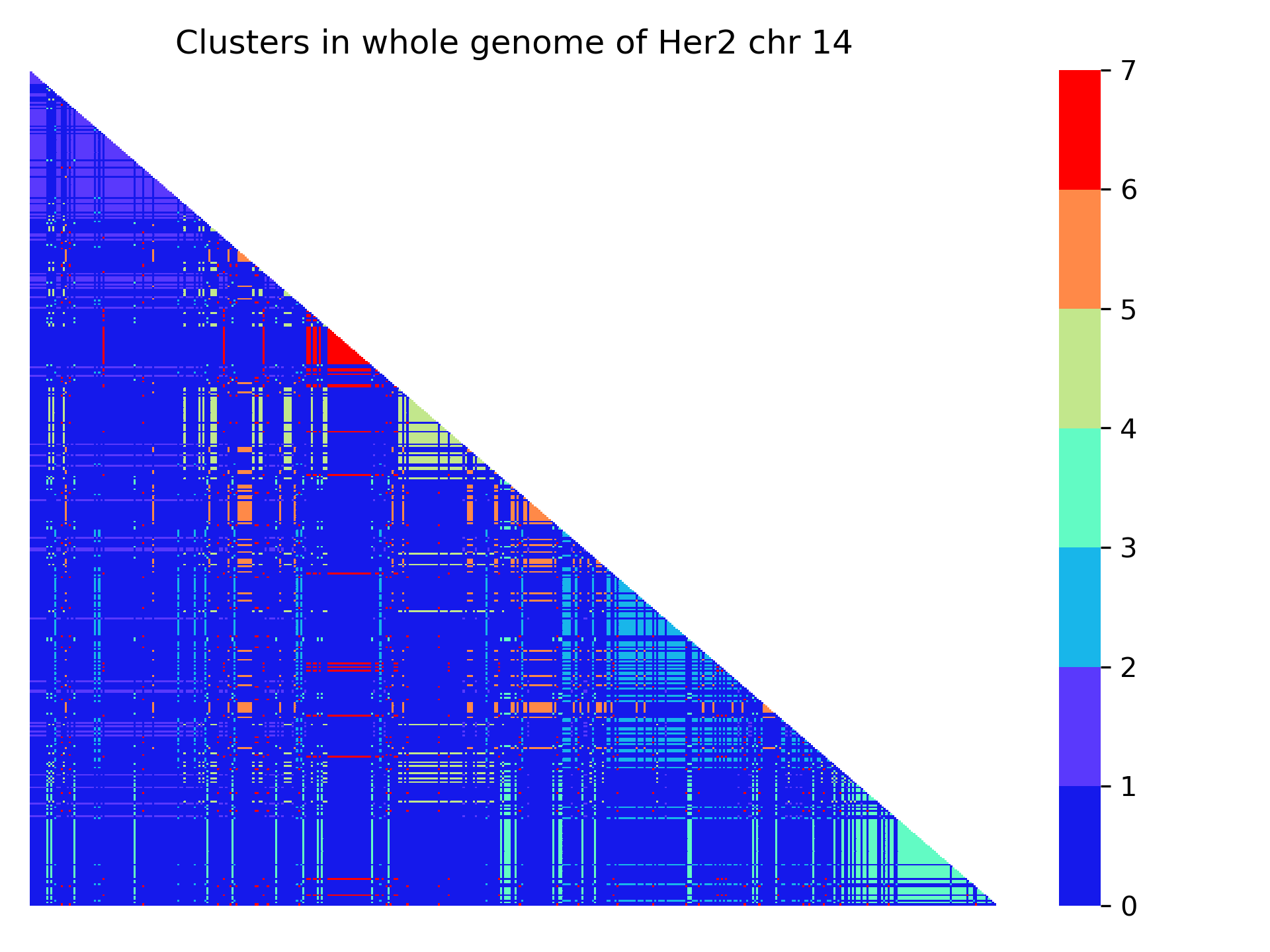

Supplement: Supplementary Material S13 — Piece-wise permutation p-values of the KS statistics, calculated for all bins obtained in Supplementary Material S8 , in every chromosomal region for each phenotype. [file DataSheet_13.zip › SuppMat10/SuppMat10/chr14/Her2-chr14-gstart-heat.png]

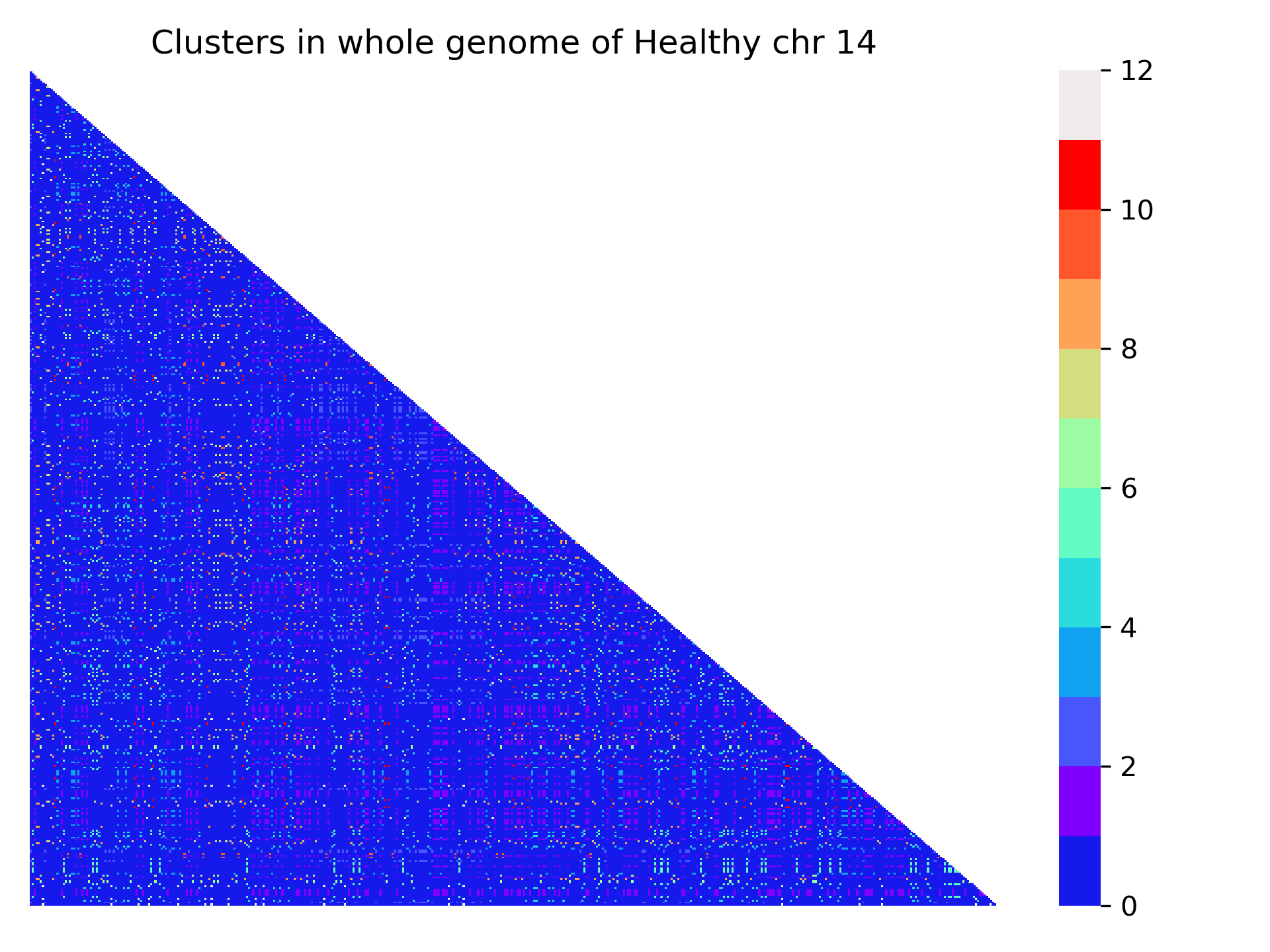

Supplement: Supplementary Material S13 — Piece-wise permutation p-values of the KS statistics, calculated for all bins obtained in Supplementary Material S8 , in every chromosomal region for each phenotype. [file DataSheet_13.zip › SuppMat10/SuppMat10/chr14/Healthy-chr14-gstart-heat.png]

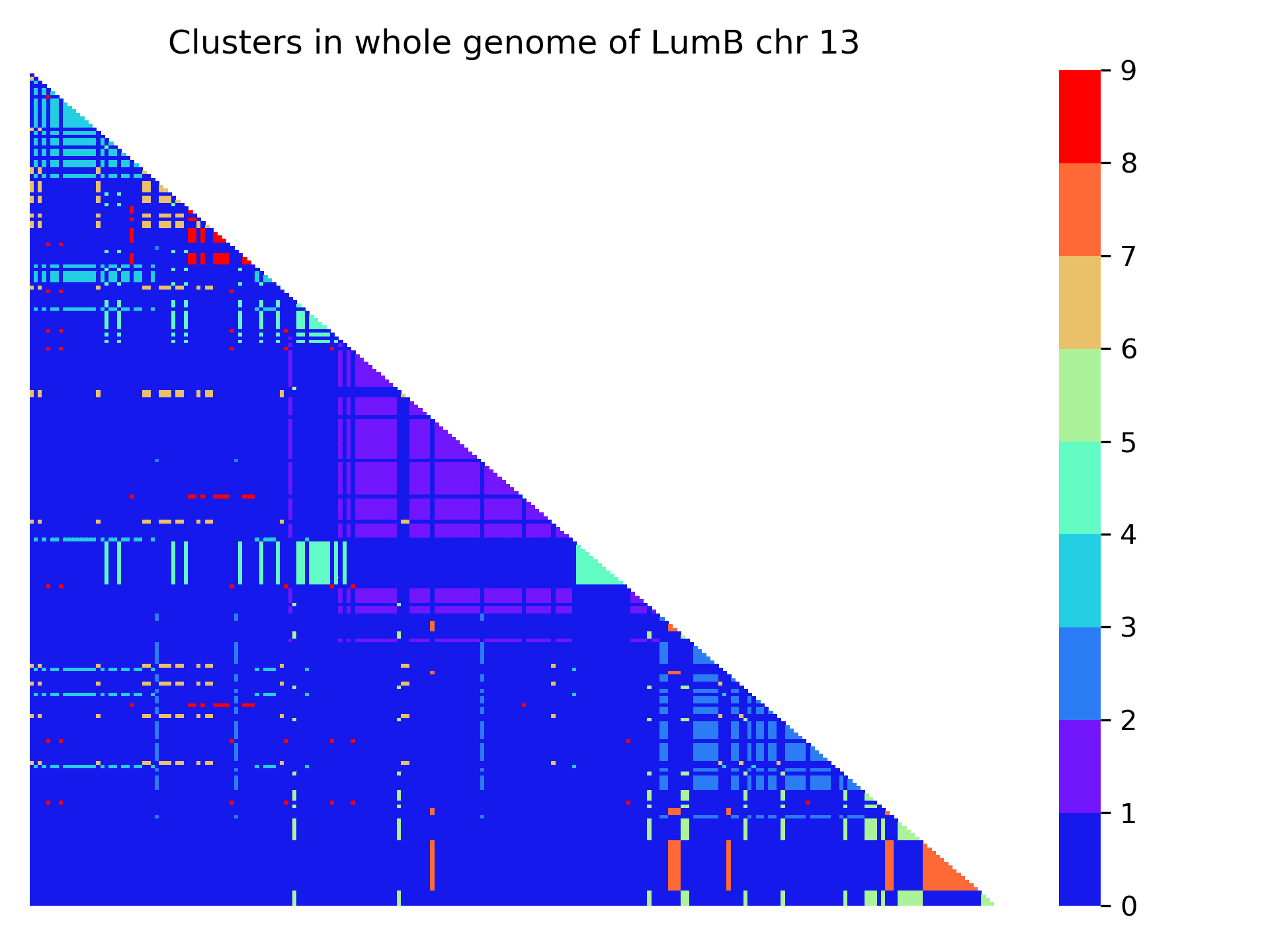

Supplement: Supplementary Material S13 — Piece-wise permutation p-values of the KS statistics, calculated for all bins obtained in Supplementary Material S8 , in every chromosomal region for each phenotype. [file DataSheet_13.zip › SuppMat10/SuppMat10/chr13/LumB-chr13-gstart-heat.png]

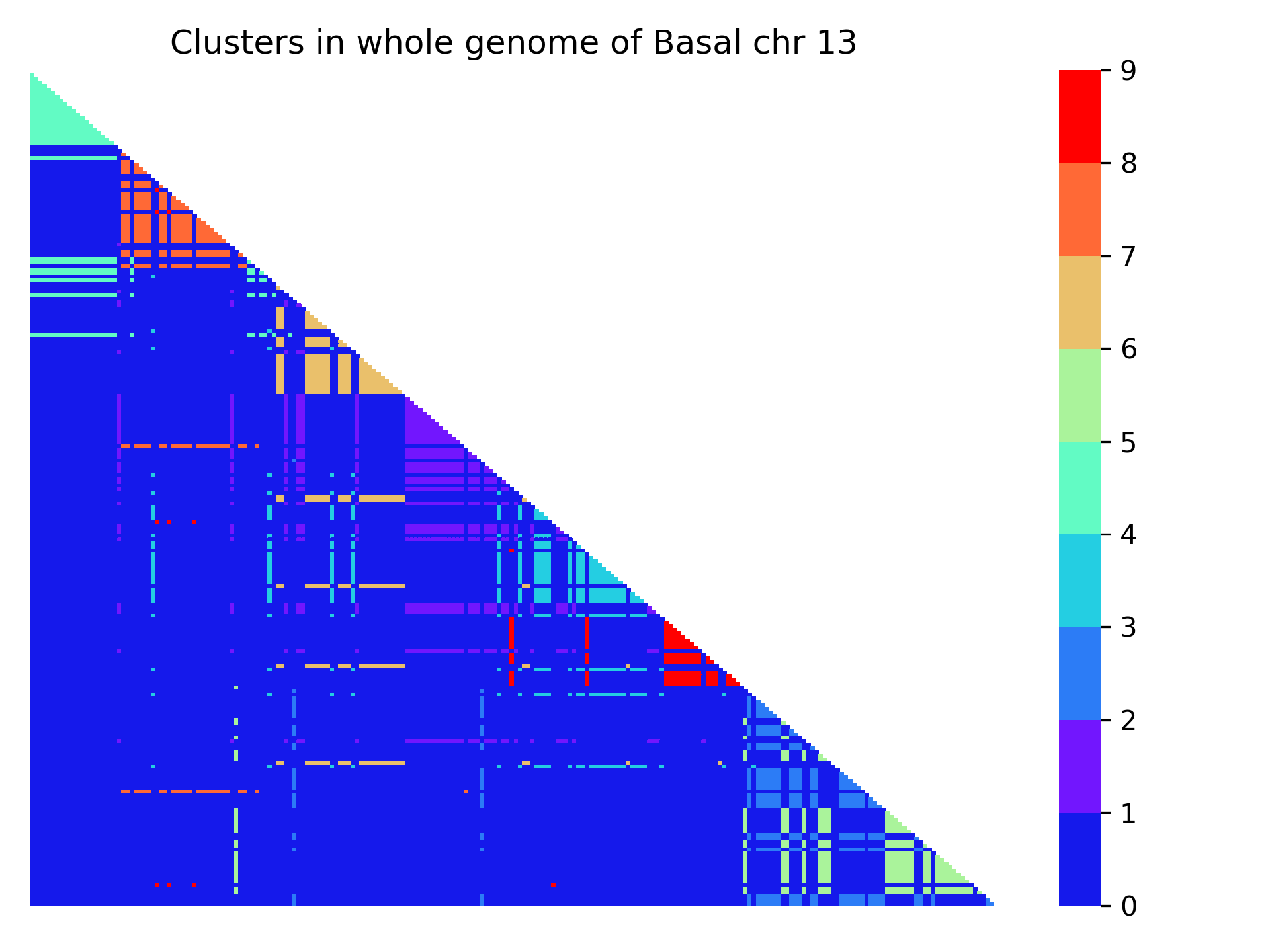

Supplement: Supplementary Material S13 — Piece-wise permutation p-values of the KS statistics, calculated for all bins obtained in Supplementary Material S8 , in every chromosomal region for each phenotype. [file DataSheet_13.zip › SuppMat10/SuppMat10/chr13/Basal-chr13-gstart-heat.png]

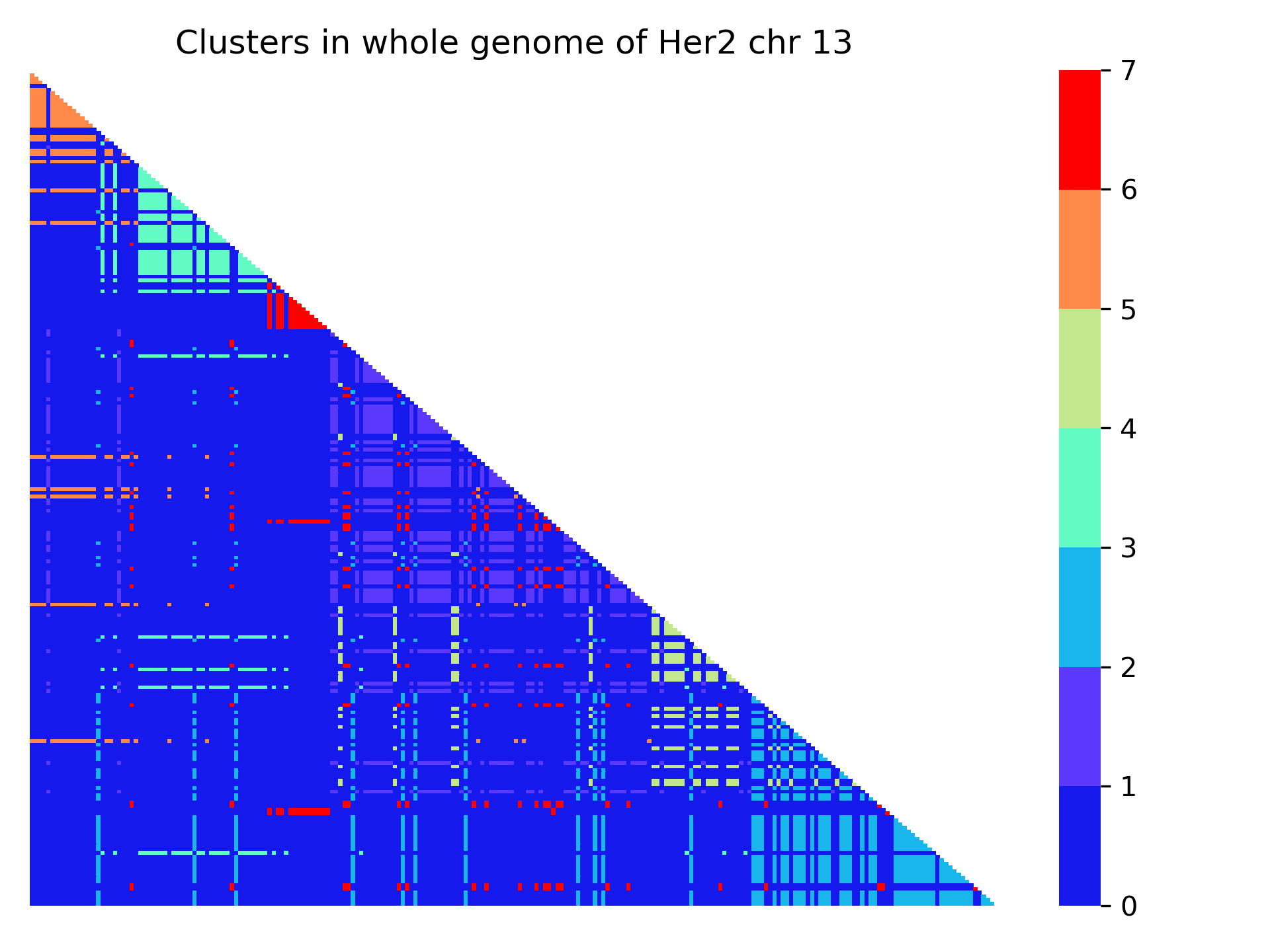

Supplement: Supplementary Material S13 — Piece-wise permutation p-values of the KS statistics, calculated for all bins obtained in Supplementary Material S8 , in every chromosomal region for each phenotype. [file DataSheet_13.zip › SuppMat10/SuppMat10/chr13/Her2-chr13-gstart-heat.png]

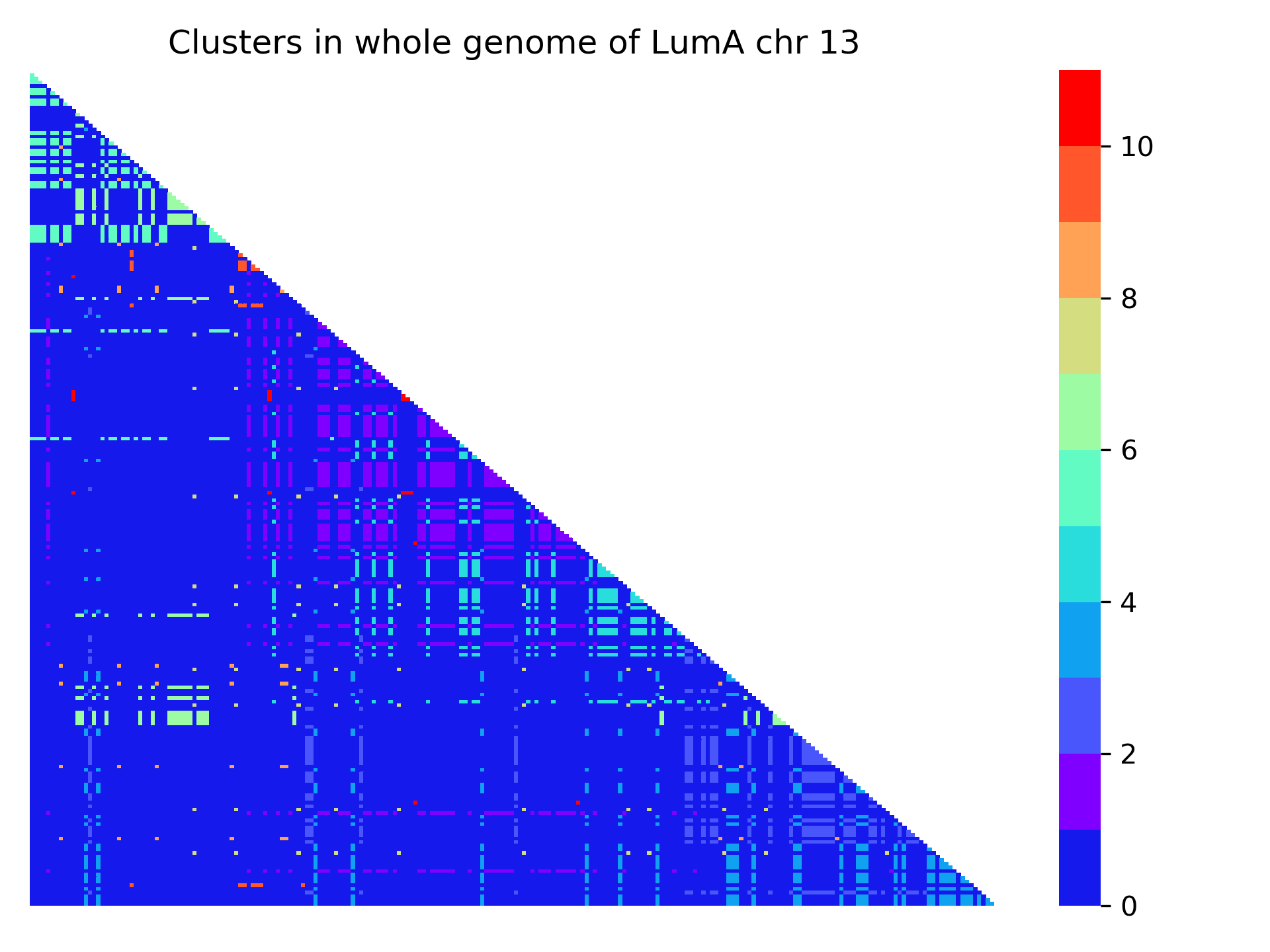

Supplement: Supplementary Material S13 — Piece-wise permutation p-values of the KS statistics, calculated for all bins obtained in Supplementary Material S8 , in every chromosomal region for each phenotype. [file DataSheet_13.zip › SuppMat10/SuppMat10/chr13/LumA-chr13-gstart-heat.png]

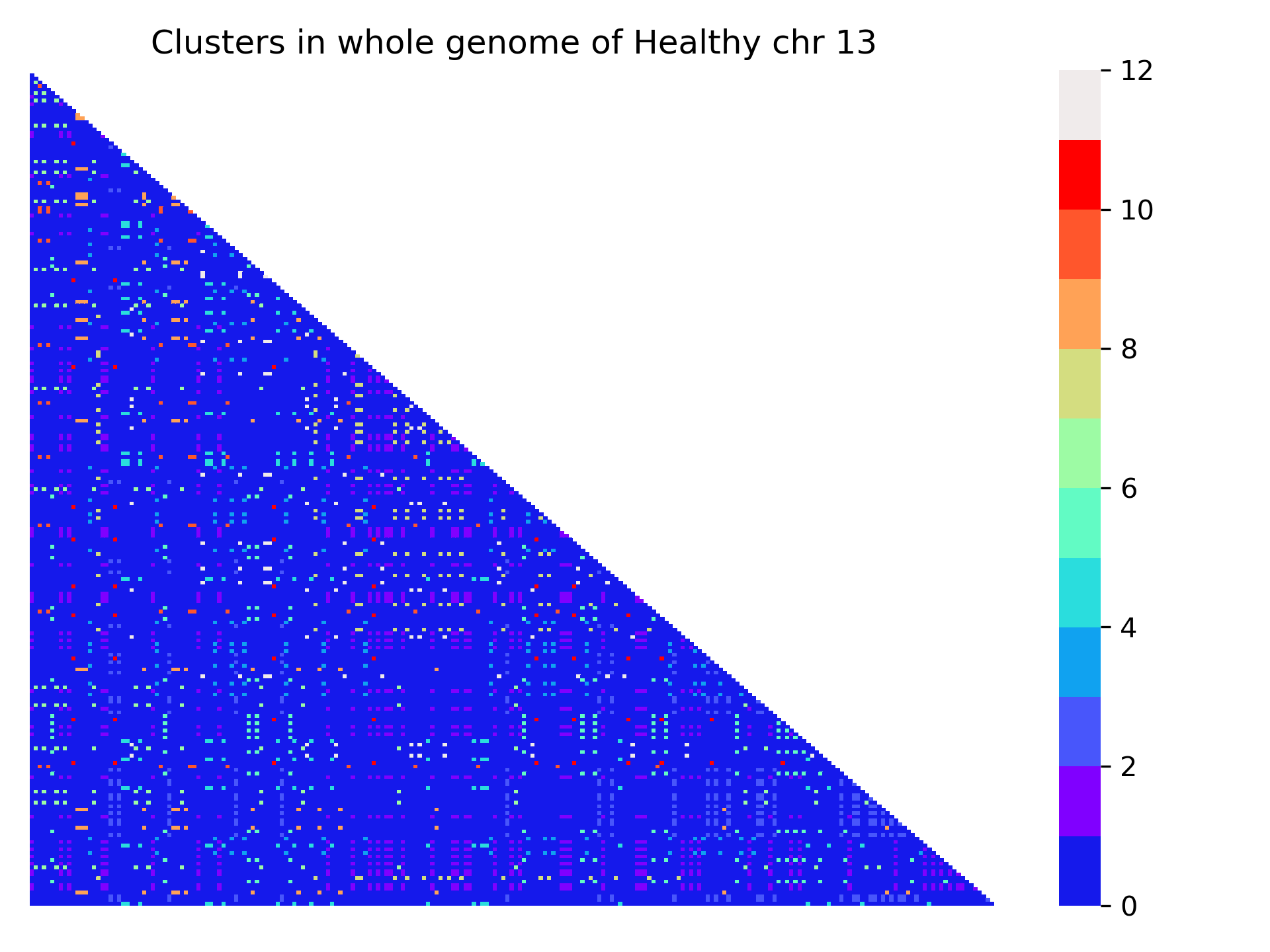

Supplement: Supplementary Material S13 — Piece-wise permutation p-values of the KS statistics, calculated for all bins obtained in Supplementary Material S8 , in every chromosomal region for each phenotype. [file DataSheet_13.zip › SuppMat10/SuppMat10/chr13/Healthy-chr13-gstart-heat.png]

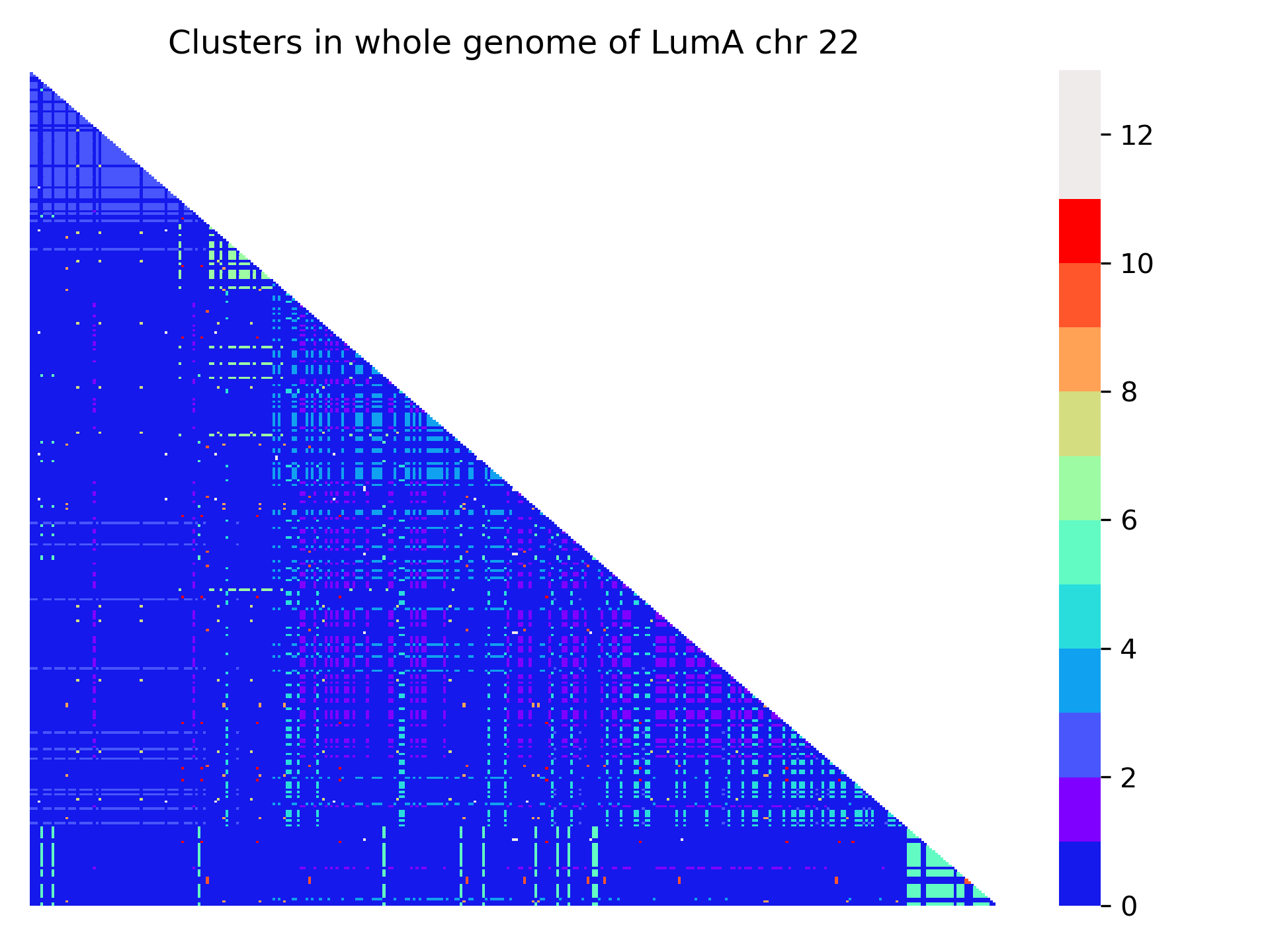

Supplement: Supplementary Material S13 — Piece-wise permutation p-values of the KS statistics, calculated for all bins obtained in Supplementary Material S8 , in every chromosomal region for each phenotype. [file DataSheet_13.zip › SuppMat10/SuppMat10/chr22/LumA-chr22-gstart-heat.png]

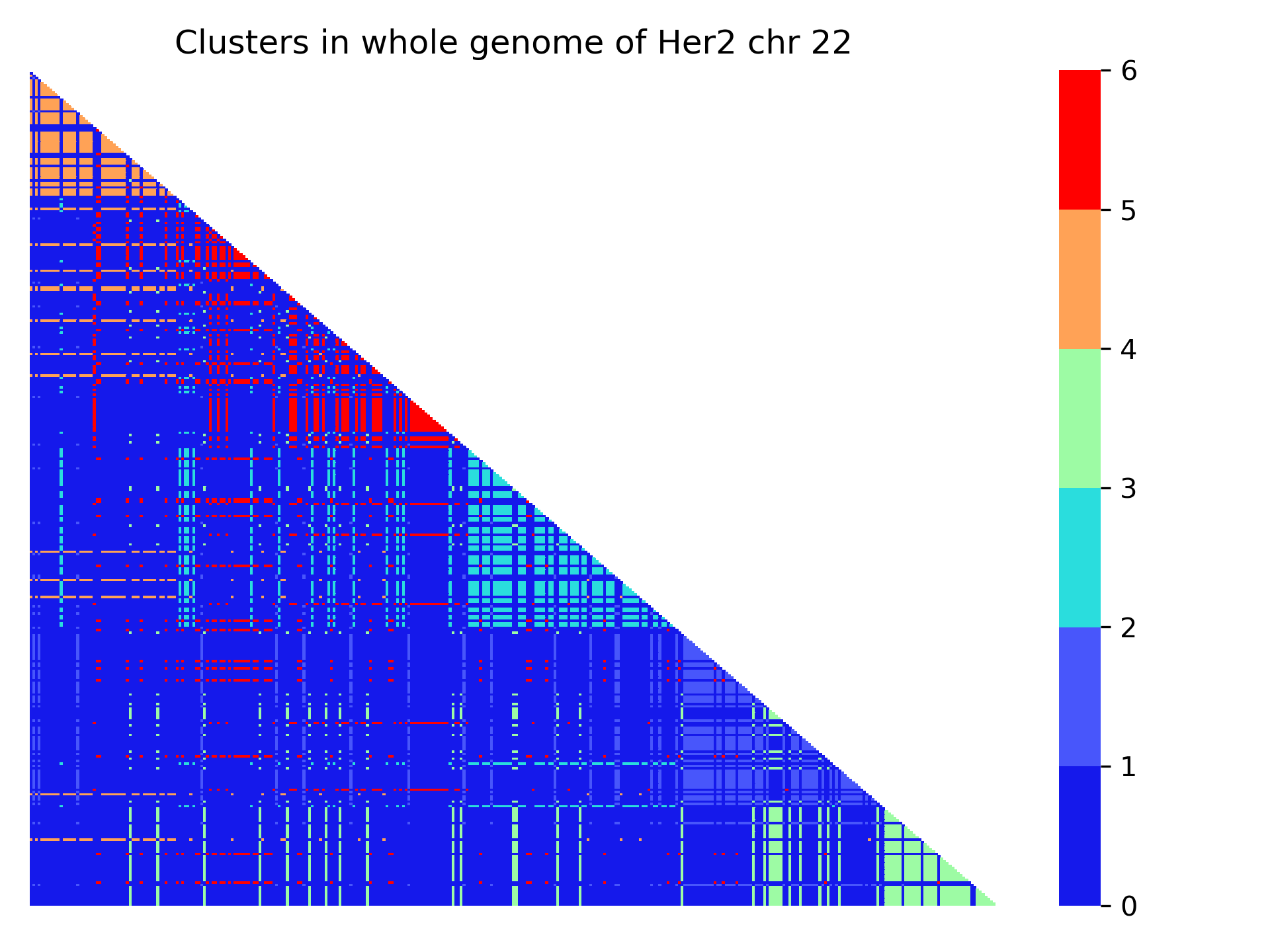

Supplement: Supplementary Material S13 — Piece-wise permutation p-values of the KS statistics, calculated for all bins obtained in Supplementary Material S8 , in every chromosomal region for each phenotype. [file DataSheet_13.zip › SuppMat10/SuppMat10/chr22/Her2-chr22-gstart-heat.png]

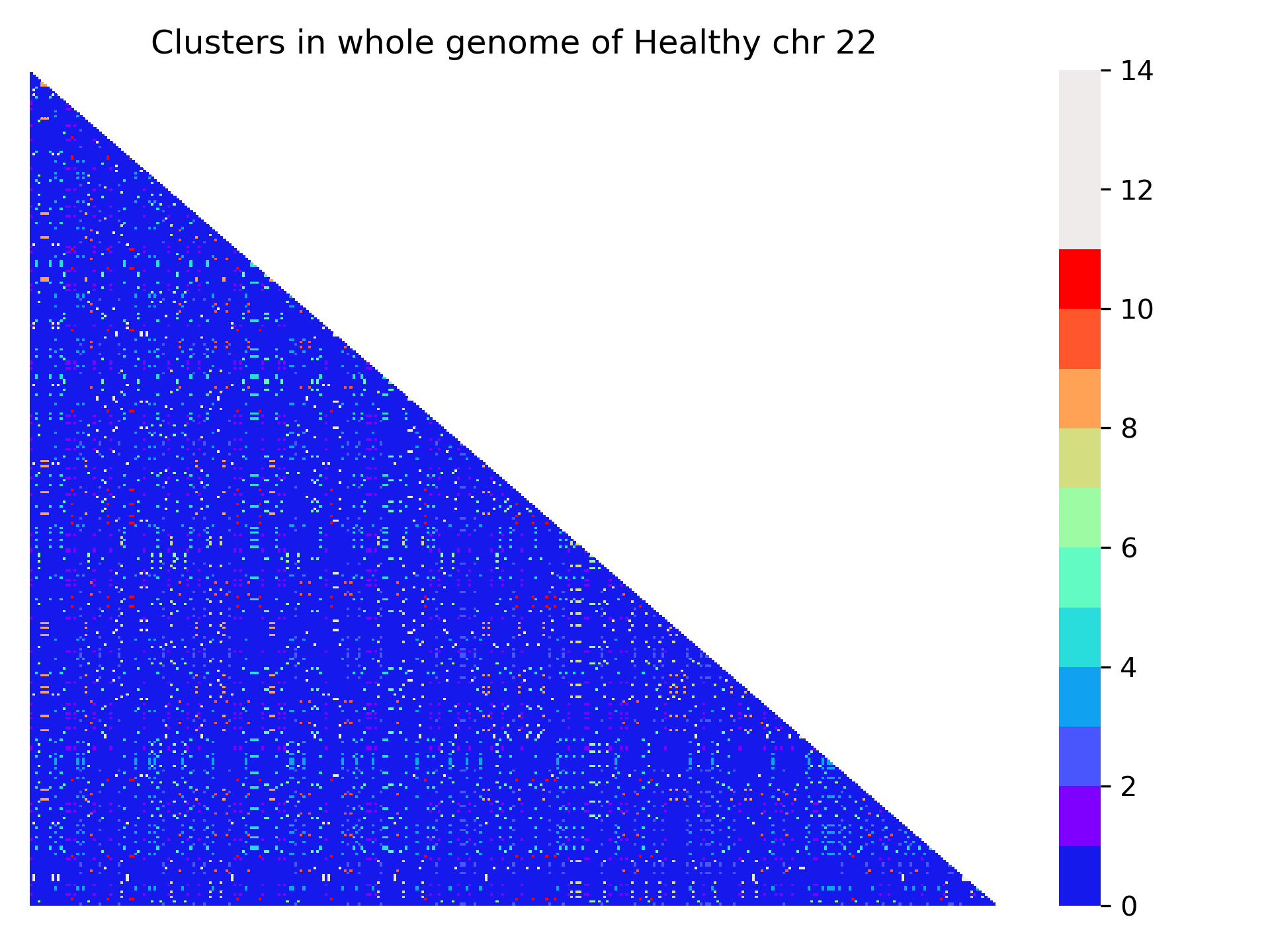

Supplement: Supplementary Material S13 — Piece-wise permutation p-values of the KS statistics, calculated for all bins obtained in Supplementary Material S8 , in every chromosomal region for each phenotype. [file DataSheet_13.zip › SuppMat10/SuppMat10/chr22/Healthy-chr22-gstart-heat.png]

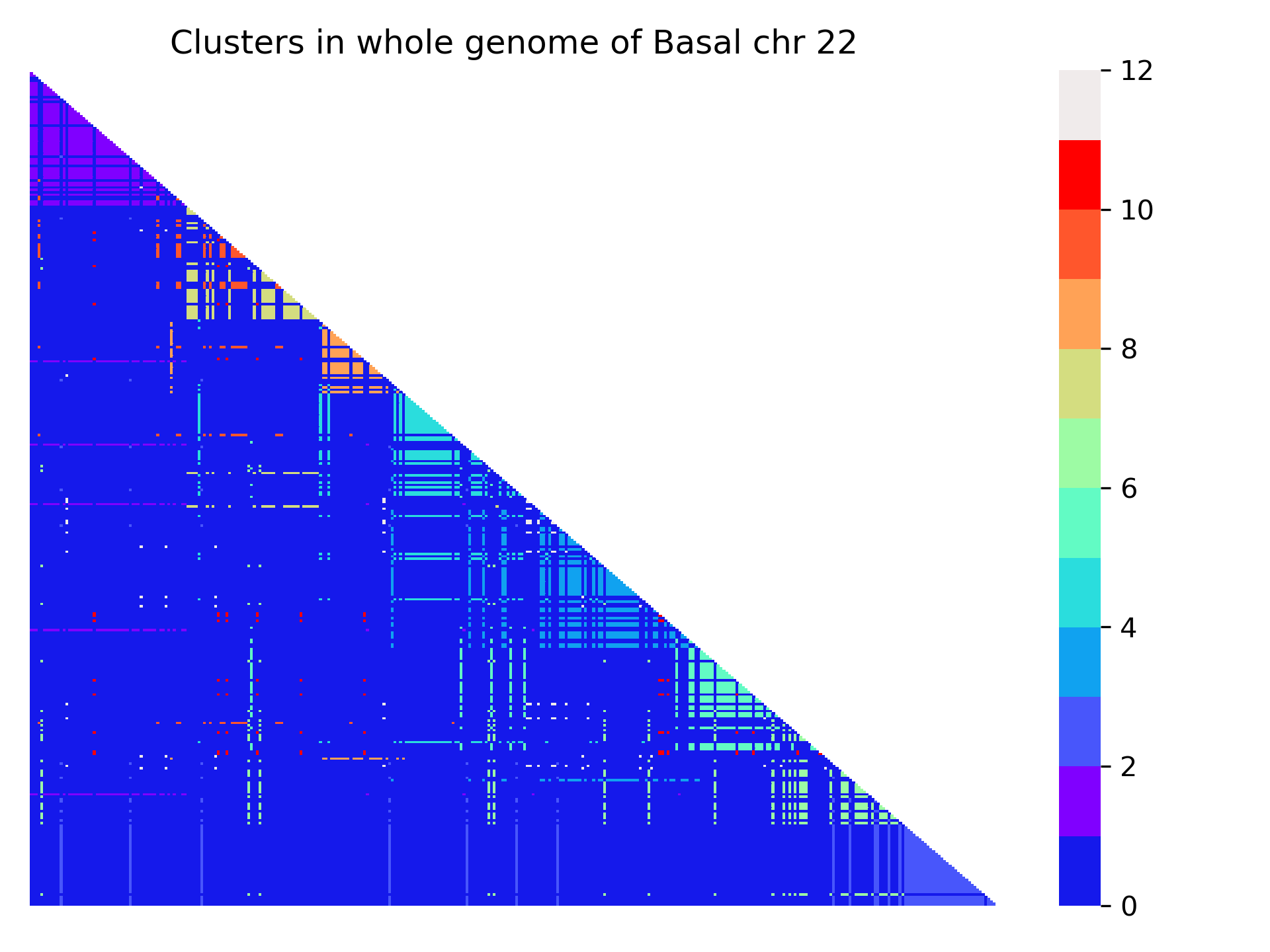

Supplement: Supplementary Material S13 — Piece-wise permutation p-values of the KS statistics, calculated for all bins obtained in Supplementary Material S8 , in every chromosomal region for each phenotype. [file DataSheet_13.zip › SuppMat10/SuppMat10/chr22/Basal-chr22-gstart-heat.png]

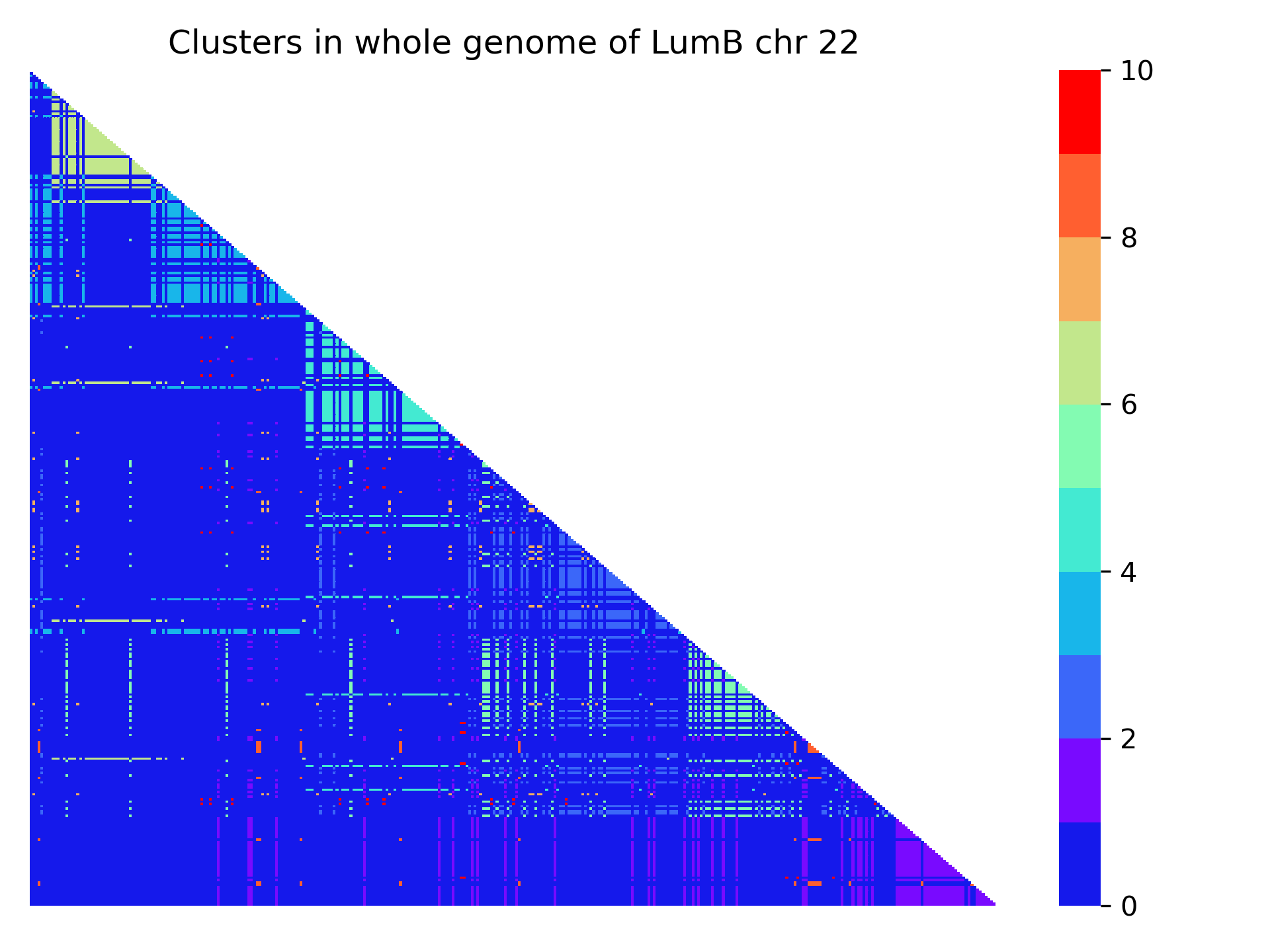

Supplement: Supplementary Material S13 — Piece-wise permutation p-values of the KS statistics, calculated for all bins obtained in Supplementary Material S8 , in every chromosomal region for each phenotype. [file DataSheet_13.zip › SuppMat10/SuppMat10/chr22/LumB-chr22-gstart-heat.png]

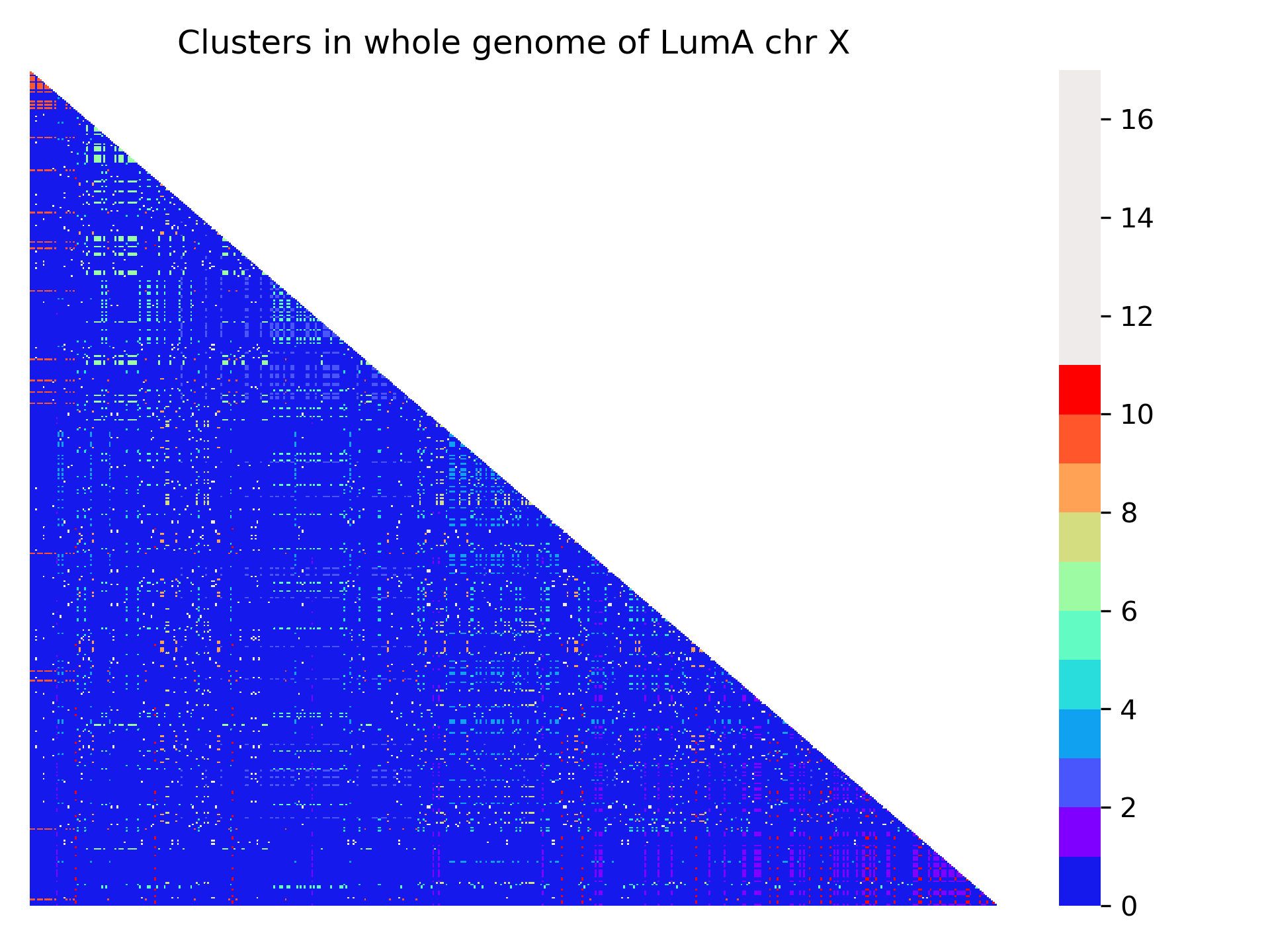

Supplement: Supplementary Material S13 — Piece-wise permutation p-values of the KS statistics, calculated for all bins obtained in Supplementary Material S8 , in every chromosomal region for each phenotype. [file DataSheet_13.zip › SuppMat10/SuppMat10/chrX/LumA-chrX-gstart-heat.png]

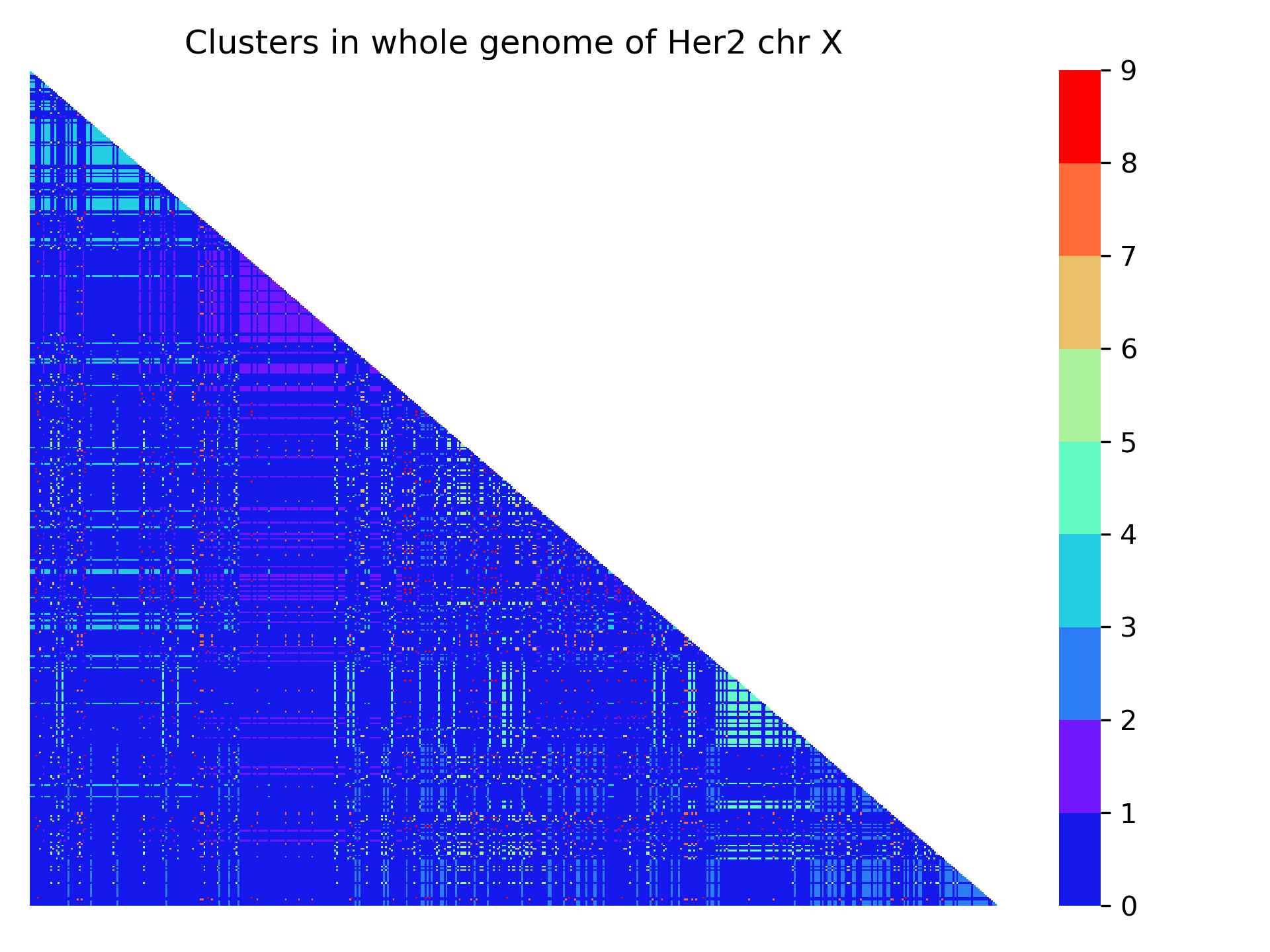

Supplement: Supplementary Material S13 — Piece-wise permutation p-values of the KS statistics, calculated for all bins obtained in Supplementary Material S8 , in every chromosomal region for each phenotype. [file DataSheet_13.zip › SuppMat10/SuppMat10/chrX/Her2-chrX-gstart-heat.png]

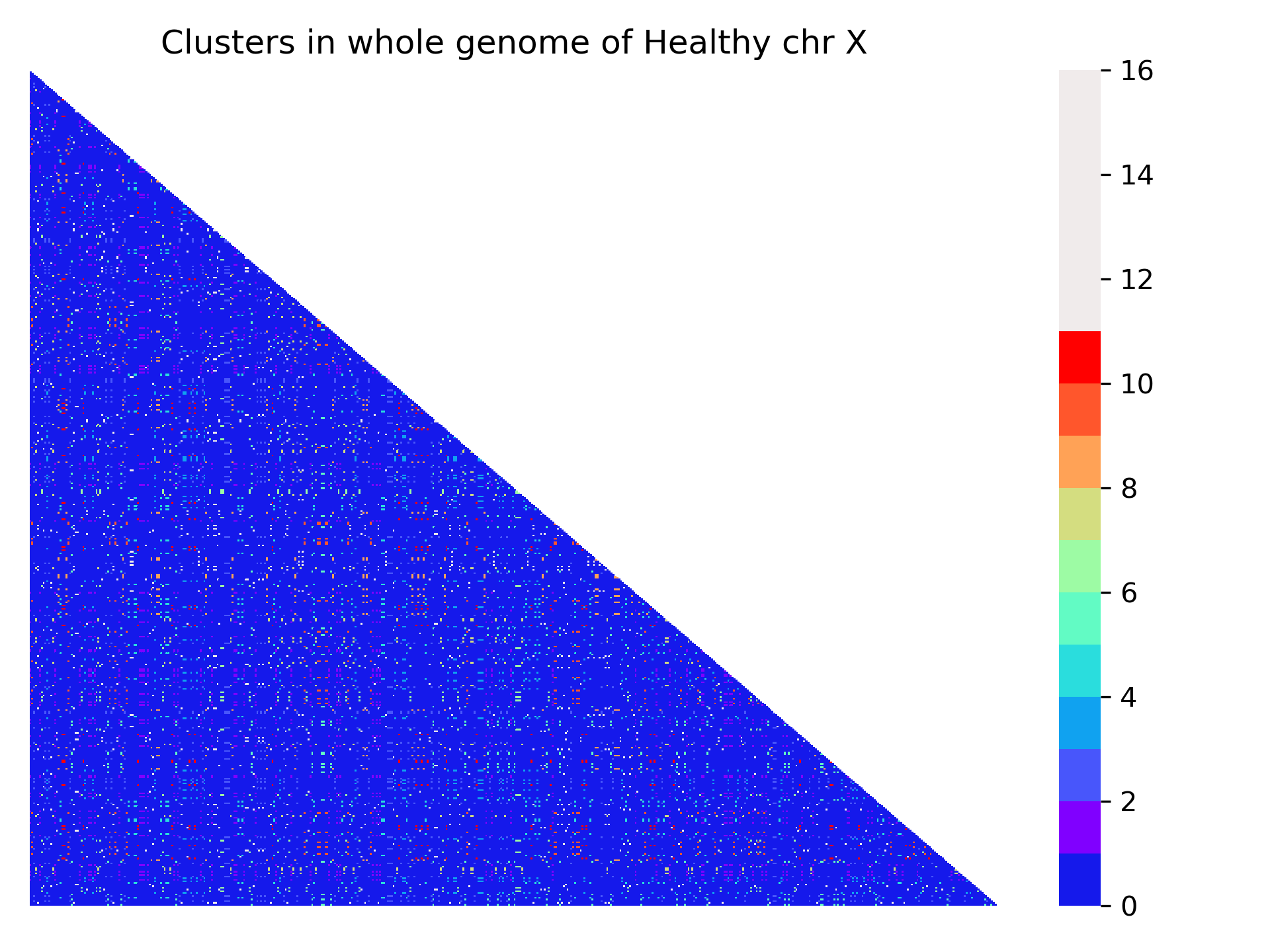

Supplement: Supplementary Material S13 — Piece-wise permutation p-values of the KS statistics, calculated for all bins obtained in Supplementary Material S8 , in every chromosomal region for each phenotype. [file DataSheet_13.zip › SuppMat10/SuppMat10/chrX/Healthy-chrX-gstart-heat.png]

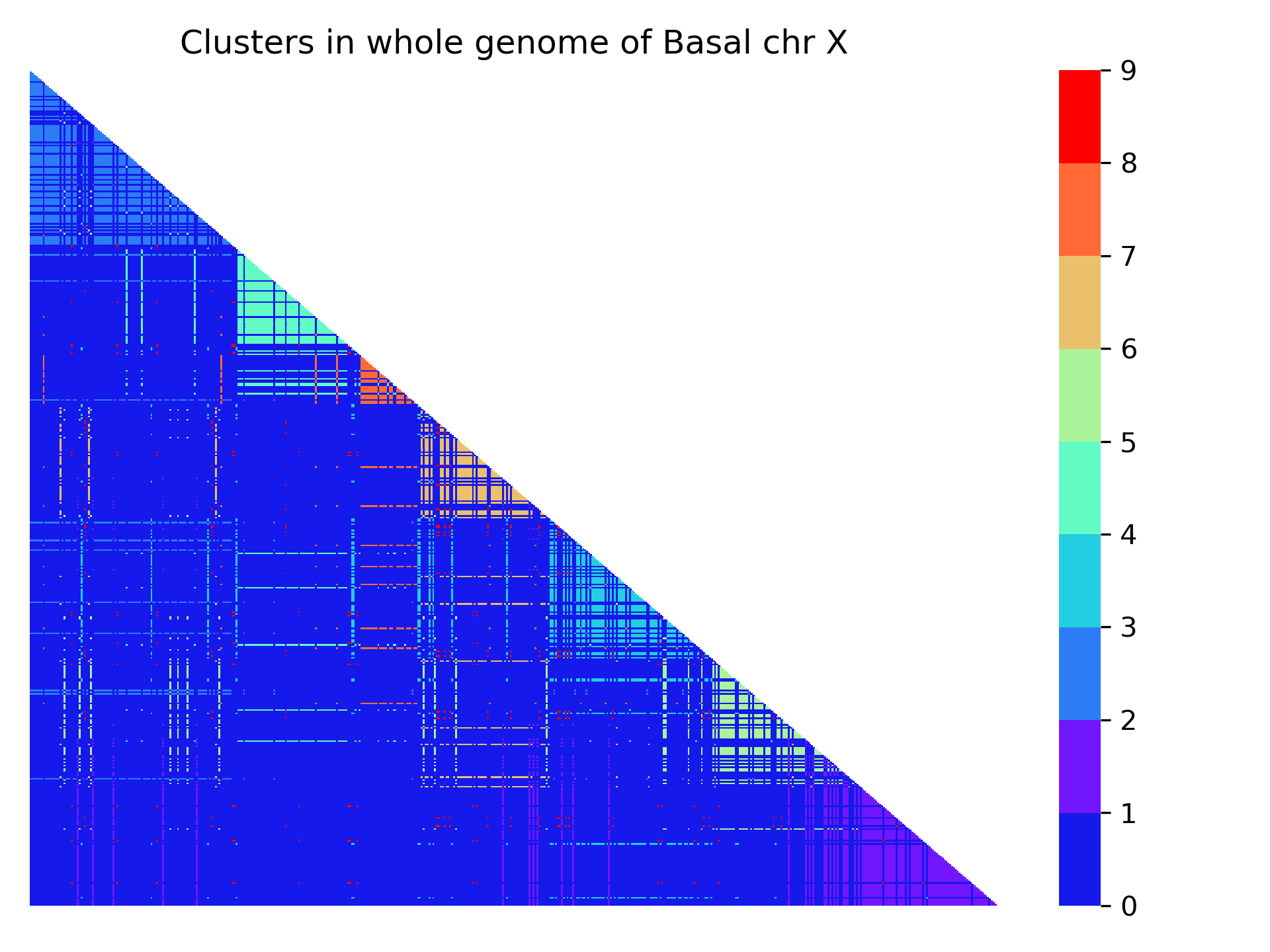

Supplement: Supplementary Material S13 — Piece-wise permutation p-values of the KS statistics, calculated for all bins obtained in Supplementary Material S8 , in every chromosomal region for each phenotype. [file DataSheet_13.zip › SuppMat10/SuppMat10/chrX/Basal-chrX-gstart-heat.png]

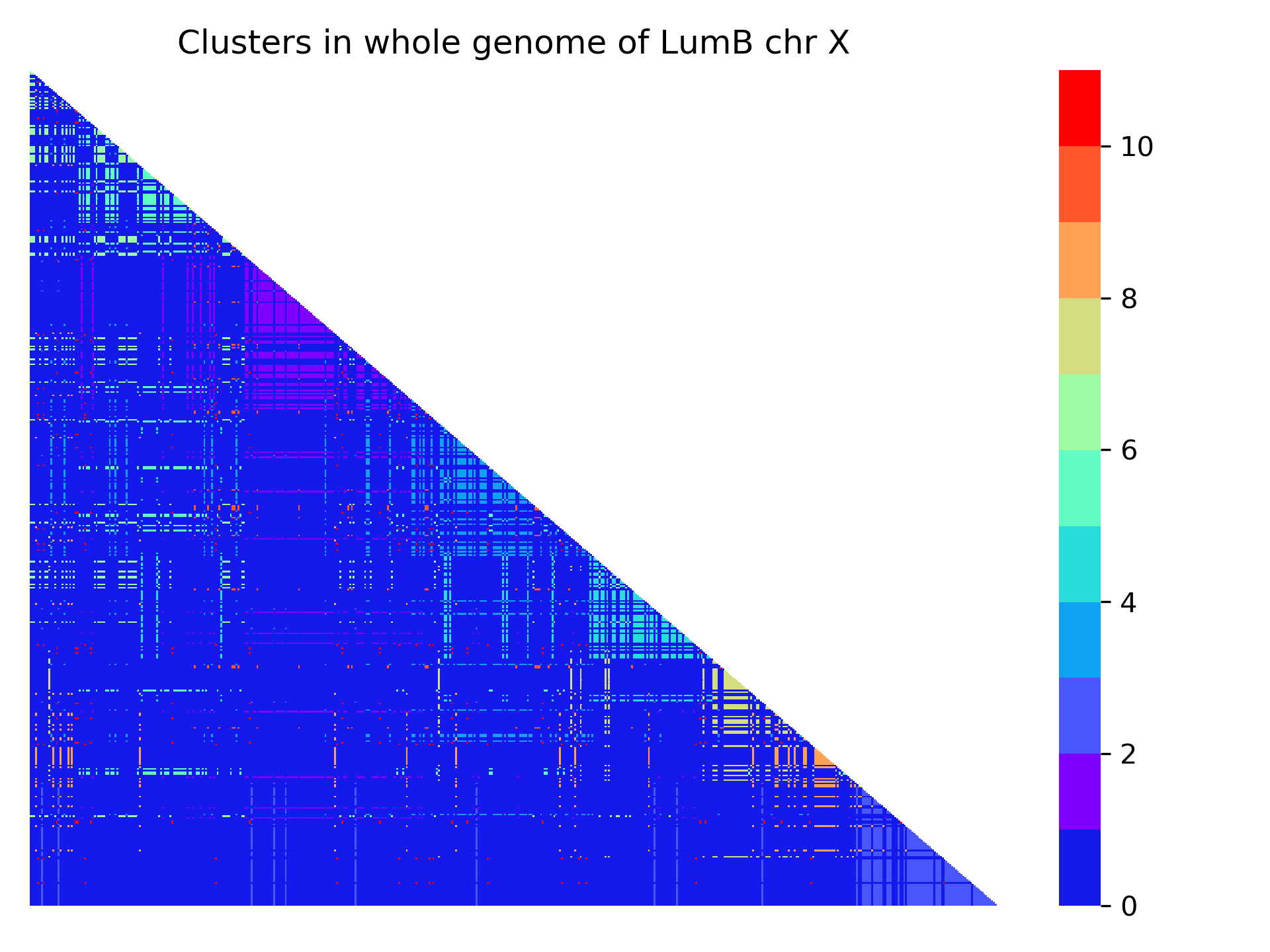

Supplement: Supplementary Material S13 — Piece-wise permutation p-values of the KS statistics, calculated for all bins obtained in Supplementary Material S8 , in every chromosomal region for each phenotype. [file DataSheet_13.zip › SuppMat10/SuppMat10/chrX/LumB-chrX-gstart-heat.png]

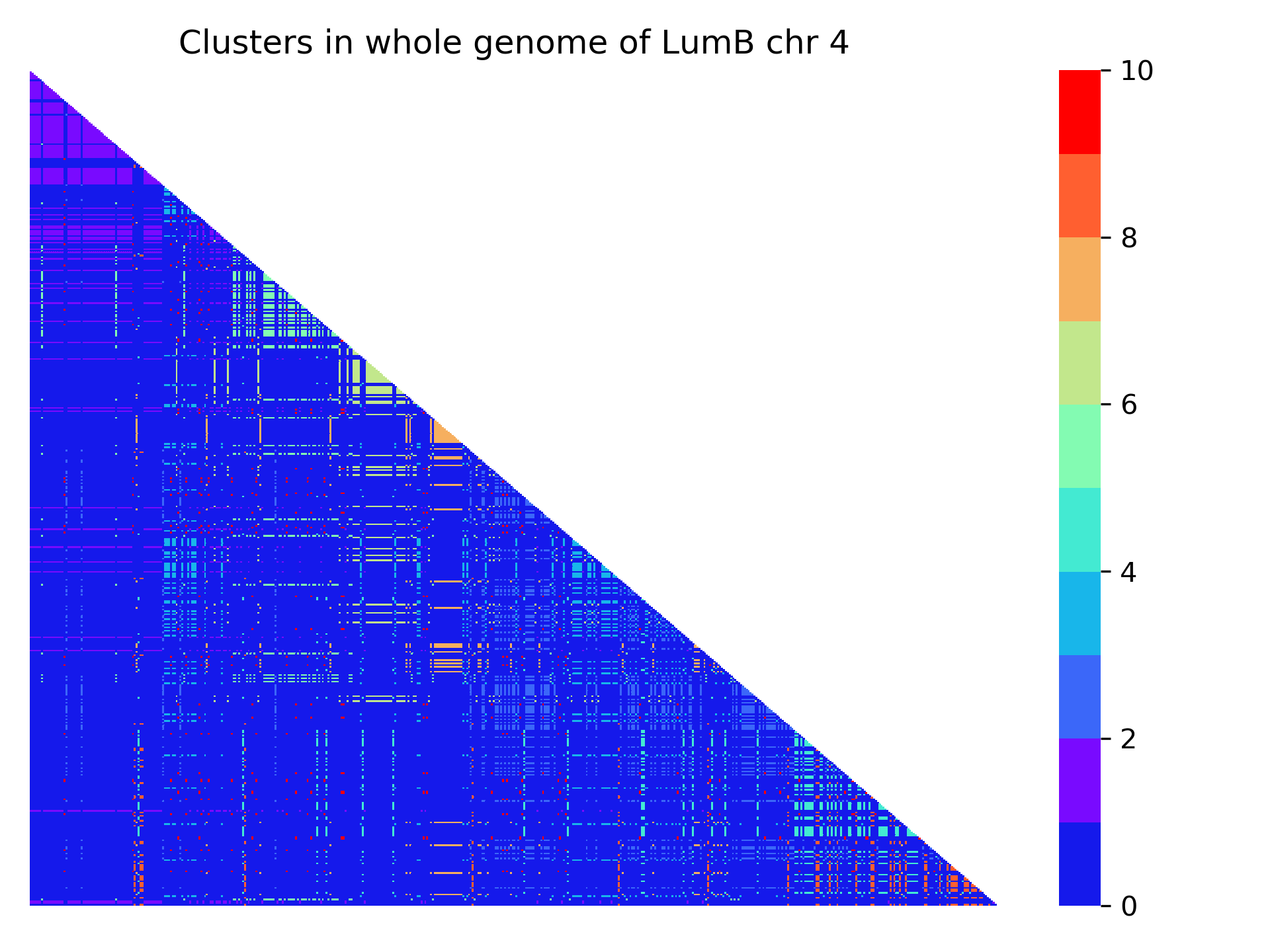

Supplement: Supplementary Material S13 — Piece-wise permutation p-values of the KS statistics, calculated for all bins obtained in Supplementary Material S8 , in every chromosomal region for each phenotype. [file DataSheet_13.zip › SuppMat10/SuppMat10/chr4/LumB-chr4-gstart-heat.png]

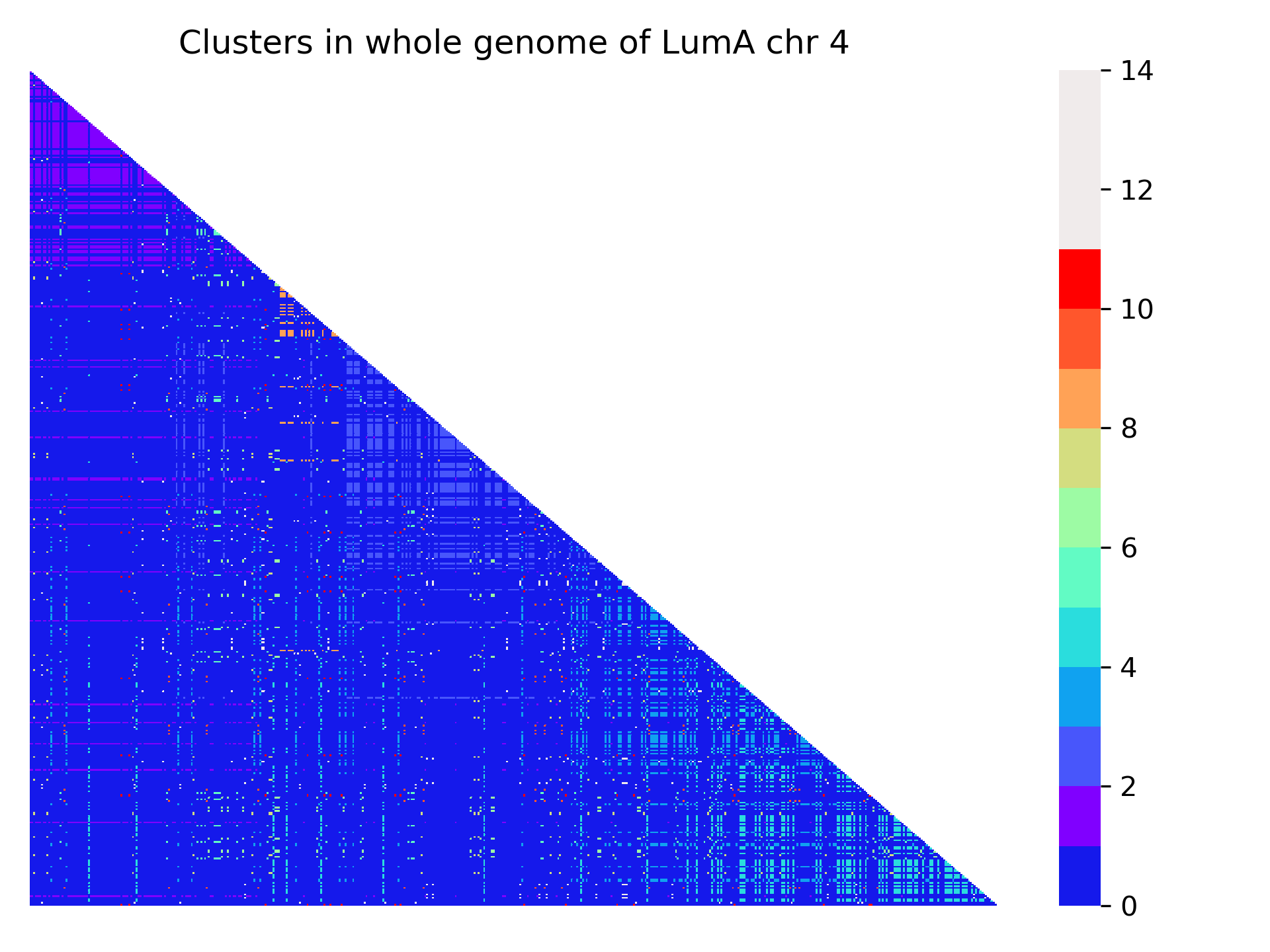

Supplement: Supplementary Material S13 — Piece-wise permutation p-values of the KS statistics, calculated for all bins obtained in Supplementary Material S8 , in every chromosomal region for each phenotype. [file DataSheet_13.zip › SuppMat10/SuppMat10/chr4/LumA-chr4-gstart-heat.png]

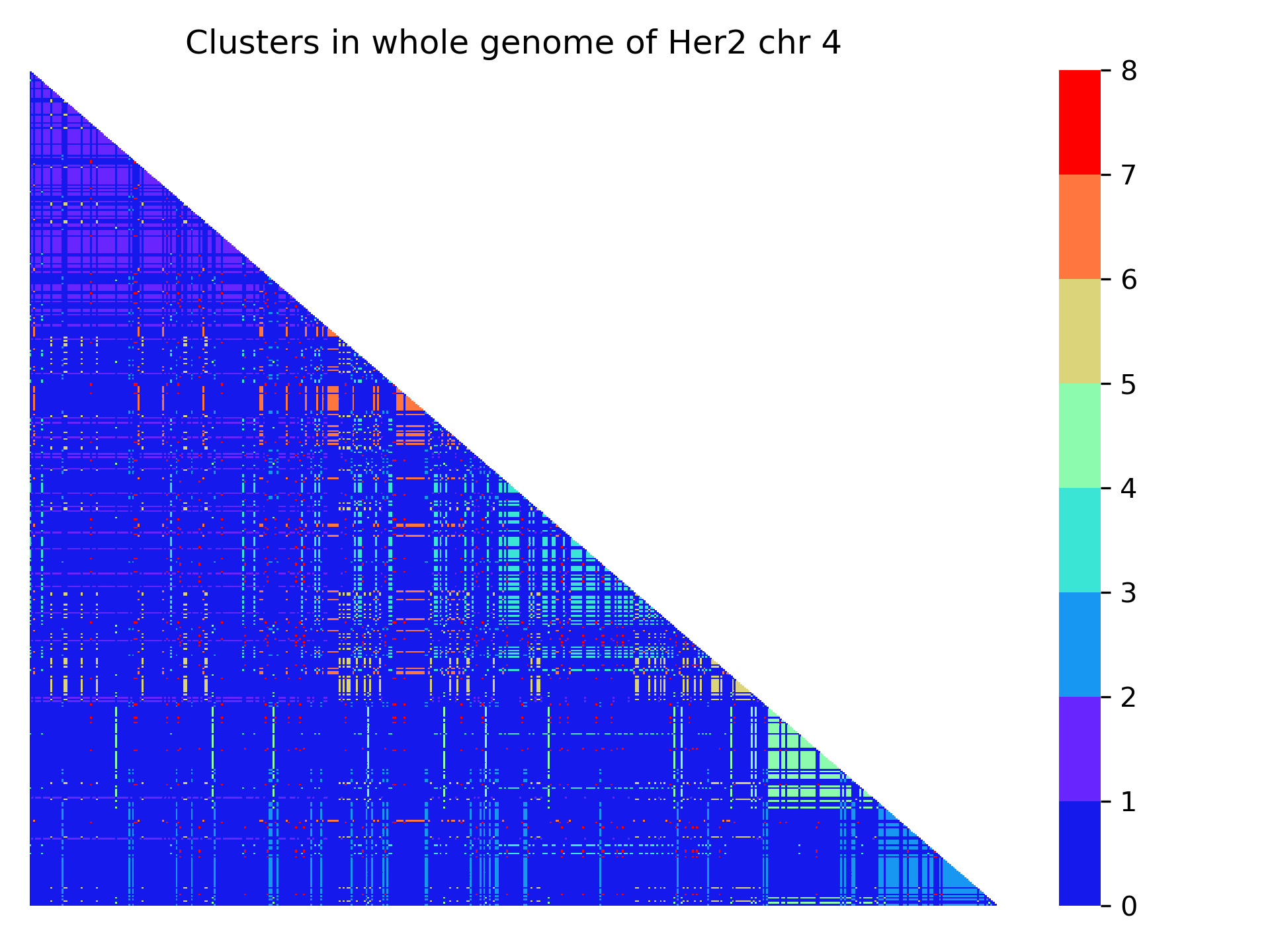

Supplement: Supplementary Material S13 — Piece-wise permutation p-values of the KS statistics, calculated for all bins obtained in Supplementary Material S8 , in every chromosomal region for each phenotype. [file DataSheet_13.zip › SuppMat10/SuppMat10/chr4/Her2-chr4-gstart-heat.png]

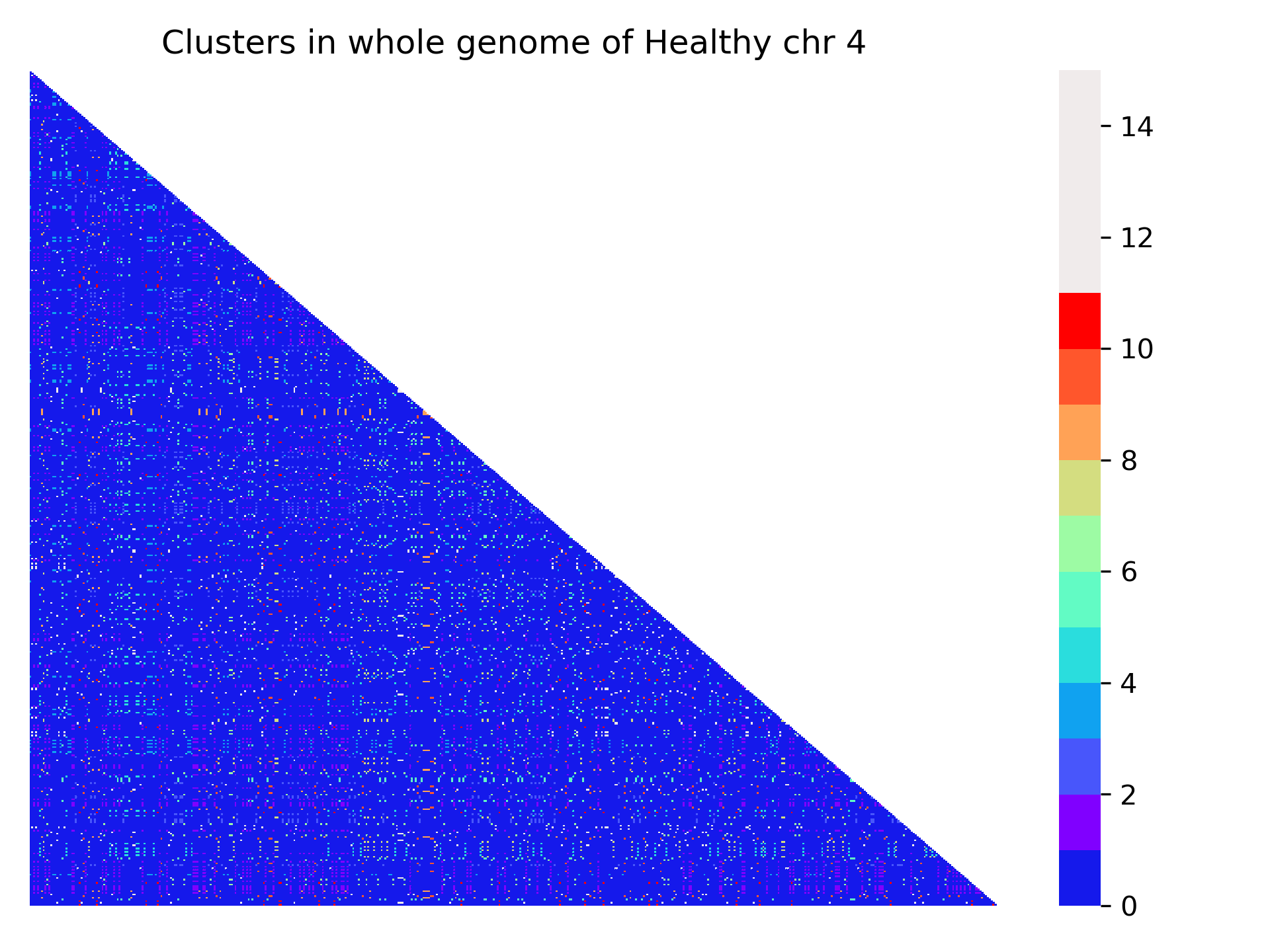

Supplement: Supplementary Material S13 — Piece-wise permutation p-values of the KS statistics, calculated for all bins obtained in Supplementary Material S8 , in every chromosomal region for each phenotype. [file DataSheet_13.zip › SuppMat10/SuppMat10/chr4/Healthy-chr4-gstart-heat.png]

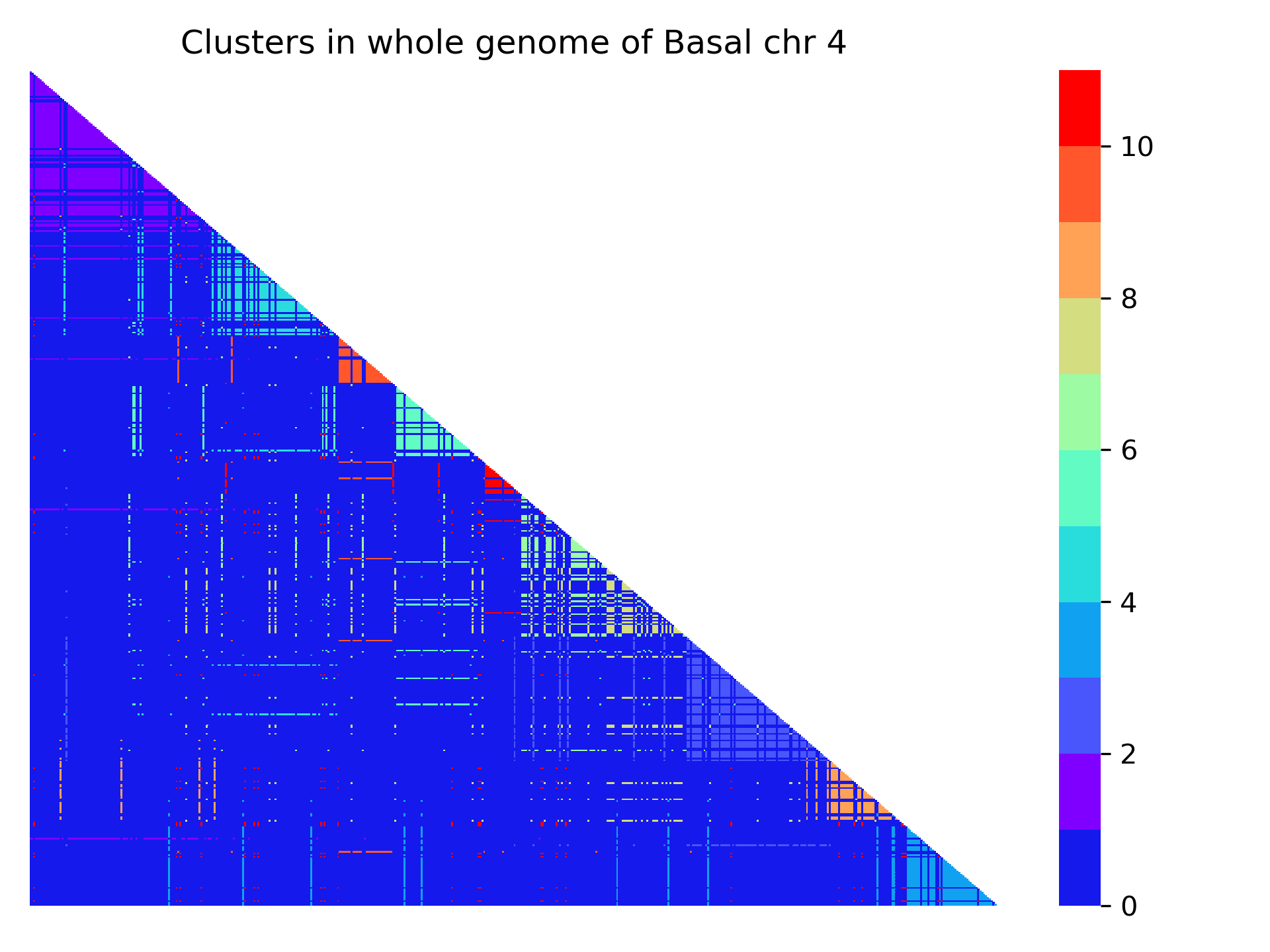

Supplement: Supplementary Material S13 — Piece-wise permutation p-values of the KS statistics, calculated for all bins obtained in Supplementary Material S8 , in every chromosomal region for each phenotype. [file DataSheet_13.zip › SuppMat10/SuppMat10/chr4/Basal-chr4-gstart-heat.png]

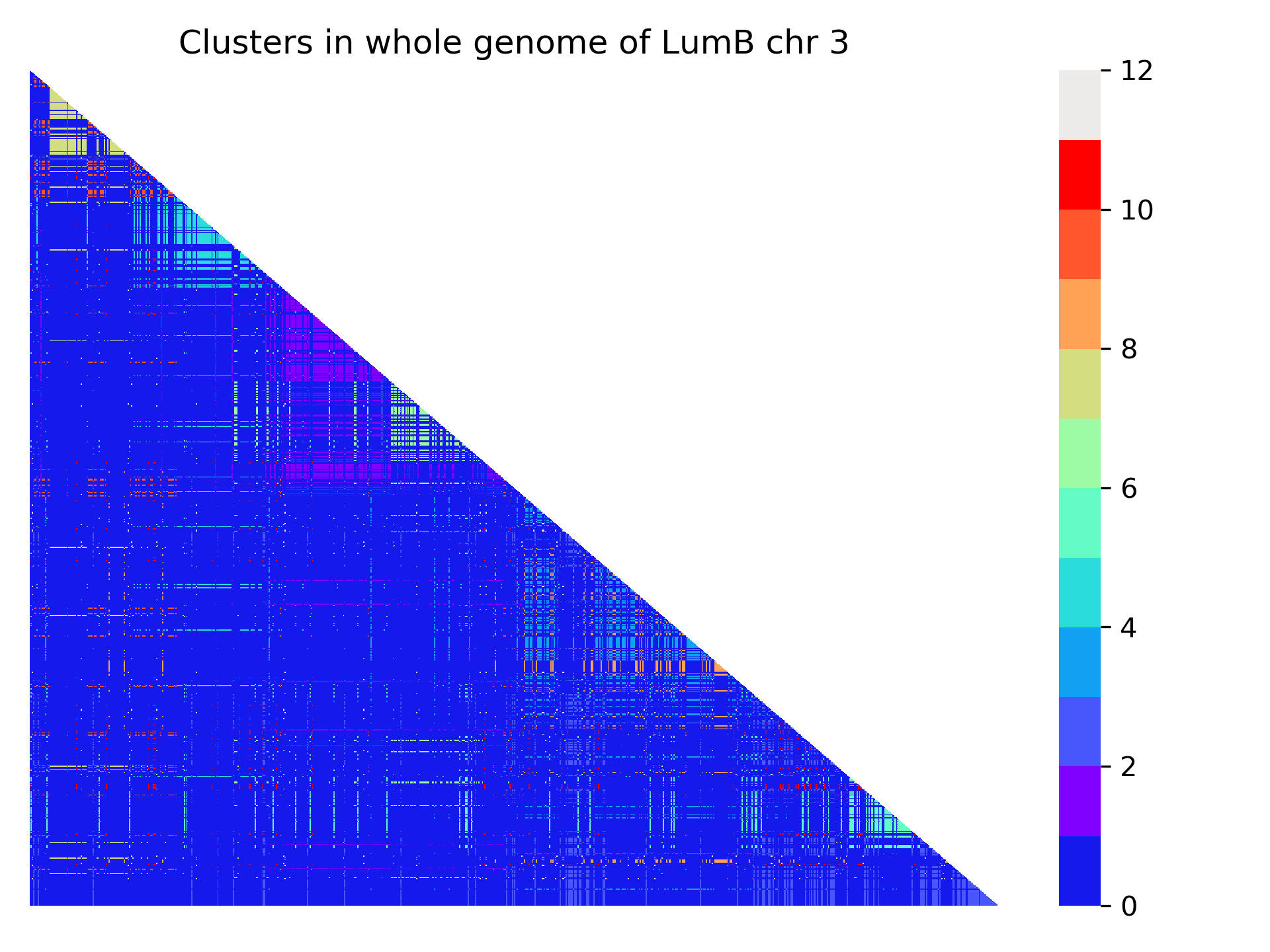

Supplement: Supplementary Material S13 — Piece-wise permutation p-values of the KS statistics, calculated for all bins obtained in Supplementary Material S8 , in every chromosomal region for each phenotype. [file DataSheet_13.zip › SuppMat10/SuppMat10/chr3/LumB-chr3-gstart-heat.png]

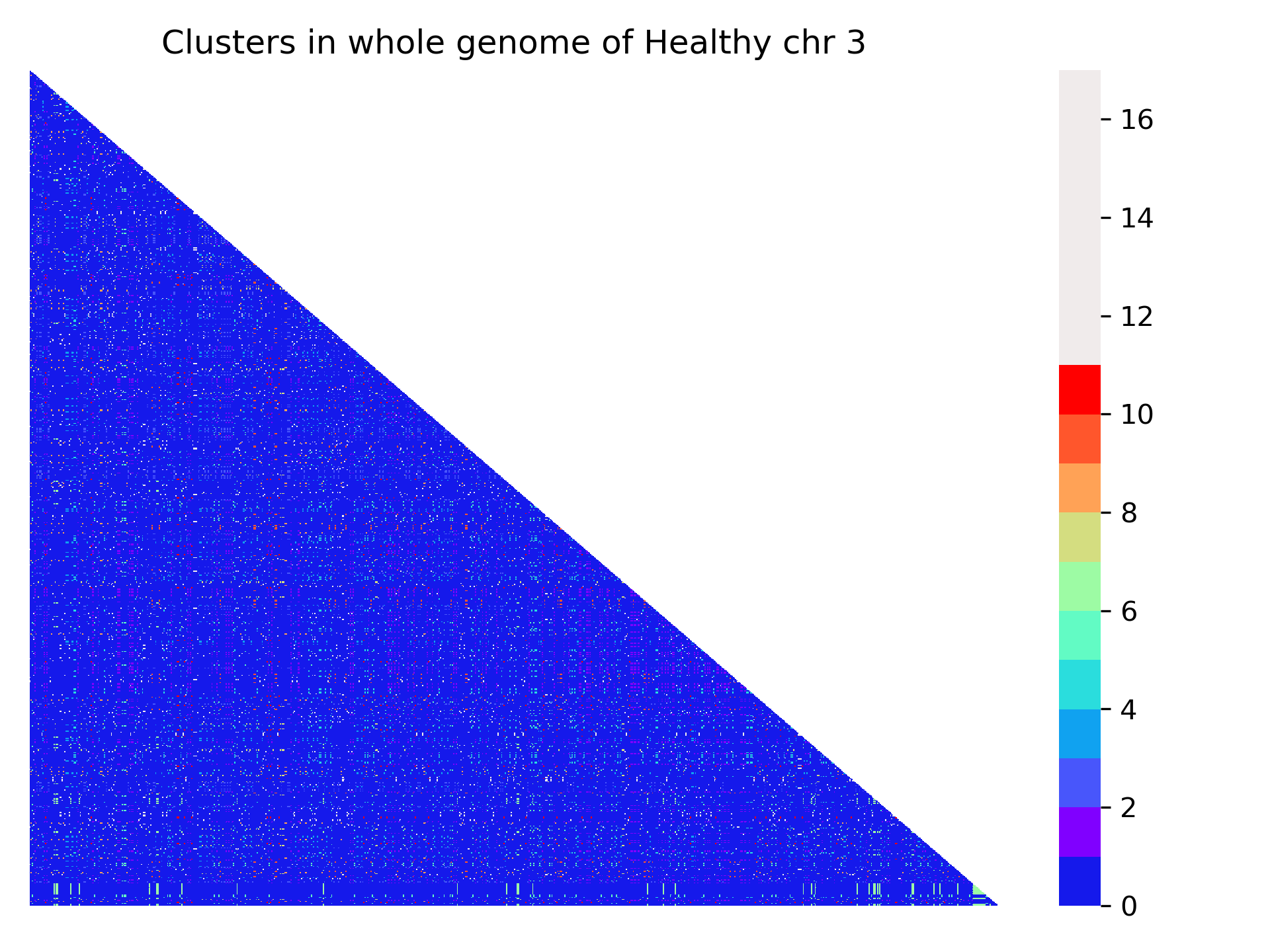

Supplement: Supplementary Material S13 — Piece-wise permutation p-values of the KS statistics, calculated for all bins obtained in Supplementary Material S8 , in every chromosomal region for each phenotype. [file DataSheet_13.zip › SuppMat10/SuppMat10/chr3/Healthy-chr3-gstart-heat.png]

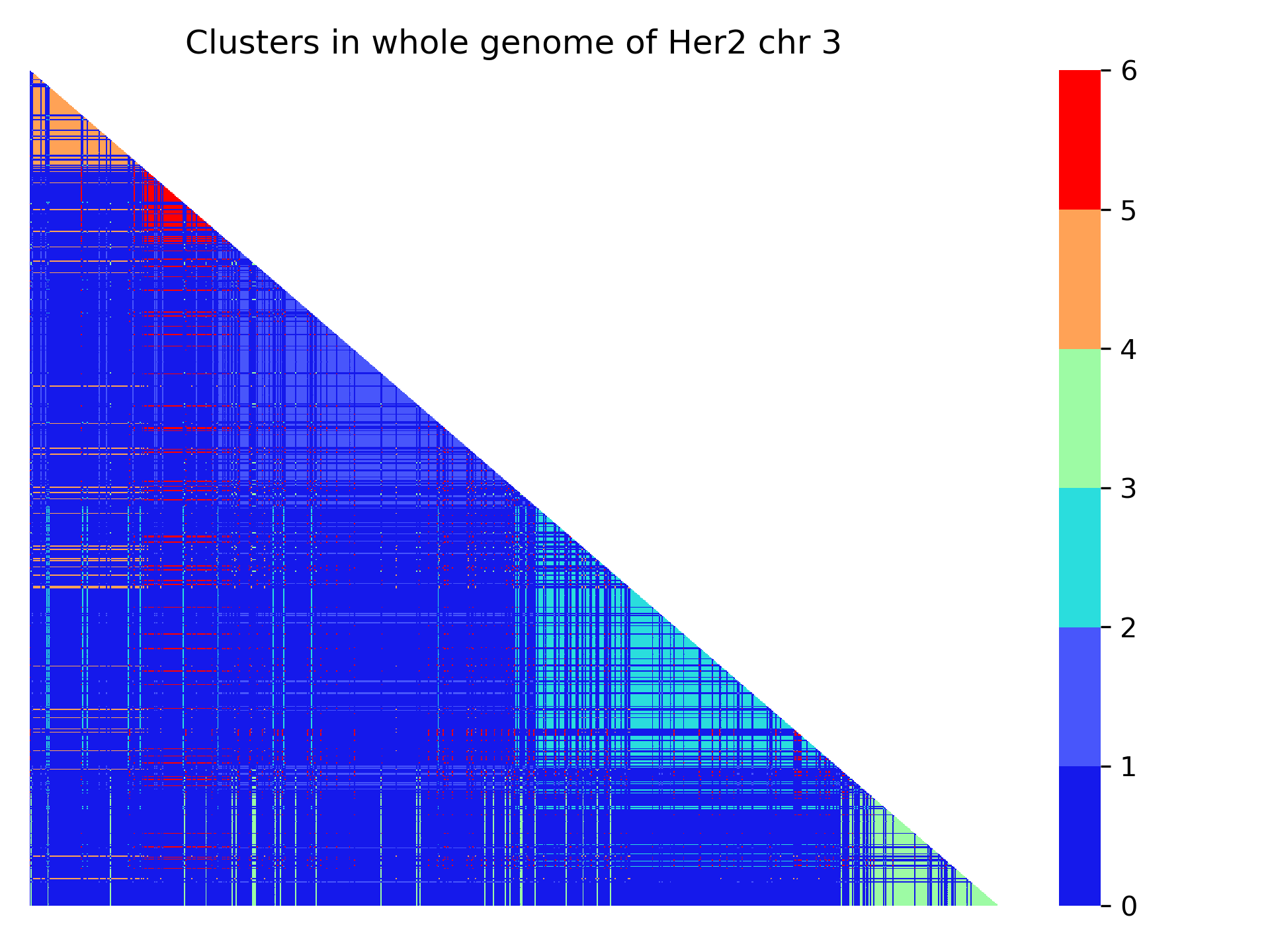

Supplement: Supplementary Material S13 — Piece-wise permutation p-values of the KS statistics, calculated for all bins obtained in Supplementary Material S8 , in every chromosomal region for each phenotype. [file DataSheet_13.zip › SuppMat10/SuppMat10/chr3/Her2-chr3-gstart-heat.png]

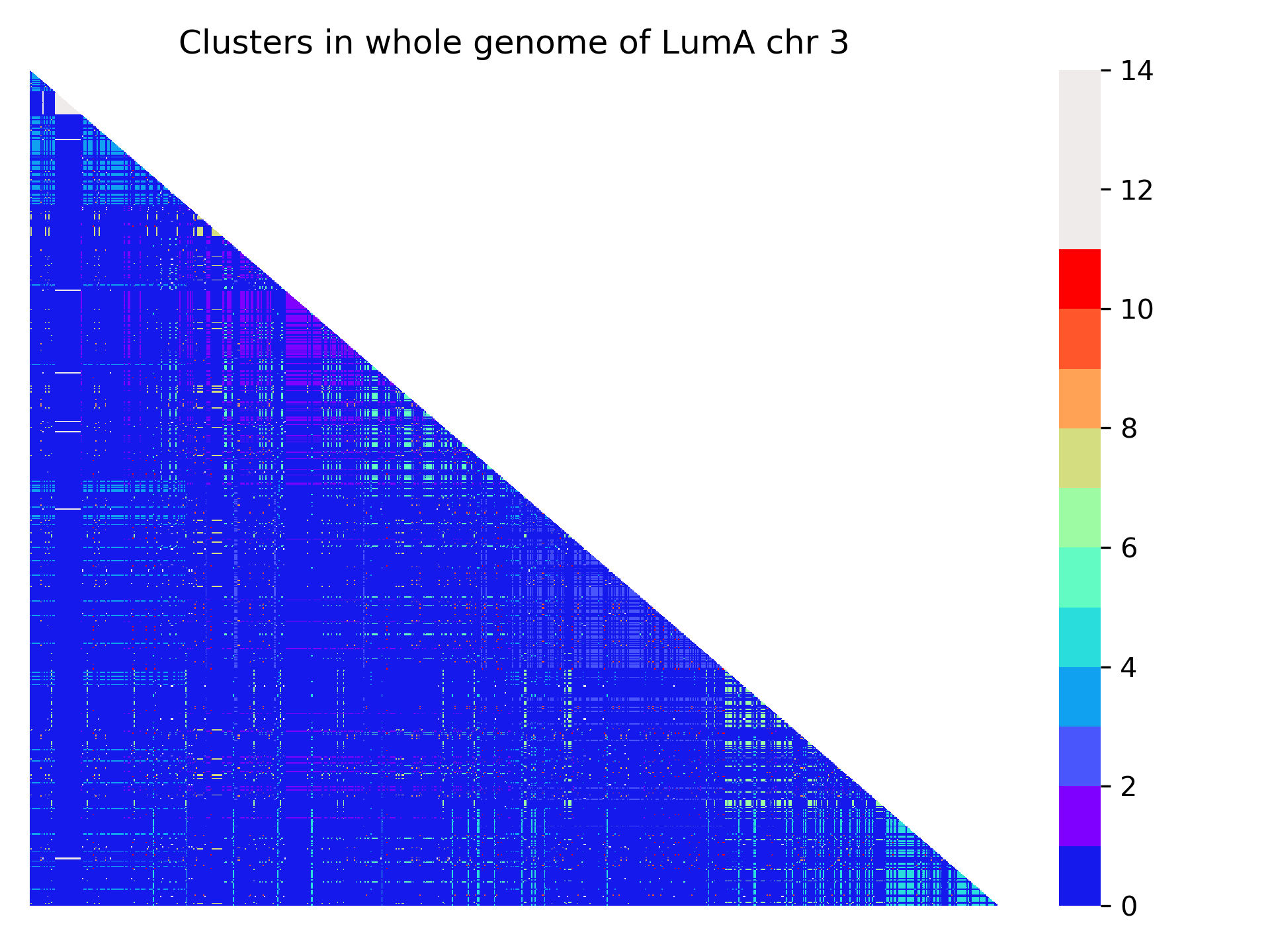

Supplement: Supplementary Material S13 — Piece-wise permutation p-values of the KS statistics, calculated for all bins obtained in Supplementary Material S8 , in every chromosomal region for each phenotype. [file DataSheet_13.zip › SuppMat10/SuppMat10/chr3/LumA-chr3-gstart-heat.png]

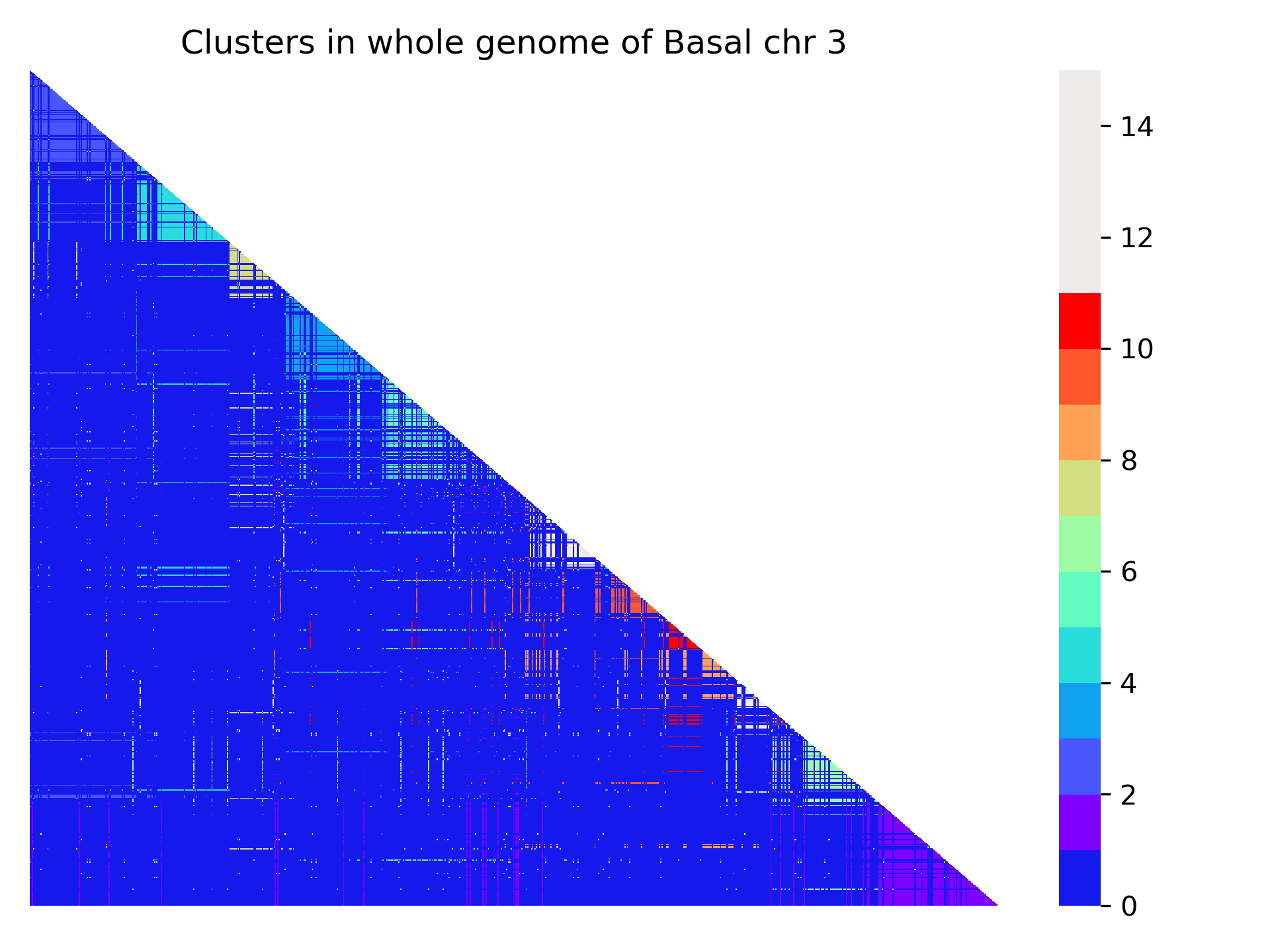

Supplement: Supplementary Material S13 — Piece-wise permutation p-values of the KS statistics, calculated for all bins obtained in Supplementary Material S8 , in every chromosomal region for each phenotype. [file DataSheet_13.zip › SuppMat10/SuppMat10/chr3/Basal-chr3-gstart-heat.png]

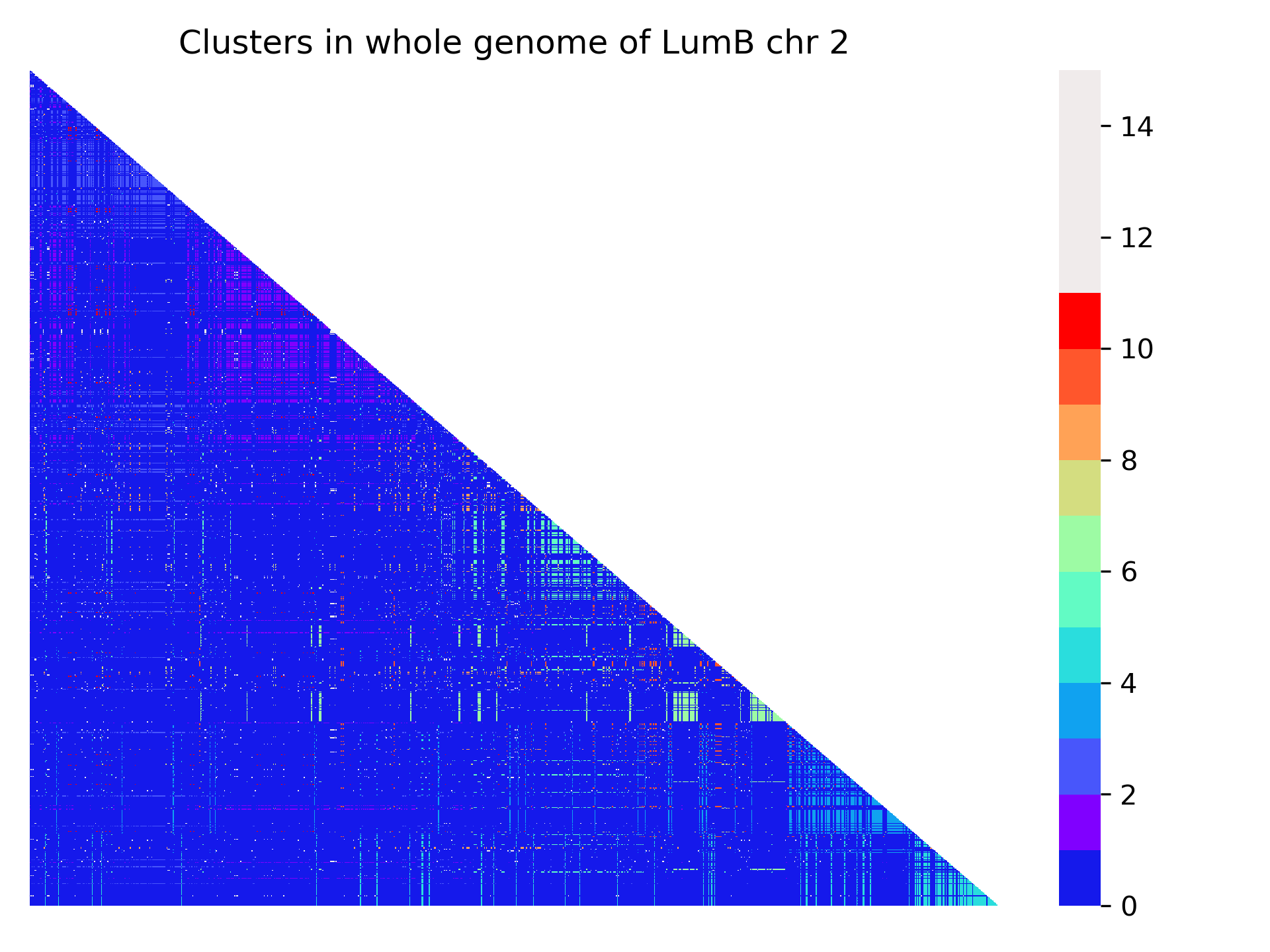

Supplement: Supplementary Material S13 — Piece-wise permutation p-values of the KS statistics, calculated for all bins obtained in Supplementary Material S8 , in every chromosomal region for each phenotype. [file DataSheet_13.zip › SuppMat10/SuppMat10/chr2/LumB-chr2-gstart-heat.png]
