# Supplementary figures and images for: Gene Co-Expression in Breast Cancer: A Matter of Distance (part 3 of 5)
Source: Front Oncol. 2021 Nov 17;11:726493. doi: 10.3389/fonc.2021.726493 (PMC8636045; doi:10.3389/fonc.2021.726493)

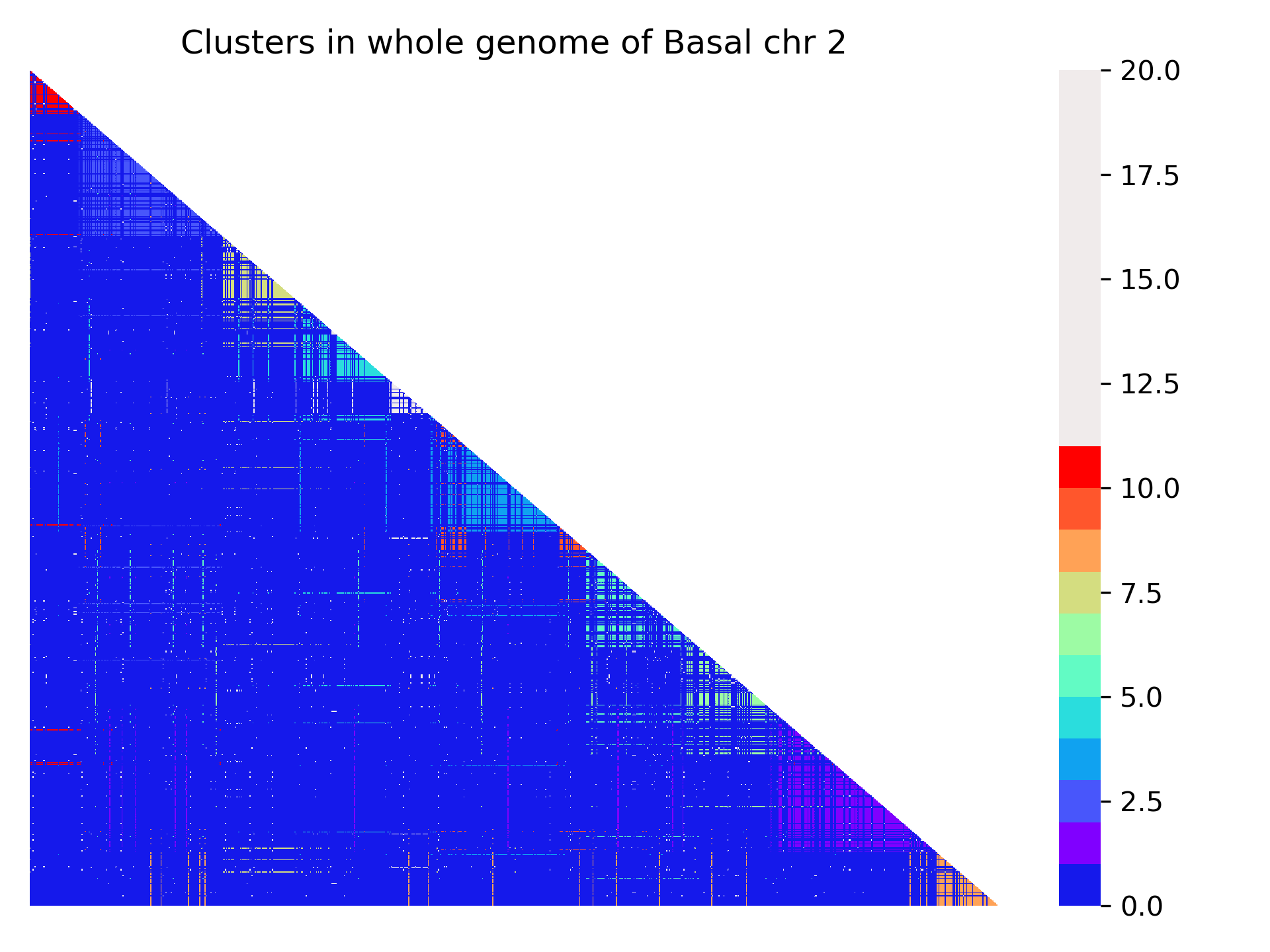

Supplement: Supplementary Material S13 — Piece-wise permutation p-values of the KS statistics, calculated for all bins obtained in Supplementary Material S8 , in every chromosomal region for each phenotype. [file DataSheet_13.zip › SuppMat10/SuppMat10/chr2/Basal-chr2-gstart-heat.png]

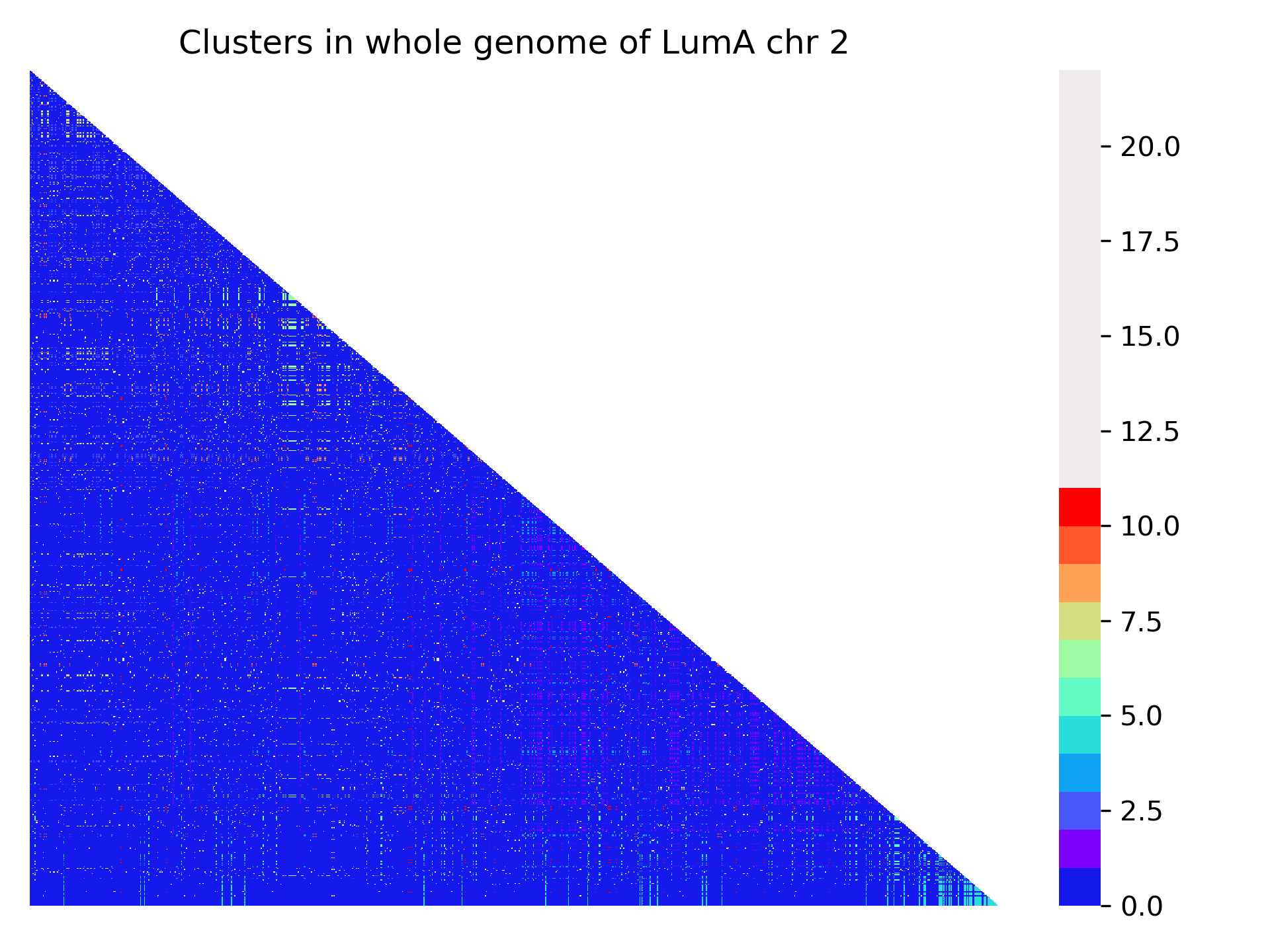

Supplement: Supplementary Material S13 — Piece-wise permutation p-values of the KS statistics, calculated for all bins obtained in Supplementary Material S8 , in every chromosomal region for each phenotype. [file DataSheet_13.zip › SuppMat10/SuppMat10/chr2/LumA-chr2-gstart-heat.png]

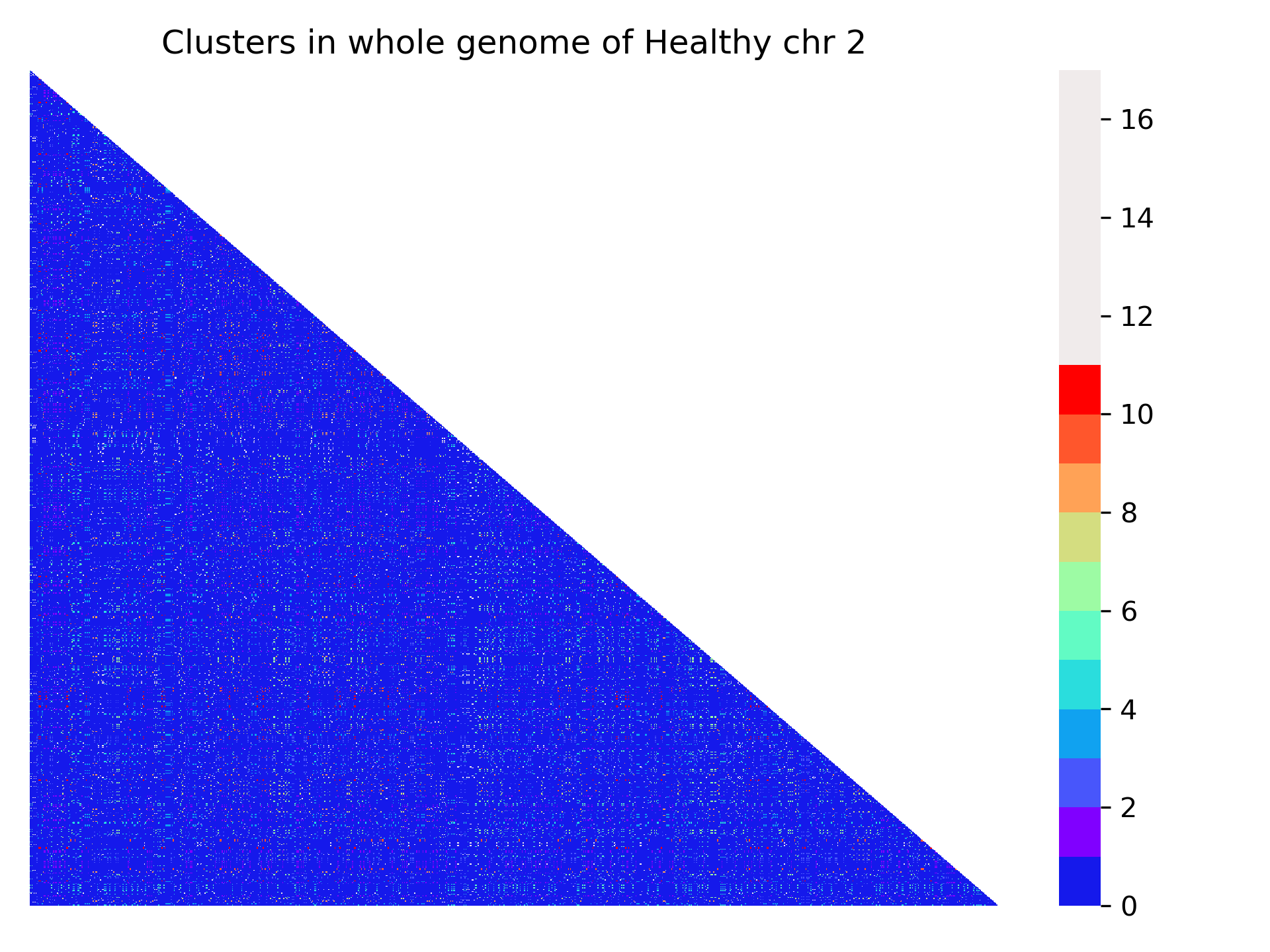

Supplement: Supplementary Material S13 — Piece-wise permutation p-values of the KS statistics, calculated for all bins obtained in Supplementary Material S8 , in every chromosomal region for each phenotype. [file DataSheet_13.zip › SuppMat10/SuppMat10/chr2/Healthy-chr2-gstart-heat.png]

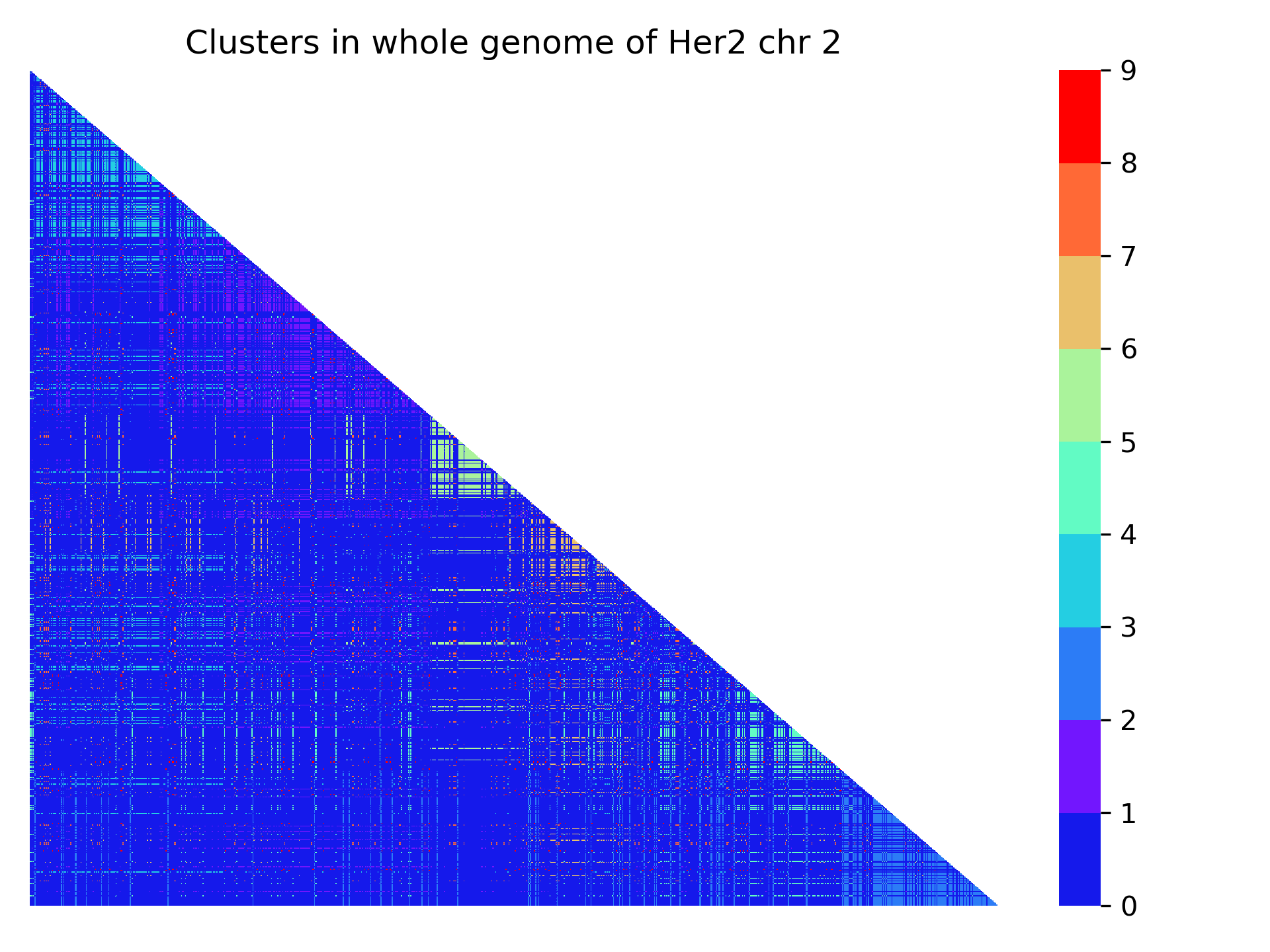

Supplement: Supplementary Material S13 — Piece-wise permutation p-values of the KS statistics, calculated for all bins obtained in Supplementary Material S8 , in every chromosomal region for each phenotype. [file DataSheet_13.zip › SuppMat10/SuppMat10/chr2/Her2-chr2-gstart-heat.png]

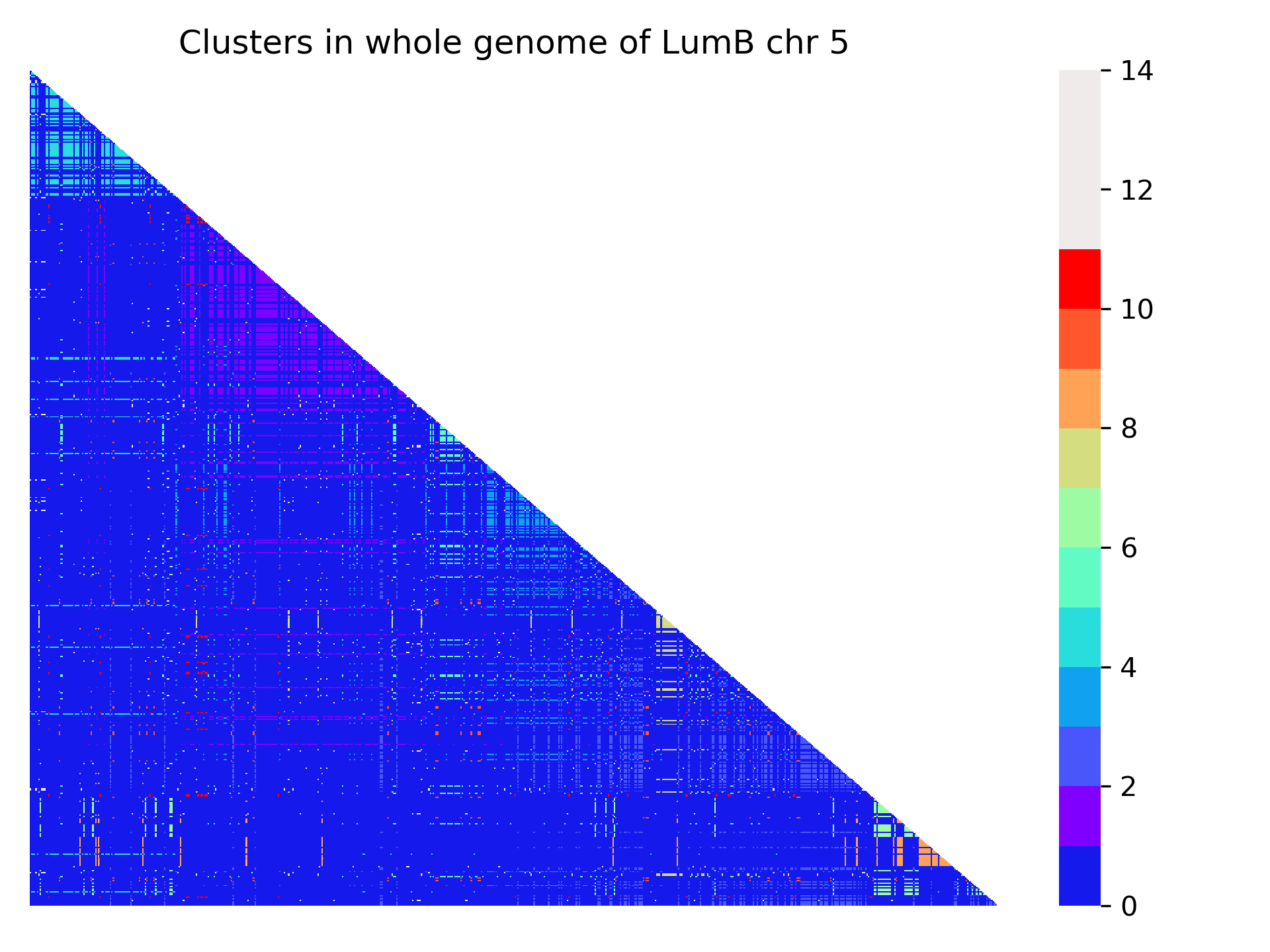

Supplement: Supplementary Material S13 — Piece-wise permutation p-values of the KS statistics, calculated for all bins obtained in Supplementary Material S8 , in every chromosomal region for each phenotype. [file DataSheet_13.zip › SuppMat10/SuppMat10/chr5/LumB-chr5-gstart-heat.png]

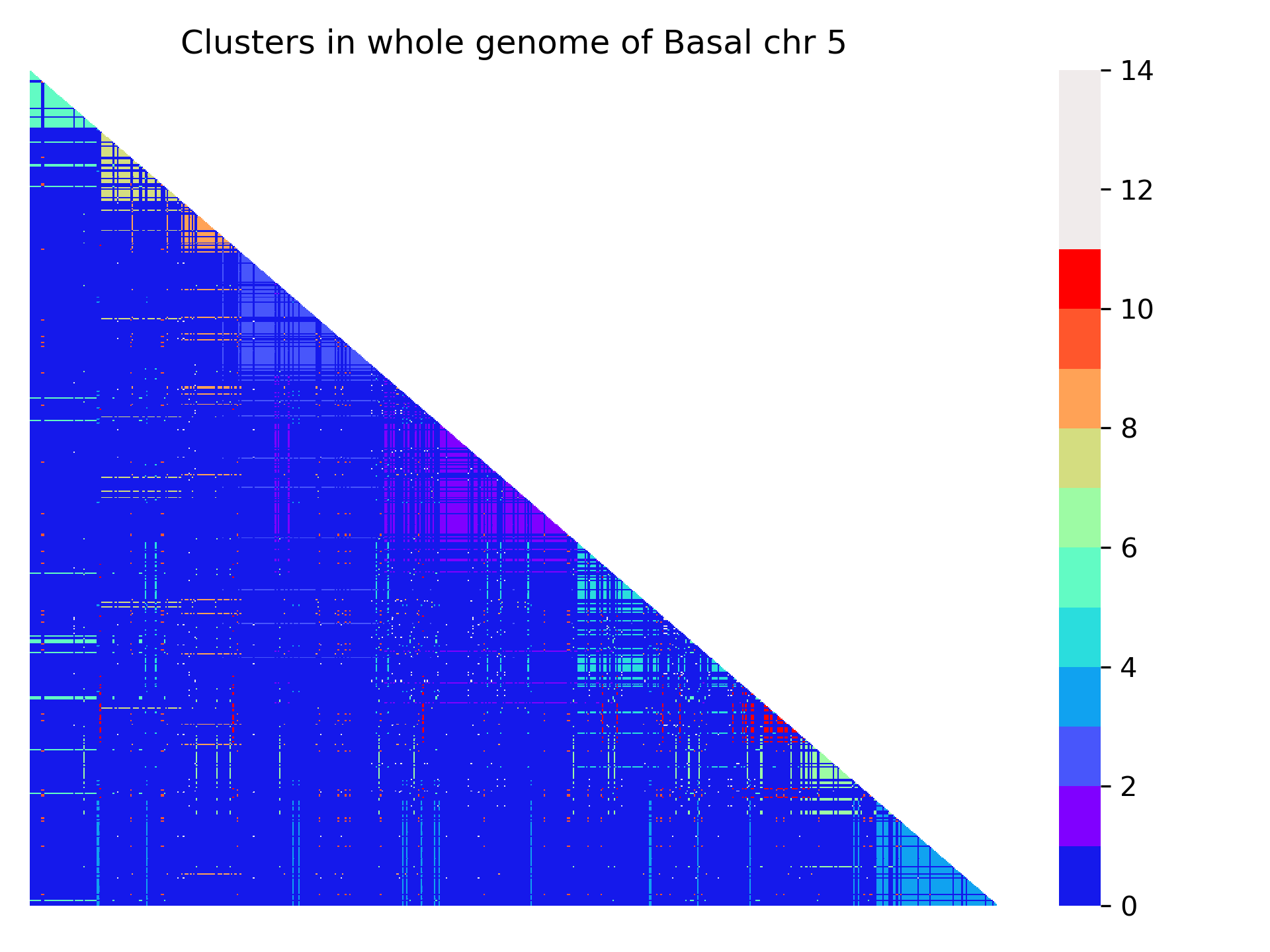

Supplement: Supplementary Material S13 — Piece-wise permutation p-values of the KS statistics, calculated for all bins obtained in Supplementary Material S8 , in every chromosomal region for each phenotype. [file DataSheet_13.zip › SuppMat10/SuppMat10/chr5/Basal-chr5-gstart-heat.png]

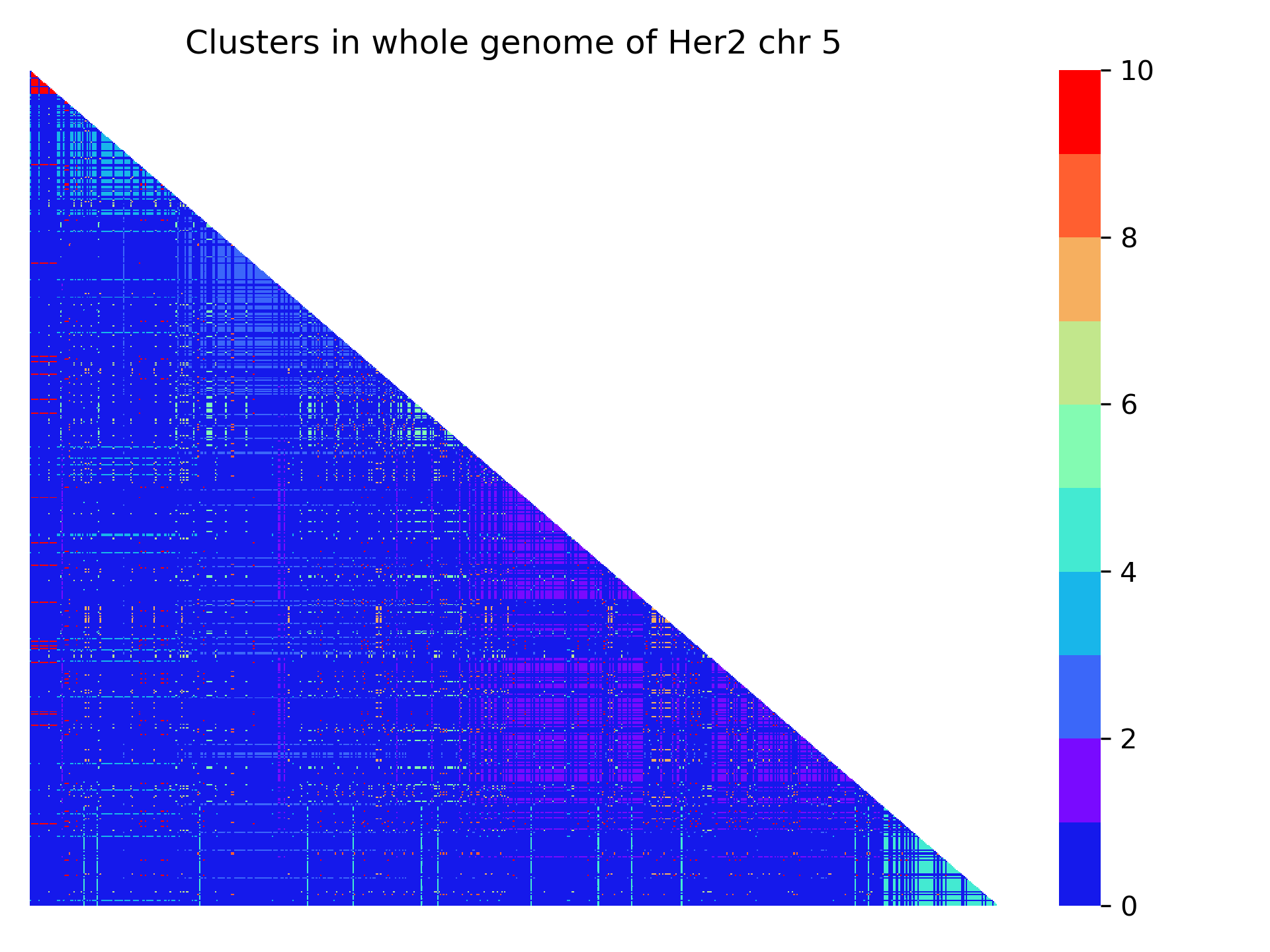

Supplement: Supplementary Material S13 — Piece-wise permutation p-values of the KS statistics, calculated for all bins obtained in Supplementary Material S8 , in every chromosomal region for each phenotype. [file DataSheet_13.zip › SuppMat10/SuppMat10/chr5/Her2-chr5-gstart-heat.png]

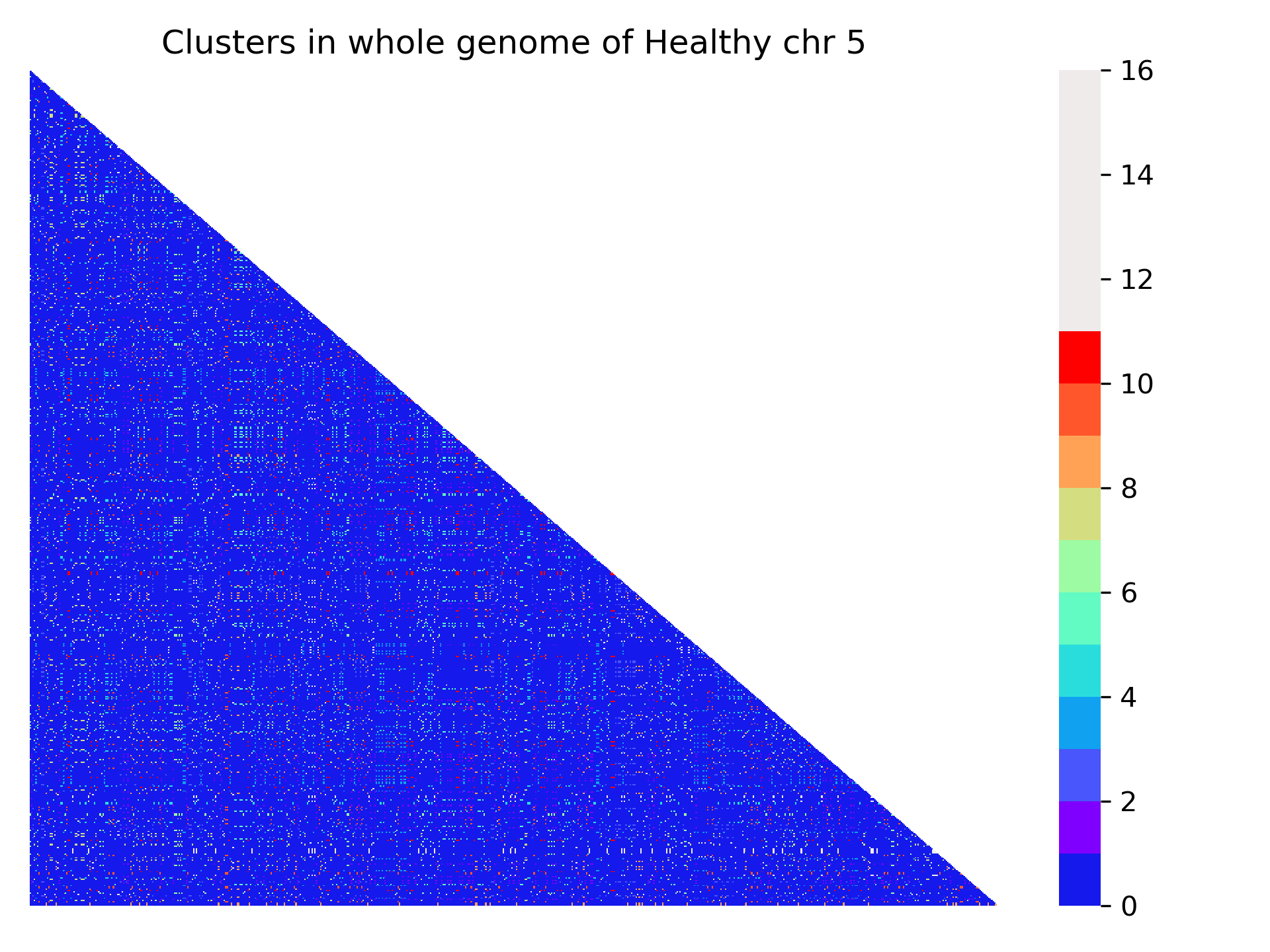

Supplement: Supplementary Material S13 — Piece-wise permutation p-values of the KS statistics, calculated for all bins obtained in Supplementary Material S8 , in every chromosomal region for each phenotype. [file DataSheet_13.zip › SuppMat10/SuppMat10/chr5/Healthy-chr5-gstart-heat.png]

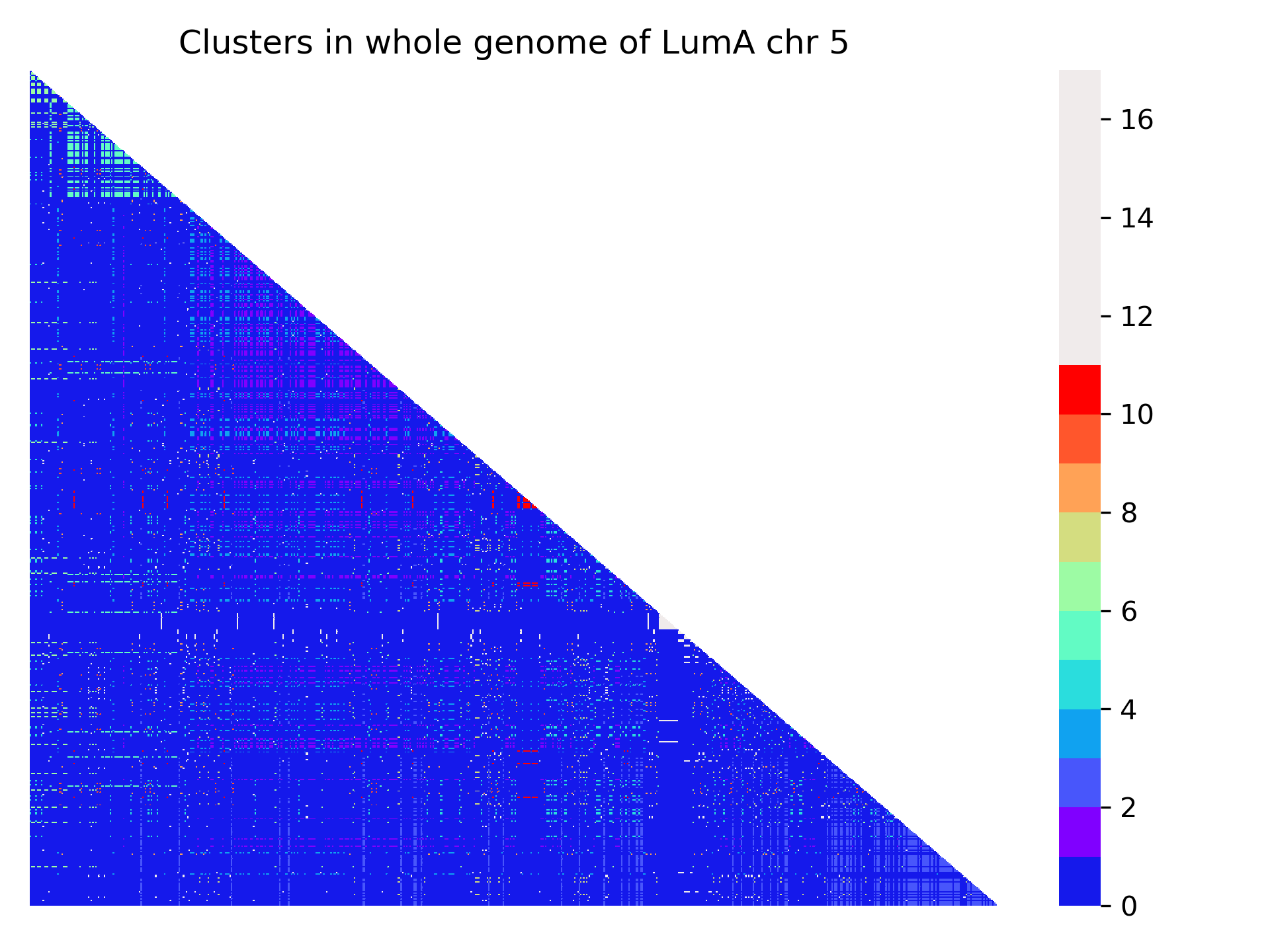

Supplement: Supplementary Material S13 — Piece-wise permutation p-values of the KS statistics, calculated for all bins obtained in Supplementary Material S8 , in every chromosomal region for each phenotype. [file DataSheet_13.zip › SuppMat10/SuppMat10/chr5/LumA-chr5-gstart-heat.png]

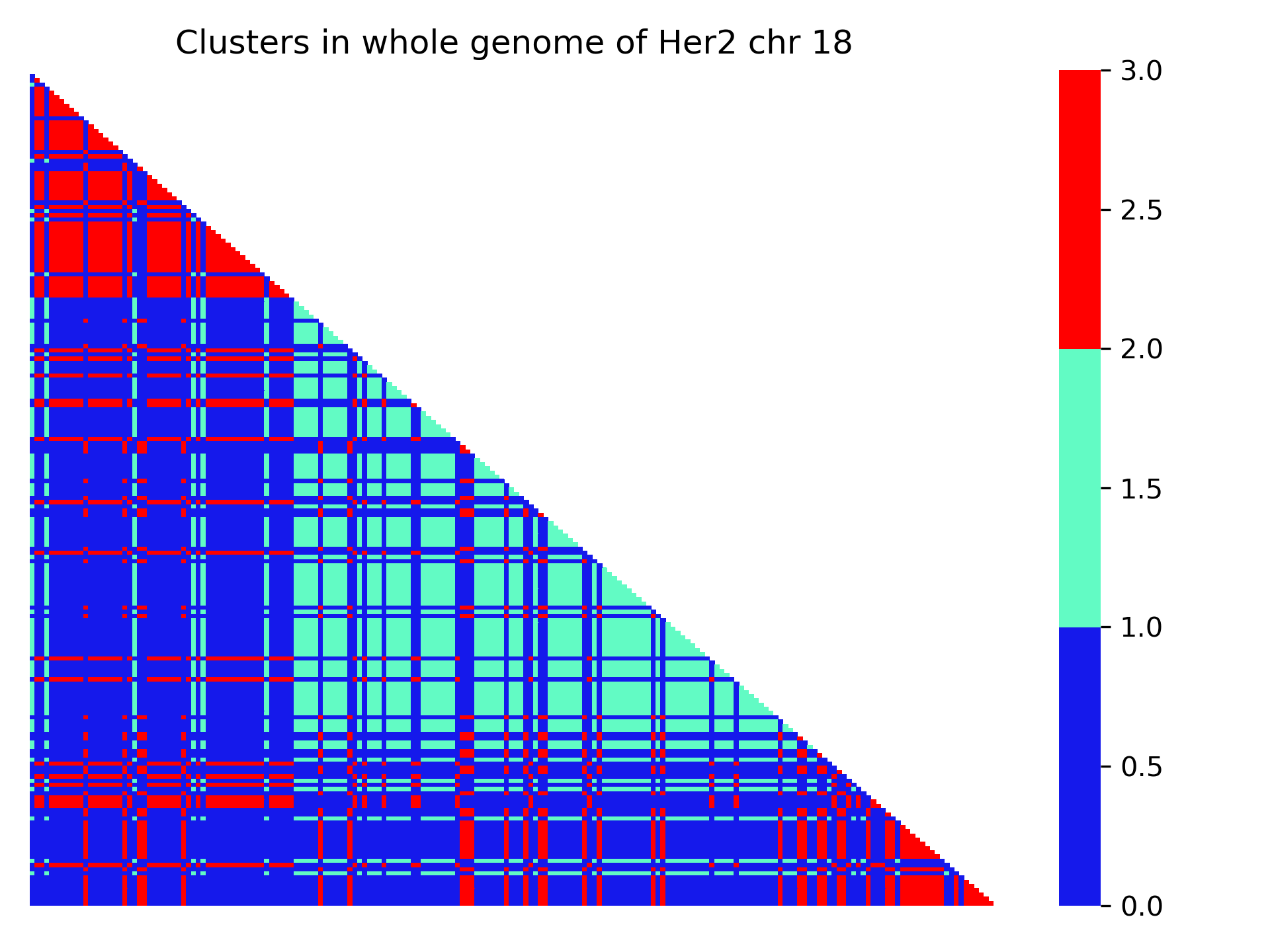

Supplement: Supplementary Material S13 — Piece-wise permutation p-values of the KS statistics, calculated for all bins obtained in Supplementary Material S8 , in every chromosomal region for each phenotype. [file DataSheet_13.zip › SuppMat10/SuppMat10/chr18/Her2-chr18-gstart-heat.png]

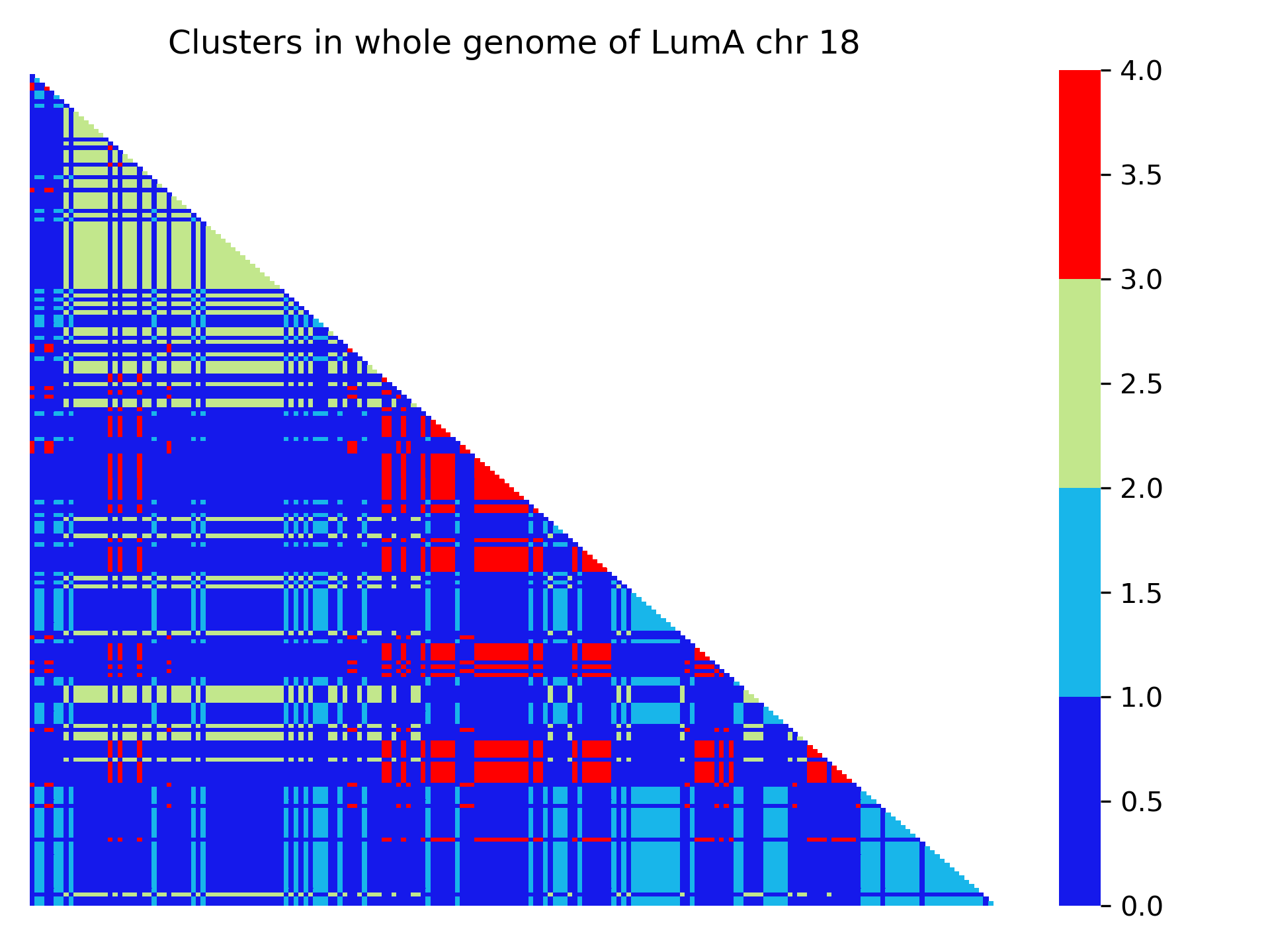

Supplement: Supplementary Material S13 — Piece-wise permutation p-values of the KS statistics, calculated for all bins obtained in Supplementary Material S8 , in every chromosomal region for each phenotype. [file DataSheet_13.zip › SuppMat10/SuppMat10/chr18/LumA-chr18-gstart-heat.png]

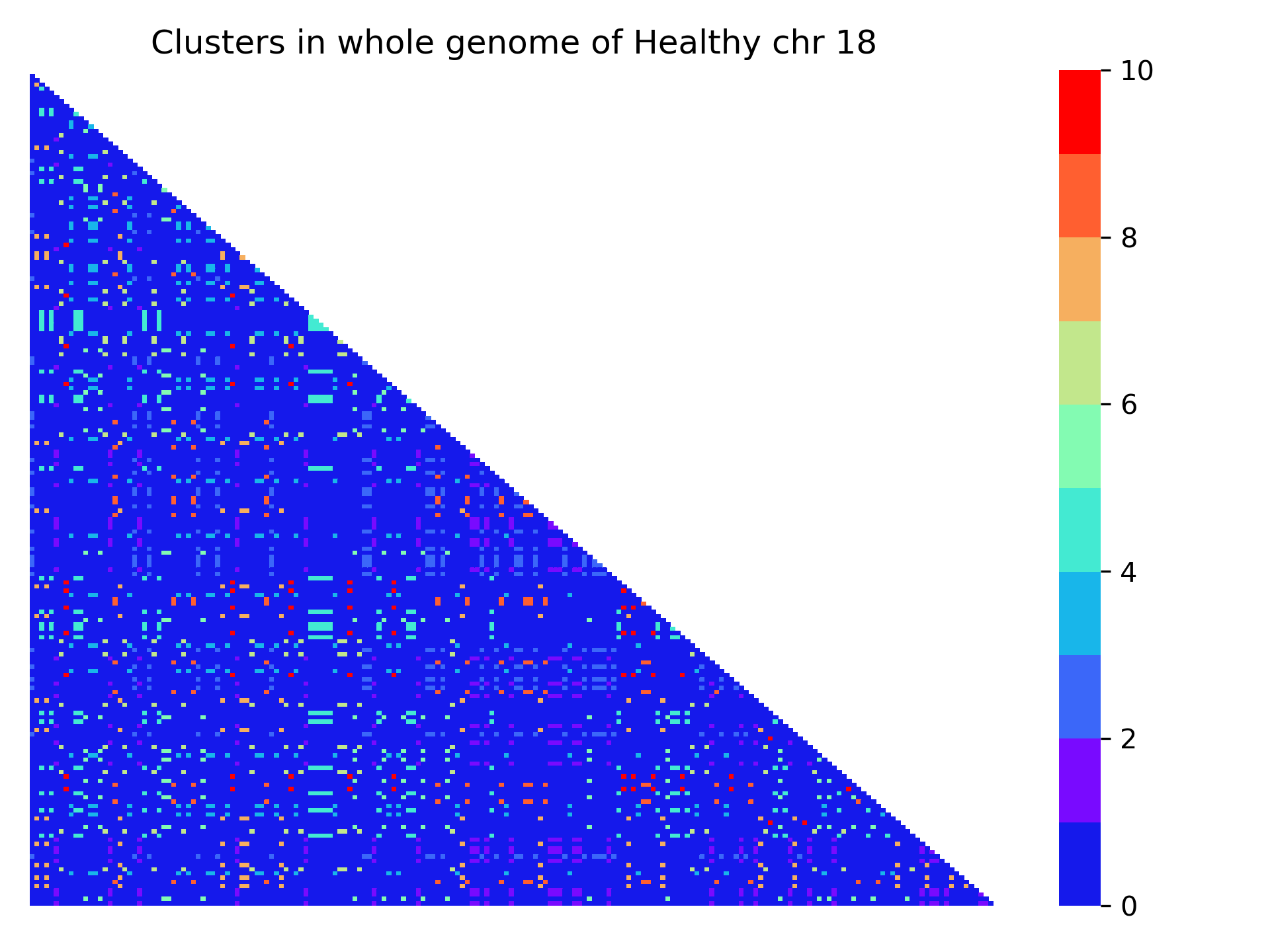

Supplement: Supplementary Material S13 — Piece-wise permutation p-values of the KS statistics, calculated for all bins obtained in Supplementary Material S8 , in every chromosomal region for each phenotype. [file DataSheet_13.zip › SuppMat10/SuppMat10/chr18/Healthy-chr18-gstart-heat.png]

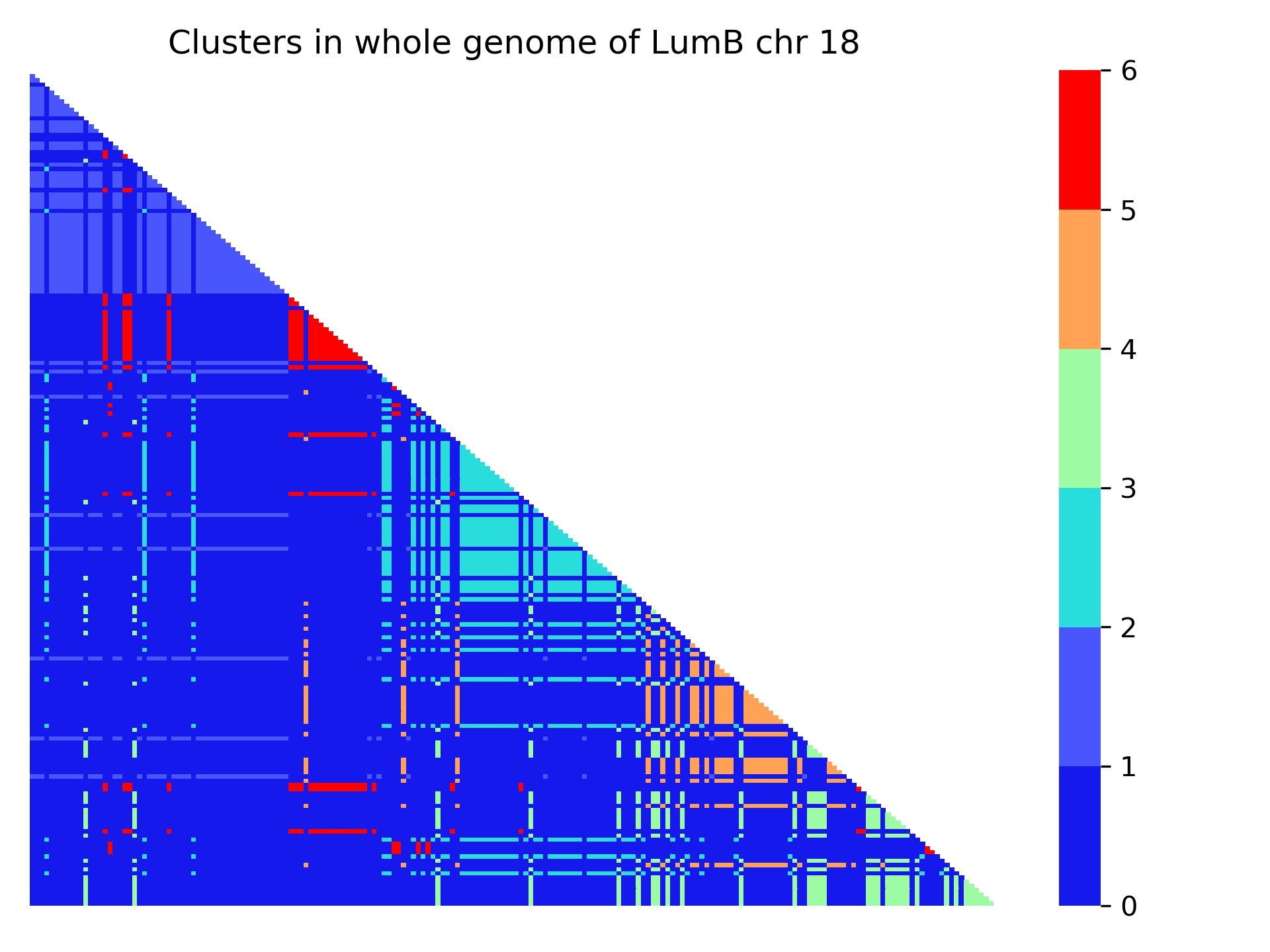

Supplement: Supplementary Material S13 — Piece-wise permutation p-values of the KS statistics, calculated for all bins obtained in Supplementary Material S8 , in every chromosomal region for each phenotype. [file DataSheet_13.zip › SuppMat10/SuppMat10/chr18/LumB-chr18-gstart-heat.png]

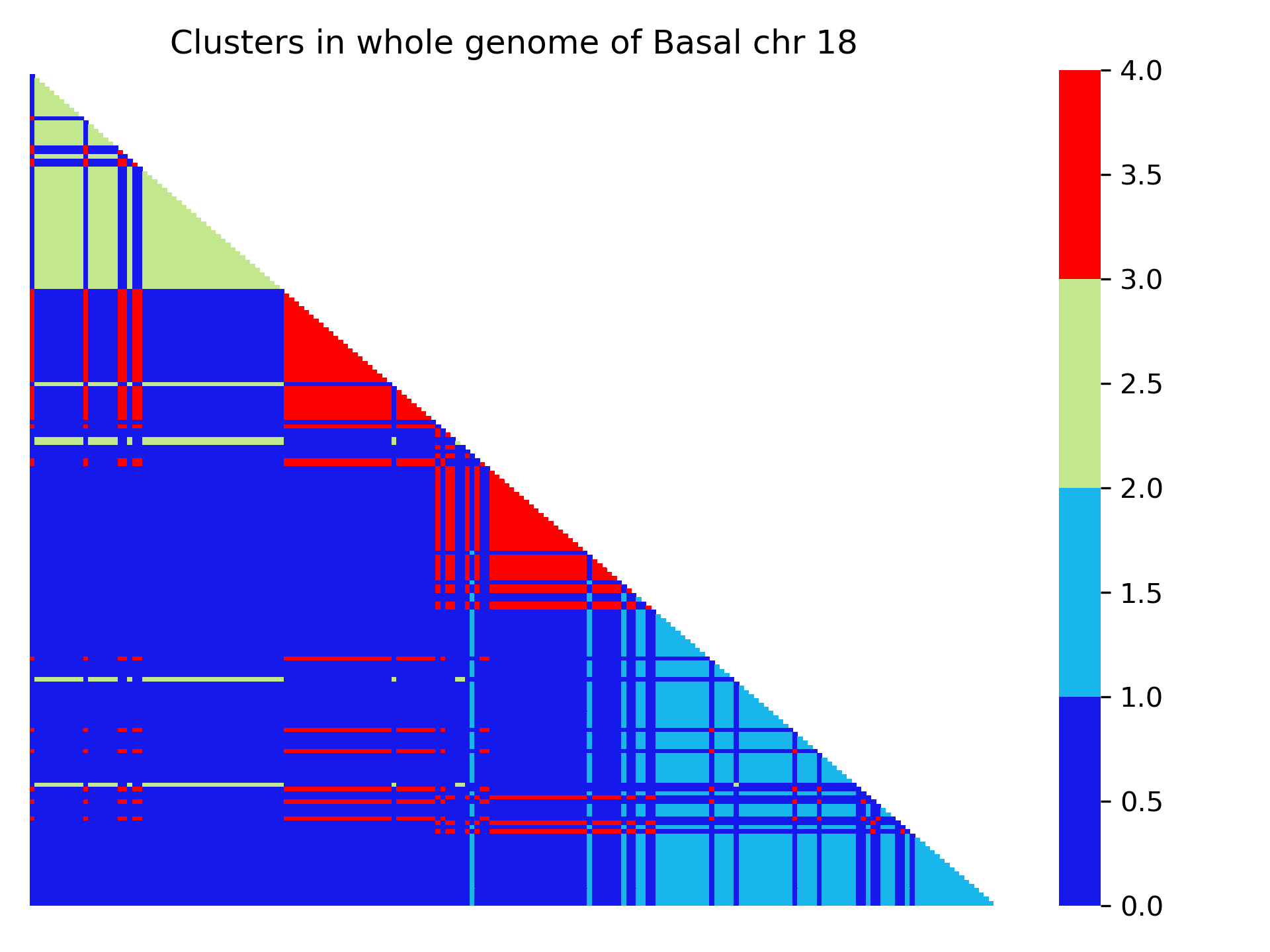

Supplement: Supplementary Material S13 — Piece-wise permutation p-values of the KS statistics, calculated for all bins obtained in Supplementary Material S8 , in every chromosomal region for each phenotype. [file DataSheet_13.zip › SuppMat10/SuppMat10/chr18/Basal-chr18-gstart-heat.png]

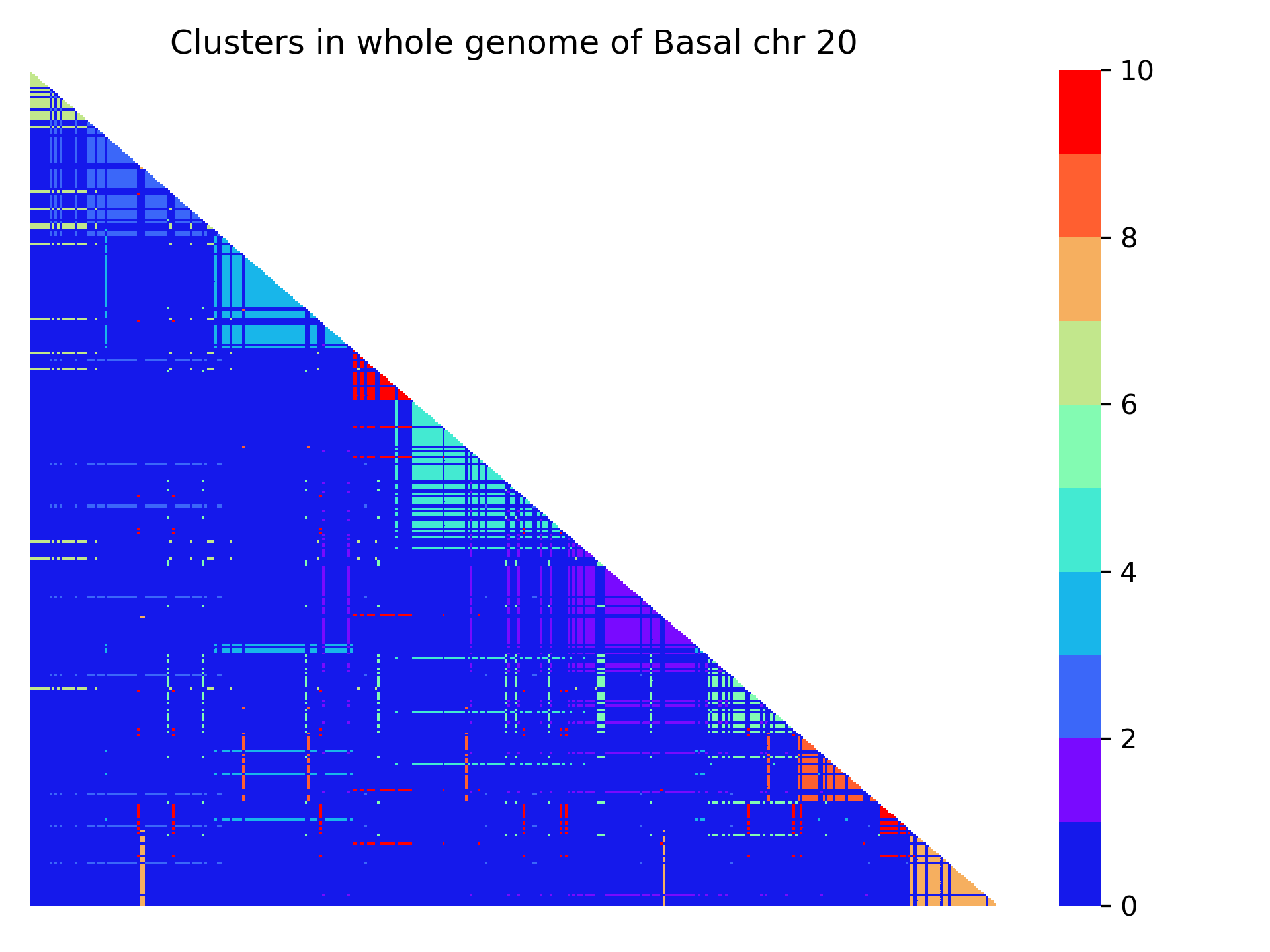

Supplement: Supplementary Material S13 — Piece-wise permutation p-values of the KS statistics, calculated for all bins obtained in Supplementary Material S8 , in every chromosomal region for each phenotype. [file DataSheet_13.zip › SuppMat10/SuppMat10/chr20/Basal-chr20-gstart-heat.png]

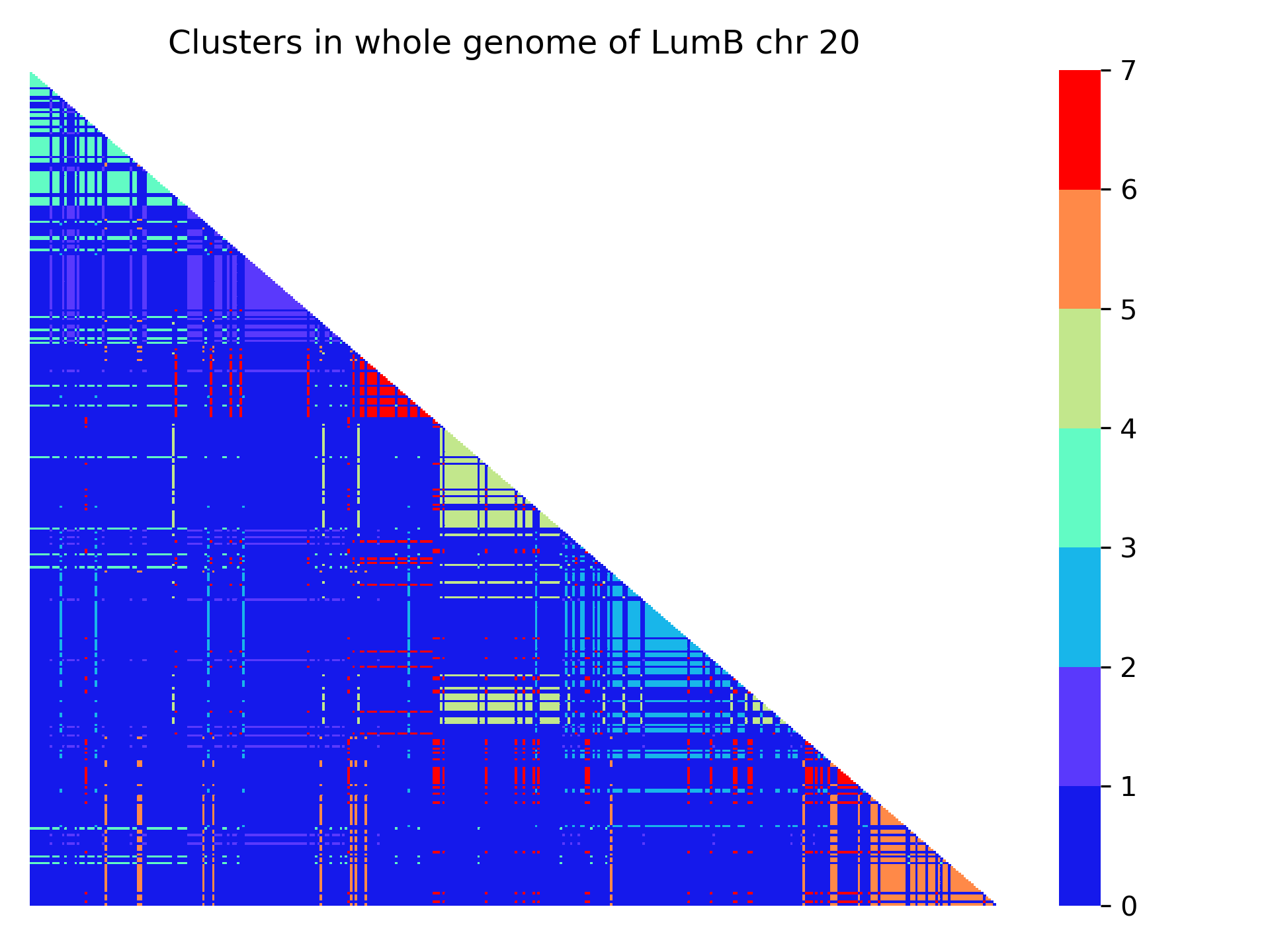

Supplement: Supplementary Material S13 — Piece-wise permutation p-values of the KS statistics, calculated for all bins obtained in Supplementary Material S8 , in every chromosomal region for each phenotype. [file DataSheet_13.zip › SuppMat10/SuppMat10/chr20/LumB-chr20-gstart-heat.png]

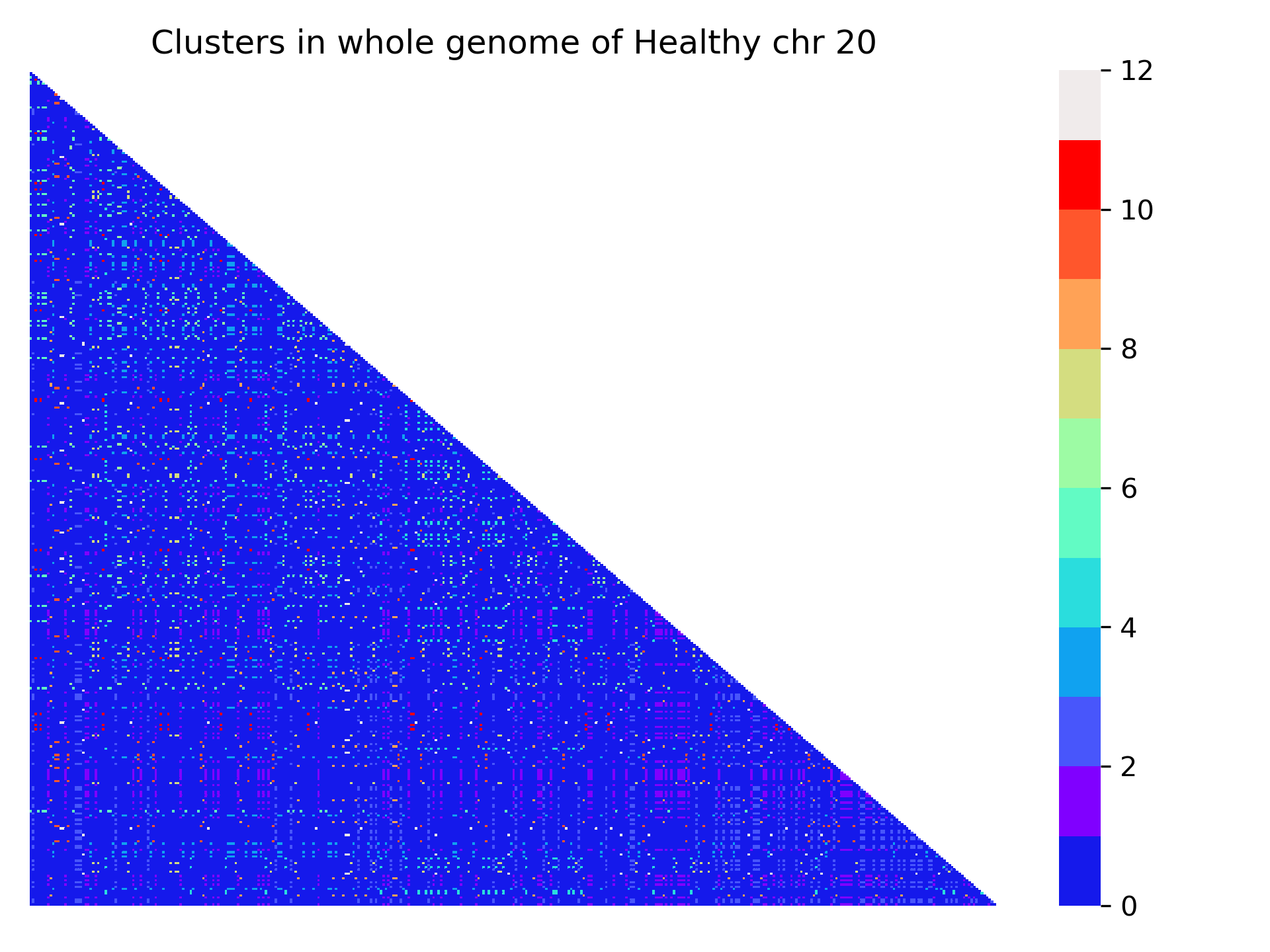

Supplement: Supplementary Material S13 — Piece-wise permutation p-values of the KS statistics, calculated for all bins obtained in Supplementary Material S8 , in every chromosomal region for each phenotype. [file DataSheet_13.zip › SuppMat10/SuppMat10/chr20/Healthy-chr20-gstart-heat.png]

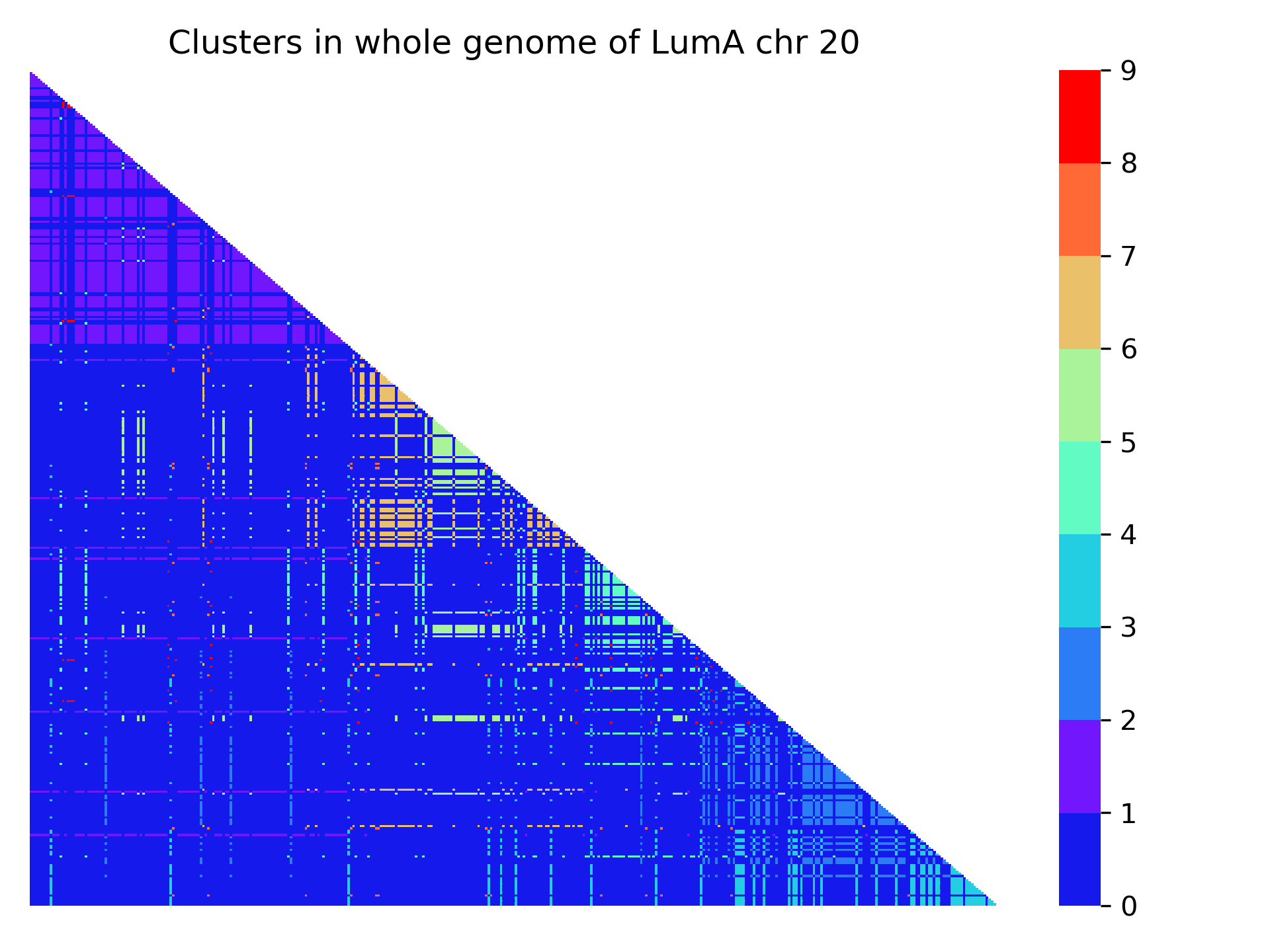

Supplement: Supplementary Material S13 — Piece-wise permutation p-values of the KS statistics, calculated for all bins obtained in Supplementary Material S8 , in every chromosomal region for each phenotype. [file DataSheet_13.zip › SuppMat10/SuppMat10/chr20/LumA-chr20-gstart-heat.png]

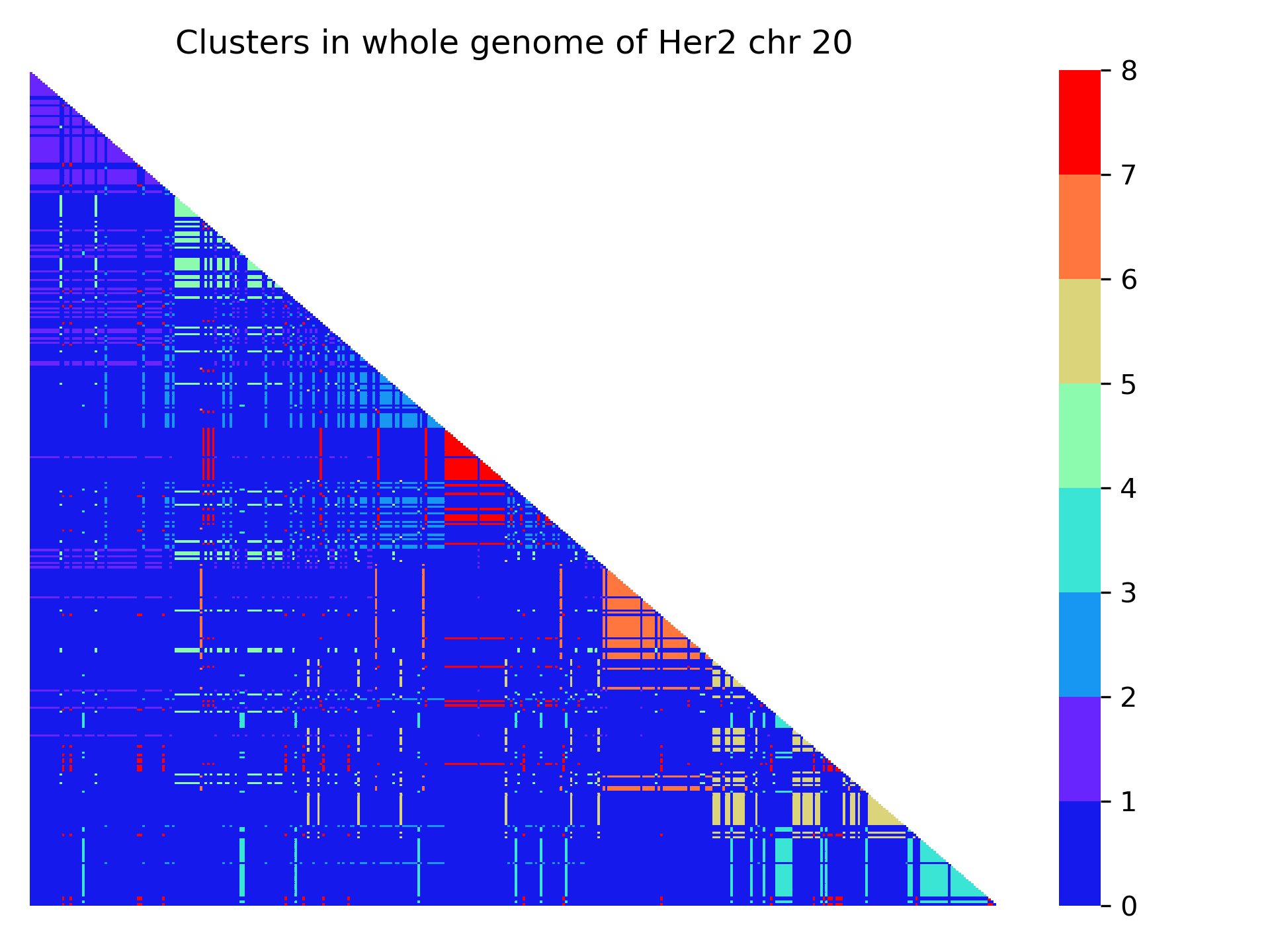

Supplement: Supplementary Material S13 — Piece-wise permutation p-values of the KS statistics, calculated for all bins obtained in Supplementary Material S8 , in every chromosomal region for each phenotype. [file DataSheet_13.zip › SuppMat10/SuppMat10/chr20/Her2-chr20-gstart-heat.png]

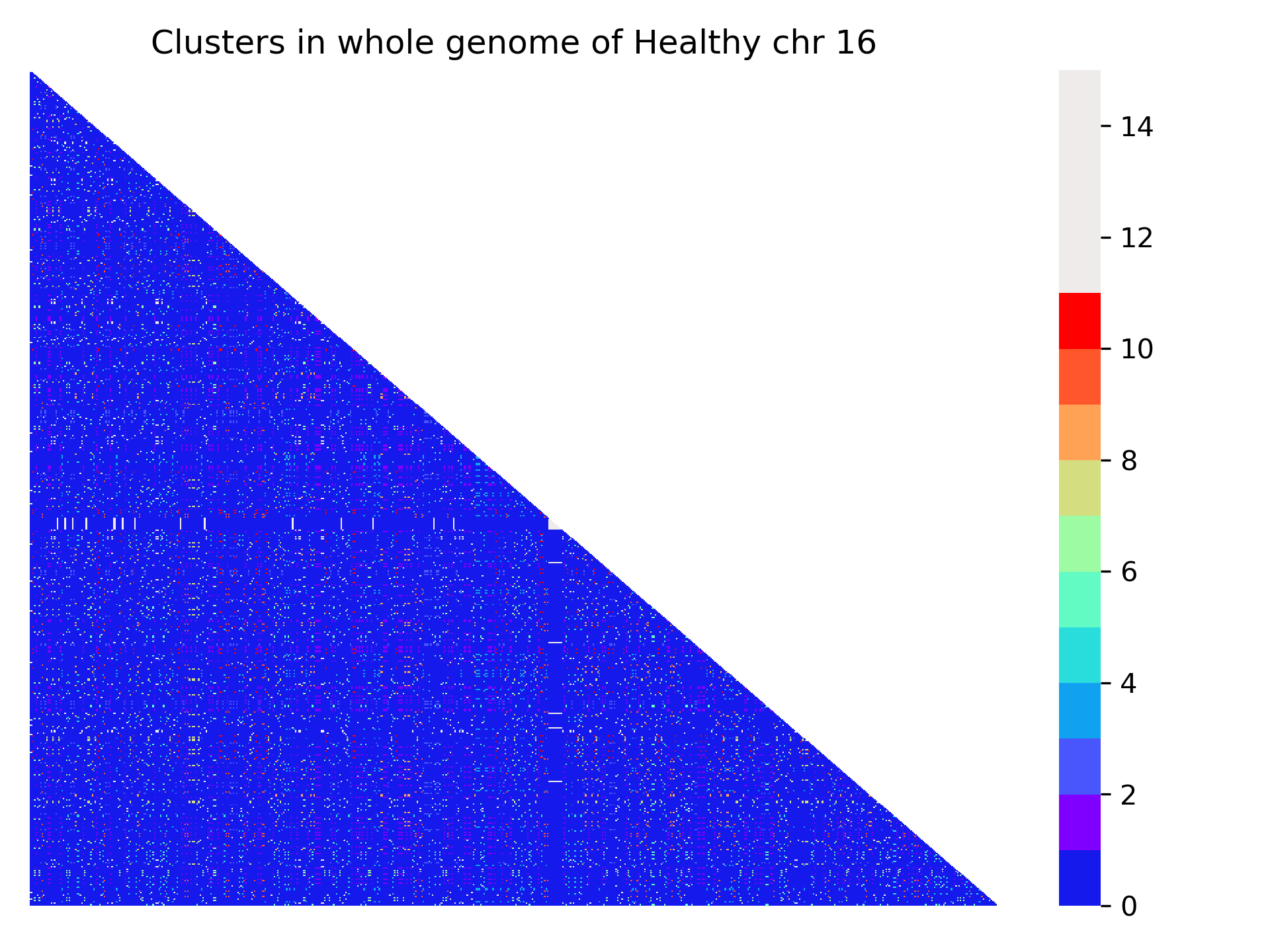

Supplement: Supplementary Material S13 — Piece-wise permutation p-values of the KS statistics, calculated for all bins obtained in Supplementary Material S8 , in every chromosomal region for each phenotype. [file DataSheet_13.zip › SuppMat10/SuppMat10/chr16/Healthy-chr16-gstart-heat.png]

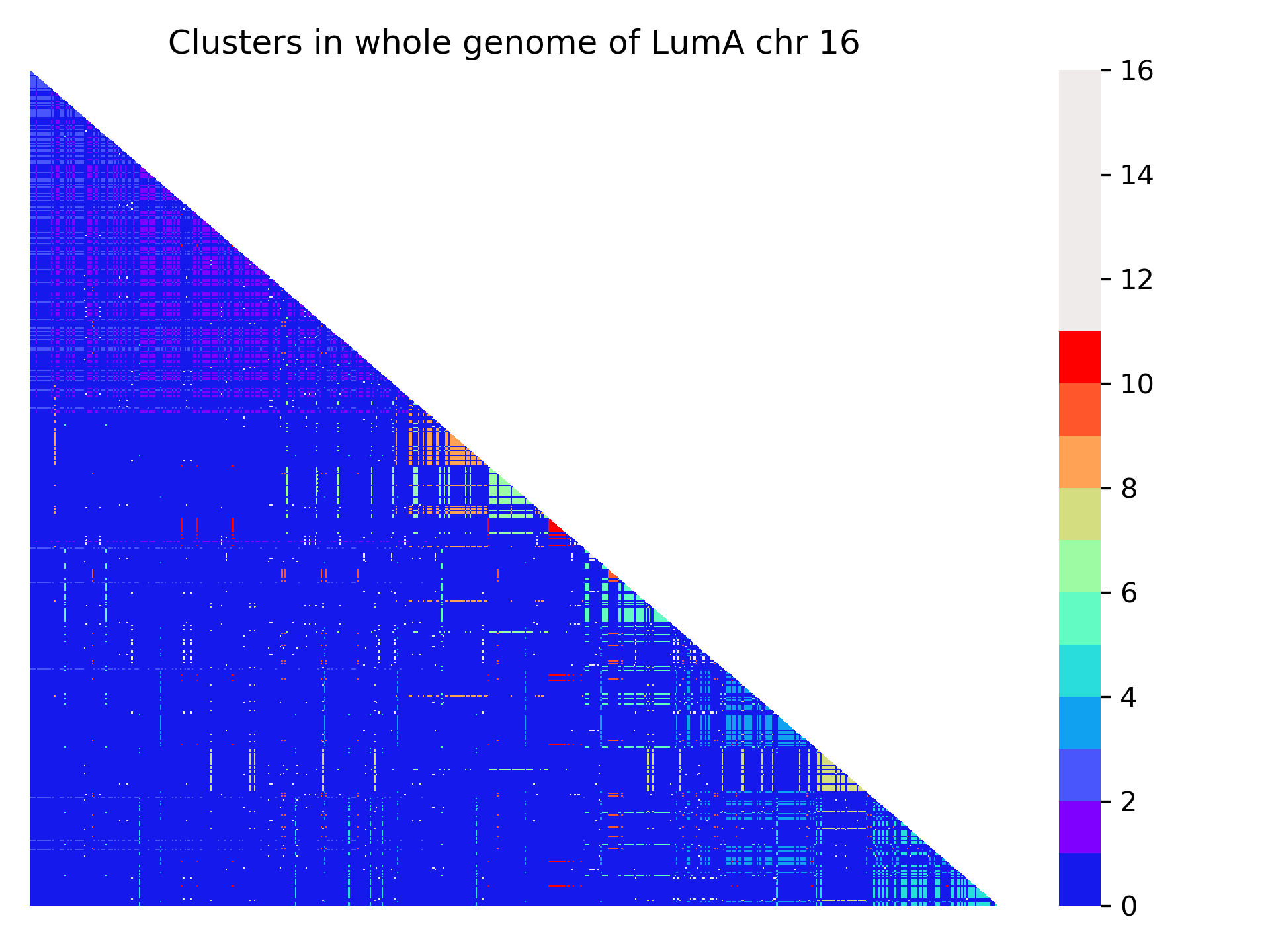

Supplement: Supplementary Material S13 — Piece-wise permutation p-values of the KS statistics, calculated for all bins obtained in Supplementary Material S8 , in every chromosomal region for each phenotype. [file DataSheet_13.zip › SuppMat10/SuppMat10/chr16/LumA-chr16-gstart-heat.png]

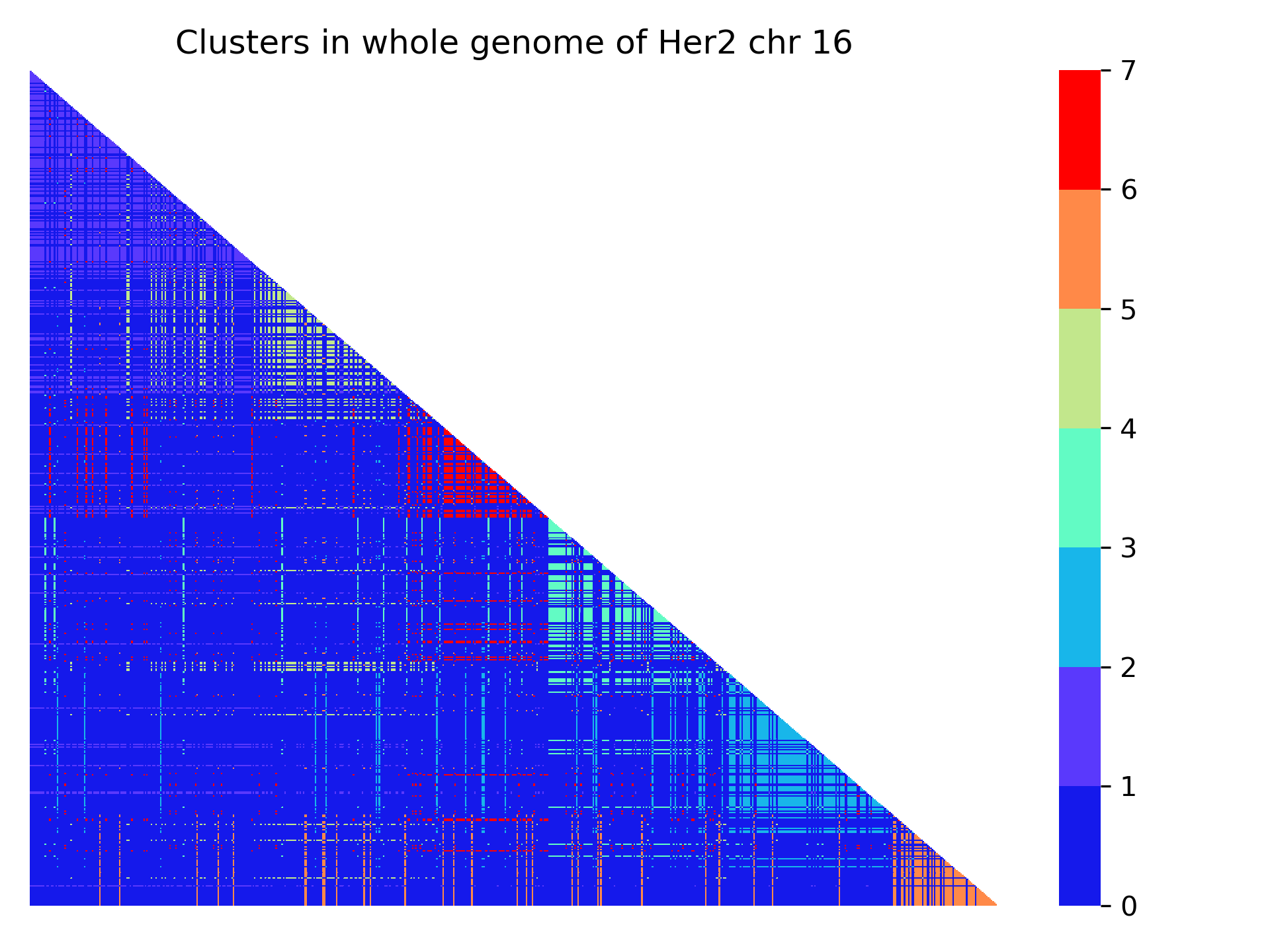

Supplement: Supplementary Material S13 — Piece-wise permutation p-values of the KS statistics, calculated for all bins obtained in Supplementary Material S8 , in every chromosomal region for each phenotype. [file DataSheet_13.zip › SuppMat10/SuppMat10/chr16/Her2-chr16-gstart-heat.png]

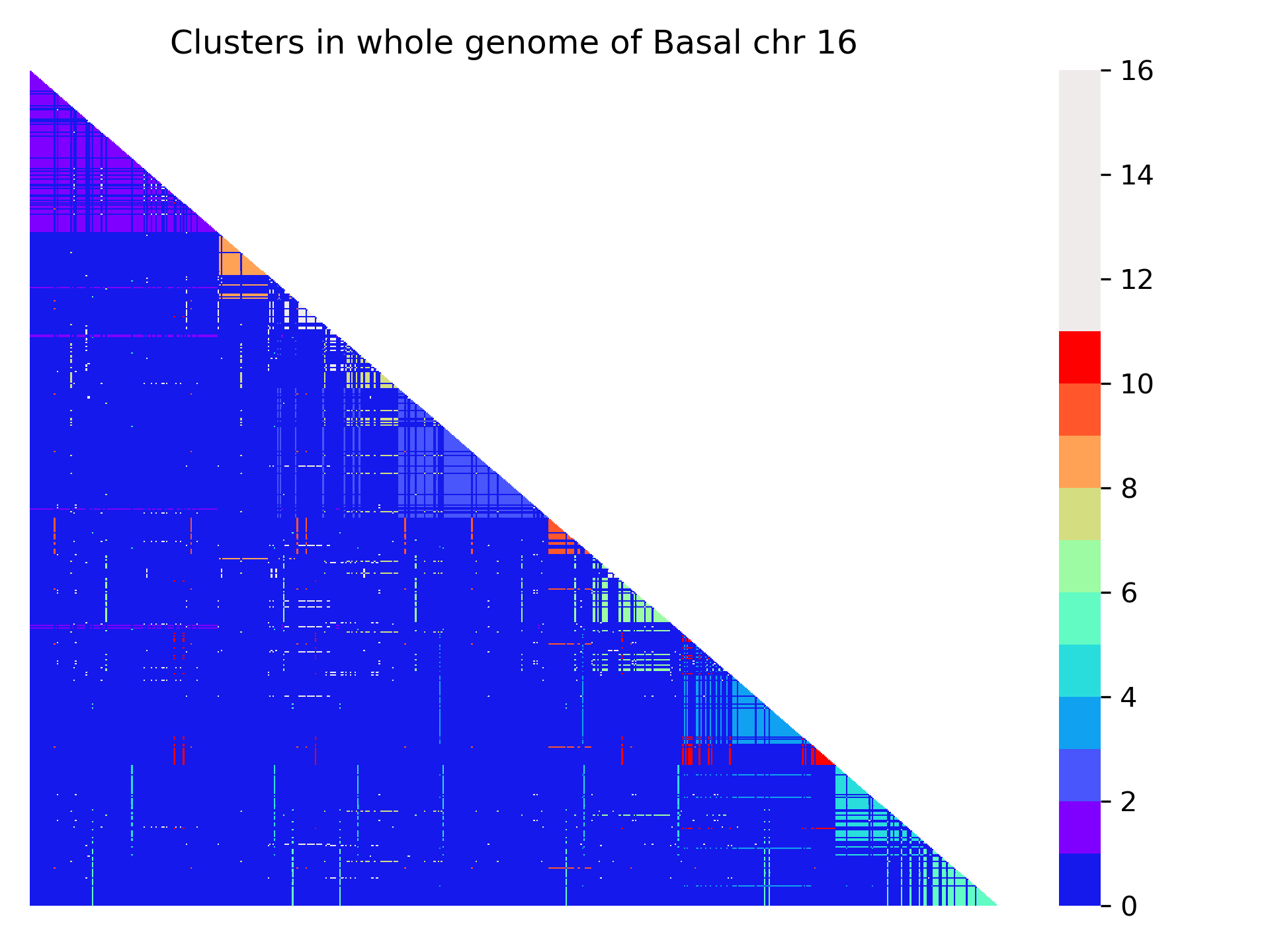

Supplement: Supplementary Material S13 — Piece-wise permutation p-values of the KS statistics, calculated for all bins obtained in Supplementary Material S8 , in every chromosomal region for each phenotype. [file DataSheet_13.zip › SuppMat10/SuppMat10/chr16/Basal-chr16-gstart-heat.png]

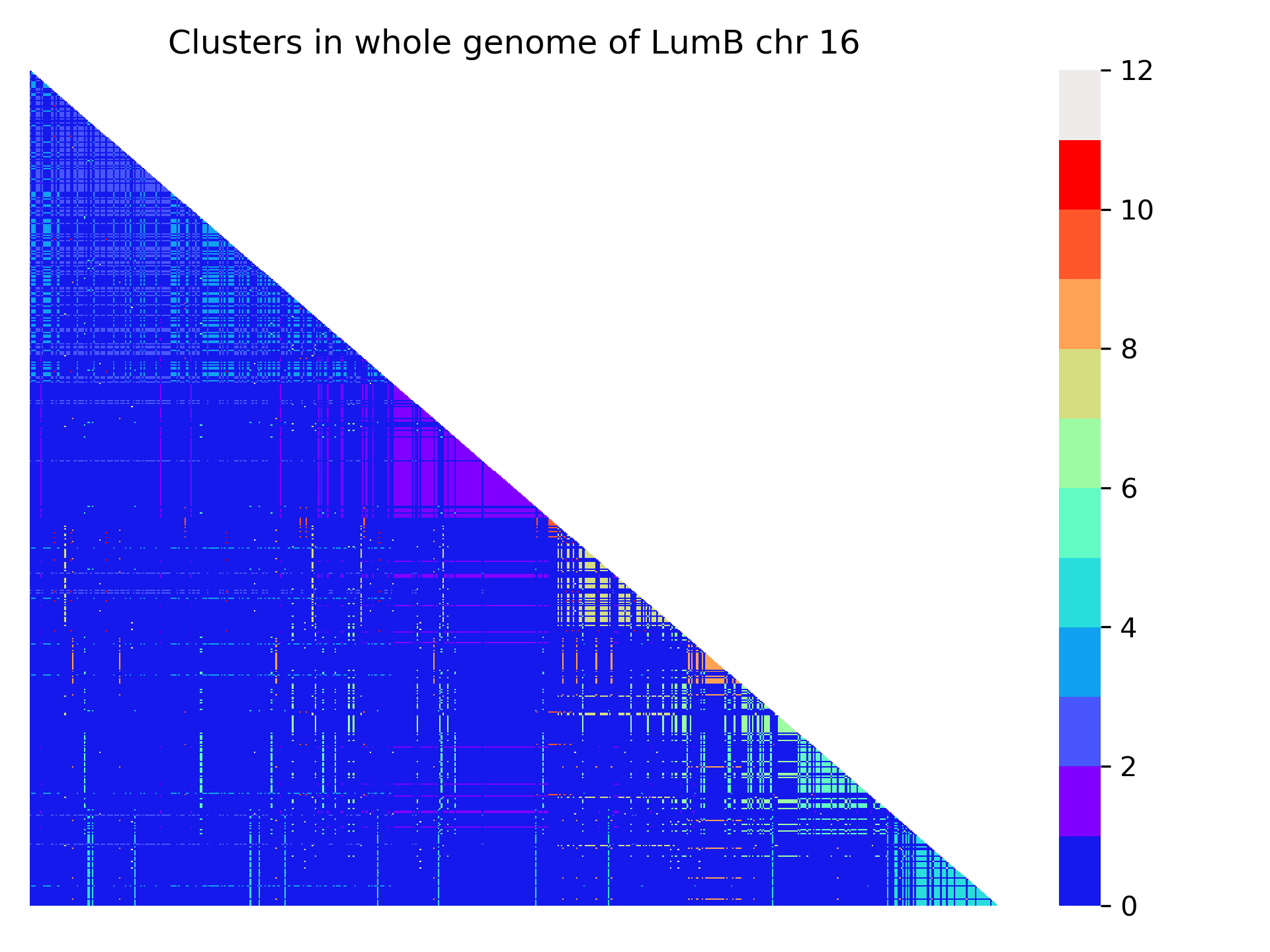

Supplement: Supplementary Material S13 — Piece-wise permutation p-values of the KS statistics, calculated for all bins obtained in Supplementary Material S8 , in every chromosomal region for each phenotype. [file DataSheet_13.zip › SuppMat10/SuppMat10/chr16/LumB-chr16-gstart-heat.png]

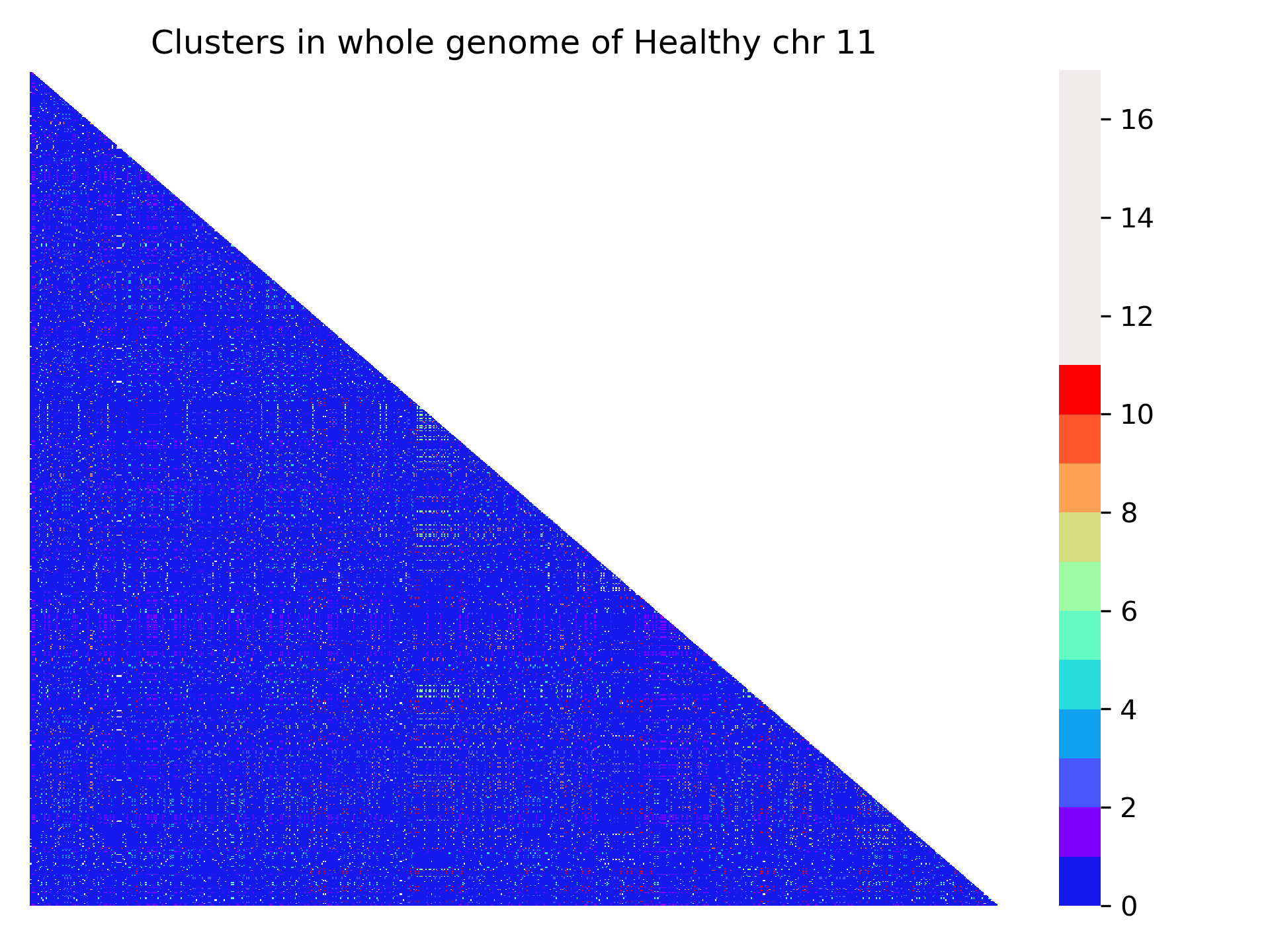

Supplement: Supplementary Material S13 — Piece-wise permutation p-values of the KS statistics, calculated for all bins obtained in Supplementary Material S8 , in every chromosomal region for each phenotype. [file DataSheet_13.zip › SuppMat10/SuppMat10/chr11/Healthy-chr11-gstart-heat.png]

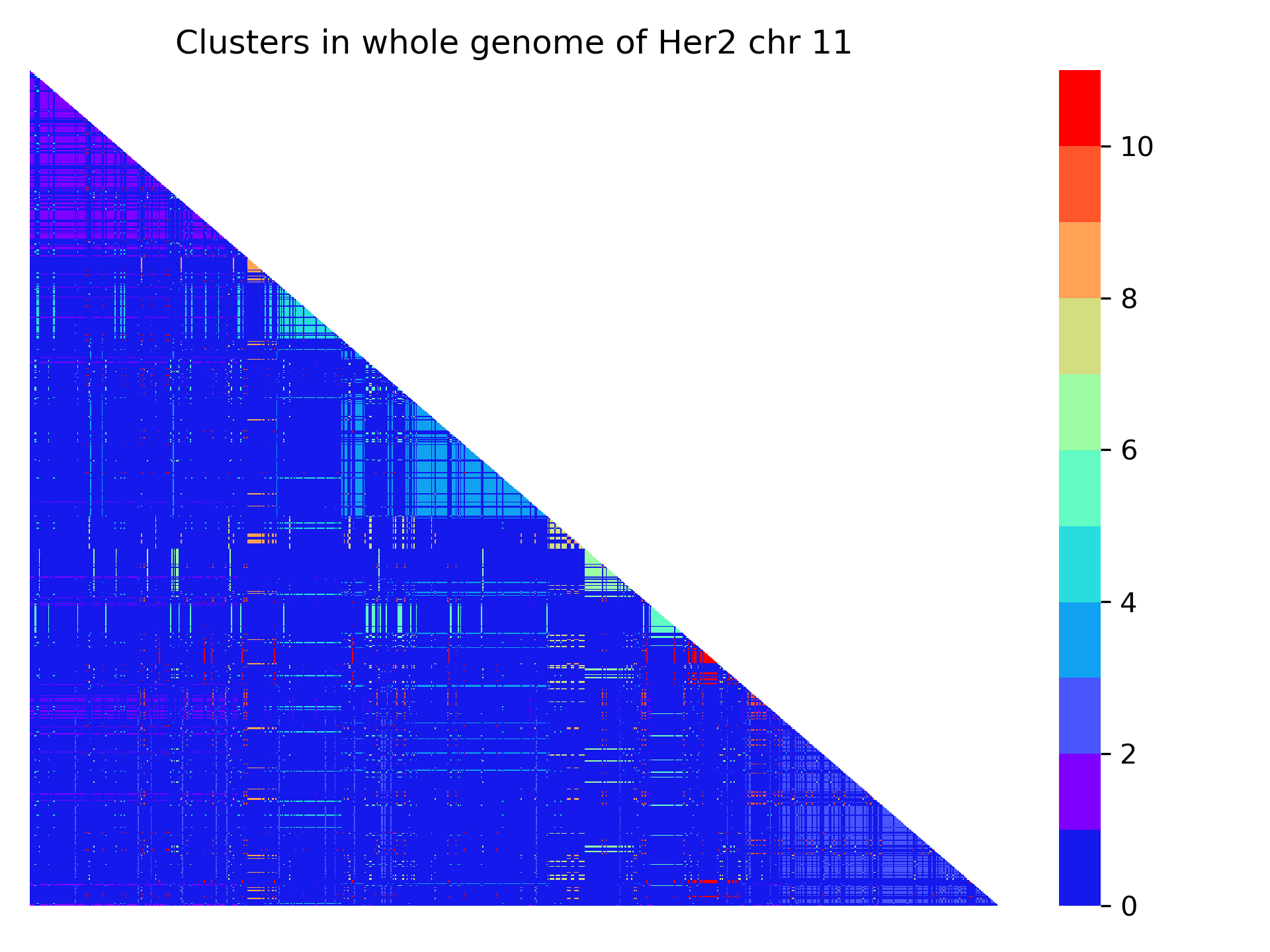

Supplement: Supplementary Material S13 — Piece-wise permutation p-values of the KS statistics, calculated for all bins obtained in Supplementary Material S8 , in every chromosomal region for each phenotype. [file DataSheet_13.zip › SuppMat10/SuppMat10/chr11/Her2-chr11-gstart-heat.png]

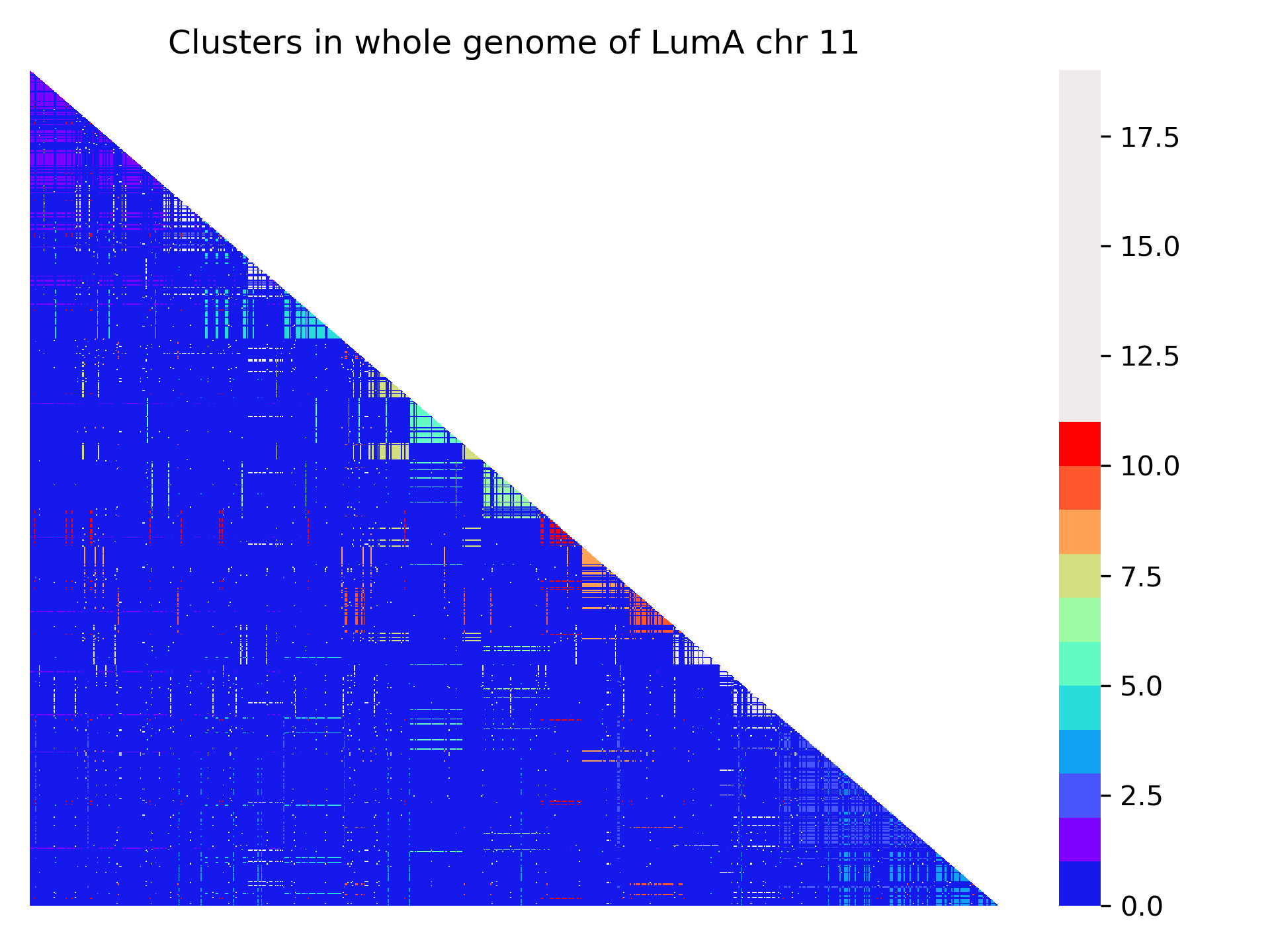

Supplement: Supplementary Material S13 — Piece-wise permutation p-values of the KS statistics, calculated for all bins obtained in Supplementary Material S8 , in every chromosomal region for each phenotype. [file DataSheet_13.zip › SuppMat10/SuppMat10/chr11/LumA-chr11-gstart-heat.png]

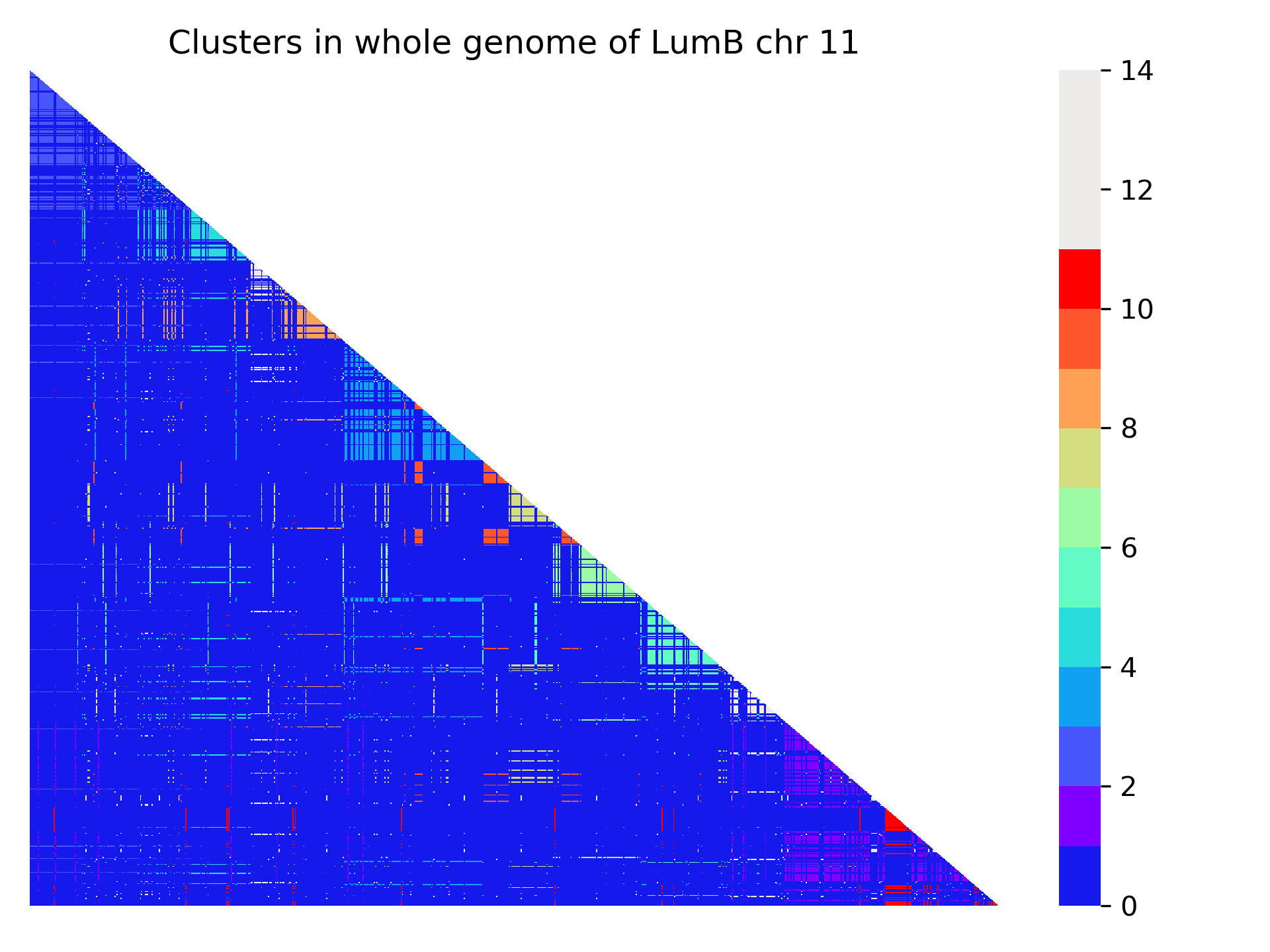

Supplement: Supplementary Material S13 — Piece-wise permutation p-values of the KS statistics, calculated for all bins obtained in Supplementary Material S8 , in every chromosomal region for each phenotype. [file DataSheet_13.zip › SuppMat10/SuppMat10/chr11/LumB-chr11-gstart-heat.png]

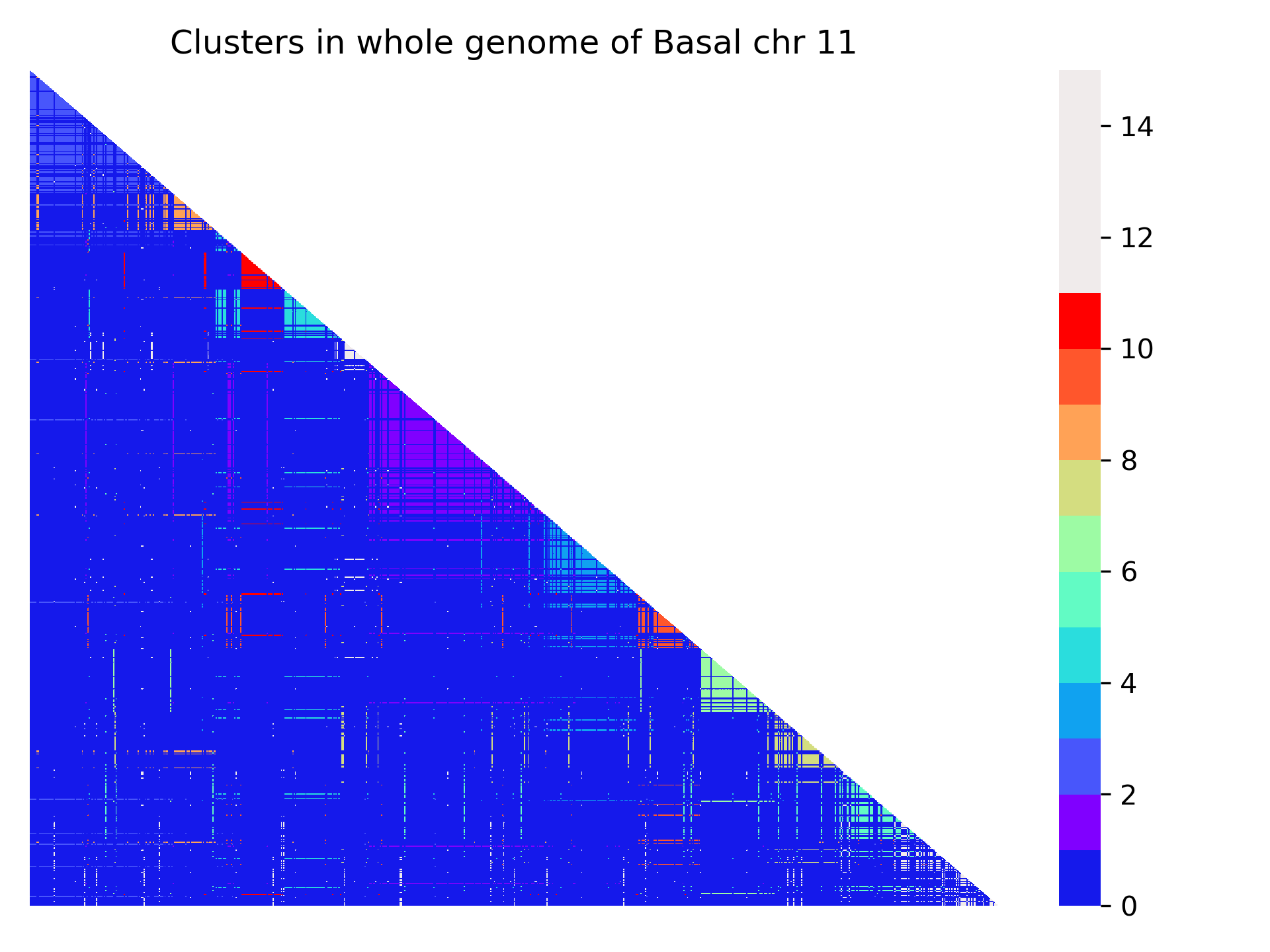

Supplement: Supplementary Material S13 — Piece-wise permutation p-values of the KS statistics, calculated for all bins obtained in Supplementary Material S8 , in every chromosomal region for each phenotype. [file DataSheet_13.zip › SuppMat10/SuppMat10/chr11/Basal-chr11-gstart-heat.png]

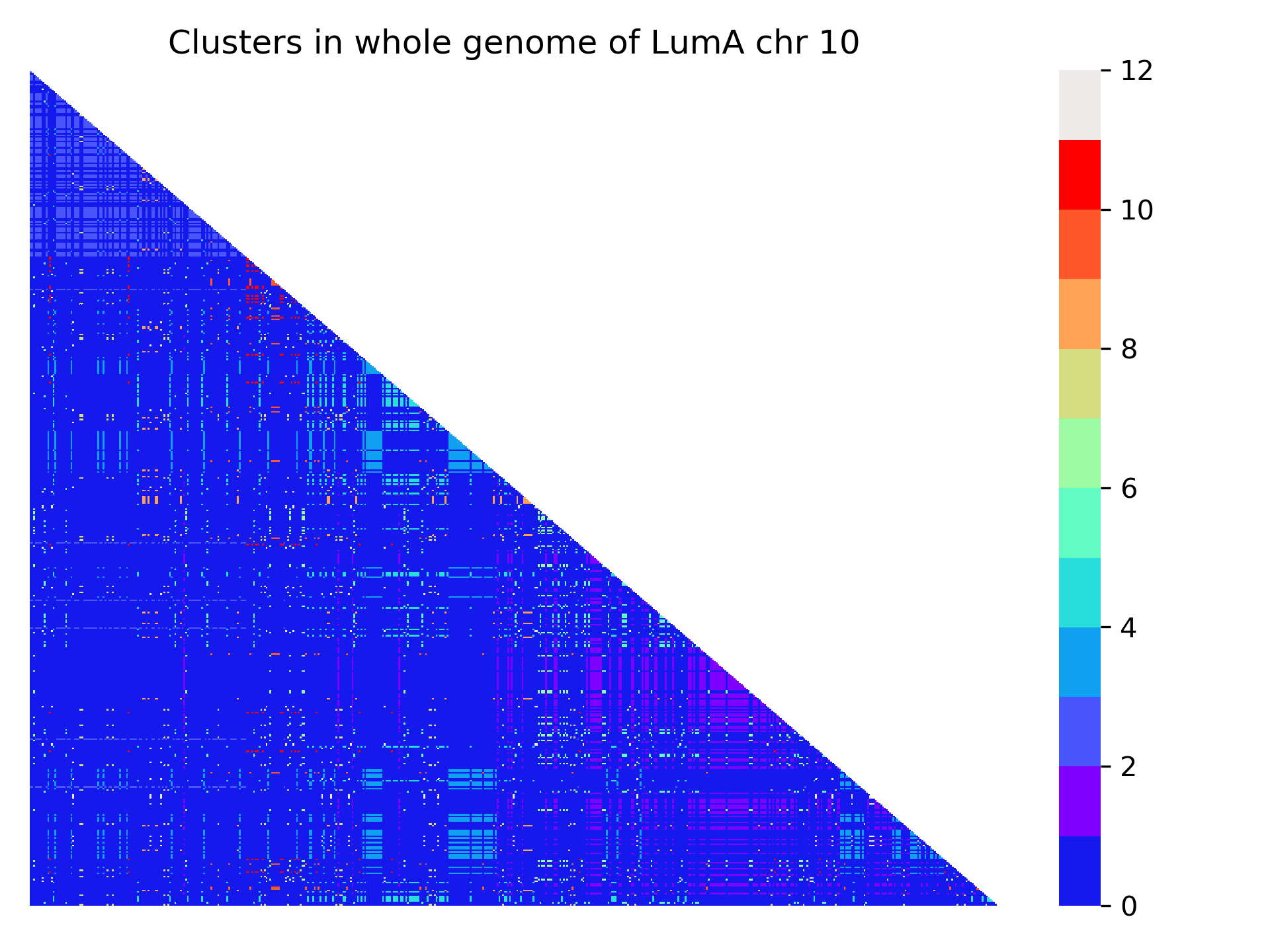

Supplement: Supplementary Material S13 — Piece-wise permutation p-values of the KS statistics, calculated for all bins obtained in Supplementary Material S8 , in every chromosomal region for each phenotype. [file DataSheet_13.zip › SuppMat10/SuppMat10/chr10/LumA-chr10-gstart-heat.png]

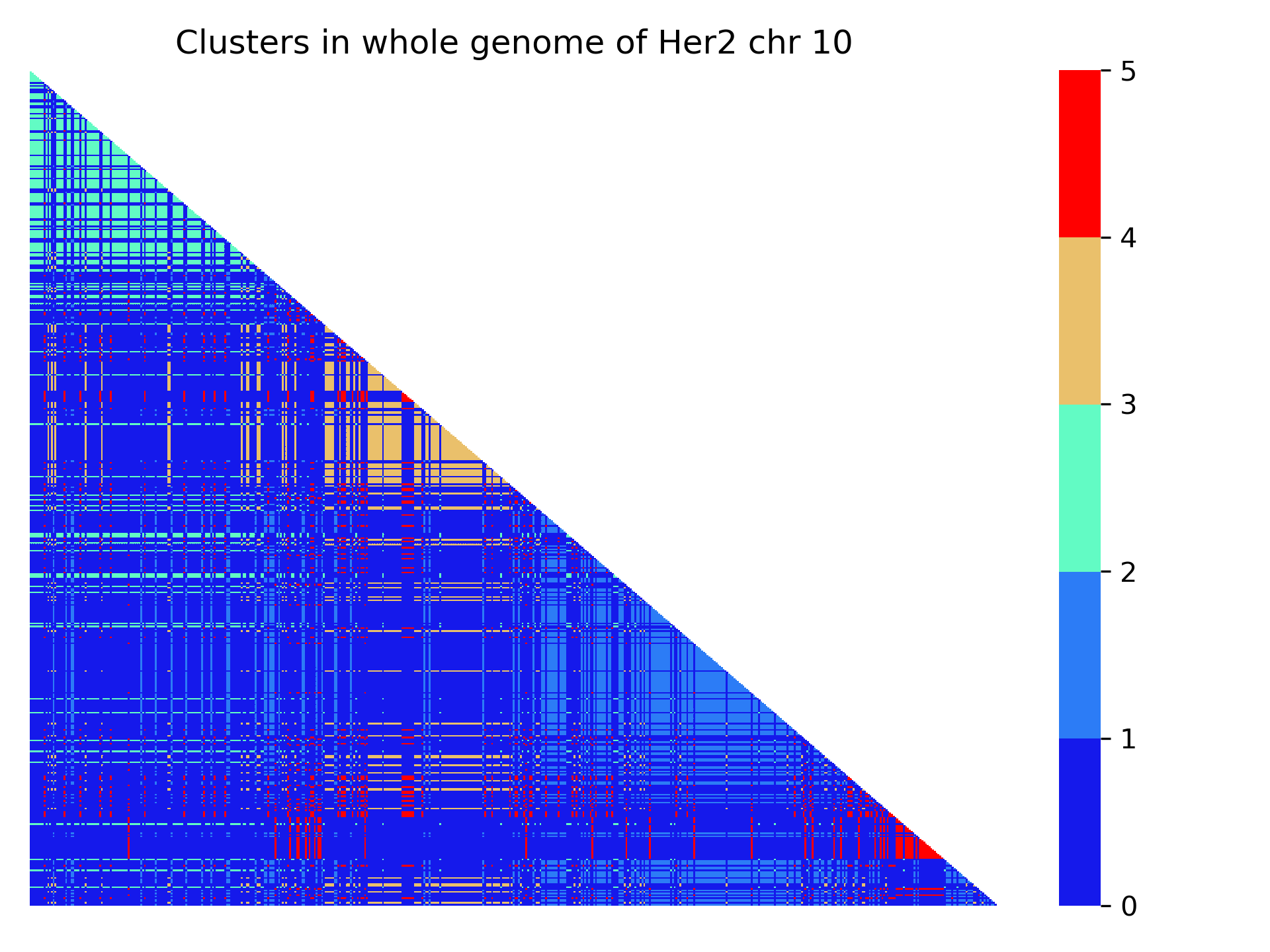

Supplement: Supplementary Material S13 — Piece-wise permutation p-values of the KS statistics, calculated for all bins obtained in Supplementary Material S8 , in every chromosomal region for each phenotype. [file DataSheet_13.zip › SuppMat10/SuppMat10/chr10/Her2-chr10-gstart-heat.png]

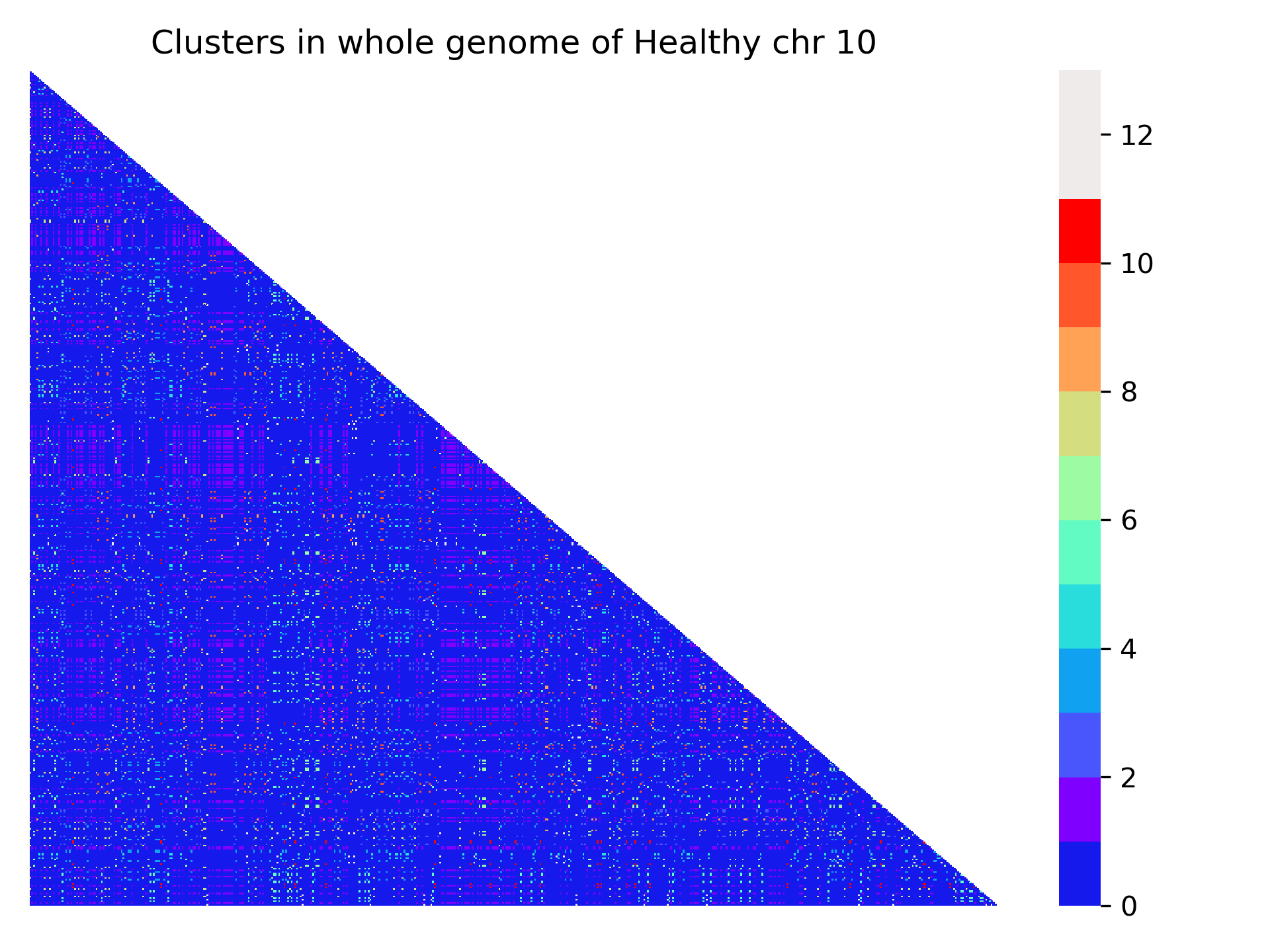

Supplement: Supplementary Material S13 — Piece-wise permutation p-values of the KS statistics, calculated for all bins obtained in Supplementary Material S8 , in every chromosomal region for each phenotype. [file DataSheet_13.zip › SuppMat10/SuppMat10/chr10/Healthy-chr10-gstart-heat.png]

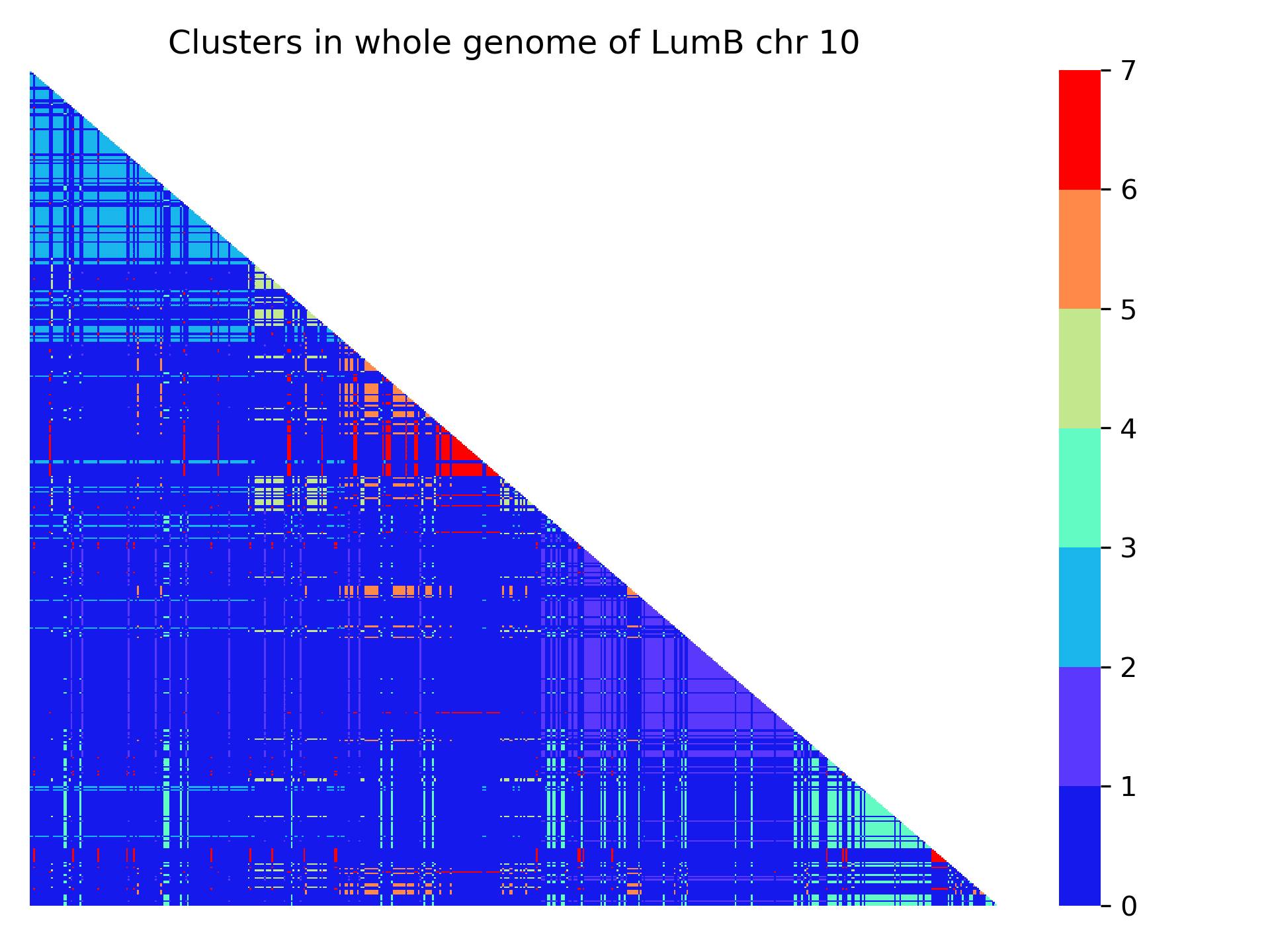

Supplement: Supplementary Material S13 — Piece-wise permutation p-values of the KS statistics, calculated for all bins obtained in Supplementary Material S8 , in every chromosomal region for each phenotype. [file DataSheet_13.zip › SuppMat10/SuppMat10/chr10/LumB-chr10-gstart-heat.png]

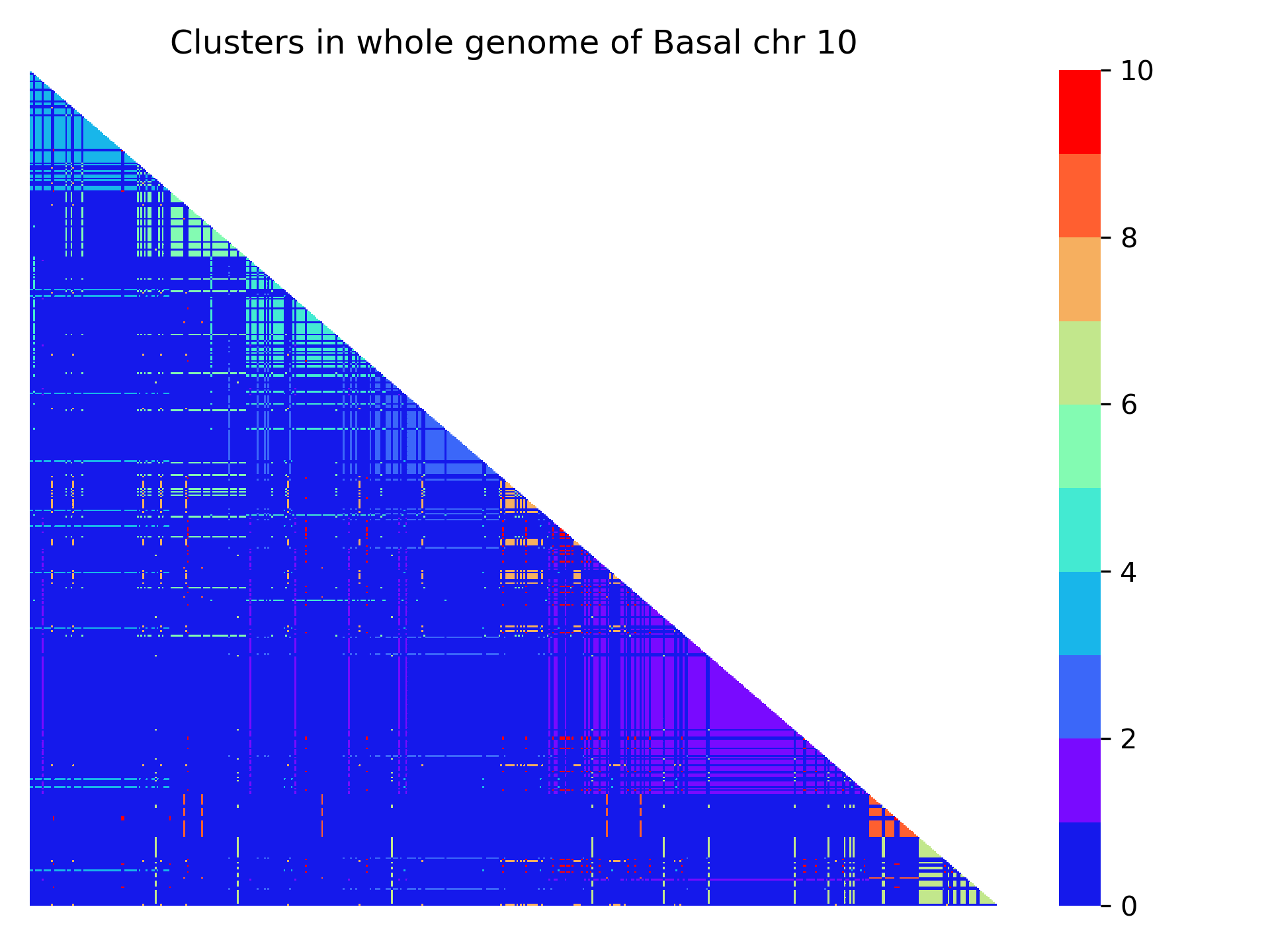

Supplement: Supplementary Material S13 — Piece-wise permutation p-values of the KS statistics, calculated for all bins obtained in Supplementary Material S8 , in every chromosomal region for each phenotype. [file DataSheet_13.zip › SuppMat10/SuppMat10/chr10/Basal-chr10-gstart-heat.png]

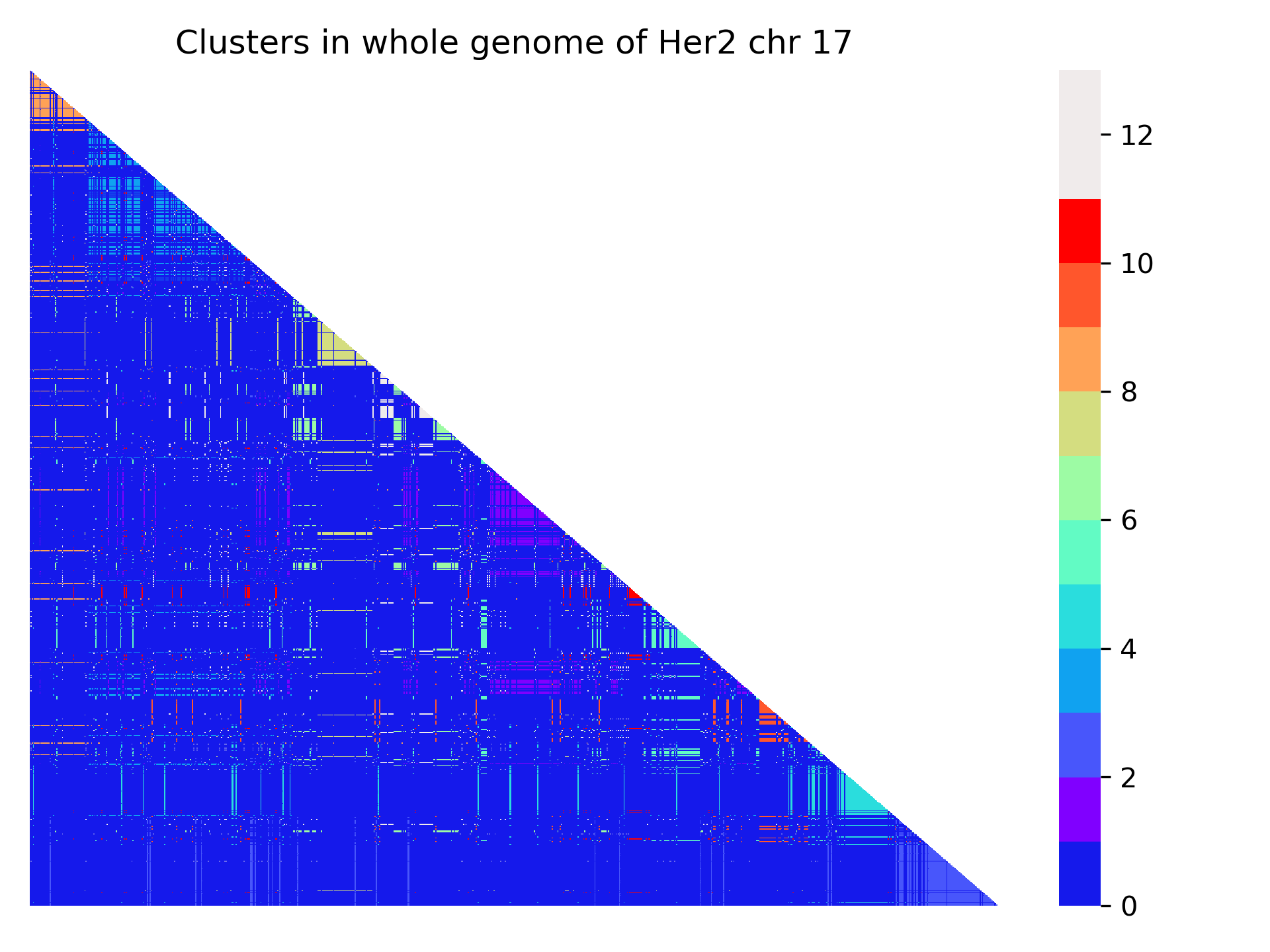

Supplement: Supplementary Material S13 — Piece-wise permutation p-values of the KS statistics, calculated for all bins obtained in Supplementary Material S8 , in every chromosomal region for each phenotype. [file DataSheet_13.zip › SuppMat10/SuppMat10/chr17/Her2-chr17-gstart-heat.png]

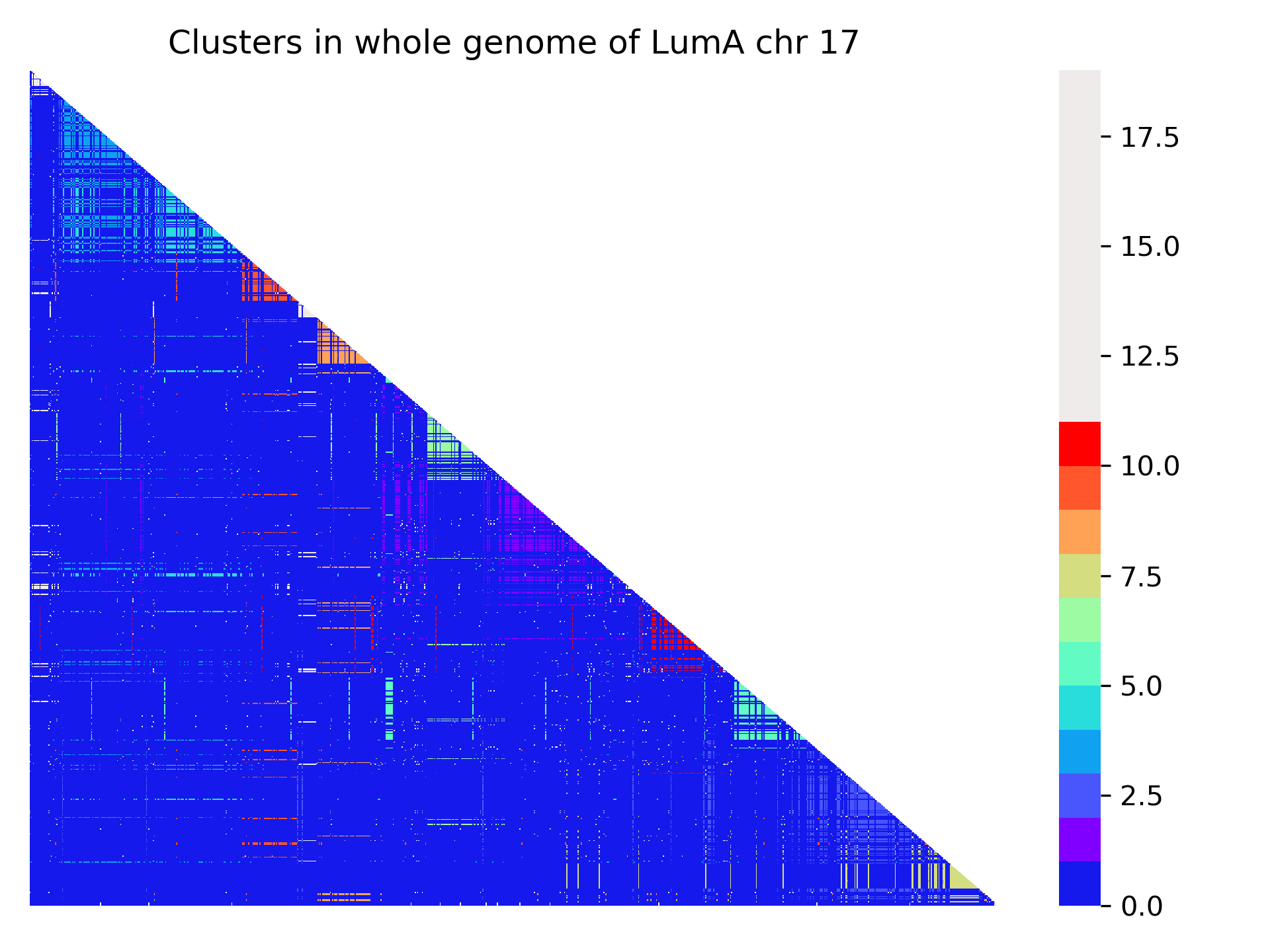

Supplement: Supplementary Material S13 — Piece-wise permutation p-values of the KS statistics, calculated for all bins obtained in Supplementary Material S8 , in every chromosomal region for each phenotype. [file DataSheet_13.zip › SuppMat10/SuppMat10/chr17/LumA-chr17-gstart-heat.png]

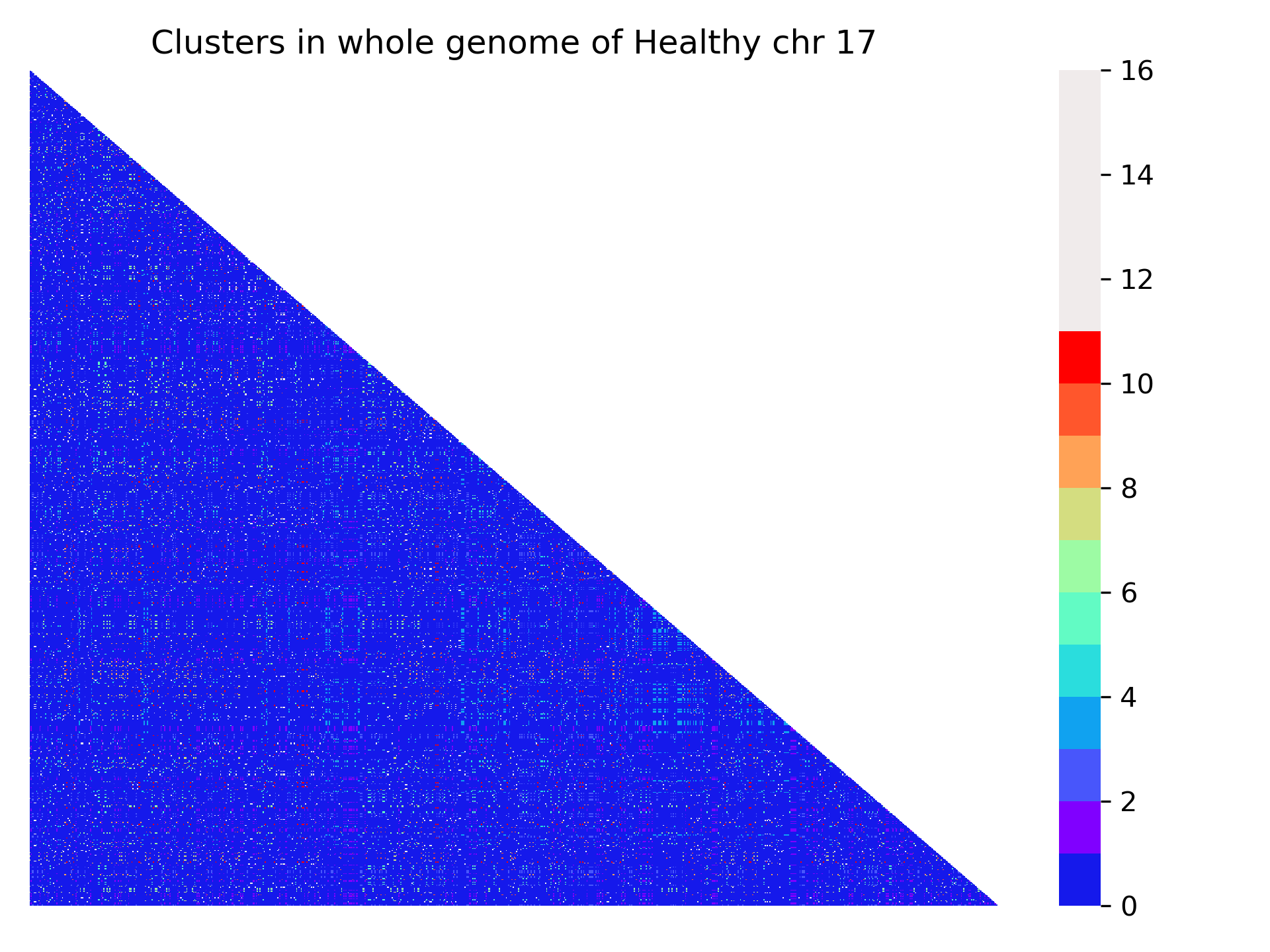

Supplement: Supplementary Material S13 — Piece-wise permutation p-values of the KS statistics, calculated for all bins obtained in Supplementary Material S8 , in every chromosomal region for each phenotype. [file DataSheet_13.zip › SuppMat10/SuppMat10/chr17/Healthy-chr17-gstart-heat.png]

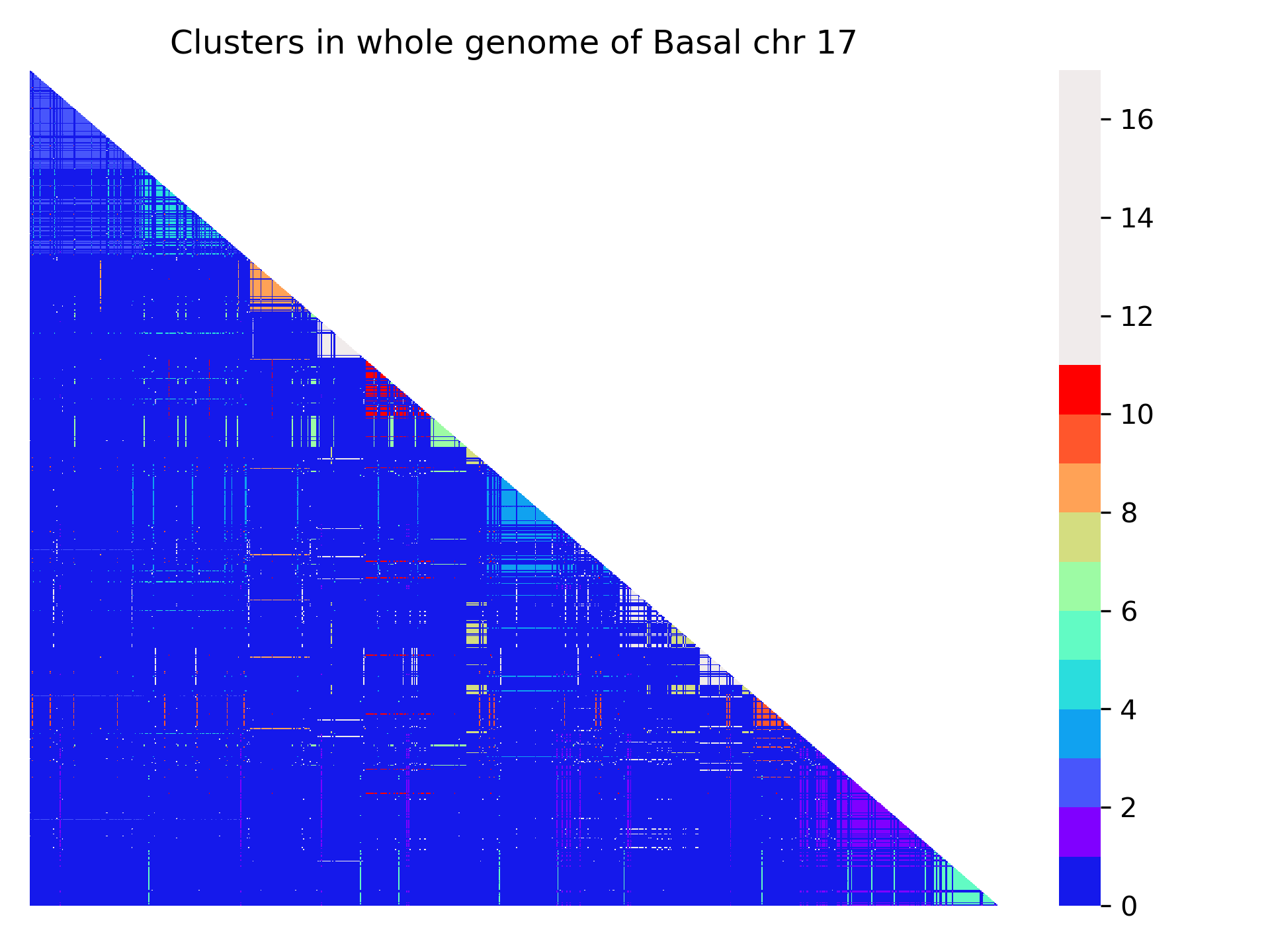

Supplement: Supplementary Material S13 — Piece-wise permutation p-values of the KS statistics, calculated for all bins obtained in Supplementary Material S8 , in every chromosomal region for each phenotype. [file DataSheet_13.zip › SuppMat10/SuppMat10/chr17/Basal-chr17-gstart-heat.png]

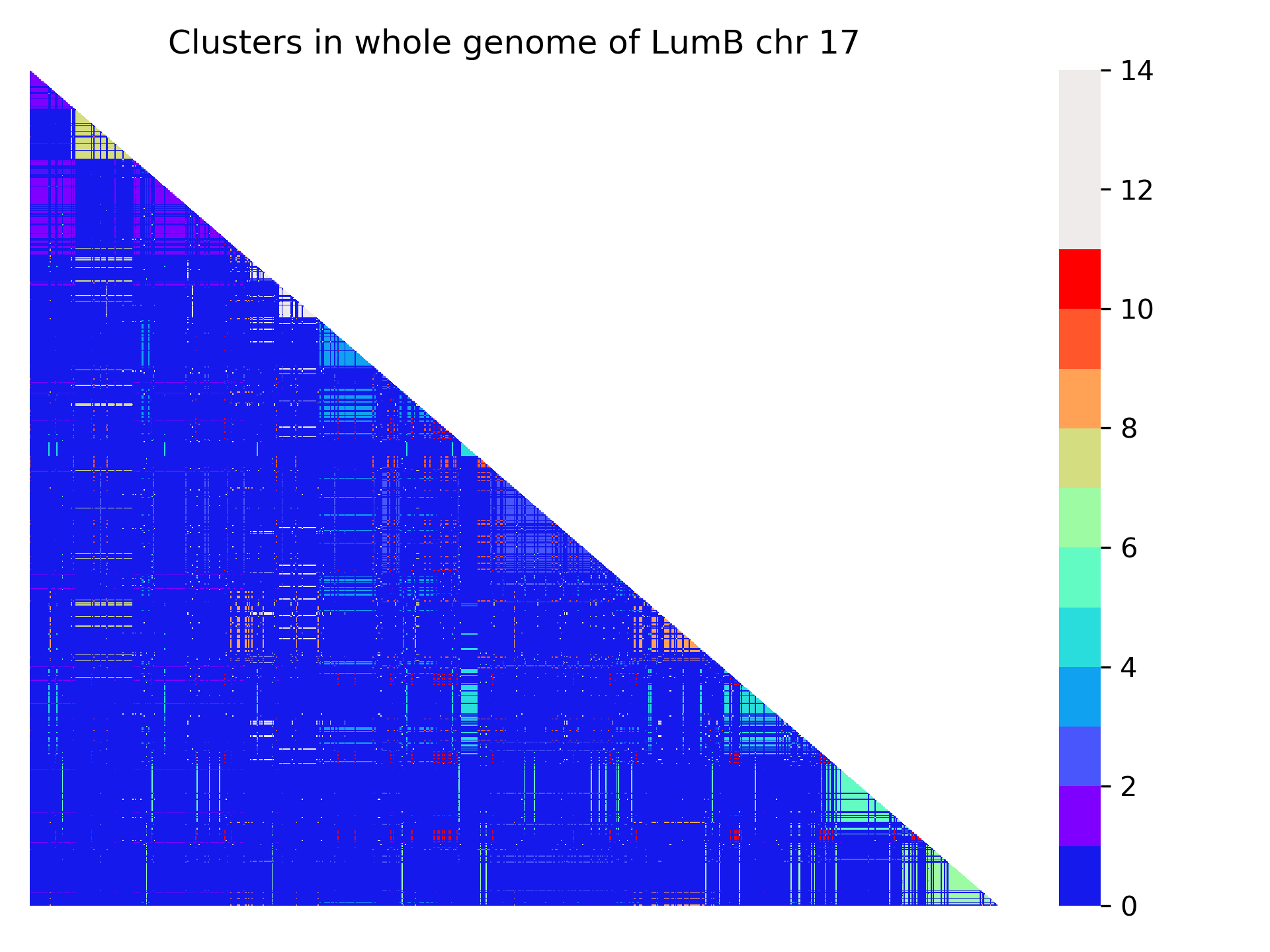

Supplement: Supplementary Material S13 — Piece-wise permutation p-values of the KS statistics, calculated for all bins obtained in Supplementary Material S8 , in every chromosomal region for each phenotype. [file DataSheet_13.zip › SuppMat10/SuppMat10/chr17/LumB-chr17-gstart-heat.png]

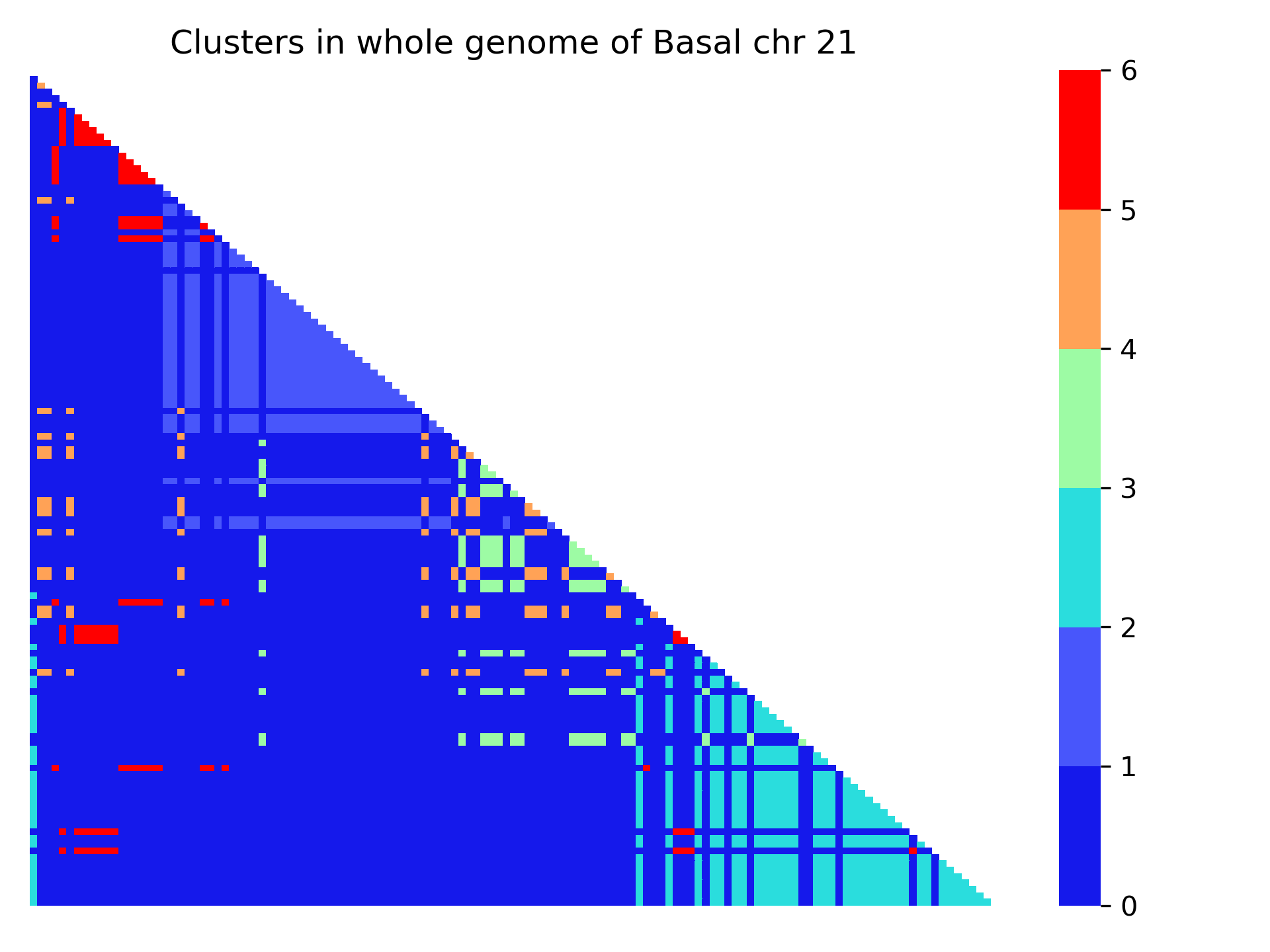

Supplement: Supplementary Material S13 — Piece-wise permutation p-values of the KS statistics, calculated for all bins obtained in Supplementary Material S8 , in every chromosomal region for each phenotype. [file DataSheet_13.zip › SuppMat10/SuppMat10/chr21/Basal-chr21-gstart-heat.png]

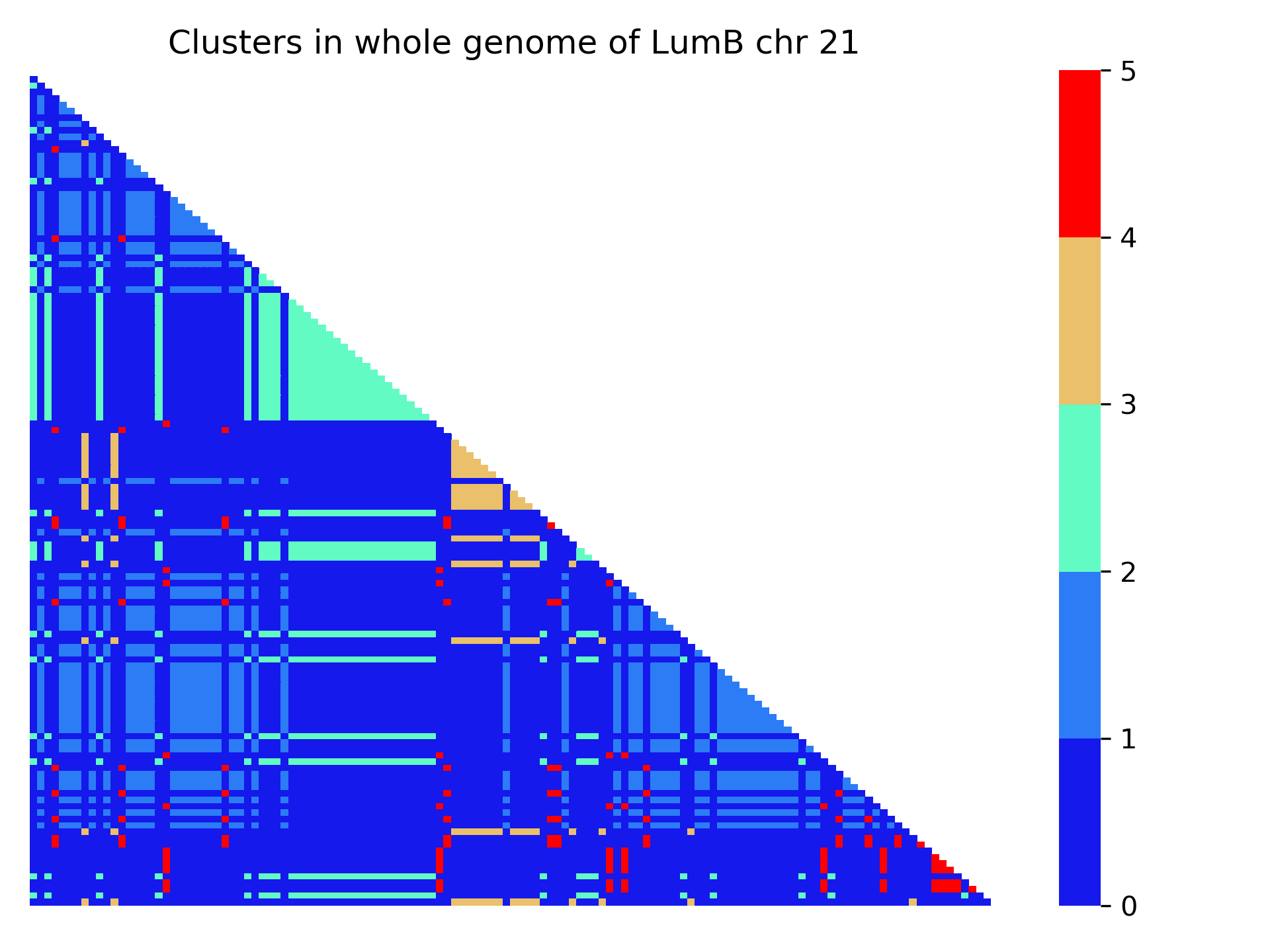

Supplement: Supplementary Material S13 — Piece-wise permutation p-values of the KS statistics, calculated for all bins obtained in Supplementary Material S8 , in every chromosomal region for each phenotype. [file DataSheet_13.zip › SuppMat10/SuppMat10/chr21/LumB-chr21-gstart-heat.png]

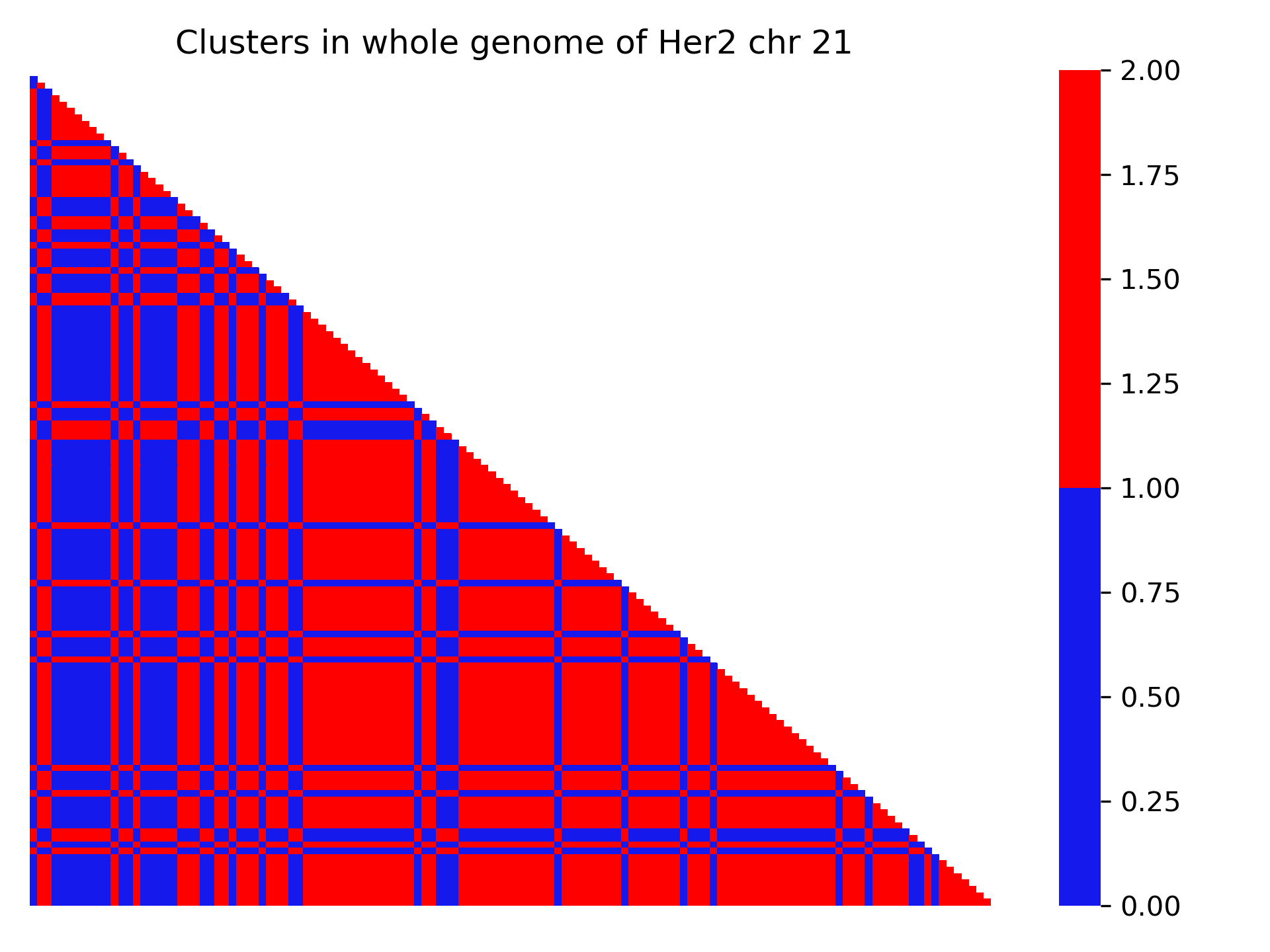

Supplement: Supplementary Material S13 — Piece-wise permutation p-values of the KS statistics, calculated for all bins obtained in Supplementary Material S8 , in every chromosomal region for each phenotype. [file DataSheet_13.zip › SuppMat10/SuppMat10/chr21/Her2-chr21-gstart-heat.png]

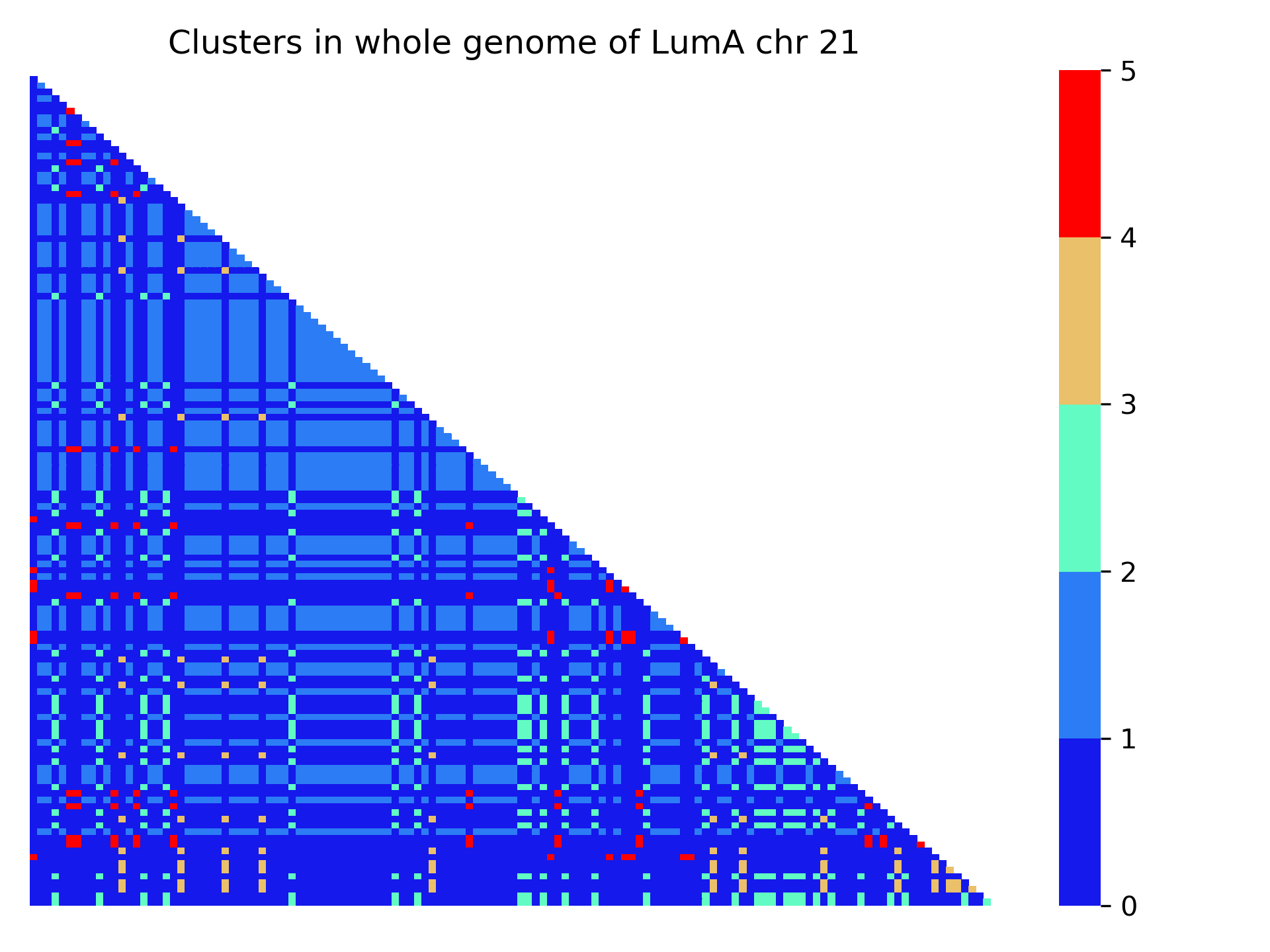

Supplement: Supplementary Material S13 — Piece-wise permutation p-values of the KS statistics, calculated for all bins obtained in Supplementary Material S8 , in every chromosomal region for each phenotype. [file DataSheet_13.zip › SuppMat10/SuppMat10/chr21/LumA-chr21-gstart-heat.png]

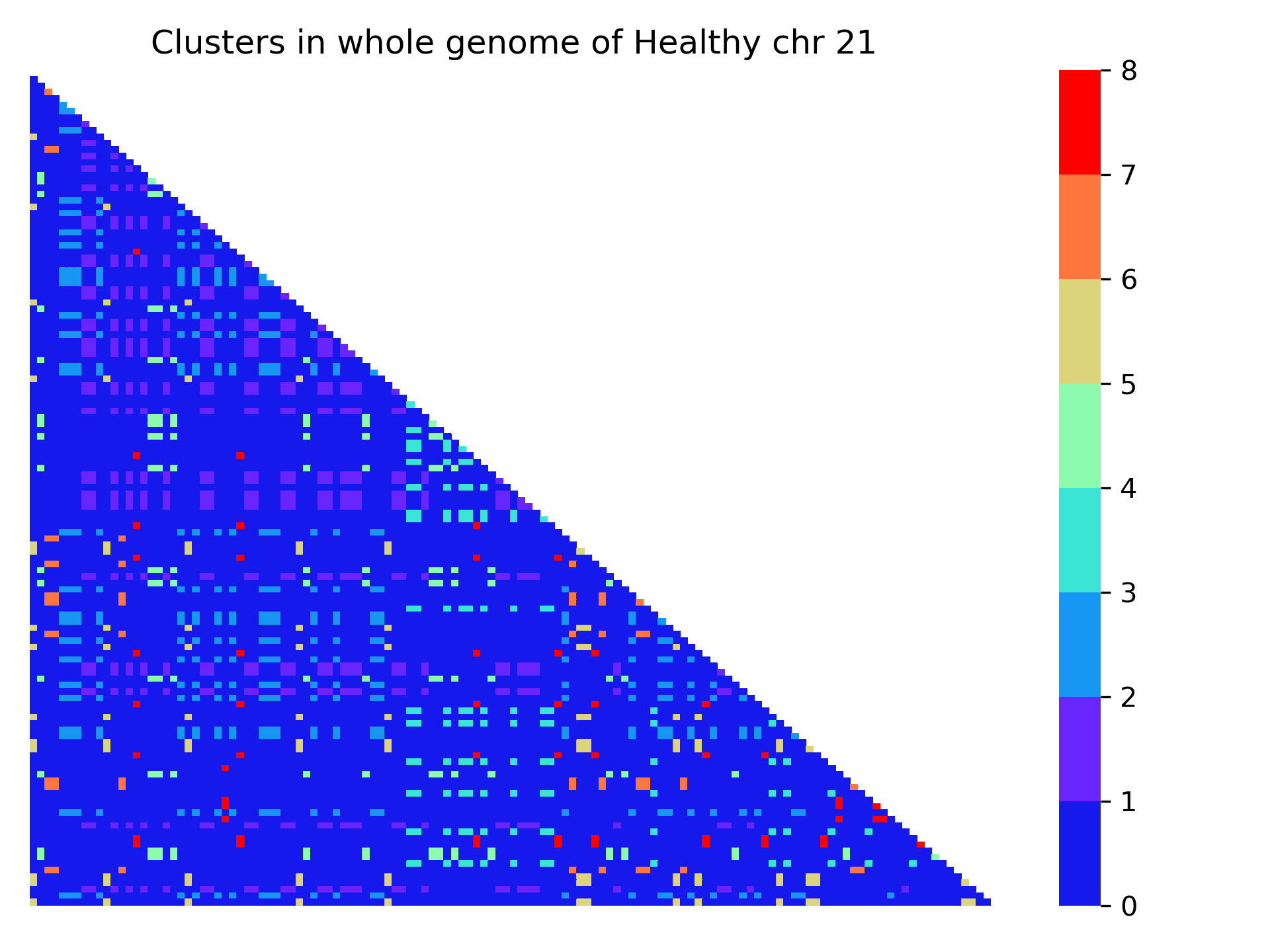

Supplement: Supplementary Material S13 — Piece-wise permutation p-values of the KS statistics, calculated for all bins obtained in Supplementary Material S8 , in every chromosomal region for each phenotype. [file DataSheet_13.zip › SuppMat10/SuppMat10/chr21/Healthy-chr21-gstart-heat.png]

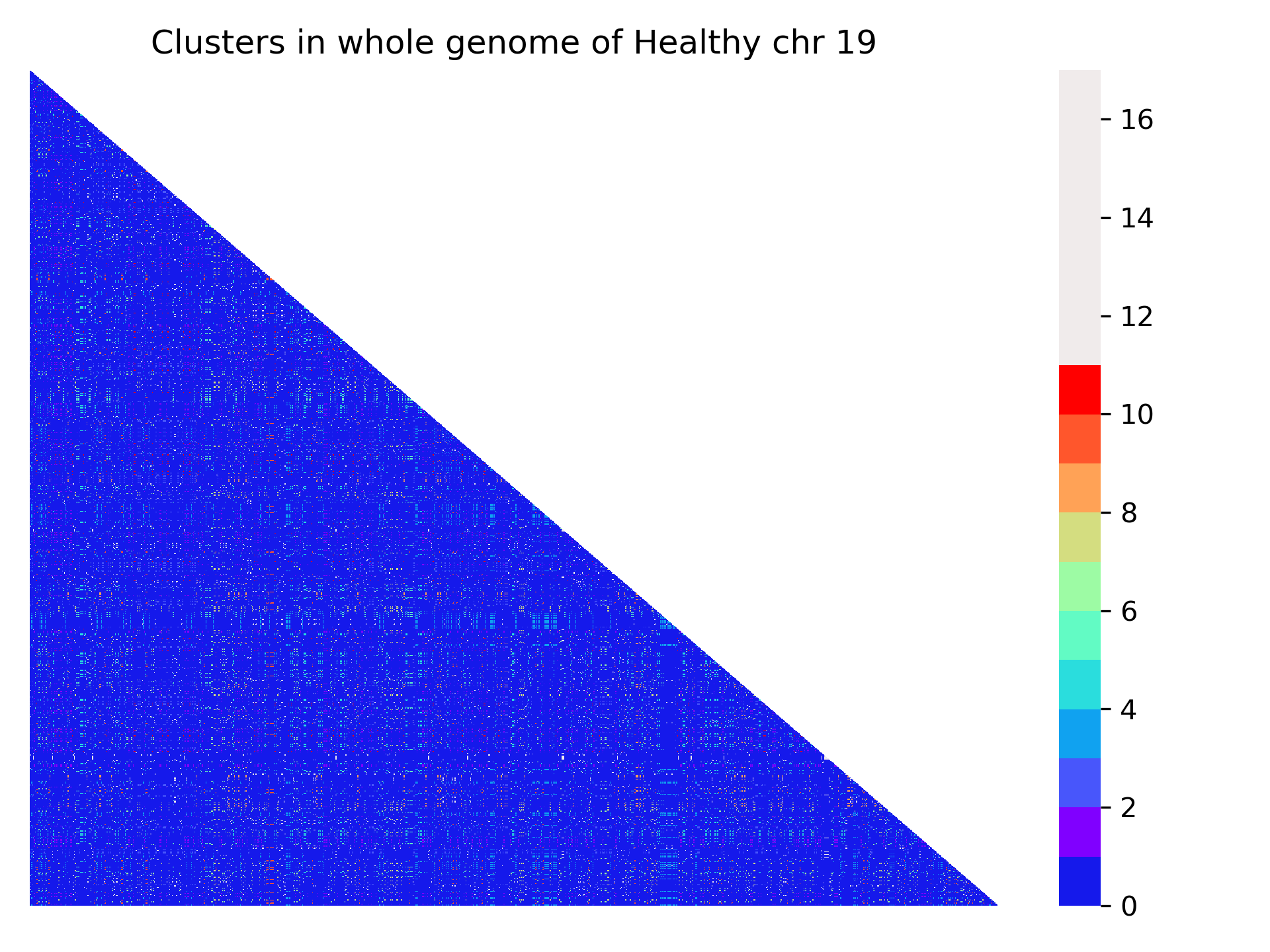

Supplement: Supplementary Material S13 — Piece-wise permutation p-values of the KS statistics, calculated for all bins obtained in Supplementary Material S8 , in every chromosomal region for each phenotype. [file DataSheet_13.zip › SuppMat10/SuppMat10/chr19/Healthy-chr19-gstart-heat.png]

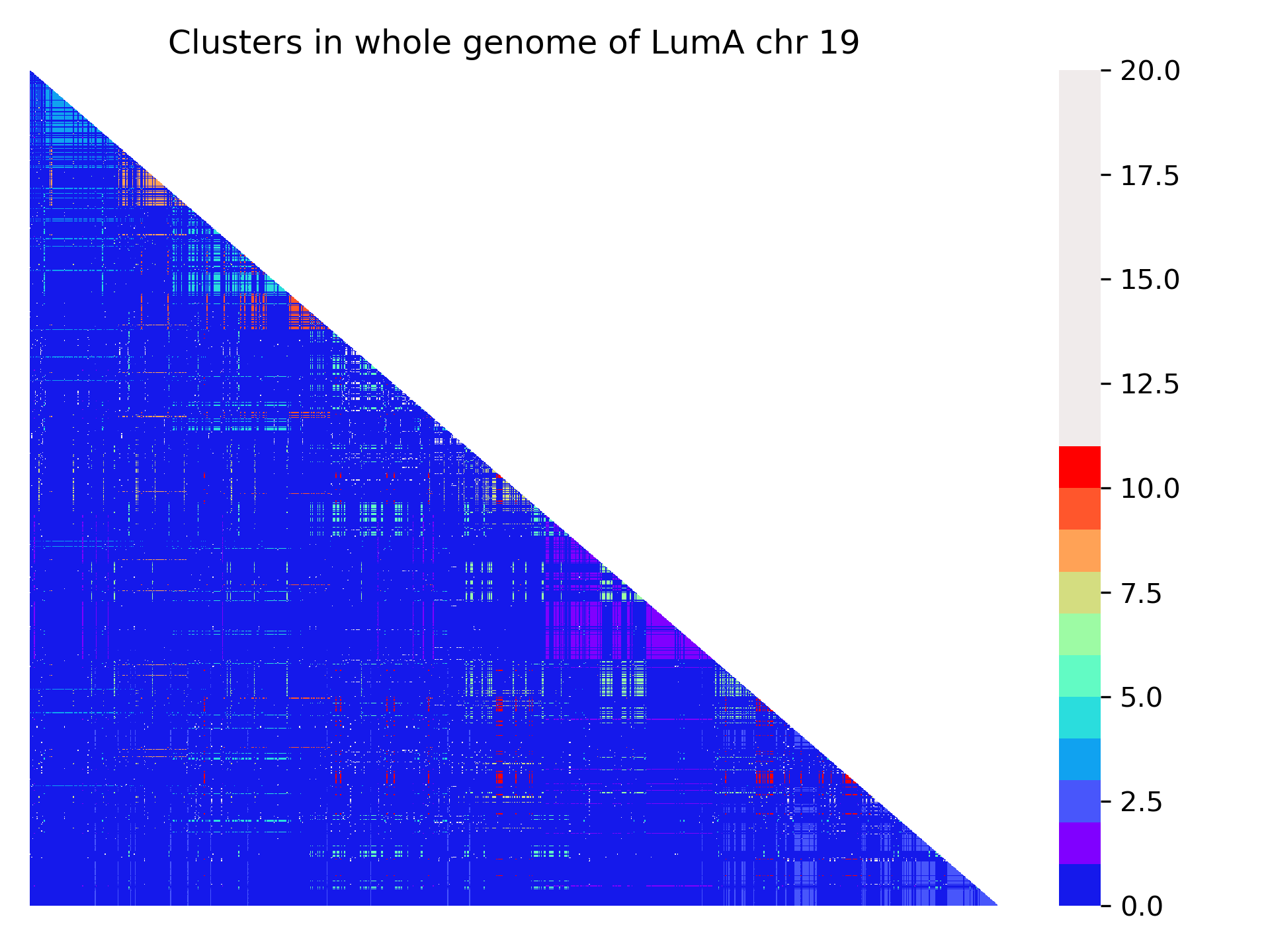

Supplement: Supplementary Material S13 — Piece-wise permutation p-values of the KS statistics, calculated for all bins obtained in Supplementary Material S8 , in every chromosomal region for each phenotype. [file DataSheet_13.zip › SuppMat10/SuppMat10/chr19/LumA-chr19-gstart-heat.png]

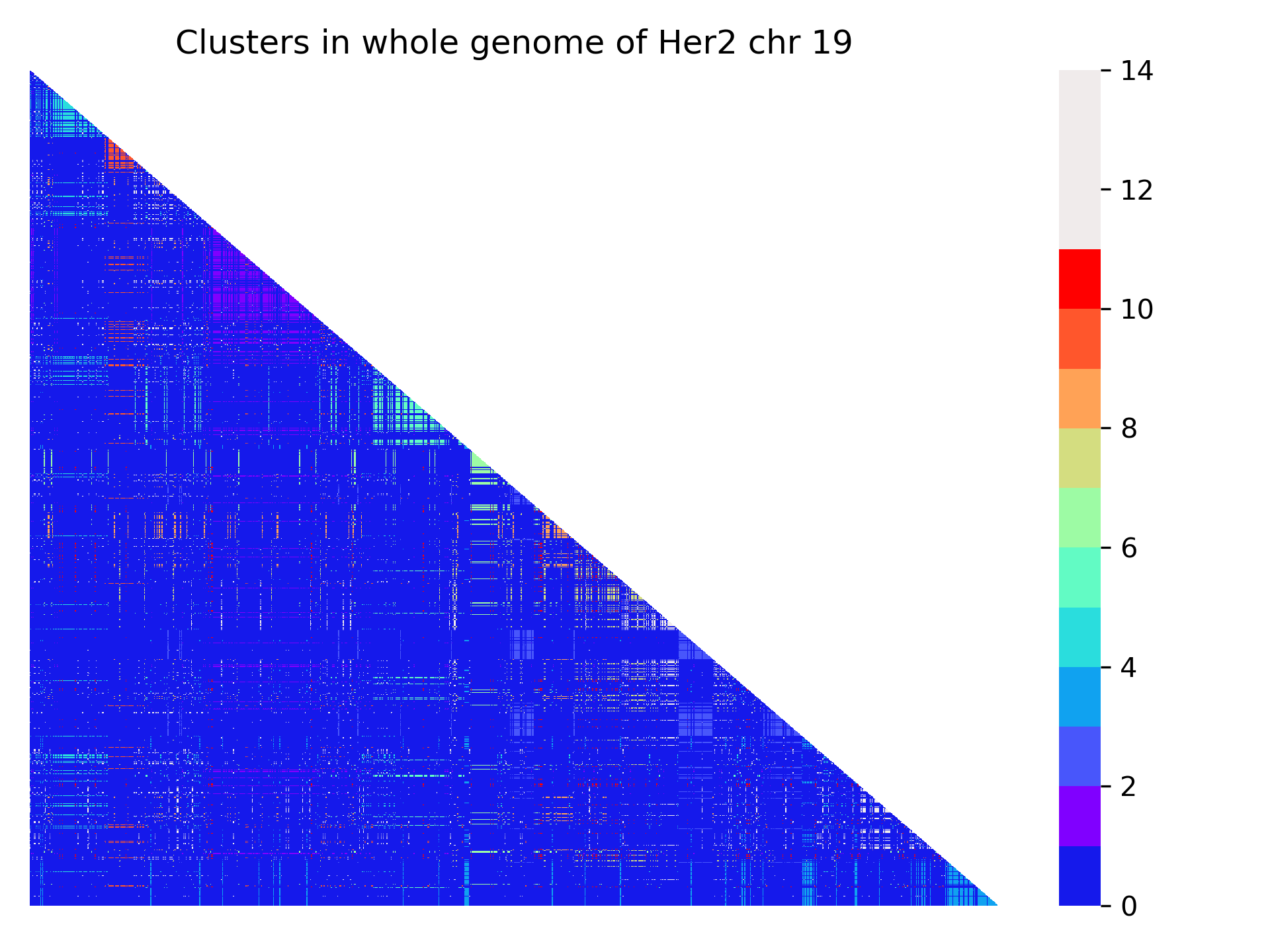

Supplement: Supplementary Material S13 — Piece-wise permutation p-values of the KS statistics, calculated for all bins obtained in Supplementary Material S8 , in every chromosomal region for each phenotype. [file DataSheet_13.zip › SuppMat10/SuppMat10/chr19/Her2-chr19-gstart-heat.png]

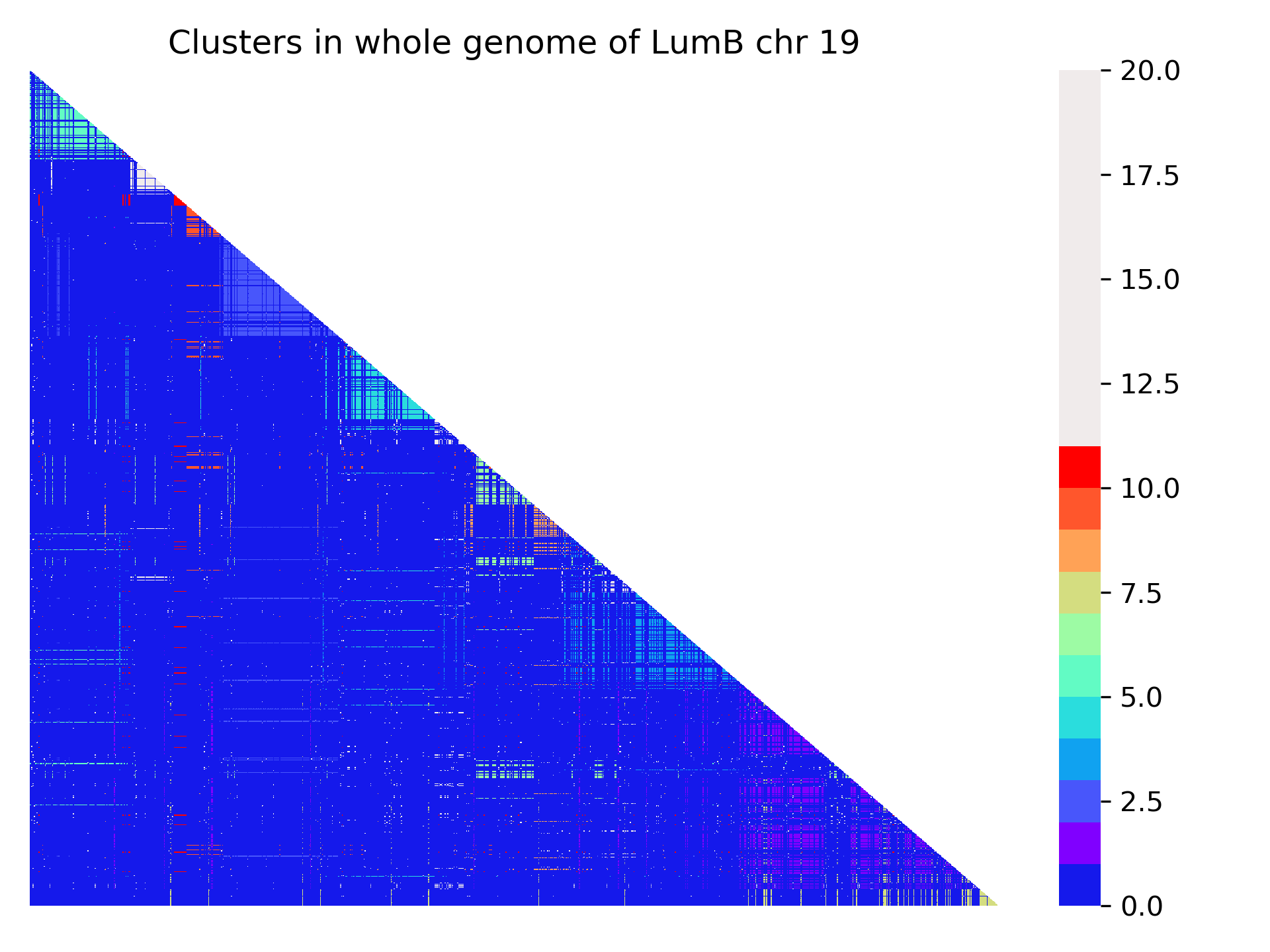

Supplement: Supplementary Material S13 — Piece-wise permutation p-values of the KS statistics, calculated for all bins obtained in Supplementary Material S8 , in every chromosomal region for each phenotype. [file DataSheet_13.zip › SuppMat10/SuppMat10/chr19/LumB-chr19-gstart-heat.png]

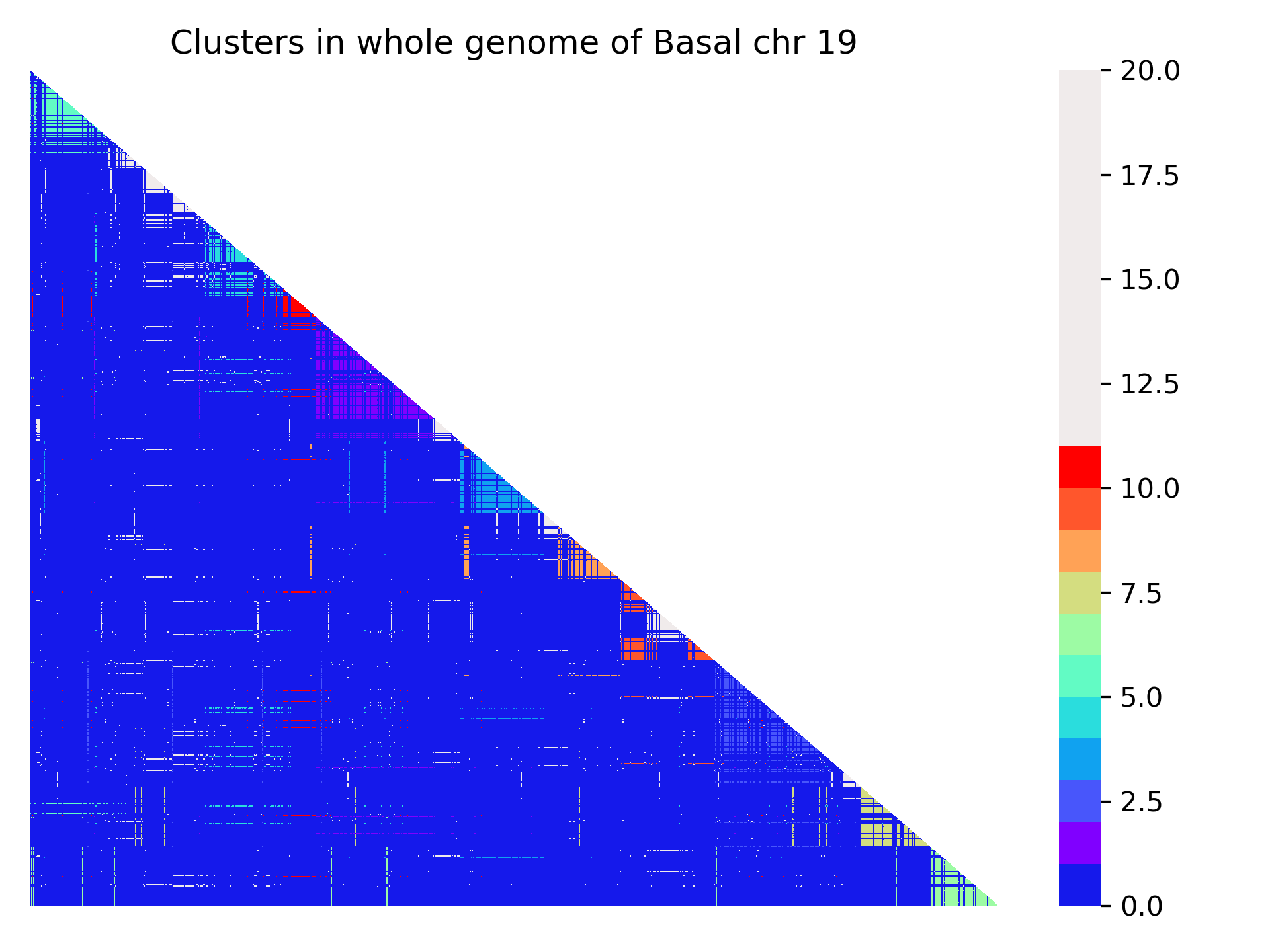

Supplement: Supplementary Material S13 — Piece-wise permutation p-values of the KS statistics, calculated for all bins obtained in Supplementary Material S8 , in every chromosomal region for each phenotype. [file DataSheet_13.zip › SuppMat10/SuppMat10/chr19/Basal-chr19-gstart-heat.png]

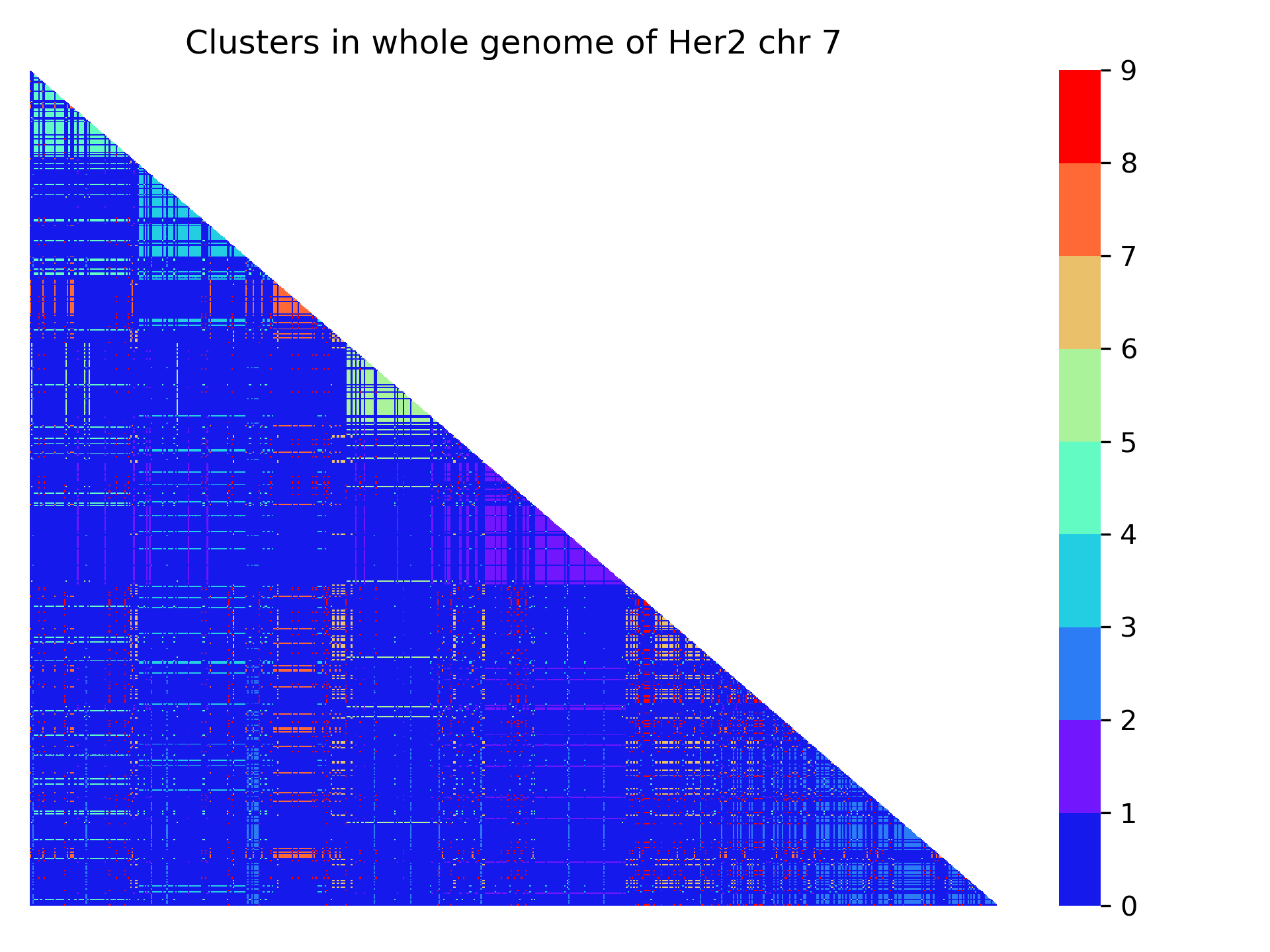

Supplement: Supplementary Material S13 — Piece-wise permutation p-values of the KS statistics, calculated for all bins obtained in Supplementary Material S8 , in every chromosomal region for each phenotype. [file DataSheet_13.zip › SuppMat10/SuppMat10/chr7/Her2-chr7-gstart-heat.png]

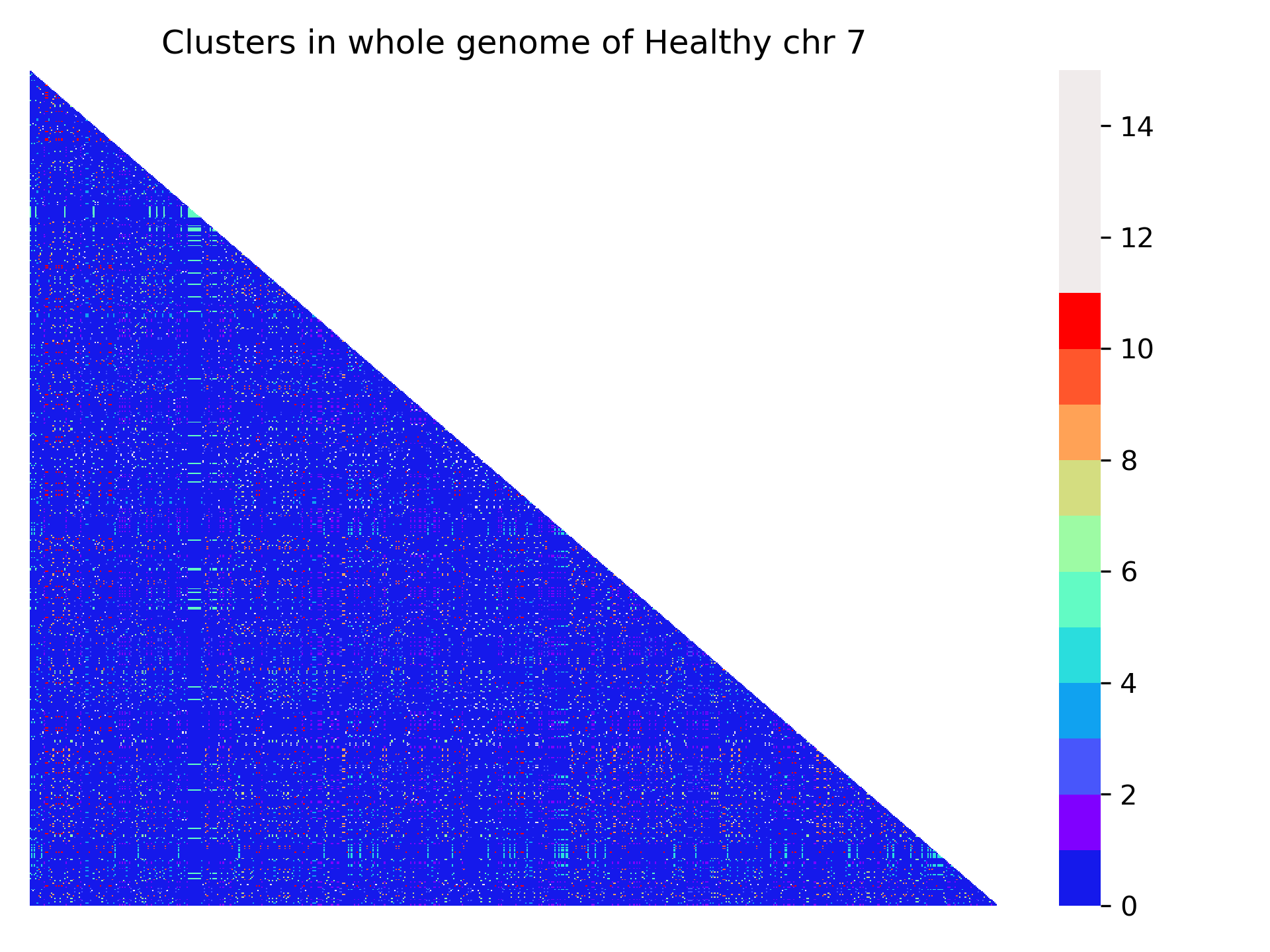

Supplement: Supplementary Material S13 — Piece-wise permutation p-values of the KS statistics, calculated for all bins obtained in Supplementary Material S8 , in every chromosomal region for each phenotype. [file DataSheet_13.zip › SuppMat10/SuppMat10/chr7/Healthy-chr7-gstart-heat.png]

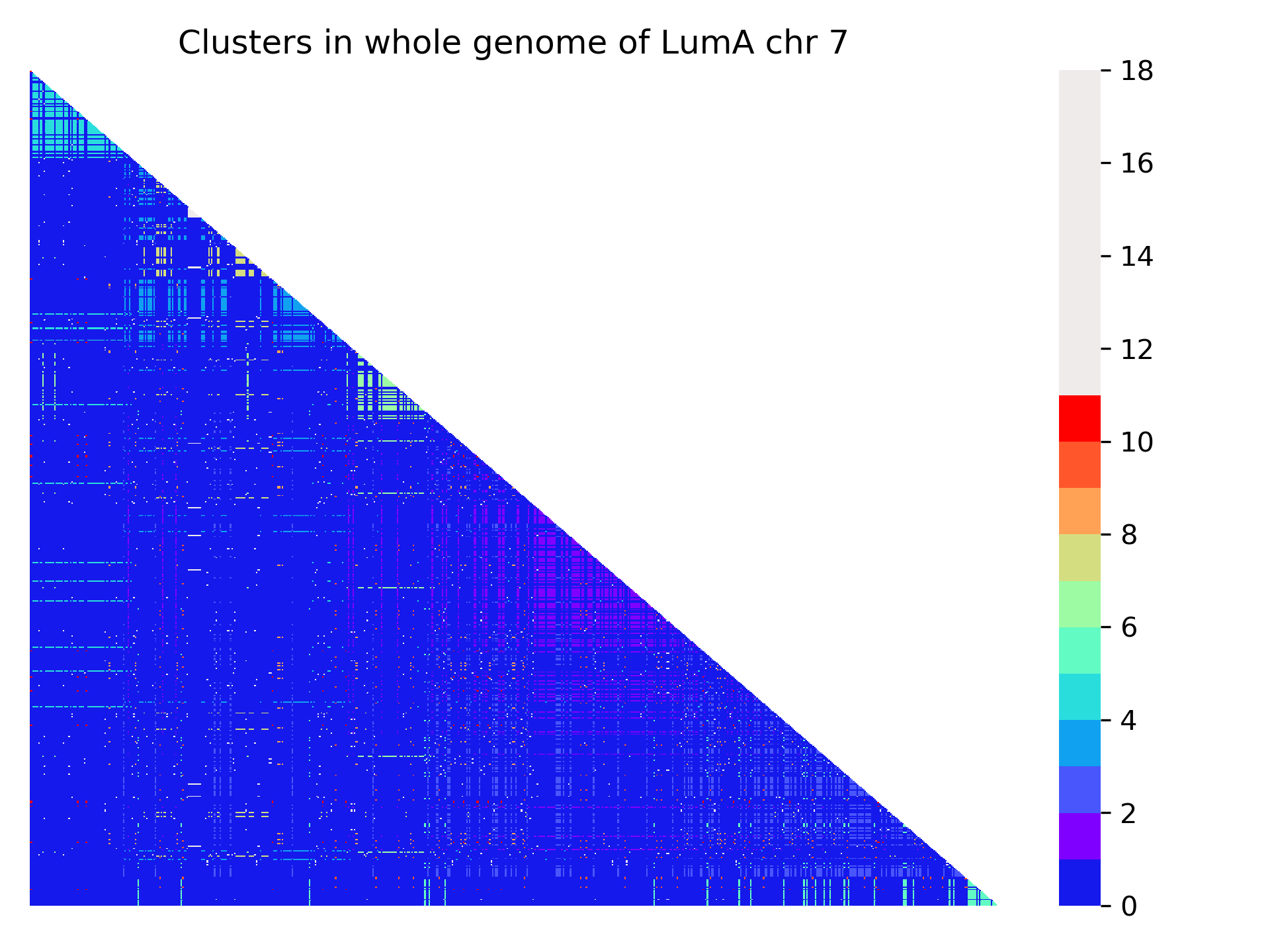

Supplement: Supplementary Material S13 — Piece-wise permutation p-values of the KS statistics, calculated for all bins obtained in Supplementary Material S8 , in every chromosomal region for each phenotype. [file DataSheet_13.zip › SuppMat10/SuppMat10/chr7/LumA-chr7-gstart-heat.png]

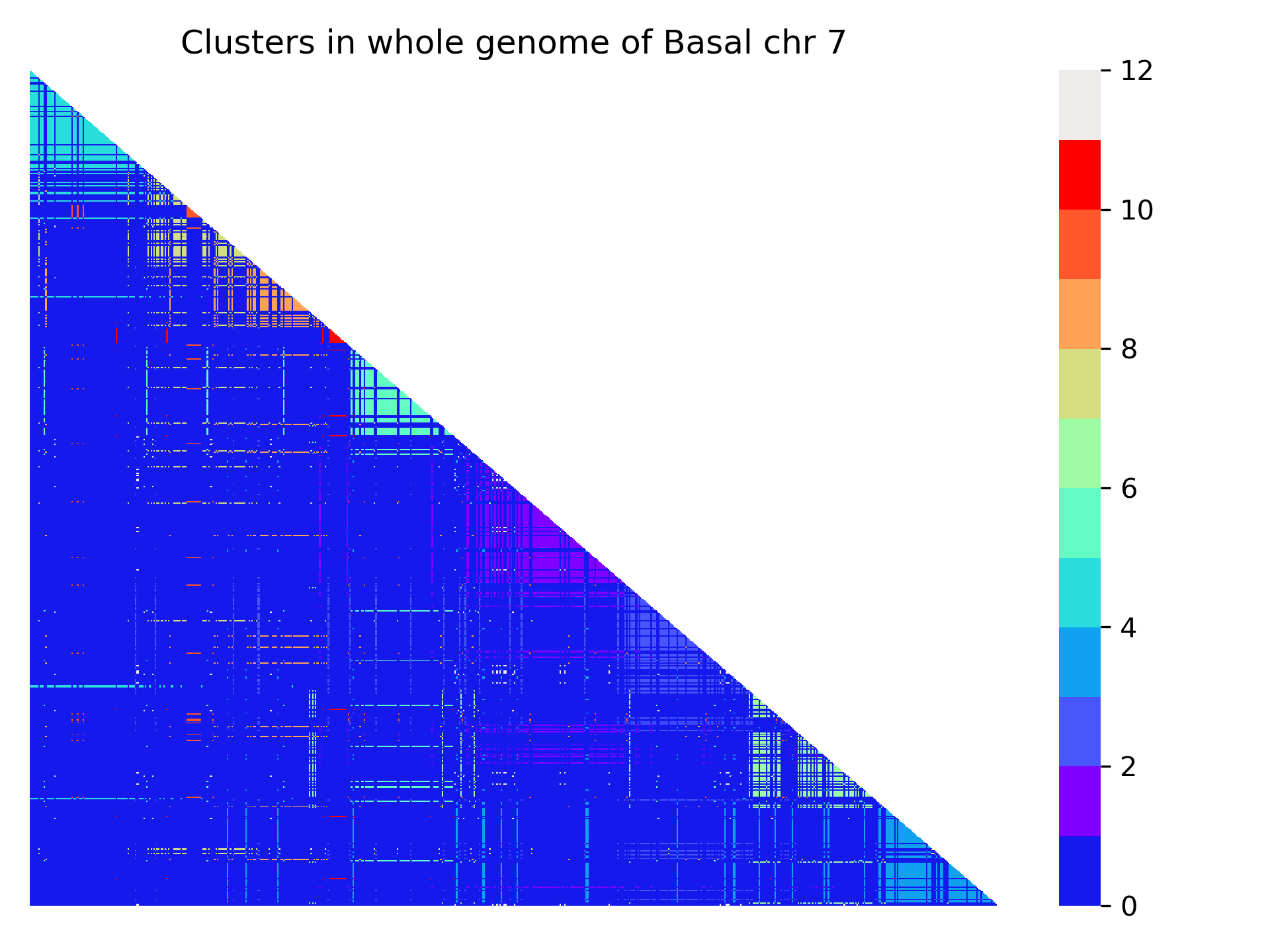

Supplement: Supplementary Material S13 — Piece-wise permutation p-values of the KS statistics, calculated for all bins obtained in Supplementary Material S8 , in every chromosomal region for each phenotype. [file DataSheet_13.zip › SuppMat10/SuppMat10/chr7/Basal-chr7-gstart-heat.png]

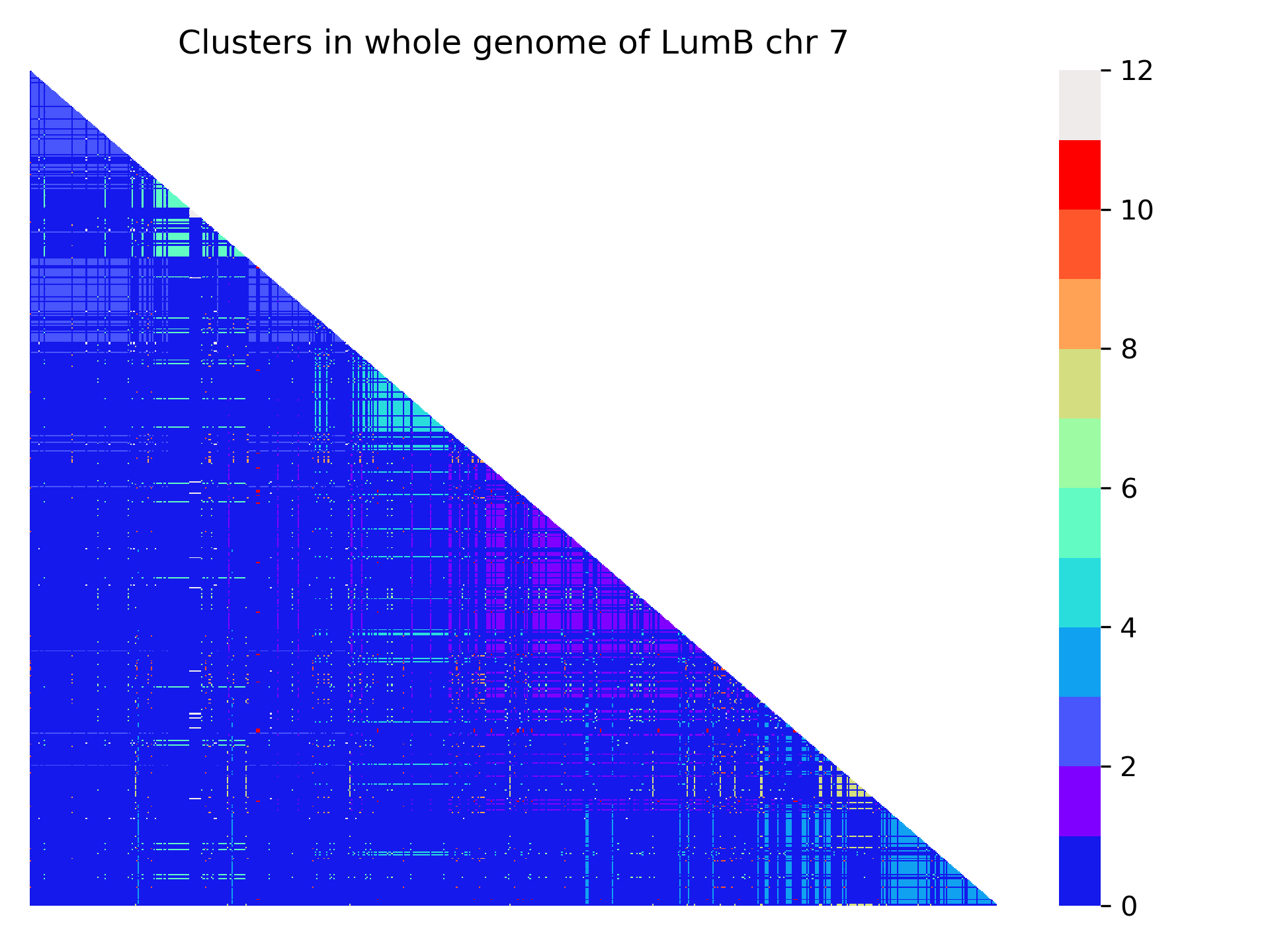

Supplement: Supplementary Material S13 — Piece-wise permutation p-values of the KS statistics, calculated for all bins obtained in Supplementary Material S8 , in every chromosomal region for each phenotype. [file DataSheet_13.zip › SuppMat10/SuppMat10/chr7/LumB-chr7-gstart-heat.png]

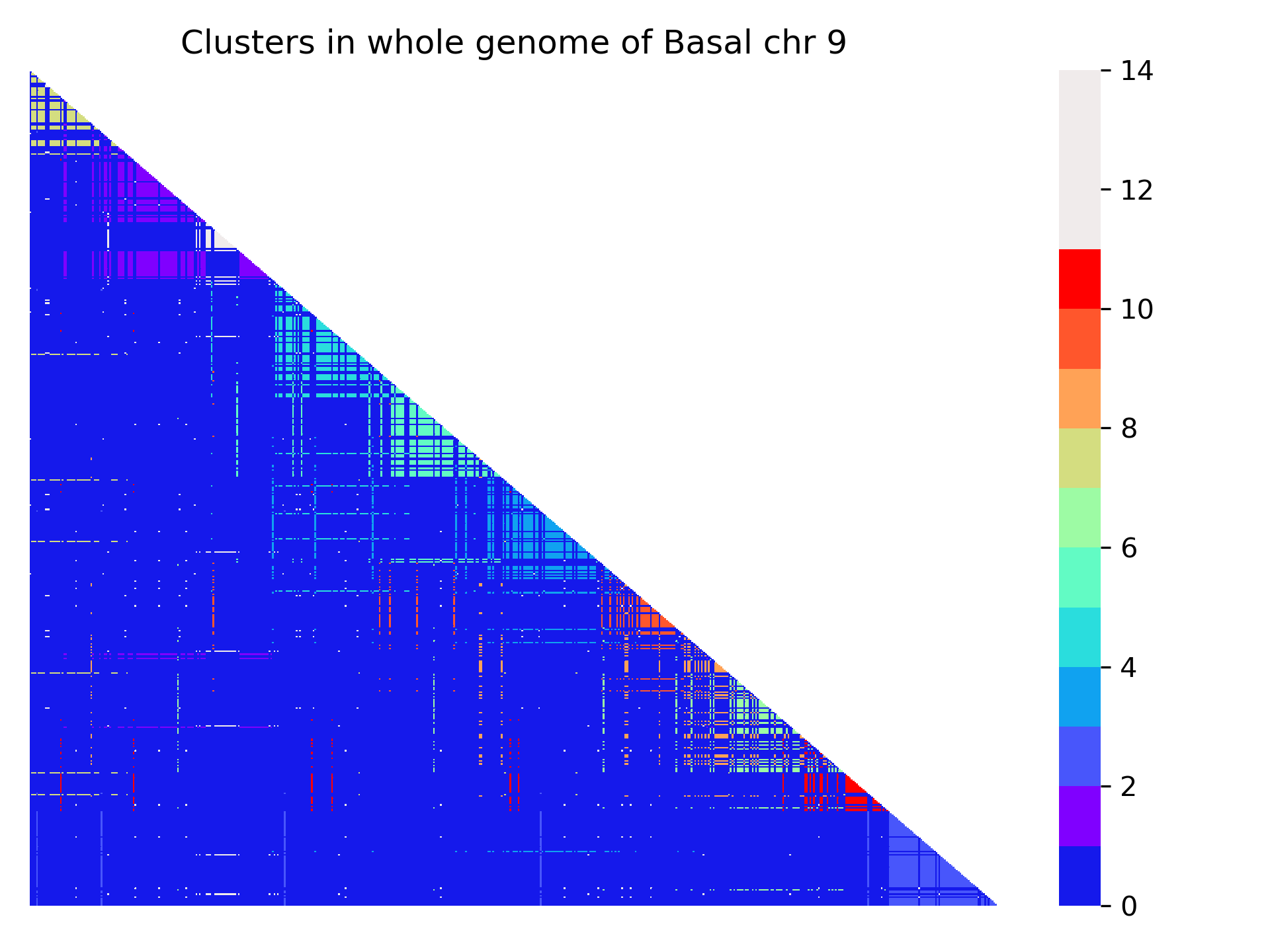

Supplement: Supplementary Material S13 — Piece-wise permutation p-values of the KS statistics, calculated for all bins obtained in Supplementary Material S8 , in every chromosomal region for each phenotype. [file DataSheet_13.zip › SuppMat10/SuppMat10/chr9/Basal-chr9-gstart-heat.png]

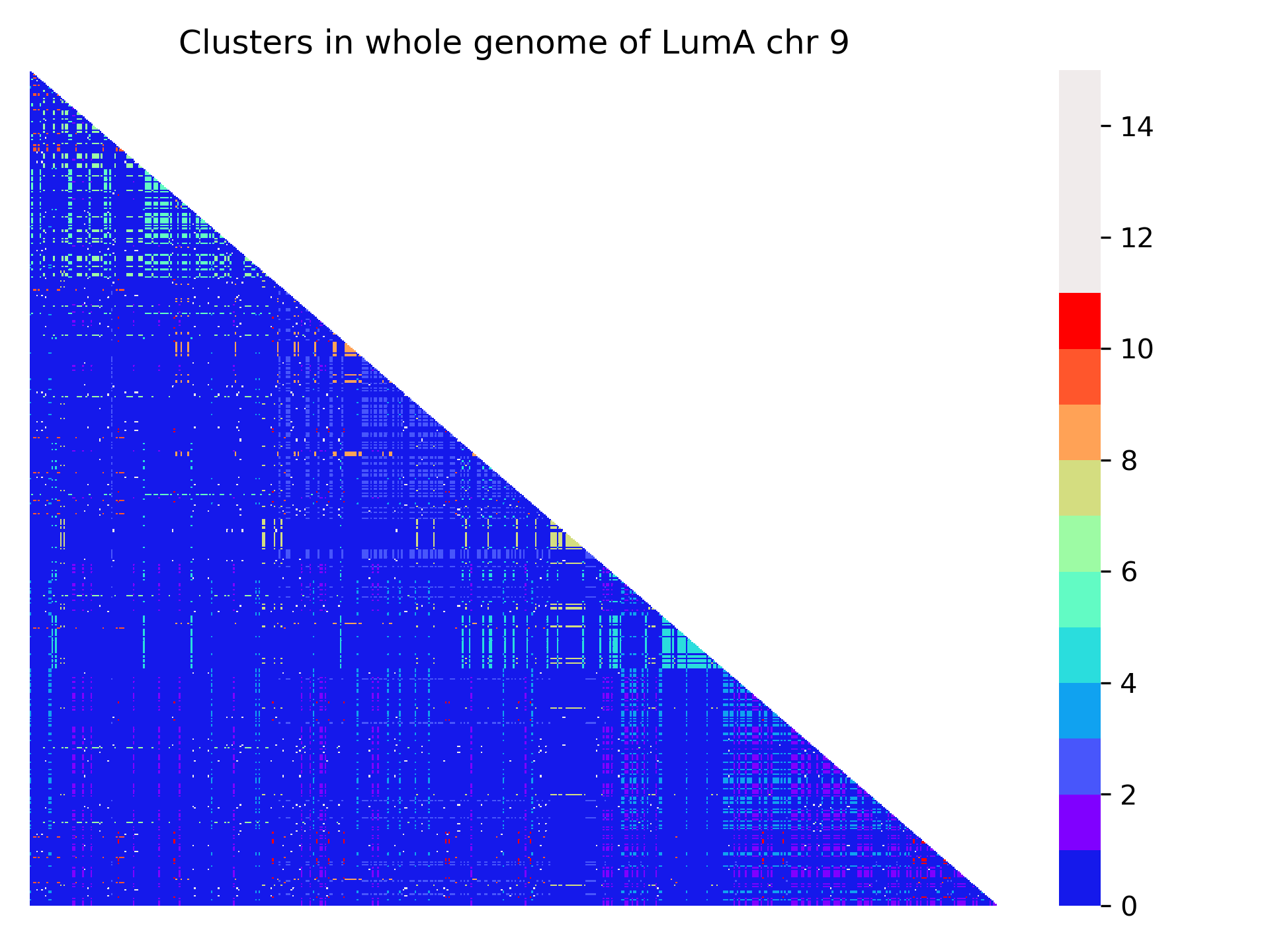

Supplement: Supplementary Material S13 — Piece-wise permutation p-values of the KS statistics, calculated for all bins obtained in Supplementary Material S8 , in every chromosomal region for each phenotype. [file DataSheet_13.zip › SuppMat10/SuppMat10/chr9/LumA-chr9-gstart-heat.png]

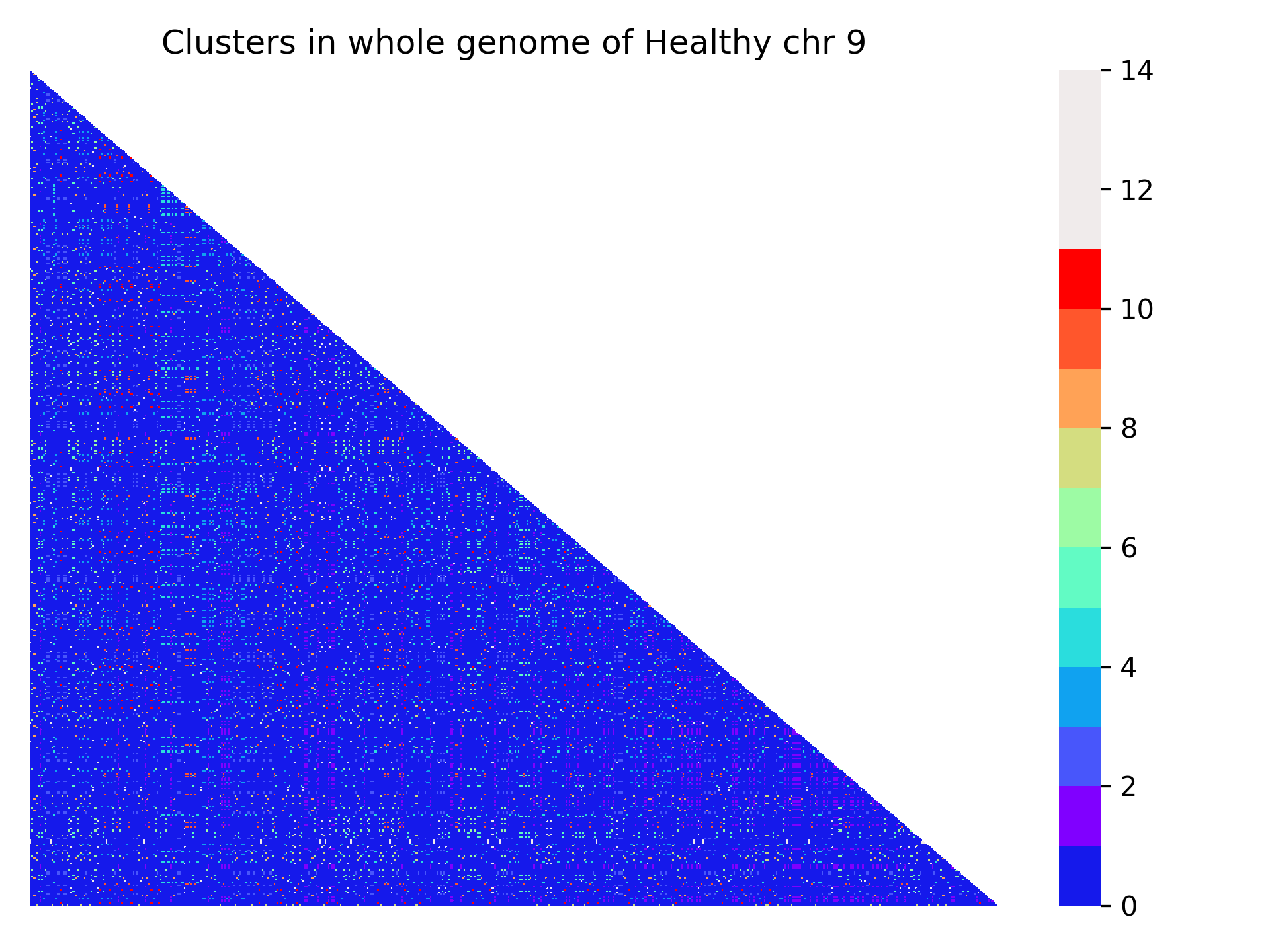

Supplement: Supplementary Material S13 — Piece-wise permutation p-values of the KS statistics, calculated for all bins obtained in Supplementary Material S8 , in every chromosomal region for each phenotype. [file DataSheet_13.zip › SuppMat10/SuppMat10/chr9/Healthy-chr9-gstart-heat.png]

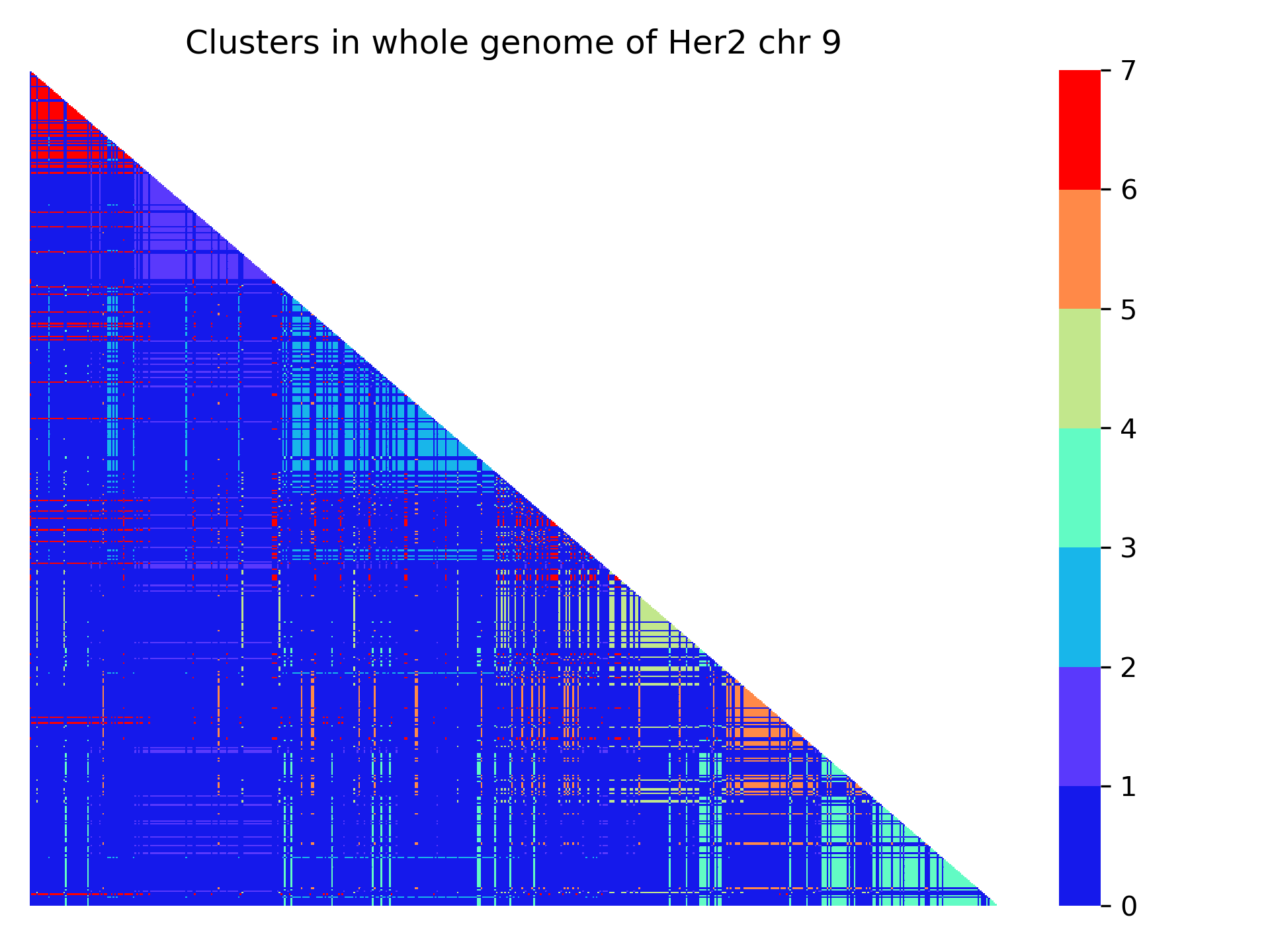

Supplement: Supplementary Material S13 — Piece-wise permutation p-values of the KS statistics, calculated for all bins obtained in Supplementary Material S8 , in every chromosomal region for each phenotype. [file DataSheet_13.zip › SuppMat10/SuppMat10/chr9/Her2-chr9-gstart-heat.png]

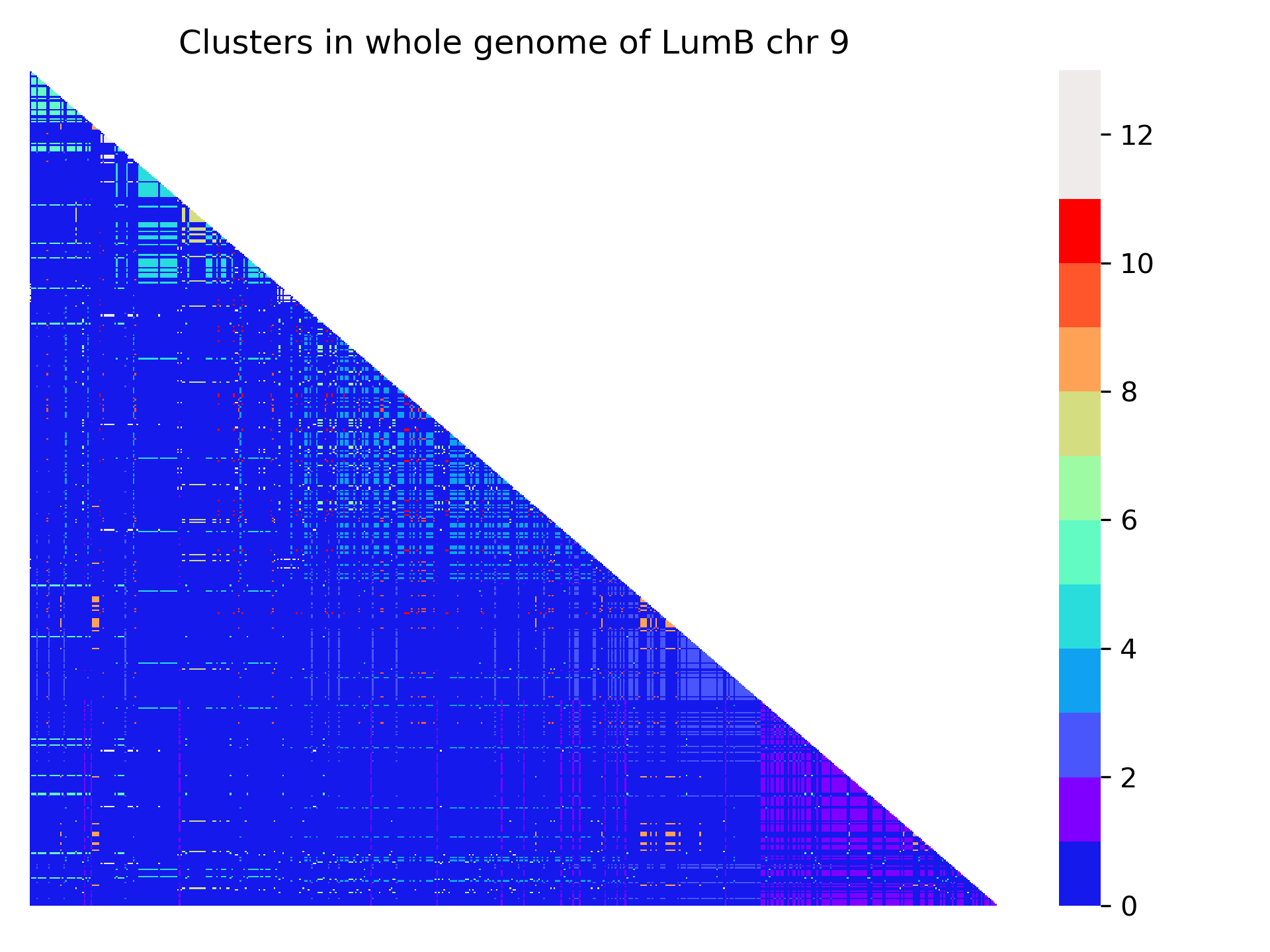

Supplement: Supplementary Material S13 — Piece-wise permutation p-values of the KS statistics, calculated for all bins obtained in Supplementary Material S8 , in every chromosomal region for each phenotype. [file DataSheet_13.zip › SuppMat10/SuppMat10/chr9/LumB-chr9-gstart-heat.png]

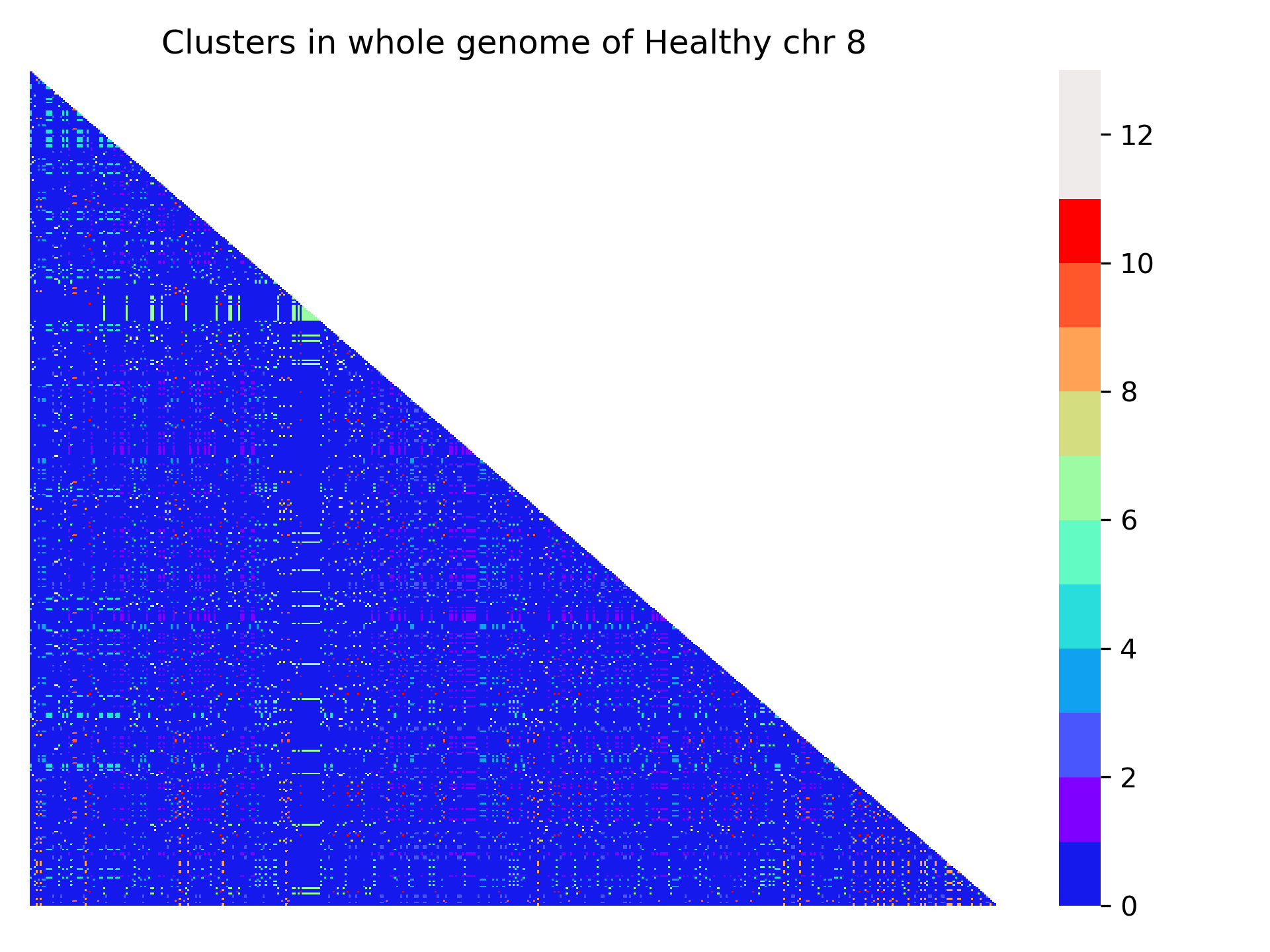

Supplement: Supplementary Material S13 — Piece-wise permutation p-values of the KS statistics, calculated for all bins obtained in Supplementary Material S8 , in every chromosomal region for each phenotype. [file DataSheet_13.zip › SuppMat10/SuppMat10/chr8/Healthy-chr8-gstart-heat.png]

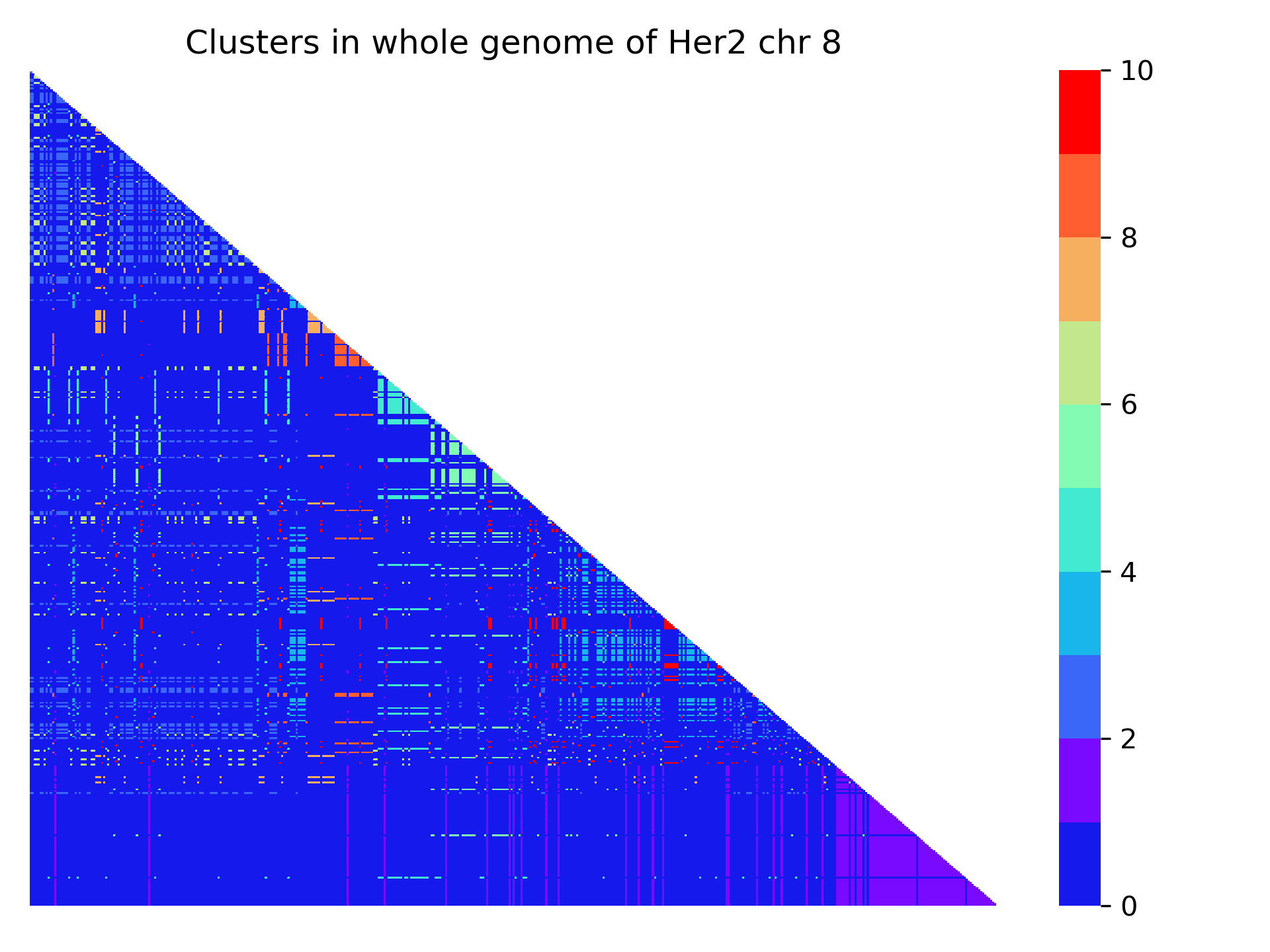

Supplement: Supplementary Material S13 — Piece-wise permutation p-values of the KS statistics, calculated for all bins obtained in Supplementary Material S8 , in every chromosomal region for each phenotype. [file DataSheet_13.zip › SuppMat10/SuppMat10/chr8/Her2-chr8-gstart-heat.png]

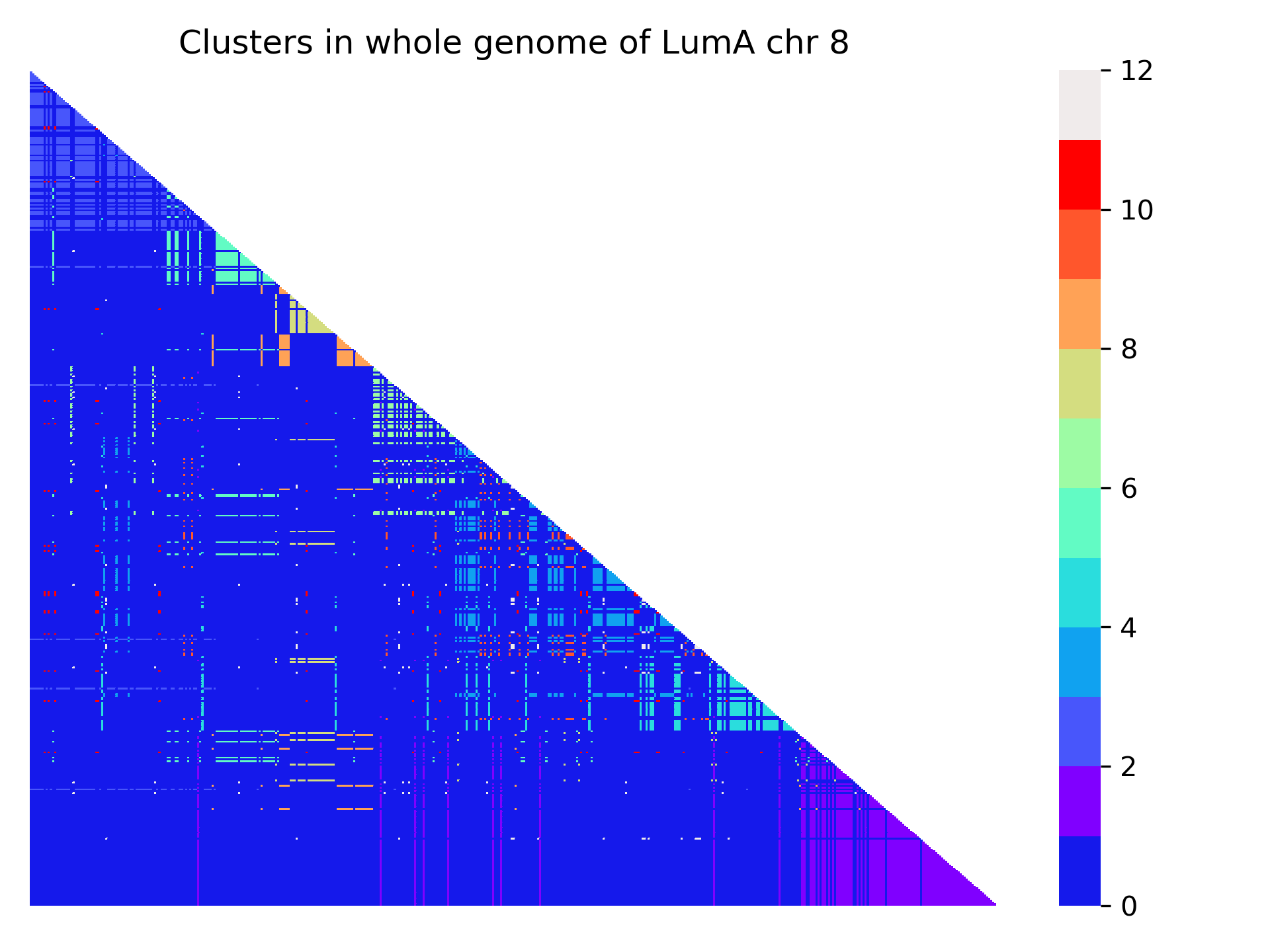

Supplement: Supplementary Material S13 — Piece-wise permutation p-values of the KS statistics, calculated for all bins obtained in Supplementary Material S8 , in every chromosomal region for each phenotype. [file DataSheet_13.zip › SuppMat10/SuppMat10/chr8/LumA-chr8-gstart-heat.png]

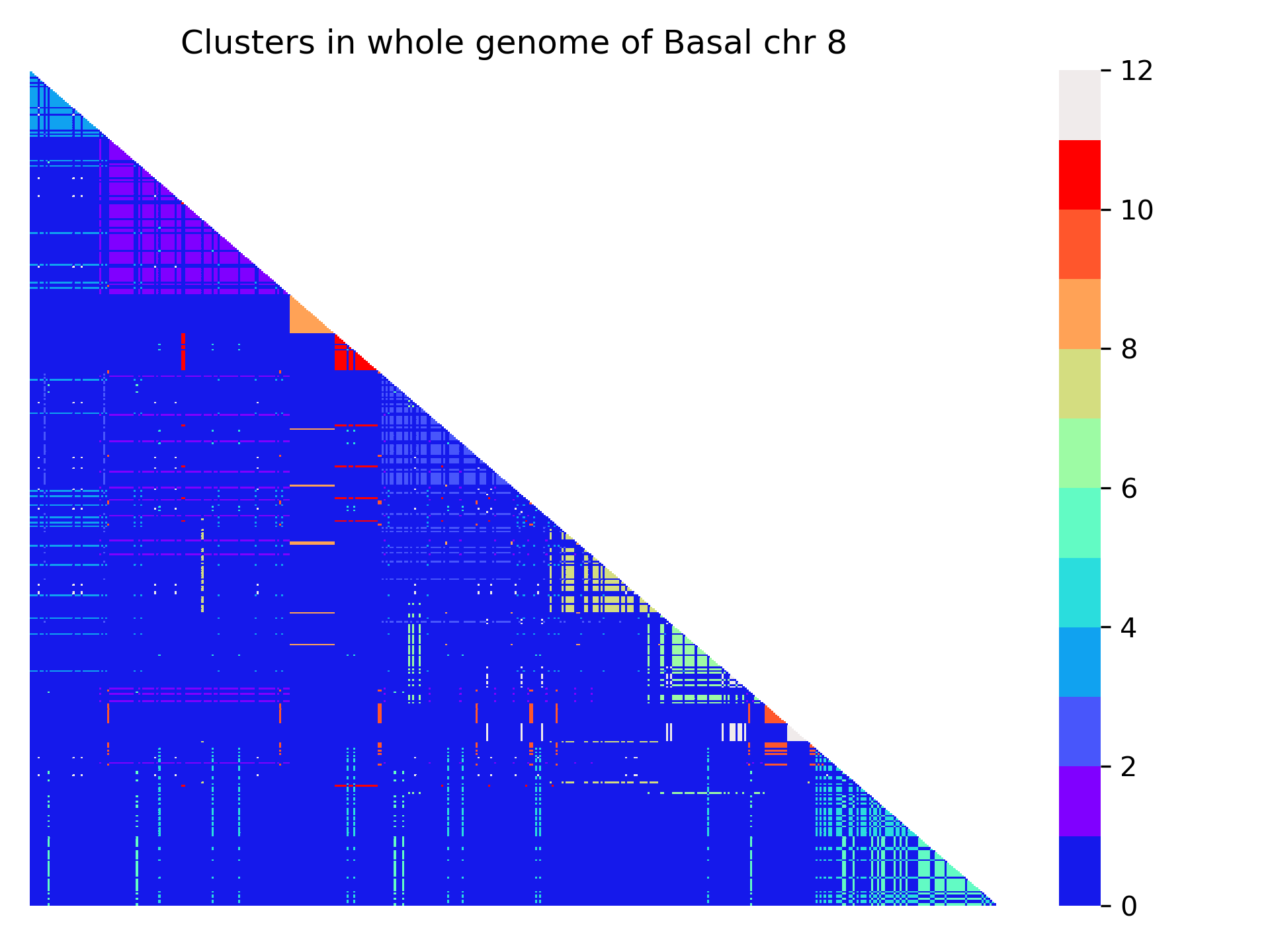

Supplement: Supplementary Material S13 — Piece-wise permutation p-values of the KS statistics, calculated for all bins obtained in Supplementary Material S8 , in every chromosomal region for each phenotype. [file DataSheet_13.zip › SuppMat10/SuppMat10/chr8/Basal-chr8-gstart-heat.png]

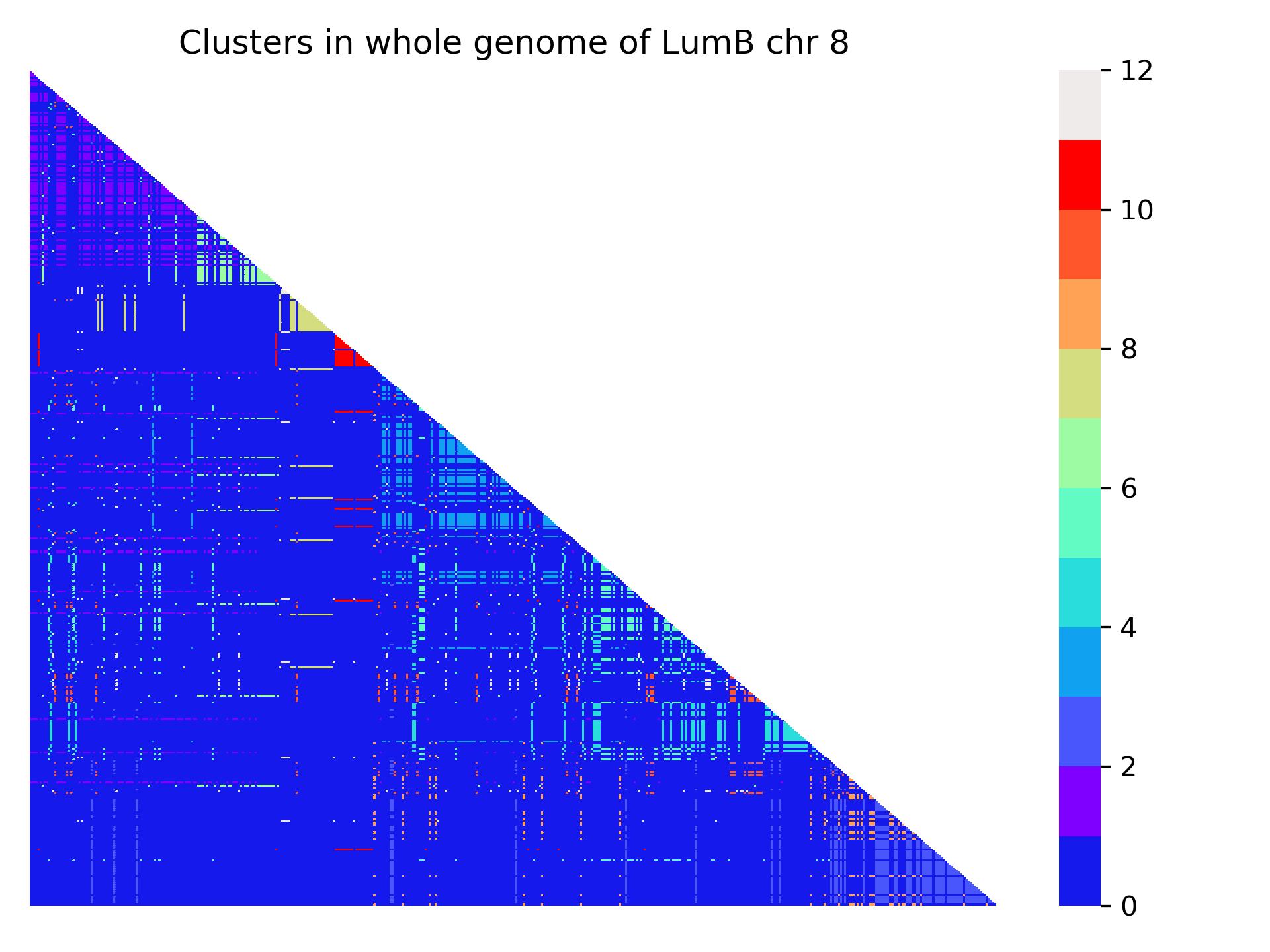

Supplement: Supplementary Material S13 — Piece-wise permutation p-values of the KS statistics, calculated for all bins obtained in Supplementary Material S8 , in every chromosomal region for each phenotype. [file DataSheet_13.zip › SuppMat10/SuppMat10/chr8/LumB-chr8-gstart-heat.png]

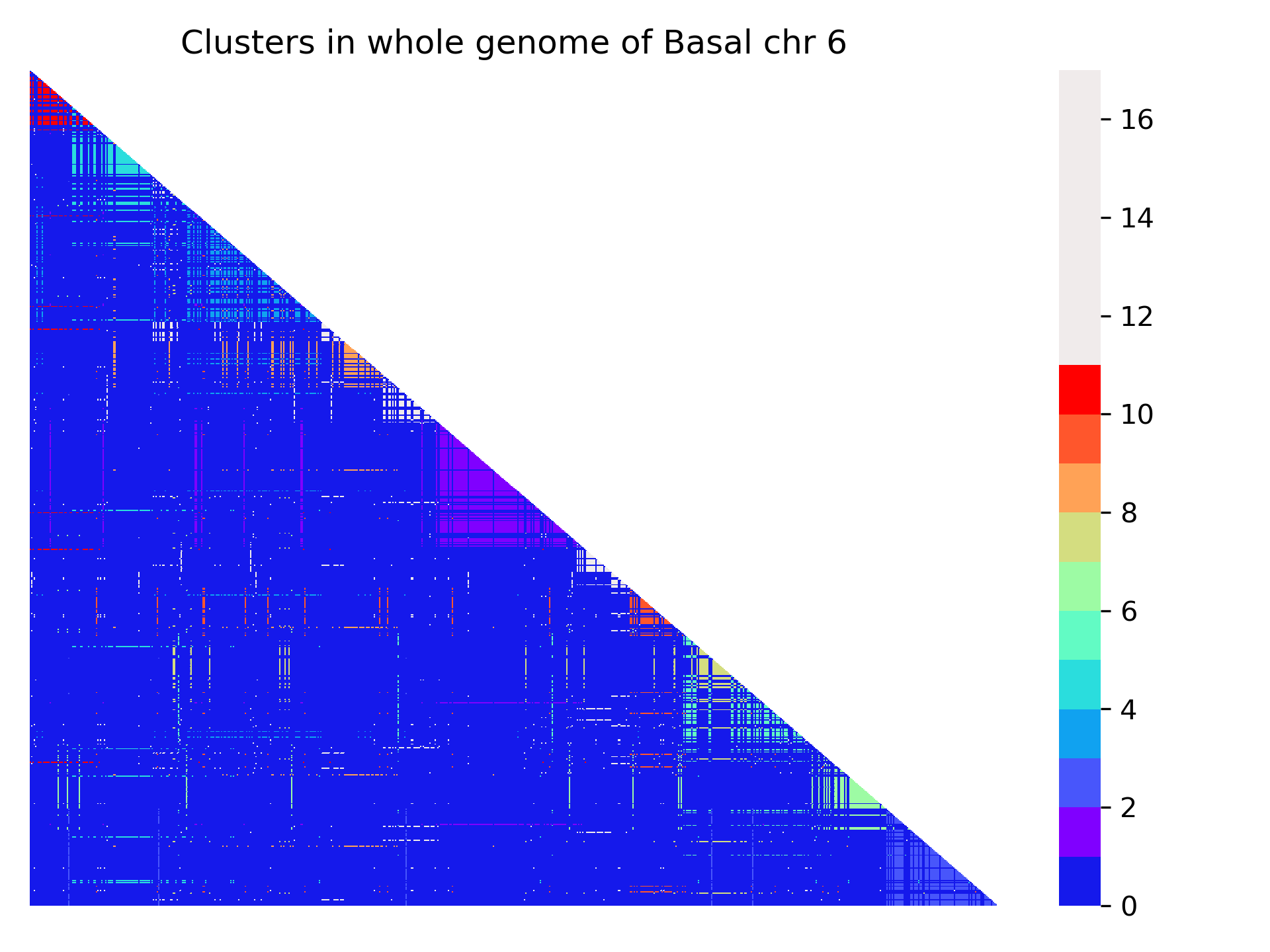

Supplement: Supplementary Material S13 — Piece-wise permutation p-values of the KS statistics, calculated for all bins obtained in Supplementary Material S8 , in every chromosomal region for each phenotype. [file DataSheet_13.zip › SuppMat10/SuppMat10/chr6/Basal-chr6-gstart-heat.png]

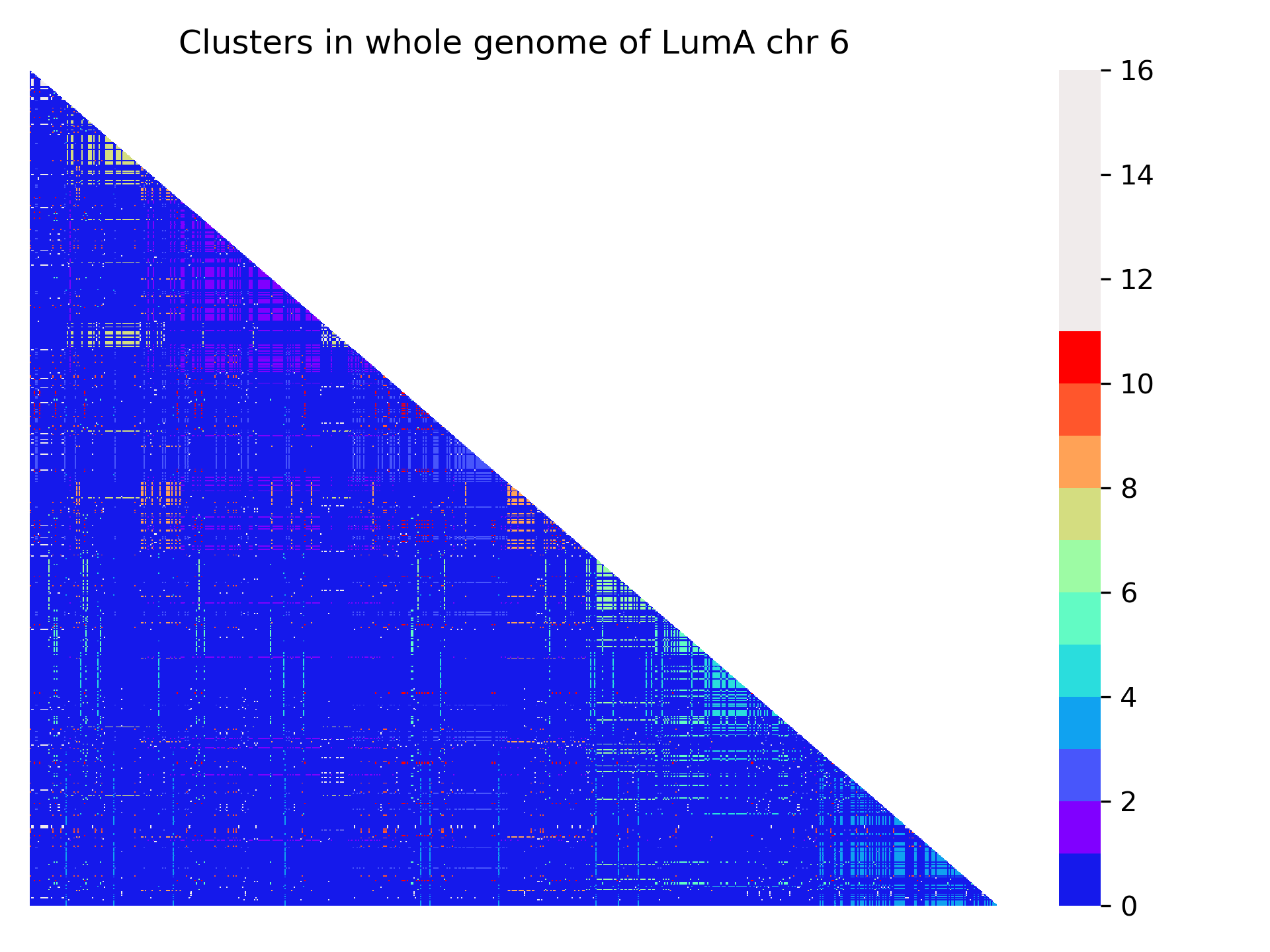

Supplement: Supplementary Material S13 — Piece-wise permutation p-values of the KS statistics, calculated for all bins obtained in Supplementary Material S8 , in every chromosomal region for each phenotype. [file DataSheet_13.zip › SuppMat10/SuppMat10/chr6/LumA-chr6-gstart-heat.png]

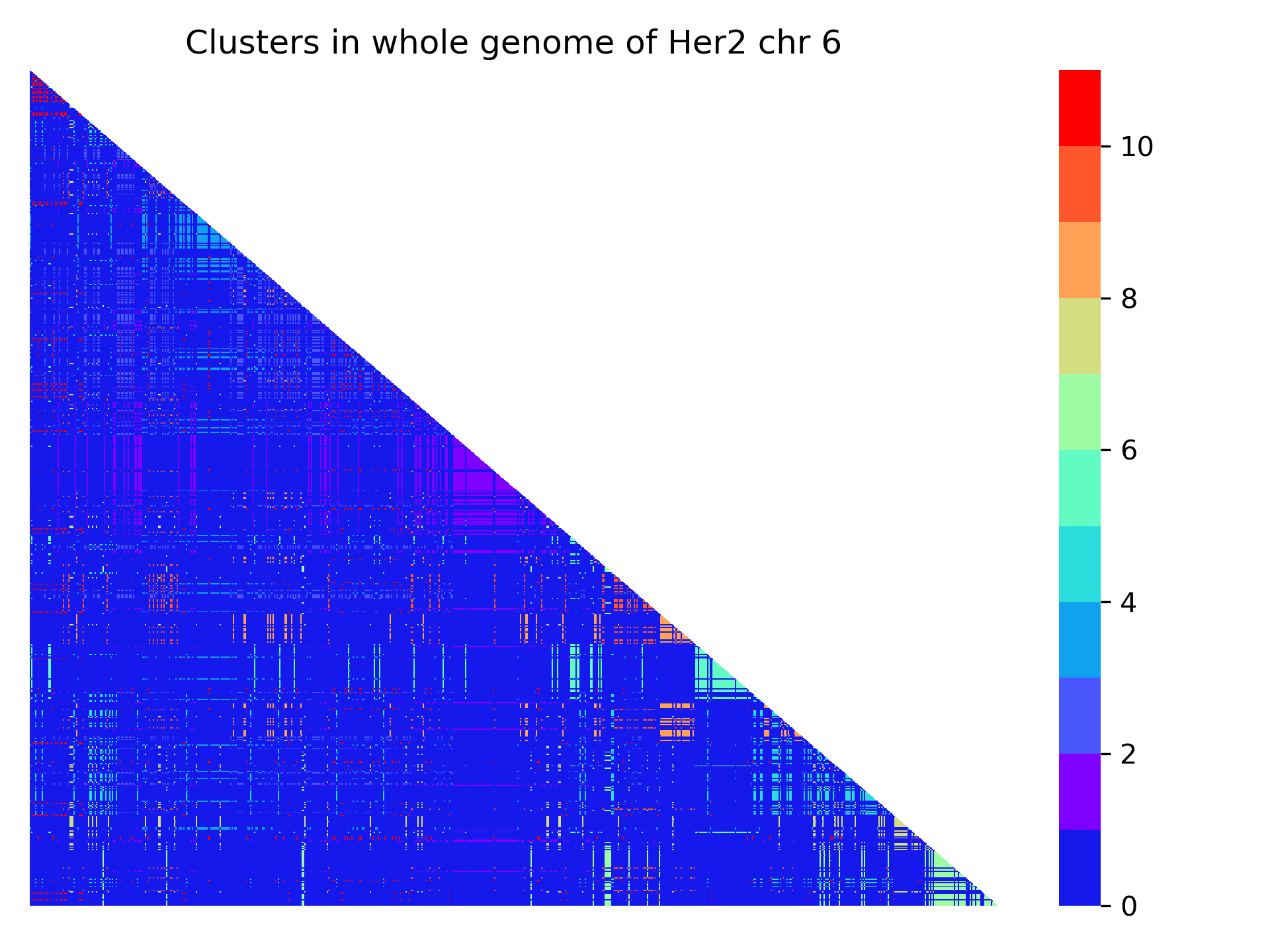

Supplement: Supplementary Material S13 — Piece-wise permutation p-values of the KS statistics, calculated for all bins obtained in Supplementary Material S8 , in every chromosomal region for each phenotype. [file DataSheet_13.zip › SuppMat10/SuppMat10/chr6/Her2-chr6-gstart-heat.png]

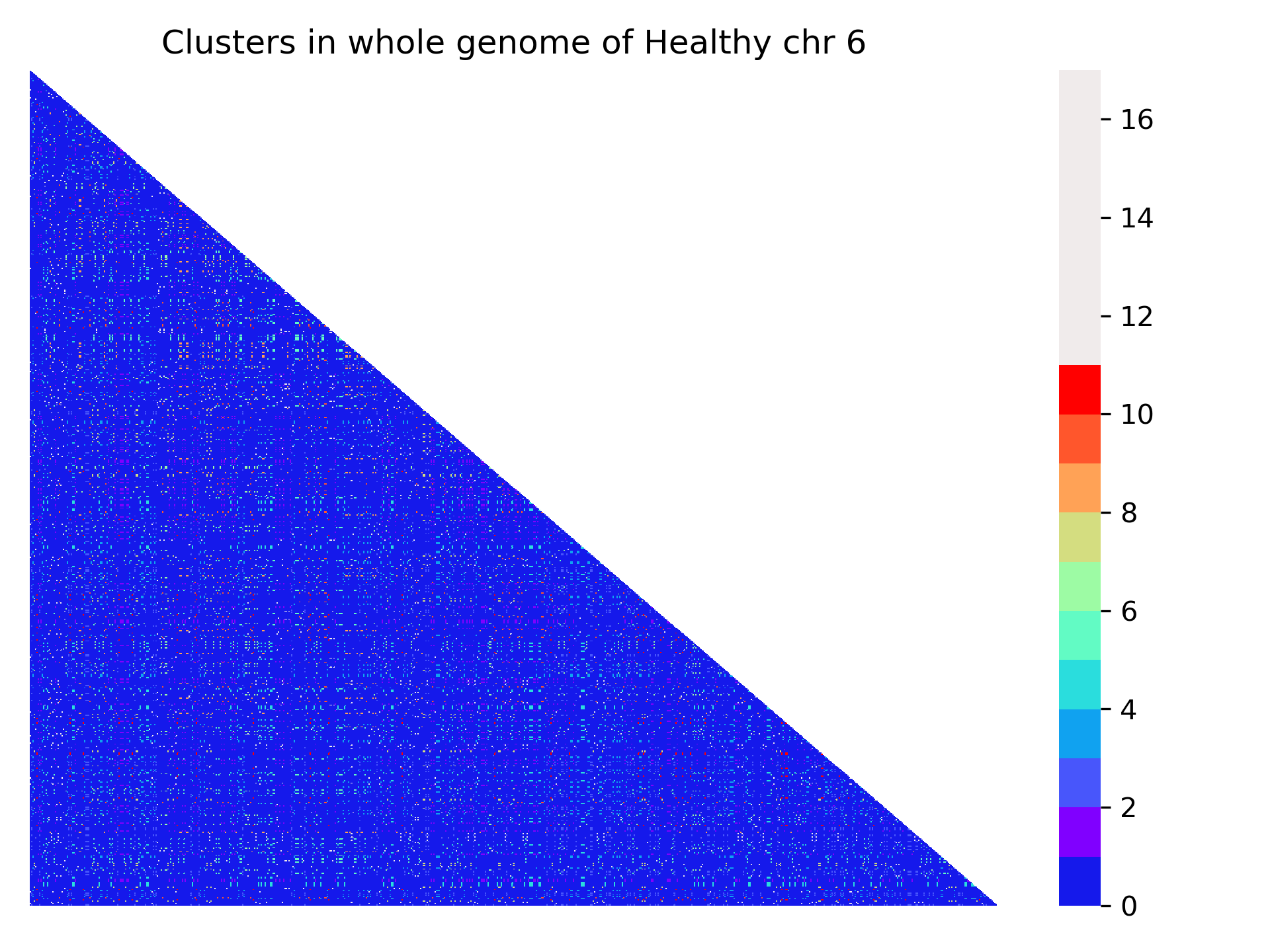

Supplement: Supplementary Material S13 — Piece-wise permutation p-values of the KS statistics, calculated for all bins obtained in Supplementary Material S8 , in every chromosomal region for each phenotype. [file DataSheet_13.zip › SuppMat10/SuppMat10/chr6/Healthy-chr6-gstart-heat.png]

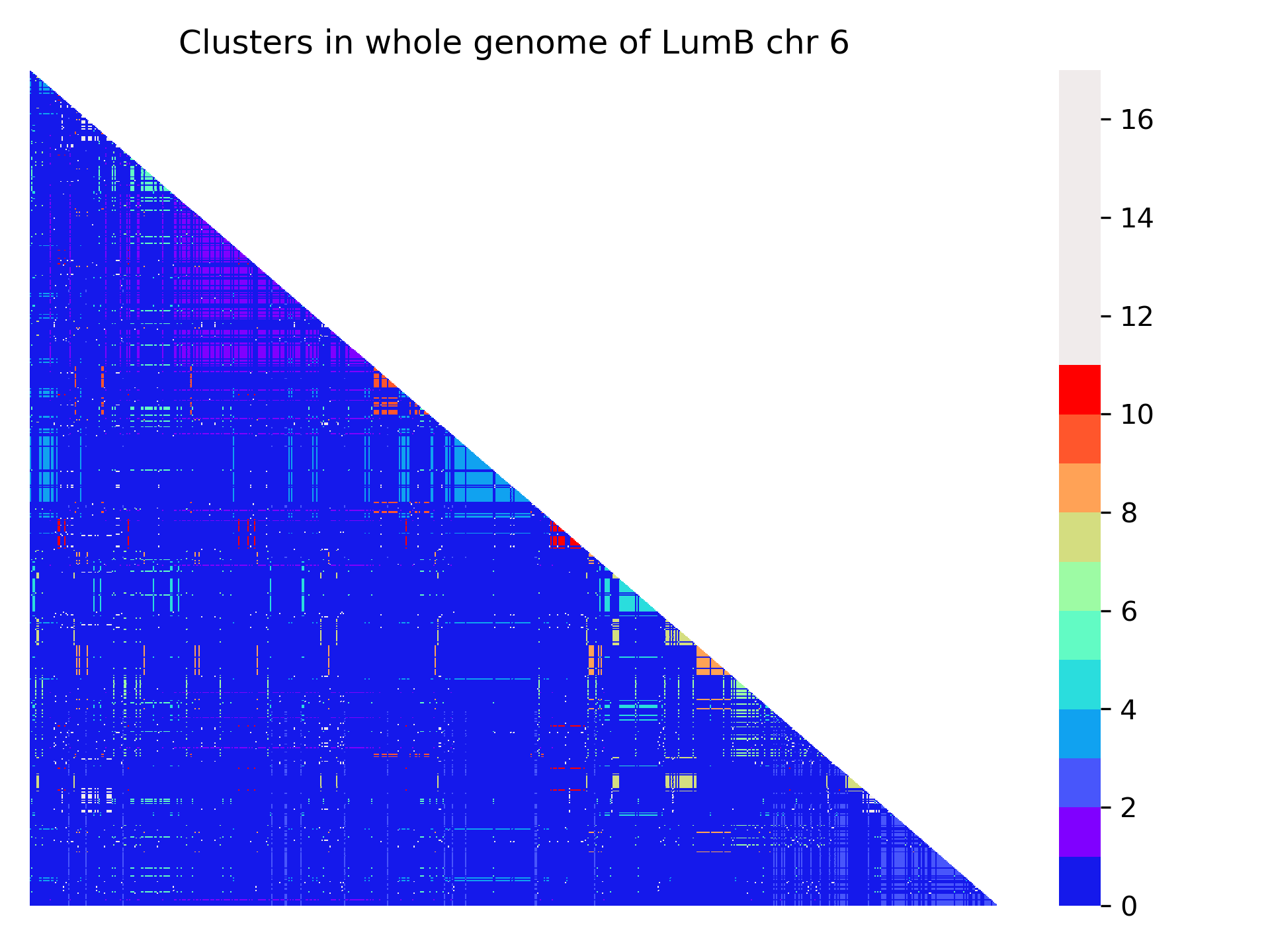

Supplement: Supplementary Material S13 — Piece-wise permutation p-values of the KS statistics, calculated for all bins obtained in Supplementary Material S8 , in every chromosomal region for each phenotype. [file DataSheet_13.zip › SuppMat10/SuppMat10/chr6/LumB-chr6-gstart-heat.png]

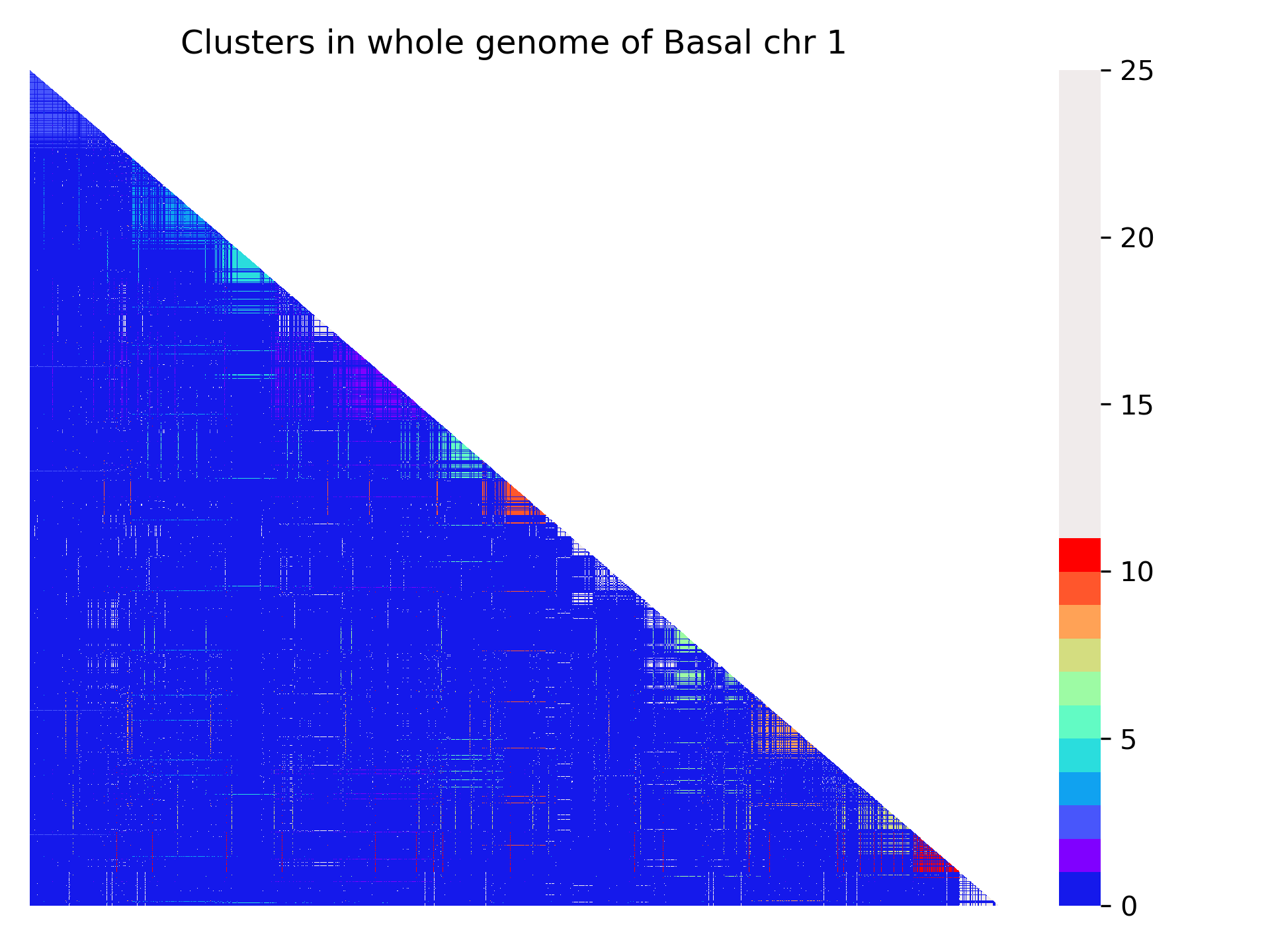

Supplement: Supplementary Material S13 — Piece-wise permutation p-values of the KS statistics, calculated for all bins obtained in Supplementary Material S8 , in every chromosomal region for each phenotype. [file DataSheet_13.zip › SuppMat10/SuppMat10/chr1/Basal-chr1-gstart-heat.png]

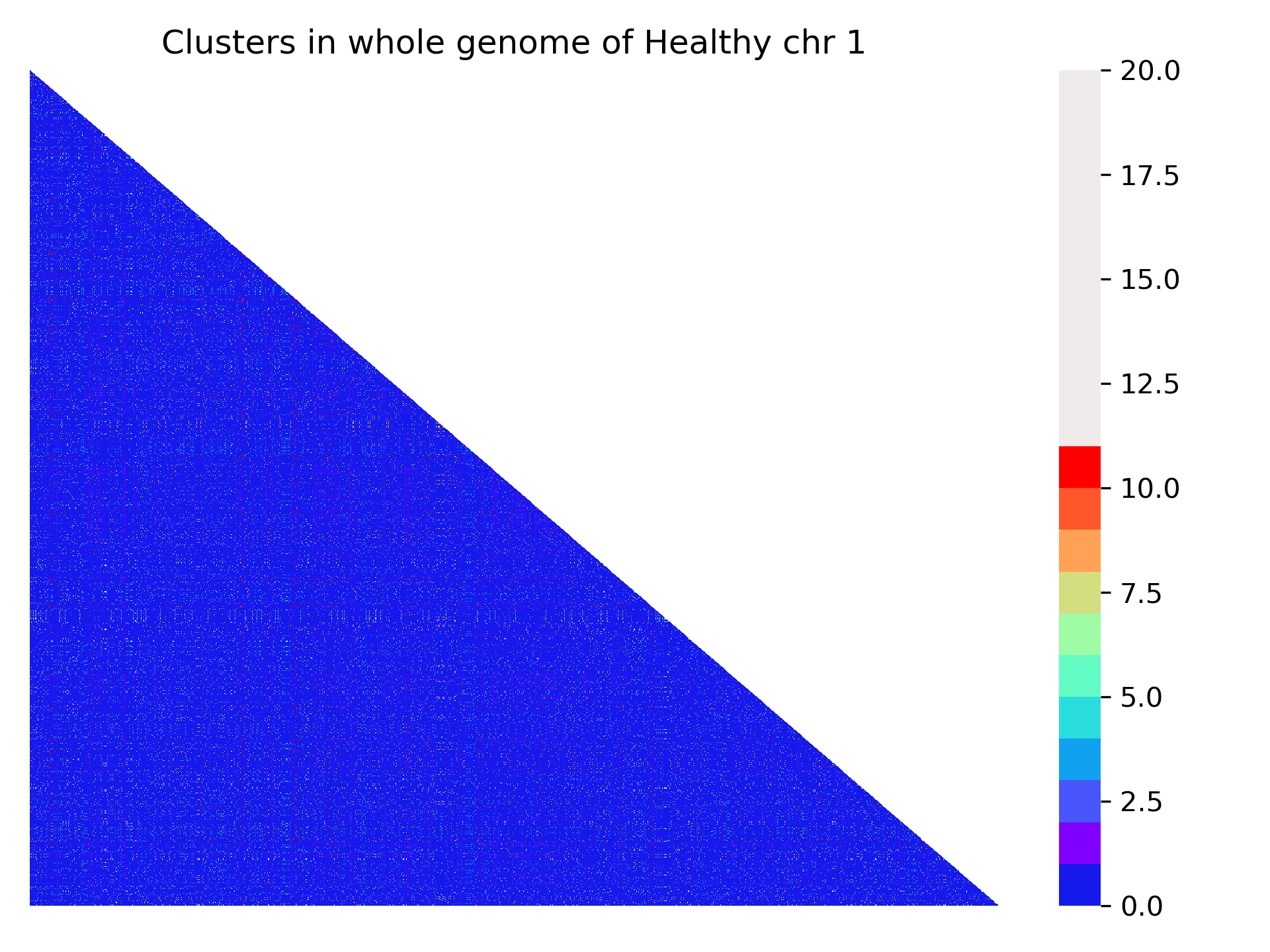

Supplement: Supplementary Material S13 — Piece-wise permutation p-values of the KS statistics, calculated for all bins obtained in Supplementary Material S8 , in every chromosomal region for each phenotype. [file DataSheet_13.zip › SuppMat10/SuppMat10/chr1/Healthy-chr1-gstart-heat.png]

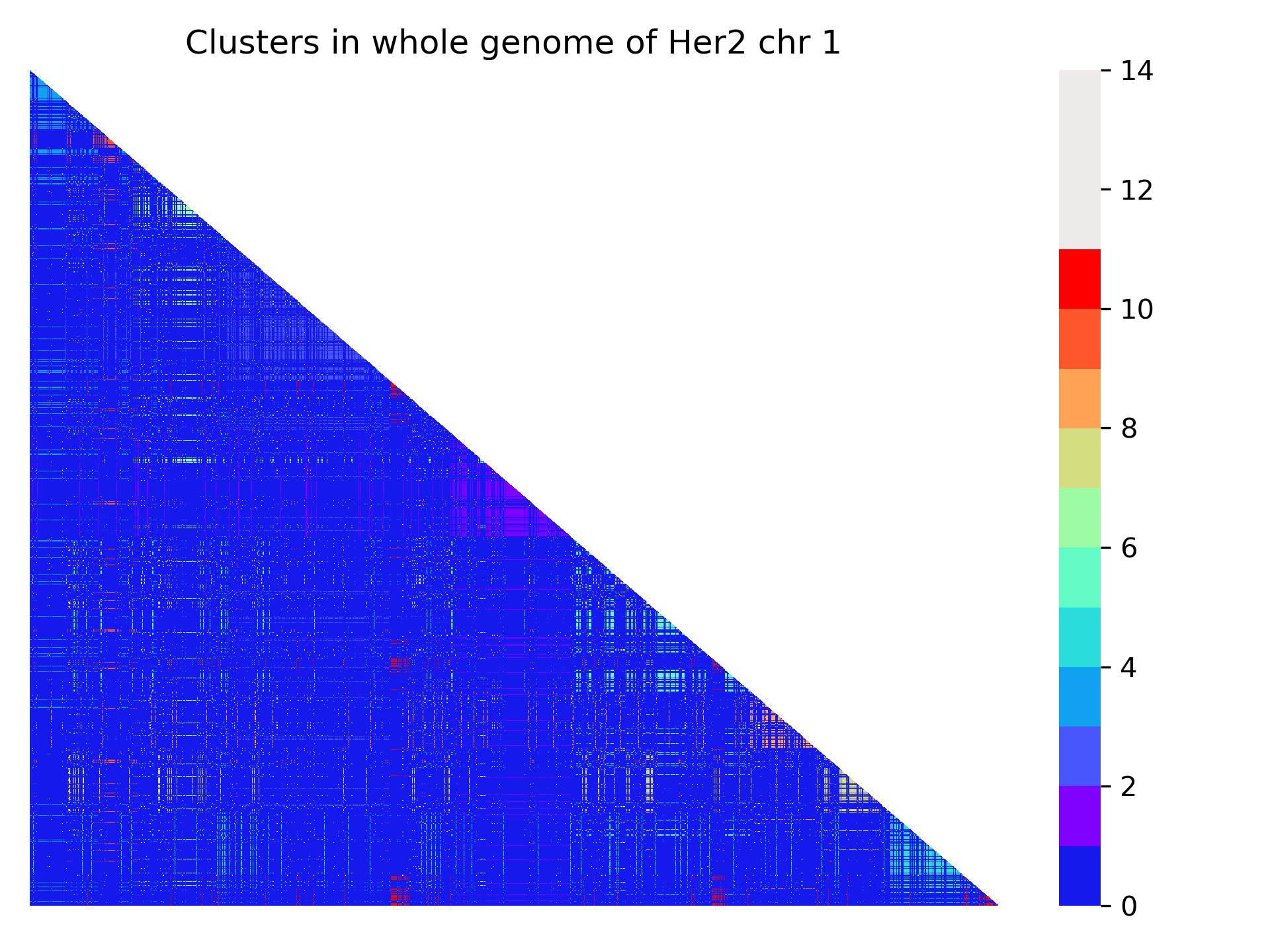

Supplement: Supplementary Material S13 — Piece-wise permutation p-values of the KS statistics, calculated for all bins obtained in Supplementary Material S8 , in every chromosomal region for each phenotype. [file DataSheet_13.zip › SuppMat10/SuppMat10/chr1/Her2-chr1-gstart-heat.png]

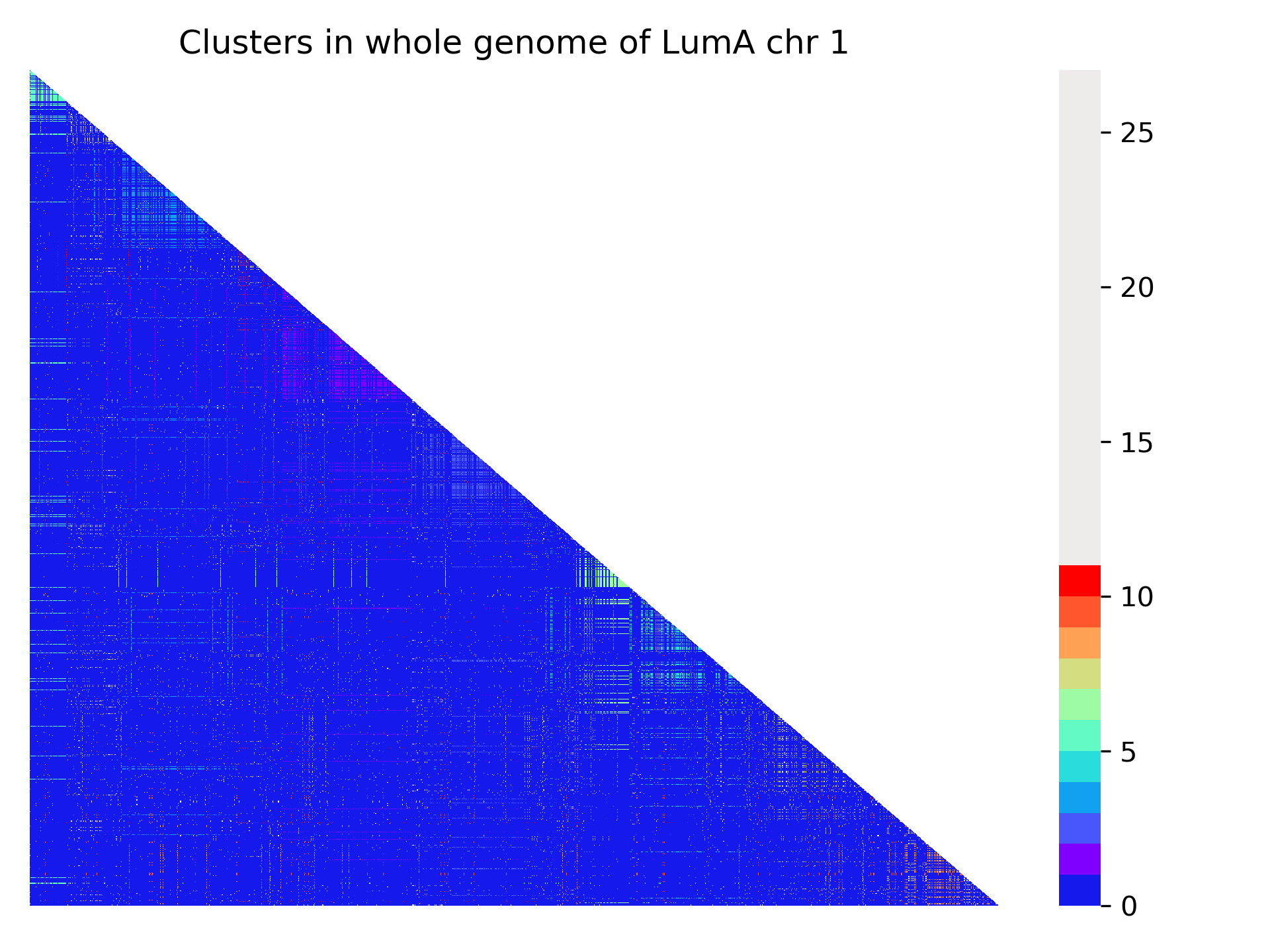

Supplement: Supplementary Material S13 — Piece-wise permutation p-values of the KS statistics, calculated for all bins obtained in Supplementary Material S8 , in every chromosomal region for each phenotype. [file DataSheet_13.zip › SuppMat10/SuppMat10/chr1/LumA-chr1-gstart-heat.png]

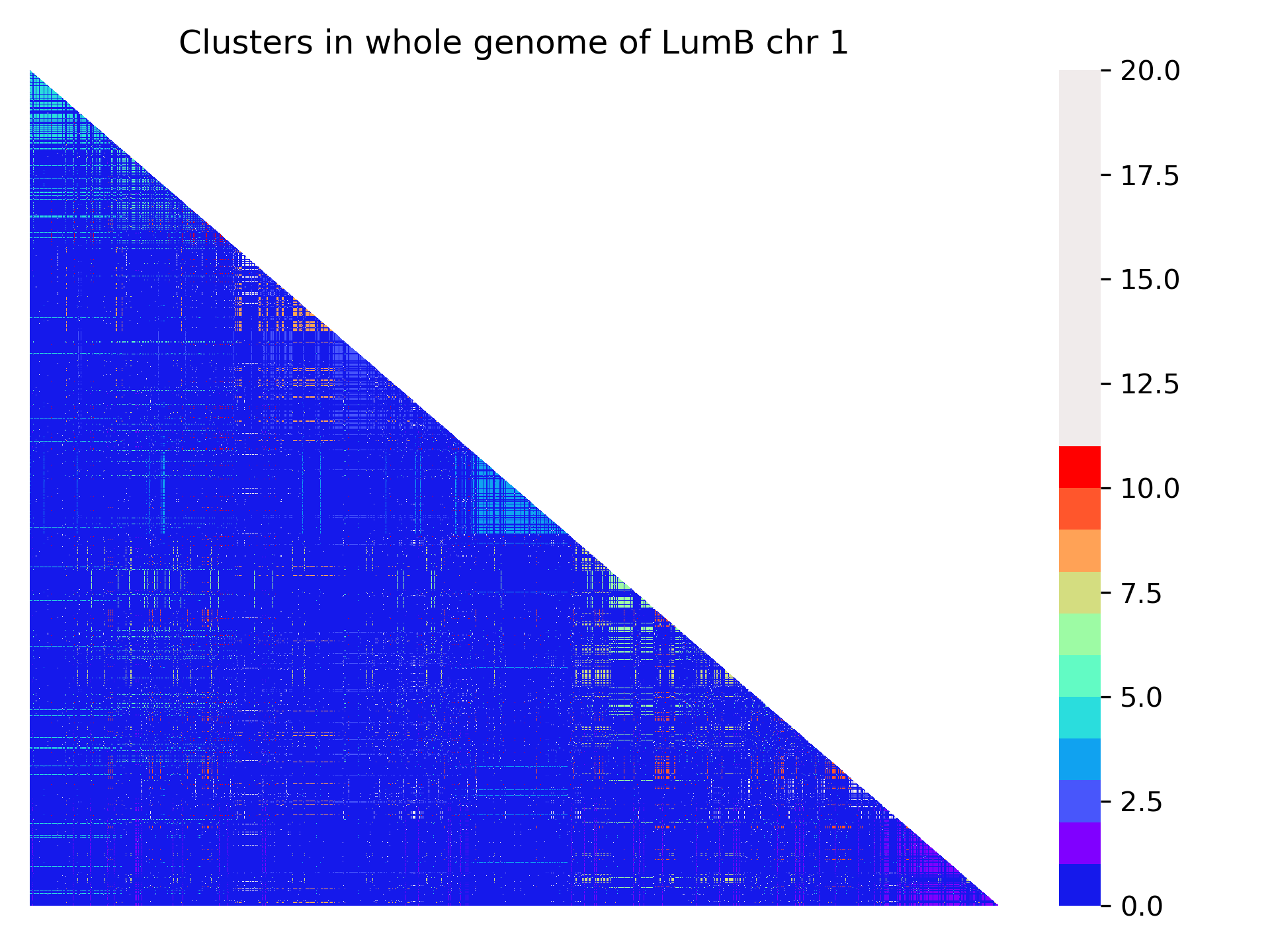

Supplement: Supplementary Material S13 — Piece-wise permutation p-values of the KS statistics, calculated for all bins obtained in Supplementary Material S8 , in every chromosomal region for each phenotype. [file DataSheet_13.zip › SuppMat10/SuppMat10/chr1/LumB-chr1-gstart-heat.png]

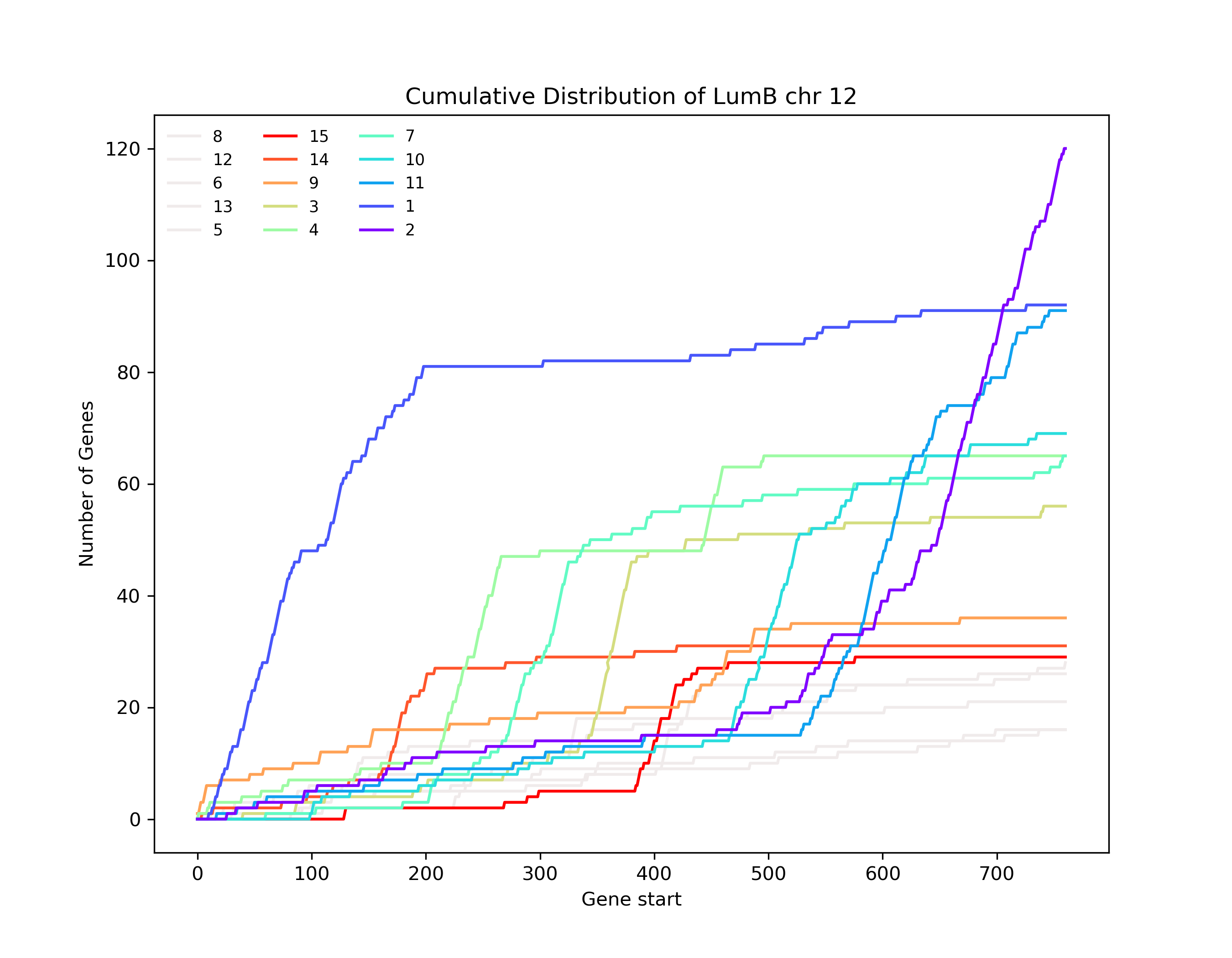

Supplement: Supplementary Material S14 — Clusters by chromosome for the five phenotypes, obtained by eigenvalue decomposition and k-medoids method. The figures are depicted as in the manuscript. Additionally, this material contains files for clusters including the name of the gene, the cluster that the gene belong to, the assignment cost function value, the chromosome location of the gene, and the gene start position of said gene. [file DataSheet_14.zip › SuppMat12/chr12/LumB-chr12-gstart-cum.png]

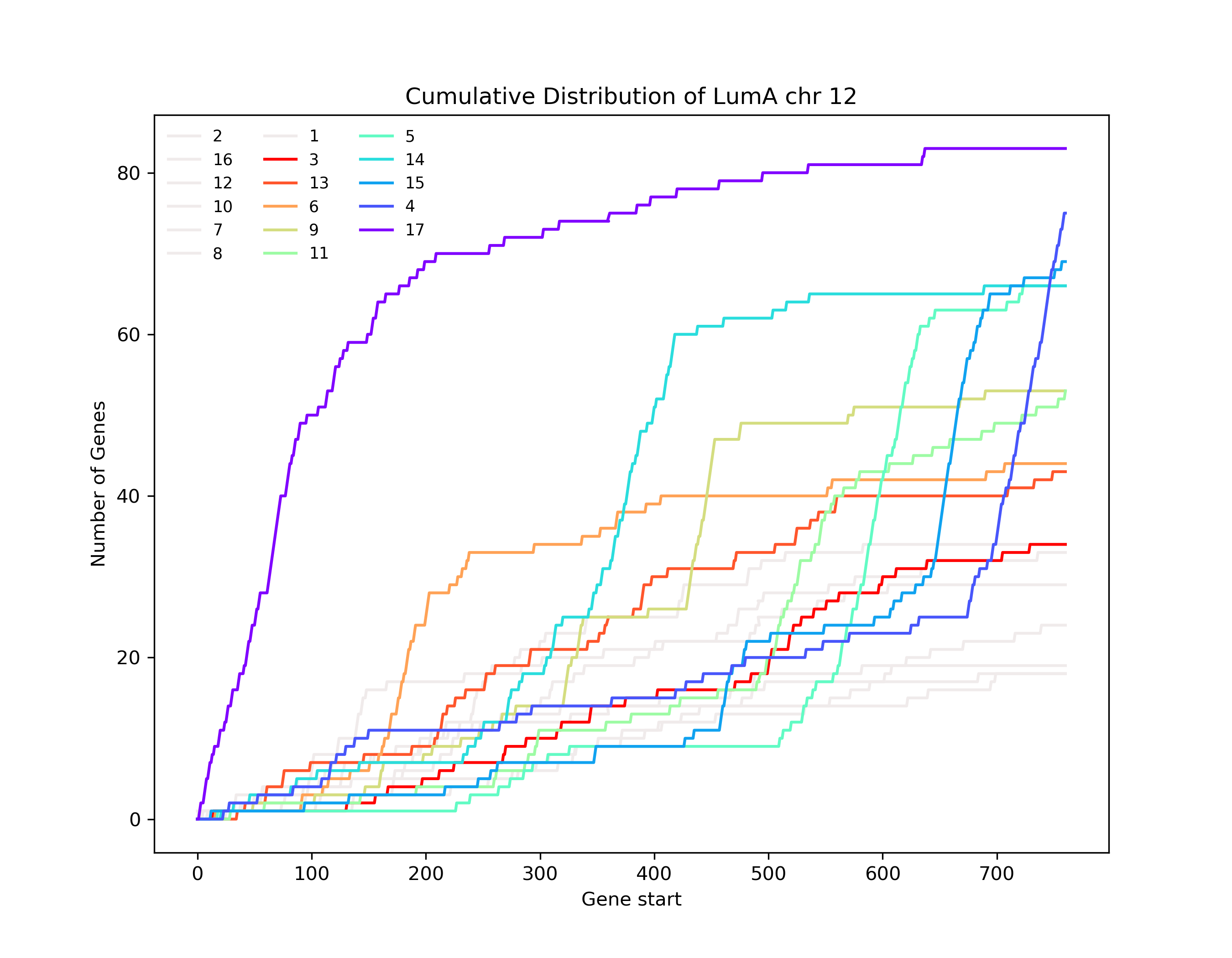

Supplement: Supplementary Material S14 — Clusters by chromosome for the five phenotypes, obtained by eigenvalue decomposition and k-medoids method. The figures are depicted as in the manuscript. Additionally, this material contains files for clusters including the name of the gene, the cluster that the gene belong to, the assignment cost function value, the chromosome location of the gene, and the gene start position of said gene. [file DataSheet_14.zip › SuppMat12/chr12/LumA-chr12-gstart-cum.png]

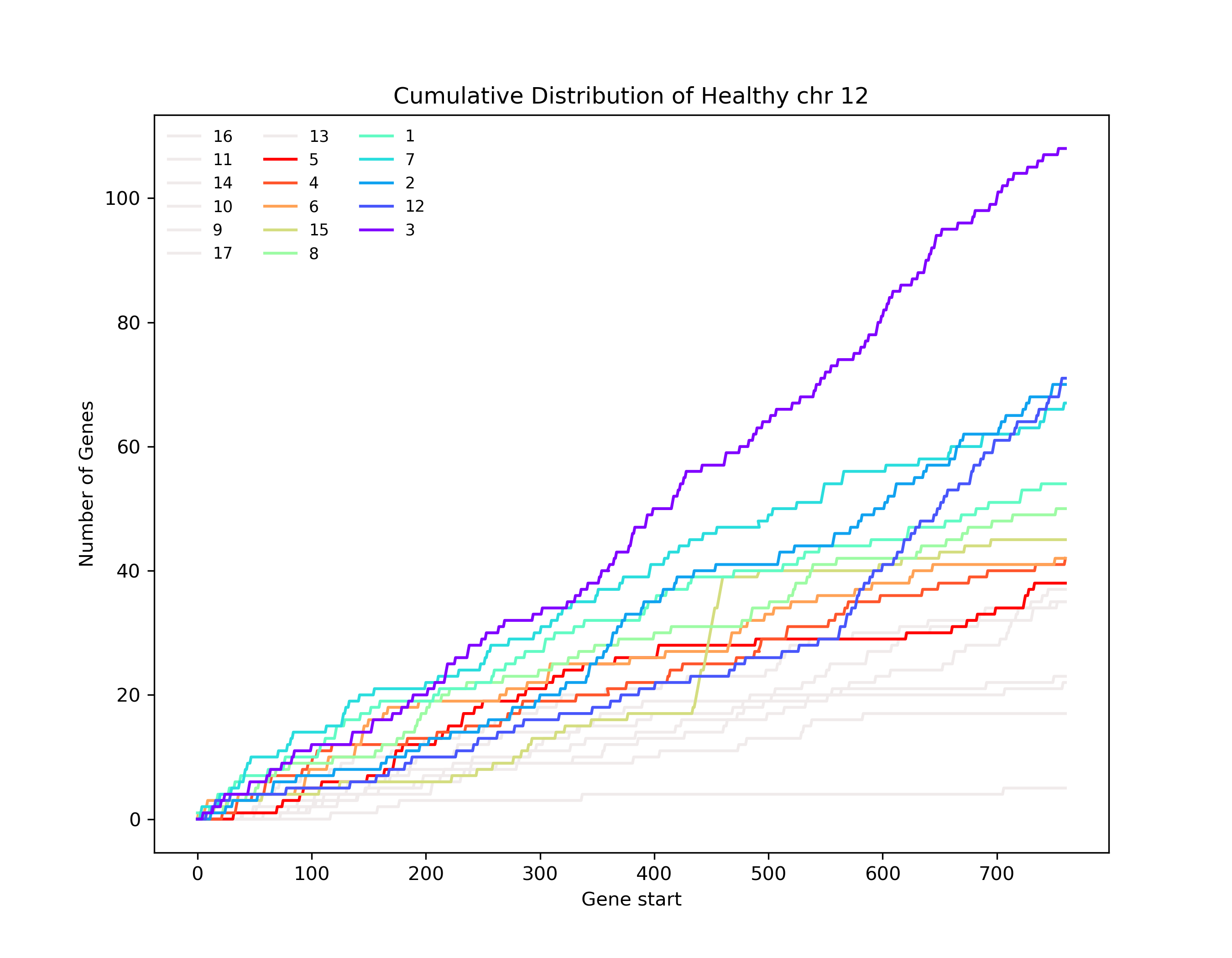

Supplement: Supplementary Material S14 — Clusters by chromosome for the five phenotypes, obtained by eigenvalue decomposition and k-medoids method. The figures are depicted as in the manuscript. Additionally, this material contains files for clusters including the name of the gene, the cluster that the gene belong to, the assignment cost function value, the chromosome location of the gene, and the gene start position of said gene. [file DataSheet_14.zip › SuppMat12/chr12/Healthy-chr12-gstart-cum.png]

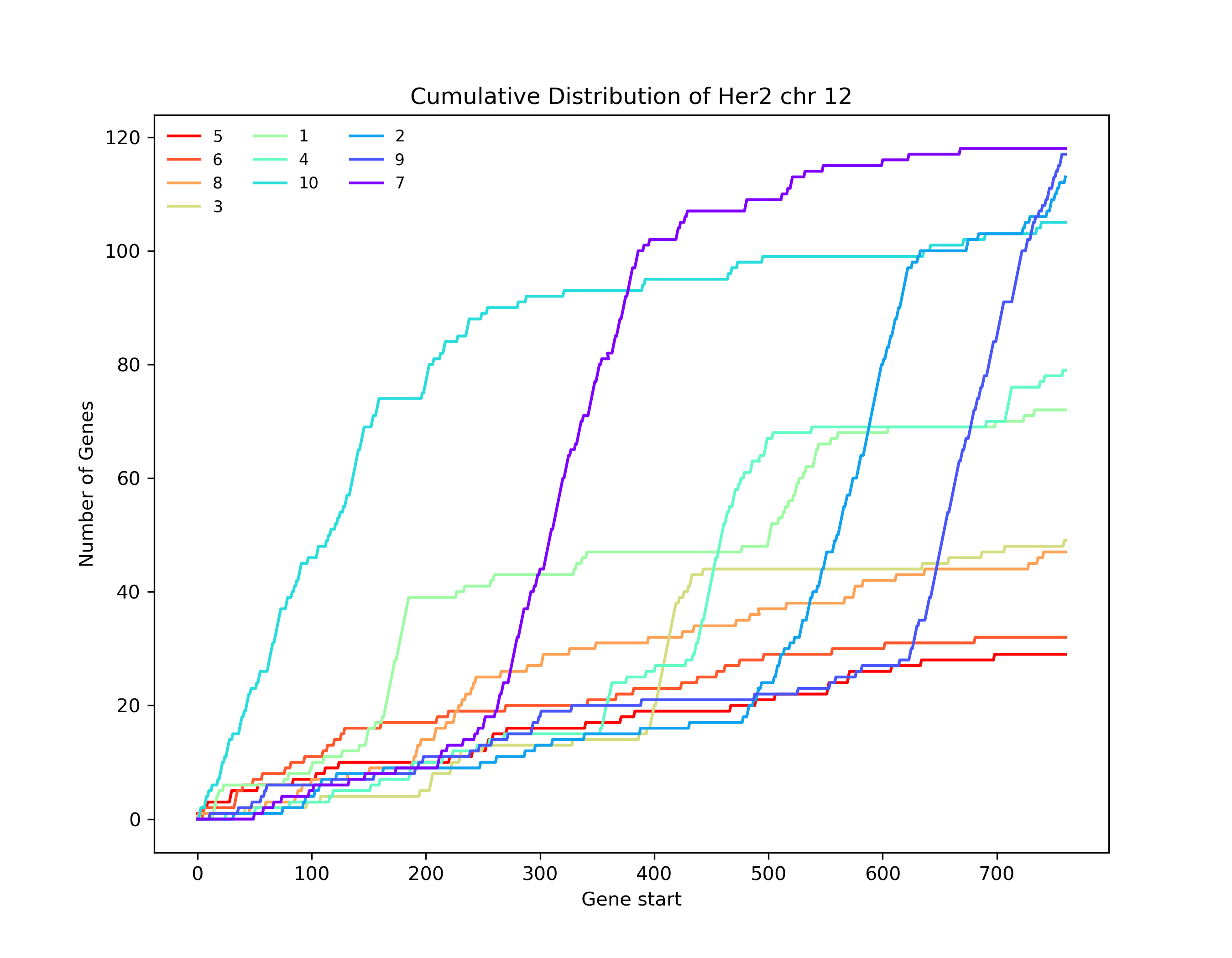

Supplement: Supplementary Material S14 — Clusters by chromosome for the five phenotypes, obtained by eigenvalue decomposition and k-medoids method. The figures are depicted as in the manuscript. Additionally, this material contains files for clusters including the name of the gene, the cluster that the gene belong to, the assignment cost function value, the chromosome location of the gene, and the gene start position of said gene. [file DataSheet_14.zip › SuppMat12/chr12/Her2-chr12-gstart-cum.png]

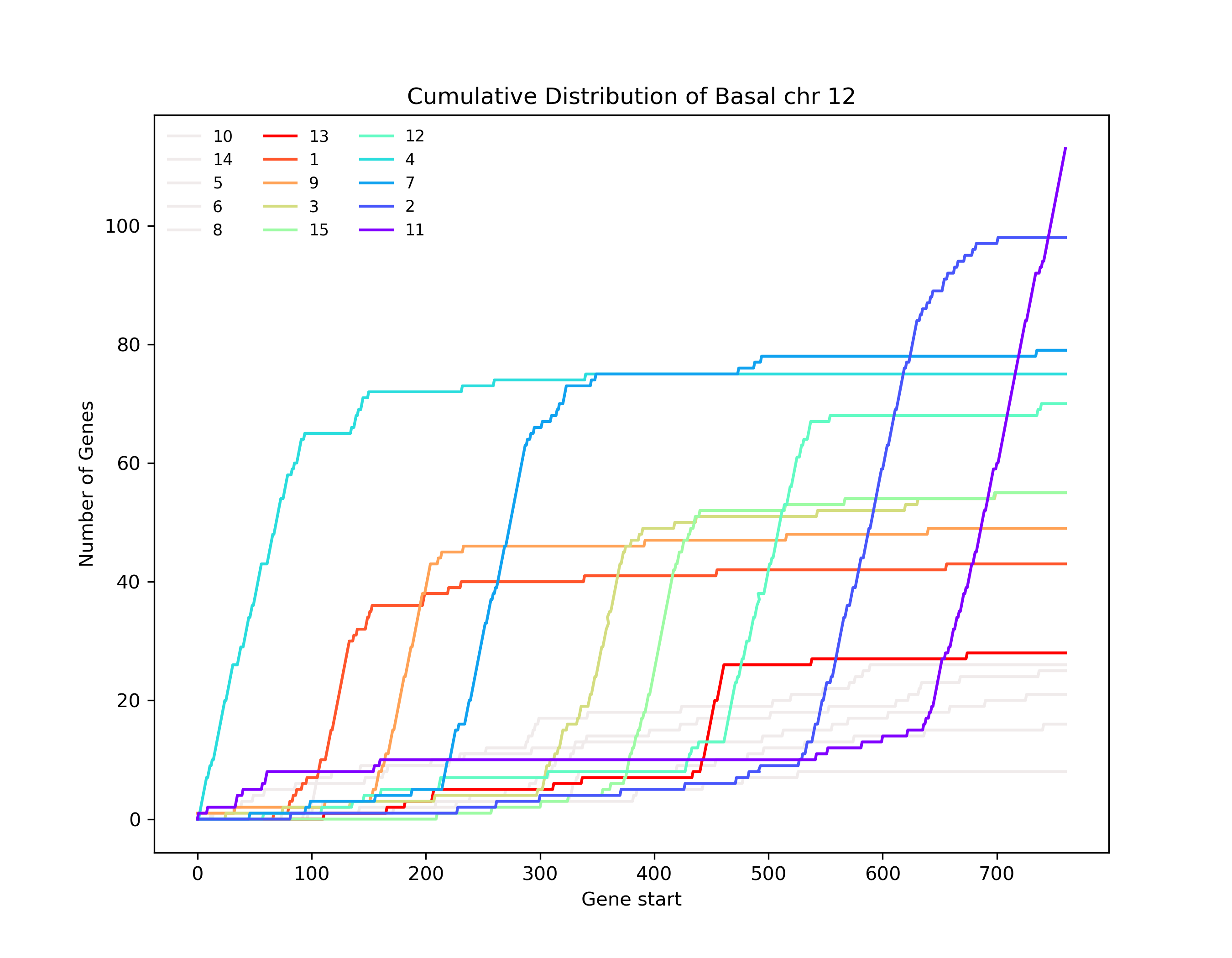

Supplement: Supplementary Material S14 — Clusters by chromosome for the five phenotypes, obtained by eigenvalue decomposition and k-medoids method. The figures are depicted as in the manuscript. Additionally, this material contains files for clusters including the name of the gene, the cluster that the gene belong to, the assignment cost function value, the chromosome location of the gene, and the gene start position of said gene. [file DataSheet_14.zip › SuppMat12/chr12/Basal-chr12-gstart-cum.png]

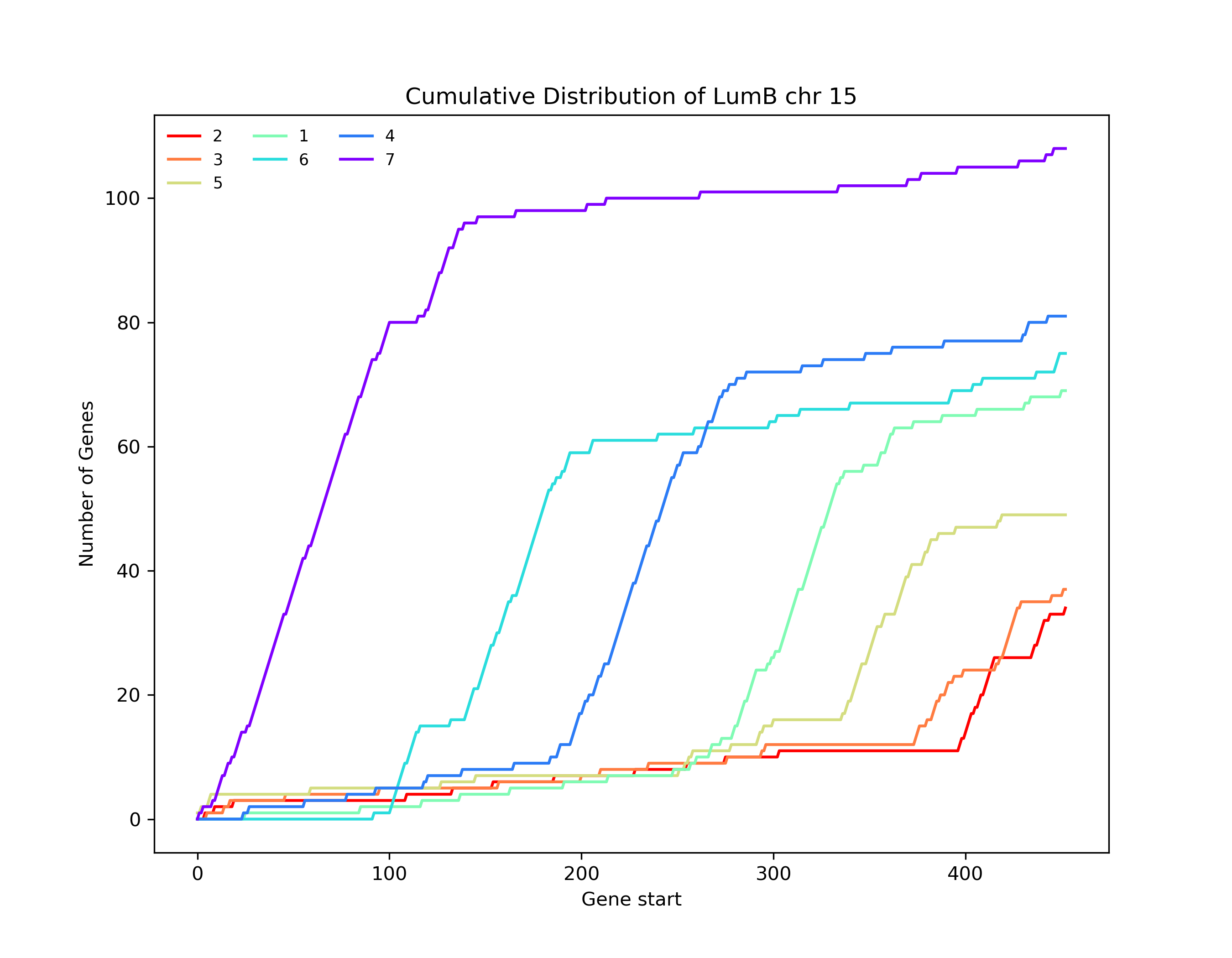

Supplement: Supplementary Material S14 — Clusters by chromosome for the five phenotypes, obtained by eigenvalue decomposition and k-medoids method. The figures are depicted as in the manuscript. Additionally, this material contains files for clusters including the name of the gene, the cluster that the gene belong to, the assignment cost function value, the chromosome location of the gene, and the gene start position of said gene. [file DataSheet_14.zip › SuppMat12/chr15/LumB-chr15-gstart-cum.png]

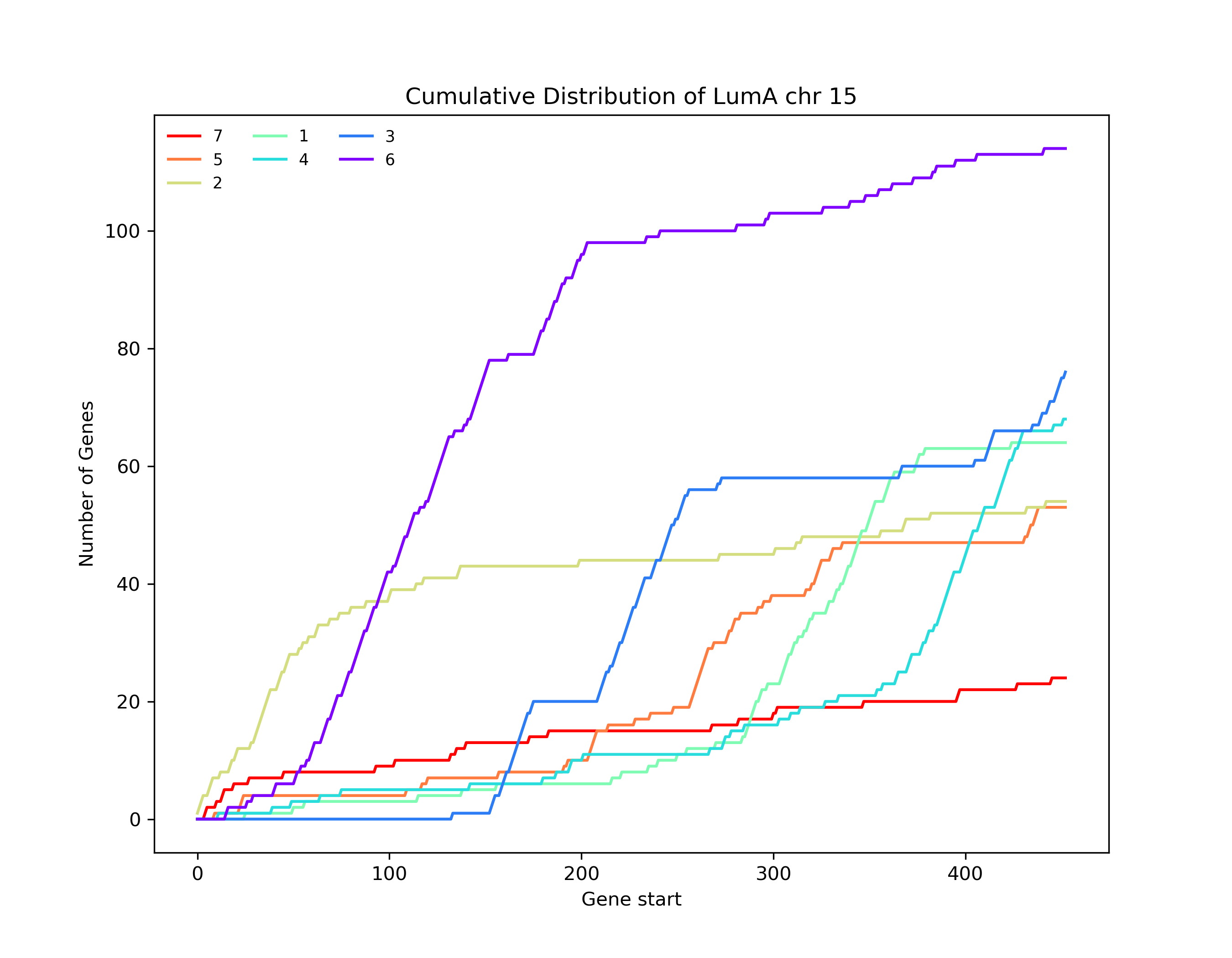

Supplement: Supplementary Material S14 — Clusters by chromosome for the five phenotypes, obtained by eigenvalue decomposition and k-medoids method. The figures are depicted as in the manuscript. Additionally, this material contains files for clusters including the name of the gene, the cluster that the gene belong to, the assignment cost function value, the chromosome location of the gene, and the gene start position of said gene. [file DataSheet_14.zip › SuppMat12/chr15/LumA-chr15-gstart-cum.png]

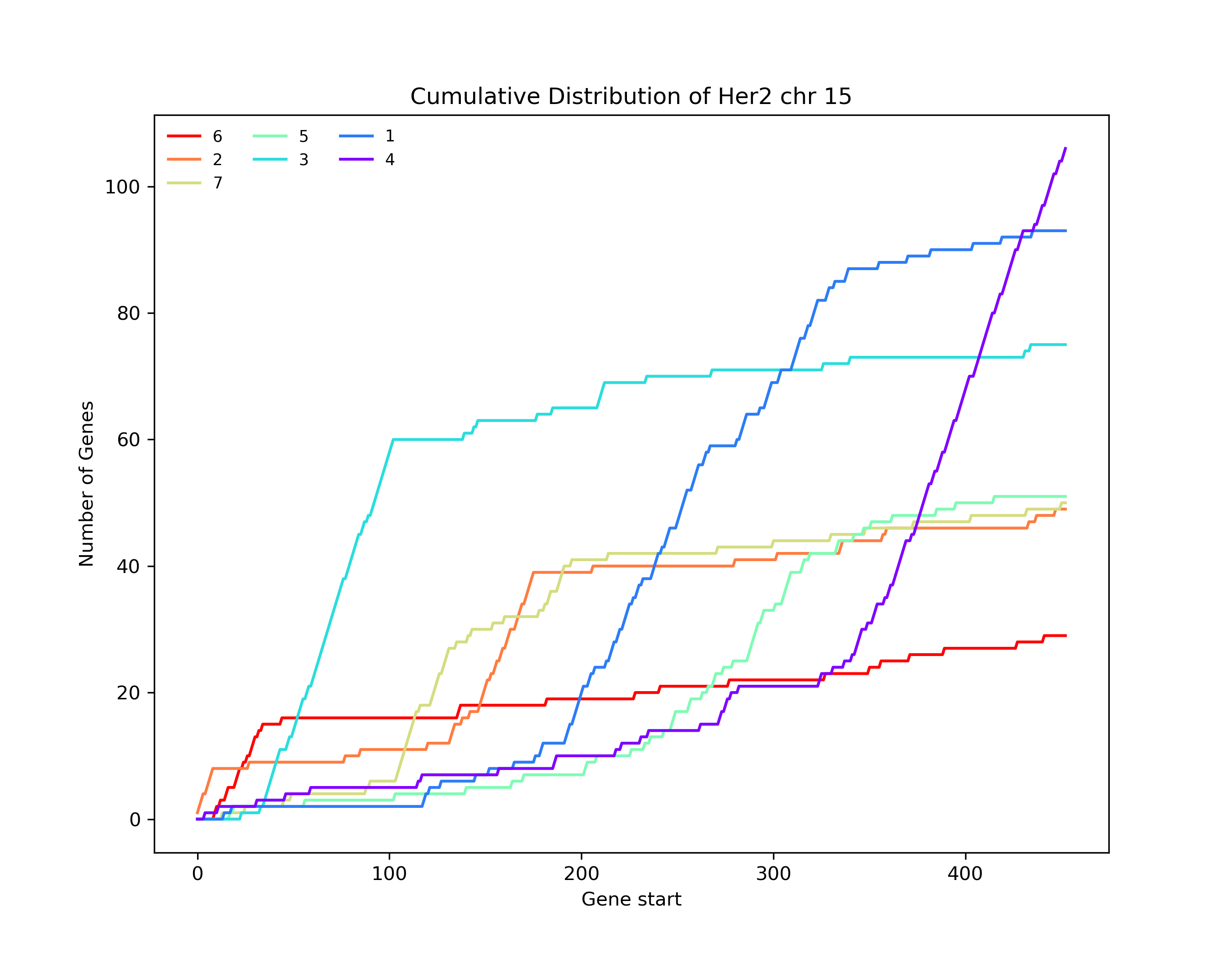

Supplement: Supplementary Material S14 — Clusters by chromosome for the five phenotypes, obtained by eigenvalue decomposition and k-medoids method. The figures are depicted as in the manuscript. Additionally, this material contains files for clusters including the name of the gene, the cluster that the gene belong to, the assignment cost function value, the chromosome location of the gene, and the gene start position of said gene. [file DataSheet_14.zip › SuppMat12/chr15/Her2-chr15-gstart-cum.png]

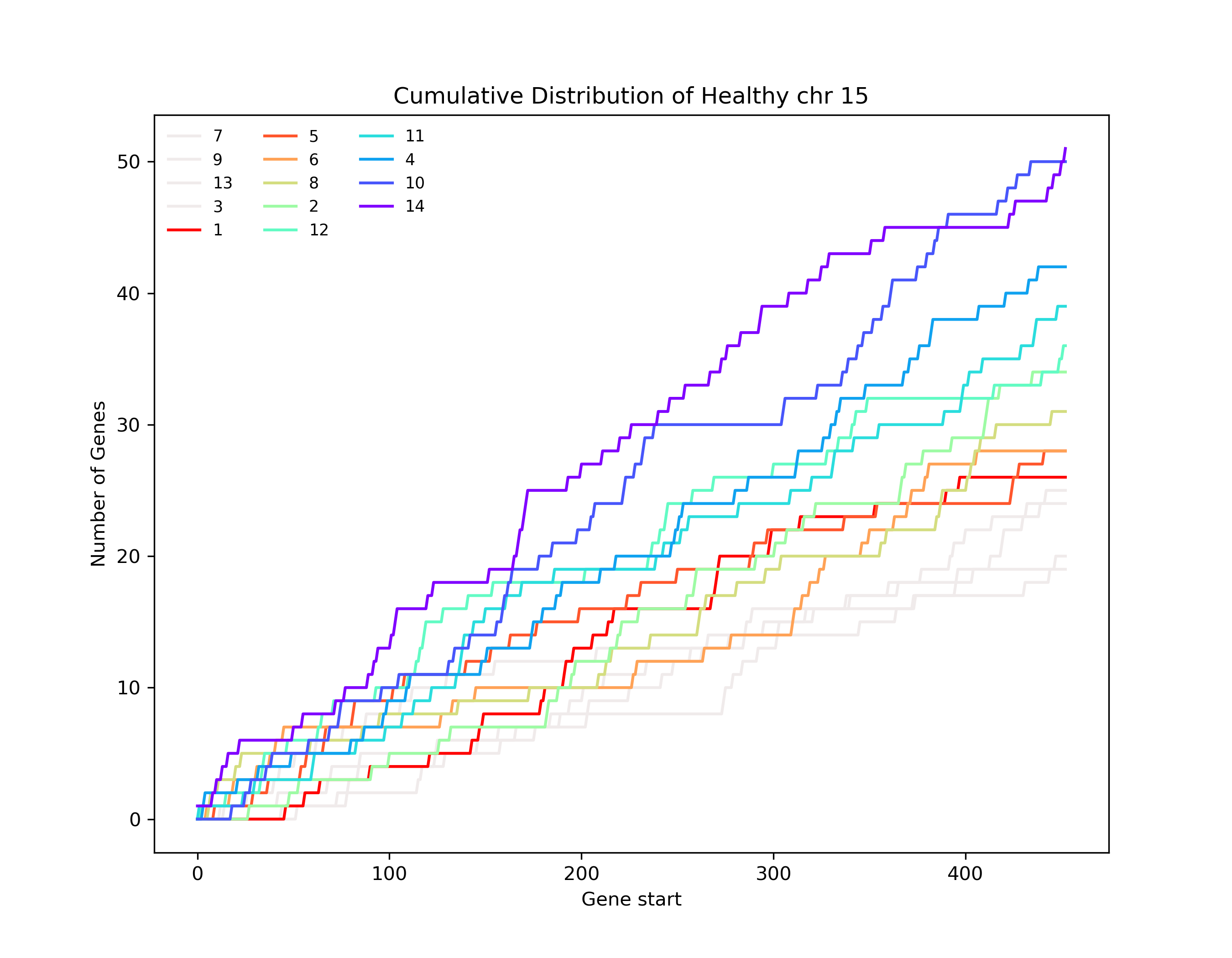

Supplement: Supplementary Material S14 — Clusters by chromosome for the five phenotypes, obtained by eigenvalue decomposition and k-medoids method. The figures are depicted as in the manuscript. Additionally, this material contains files for clusters including the name of the gene, the cluster that the gene belong to, the assignment cost function value, the chromosome location of the gene, and the gene start position of said gene. [file DataSheet_14.zip › SuppMat12/chr15/Healthy-chr15-gstart-cum.png]

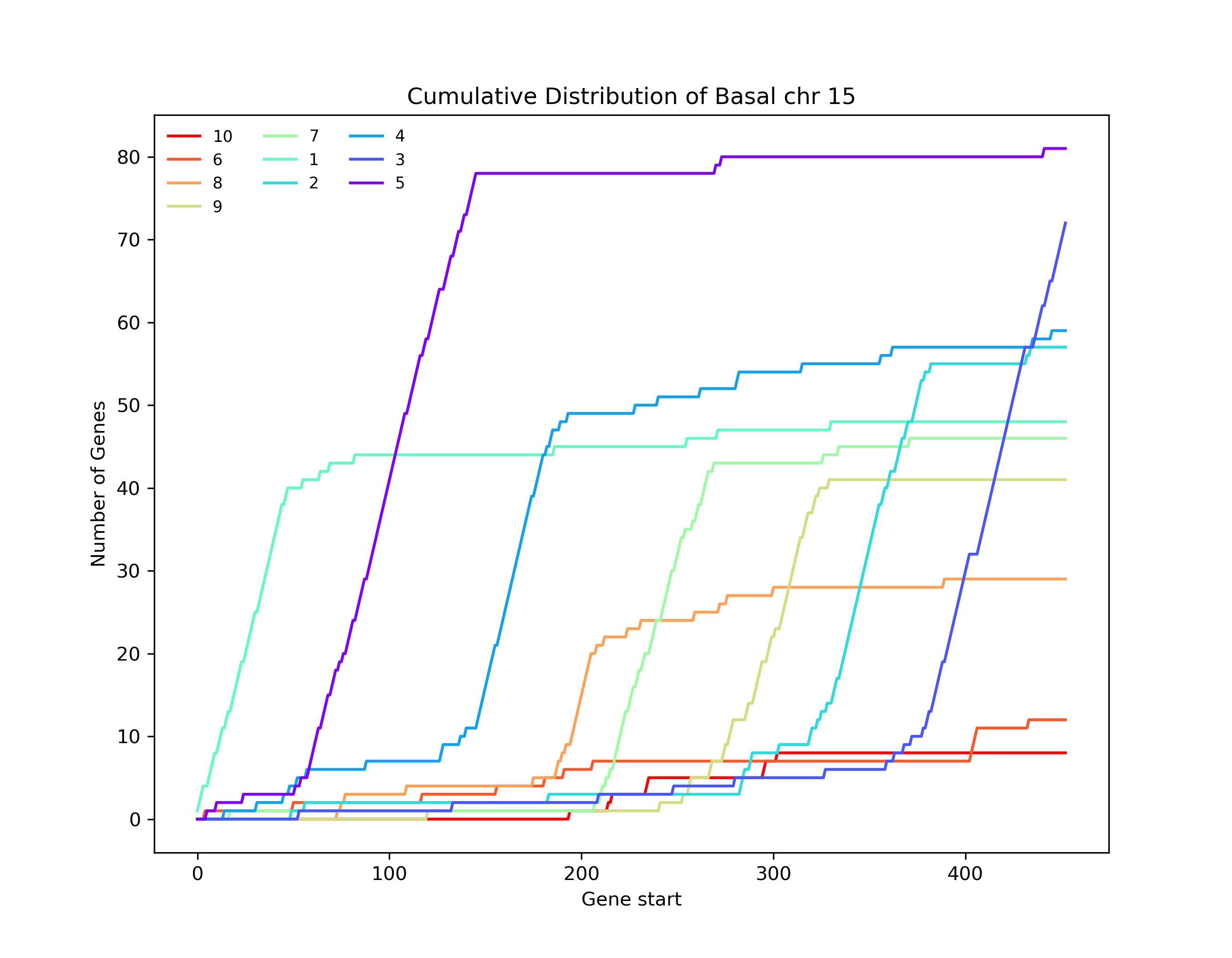

Supplement: Supplementary Material S14 — Clusters by chromosome for the five phenotypes, obtained by eigenvalue decomposition and k-medoids method. The figures are depicted as in the manuscript. Additionally, this material contains files for clusters including the name of the gene, the cluster that the gene belong to, the assignment cost function value, the chromosome location of the gene, and the gene start position of said gene. [file DataSheet_14.zip › SuppMat12/chr15/Basal-chr15-gstart-cum.png]

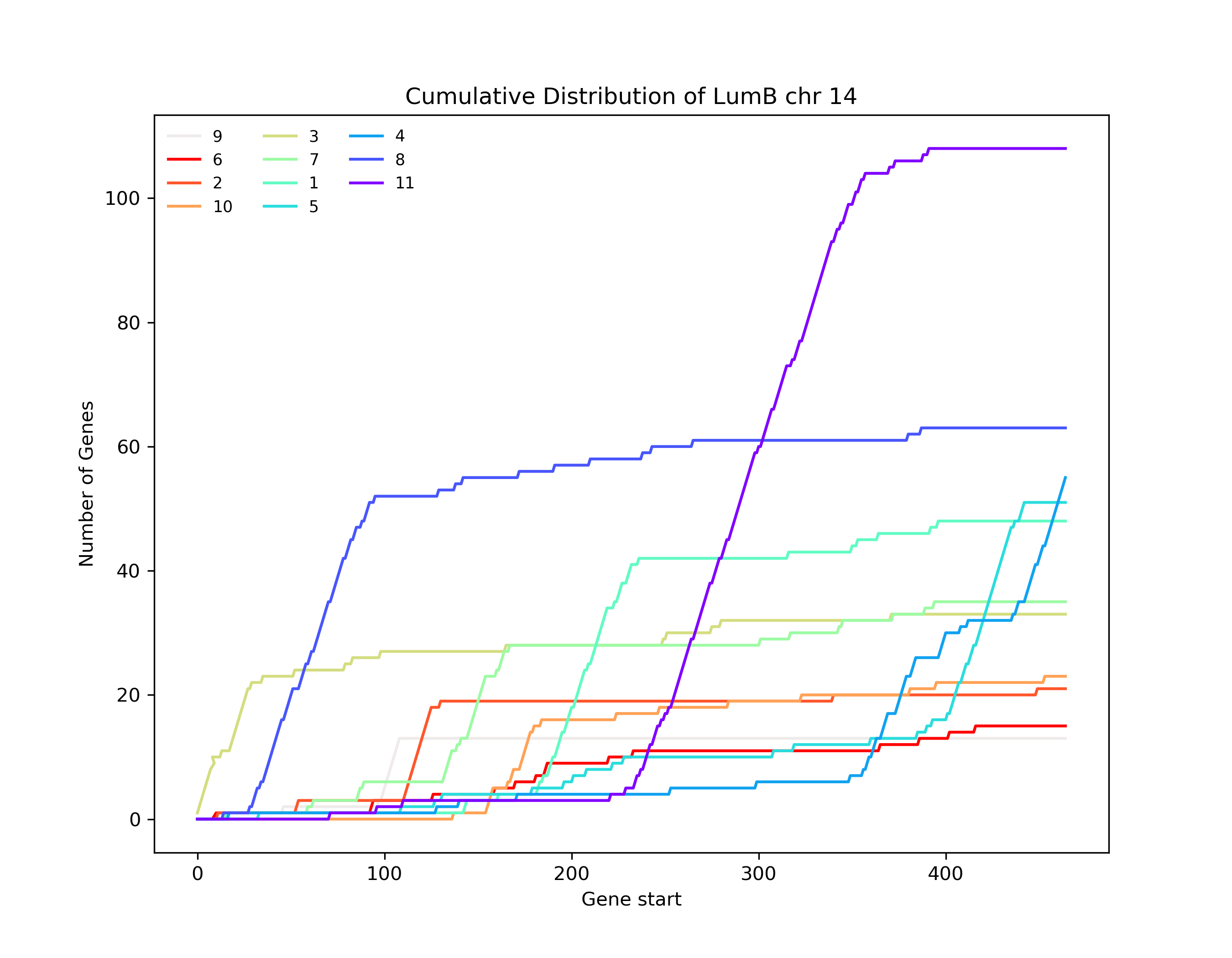

Supplement: Supplementary Material S14 — Clusters by chromosome for the five phenotypes, obtained by eigenvalue decomposition and k-medoids method. The figures are depicted as in the manuscript. Additionally, this material contains files for clusters including the name of the gene, the cluster that the gene belong to, the assignment cost function value, the chromosome location of the gene, and the gene start position of said gene. [file DataSheet_14.zip › SuppMat12/chr14/LumB-chr14-gstart-cum.png]

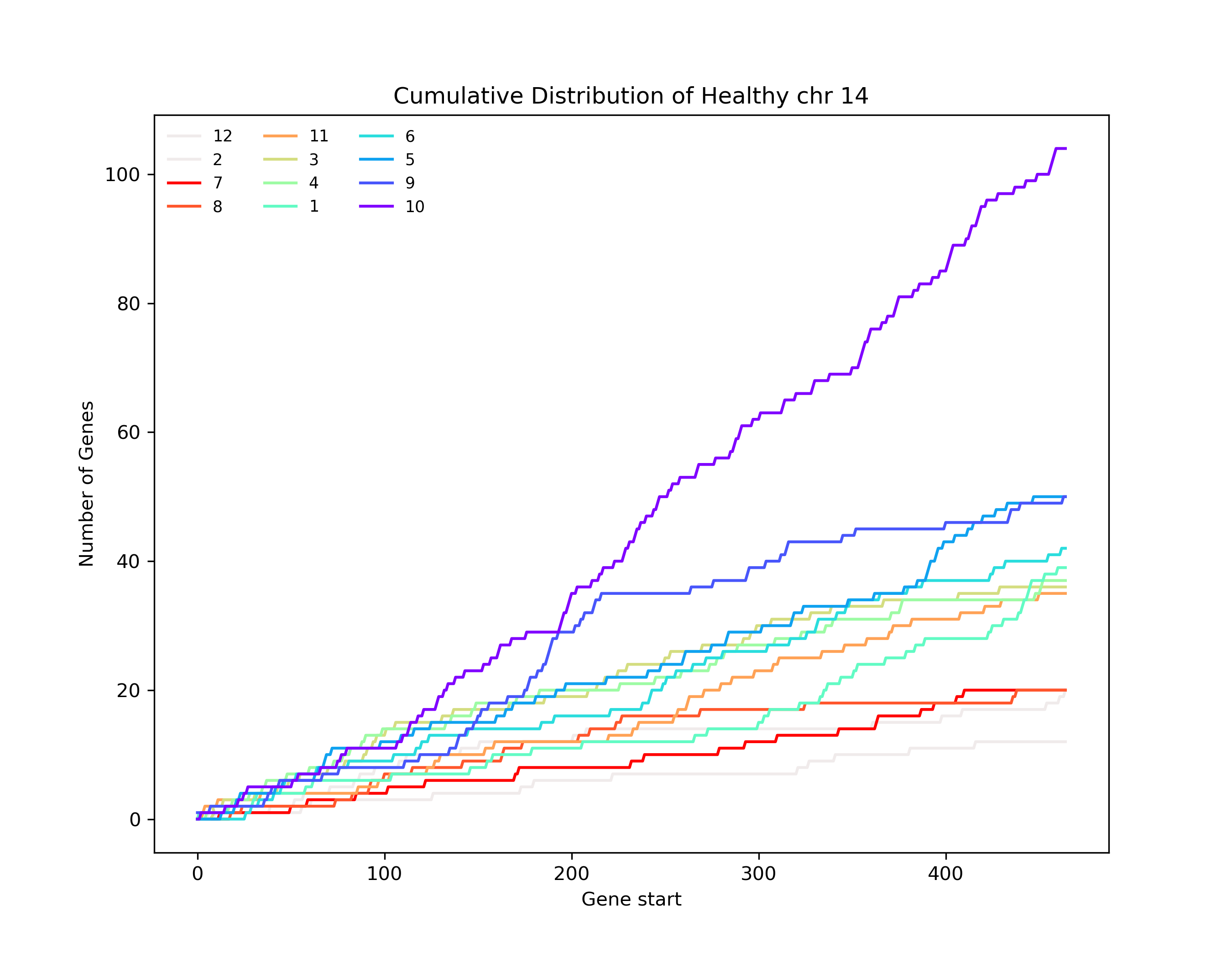

Supplement: Supplementary Material S14 — Clusters by chromosome for the five phenotypes, obtained by eigenvalue decomposition and k-medoids method. The figures are depicted as in the manuscript. Additionally, this material contains files for clusters including the name of the gene, the cluster that the gene belong to, the assignment cost function value, the chromosome location of the gene, and the gene start position of said gene. [file DataSheet_14.zip › SuppMat12/chr14/Healthy-chr14-gstart-cum.png]

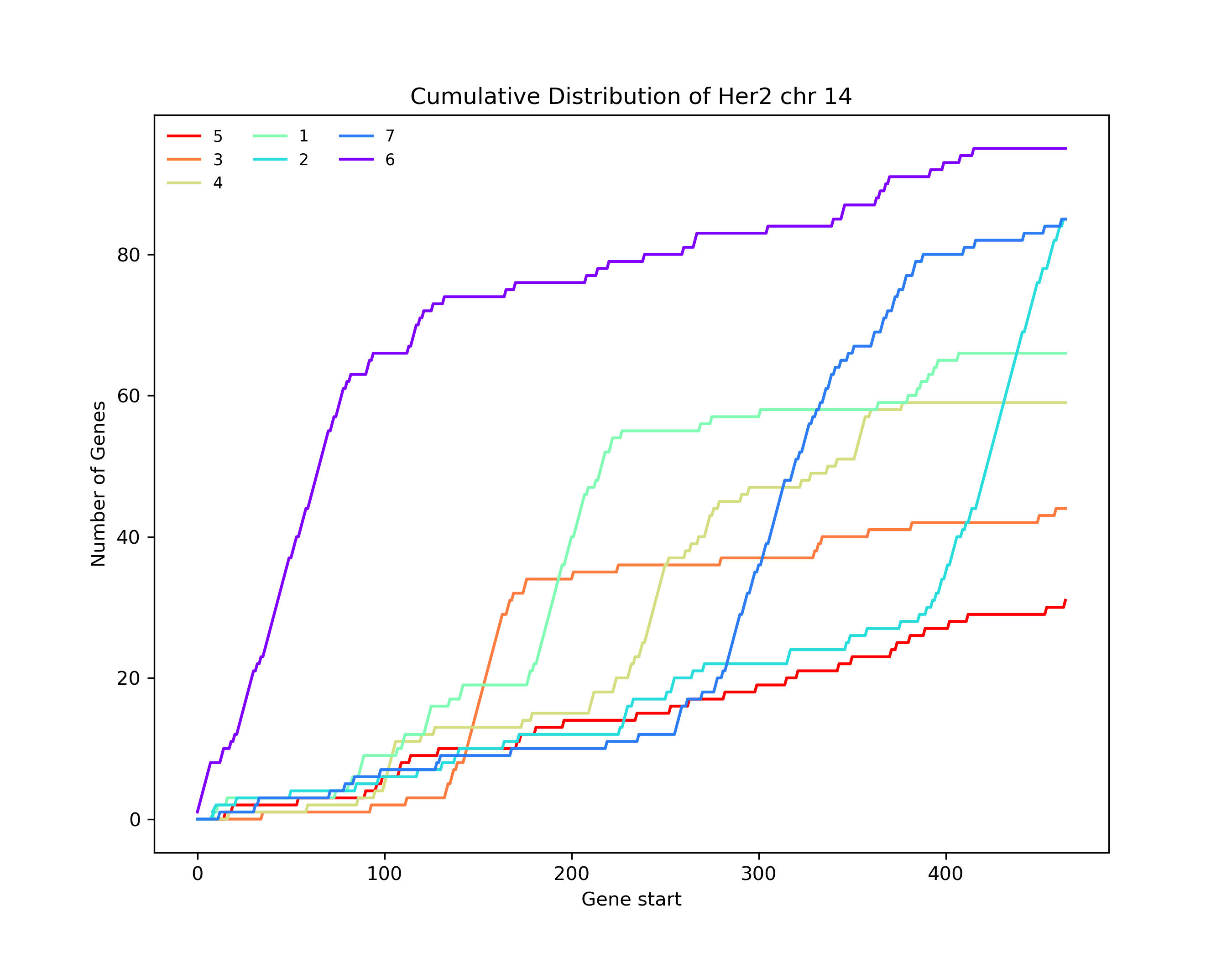

Supplement: Supplementary Material S14 — Clusters by chromosome for the five phenotypes, obtained by eigenvalue decomposition and k-medoids method. The figures are depicted as in the manuscript. Additionally, this material contains files for clusters including the name of the gene, the cluster that the gene belong to, the assignment cost function value, the chromosome location of the gene, and the gene start position of said gene. [file DataSheet_14.zip › SuppMat12/chr14/Her2-chr14-gstart-cum.png]

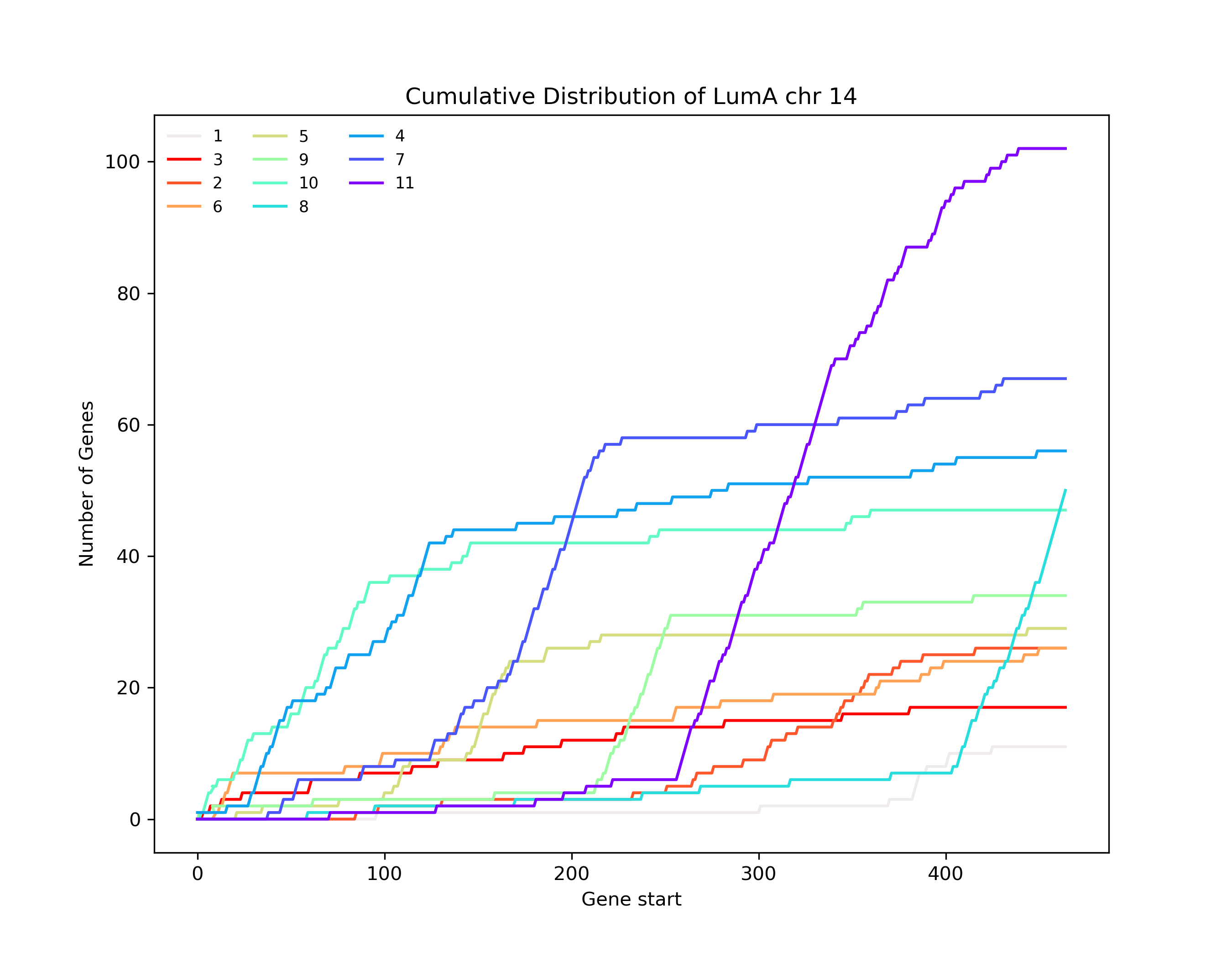

Supplement: Supplementary Material S14 — Clusters by chromosome for the five phenotypes, obtained by eigenvalue decomposition and k-medoids method. The figures are depicted as in the manuscript. Additionally, this material contains files for clusters including the name of the gene, the cluster that the gene belong to, the assignment cost function value, the chromosome location of the gene, and the gene start position of said gene. [file DataSheet_14.zip › SuppMat12/chr14/LumA-chr14-gstart-cum.png]

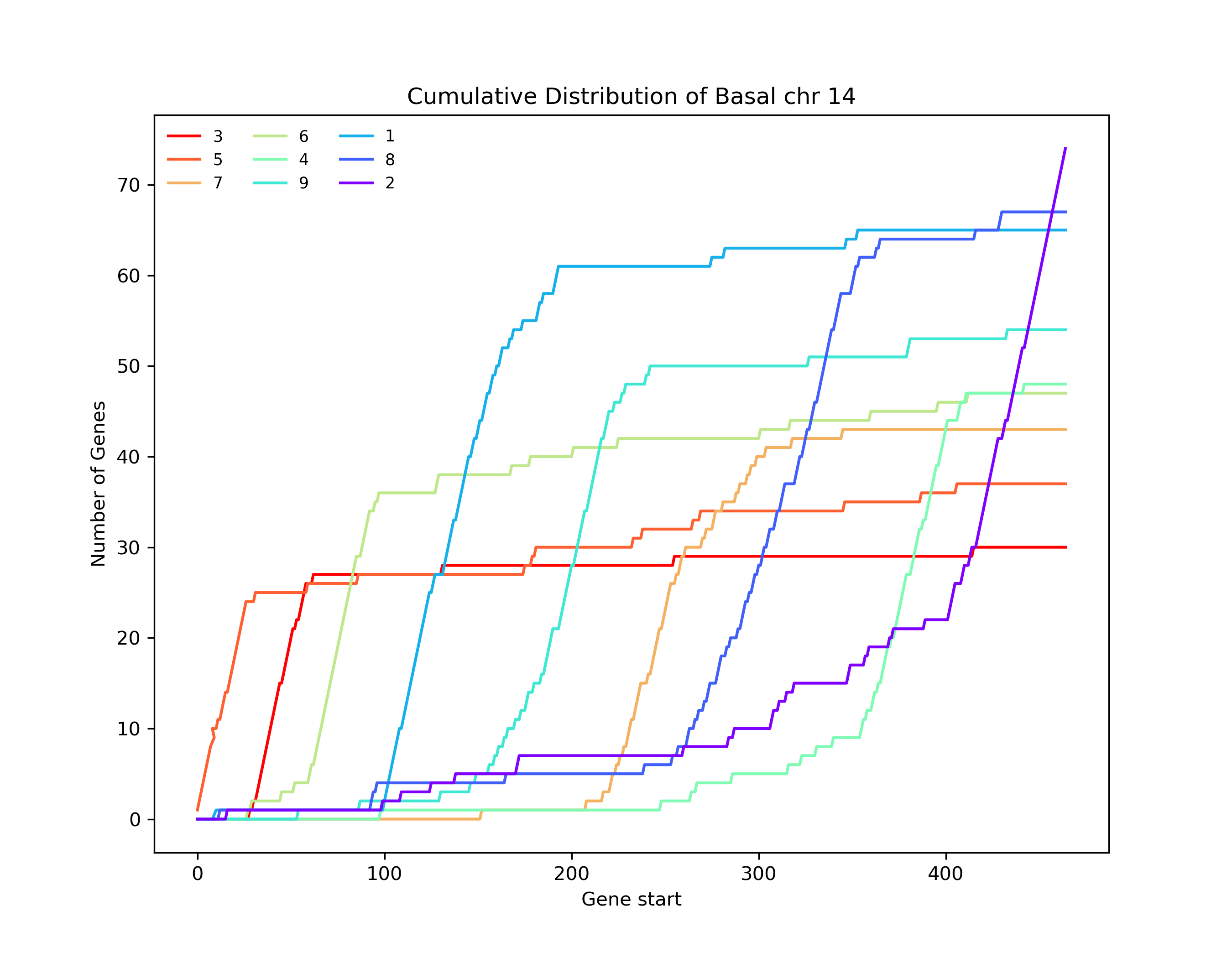

Supplement: Supplementary Material S14 — Clusters by chromosome for the five phenotypes, obtained by eigenvalue decomposition and k-medoids method. The figures are depicted as in the manuscript. Additionally, this material contains files for clusters including the name of the gene, the cluster that the gene belong to, the assignment cost function value, the chromosome location of the gene, and the gene start position of said gene. [file DataSheet_14.zip › SuppMat12/chr14/Basal-chr14-gstart-cum.png]

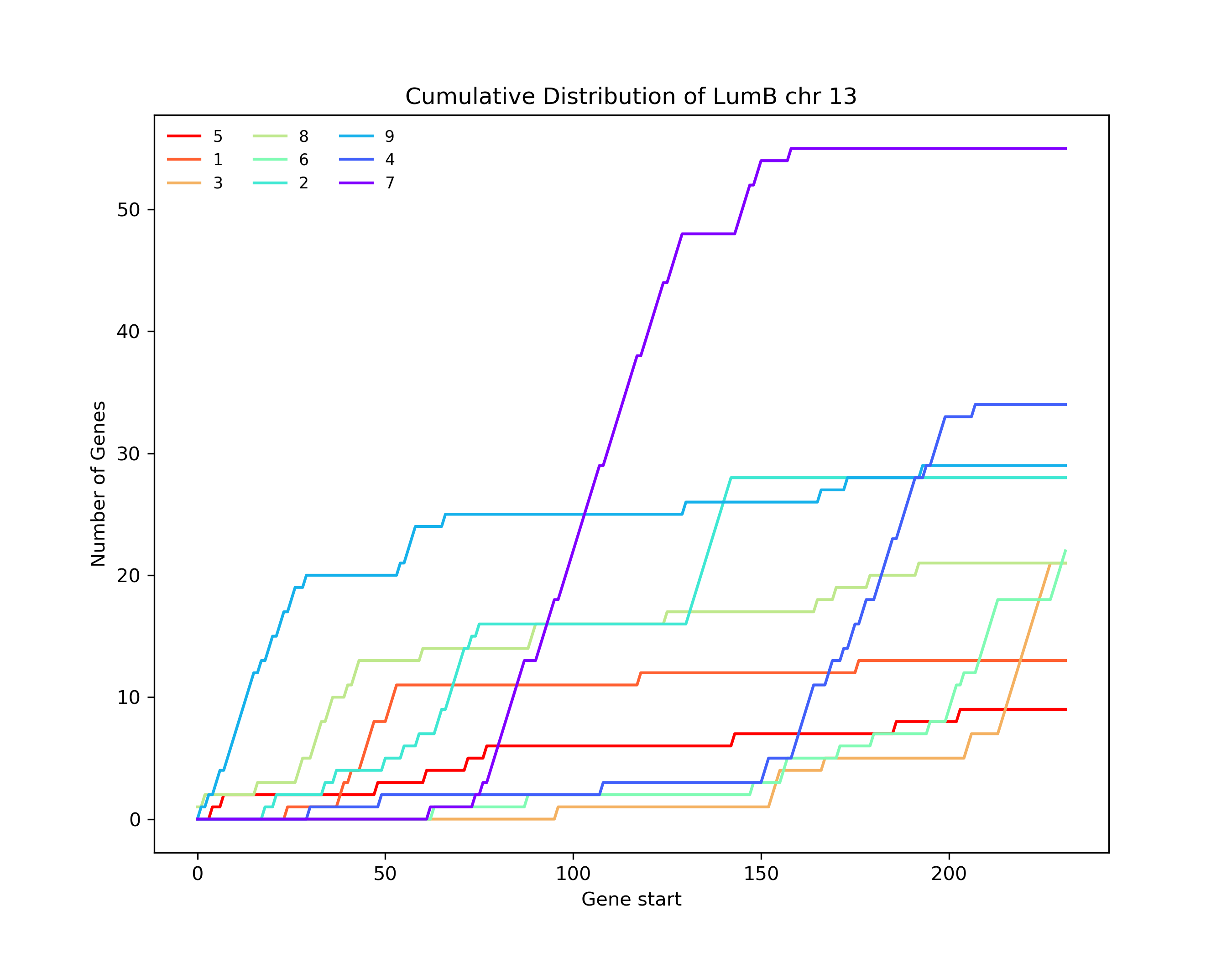

Supplement: Supplementary Material S14 — Clusters by chromosome for the five phenotypes, obtained by eigenvalue decomposition and k-medoids method. The figures are depicted as in the manuscript. Additionally, this material contains files for clusters including the name of the gene, the cluster that the gene belong to, the assignment cost function value, the chromosome location of the gene, and the gene start position of said gene. [file DataSheet_14.zip › SuppMat12/chr13/LumB-chr13-gstart-cum.png]

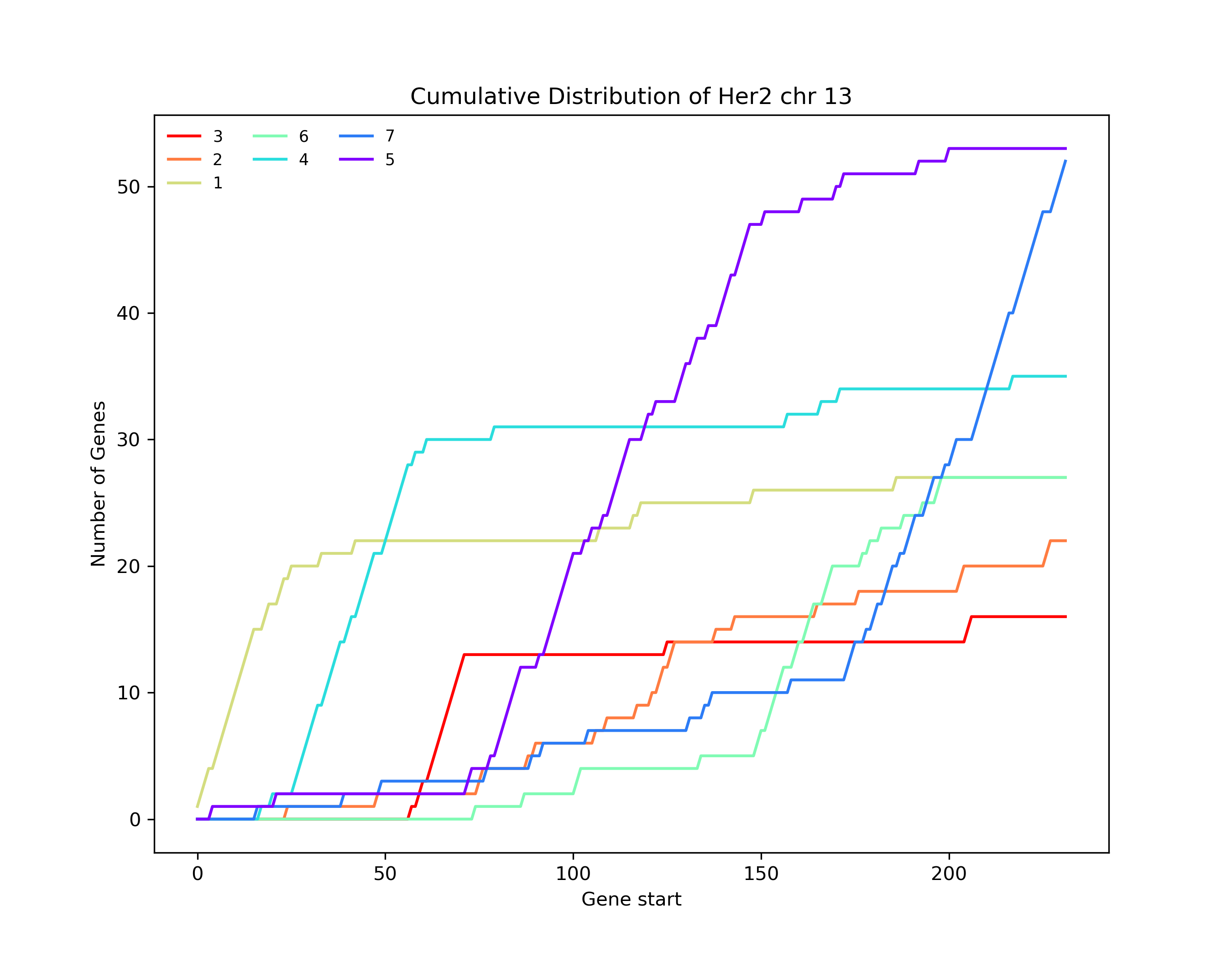

Supplement: Supplementary Material S14 — Clusters by chromosome for the five phenotypes, obtained by eigenvalue decomposition and k-medoids method. The figures are depicted as in the manuscript. Additionally, this material contains files for clusters including the name of the gene, the cluster that the gene belong to, the assignment cost function value, the chromosome location of the gene, and the gene start position of said gene. [file DataSheet_14.zip › SuppMat12/chr13/Her2-chr13-gstart-cum.png]

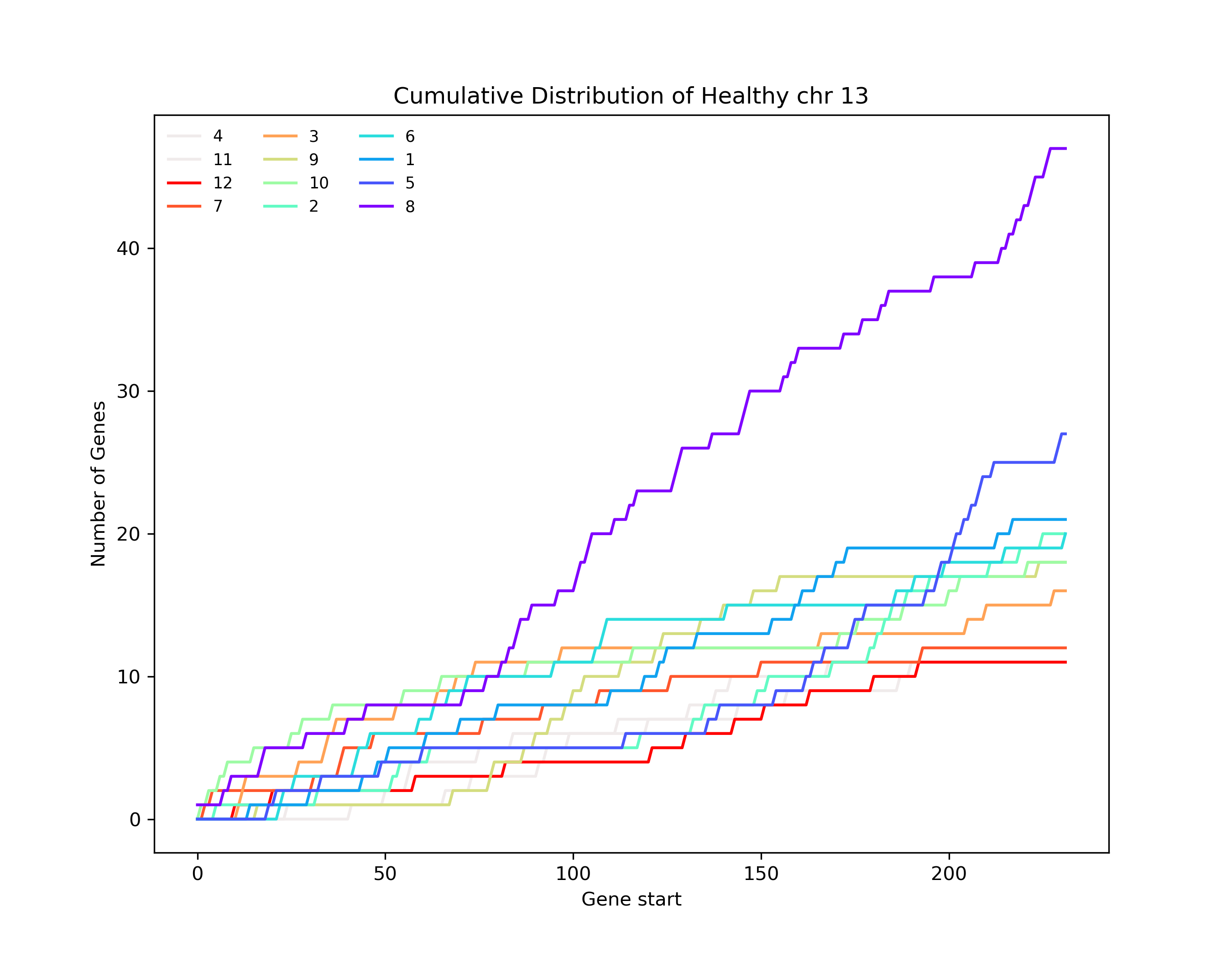

Supplement: Supplementary Material S14 — Clusters by chromosome for the five phenotypes, obtained by eigenvalue decomposition and k-medoids method. The figures are depicted as in the manuscript. Additionally, this material contains files for clusters including the name of the gene, the cluster that the gene belong to, the assignment cost function value, the chromosome location of the gene, and the gene start position of said gene. [file DataSheet_14.zip › SuppMat12/chr13/Healthy-chr13-gstart-cum.png]

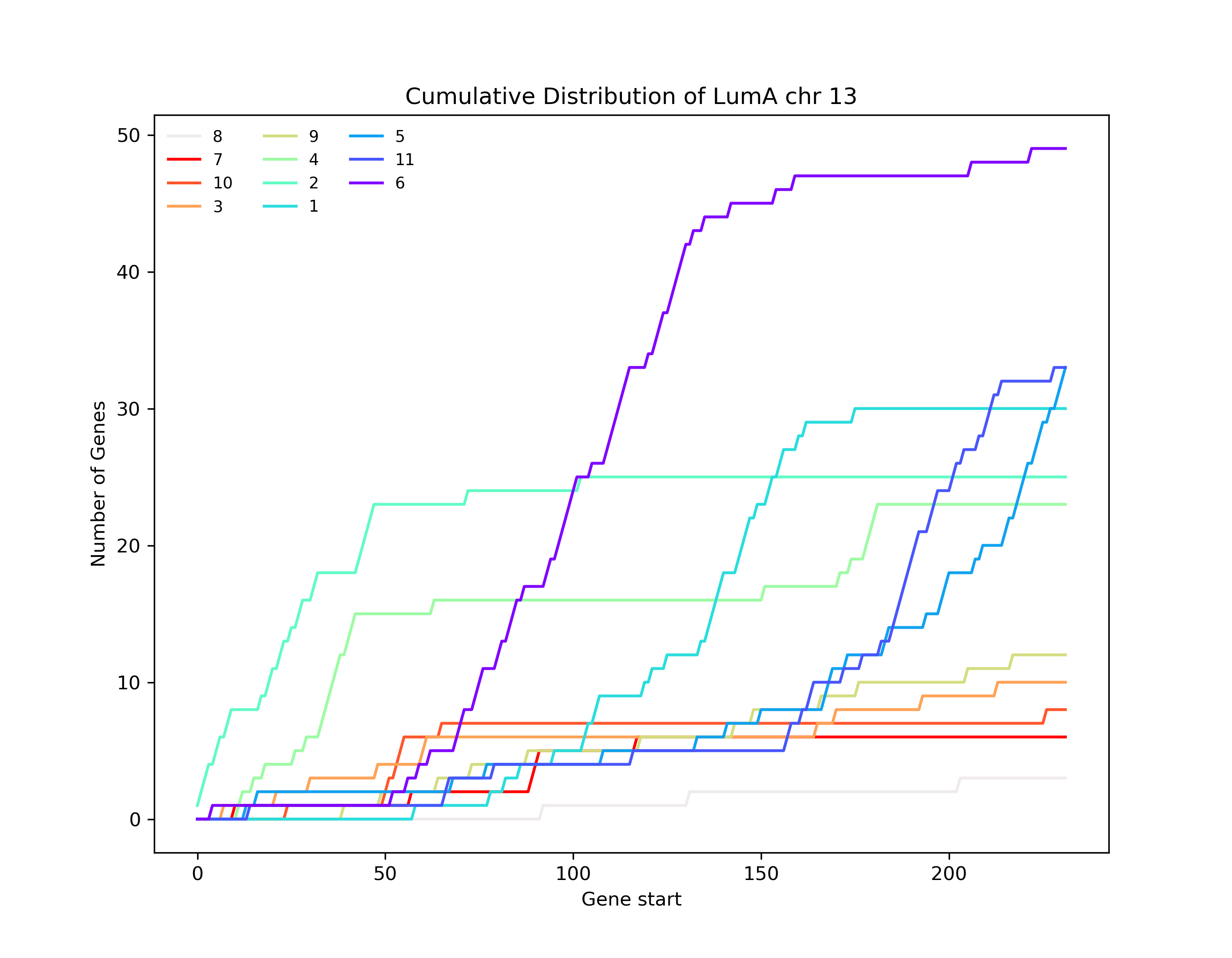

Supplement: Supplementary Material S14 — Clusters by chromosome for the five phenotypes, obtained by eigenvalue decomposition and k-medoids method. The figures are depicted as in the manuscript. Additionally, this material contains files for clusters including the name of the gene, the cluster that the gene belong to, the assignment cost function value, the chromosome location of the gene, and the gene start position of said gene. [file DataSheet_14.zip › SuppMat12/chr13/LumA-chr13-gstart-cum.png]

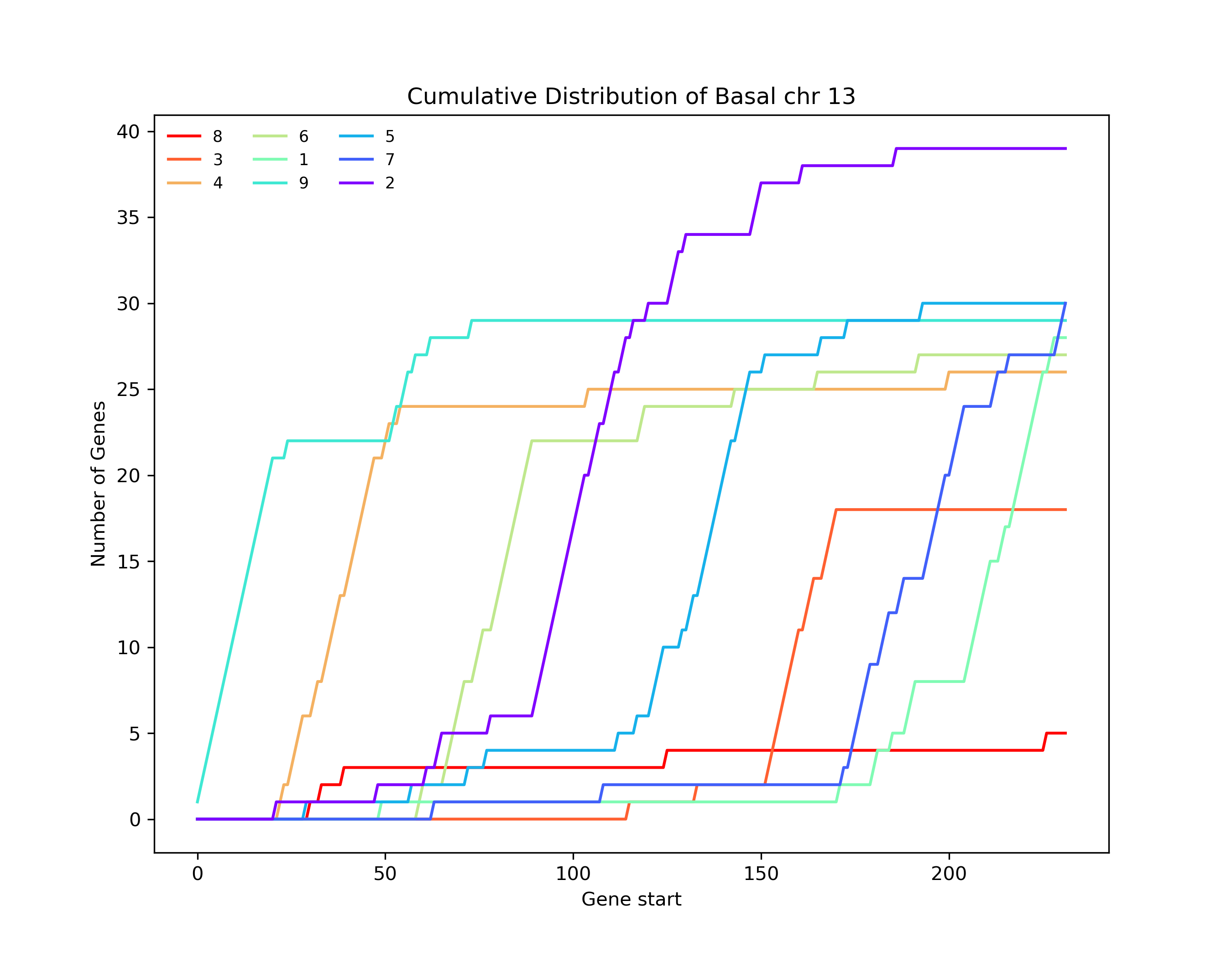

Supplement: Supplementary Material S14 — Clusters by chromosome for the five phenotypes, obtained by eigenvalue decomposition and k-medoids method. The figures are depicted as in the manuscript. Additionally, this material contains files for clusters including the name of the gene, the cluster that the gene belong to, the assignment cost function value, the chromosome location of the gene, and the gene start position of said gene. [file DataSheet_14.zip › SuppMat12/chr13/Basal-chr13-gstart-cum.png]

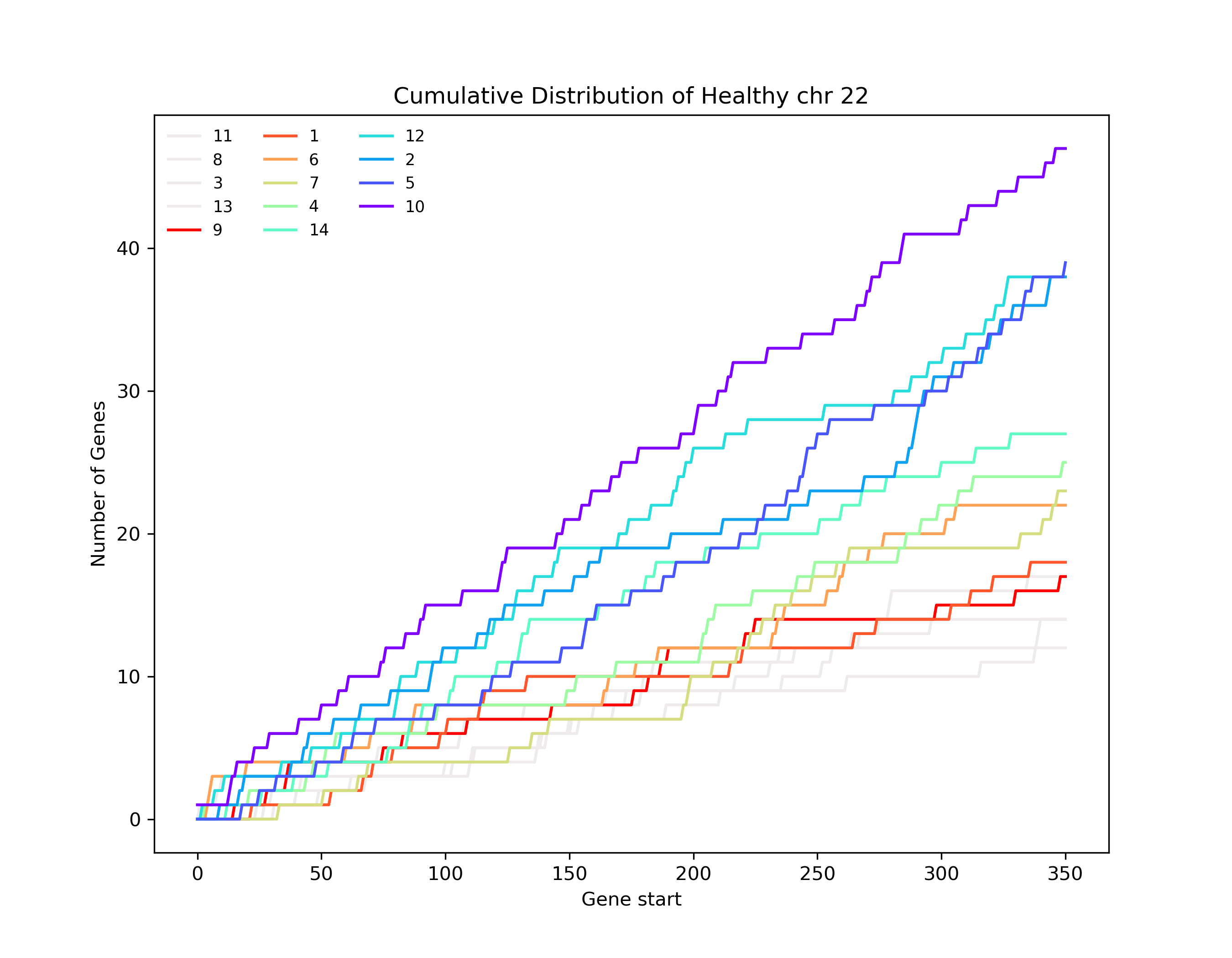

Supplement: Supplementary Material S14 — Clusters by chromosome for the five phenotypes, obtained by eigenvalue decomposition and k-medoids method. The figures are depicted as in the manuscript. Additionally, this material contains files for clusters including the name of the gene, the cluster that the gene belong to, the assignment cost function value, the chromosome location of the gene, and the gene start position of said gene. [file DataSheet_14.zip › SuppMat12/chr22/Healthy-chr22-gstart-cum.png]

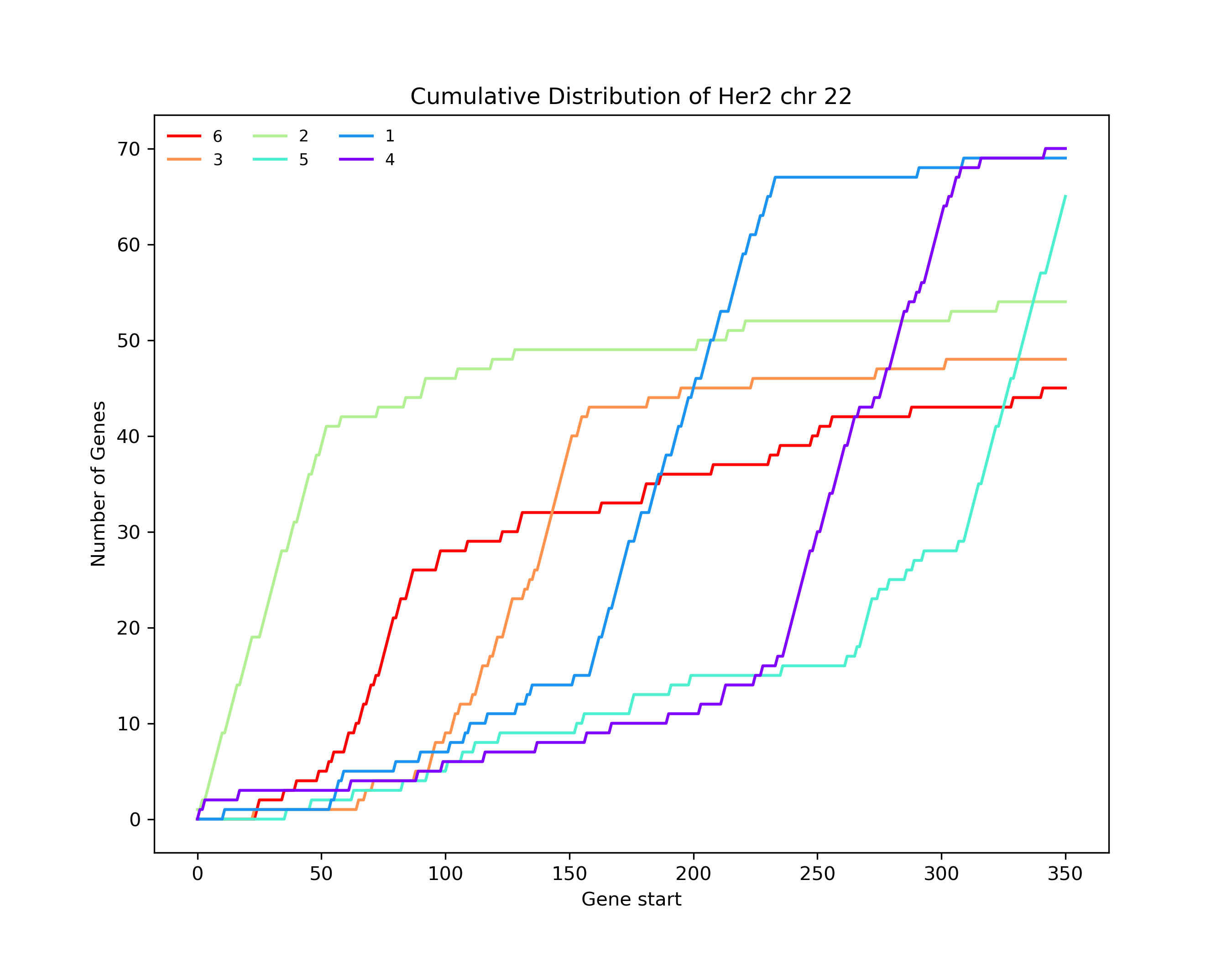

Supplement: Supplementary Material S14 — Clusters by chromosome for the five phenotypes, obtained by eigenvalue decomposition and k-medoids method. The figures are depicted as in the manuscript. Additionally, this material contains files for clusters including the name of the gene, the cluster that the gene belong to, the assignment cost function value, the chromosome location of the gene, and the gene start position of said gene. [file DataSheet_14.zip › SuppMat12/chr22/Her2-chr22-gstart-cum.png]

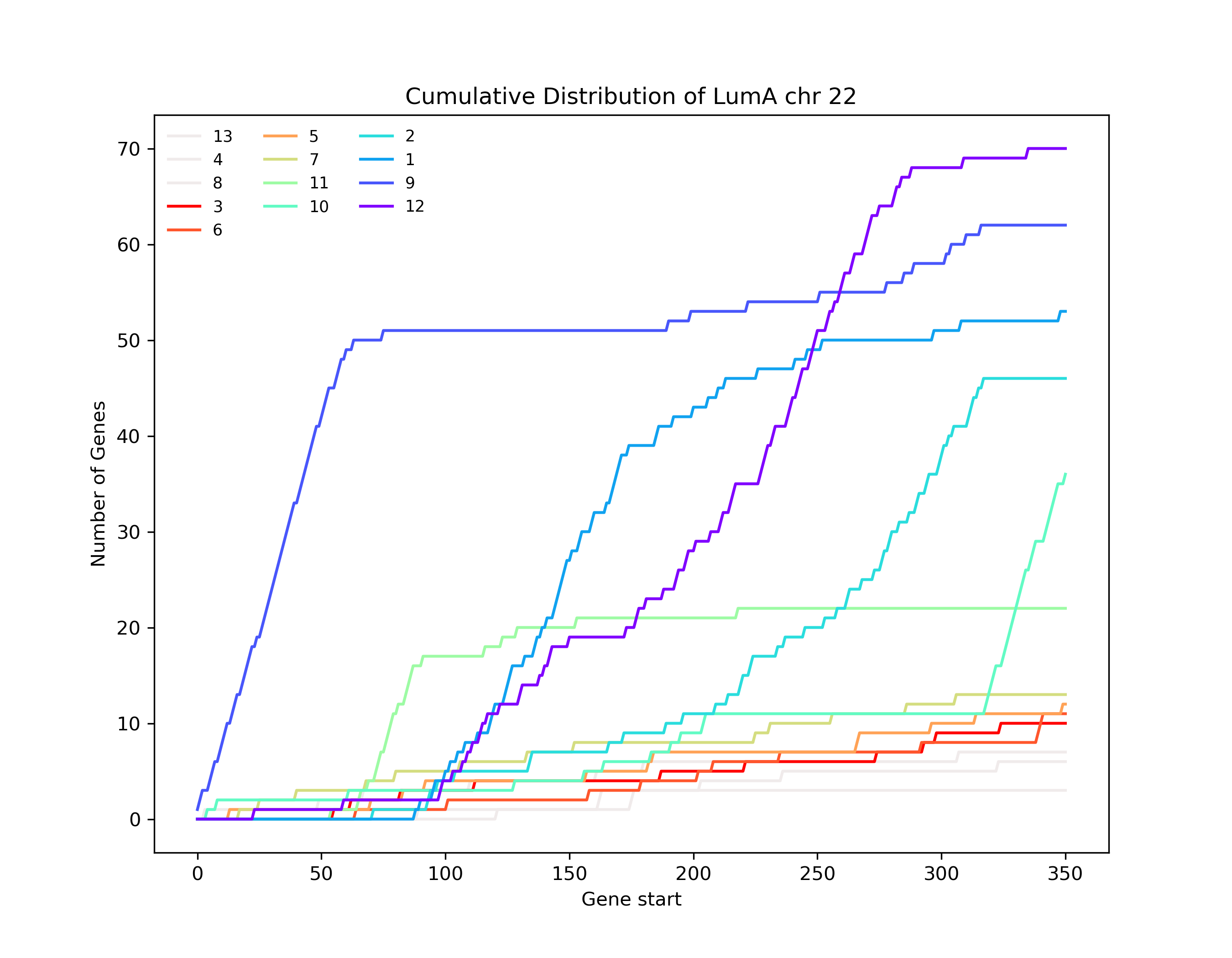

Supplement: Supplementary Material S14 — Clusters by chromosome for the five phenotypes, obtained by eigenvalue decomposition and k-medoids method. The figures are depicted as in the manuscript. Additionally, this material contains files for clusters including the name of the gene, the cluster that the gene belong to, the assignment cost function value, the chromosome location of the gene, and the gene start position of said gene. [file DataSheet_14.zip › SuppMat12/chr22/LumA-chr22-gstart-cum.png]

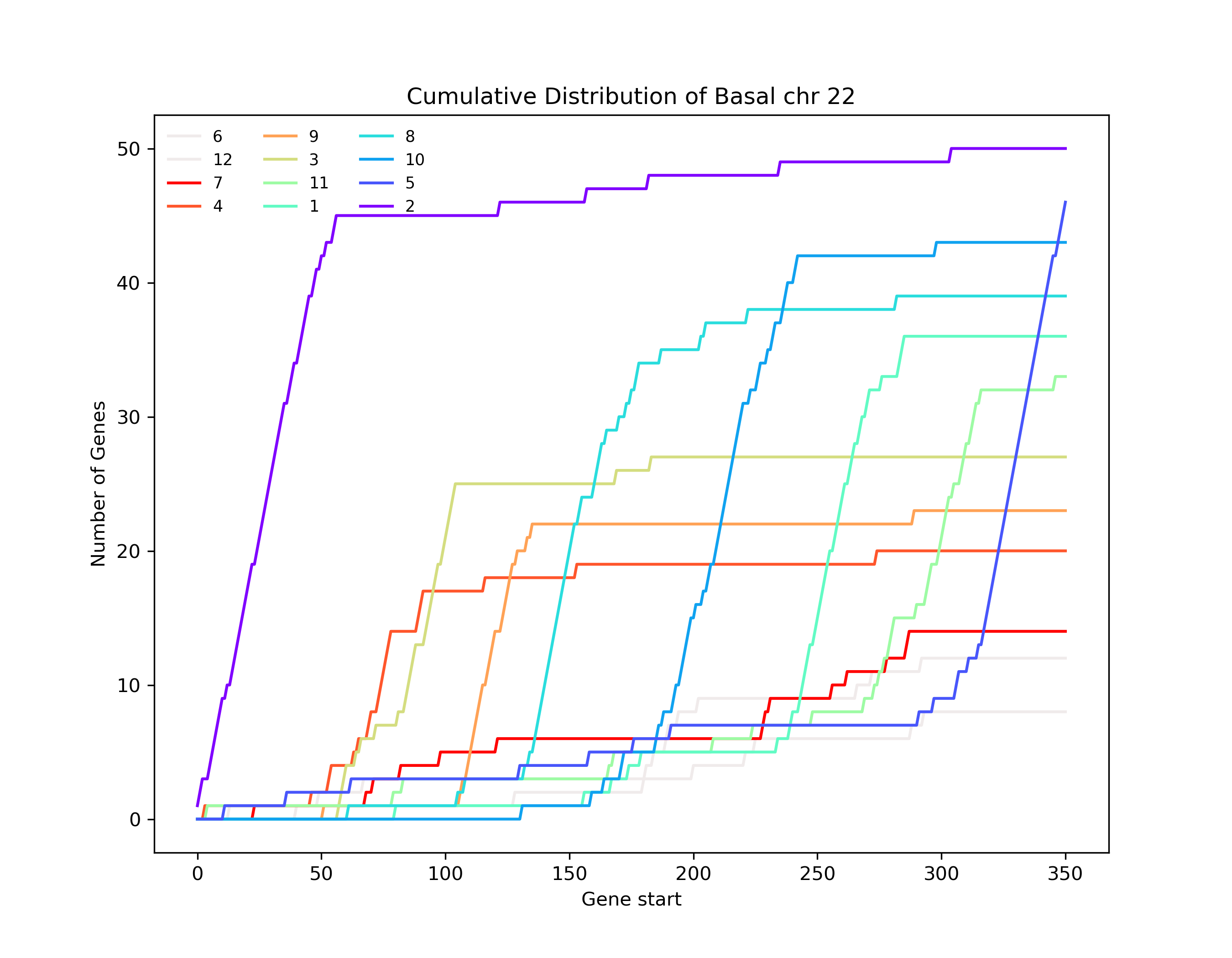

Supplement: Supplementary Material S14 — Clusters by chromosome for the five phenotypes, obtained by eigenvalue decomposition and k-medoids method. The figures are depicted as in the manuscript. Additionally, this material contains files for clusters including the name of the gene, the cluster that the gene belong to, the assignment cost function value, the chromosome location of the gene, and the gene start position of said gene. [file DataSheet_14.zip › SuppMat12/chr22/Basal-chr22-gstart-cum.png]

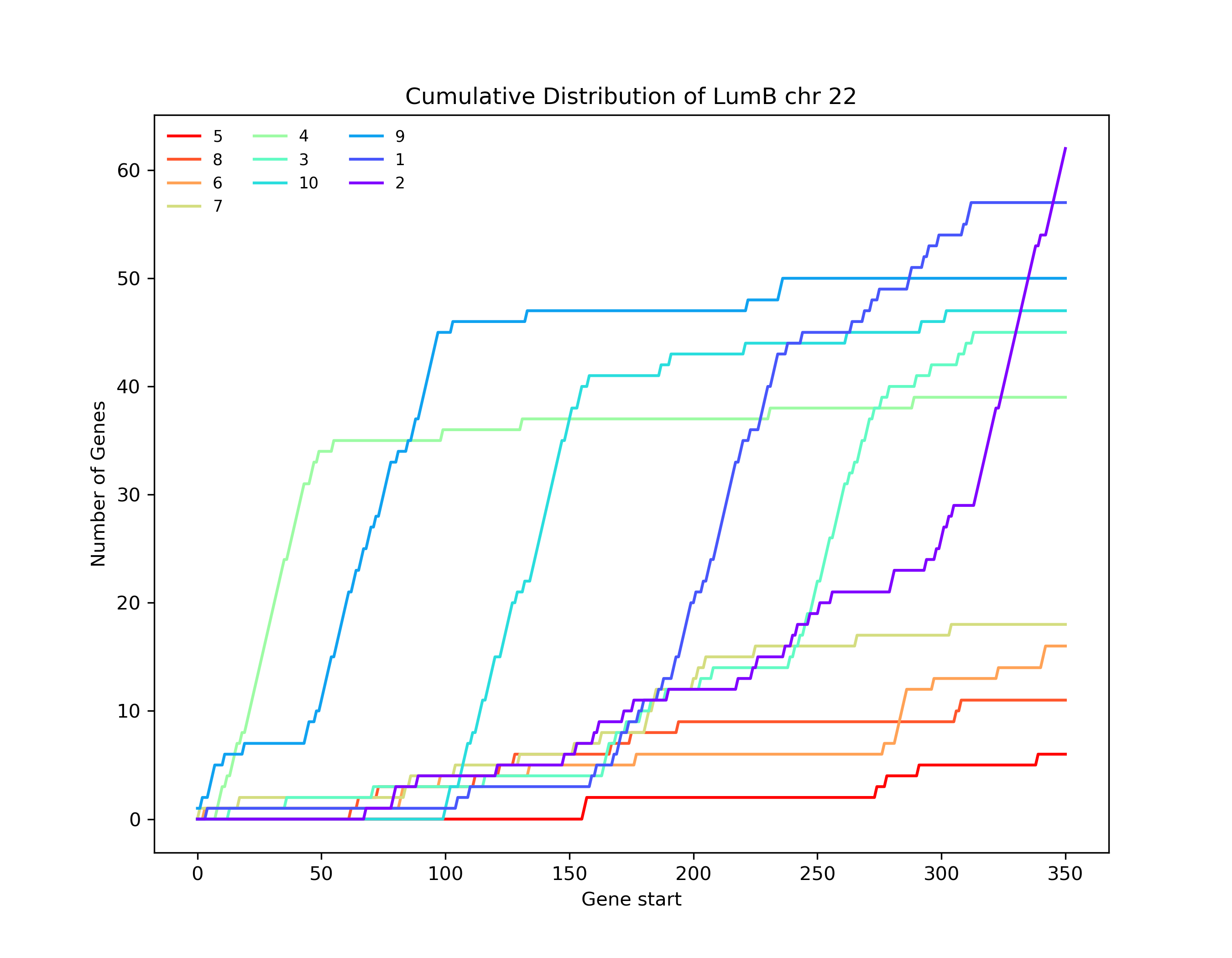

Supplement: Supplementary Material S14 — Clusters by chromosome for the five phenotypes, obtained by eigenvalue decomposition and k-medoids method. The figures are depicted as in the manuscript. Additionally, this material contains files for clusters including the name of the gene, the cluster that the gene belong to, the assignment cost function value, the chromosome location of the gene, and the gene start position of said gene. [file DataSheet_14.zip › SuppMat12/chr22/LumB-chr22-gstart-cum.png]

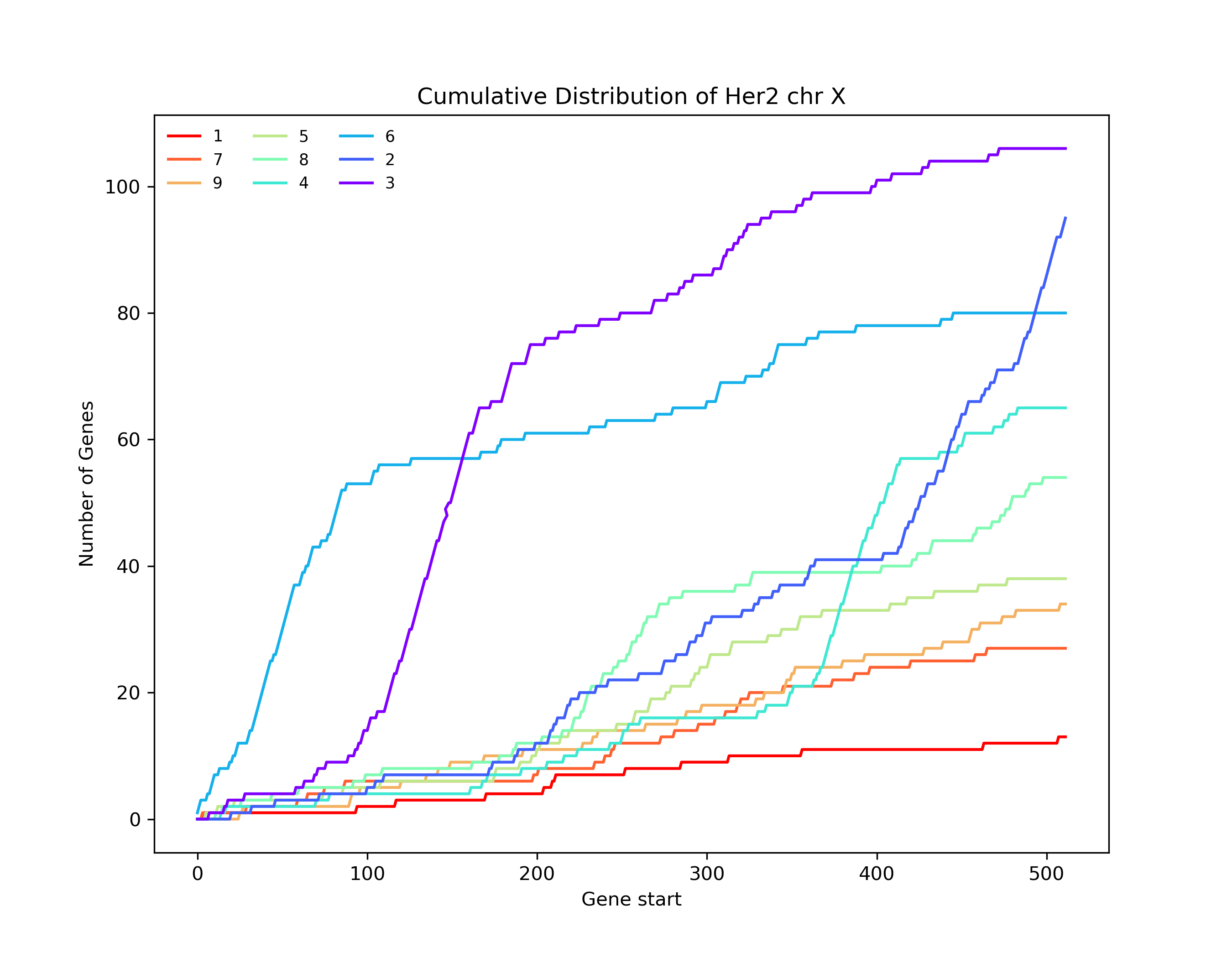

Supplement: Supplementary Material S14 — Clusters by chromosome for the five phenotypes, obtained by eigenvalue decomposition and k-medoids method. The figures are depicted as in the manuscript. Additionally, this material contains files for clusters including the name of the gene, the cluster that the gene belong to, the assignment cost function value, the chromosome location of the gene, and the gene start position of said gene. [file DataSheet_14.zip › SuppMat12/chrX/Her2-chrX-gstart-cum.png]
