# Supplementary figures and images for: Gene Co-Expression in Breast Cancer: A Matter of Distance (part 5 of 5)
Source: Front Oncol. 2021 Nov 17;11:726493. doi: 10.3389/fonc.2021.726493 (PMC8636045; doi:10.3389/fonc.2021.726493)

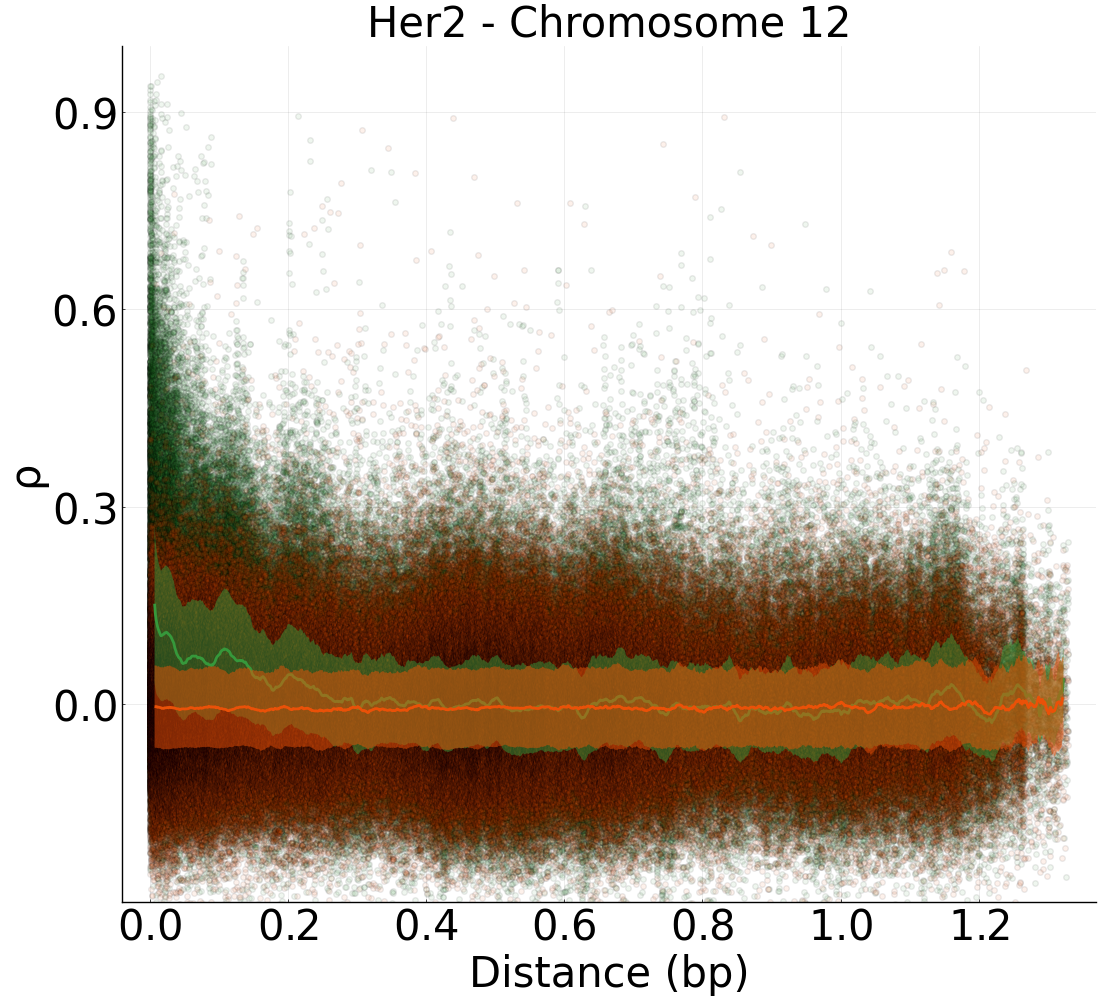

Supplement: Supplementary file 15 [file DataSheet_8.zip › SuppMat9Her/Chromosome-12-Her2.png]

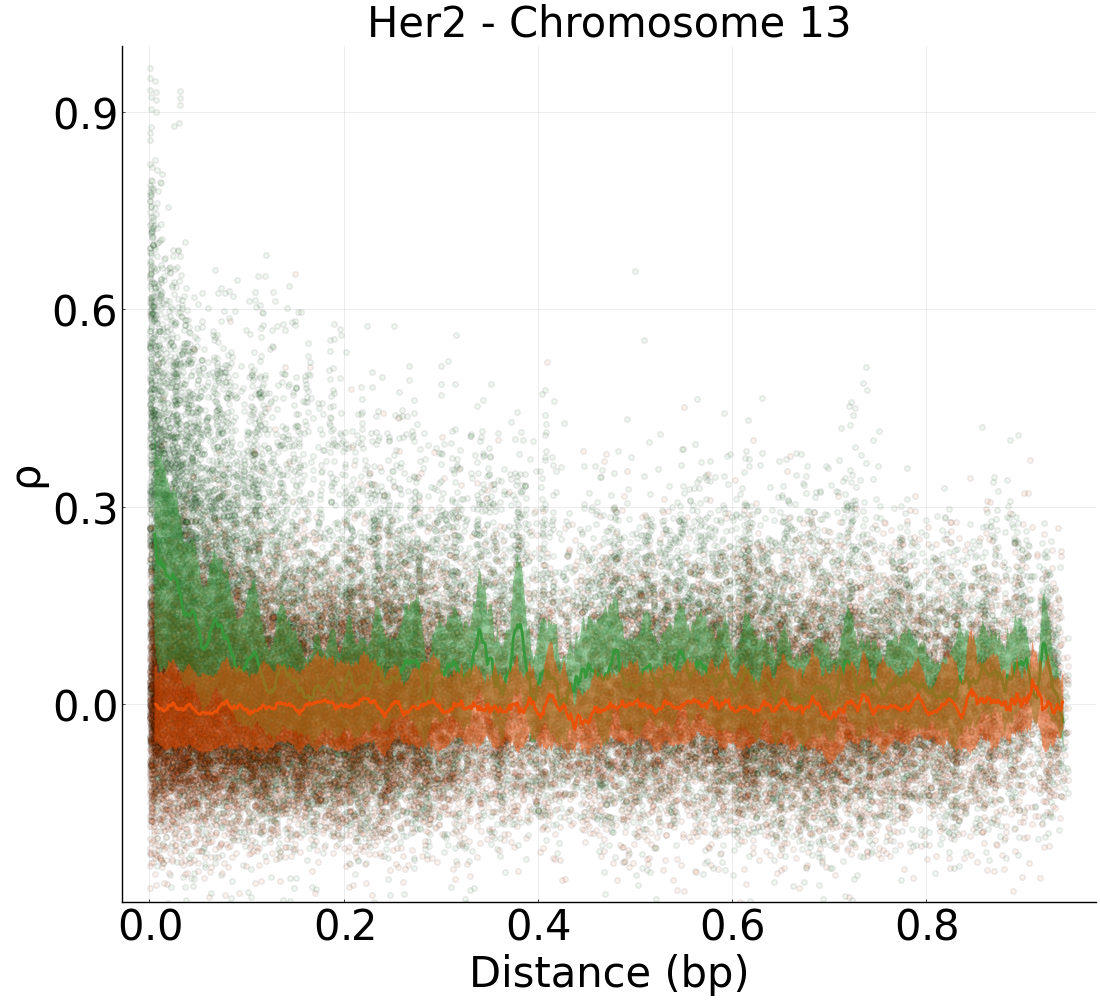

Supplement: Supplementary file 15 [file DataSheet_8.zip › SuppMat9Her/Chromosome-13-Her2.png]

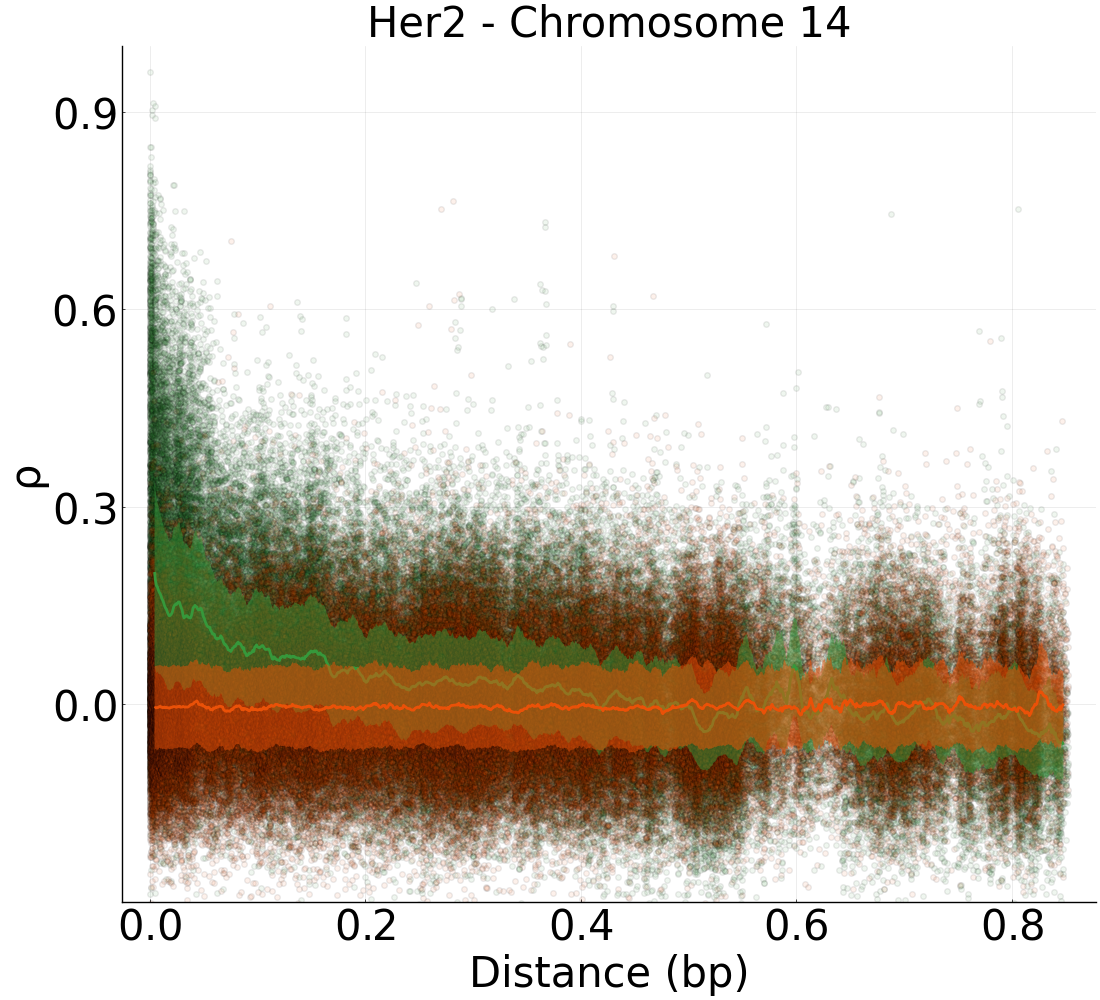

Supplement: Supplementary file 15 [file DataSheet_8.zip › SuppMat9Her/Chromosome-14-Her2.png]

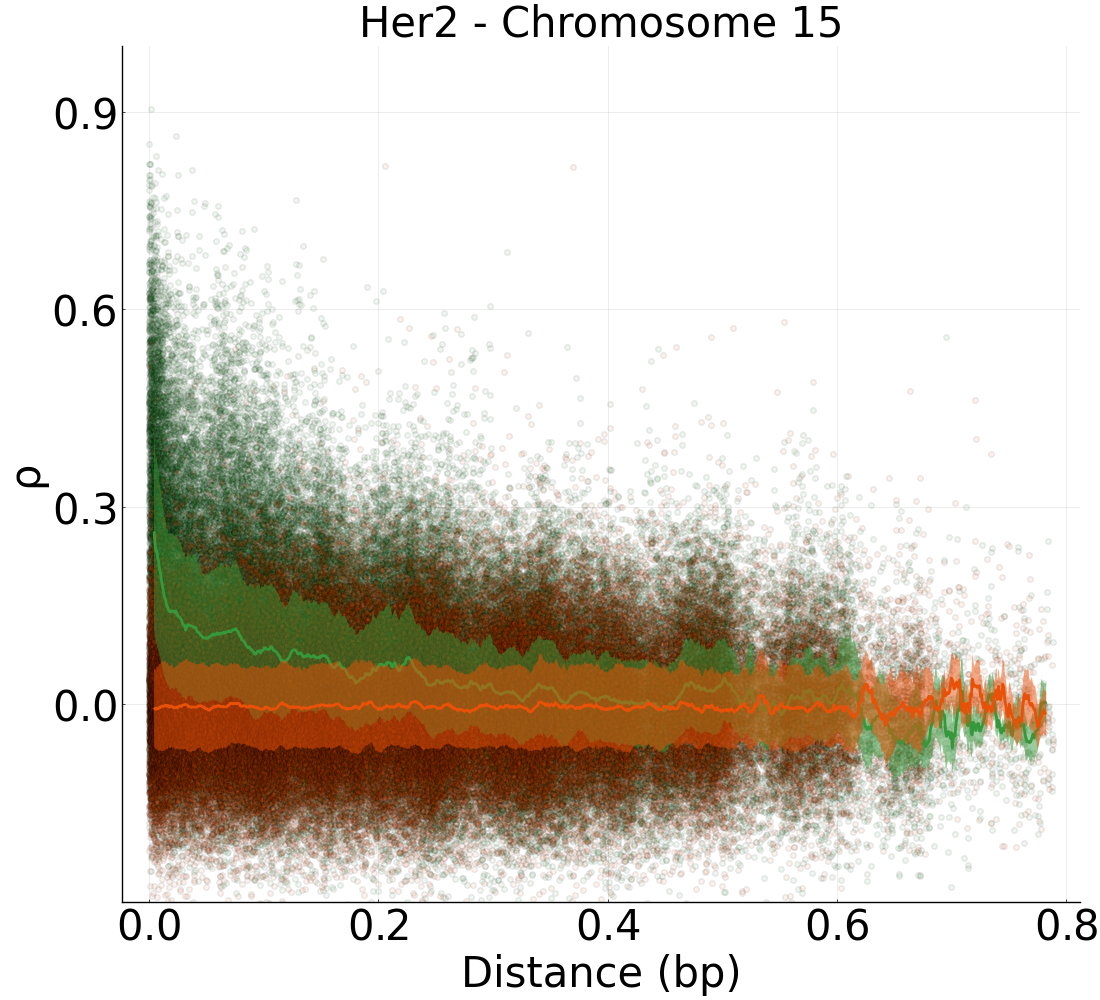

Supplement: Supplementary file 15 [file DataSheet_8.zip › SuppMat9Her/Chromosome-15-Her2.png]

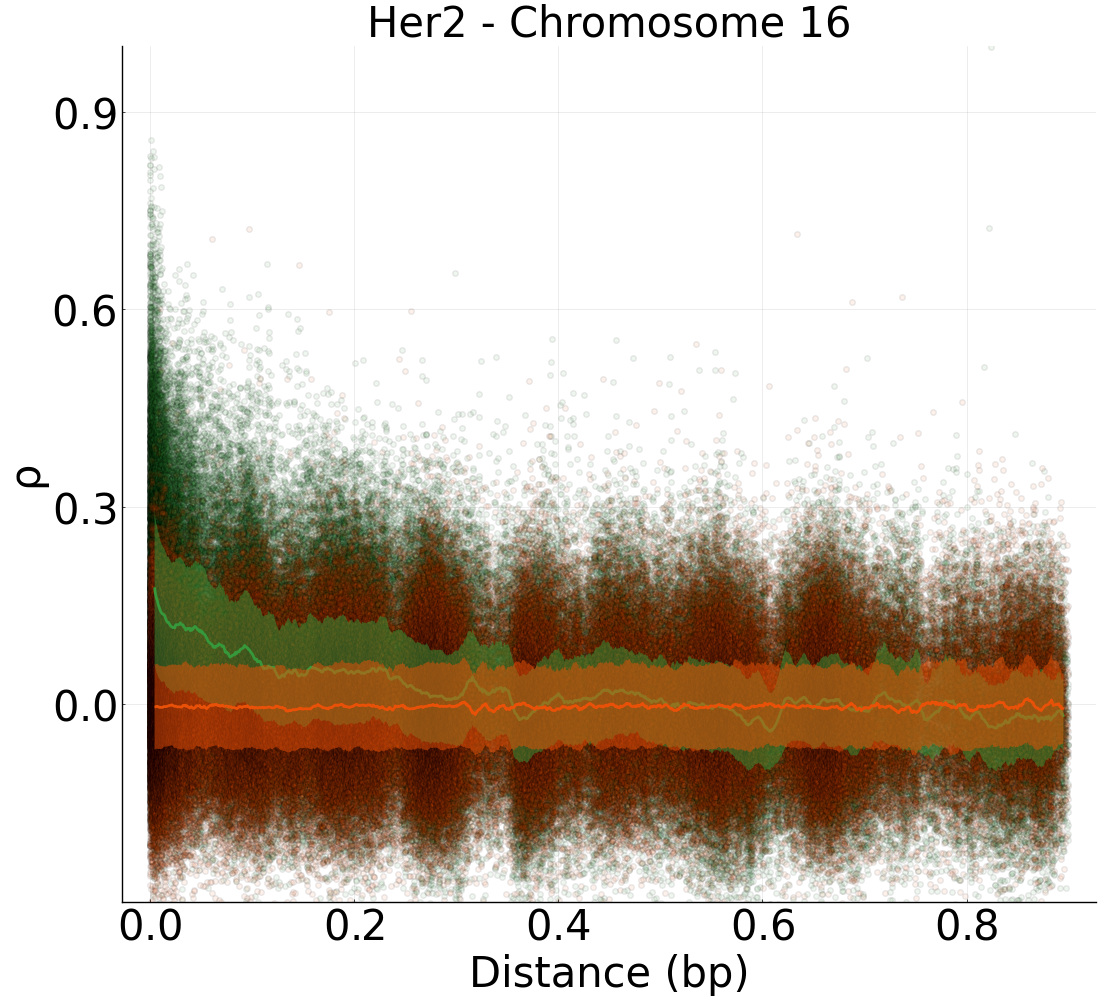

Supplement: Supplementary file 15 [file DataSheet_8.zip › SuppMat9Her/Chromosome-16-Her2.png]

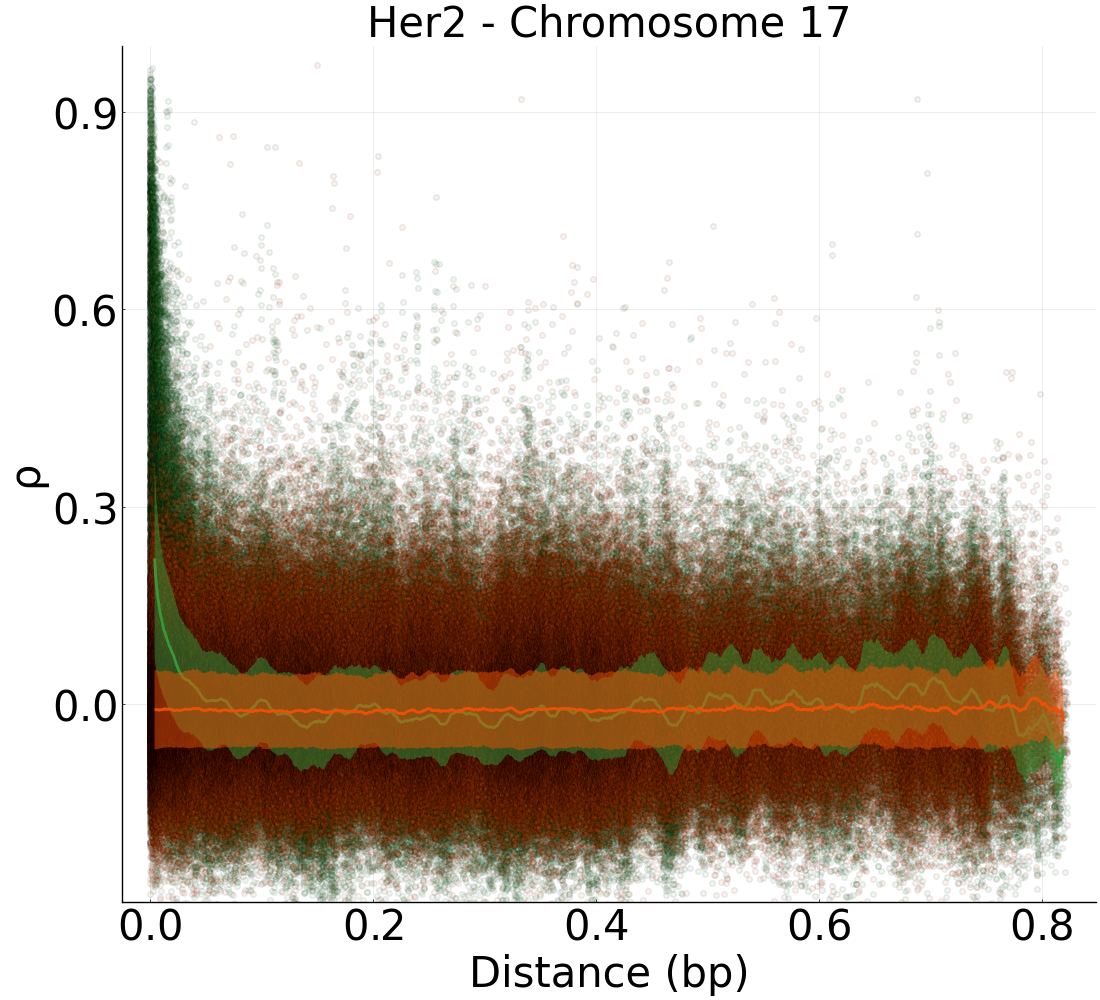

Supplement: Supplementary file 15 [file DataSheet_8.zip › SuppMat9Her/Chromosome-17-Her2.png]

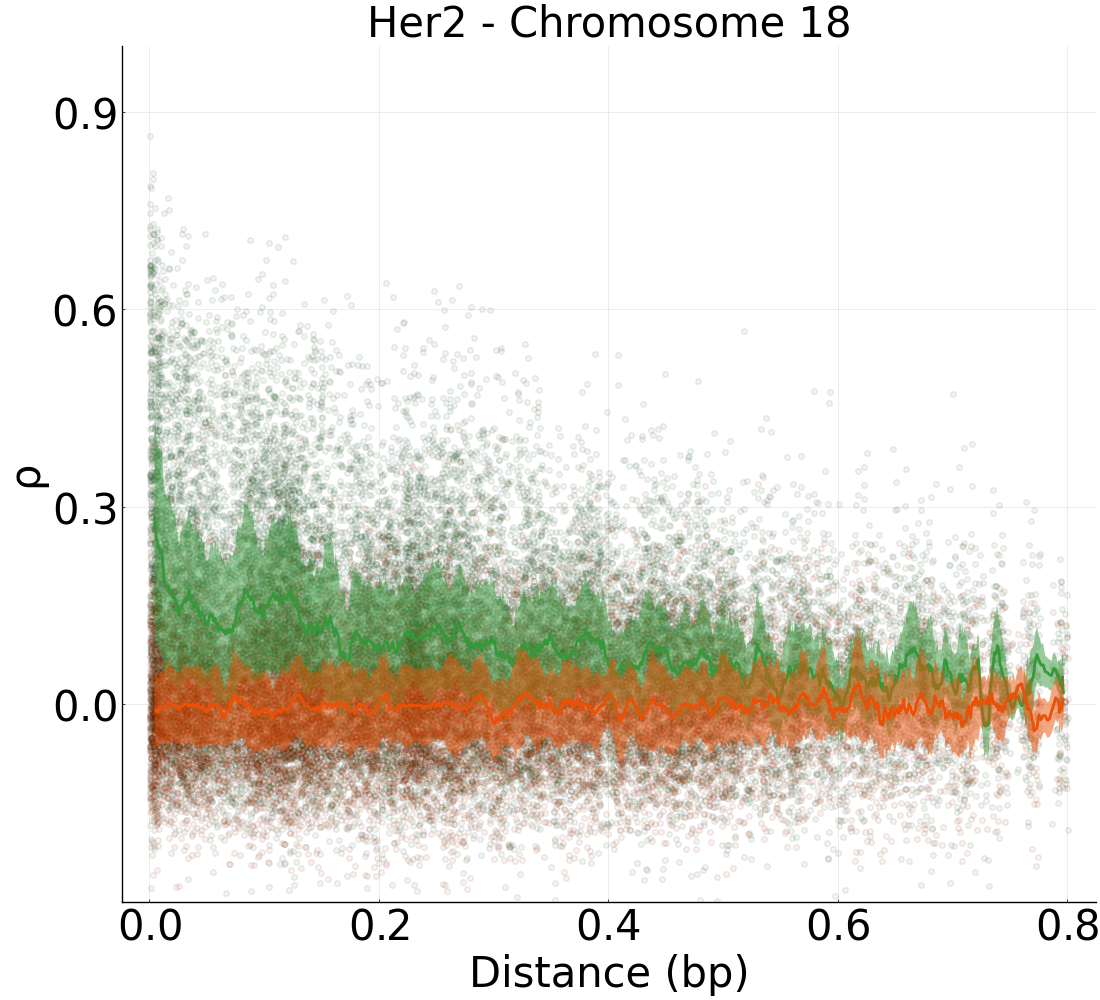

Supplement: Supplementary file 15 [file DataSheet_8.zip › SuppMat9Her/Chromosome-18-Her2.png]

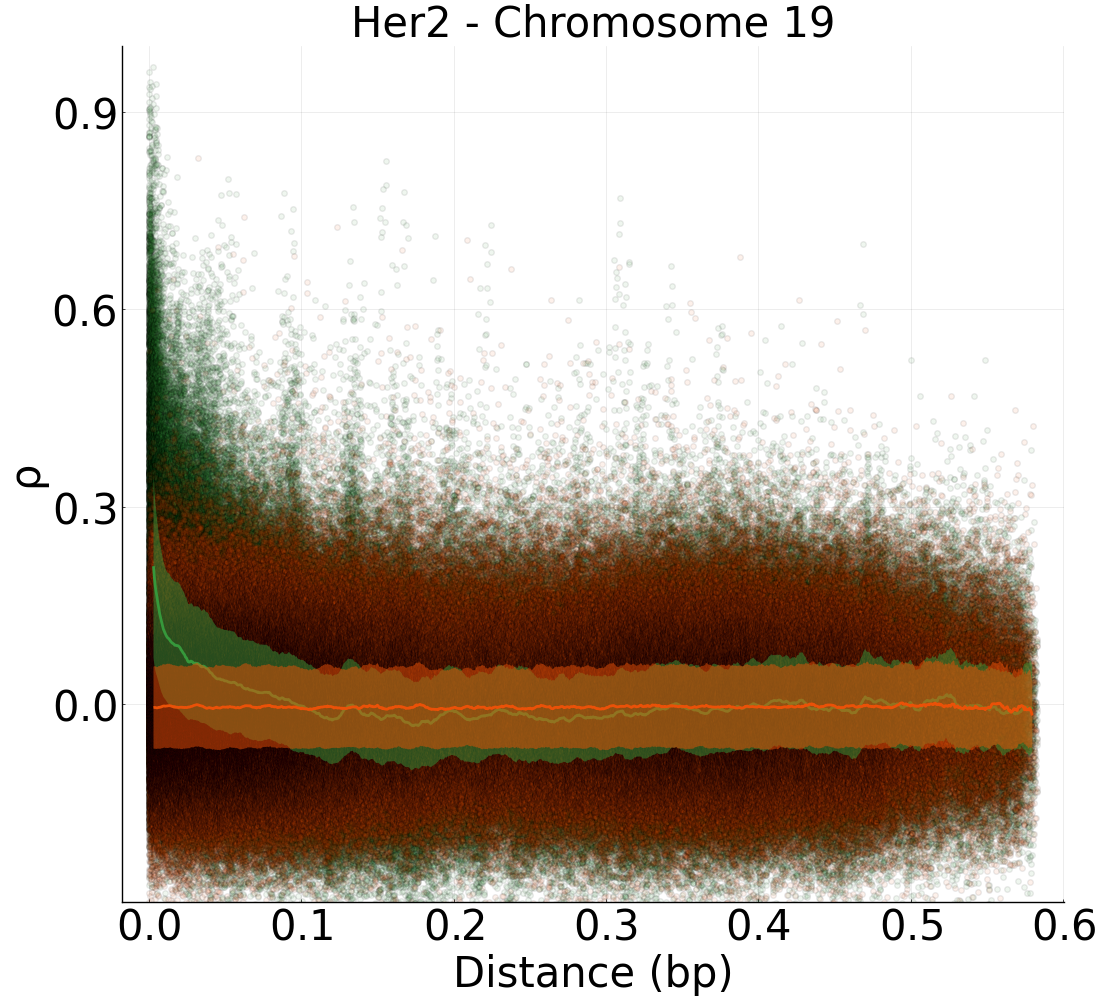

Supplement: Supplementary file 15 [file DataSheet_8.zip › SuppMat9Her/Chromosome-19-Her2.png]

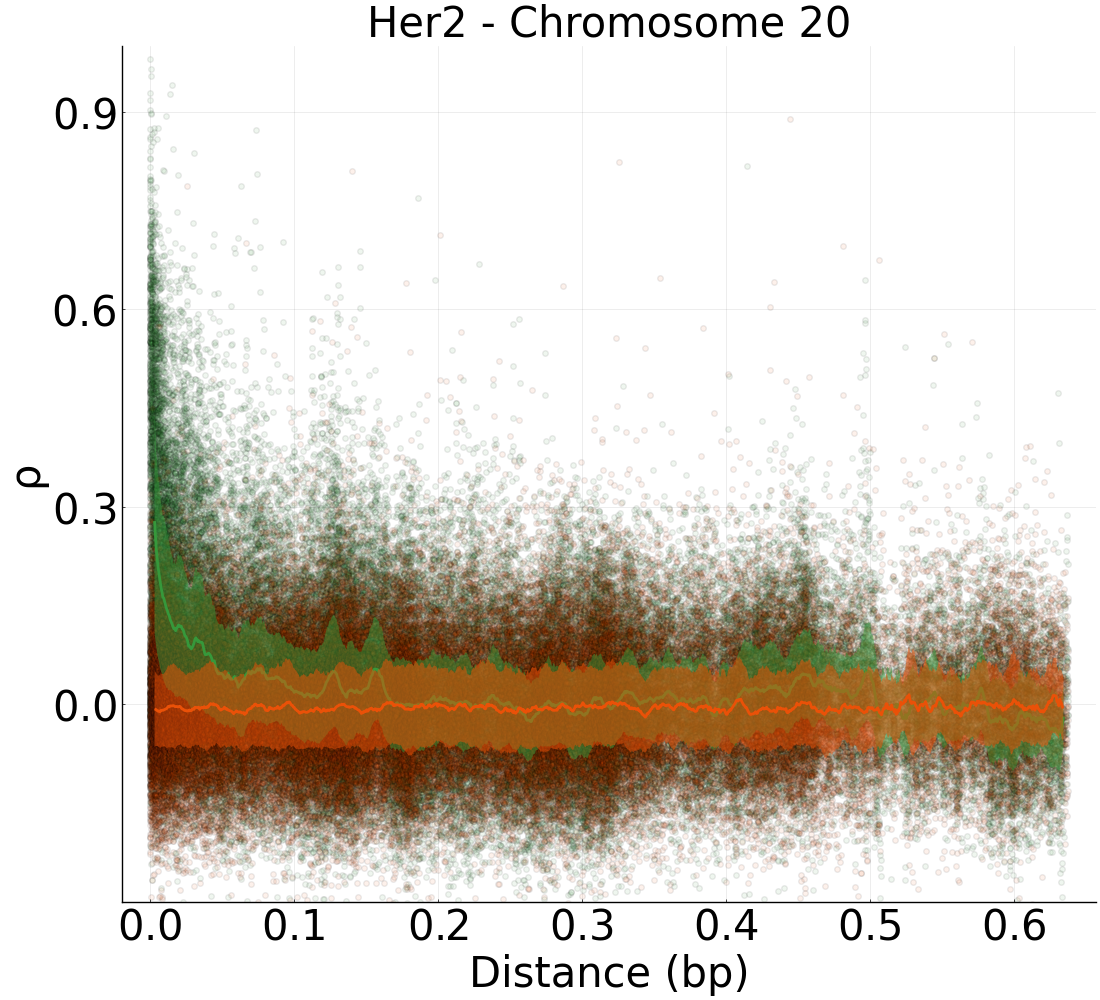

Supplement: Supplementary file 15 [file DataSheet_8.zip › SuppMat9Her/Chromosome-20-Her2.png]

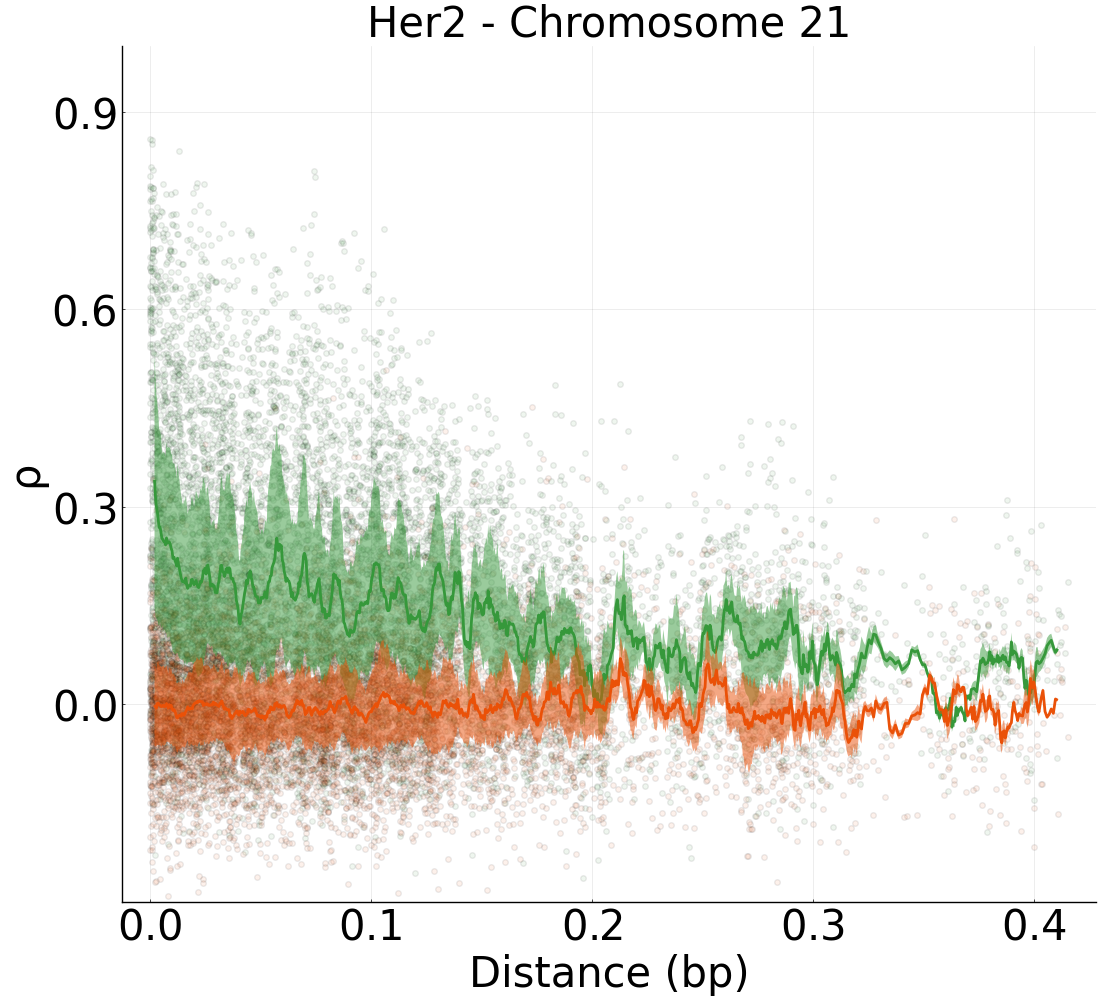

Supplement: Supplementary file 15 [file DataSheet_8.zip › SuppMat9Her/Chromosome-21-Her2.png]

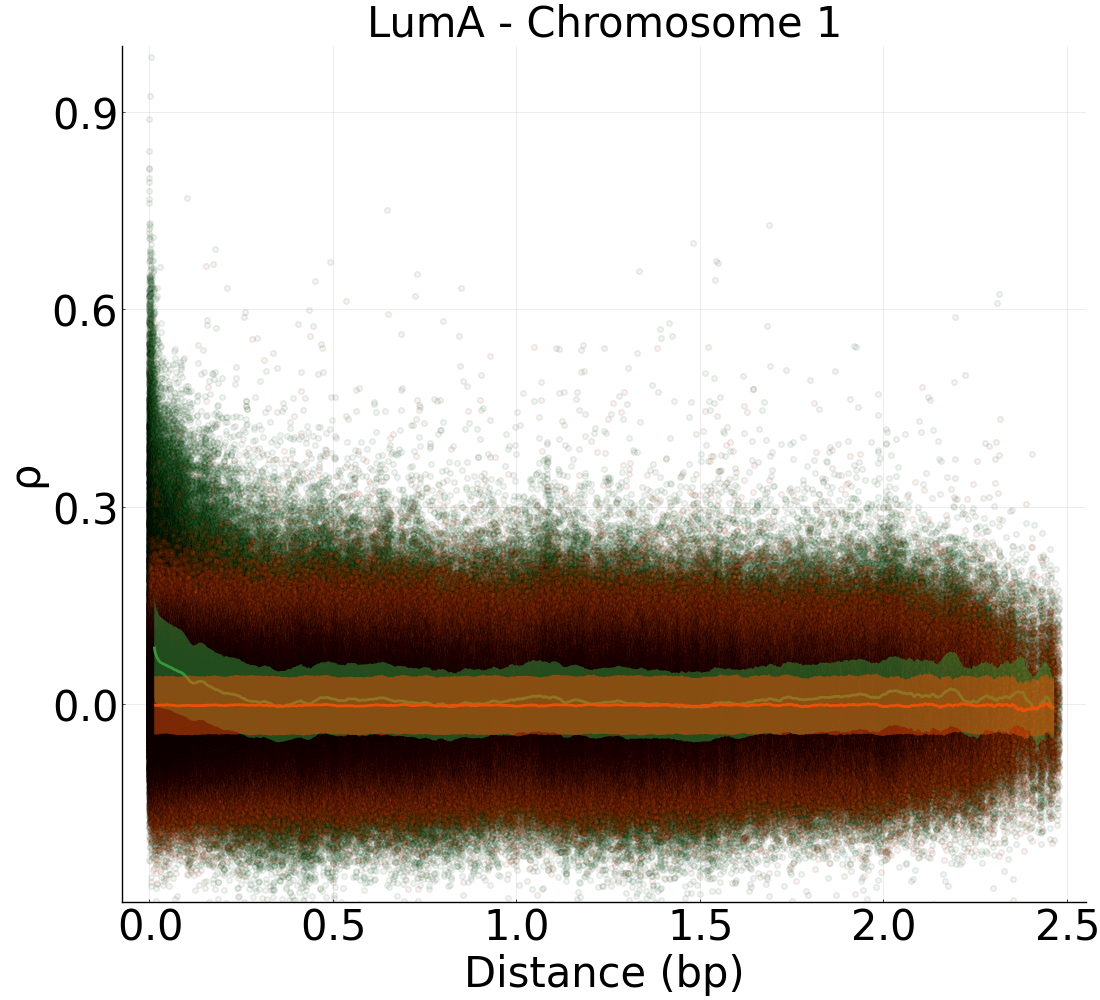

Supplement: Supplementary file 16 [file DataSheet_9.zip › SuppMat10Luma/Chromosome-1-LumA.png]

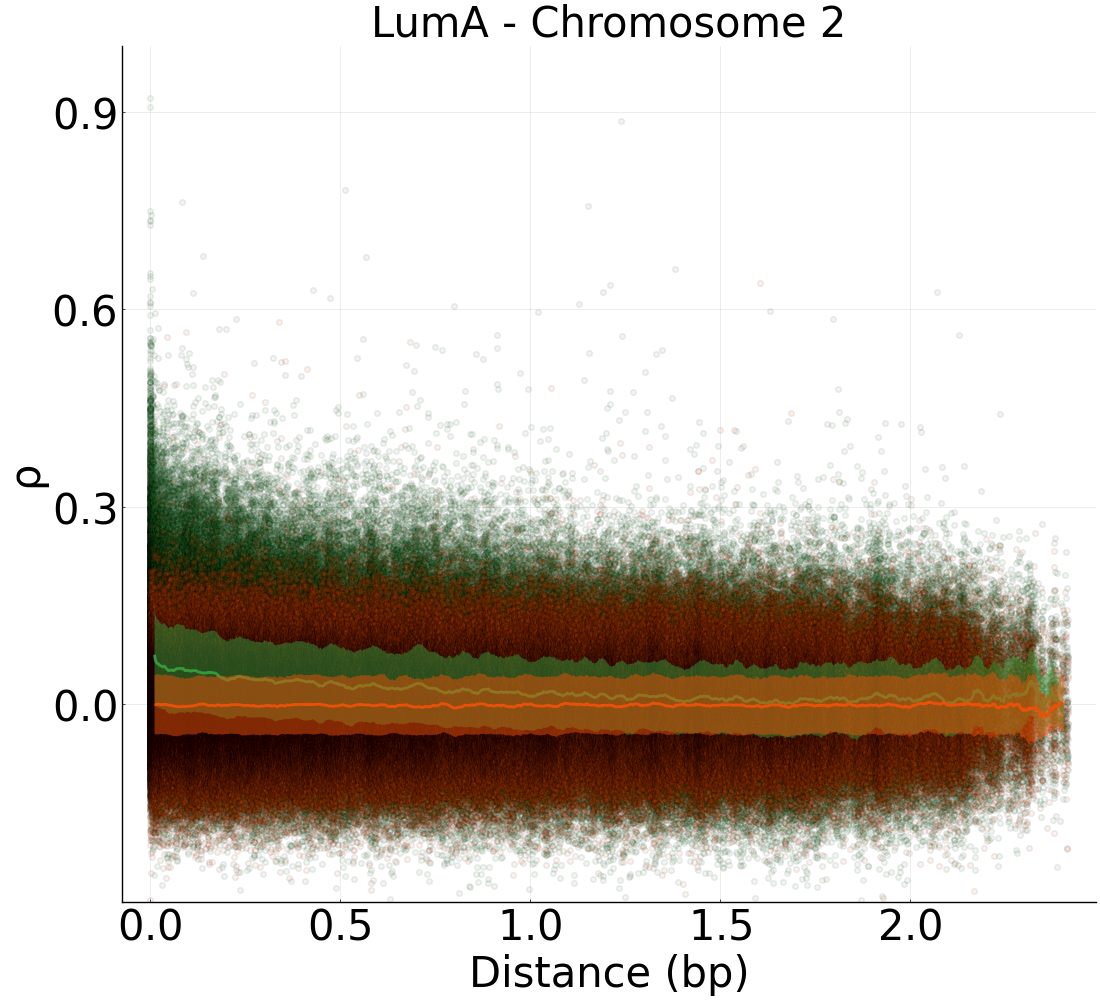

Supplement: Supplementary file 16 [file DataSheet_9.zip › SuppMat10Luma/Chromosome-2-LumA.png]

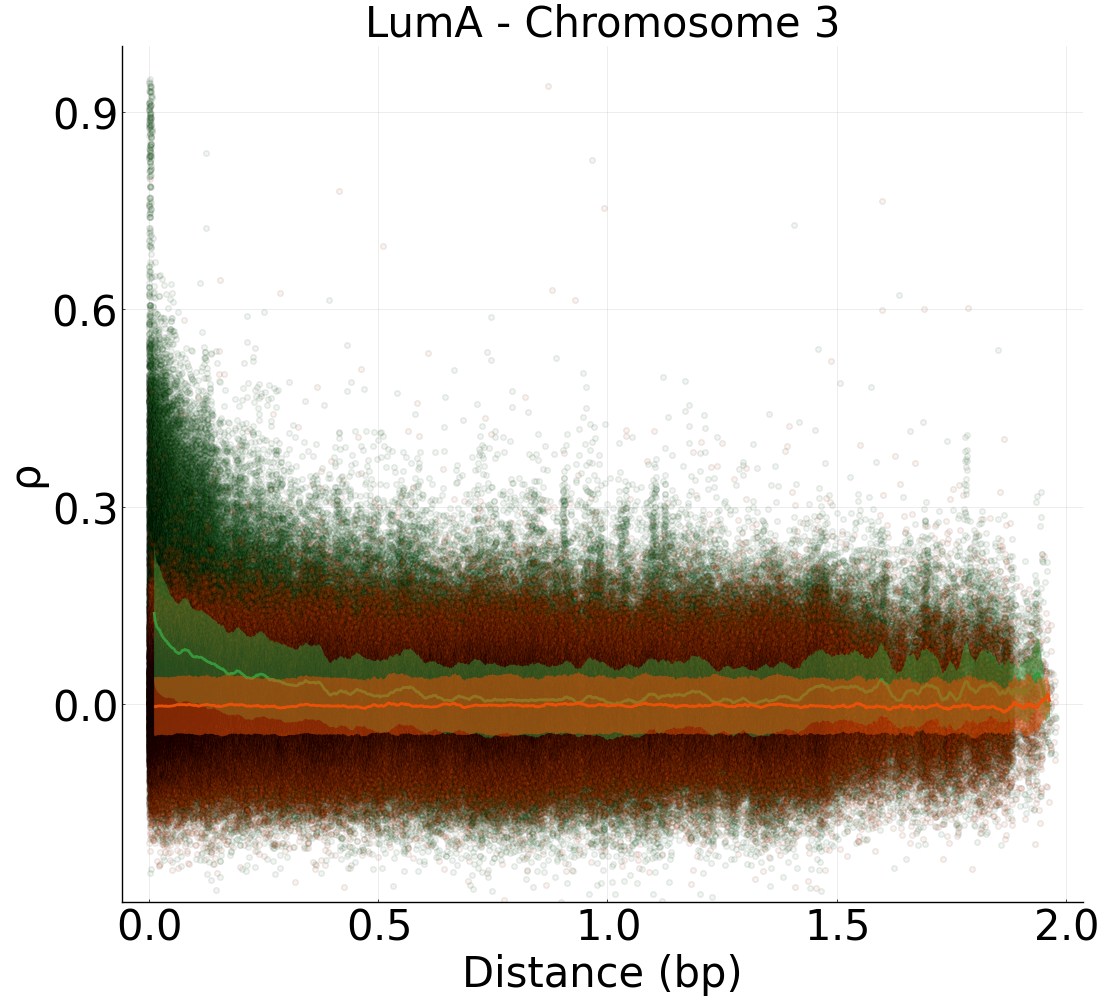

Supplement: Supplementary file 16 [file DataSheet_9.zip › SuppMat10Luma/Chromosome-3-LumA.png]

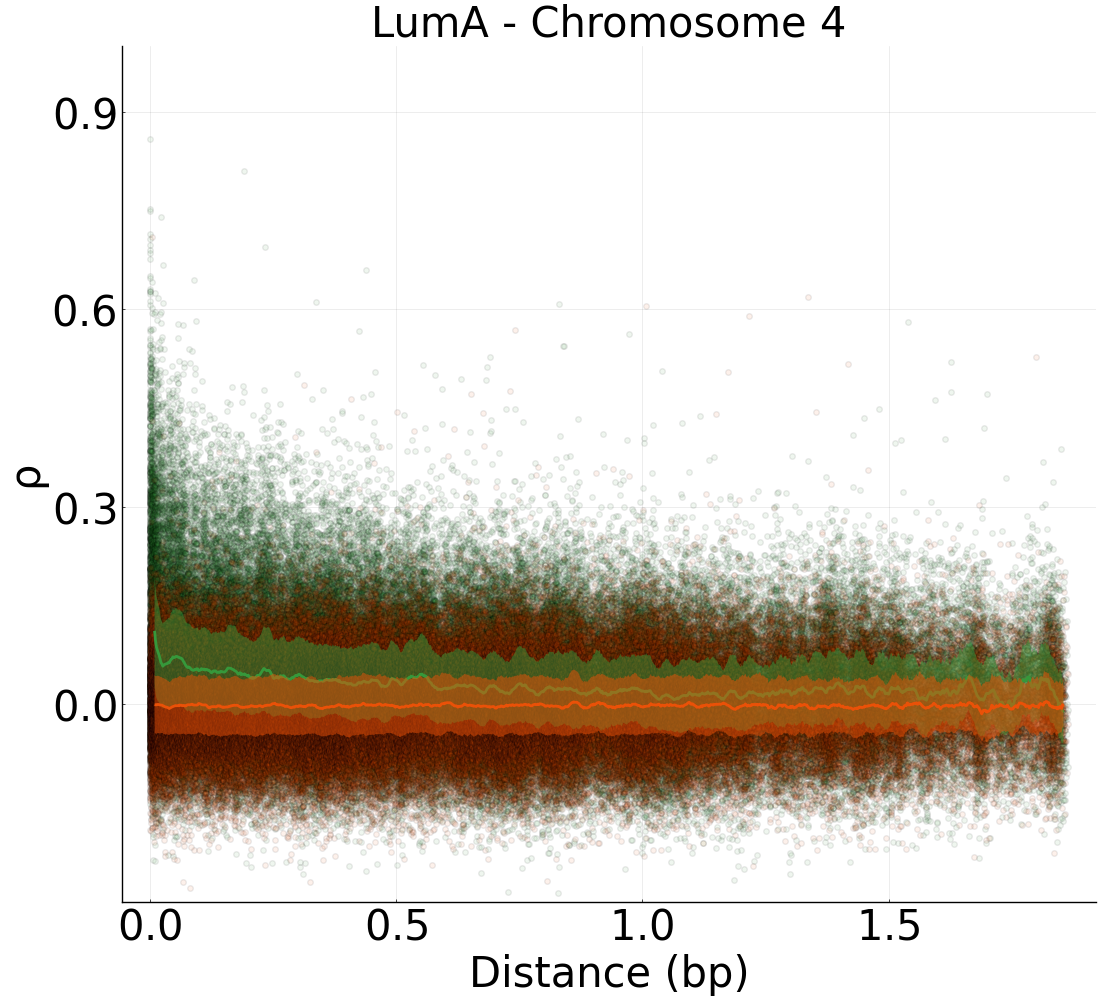

Supplement: Supplementary file 16 [file DataSheet_9.zip › SuppMat10Luma/Chromosome-4-LumA.png]

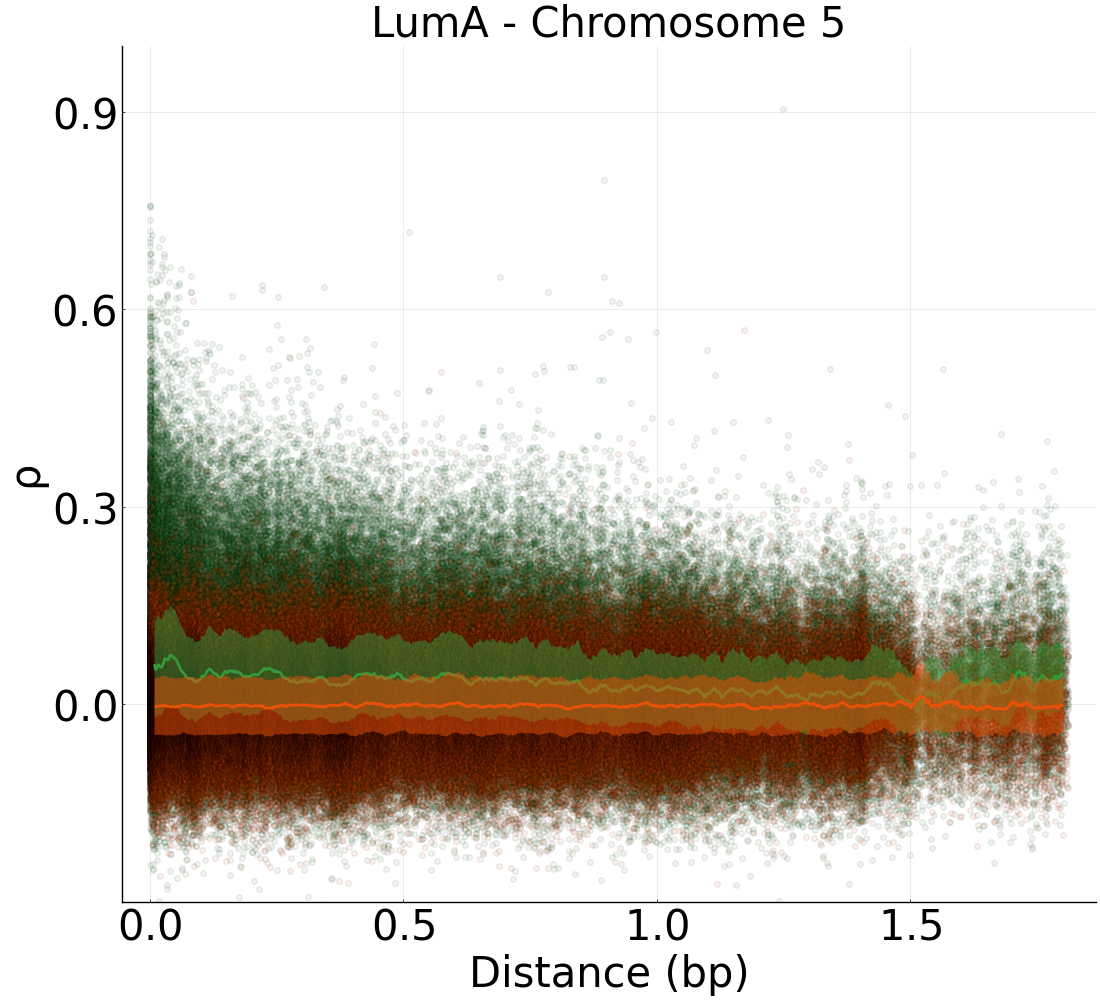

Supplement: Supplementary file 16 [file DataSheet_9.zip › SuppMat10Luma/Chromosome-5-LumA.png]

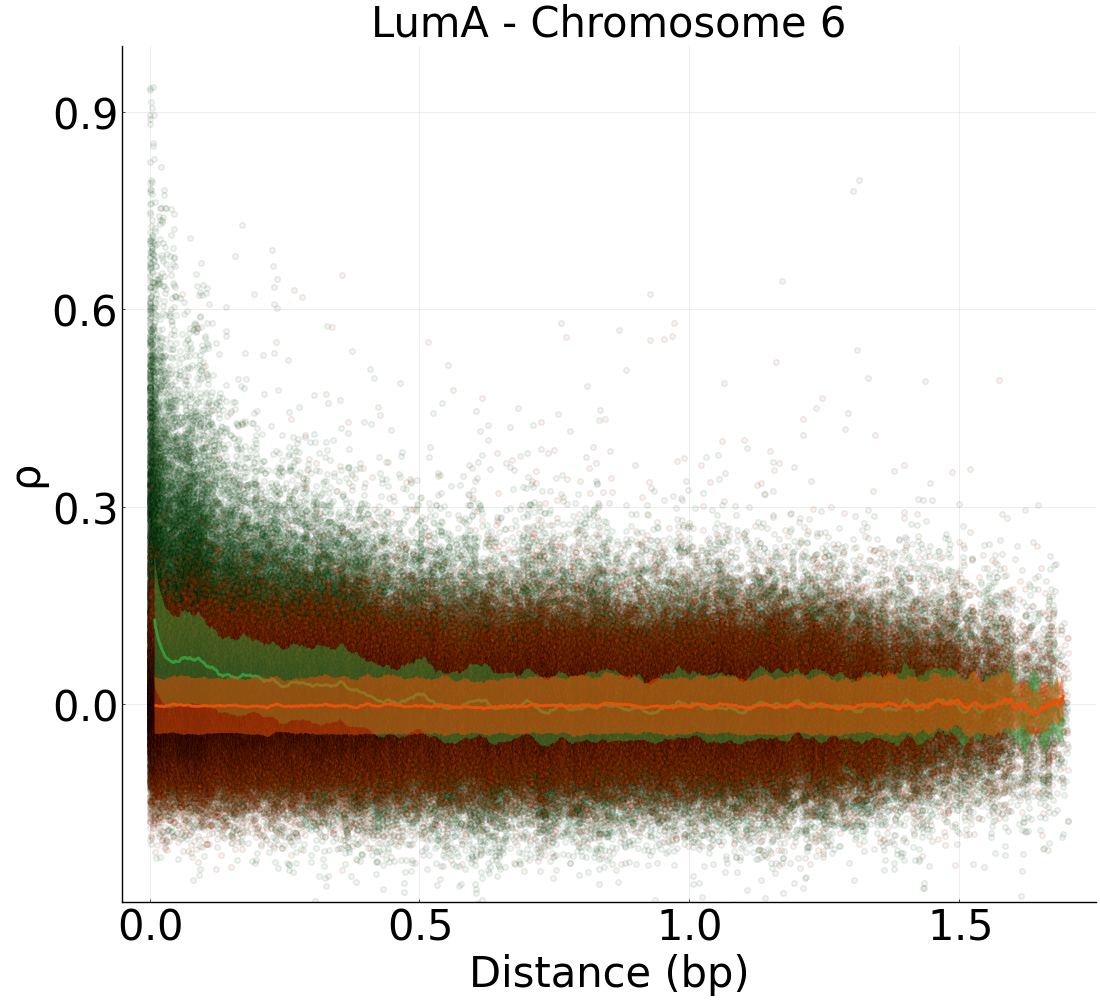

Supplement: Supplementary file 16 [file DataSheet_9.zip › SuppMat10Luma/Chromosome-6-LumA.png]

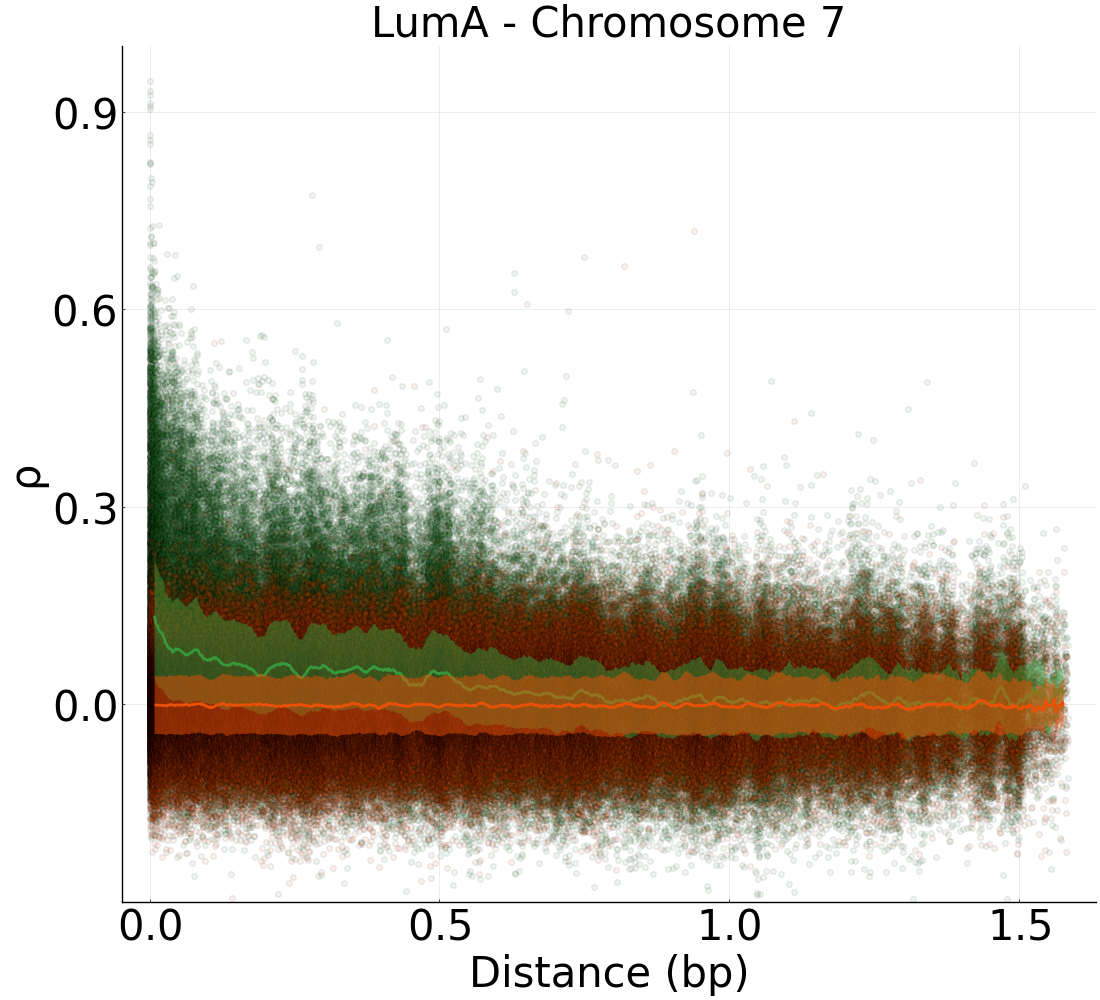

Supplement: Supplementary file 16 [file DataSheet_9.zip › SuppMat10Luma/Chromosome-7-LumA.png]

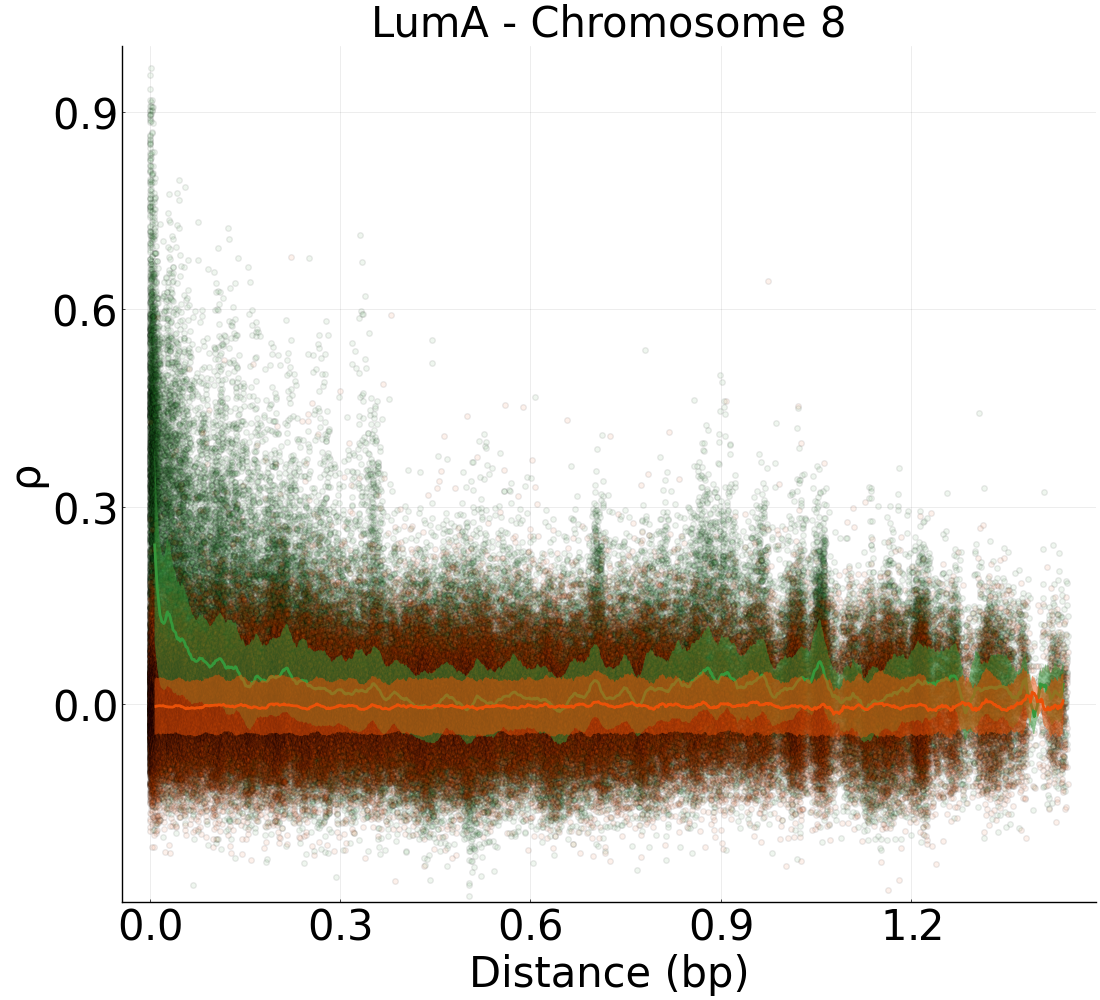

Supplement: Supplementary file 16 [file DataSheet_9.zip › SuppMat10Luma/Chromosome-8-LumA.png]

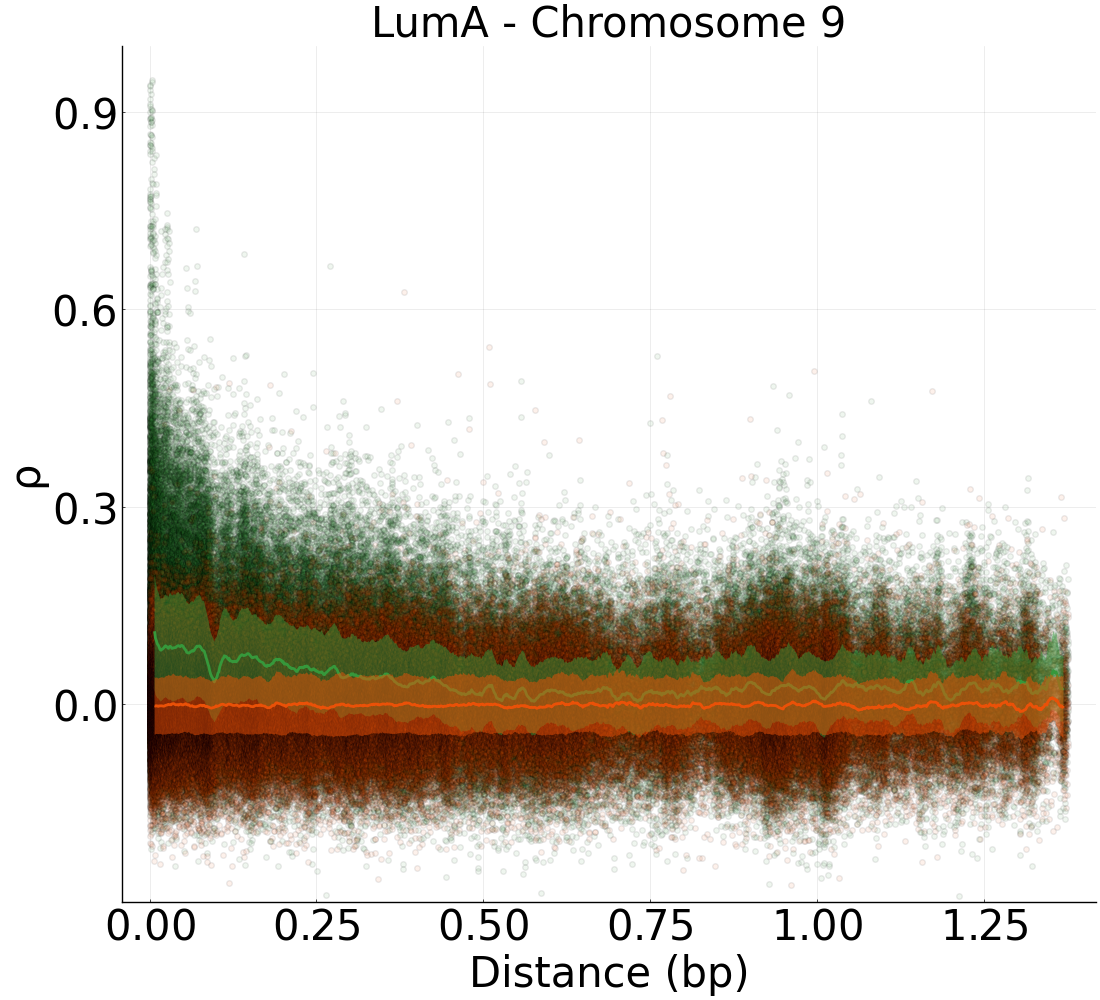

Supplement: Supplementary file 16 [file DataSheet_9.zip › SuppMat10Luma/Chromosome-9-LumA.png]

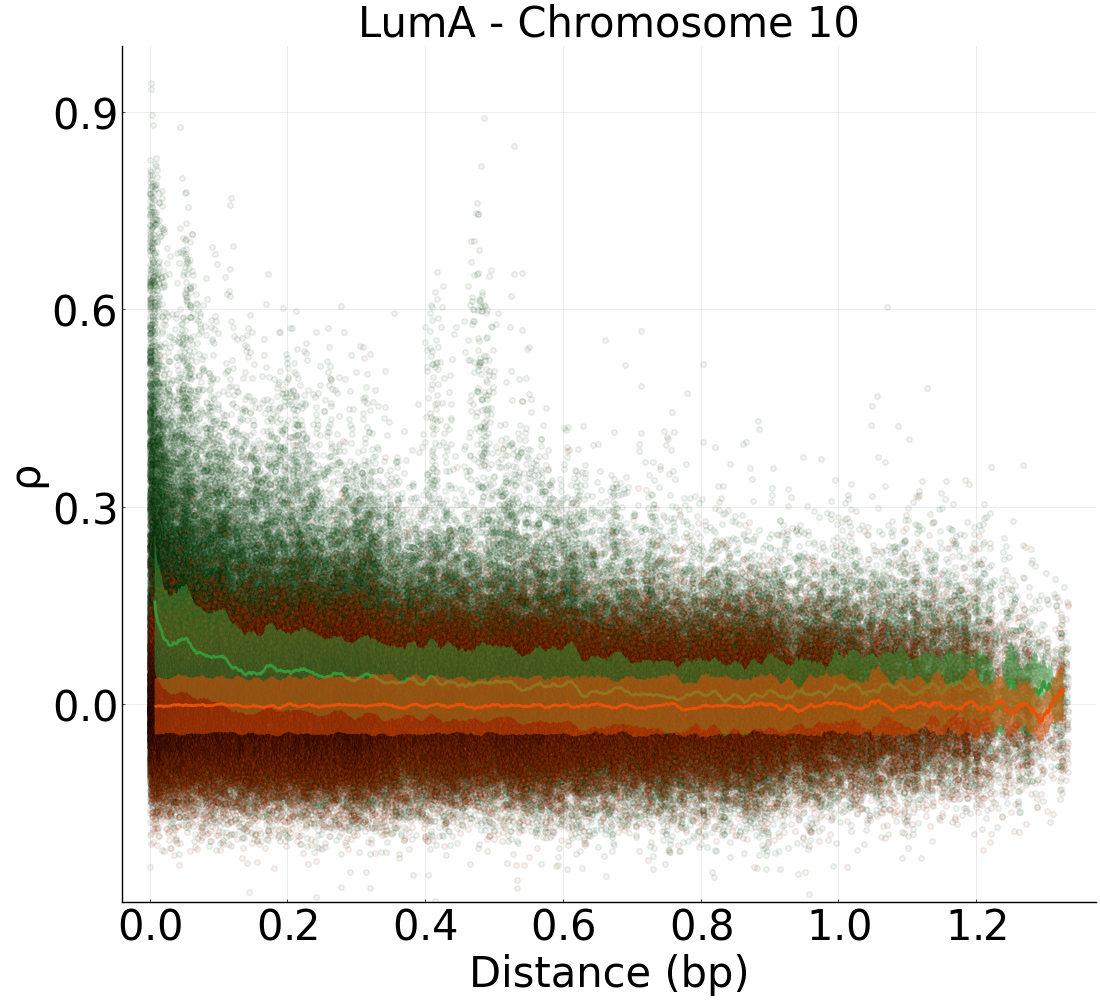

Supplement: Supplementary file 16 [file DataSheet_9.zip › SuppMat10Luma/Chromosome-10-LumA.png]

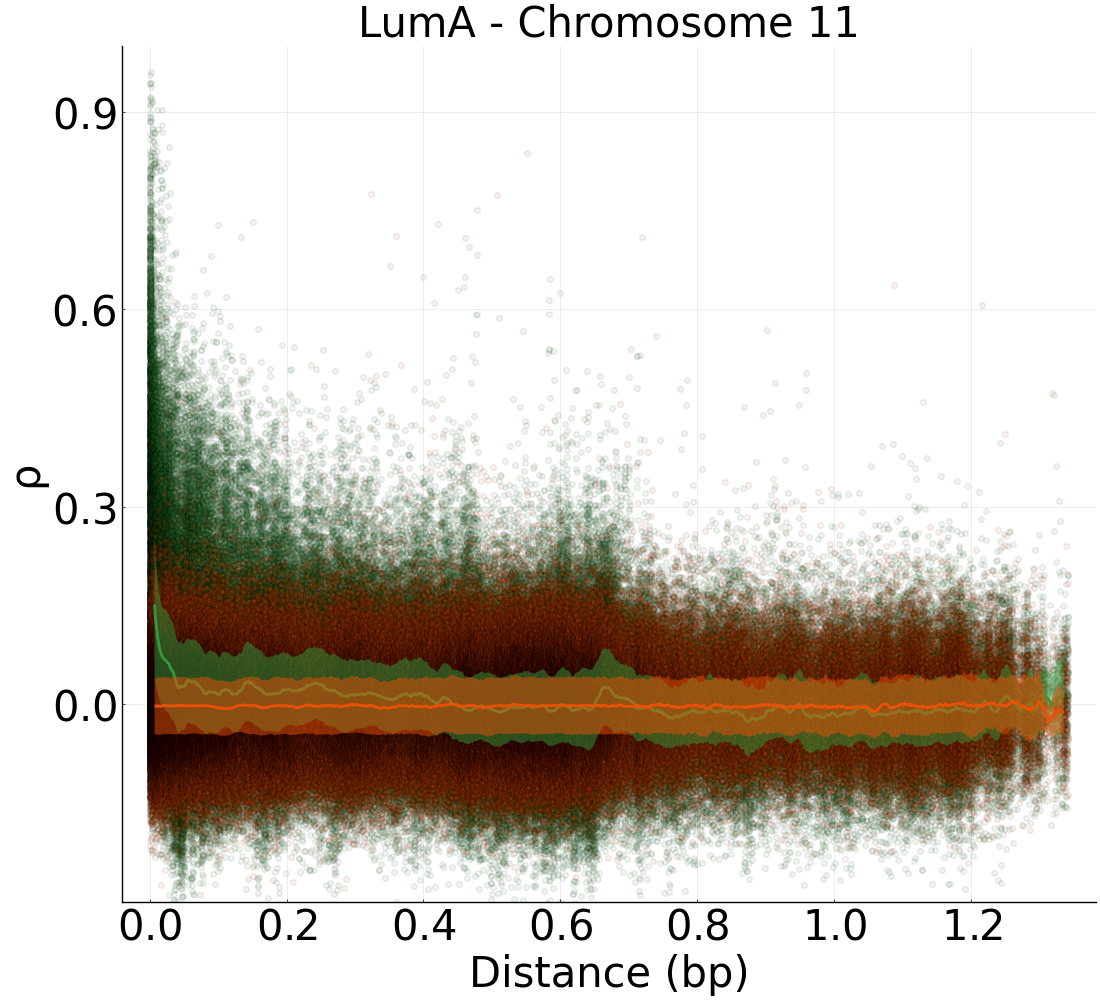

Supplement: Supplementary file 16 [file DataSheet_9.zip › SuppMat10Luma/Chromosome-11-LumA.png]

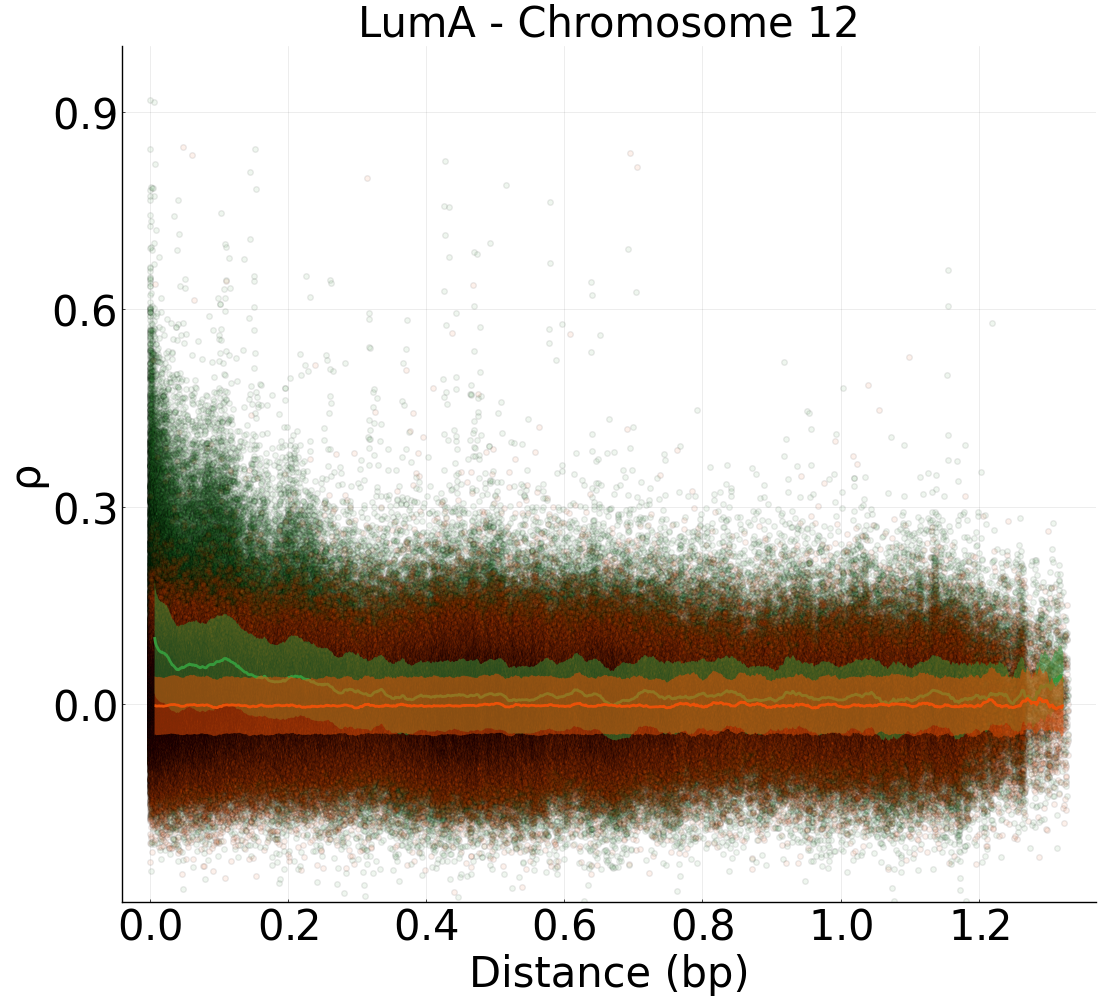

Supplement: Supplementary file 16 [file DataSheet_9.zip › SuppMat10Luma/Chromosome-12-LumA.png]

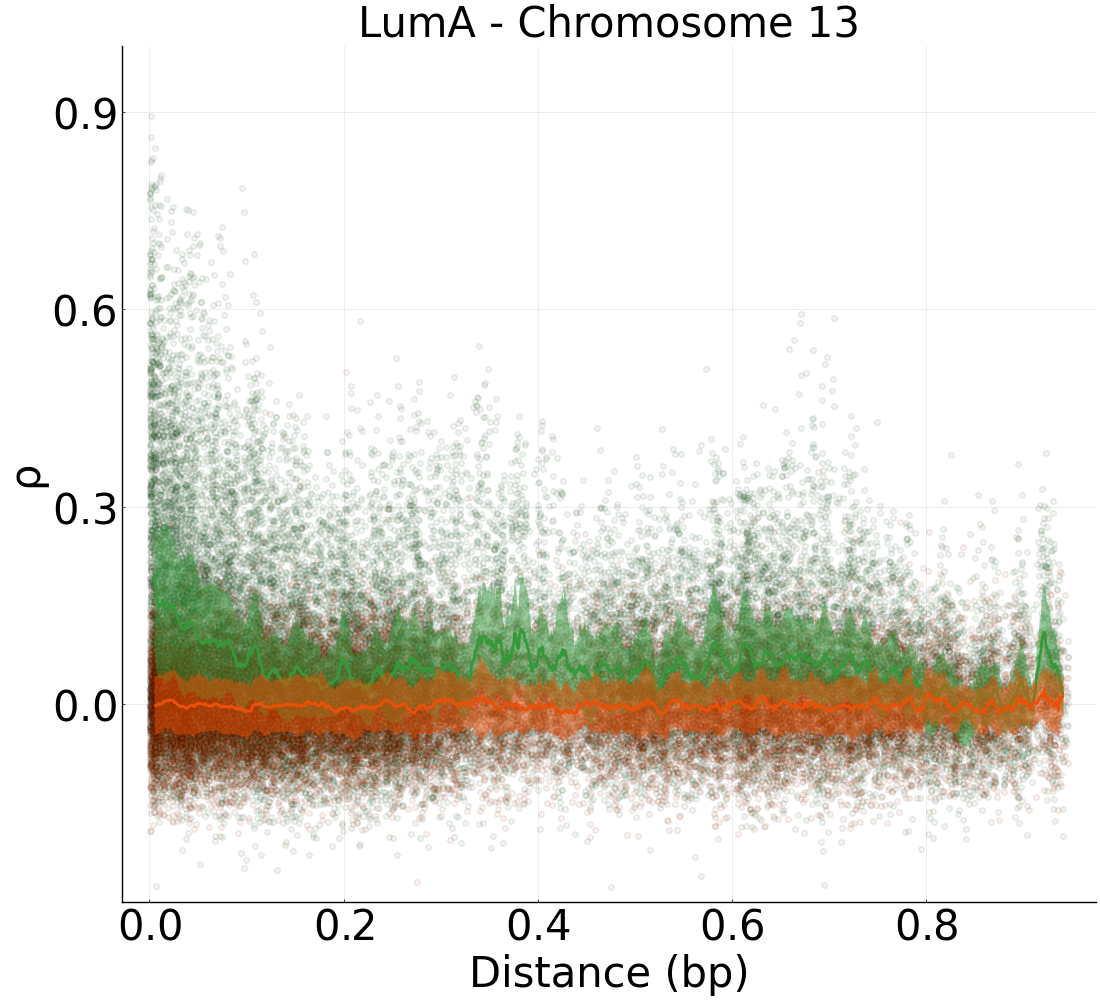

Supplement: Supplementary file 16 [file DataSheet_9.zip › SuppMat10Luma/Chromosome-13-LumA.png]

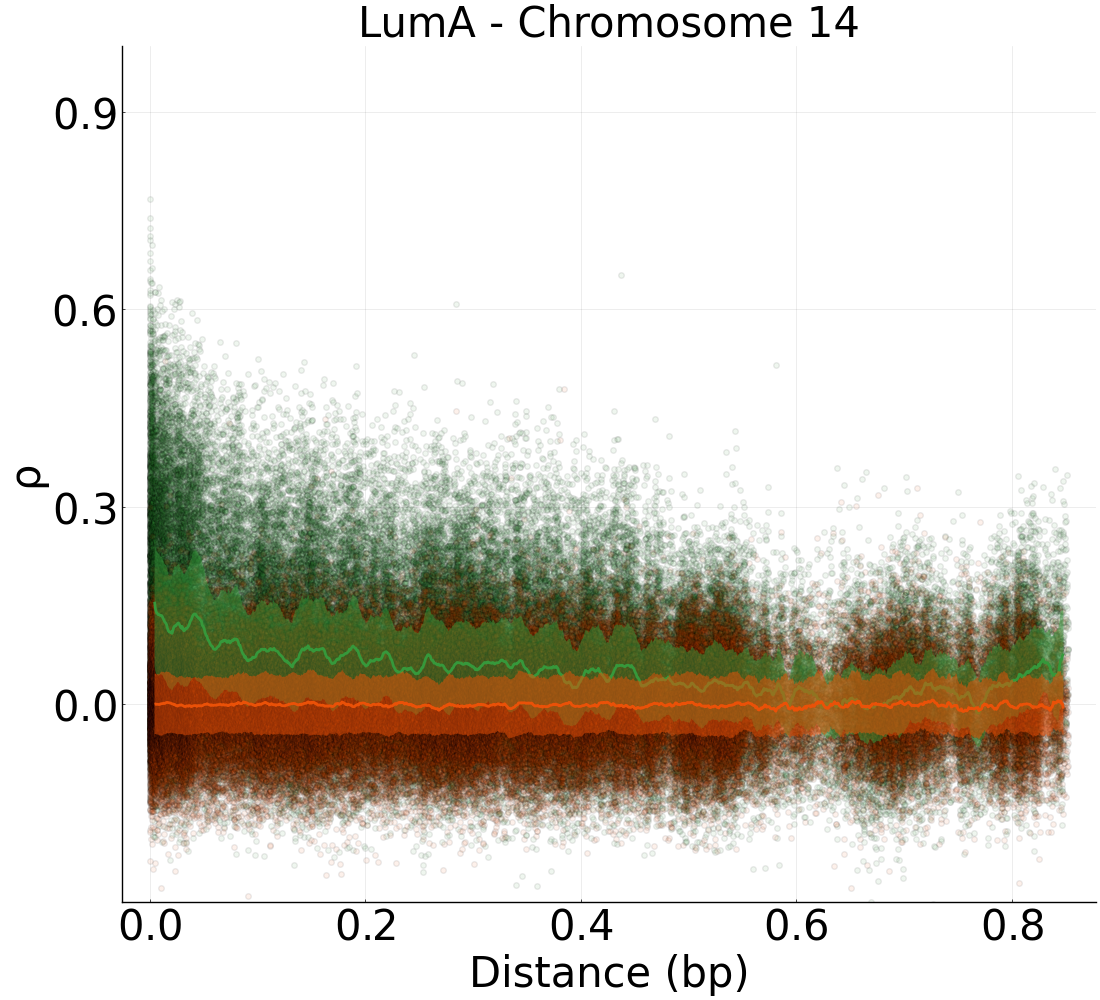

Supplement: Supplementary file 16 [file DataSheet_9.zip › SuppMat10Luma/Chromosome-14-LumA.png]

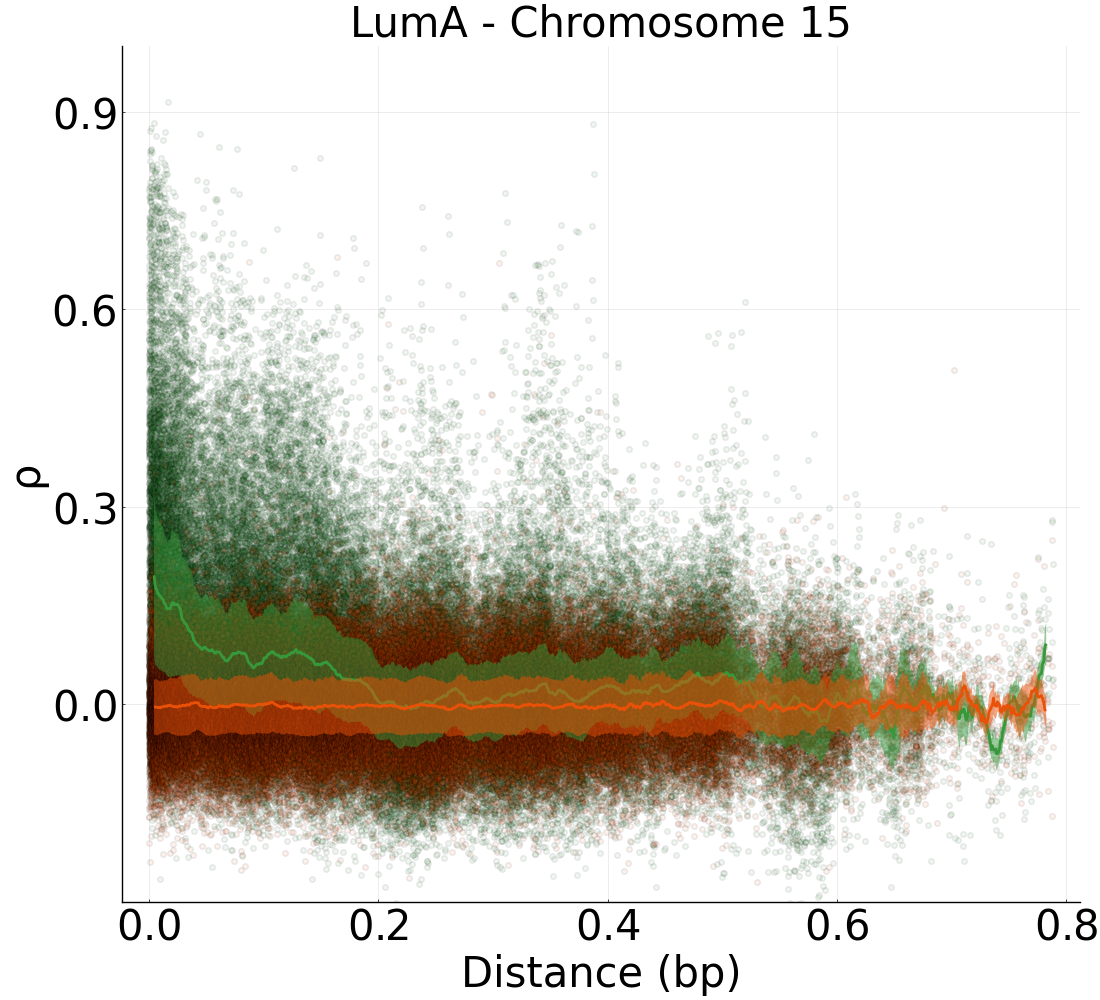

Supplement: Supplementary file 16 [file DataSheet_9.zip › SuppMat10Luma/Chromosome-15-LumA.png]

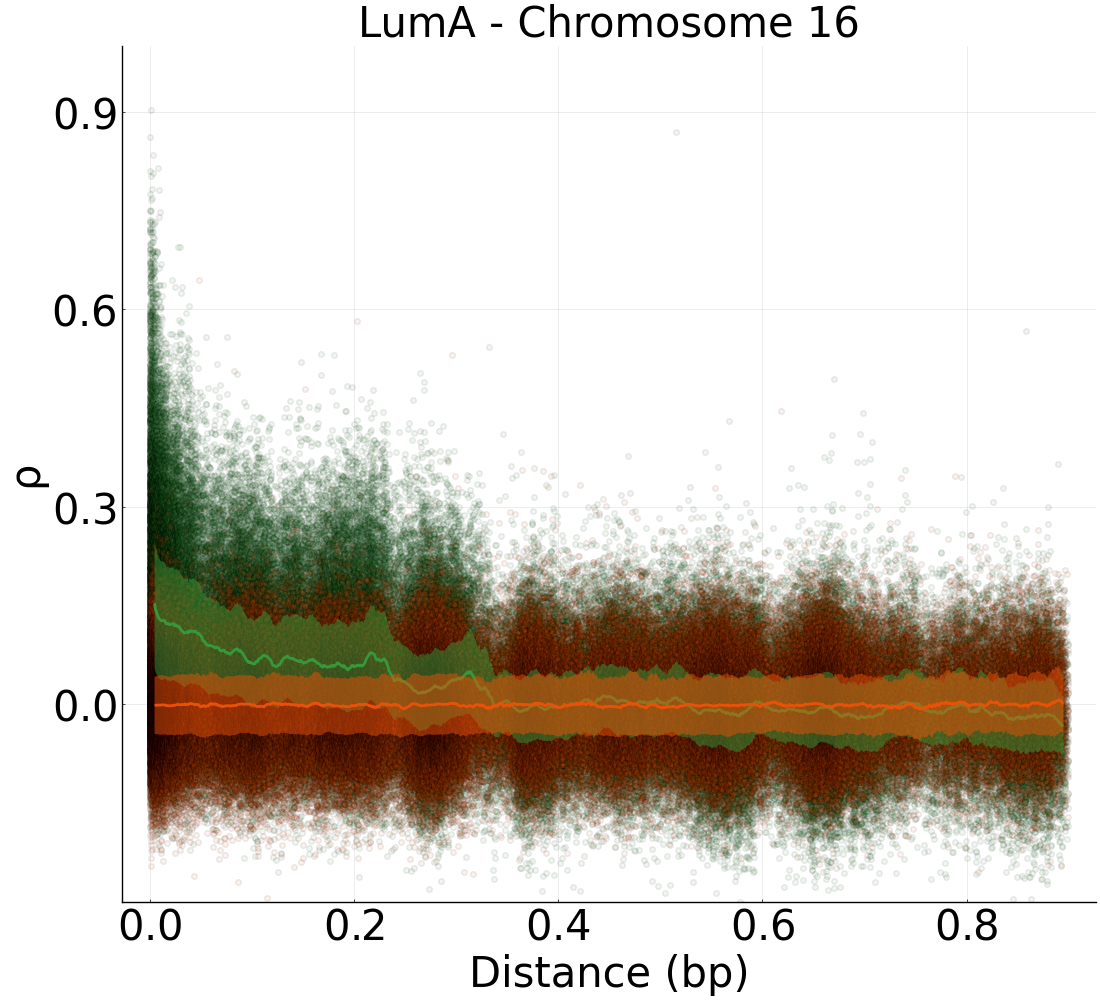

Supplement: Supplementary file 16 [file DataSheet_9.zip › SuppMat10Luma/Chromosome-16-LumA.png]

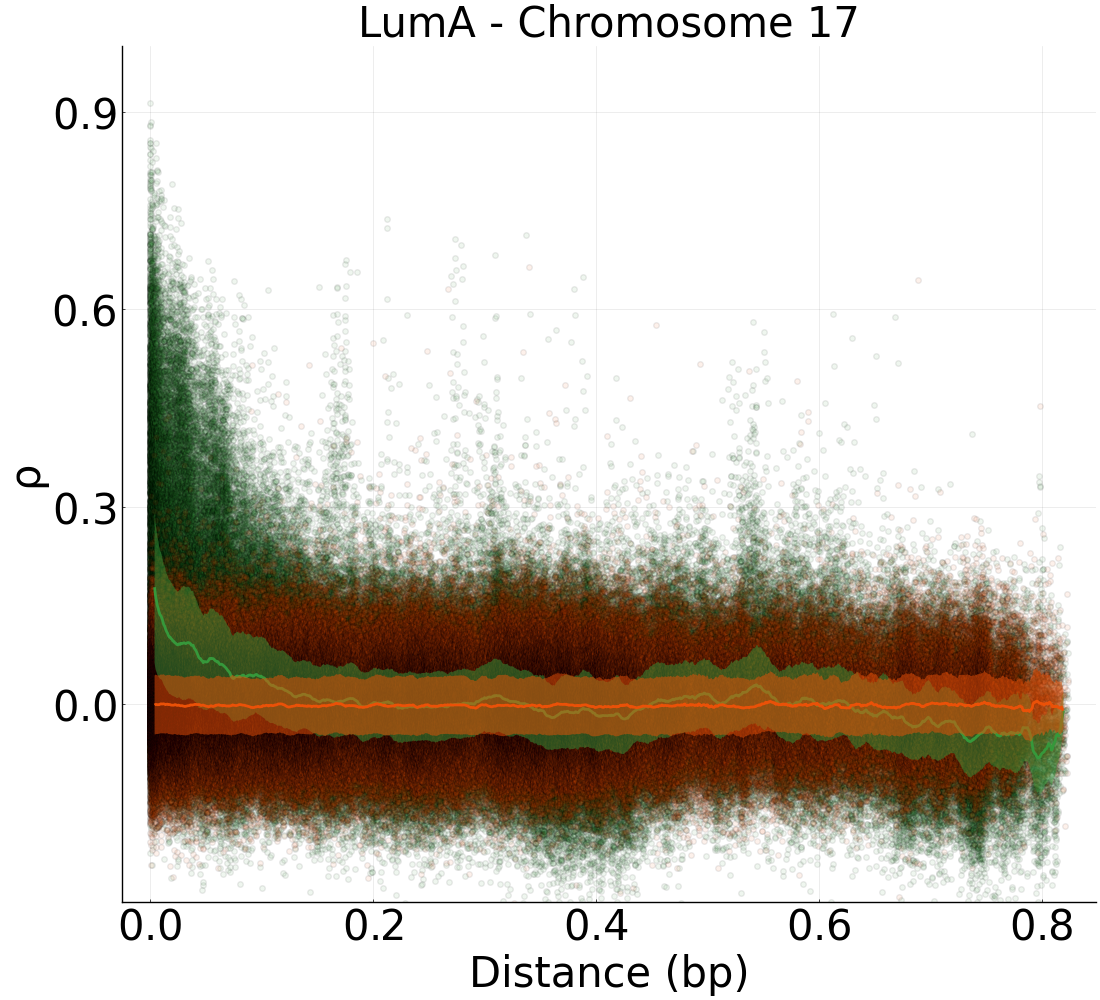

Supplement: Supplementary file 16 [file DataSheet_9.zip › SuppMat10Luma/Chromosome-17-LumA.png]

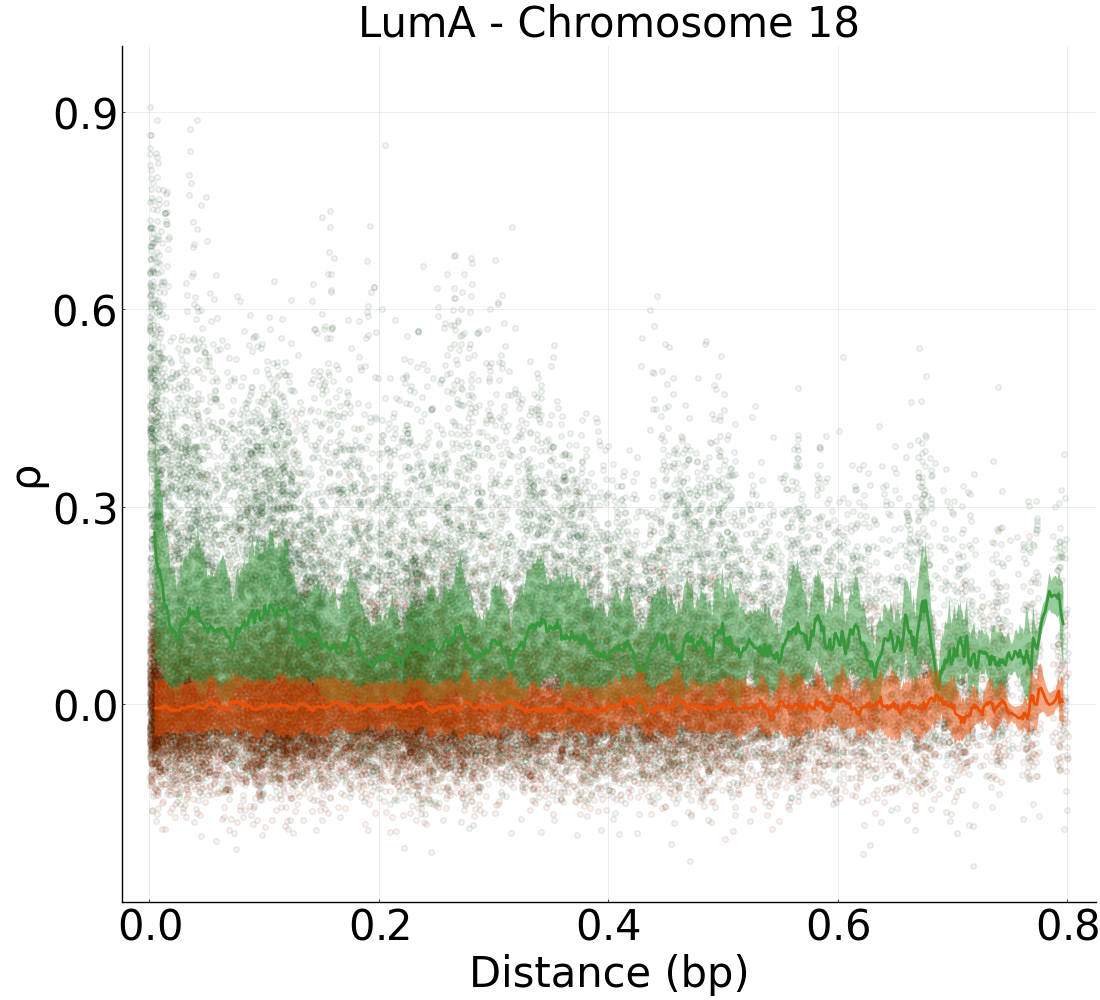

Supplement: Supplementary file 16 [file DataSheet_9.zip › SuppMat10Luma/Chromosome-18-LumA.png]

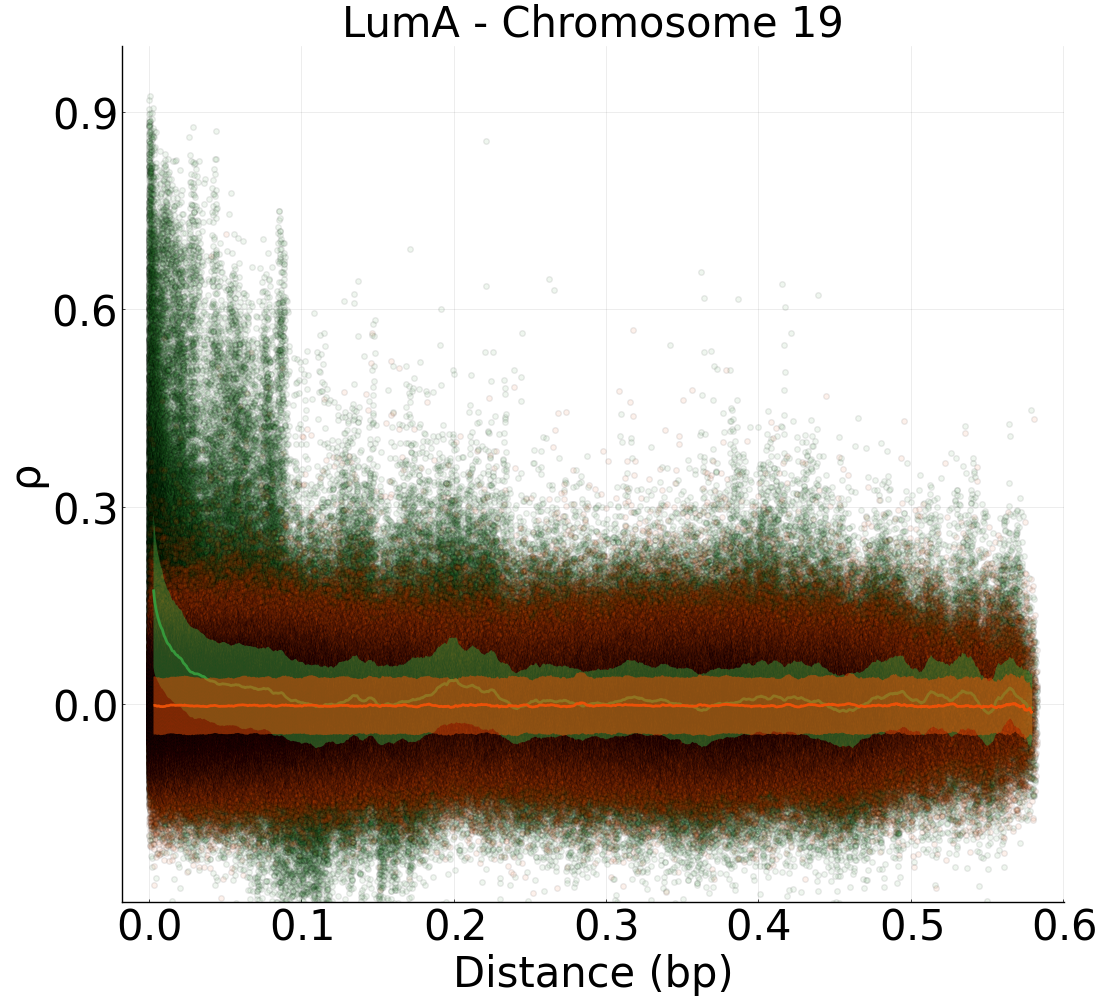

Supplement: Supplementary file 16 [file DataSheet_9.zip › SuppMat10Luma/Chromosome-19-LumA.png]

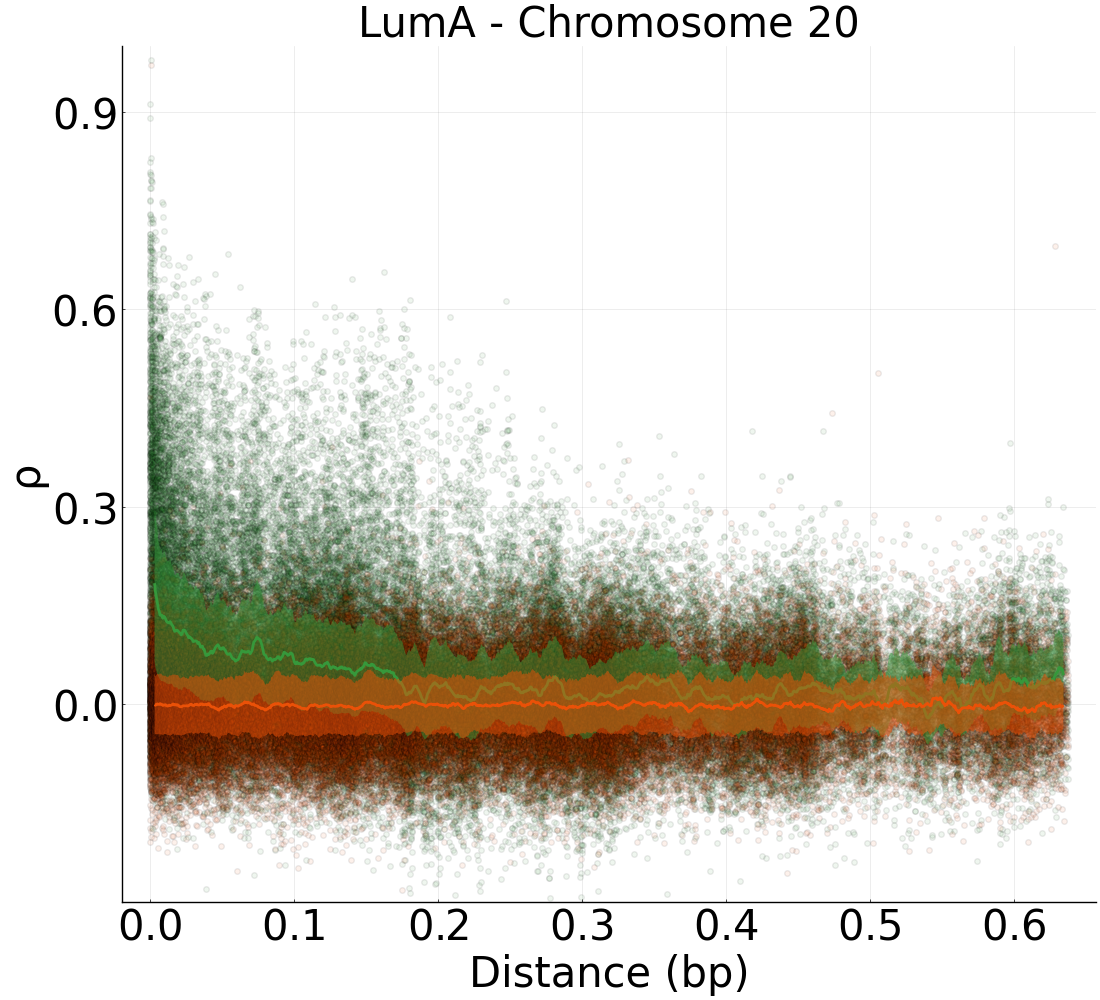

Supplement: Supplementary file 16 [file DataSheet_9.zip › SuppMat10Luma/Chromosome-20-LumA.png]

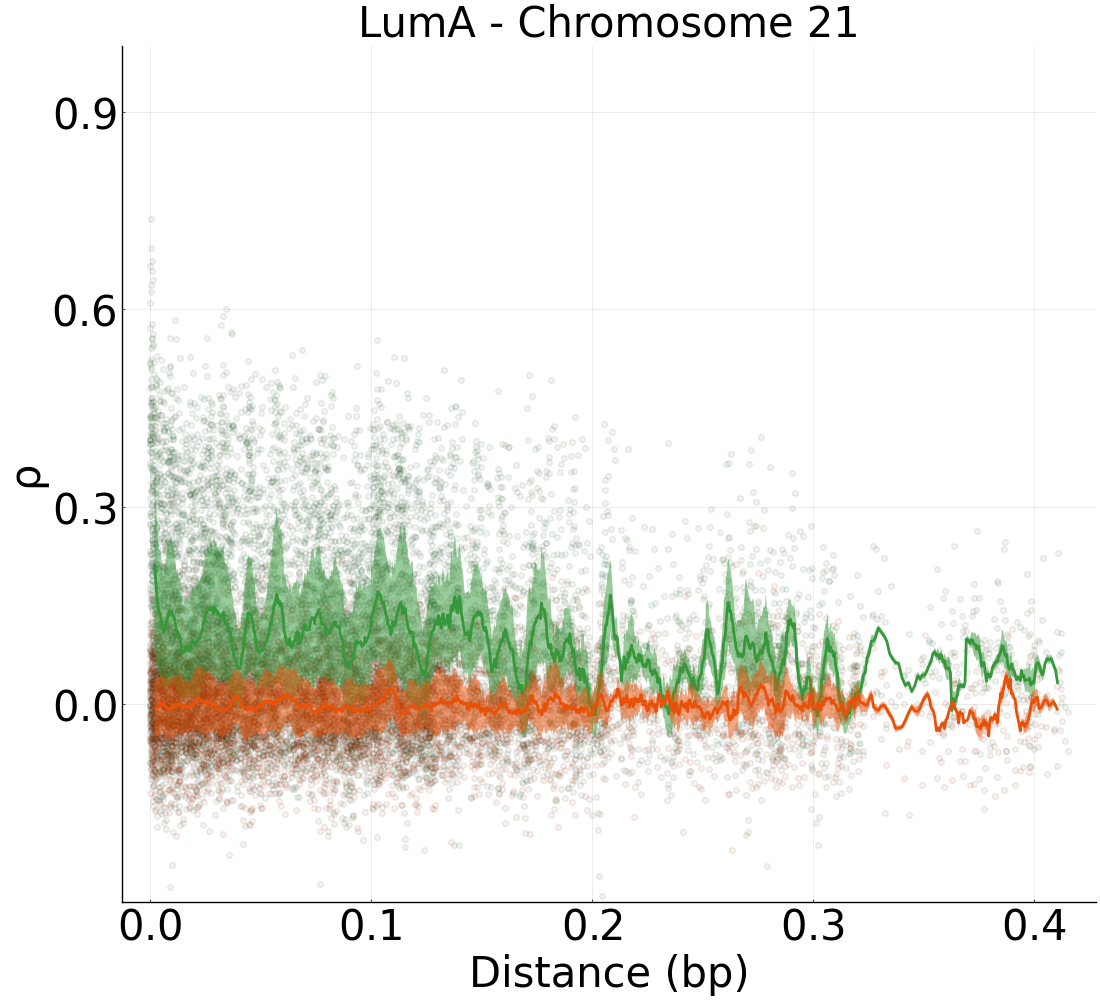

Supplement: Supplementary file 16 [file DataSheet_9.zip › SuppMat10Luma/Chromosome-21-LumA.png]

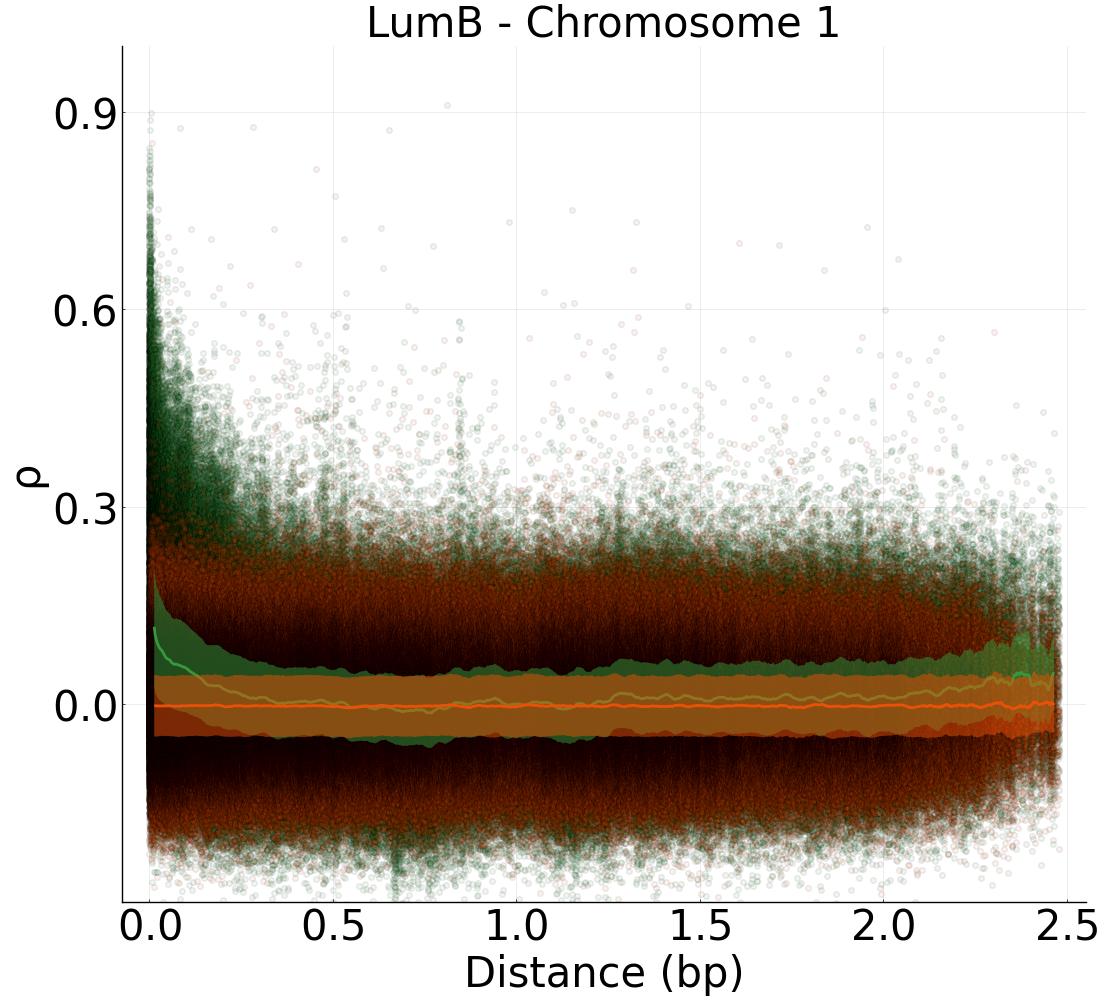

Supplement: Supplementary file 17 [file DataSheet_10.zip › SuppMat11Lumb/Chromosome-1-LumB.png]

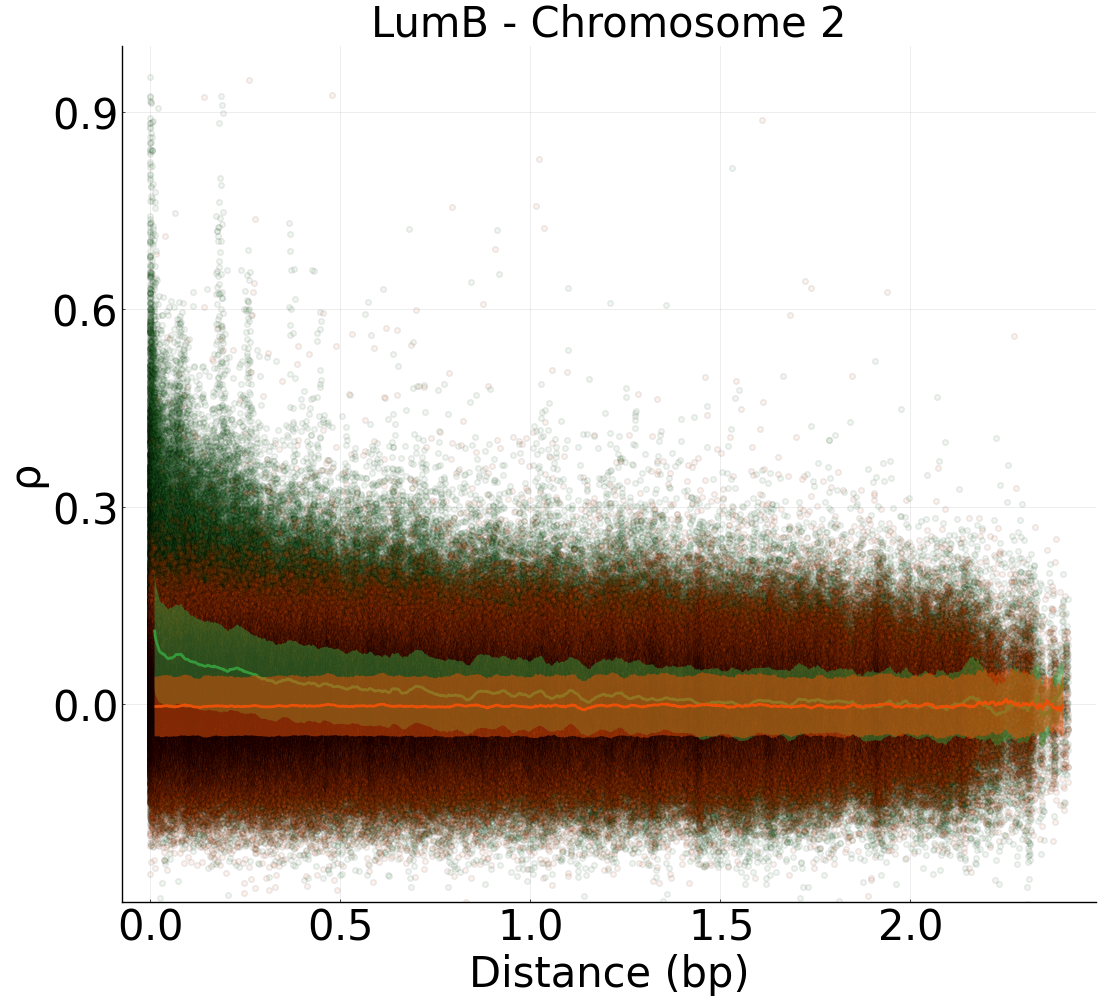

Supplement: Supplementary file 17 [file DataSheet_10.zip › SuppMat11Lumb/Chromosome-2-LumB.png]

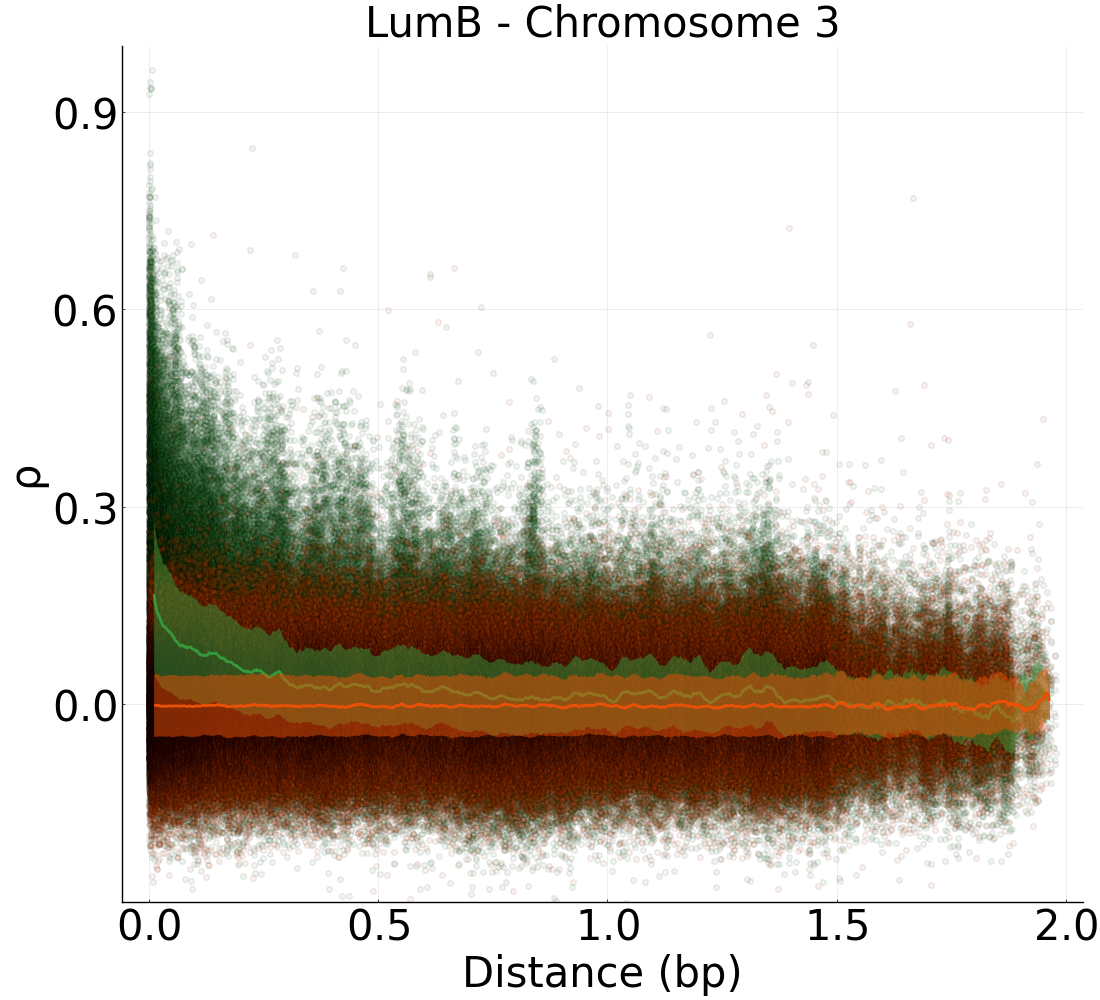

Supplement: Supplementary file 17 [file DataSheet_10.zip › SuppMat11Lumb/Chromosome-3-LumB.png]

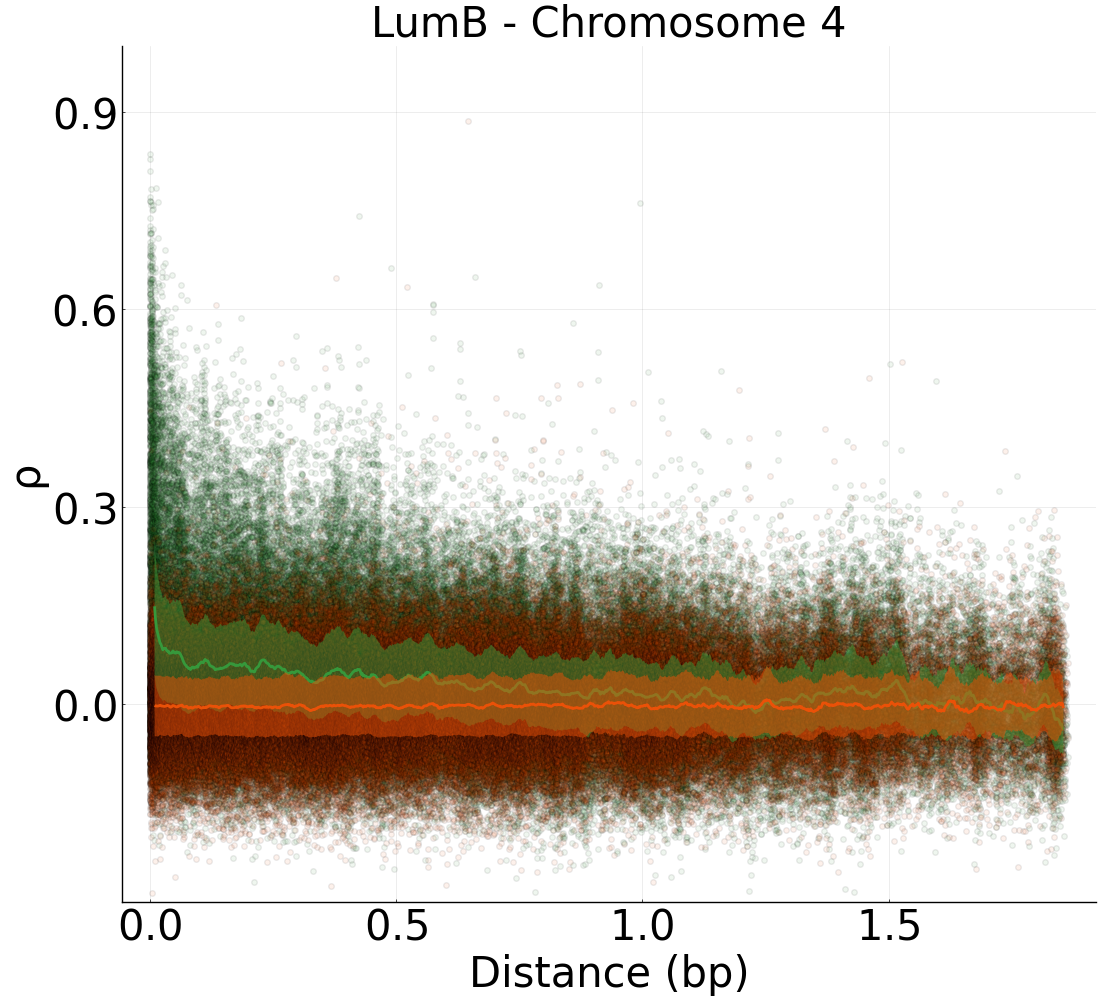

Supplement: Supplementary file 17 [file DataSheet_10.zip › SuppMat11Lumb/Chromosome-4-LumB.png]

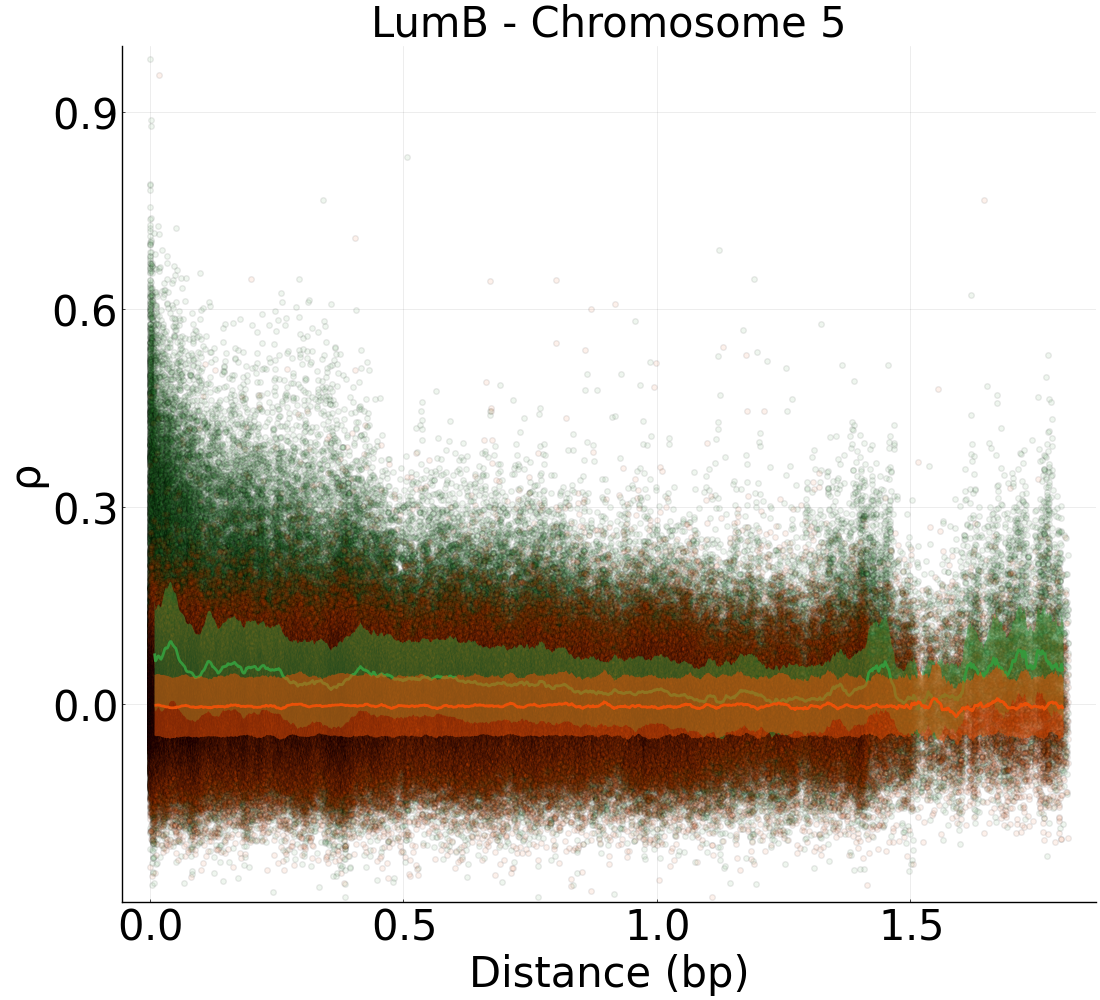

Supplement: Supplementary file 17 [file DataSheet_10.zip › SuppMat11Lumb/Chromosome-5-LumB.png]

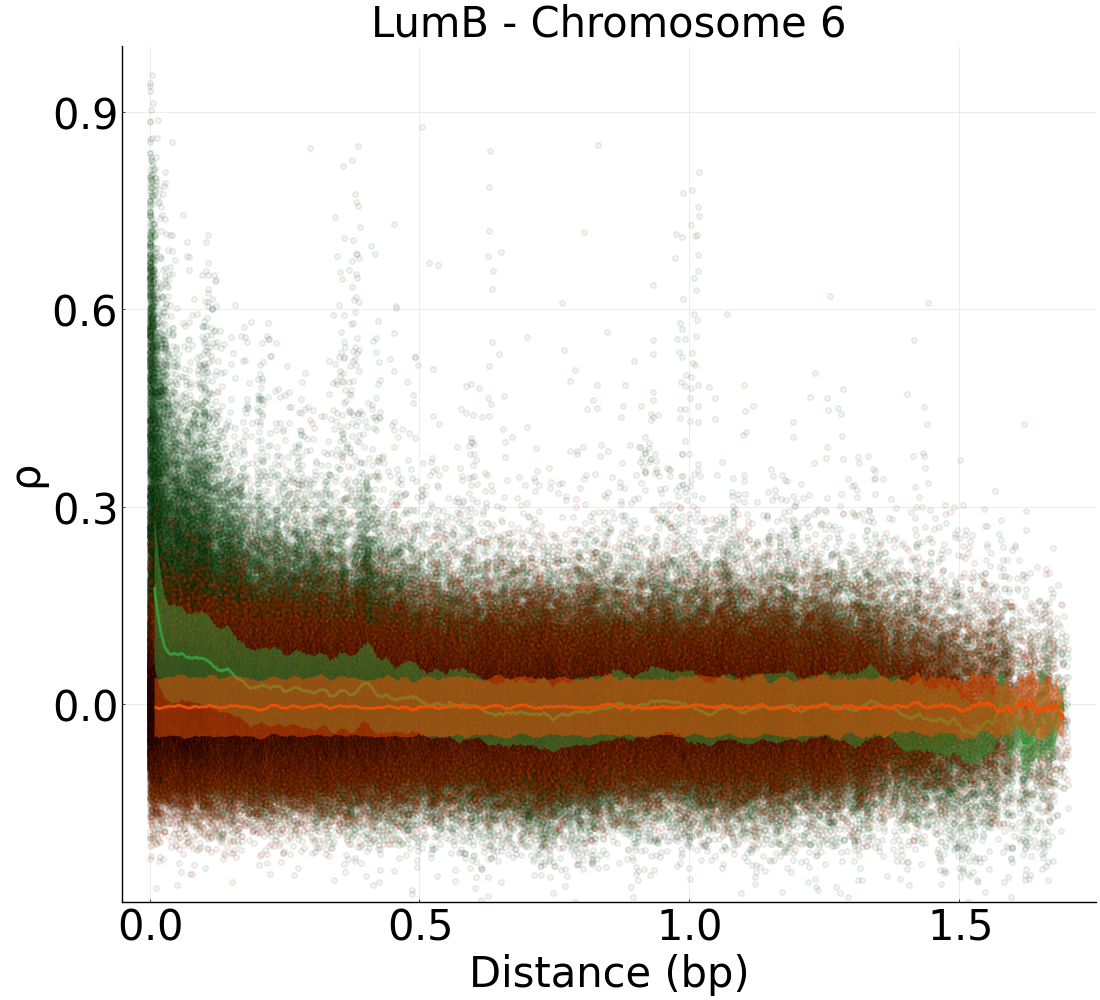

Supplement: Supplementary file 17 [file DataSheet_10.zip › SuppMat11Lumb/Chromosome-6-LumB.png]

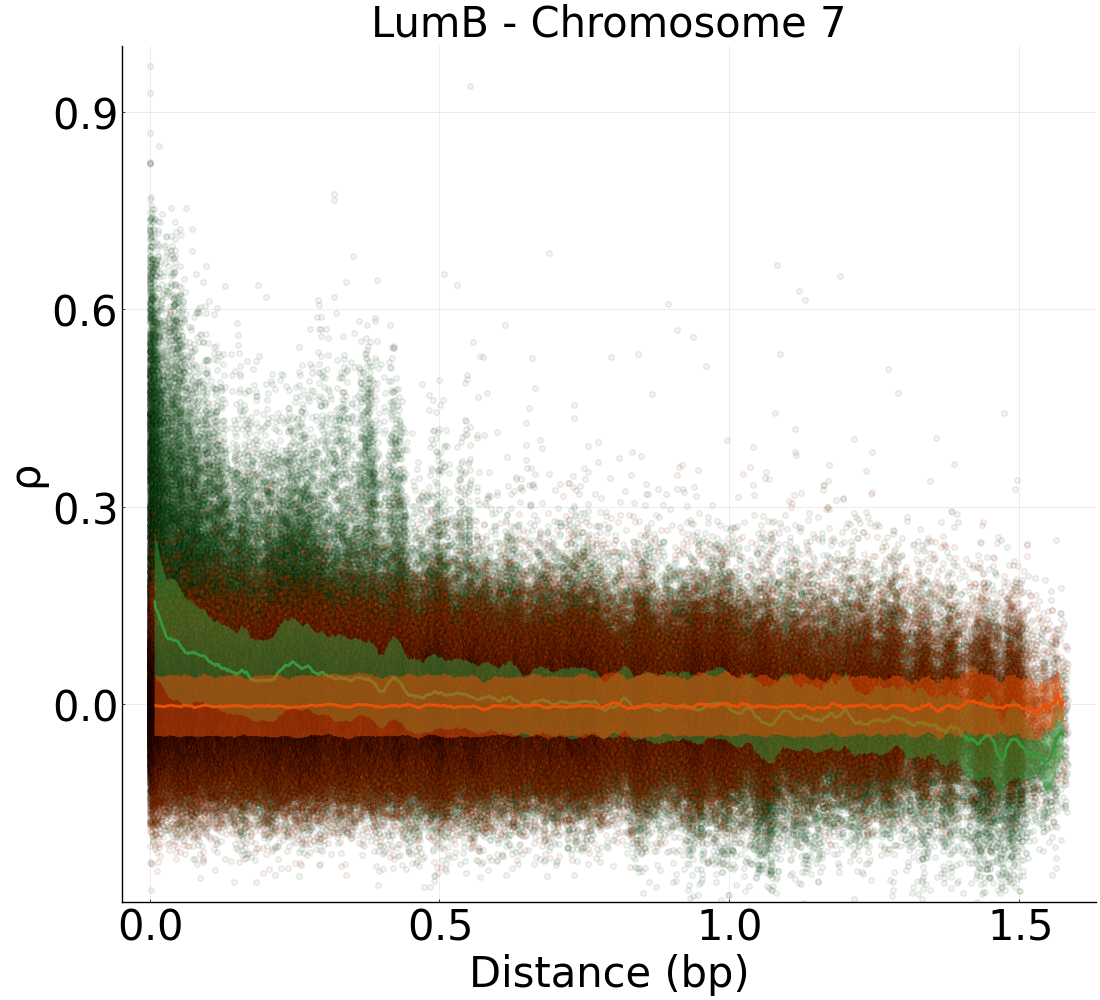

Supplement: Supplementary file 17 [file DataSheet_10.zip › SuppMat11Lumb/Chromosome-7-LumB.png]

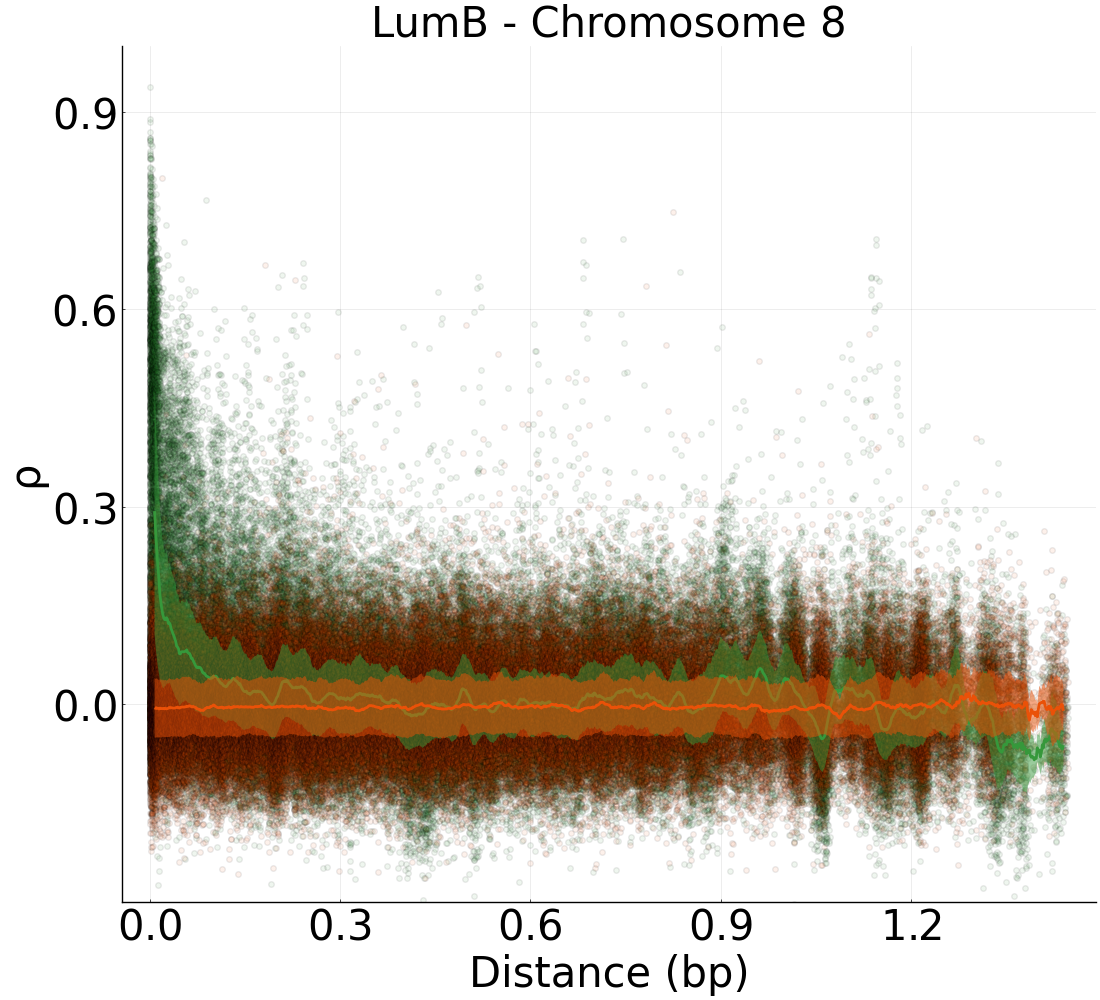

Supplement: Supplementary file 17 [file DataSheet_10.zip › SuppMat11Lumb/Chromosome-8-LumB.png]

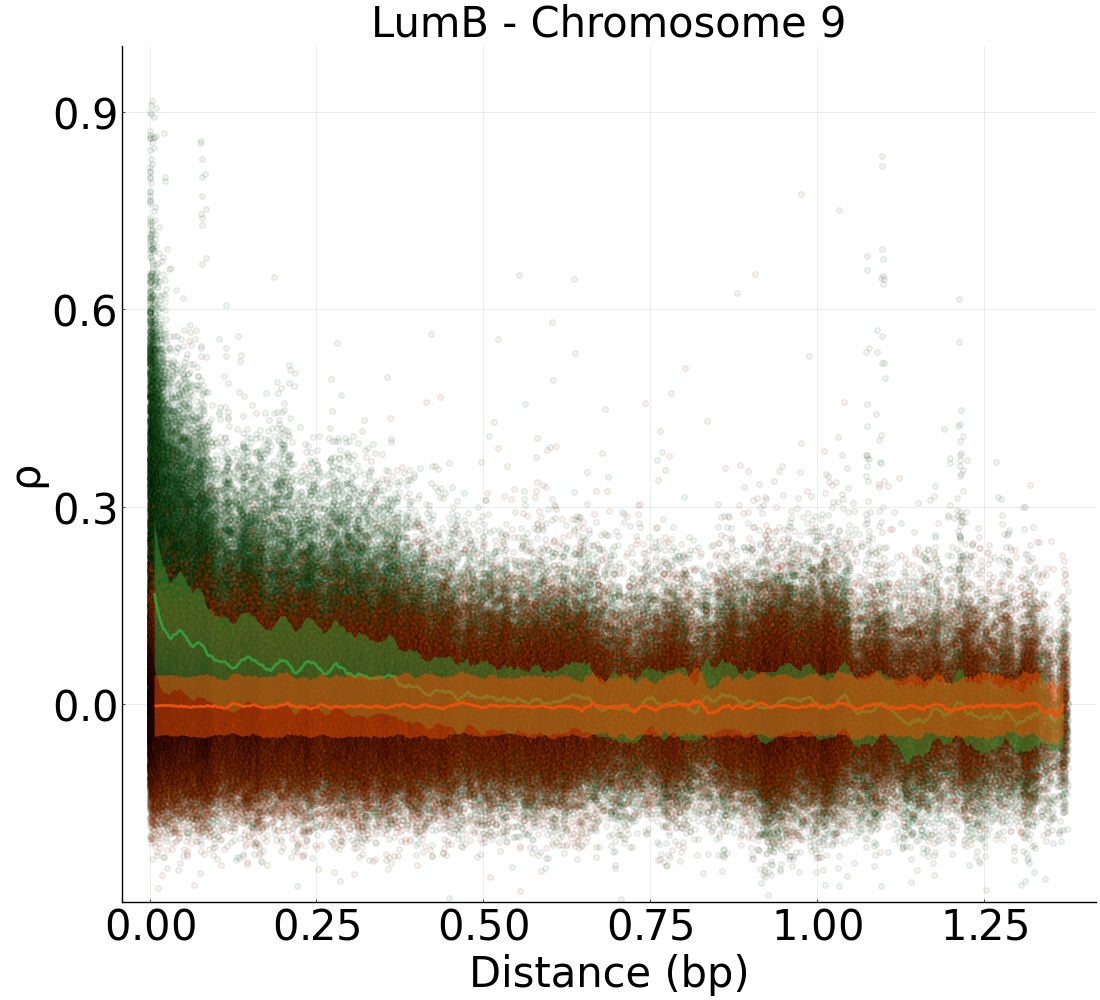

Supplement: Supplementary file 17 [file DataSheet_10.zip › SuppMat11Lumb/Chromosome-9-LumB.png]

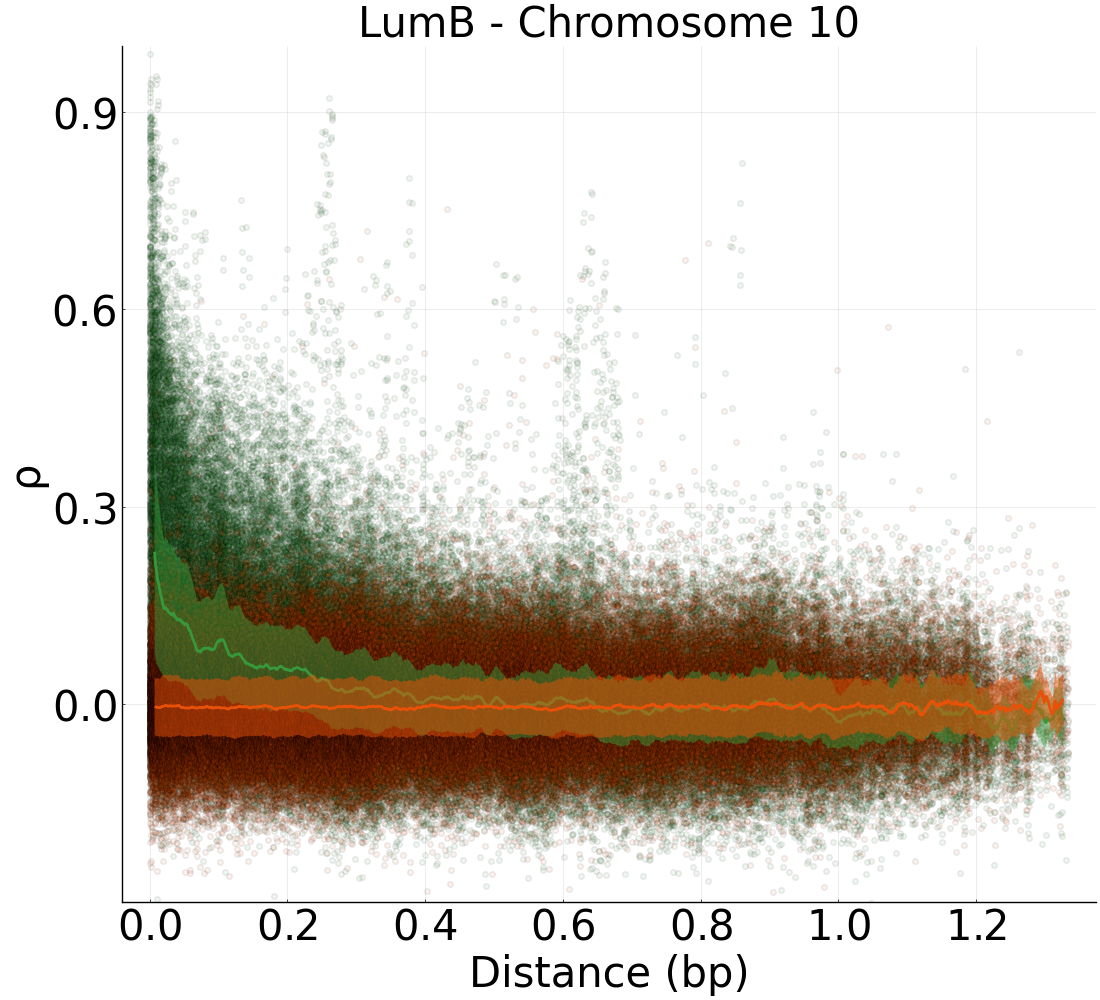

Supplement: Supplementary file 17 [file DataSheet_10.zip › SuppMat11Lumb/Chromosome-10-LumB.png]

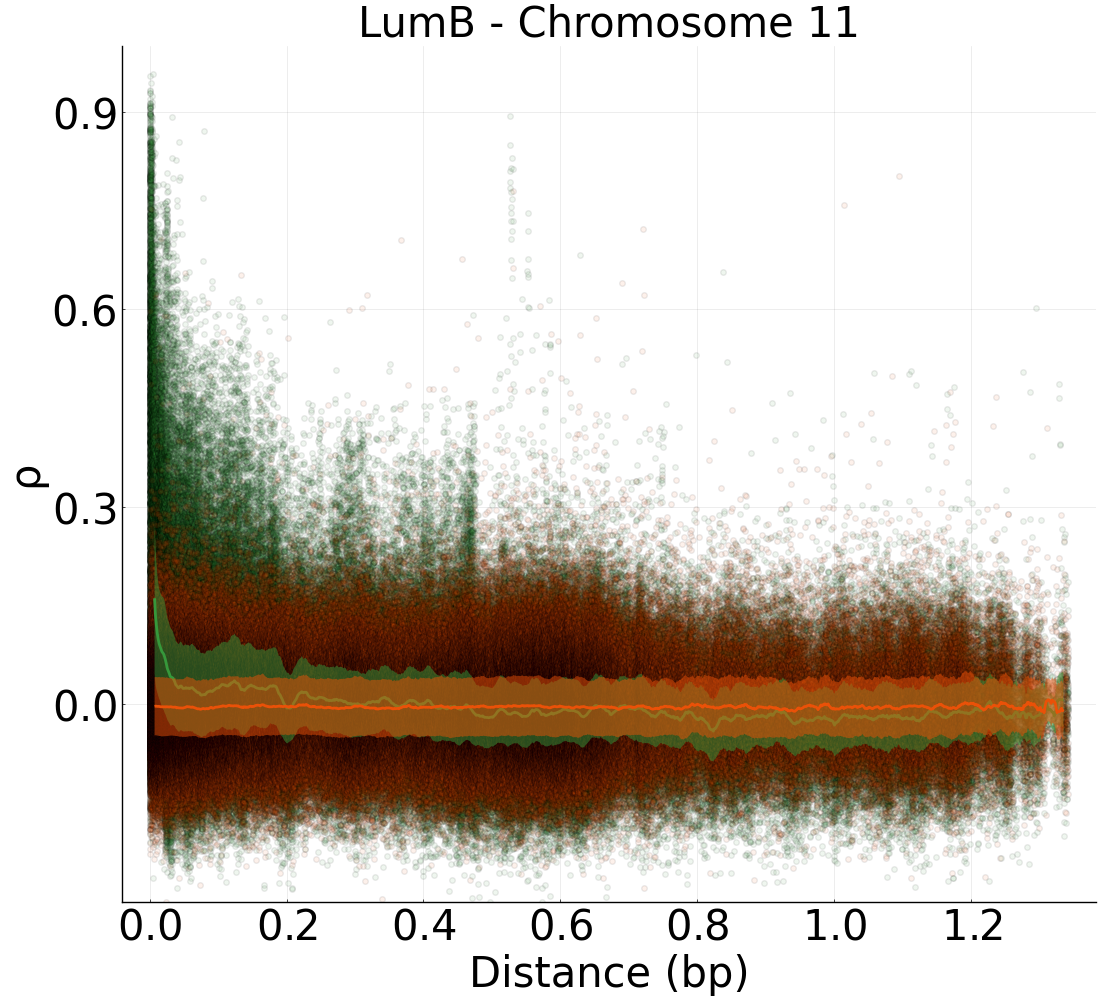

Supplement: Supplementary file 17 [file DataSheet_10.zip › SuppMat11Lumb/Chromosome-11-LumB.png]

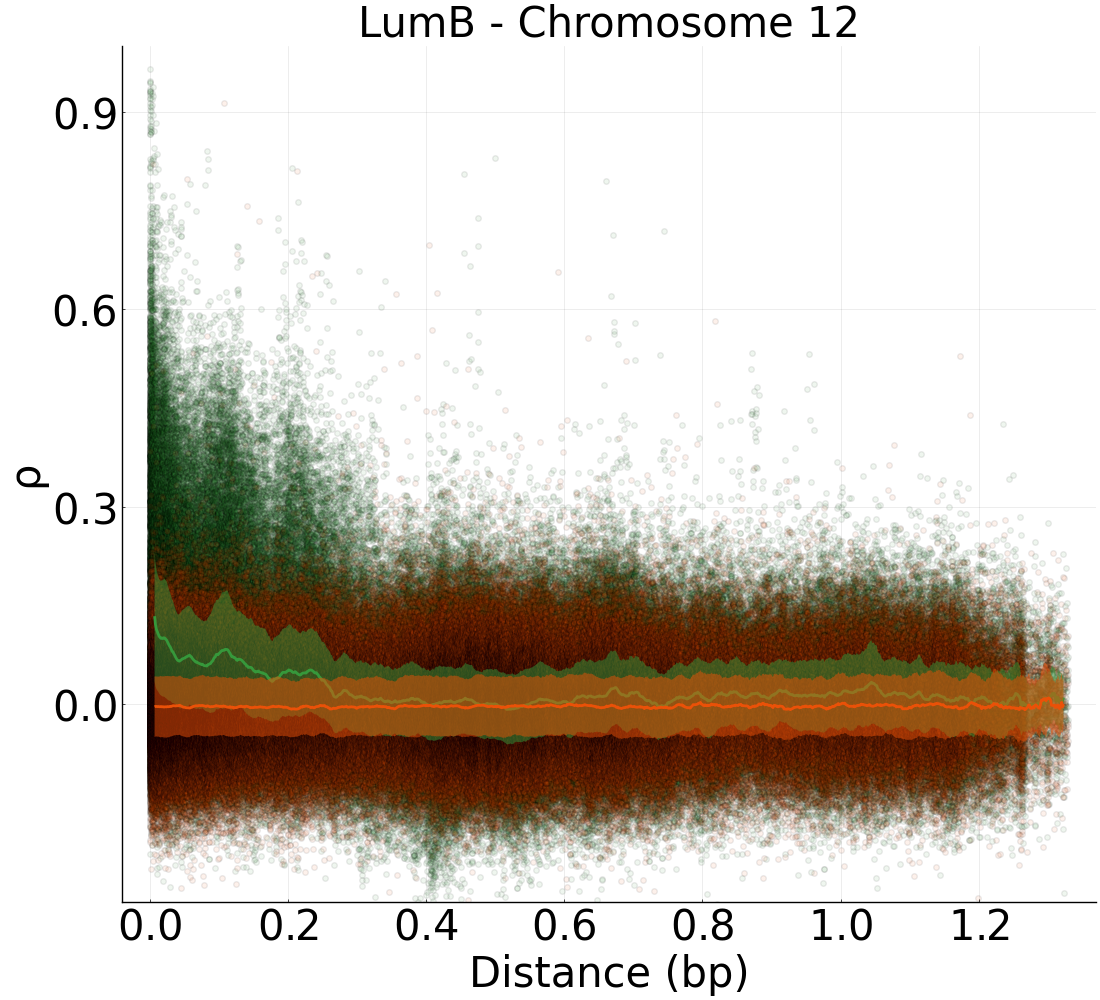

Supplement: Supplementary file 17 [file DataSheet_10.zip › SuppMat11Lumb/Chromosome-12-LumB.png]

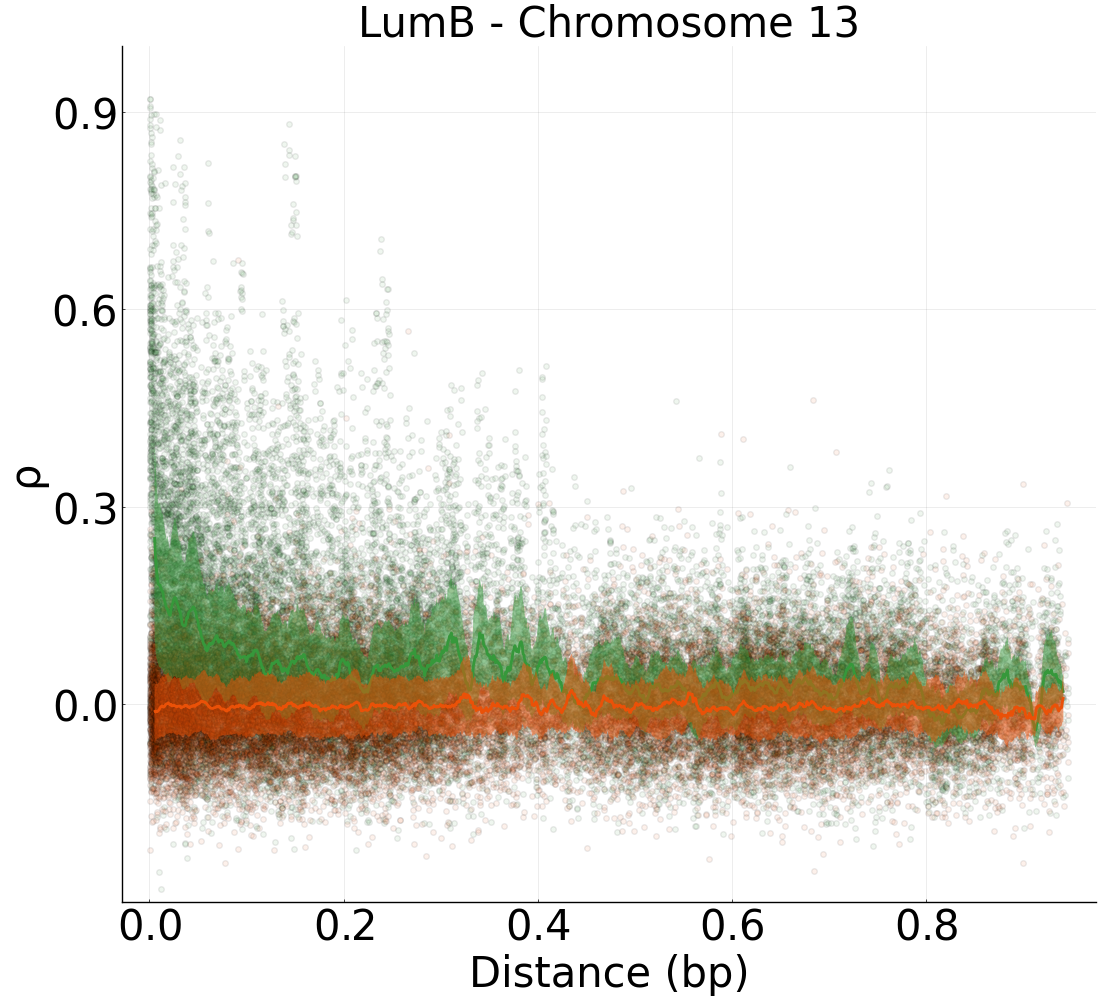

Supplement: Supplementary file 17 [file DataSheet_10.zip › SuppMat11Lumb/Chromosome-13-LumB.png]

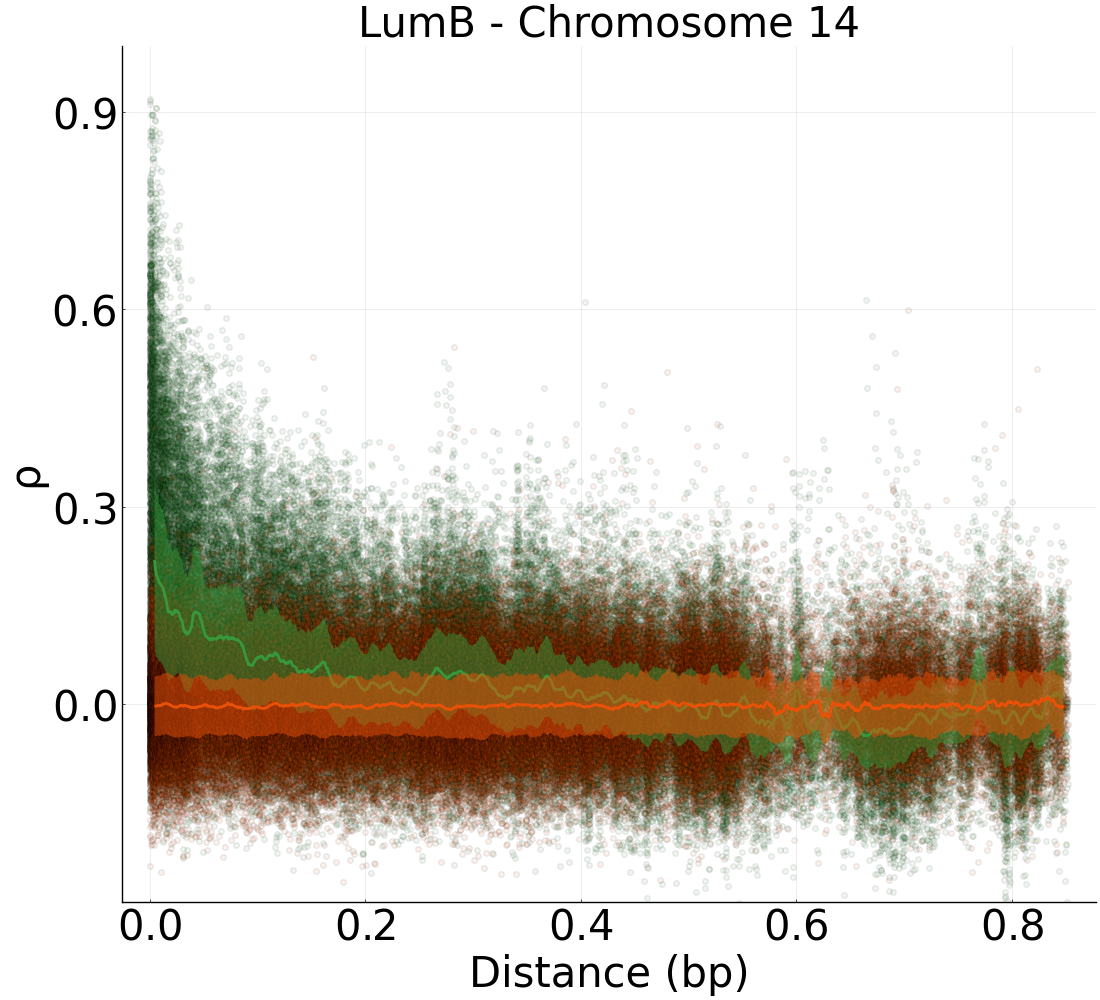

Supplement: Supplementary file 17 [file DataSheet_10.zip › SuppMat11Lumb/Chromosome-14-LumB.png]

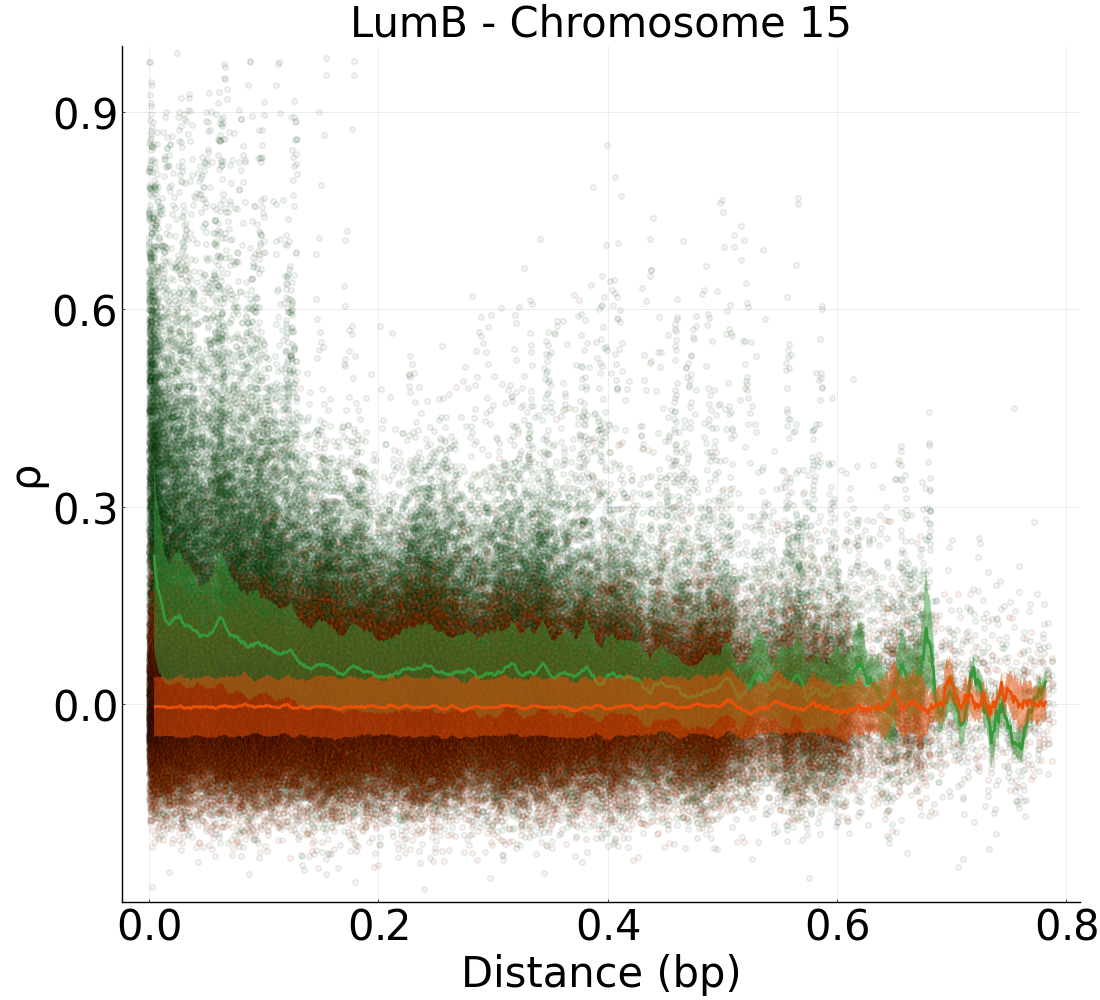

Supplement: Supplementary file 17 [file DataSheet_10.zip › SuppMat11Lumb/Chromosome-15-LumB.png]

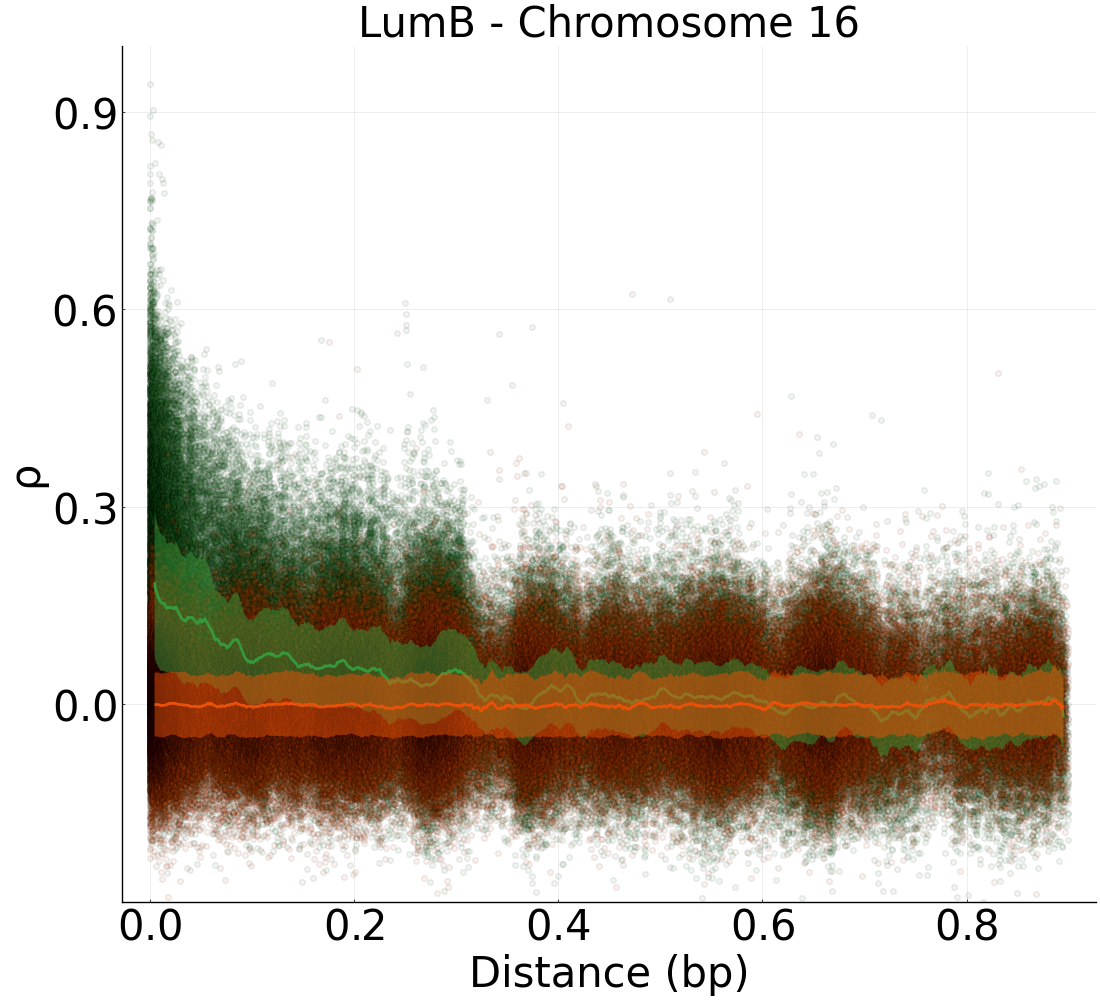

Supplement: Supplementary file 17 [file DataSheet_10.zip › SuppMat11Lumb/Chromosome-16-LumB.png]

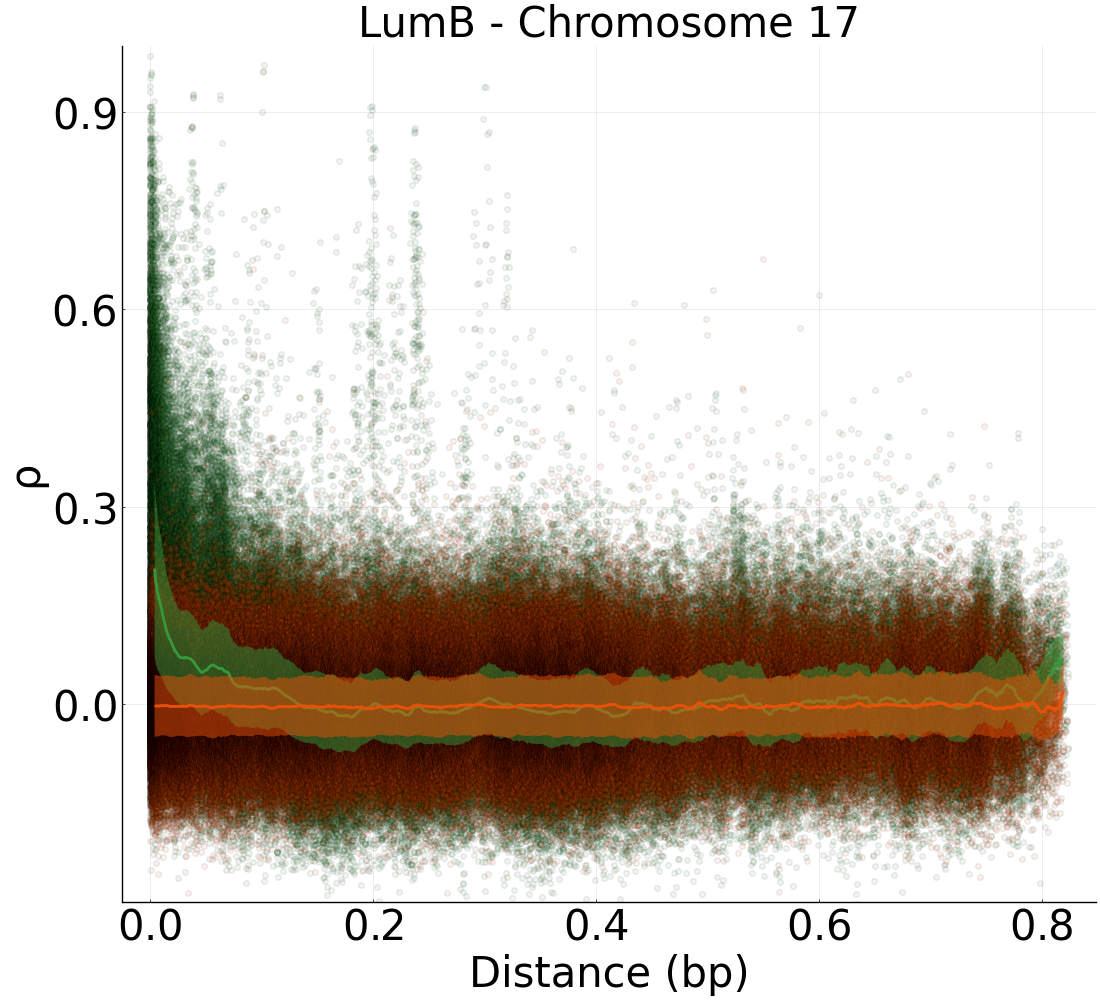

Supplement: Supplementary file 17 [file DataSheet_10.zip › SuppMat11Lumb/Chromosome-17-LumB.png]

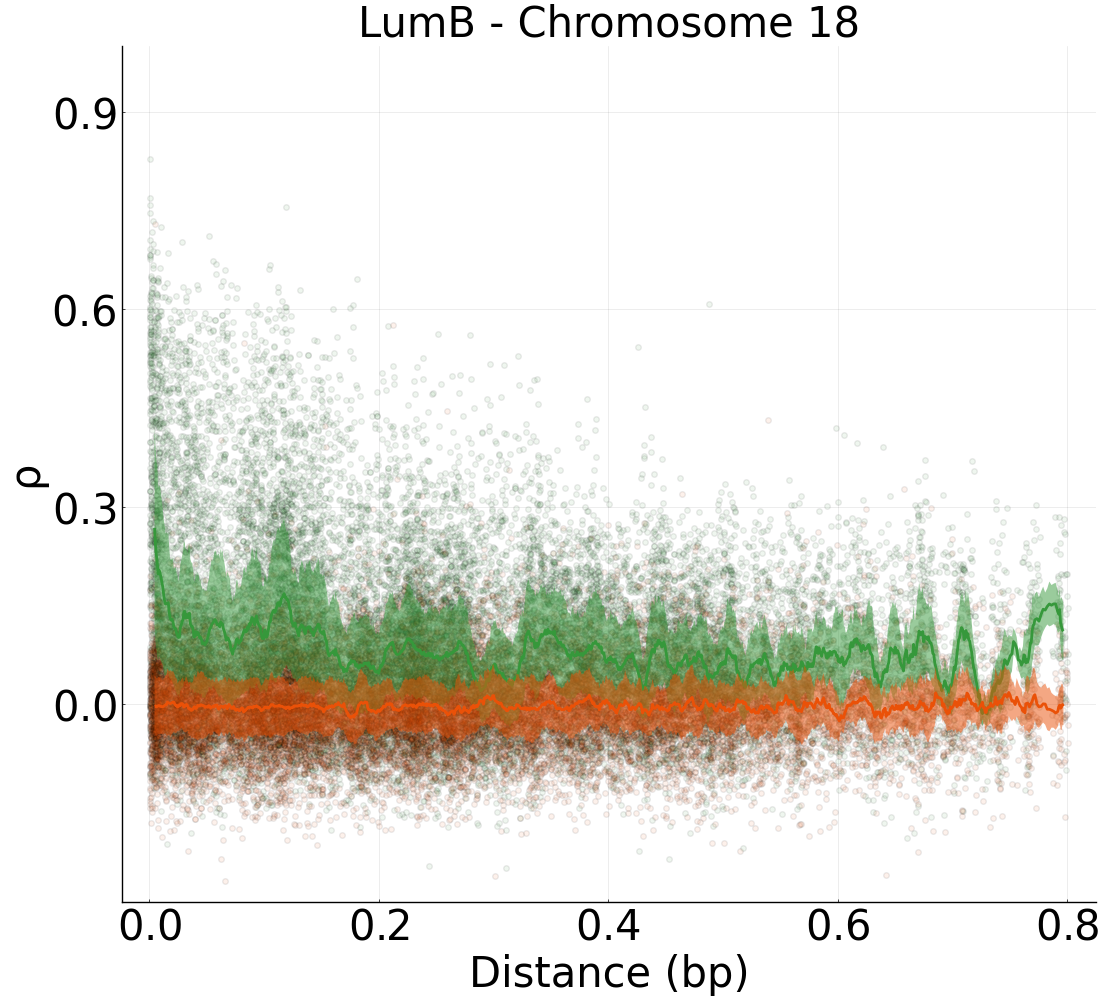

Supplement: Supplementary file 17 [file DataSheet_10.zip › SuppMat11Lumb/Chromosome-18-LumB.png]

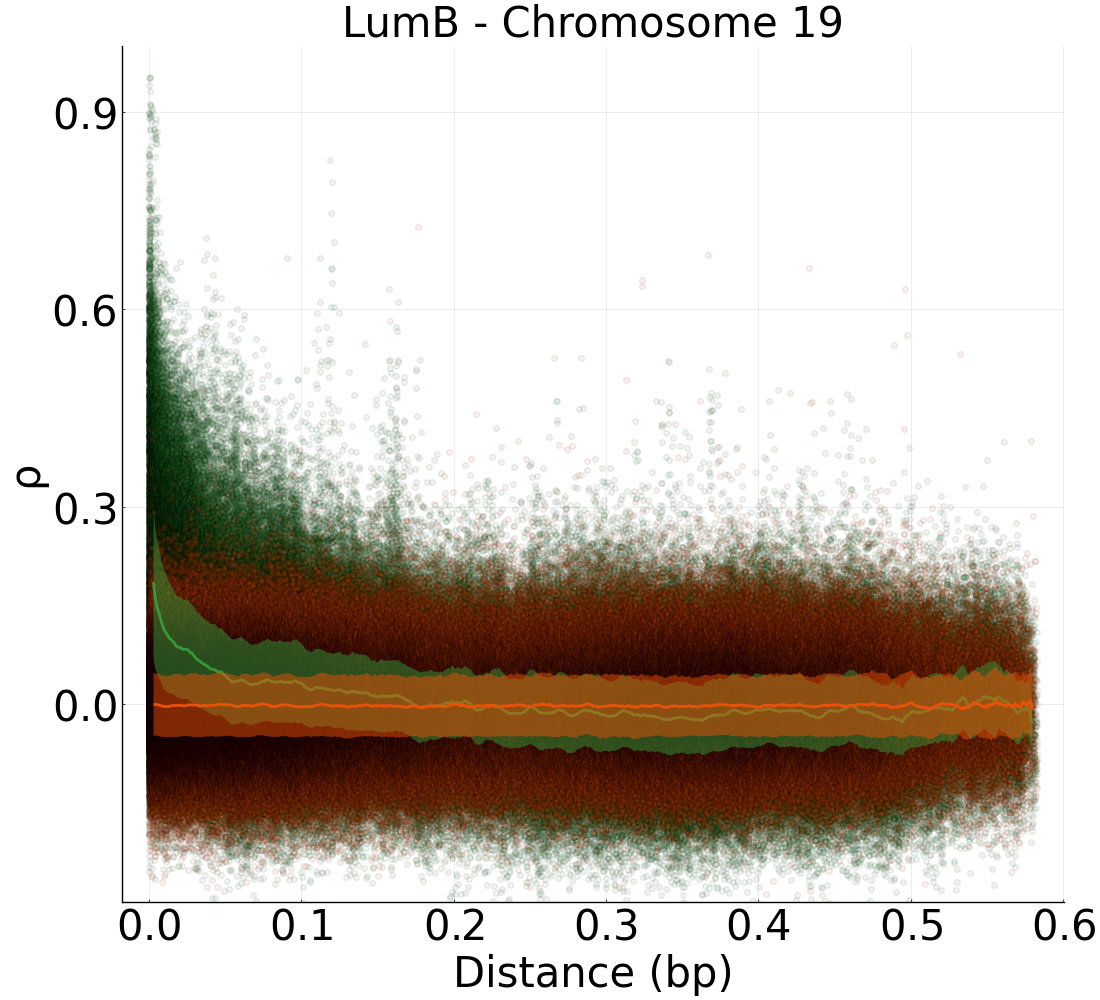

Supplement: Supplementary file 17 [file DataSheet_10.zip › SuppMat11Lumb/Chromosome-19-LumB.png]

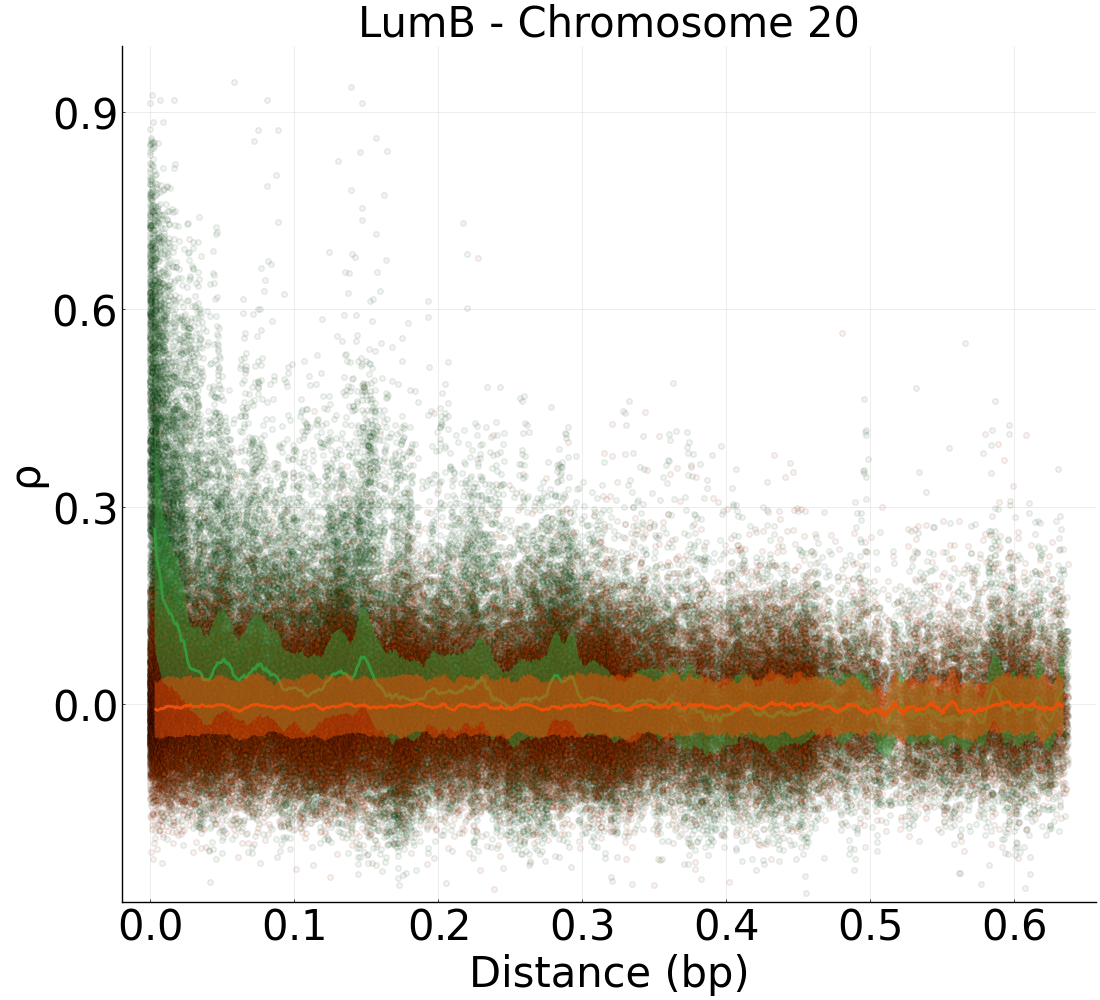

Supplement: Supplementary file 17 [file DataSheet_10.zip › SuppMat11Lumb/Chromosome-20-LumB.png]

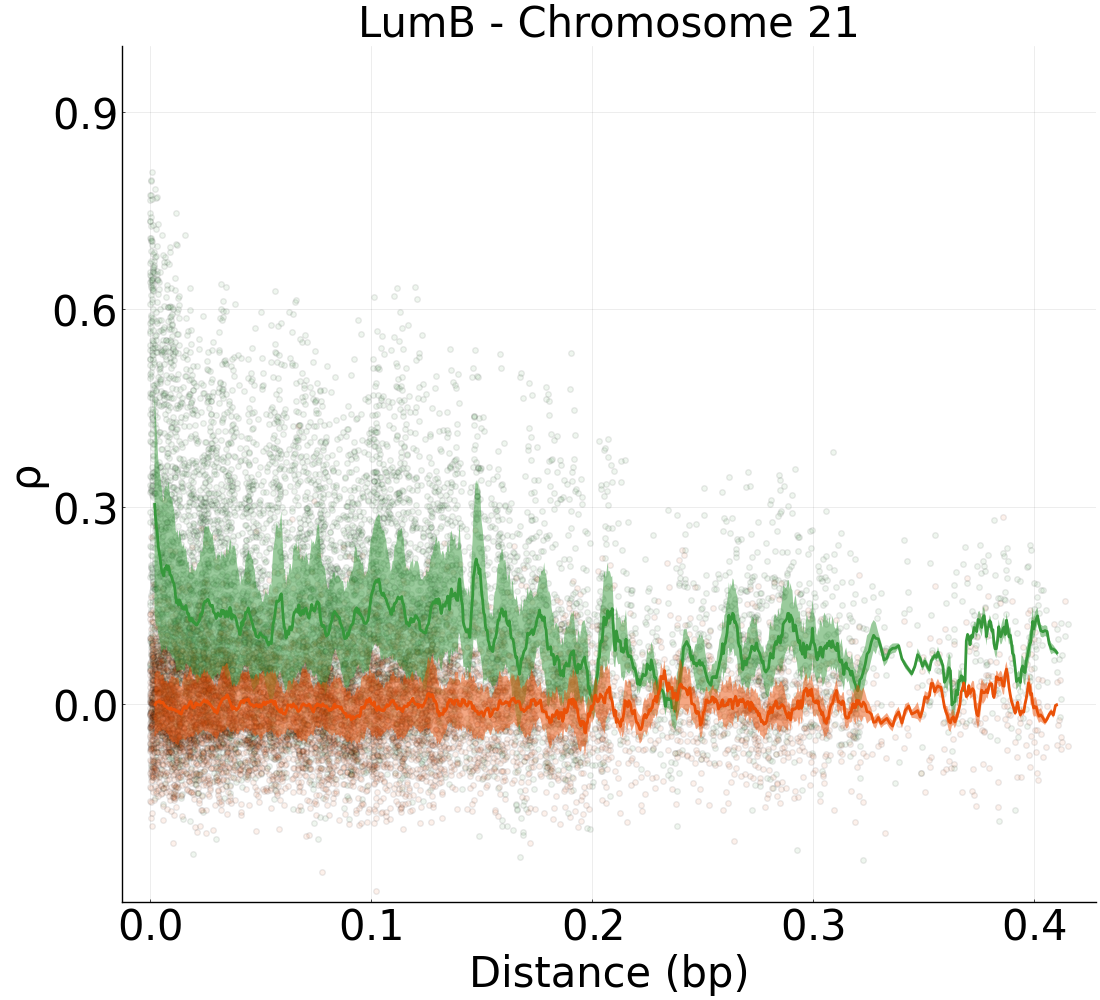

Supplement: Supplementary file 17 [file DataSheet_10.zip › SuppMat11Lumb/Chromosome-21-LumB.png]

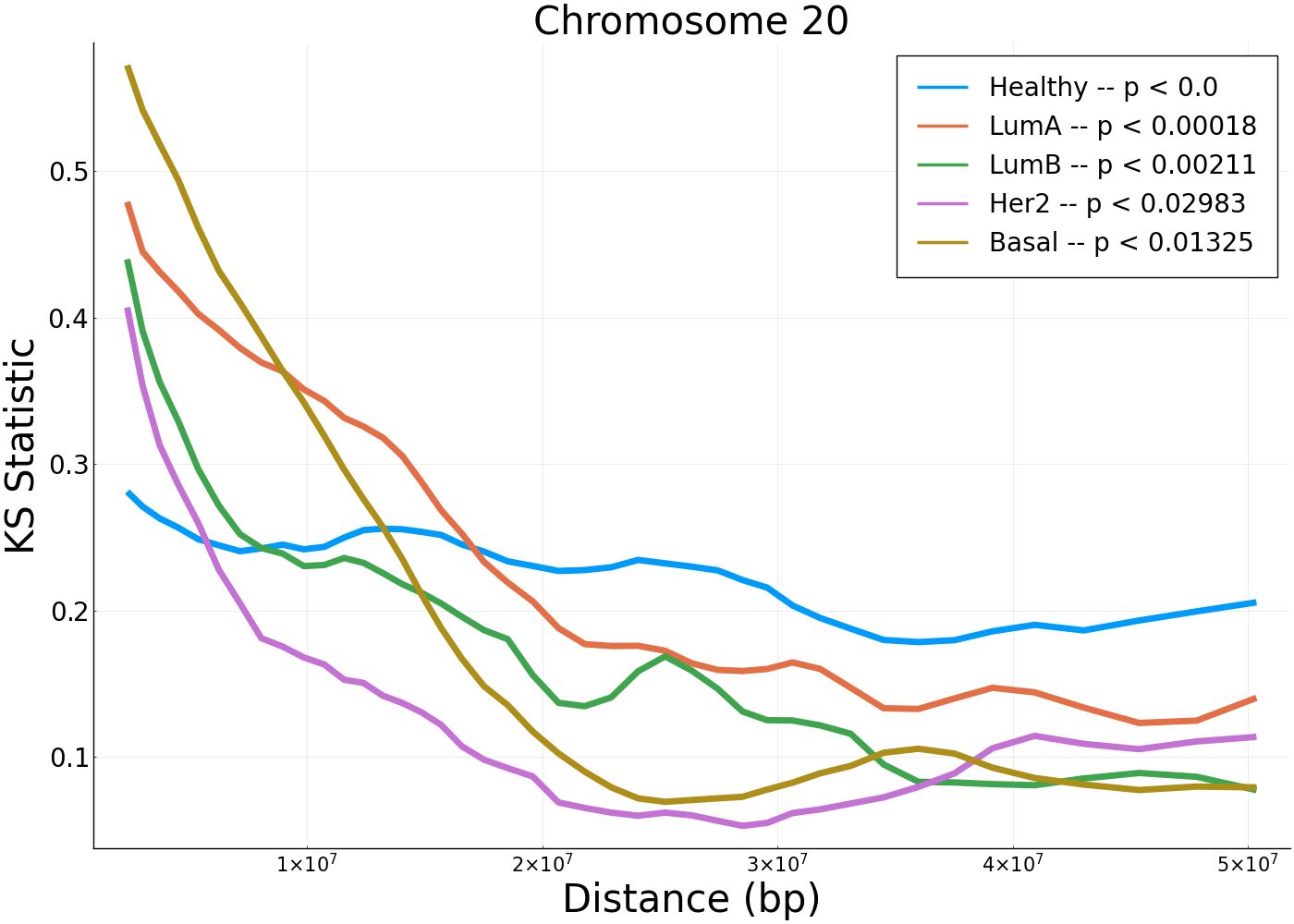

Supplement: Supplementary file 18 [file DataSheet_11.zip › SuppMat8/SuppMat8/KS-Test-Chromosome-20.png]

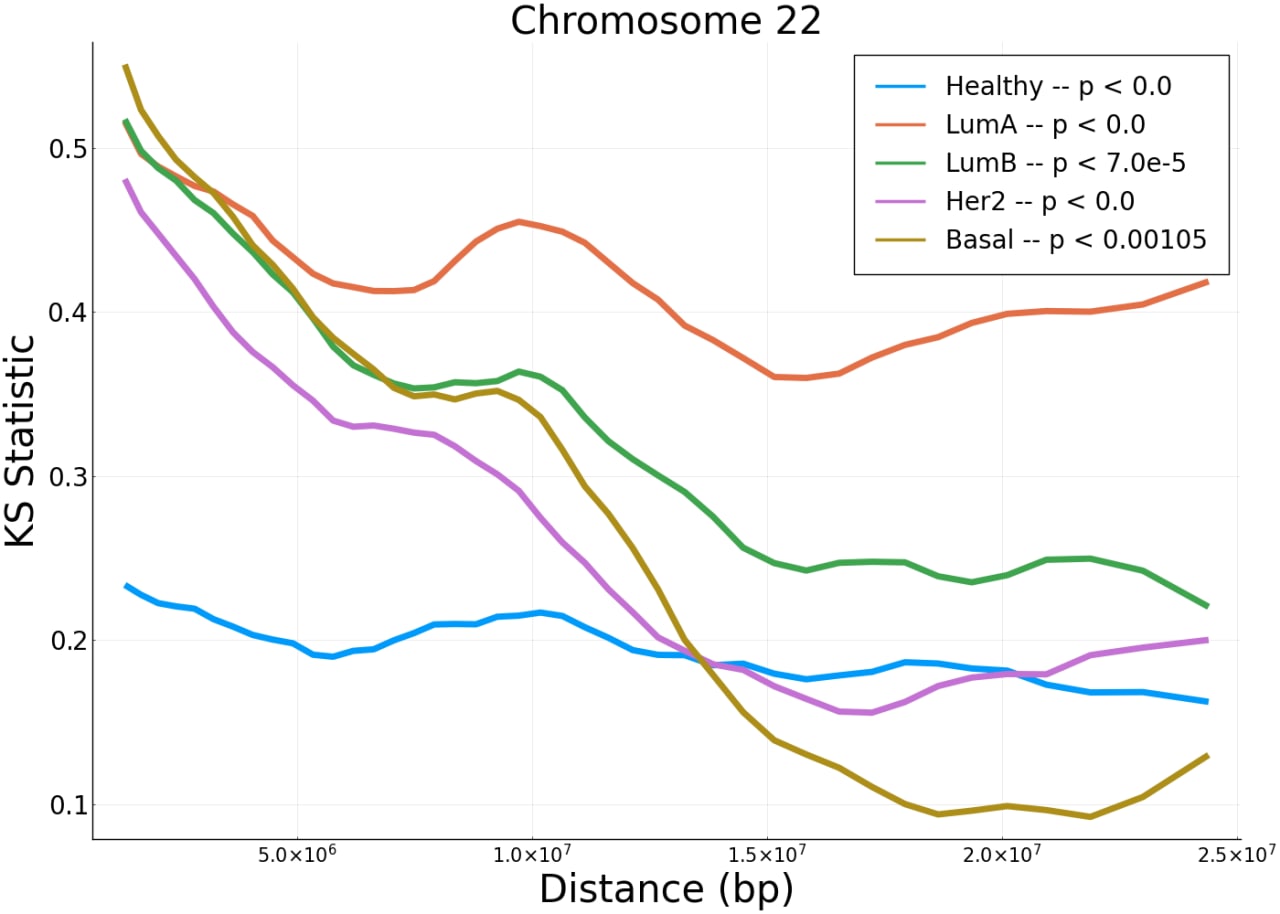

Supplement: Supplementary file 18 [file DataSheet_11.zip › SuppMat8/SuppMat8/KS-Test-Chromosome-22.jpeg]

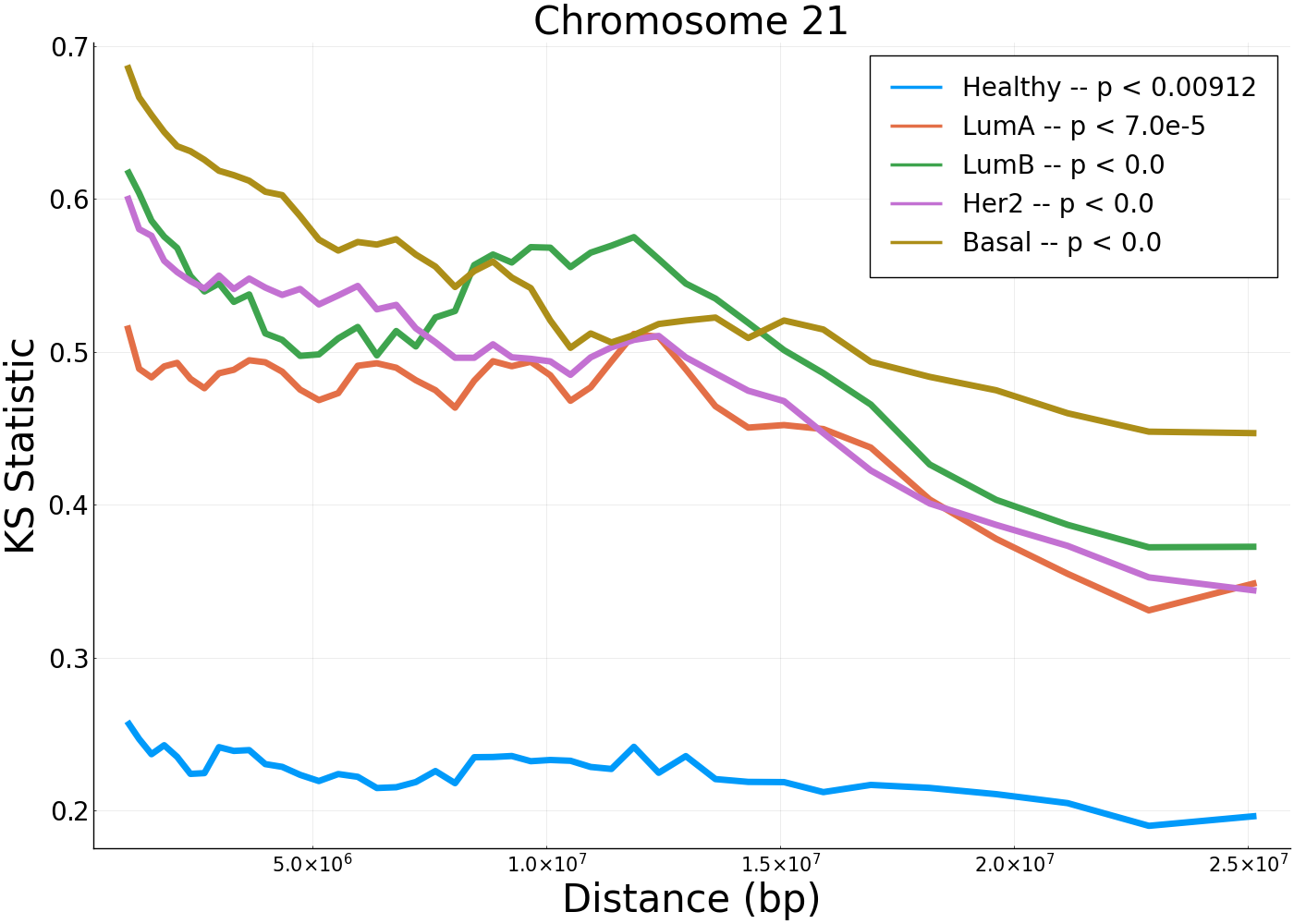

Supplement: Supplementary file 18 [file DataSheet_11.zip › SuppMat8/SuppMat8/KS-Test-Chromosome-21.png]

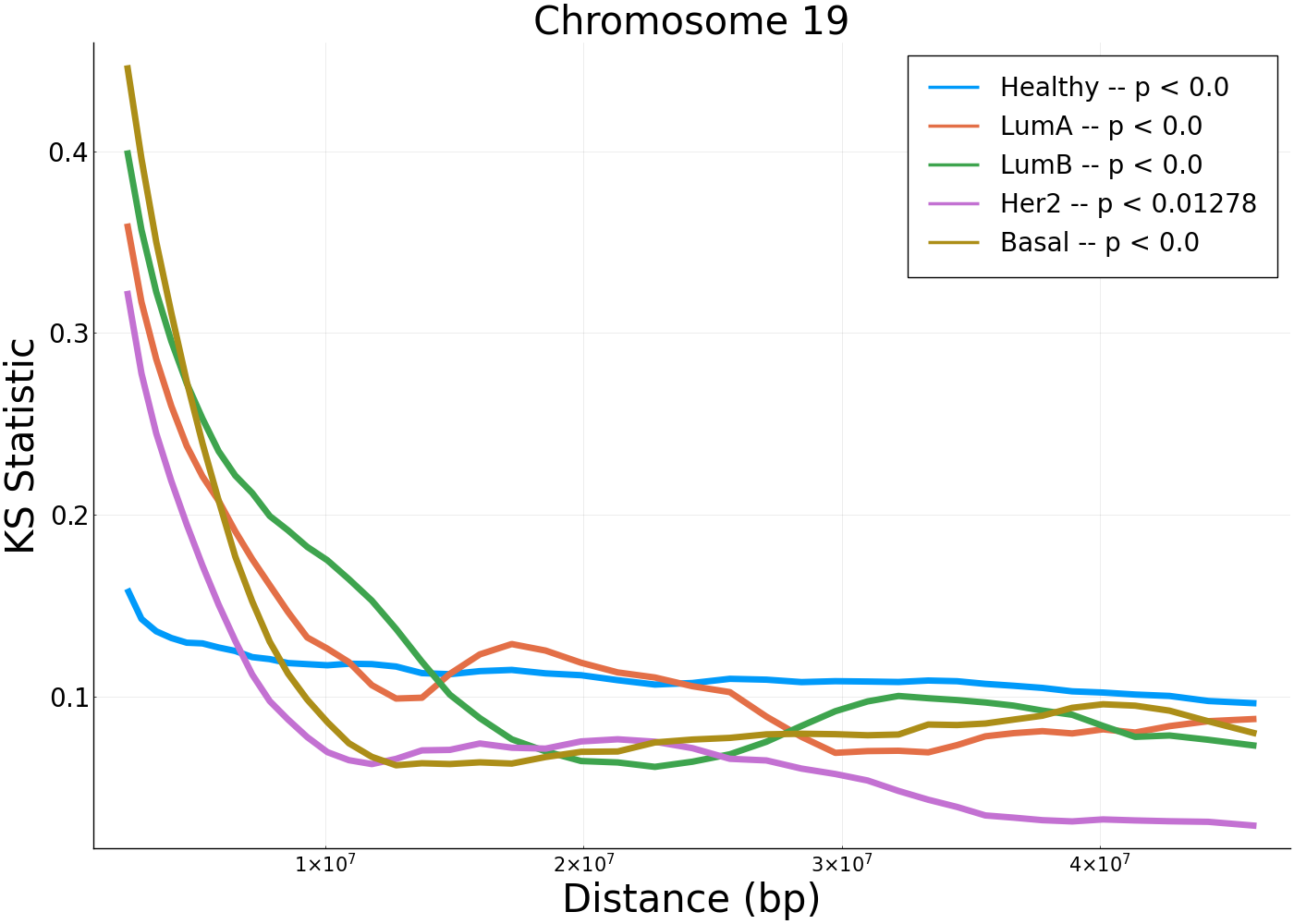

Supplement: Supplementary file 18 [file DataSheet_11.zip › SuppMat8/SuppMat8/KS-Test-Chromosome-19.png]

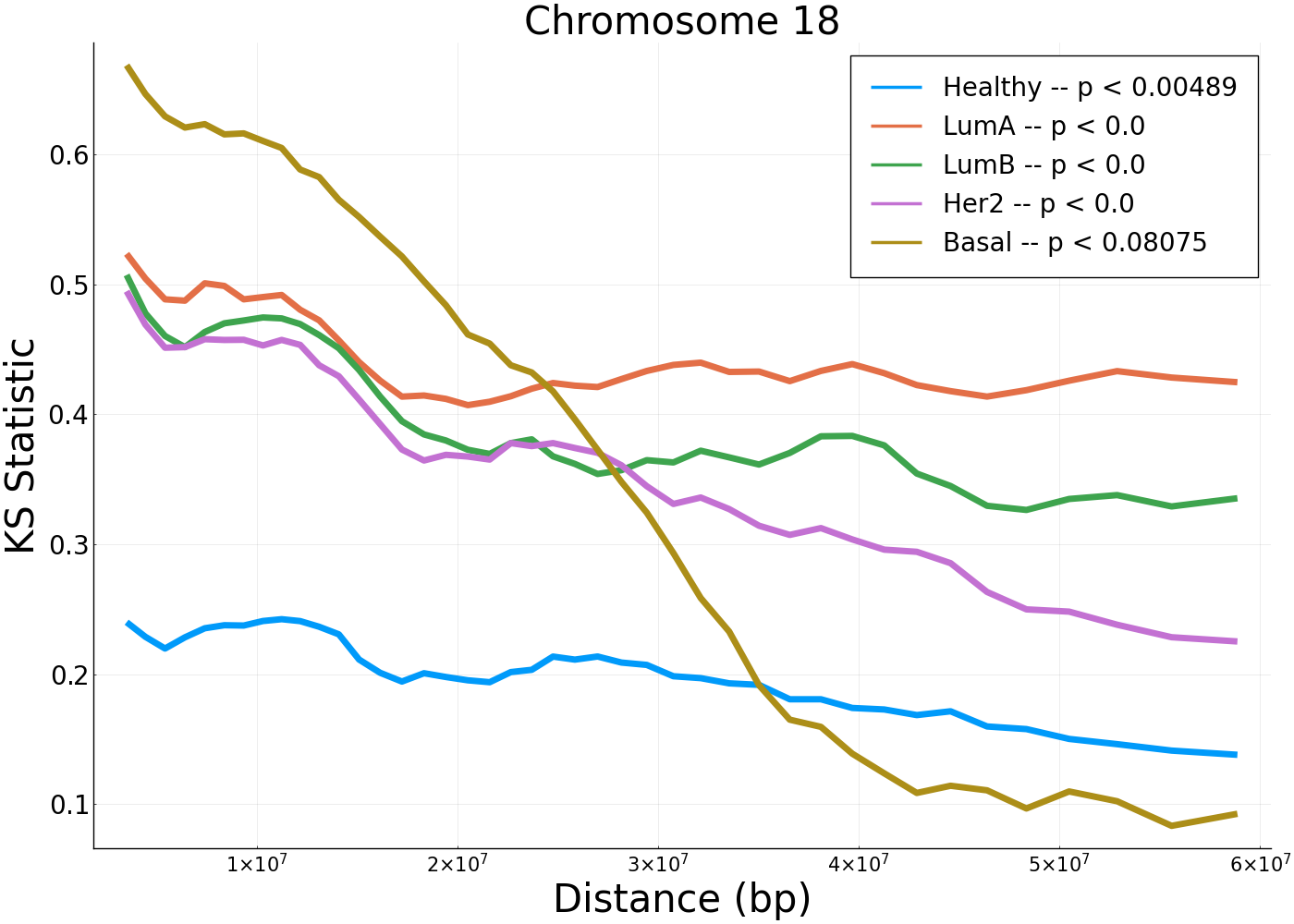

Supplement: Supplementary file 18 [file DataSheet_11.zip › SuppMat8/SuppMat8/KS-Test-Chromosome-18.png]

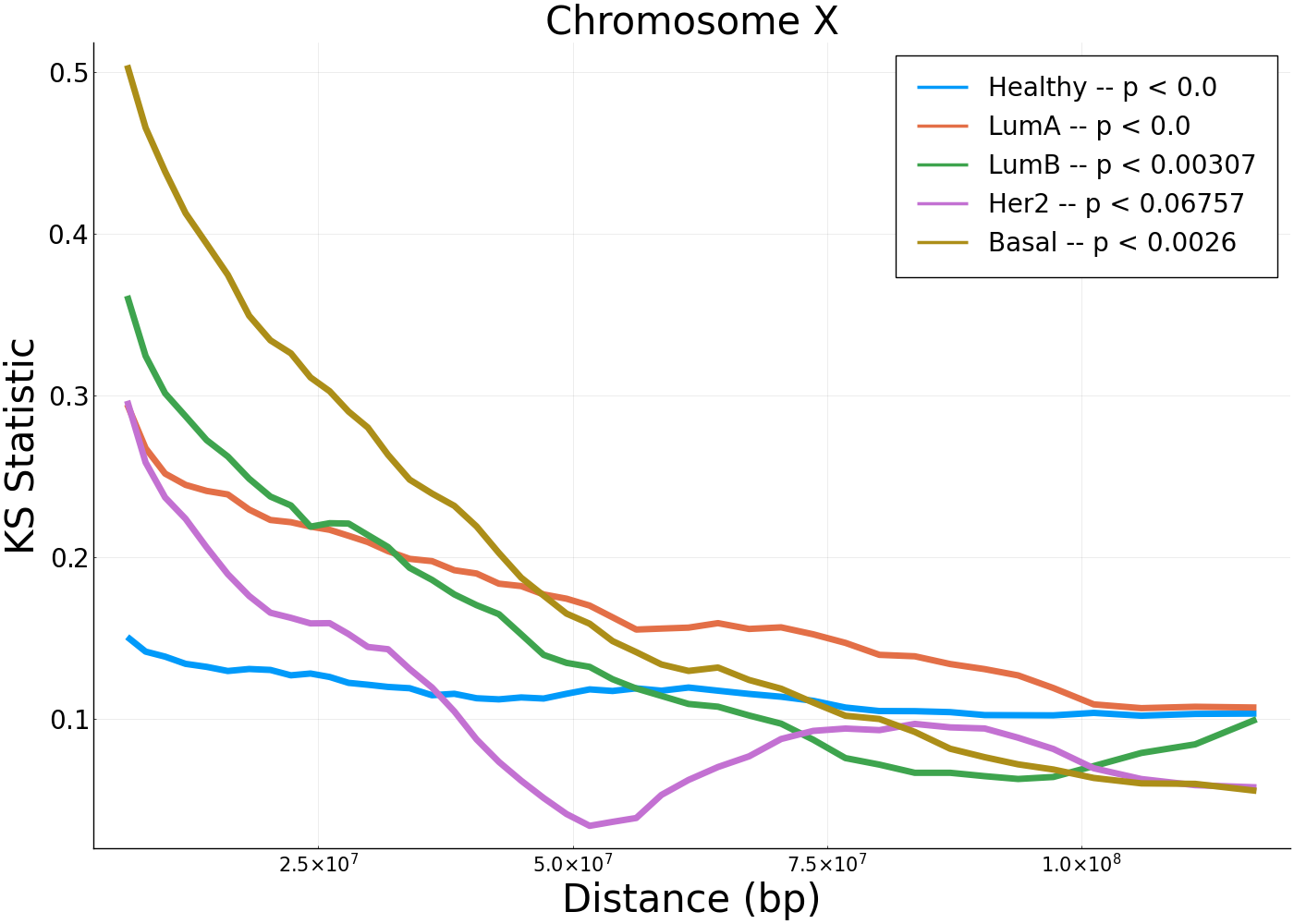

Supplement: Supplementary file 18 [file DataSheet_11.zip › SuppMat8/SuppMat8/KS-Test-Chromosome-X.png]

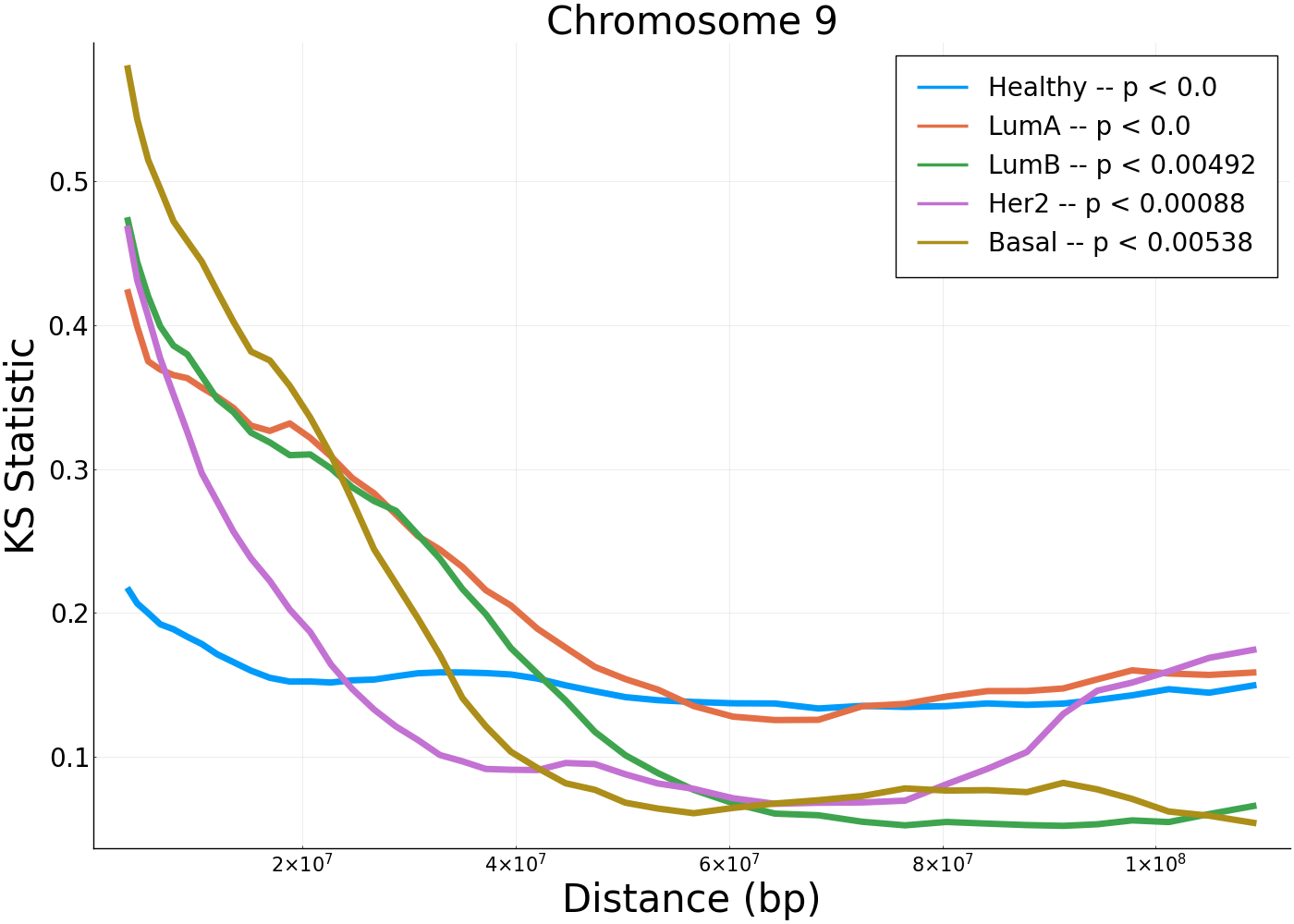

Supplement: Supplementary file 18 [file DataSheet_11.zip › SuppMat8/SuppMat8/KS-Test-Chromosome-9.png]

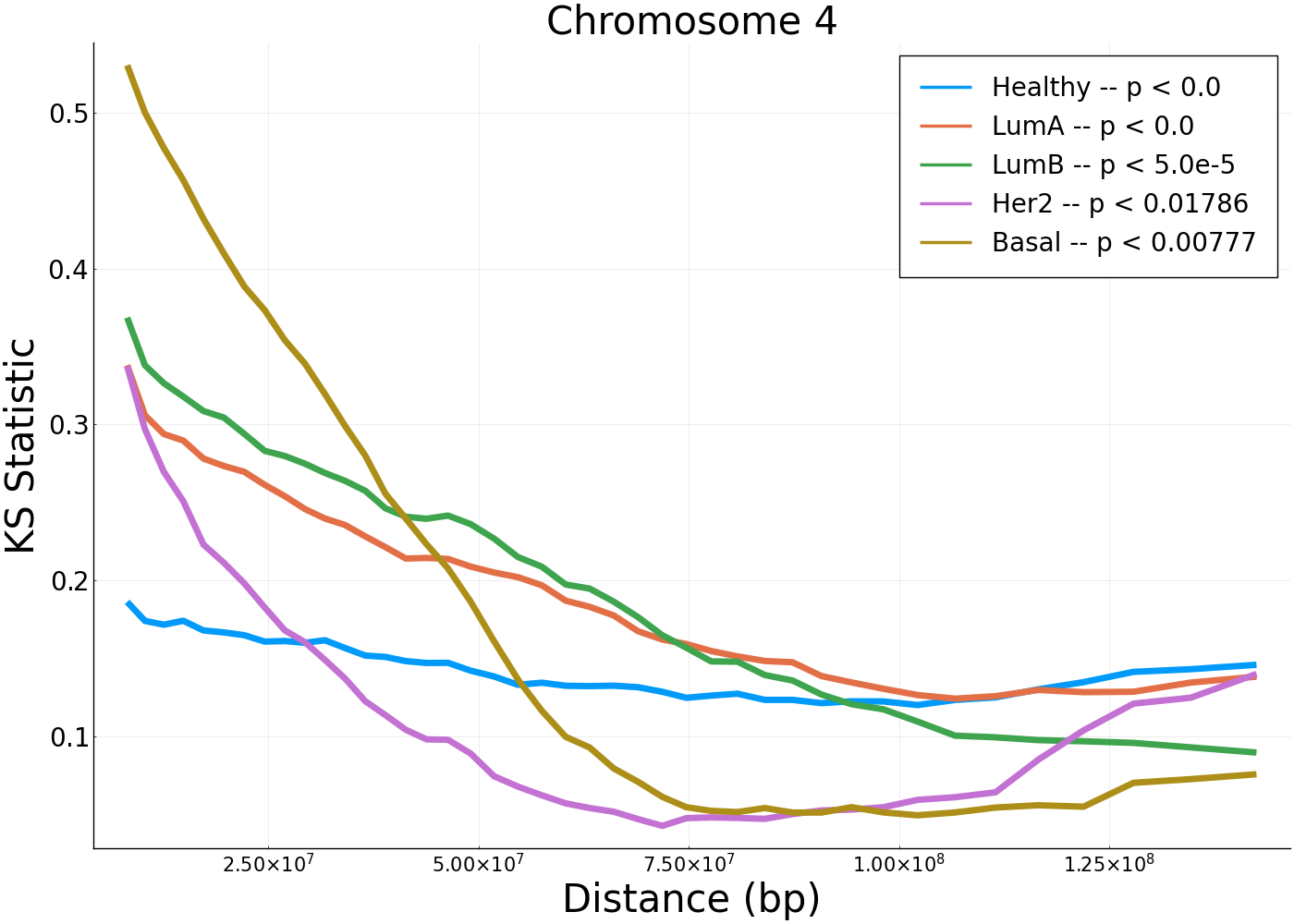

Supplement: Supplementary file 18 [file DataSheet_11.zip › SuppMat8/SuppMat8/KS-Test-Chromosome-4.png]

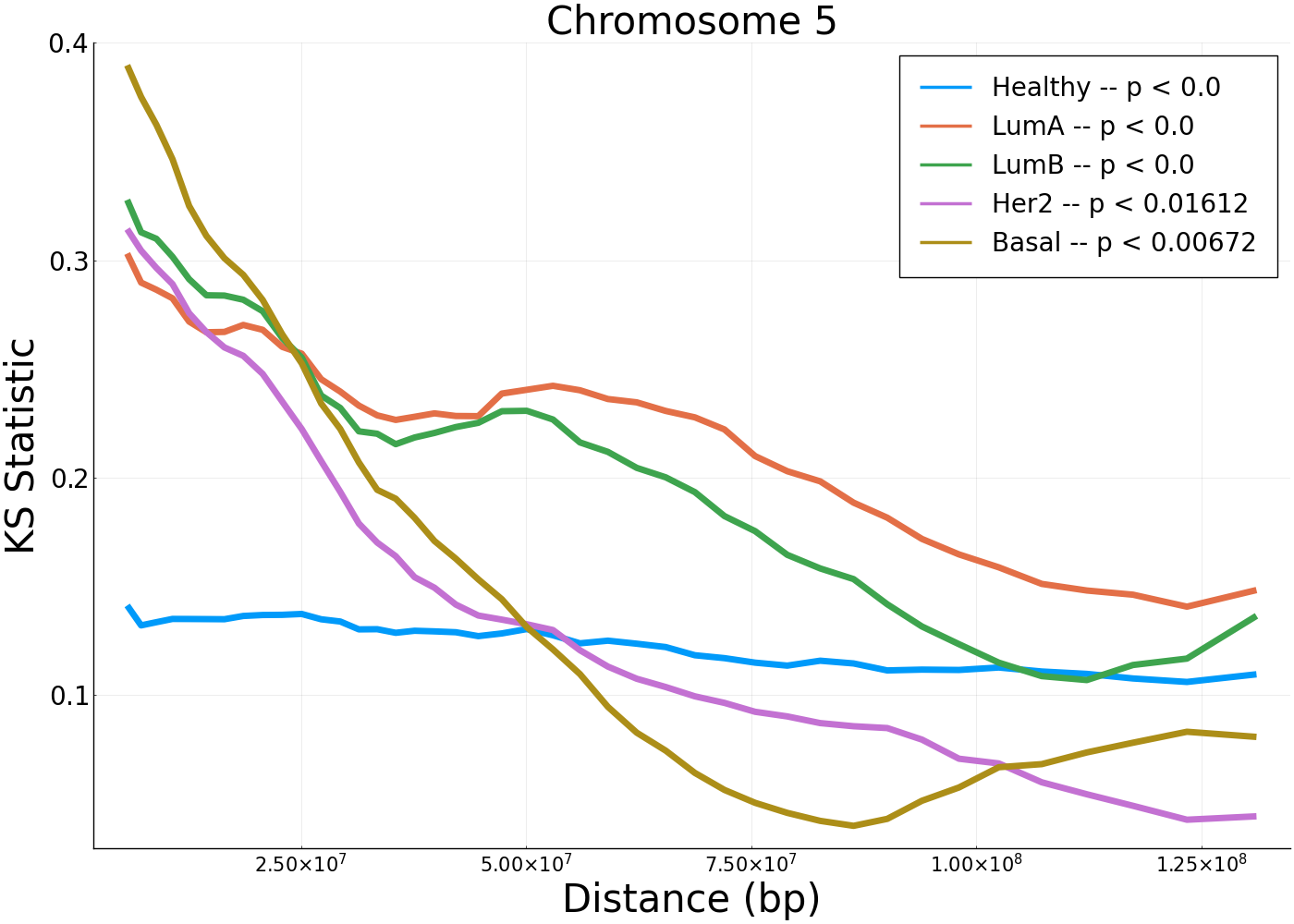

Supplement: Supplementary file 18 [file DataSheet_11.zip › SuppMat8/SuppMat8/KS-Test-Chromosome-5.png]

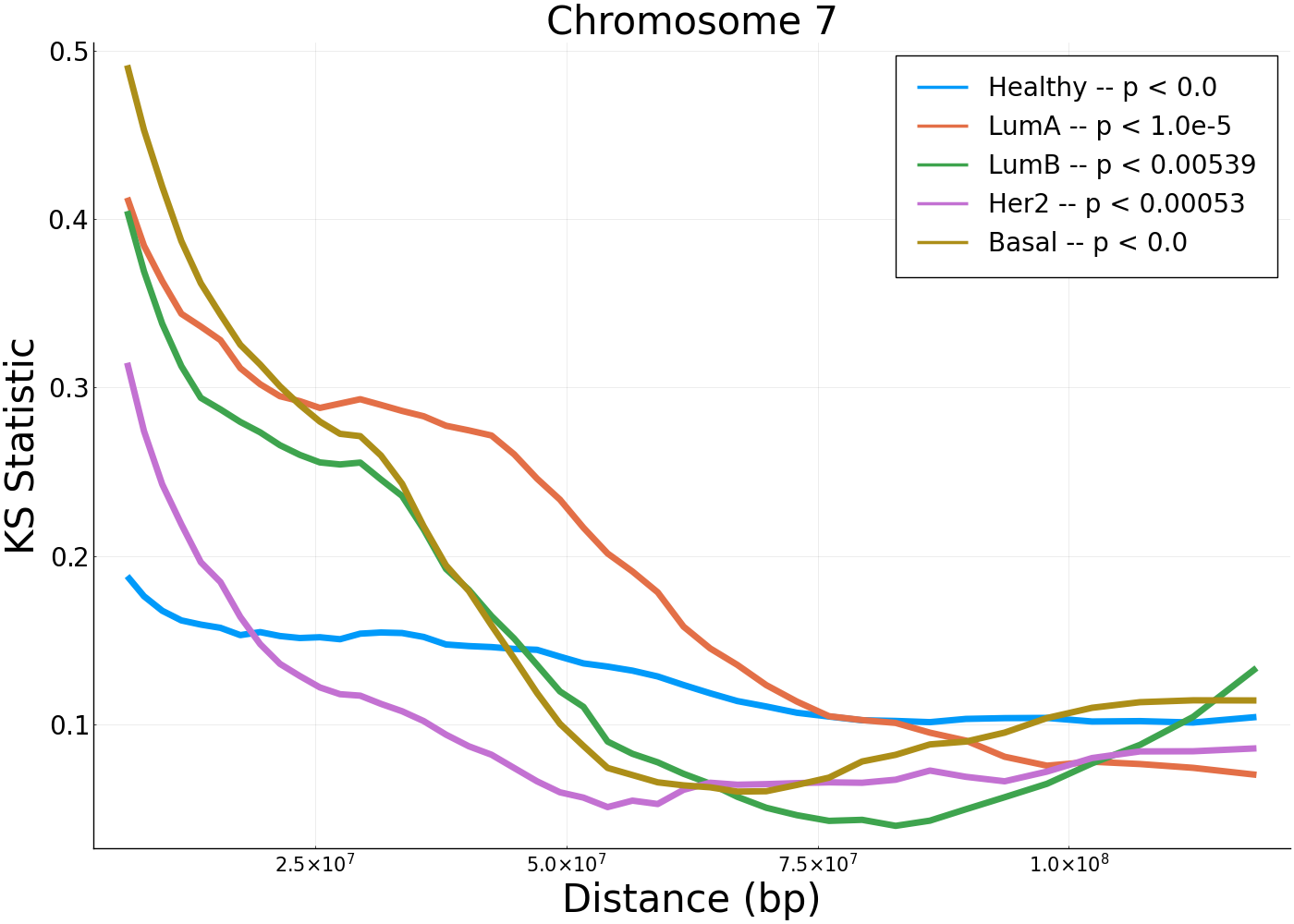

Supplement: Supplementary file 18 [file DataSheet_11.zip › SuppMat8/SuppMat8/KS-Test-Chromosome-7.png]

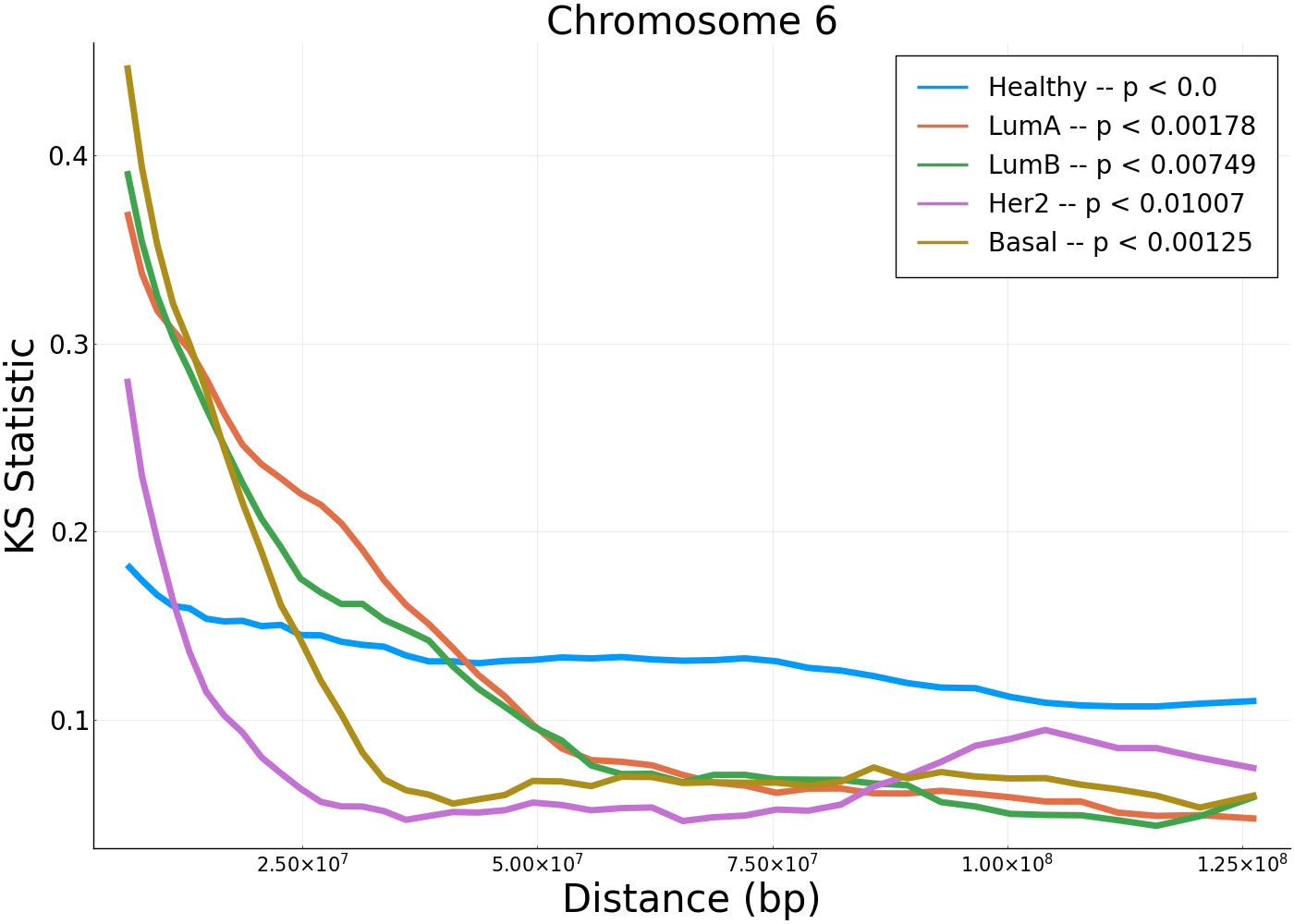

Supplement: Supplementary file 18 [file DataSheet_11.zip › SuppMat8/SuppMat8/KS-Test-Chromosome-6.png]

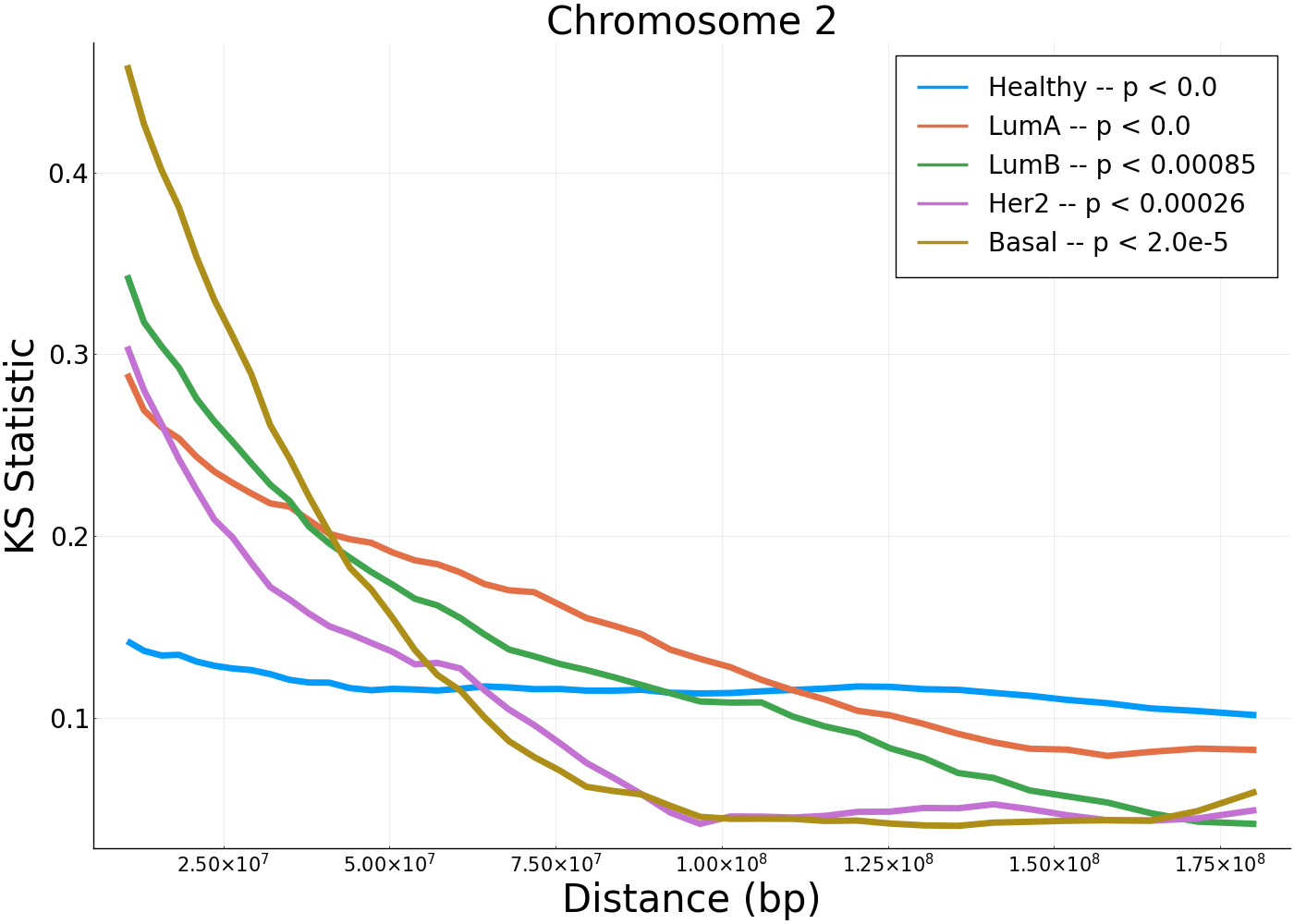

Supplement: Supplementary file 18 [file DataSheet_11.zip › SuppMat8/SuppMat8/KS-Test-Chromosome-2.png]

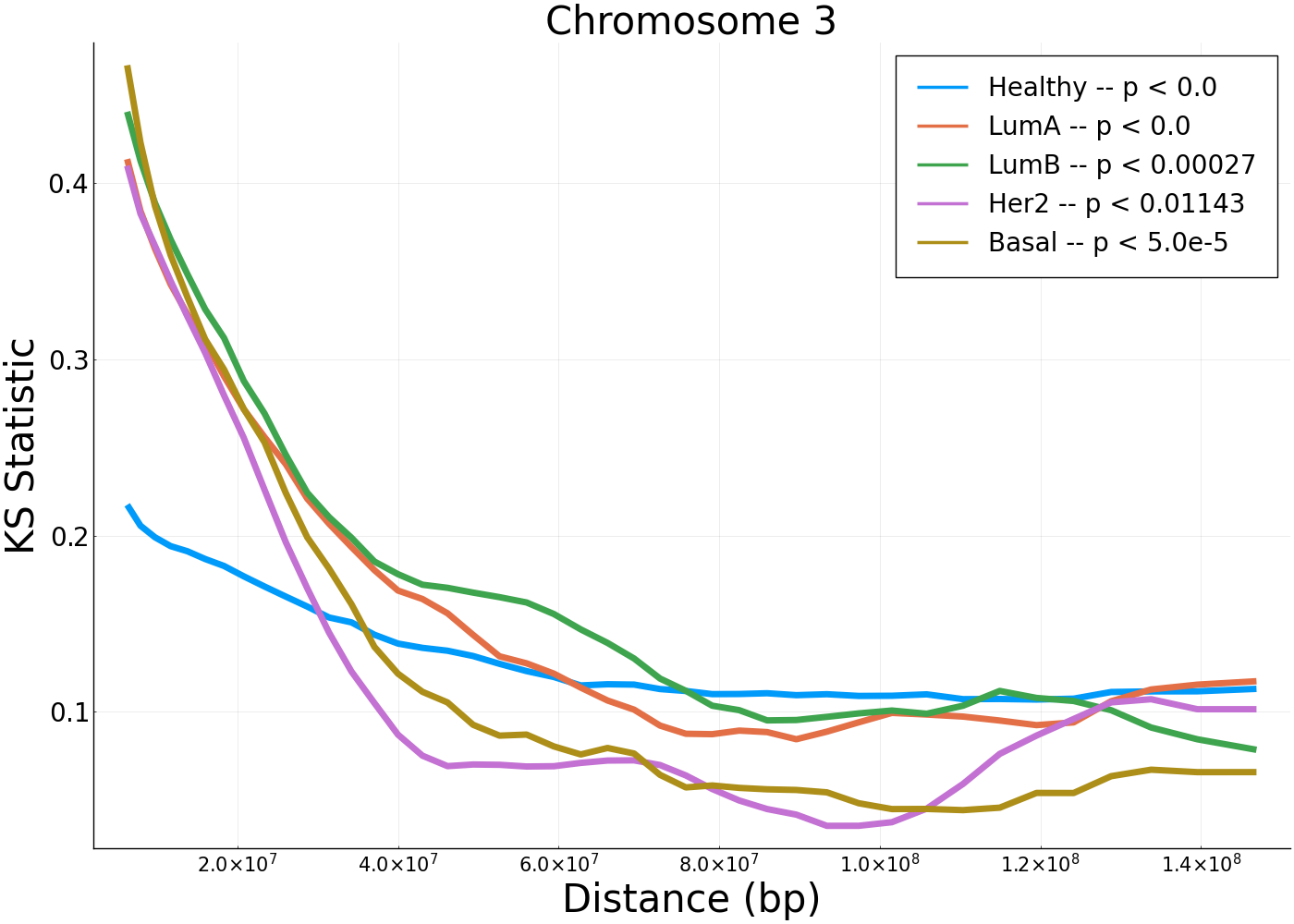

Supplement: Supplementary file 18 [file DataSheet_11.zip › SuppMat8/SuppMat8/KS-Test-Chromosome-3.png]

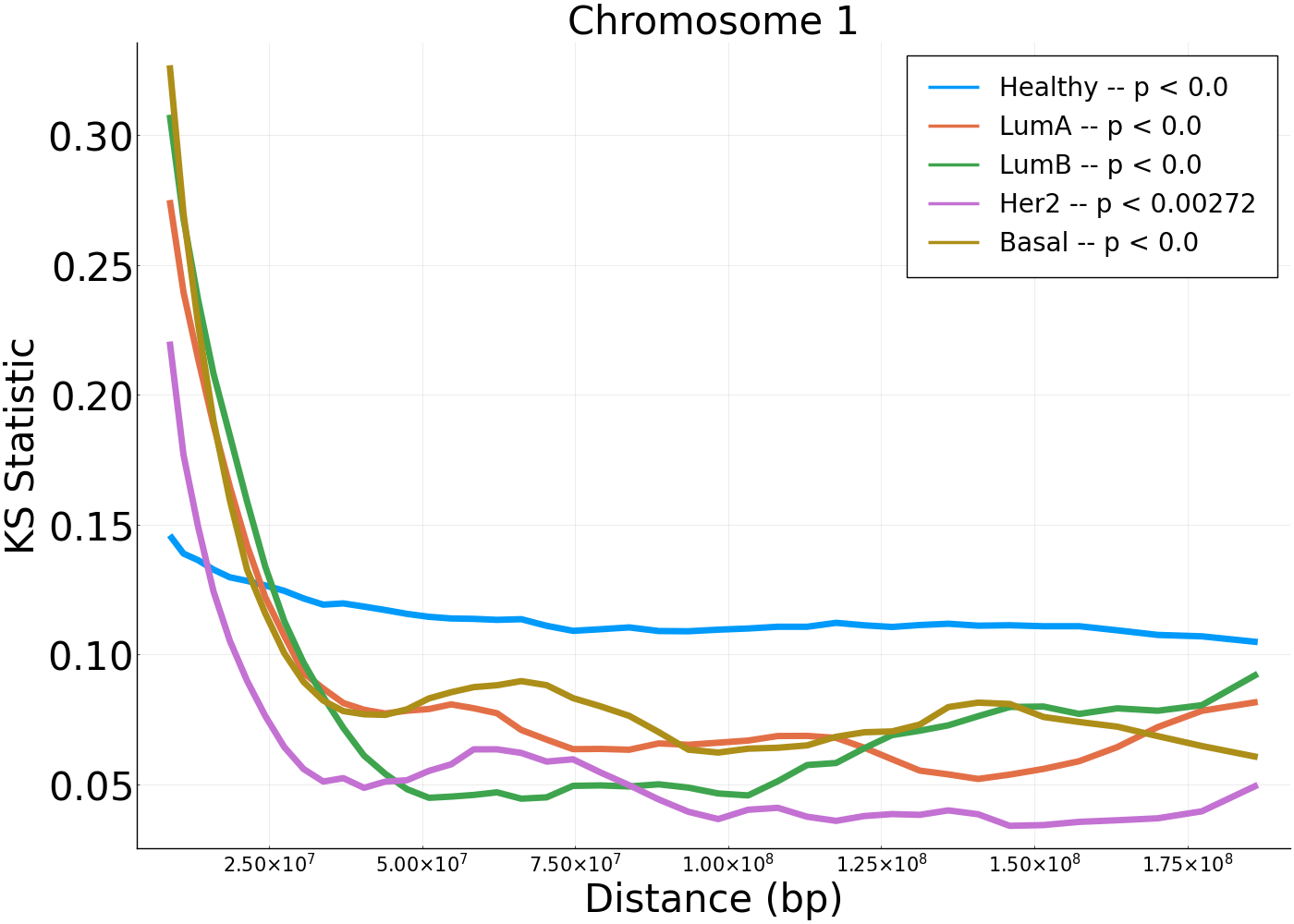

Supplement: Supplementary file 18 [file DataSheet_11.zip › SuppMat8/SuppMat8/KS-Test-Chromosome-1.png]

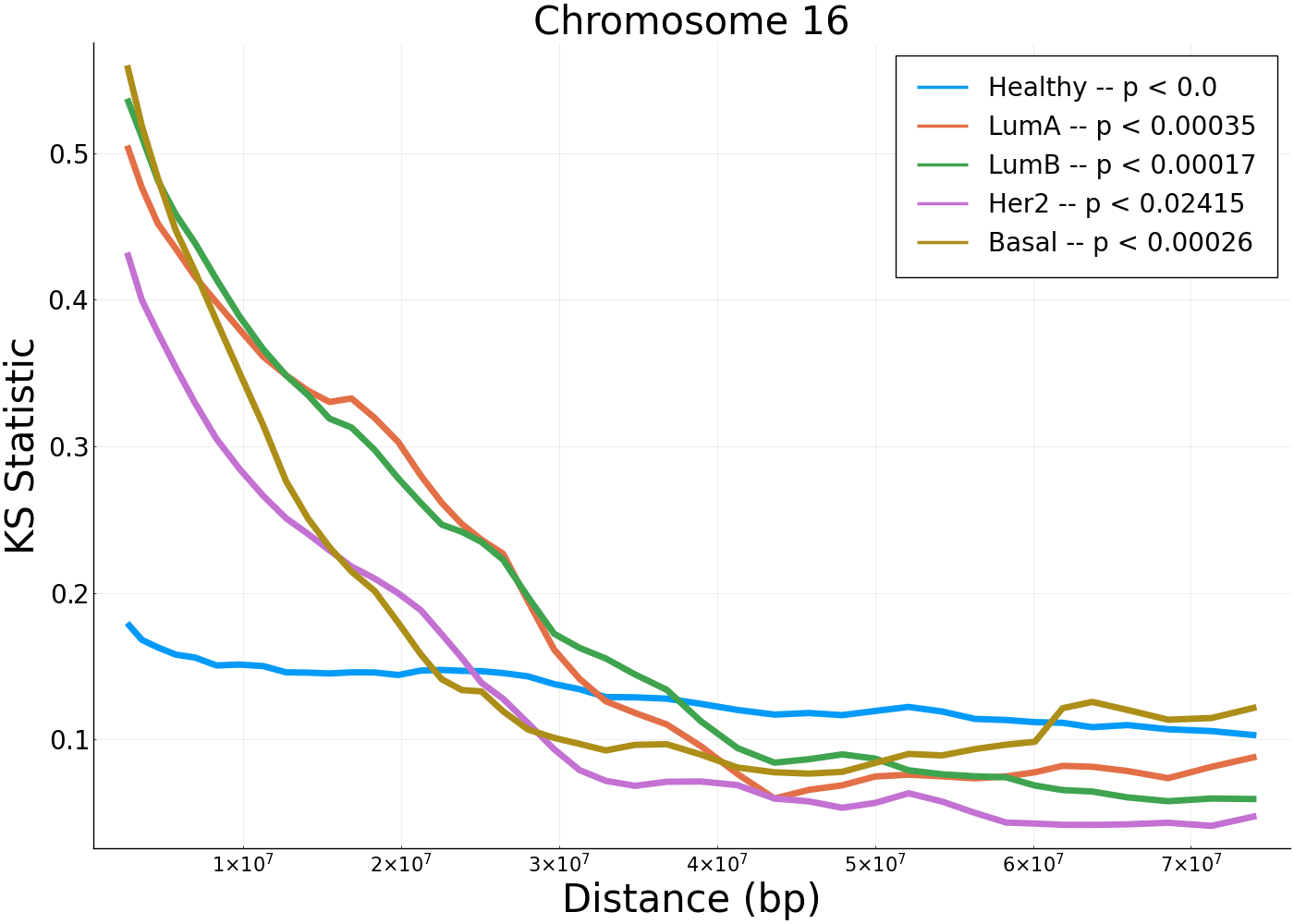

Supplement: Supplementary file 18 [file DataSheet_11.zip › SuppMat8/SuppMat8/KS-Test-Chromosome-16.png]

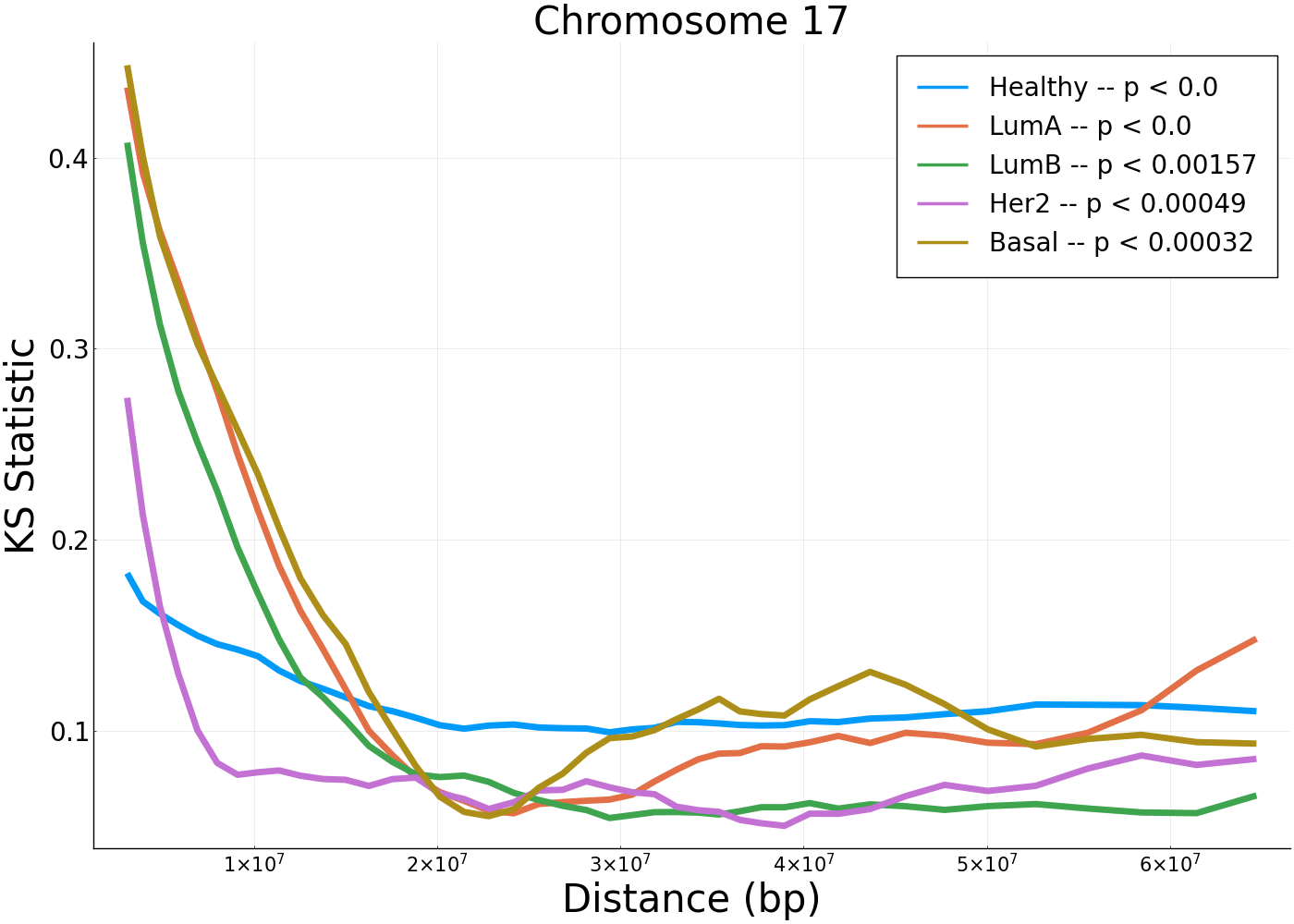

Supplement: Supplementary file 18 [file DataSheet_11.zip › SuppMat8/SuppMat8/KS-Test-Chromosome-17.png]

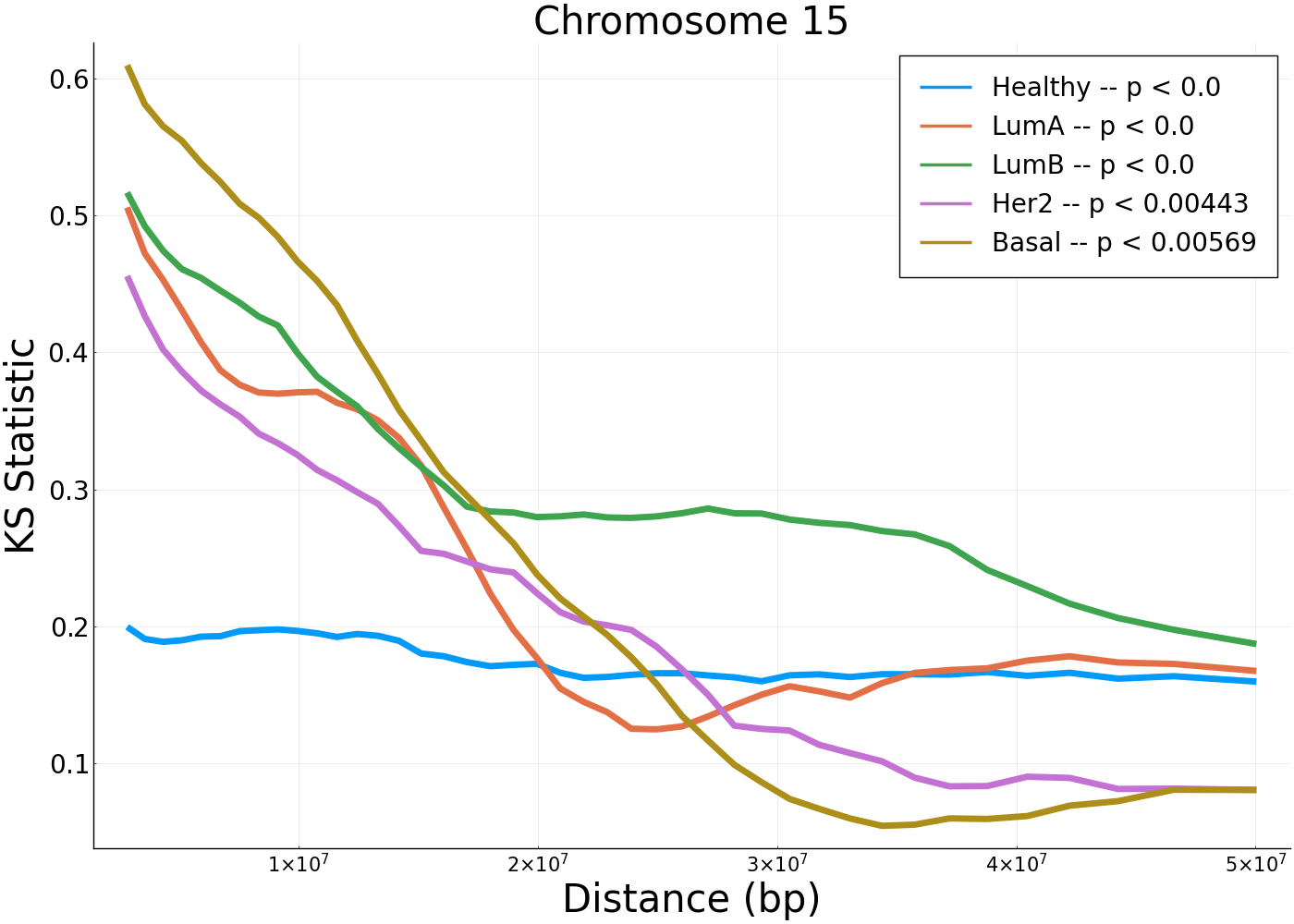

Supplement: Supplementary file 18 [file DataSheet_11.zip › SuppMat8/SuppMat8/KS-Test-Chromosome-15.png]

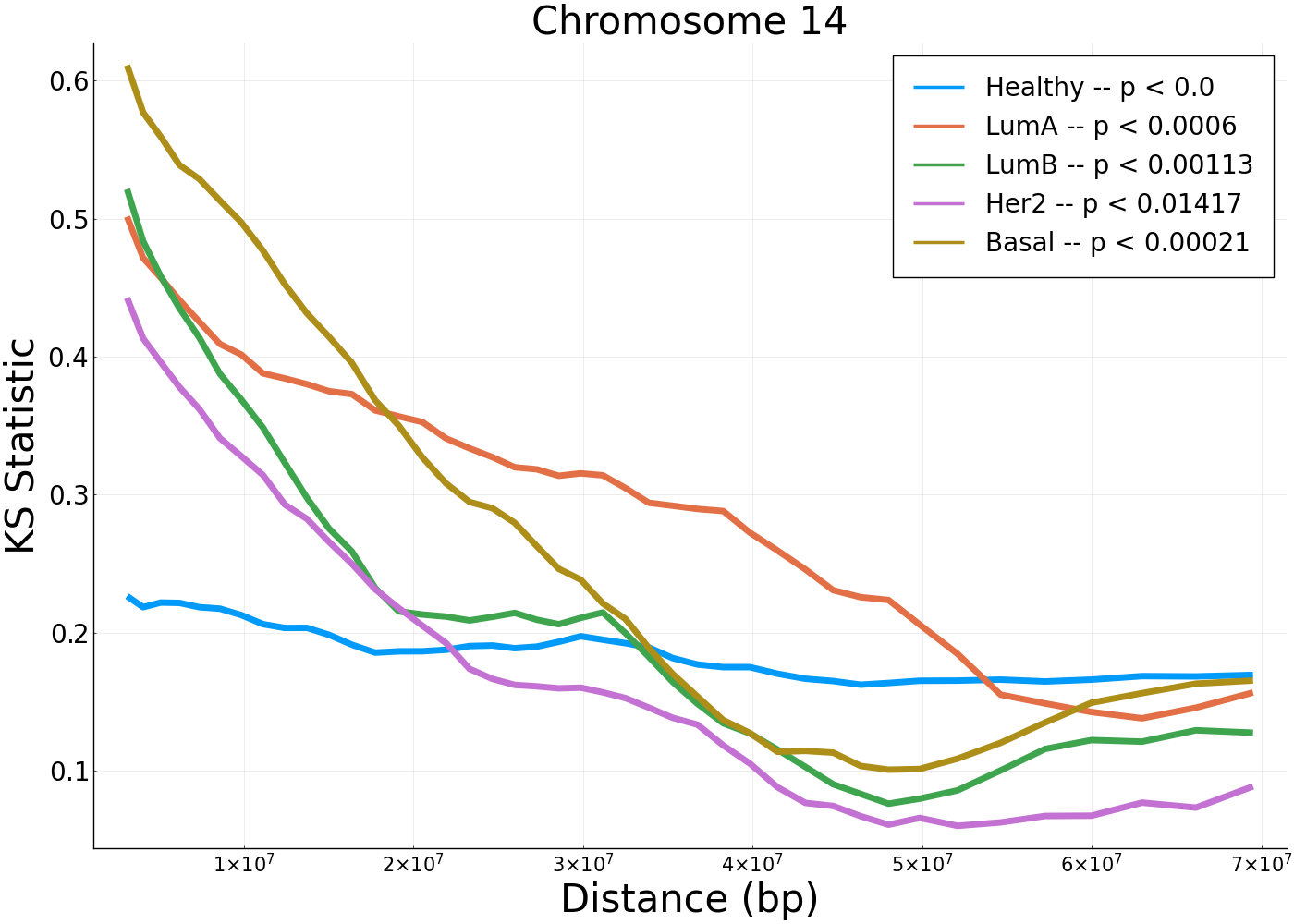

Supplement: Supplementary file 18 [file DataSheet_11.zip › SuppMat8/SuppMat8/KS-Test-Chromosome-14.png]

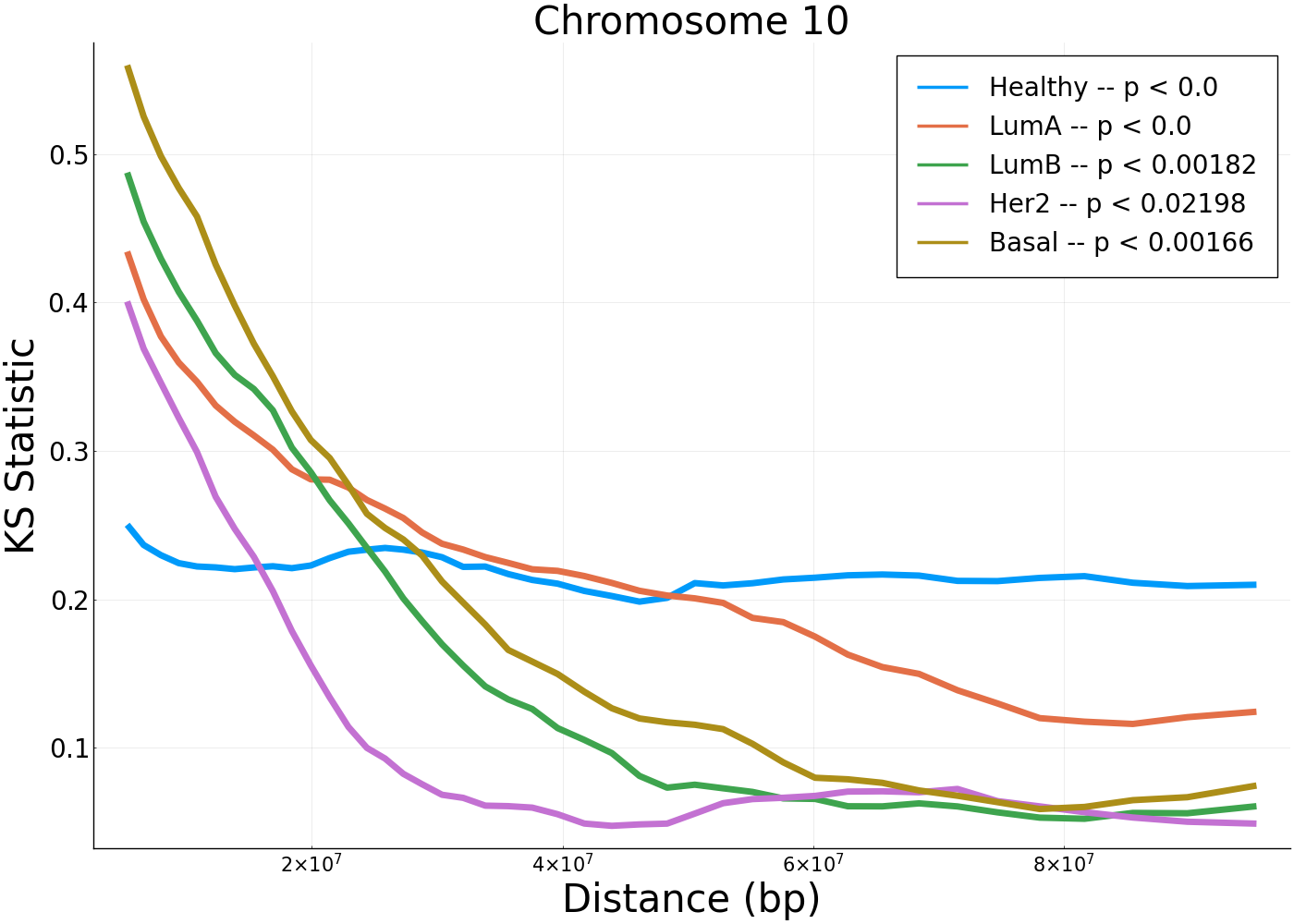

Supplement: Supplementary file 18 [file DataSheet_11.zip › SuppMat8/SuppMat8/KS-Test-Chromosome-10.png]

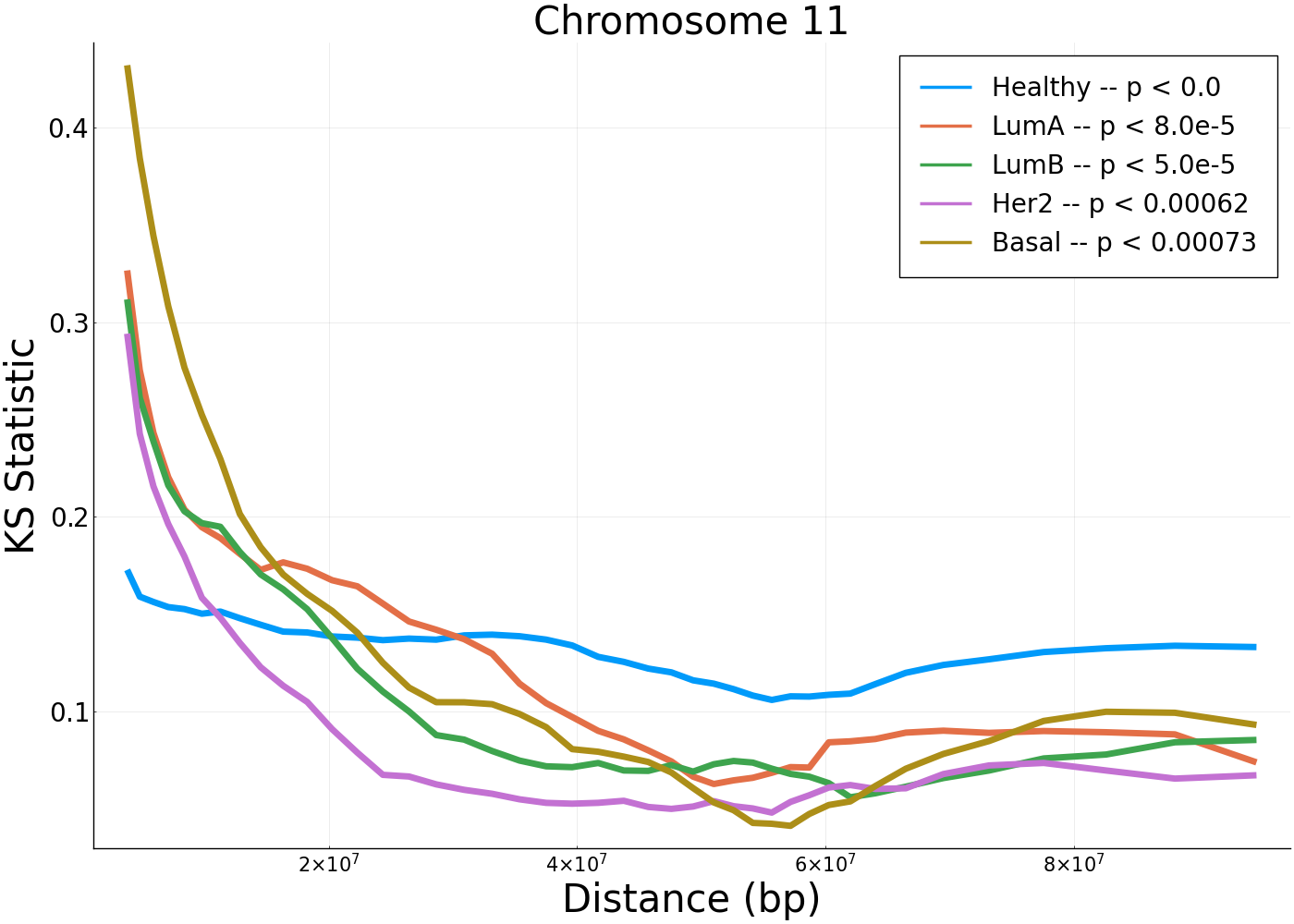

Supplement: Supplementary file 18 [file DataSheet_11.zip › SuppMat8/SuppMat8/KS-Test-Chromosome-11.png]

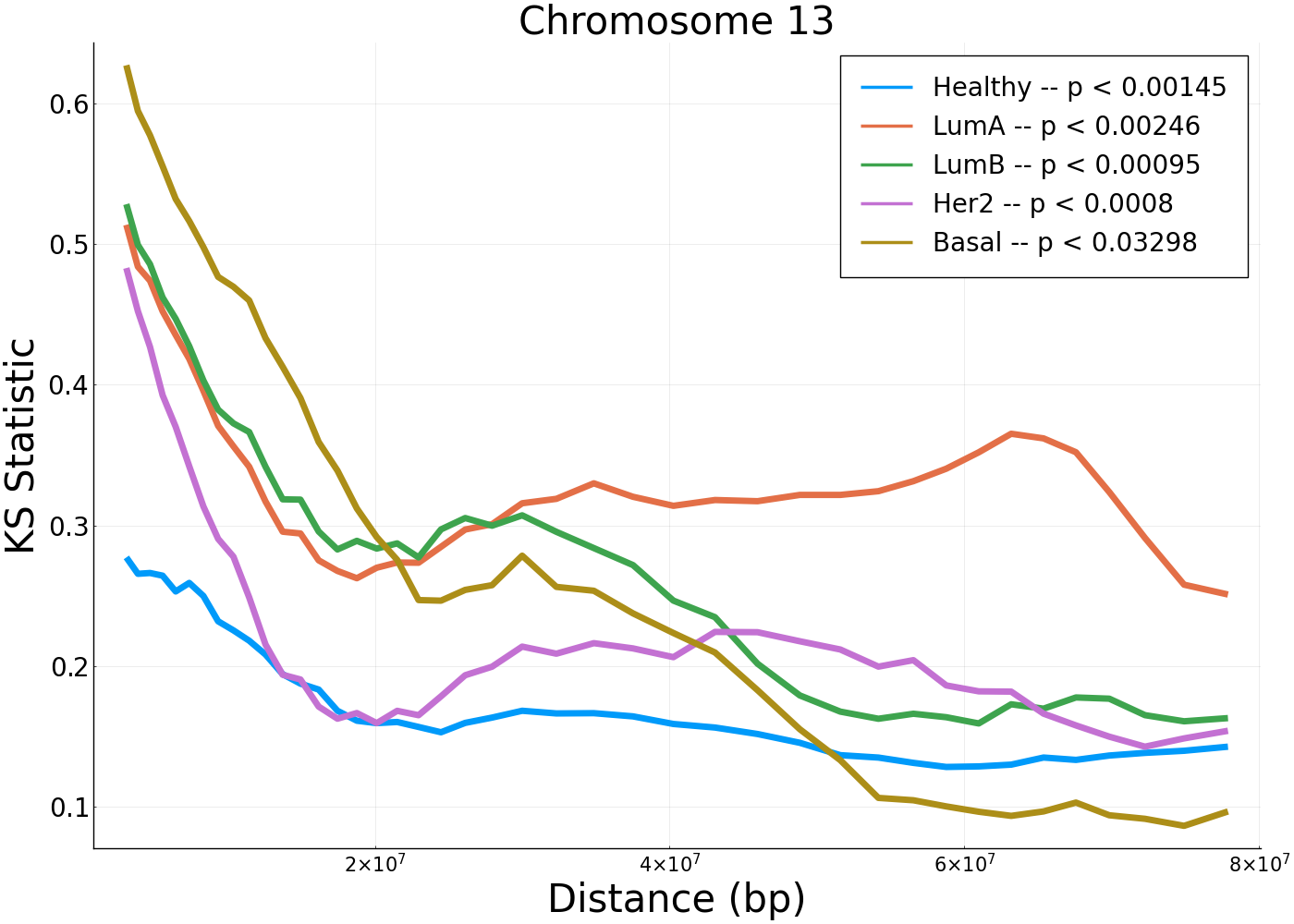

Supplement: Supplementary file 18 [file DataSheet_11.zip › SuppMat8/SuppMat8/KS-Test-Chromosome-13.png]

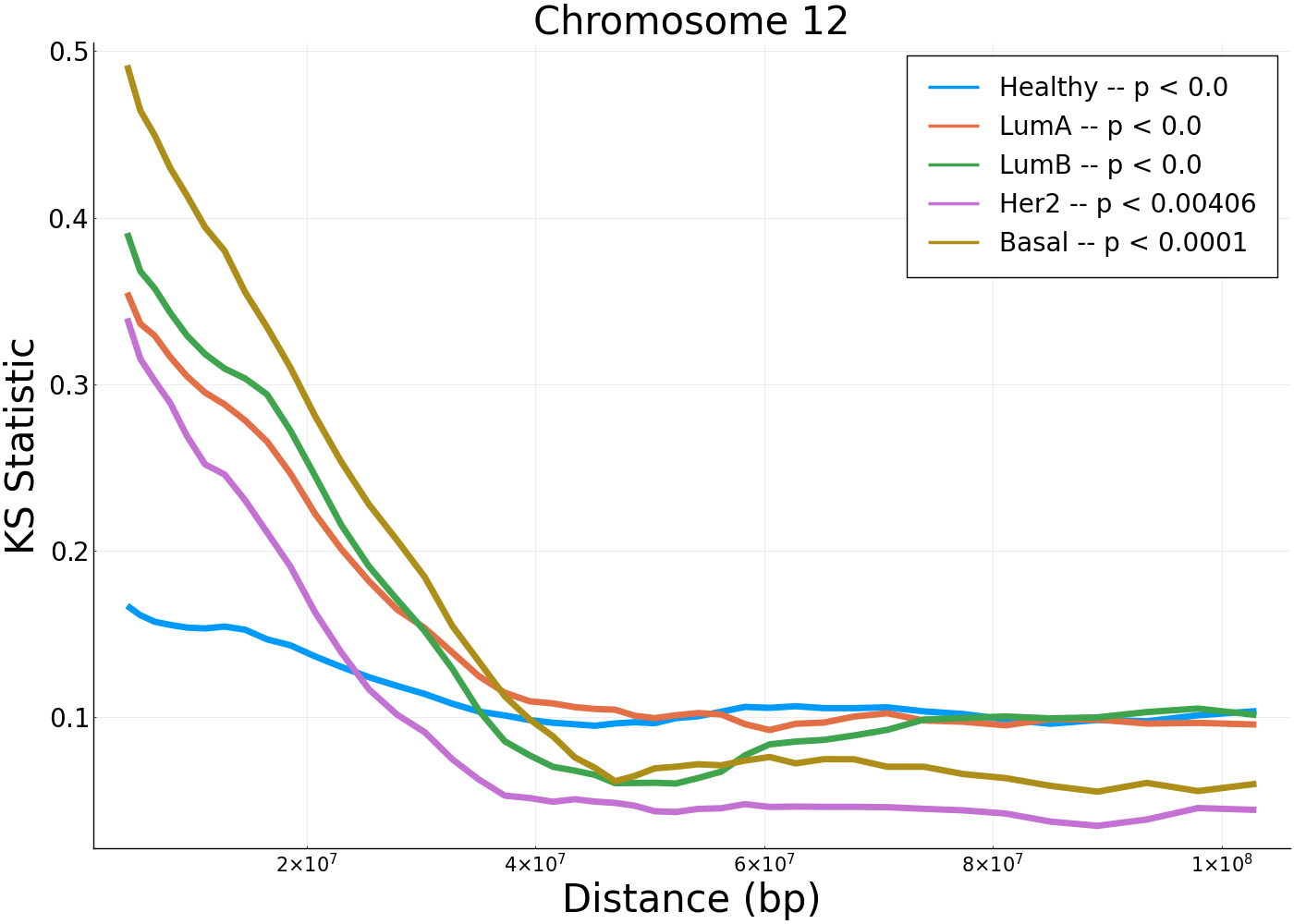

Supplement: Supplementary file 18 [file DataSheet_11.zip › SuppMat8/SuppMat8/KS-Test-Chromosome-12.png]
